# Human movement and gully erosion: Investigating feedback mechanisms using Frequency Ratio and Least Cost Path analysis in Tigray, Ethiopia

**DOI:** 10.1371/journal.pone.0245248

**Published:** 2021-02-05

**Authors:** Nadav Nir, Daniel Knitter, Jacob Hardt, Brigitta Schütt

**Affiliations:** 1 Institute of Geographical Sciences, Freie Universität Berlin, Berlin, Germany; 2 Department of Geography, Physical Geography, Christian-Albrechts-Universität zu Kiel, Kiel, Germany; Ghent University, BELGIUM

## Abstract

The cost of human movement, whether expressed in time, effort, or distance, is a function of natural and human related variables. At the same time, human movement itself, whether on land, air or sea, causes environmental cost. We are looking into the long-term environmental relationship of this interplay. Gullies—linear landforms, which dissect the landscape—are considered to be a cost for human movement, as they can form unpassable barriers destroying present path networks. On the other hand, human movement creates pathways, which flatten the surface and decrease the water permeability potential. This process results in runoff generation and possibly gully erosion. Accordingly, the spatial relationship between pathways and gullies is investigated. In the Tigray region of the Northern Ethiopian Highlands, gullies and pathways were mapped using remote sensing data. Frequency Ratio was used for assessing pathways as a variable affecting the location of gullies while Least Cost Paths were tested to evaluate the possible constraining impact gullies have on mobility. Based on these results, it is concluded that a positive feedback exists between the cost of human movement and gully erosion. We further discuss possible effects gullies may have had on trade, territory, and political affairs in Tigray. Consequently, we suggest that movement cost and gullying may not only hold strictly environmental or movement-related implications, but also socio-cultural ones.

## Introduction

The history of human movement, whether as individuals or groups, on a daily frequency or migrational scale, is constrained or encouraged by its cost, i.e. distance and its difficulty, requirements, and time [1]. Decision making processes involving the cost of movement are based on several social, economic, and environmental factors [2]. The first two of the cost categories, the social and economic constraints on movement, are usually the result of direct human involvement, although at times they originate from natural factors (e.g. resource distribution such as raw materials). In contrast, environmental costs of movement such as distance, climate and topography are not considered as a result of human activities. Investigations of both natural and socio-economical aspects of cost, have been in the focus of several scientific disciplines with geography frequently taking the lead, as early as the 19th century, with ‘laws of migration’ addressing distance and growing urban areas. In the early 20th century, distance and cost which were based on the location of raw material, became essential for productivity [3, 4]. Later works looked into commuting opportunities as a factor affecting employment in cities [5]. Physical distance between individuals, societies and countries still holds great importance and relevance in a modern globalized economy [6]. This is even more evident when we investigate historic and prehistoric societies [7].

### Movement cost in historical perspective

In archaeology, various attempts have been made to evaluate the cost of movement: (a) research on Palaeolithic hunter-gatherers, takes ethnographic mobility patterns, distribution of raw material, and availability of game into account [8–10], while (b) in the studies of complex societies, movement cost is more focused on trade and international relations [11, 12]. These types of inquiries into human history would take topography, a presumably natural constraint, as a vital category in cost calculations (e.g. Tobler’s hiking function, Uriarte Gonzalez’s slope-dependent cost function, Herzog’s metabolic cost function; [13–17]). Using these topography-based cost theories to apply a Least Cost Path (LCP) calculation between two points, is at the heart of historical human movement investigation [12, 18]. In one early LCP study, discrepancies between topographic based LCPs and real-life pathways, has been interpreted as related to metabolic costs [13, 15]. More recently, topographic based LCP calculation was insignificantly different from real-life pathway selection and gives good estimations of kilocalorie expenditure [19]. While calculating cost of movement, rivers as lines of broken traffic have a considerable movement cost [20, 21]. Gully erosion, that has long been correlated with land degradation by human activities [22], triggers the development of deeply incised stream-like landforms resulting in a possible barrier-function to movement.

### Gully erosion and pathways

Erosion is a natural process directed towards a levelling of relief, reaching a dynamic equilibrium when environmental factors remain stable. Extreme events can disturb this dynamic equilibrium [23]. Settlement activities result in a decrease of the thresholds for these erosional processes, promoting accelerated erosion [22]. The resulting processes include removal, transport and deposition of sediments or soil particles by water with on-site effects by inter-rill, rill, and gully erosion. Inter-rill erosion results in sheet wash where thin layers of surface substrates can be removed over larger areas [24]. In contrast, rill erosion is caused by concentrated flow in relatively small channels (depth < 30 cm) [25]. If there is no tillage, and the incision exceeds 30 cm, the process is termed gully erosion, and the resulting landforms are termed gullies. Hence, gullies are linear soil erosion features with a minimum depth of 30 cm or a minimum cross-section area of 930 cm^2^ and a minimum volume of 25–45 m^3^ [26–29]. Frequently, gullies are only a few hundred meters in length with an upstream gully-head where erosion begins and a downstream outlet, at times coinciding with fan accumulation at a break of slope [23, 30]. Gullies can develop during one extreme rainfall event, resulting in surface runoff corresponding to a flash-flood and further deepen and destabilize during later events of various intensities [31]. The global distribution of gullies is vast, with gullies occurring in most environments [32]. Especially bare surfaces caused by agricultural and pastoral practices are considered as favourable for gully erosion [33, 34]. Highly susceptible substrates include loess, marls and sandy deposits [25].

Gullies have been investigated from several perspectives with a focus on severe global soil loss problems and agricultural productivity [28], but also for example as indicators for geomorphological activities on Mars [35]. From a mechanical perspective, the threshold of the amount and form of surface runoff necessary to initiate gully erosion is determined by soil porosity and permeability, its shear strength and the size, and slope angles of a catchment area [29, 33, 36]. These properties have been correlated with geological, topographic, climatic and human induced variables [28, 31] and resulted in several studies that generated Gully Erosion Susceptibility Maps (GESM) for certain areas [e. g. 37, 38].

Notwithstanding the above-mentioned variables, few recent investigations have looked into a possible effect of paved and unpaved roads and animal trails on surface flow dynamics [27, 30, 39–42]. Based on these works it is suggested that roads or pathways, resulting in compaction and thus reduced soil porosity and permeability, may be responsible for gully volume changes as well as for the formation of new gullies. Experimental work points out that road and pathway surfaces have significantly higher penetration resistance values than various types of agricultural fields. Based on this observation it has been conclude that footpaths can be the point of origin for soil erosion [43]. Hence, once the surface of a pathway is compacted and infiltration capacity is depleted, it might be the starting point for gully erosion triggered by an extreme rainfall event [39]. Two forms of pathways-related erosion may take place: (a) the gully might follow the pathway forming a holloway [the channelizing of pathway’s surfaces; 40] or (b) in inclined terrain where footpaths are diagonal to the slope’s angle, the gully develops perpendicularly to the footpath, where the concentrated runoff following the footpath overflowed its ‘banks’ [27]. While holloways have an important role in pathways and soil erosion dynamics, in the Ethiopian Highlands, gullies are more noticeable landscape features, as they can result in a deep and at times long dissection of their surroundings [27, 38].

### Movement cost and gully erosion

We consider that gullies, which are at least 2 m wide and deep and have steep flanks (box-shaped cross-profiles), are topographic obstacles that increase the cost of human movement. As the location of gullies is frequently determined by the location of pathways and roads, their occurrence is often associated with roads resulting from movement patterns of humans across the landscape. For assessing a possible positive feedback mechanism between gullies and the cost of human movement, we hypothesize that:

1. Human movement, resulting in unpaved and paved pathways, promotes gully erosion.
2. Gully erosion, resulting in box-shaped channels, is a barrier constraining oriented human movement.

Based on remote sensing data, the first hypothesis will be examined using the Frequency Ratio (FR) index, a tool to assess variables’ importance in determining gully locations [38]. For testing the second hypothesis, gullies are mapped throughout the studied areas and randomly distributed Least Cost Paths (LCPs) are generated, with the gullies simulated as uncrossable features for these LCPs. Assuming that longer paths result from the existence of gullies, these are measured and compared prior to and following the introduction of gullies. The Tigray region of the Ethiopian Highlands, where most people nowadays still move on foot, is a well-studied area for both gully erosion and long-term human interregional movement [22, 44]. Following this, four different areas in Tigray are selected as case studies for investigating the relationship between human movement and gully erosion.

### Study area

**Fig 1:**
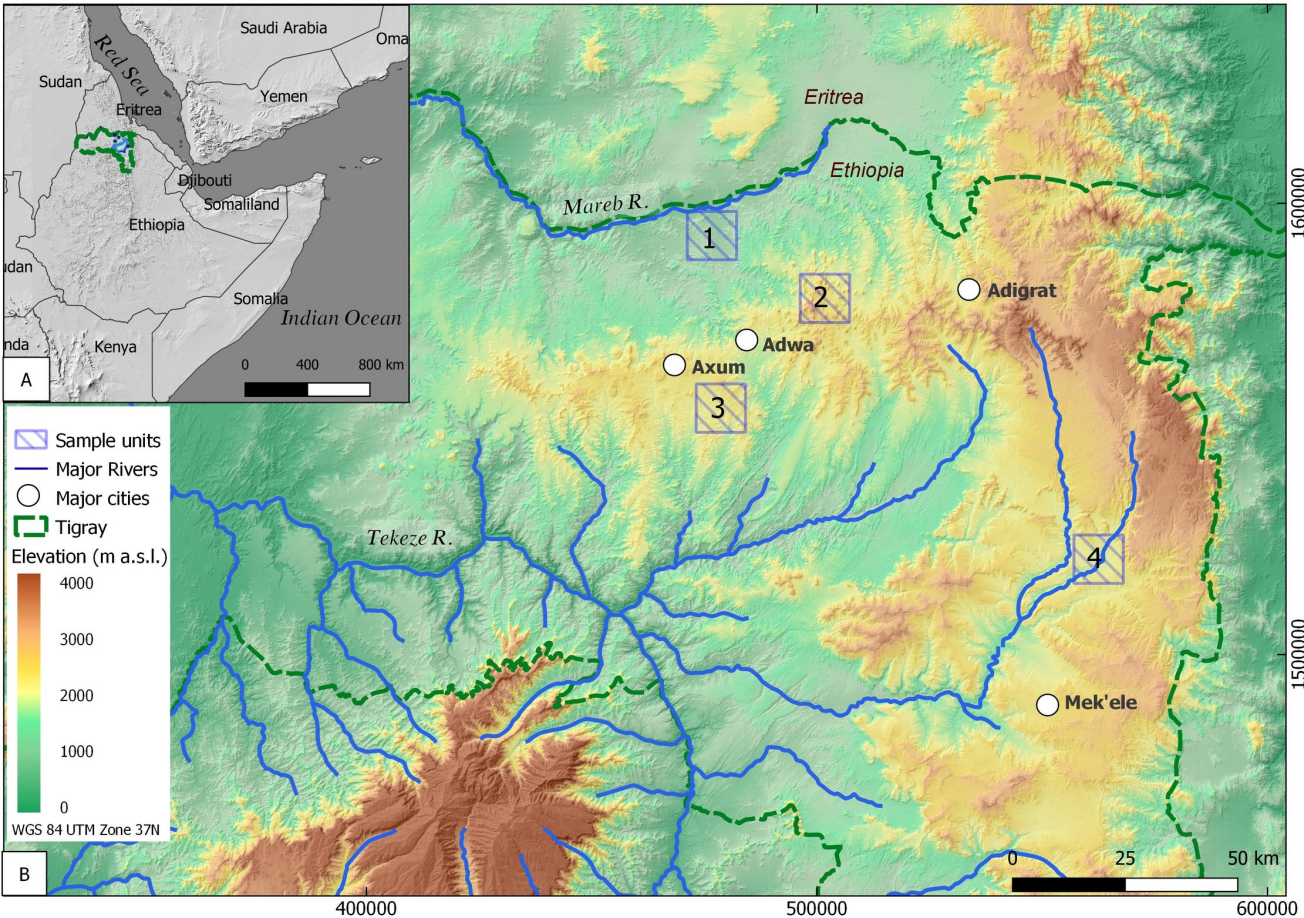
A: The Horn of Africa with the Tigray region of the Ethiopian Highlands marked in dash green line. B: The study area in central eastern Tigray. Numbered squares correspond to 10x10 km sample units: 1. Rama, 2. Yeha, 3. Daragà and 4. Wuqro. Sources: A: Natural Earth open source data available at naturalearthdata.com. B: color-coded hillshade map based on 30 m SRTM DEM provided by USGS earth explorer [81–83].

The Ethiopian region of Tigray comprises the northernmost part of the Ethiopian Highlands (Fig 1) with an area of more than 50,000 km^2^ and population nearing five million inhabitants [45–47].

### Environmental setting

The geological structure of Tigray comprises a variety of magmatic and sedimentary rocks with Late Proterozoic crystalline basement rocks being the oldest. The Paleozoic Enticho Formation (Fm.), the Mesozoic Adigrat Fm. (terrestrial sandstones), and the Mesozoic (Jurassic) Antalo Fm. (limestone formed under shallow marine conditions) occur widespread [48]. Oligocene volcanic activities of the Afar plume triggered large-scale emplacement of trap basalts [49]. Later volcanic activities in the Oligocene and Miocene resulted in plugs and domes, which pierce the modern surface [50]. Several planation surfaces are the result of a number of uplift phases triggered by tectonic movements of the Arabian-Nubian Shield. These are responsible for the stepped (locally termed “Amba”) landscape of the region [51]. Geomorphologically, the area can be subdivided into different landscape units: 1. cone shaped hills/mountains (volcanic plugs and domes), 2. flat to hilly areas of various sizes (plateaus and “Ambas”) and 3. depressions (most likely grabens). Drainage of these units is dominated by small valleys, large ephemeral streams, and few perennial streams with the Tekeze river being the major one in central Tigray [46]. Gullies can be found in all these geomorphological units [27].

The main perennial streams draining central Tigray are tributaries to the Tekeze River, a tributary to the Nile River; while few streams are tributaries to the Mareb (Gash) River, an episodic river which during extreme flood events may also serve as a tributary to the Nile. In general, these perennial streams are deeply incised forming steep valley flanks. Today’s climate in Tigray is tropical and driven by the monsoon. In eastern and central Tigray, which is in the focus of this study, the mean annual precipitation during the 20^th^ century varied between 500–800 mm [52]. Due to the influence of the monsoon three main seasons can be outlined: Rainy season corresponds to the monsoonal season and lasts from June to September (*kremt*), a dry cold season occurs from October to February (*bega*) and a pre-monsoonal warm period with little rainfall lasts from March until May (*belg*). Correspondingly, during the course of the year rainfall shows a bimodal distribution, composed of a smaller maximum from March to May and a larger maximum from July to October. During the main rainy season (*kremt*) rainfall occurs within massive stormy events rather than being gradual or persistent over time [53, 54]. Paleoclimate reconstructions suggest past colder conditions, with a possible increase by 7°C of the annual average since the Last Glacial Maximum (LGM) [22]. Based on sediment sequences in Lake Tana, Lamb et al. (2007) reconstructed three dry phases at 16.700, 15.100, 12.000 and one at 8.000 cal BP within a relatively more humid environment compared to the current one [55]. Bard et al. (2000) and Marshall et al. (2009) emphasize that since the mid-Holocene (ca. 5.600 cal BP) the Ethiopian Highlands experienced a dry-humid-dry cycle, with several periods within this cycle resembling present day climate [56, 57]. Furthermore, Nyssen et al. (2004) conclude that the current rainfall distribution pattern has existed since the mid-Holocene [22].

Current vegetation in the area is composed of a shrubs and savanna mix. In 2005 about 43.2% of the total Tigray area (~ 5 million ha) was covered by low woodlands and shrublands while high woodlands and forests composed about 0.2% of the vegetation cover in Tigray [58]. The nearly total absence of primary forests is assumed to also be related to the intensive and long-lasting land use in the whole Tigray area [22]. Land use in Tigray follows millennia long traditions with grains being the major cultivated crop. Today, agricultural areas of various types (e.g. tef (*Eragrostis tef)*, plantations and pastoral areas) occur area-wide [47]. The main type of settlement are villages along the major roads with few of them being medium sized towns (up to 50,000 inhabitants). The larger cities with administrative functions are Mek’ele, Adigrat and Aksum (Fig 1). Starting from the 1990s, a strong growth of urban areas occurred coinciding with road constructions [30]. In the rural areas the construction of numerous micro-dams and the intensification of agricultural production has taken place during this period [47, 59]. Multiple soil conservation measures are applied to prevent soil erosion, mainly using earth bunds, furrows and grassed lynchets (locally known as *daget*). Placing stone bunds and planting trees and bushes to stabilize larger earth bunds has been implemented since the early 1990s [30, 60–63].

### Archaeology

While the Afar and Omo regions hold the earliest evidence for genus *Homo* and a central piece in the global human evolutionary puzzle, substantial archaeological findings in the Ethiopian Highlands and Tigray only appear at the late Holocene with early chiefdoms and state formation [44, 52, 64–66]. Recent detailed surveys produced very few remains of Stone Age (Early, Middle and Late) material culture [11]. Intense archaeological research in Tigray has yet to produce a clear cultural chronology during the middle to late Holocene phase. Among other reasons, this is attributed to vast variations in the material culture and what appears to be a very gradual and spatially diverse transition from foraging towards nomadism and to farming [67, 68]. First evidence of hierarchical societies in the form of monumental temples appeared in the early 1^st^ millennium BCE [44, 68]. However, it has been suggested that even later, there was no clear preference for a certain central place in the Ethiopian highlands [11]. Architectural style and inscriptions brought the suggestion that the early 1^st^ millennium BCE material culture originated from an interaction of local and Arabian-peninsula originated elites. Some scholars name this cultural interaction D’MT or ‘Ethio-Sabean’, indicating a cultural exchange with the kingdom of Saba around 700 BCE [69]. However, in the Eritrean Highlands as well as in other archaeological sites in Tigray dated to this period, local rather than ‘Ethio-Sabean’ material culture is evident (‘Ancient Ona culture’). This may indicate diversified and regional cultural practices [52, 70]. During the first century BCE, the kingdom of Aksum appeared. It would become the defining culture of Ethiopia’s history, famous for its massive steles. The Aksumite, based with its capital in the city of Aksum in central Tigray, governed the highlands for most of the first millennium CE. In addition to far reaching international trade, they formed some of the governing basis for the modern states of Ethiopia and Eritrea [44, 68]. Continued urban presence in Tigray is evident during the post Aksumite period (ca. 10^th^-18^th^ century CE) from medieval European descriptions as well as a 15^th^ century chart, placing the city of Aksum at the centre of local hierarchy [12, 44].

### The sample units

**Fig 2:**
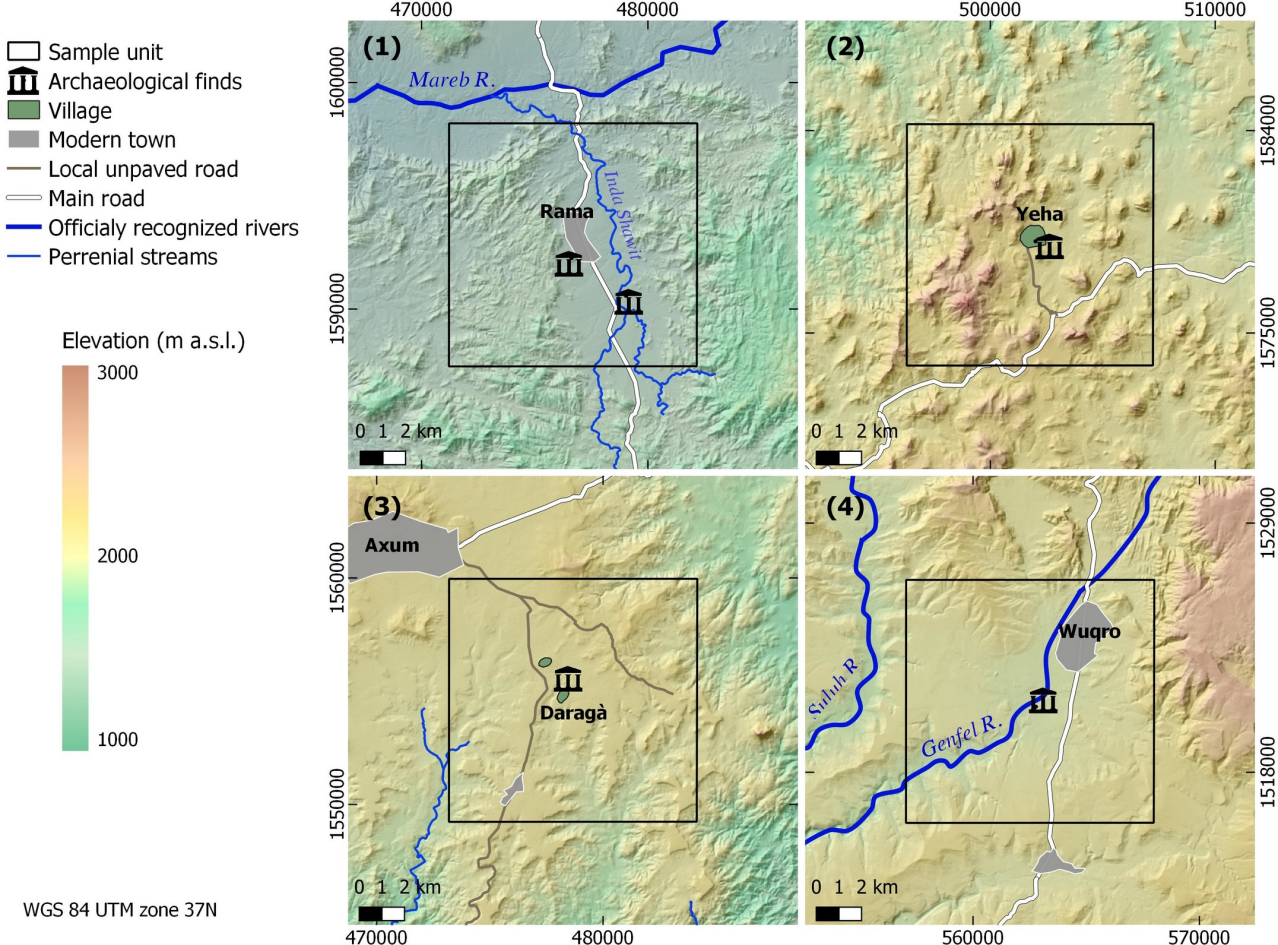
**Topographic overview of the four sample units: Rama (1), Yeha (2), Daragà (3) and Wuqro (4).** Color-coded hillshade maps based on 30 m SRTM DEM provided by USGS earth explorer [81–83].

**Fig 3:**
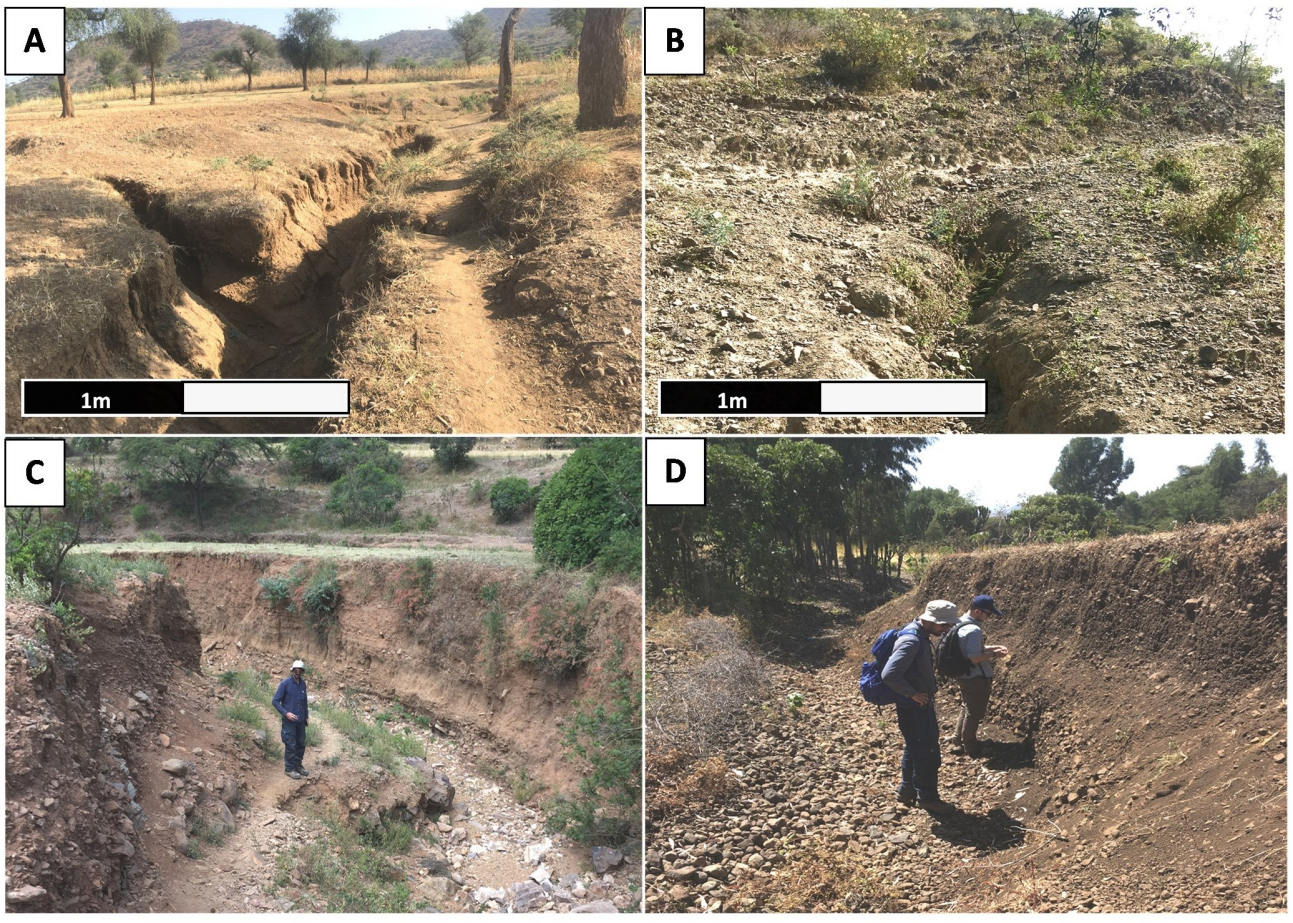
A. Gully along pathway in the Rama sample unit (1), B. Initial gully head development perpendicular to a pathway in the Rama sample unit (1), C. Gully in the Yeha sample unit (2) with a small pathway descending into it, D. Gully in the Daragà sample unit (3).

Four historically and environmentally representative sample units were chosen in order to investigate gully occurrence and indicators for human movement. It should be noted that even today, many people cross these areas on foot and no pedestrian bridges above these deep gullies were evident. Sample units were oriented around regions containing Ethio-Sabean or Aksumite archaeological sites—holding evidence of long-time human occupation. These sample units also represent a range of climatic, topographic and lithological characteristics in Tigray. To allow comparability between the sample units and due to calculation capacity limitations, a fixed plot area of 100 km^2^ was chosen for each of the four sample units (Figs 2 and 3).

#### Rama sample unit (1)

The Rama sample unit (#1 in Fig 2) is located in the Rama depression, which in the sample unit covers a 2 km wide and approximately 10 km long area striking S-N. With an average elevation of 1300 m a.s.l., the Rama sample unit is lower and warmer than the hills and ridges surrounding it. The Rama depression is most likely a graben flanked by a hilly area with moderate rise to the west and rising ridges with steeper slopes and higher elevation differences to the east; to the south the graben structure discontinues, as indicated by a number of faults appearing as ridges with steep slopes. North of the graben lies the Mareb River, flowing W-NW towards the Gash delta in Sudan and currently serving as the border river between Ethiopia and Eritrea. The lithology of the Rama sample unit is characterized by metasedimentary and metavolcanic Precambrian rocks (greywacke and sandstones) with granites and diorites as base rocks, exposed as flat and hilly granitoid units [45]. Patchy occurrence of epidote, quartz and calcite veins are also common. Faults are diverse with SW-NE as the dominating direction [71]. The Inda Shawit is a perennial river that drains the Rama graben to the north into the Mareb River (Fig 2). First and second order intermittent (mainly due to spring activity) to ephemeral channels drain the eastern and western graben flanks. Morphodynamics are characterized by intense soil erosion. Due to the high erosion intensity, with the exception of a fertile area on the floodplain of the Inda Shawit River, soils are highly degraded and locally the underlying bedrock is exposed. The area is intensively used for agriculture with only the patches of bare bedrock and badlands found uncultivated. Along the main channel citriculture and occasionally avocado plantations are alternating with the cultivation of various seasonal vegetables. Some slopes at the eastern and western graben flanks are terraced with olive trees (*Olea cuspidata*) and different grain crops including maize and sorghum as well as tef (*Eragrostis tef*) and finger millet [47, 59]. An unpaved road runs along the graben depression. At the centre of the Rama sample unit the modern town of Rama is located. Recent surveys of the area conducted by the German Archaeological Institute and by the Egyptian Museum Georg Steindorff of Leipzig University found graves and pottery attributed to the Aksumite period, while attempts to uncover pre-Aksumite occupation are ongoing [72, 73]. The Rama sample unit was defined around these archaeological features.

#### Yeha sample unit (2)

The Yeha sample unit (#2 in Fig 2) is located 10 km south-east of the Rama sample unit (Fig 1B). The most prominent geomorphological features are the cone-shaped hills and mountains reaching up to 2,400 m a.s.l., forming the eastern extension of the so-called ‘Adwa Mountains’ [74]. Oligocene intrusive volcanic activities of the Afar plume formed phonolitic and trachytic plugs and domes that resemble the hilly-mountainous cone-shaped topography [50]. Next to the volcanic rocks, siltstones, sandstone (Adigrat sandstone) and metasedimentary rocks are abundant. A 500–1200 m wide SW-NE oriented basin with alluvial infills several meters thick crosses the eastern part of the Yeha sample unit, being drained by a box-shaped channel with periodical runoff. Several gullies formed in the alluvial infills draining directly into the box-shaped channel. Grassy mountain vegetation covers the uncultivated slopes while tillage areas occur in the flat areas of the basin infills, which are dominated by the cultivation of tef [75]. An unpaved road connects the main road of central Tigray with the village of Yeha, which lies in the centre of the Yeha sample unit. Archaeological evidence indicates almost three millennia long occupation of this area including a unique and well-known temple attributed to the Ethio-Sabean culture of the pre-Aksumite period [69, 76].

#### Daragà sample unit (3)

The Daragà sample unit (#3 in Fig 2) is located 16 km south-west of the Yeha sample unit (2) and 2.5 km south of the modern city of Aksum. The landscape of the Daragà sample unit is occupied by plains that are part of the Aksum plateau located at c. 2,000 m a.s.l. Two perennial streams incised up to 100 m deep into the Aksum plateau drain the area. The plateau surface is composed of Oligocene trap basalt and the sandstones of the Mesozoic Adigrat Formation [77]. Locally exposed bedrock as well as gullies are common, indicating that soil erosion characterizes present morphodynamics. The valley floors are infilled by alluvial deposits in which the modern channels are slightly incised. The plateau area is predominantly used for tef cultivation while the alluvial plains are used as grazing areas for cattle. In the cultivated areas fields are separated from each other by field stones piled up to small walls and frequently by cacti or endemic trees. Two unpaved roads or rather wide pathways cross the area along a N-S and NW-SE axis (Fig 2). The southern outskirts of Aksum expand south-eastwards along the northern edges of the Daragà sample unit, while a few small villages occupy its central part including the hamlets of Hawelti and Daragà. In their surroundings, substantial archaeological findings were uncovered, including the temple of Melazo attributed to the Ethio-Sabean culture [78–80].

#### Wuqro sample unit (4)

The Wuqro sample unit (#4 in Fig 2) is located 75 km south-east of Daragà and 82 km south-east of Yeha and lies about 40 km north of Tigray’s capital Mek’ele. In the northern part of the Wuqro sample unit, a local mountain range rises to 2300 m a.s.l. with the slopes drained to the south and the southwest by two valleys reaching the mainstream at c. 1900 m a.s.l. Geologically the unit belongs to the northern edge of the so-called ‘Mek’ele outlier’ composed of Paleozoic to Mesozoic dolomite-limestone [81]. An inverse fault crosses the area SW-NE and SE-NW trending normal faults appear in its southern part [48, 82]. The perennial Genfel River drains the area following the major fault lines and serves as a tributary to the Geba River. Gullies are a prominent geomorphological feature along the slopes. The area is intensely cultivated by cropping maize, sorghum and tef. An asphalt road crosses the Wuqro sample unit in N-S direction connecting the cities of Mek’ele and Adigrat. The town of Wuqro lies in the centre of the Wuqro sample unit. About 300 m south of Wuqro a temple was excavated holding significant evidence of Ethio-Sabean material culture including a libation altar with a Sabaean inscription mentioning Yeha [83].

## Materials and methods

### Data collection

Present-day gullies, pathways, and roads were mapped at a scale of 1:1500 using 1 m/pixel resolution satellite images provided by CNES/Airbus Maxar Technologies Map data @2020 as they appeared on Google Maps via the Quick map service extension in QGIS version 3.4.5-Madeira [84]. Due to the difficulty assessing gullies and differentiating them from other features based strictly on remote sensing, geomorphological units and gullies in the sample units were recorded during field work in October-November 2019. Sample unit (1) was extensively surveyed for in-depth measurements of gullies’ profile parameters. The validating field work involved measuring width and depth of gullies along different locations and later comparing these values to their visibility on the satellite image. A 30x30 m Digital Elevation Model (DEM) derived from Shuttle Radar Topography Mission (SRTM data, February 2000) was provided by United States Geological Survey (USGS) via the Earth Explorer internet portal [85, 86]. In order to evaluate possible preferred orientations of gullies, the mean average and mean resultant length for circular data were applied using the ‘mean.circular’ and ‘rho.circular” functions in R environment [‘circular’ package; 87–89].

### Variables affecting gully erosion

The following categories have been shown in previous studies to influence gully erosion: elevation, slope aspect, plan curvature, slope angle, Topographic Wetness Index (TWI), Normalized Difference Vegetation Index (NDVI), Land Use and Land Cover (LULC), lithology, soil type and distance from stream [23, 26, 37, 38, 90]. Except for lithology, data for all categories were obtained via open access resources. Derivatives of the 30x30 m DEM were calculated using QGIS with GRASS (Geographic Resources Analysis Support System) GIS version 7.6 [91] and SAGA (System for automated geoscientific analyses geographic information system) version 6.3 extensions [92]. NDVI was calculated after Zhang et al. (2017) applying QGIS raster calculator to Sentinel-2 satellite imagery data (T37PDR, T37PER; 2020/06/25) [93, 94]. LULC data were provided by the European Space Agency (ESA) Climate Change Initiative (CCI) based on Sentinel-2 data [95]. Soil types were obtained from the soil map of the African continent [96]. Drainage networks were obtained from the World Bank online water database produced in cooperation with the Ethiopian Ministry of Water Resources [97]. The parameter “distance from the stream” was calculated subsequently [37, 38], using the GDAL proximity raster extension for QGIS to generate 50 meters categories (intervals) on a total distance of 250 meters from the stream [98]. The 1:250,000 scale geological maps of the area (ND 37–6, ND 37–7) were acquired from the Ethiopian Geological Survey in Addis Ababa and manually digitized. To obtain an additional layer of information about the earlier distribution of pathways (before the 1974–1991 Ethiopian Civil War and subsequent land use changes), pathways and roads were digitized from the USSR topographic maps published in 1977 (1:200000; D37-13, D37-21, D37-28) [99]. Additionally, CORONA satellite images, produced by US Central Intelligence Agency (KH4B, 1967; DS1102-2106DF072_c) were geocoded and detectable pathways were mapped [85]. With the exception of lithology and soil, input layers were divided into categories and classes based on the natural distribution of the categories in the sample units with the intention that all areas would have at least 5 similar equally distributed intervals [37]. Input layers elevation, plan curvature, TWI, NDVI and slope angles were reclassified using ‘class’, ‘rcl.m’ (R basic package) and ‘raster’ package functions in R environment, based on the evaluation of their natural distribution range, using the histogram ‘hist’ function [87, 100]. Analysing distance intervals of 25 m (for pathways) and 50 m (for roads and streams) were used following results and assessments of previous studies [39, 40, 101], this also incorporated a category of ‘outside the range’ following Roy and Saha (2019) [37]. Occasionally, additional edge intervals, i.e., classes beyond the equal interval classes (see Table 1), were formed to incorporate the entire range for each variable and avoid loss of information. Slopes’ aspects were categorized into eight intervals of intercardinal directions with additional ‘flat’ areas lacking a distinct aspect demarcated following Arabameri et al. (2018) [38]. Input layers for lithological units and soil characteristics have been categorized according to their natural features’ distributions. All non-pixel-based variables and all gullies were rasterized in the QGIS environment using SAGA [92].

**Table 1:** Classes distribution of the different natural, human and movement related variables tested for FR calculation of relative gully occurrences.

Variables		Elevation* m a.s.l.	Slope	Aspect	TWI	P.curvature	NDVI	LULC	DFS [m]	DFR [m]	DFP [m]	DFPSM [m]	DFPCI [m]
Classes	1	1800–1900	0°-5°	Flat	5–7	(-100)—(-10)	(-0.2)- 0.0751	Open water	0–50	0–50	0–25	0–50	0–25
	2	1900–2000	5°-10°	N	7–9	(-10)—(-3)	0.075–0.125	Built	50–100	50–100	25–50	50–100	25–50
	3	2000–2100	10°-15°	NE	9–11	(-3)—(-2)	0.125–0.175	Bare	100–150	100–150	50–75	100–150	50–75
	4	2100–2200	15°-20°	E	11–13	(-2)—(-1)	0.175–0.225	Sparse Veg.	150–200	150–200	75–100	150–200	75–100
	5	2200–2300	20°-25°	SE	13–15	(-1) - 0	0.225–0.275	Aquatic Veg.	200–250	200–250	100–125	200–250	100–125
	6	>2300	>25°	S	15–17.5	0–1	0.275–0.325	Cropland	>250	>250	>125	>250	>125
	7			SW		1–2	0.325–1	Grassland					
	8			W		2–9		Shrubs					
	9			NW		9–100		Tree cover					

TWI—Topographic Wetness Index, DFS—Distance From Stream, DFR—Distance From Road, DFP—Distance From Pathways, DFPSM—Distance From Pathways on Soviet Maps, DFPCI—Distance From Pathways on CORONA Images, NDVI—Normalized Difference Vegetation Index, LULC—Land Use Land Cover (as processed and described by the European Climate Change Initiative), P. Curvature—Plan Curvature with convex values represented as positive and concave values as negative. Notice variables lithology and soil type are missing as they were divided into classes according to the distribution within each sample unit based on soil and lithological maps.

*for Rama (1) elevation starts at 1100 m a.s.l and continues in similar 100 m intervals.

### Frequency Ratio calculation

Frequency Ratio (FR) is a normalizing tool set to assess the relative frequency of gullies within each of the categories of the classified variables; it was calculated using R software [87]. Based on pixels of each variable, the following equation was applied (Eq 1):

$$FR=\frac{\left(\frac{g}{C}\right)}{\left(\frac{G}{S}\right)}$$ (1)

where *g* is the number of gully pixels for a class of a variable, *C* is the total number of pixels of that class, *G* is the total number of gully pixels within a sample unit and *S* the total pixels composing the sample unit following Arabameri et al. (2018) [38]. If gullies are not at all affected by a given variable, a FR value of 1 is expected within all its classes. Any value >1 indicates concentration of gullies in a class area is higher than an expected non-variable-related distribution in the overall sample-unit. If a class’s FR is < 1, the relationship between the variable and gullies is weaker and gullies are relatively more abundant outside this class [102–106]. FR values are asymmetric; hence a strong relationship could be indefinitely higher than 1 while a weak relationship is limited to the range of 0–1. Therefore, the other variables, which have been used to produce accurate Gully Erosion Susceptibility Maps (GESM), have been evaluated to monitor the relative significance of the pathways and roads for the occurrence of gullies [37, 38]. Additionally, a randomly produced image holding the same number of pixels as do gullies for each sample unit, was generated and calculated for its FR in the pathway category, to avoid overinterpretation of the effect of pathways on the location of gullies.

### Cost of movement

The effects of gully formation on the development of the pathway network were assessed by applying Least Cost Path algorithms. The first set of LCPs were calculated on the basis of a normal (unmodified) DEM. Due to its 30m resolution, this DEM does not incorporate the gullies, which are usually not wider than 15-20m. The second set of LCPs were calculated based on a modified DEM where gullies were artificially inserted as barriers. Vectors of mapped gullies were transformed into 5 m wide pixels using the ‘Rasterize’ function in QGIS [92]. In order for the gully pixels to be represented, the DEM available in 30x30 m resolution was resampled into 5x5 m resolution using the ‘resample’ function in R and the ‘raster’ package [100] for both unmodified and modified (i.e. non-gully-added and gully added) layers. Applying a nearest neighbour resampling method ensures obtaining reliable results for cost analysis [107, 108]. The modified DEM expressing gullies as obstacles to movement (corresponding to topographic walls of height 99,999 m) was generated in R using ‘base’ and ‘raster’ packages [87, 100].

Least Cost Path (LCP) analysis is a well-established tool to compute a preferred movement route between two points and is mostly based on topographic features [e.g. 13–18, 101]. Before calculating LCPs, elevation differences in the resampled DEMs were transformed into a conductivity transition layer in R environment. Conductivity was based on the ‘Wheeled’ cost calculation (Eq 2):

$$1/(1+((abs(x[adj])*100)/critical\;slope{)}^{2})$$ (2)

The *x[adj]* stands for slope angle as rise or descend for adjacent cells. Commonly used critical slope gradients are in the range of 8–16% [16, 17]. In the current work, a critical slope gradient of 12% was applied as a threshold, meaning that the paths are trying to avoid moving along slopes that have steeper inclinations than 12% [14, 16, 21]. The resulting conductivity transition layer stores the effort of movement from a source to a target pixel, applying Dijkstra’s algorithm [13, 16]. Target pixels are adjacent pixels that are defined by either a 4 (rook), 8 (queen), or 16 (knight) pixel neighbourhood, representing either horizontal, vertical or diagonal movement [16–18, 21]. This effort is represented as conductivity, i.e. the ease of crossing. Therefore, in the presented case, the conductivity results from a calculation that is determined by the slope gradient, applying the 12% threshold, between the source and target pixel. As the gully-representing values in the modified DEM are maximum 2 pixels wide, i.e., 2 squares in the transition layer, adjacency of 8 was chosen to minimize potentially topographic related ‘jumping’ over a gully pixel [109, 110]. For the LCPs to cross as much of the sample unit’s surface as possible, the units’ northern, southern eastern and western edges were chosen as areas for goal and origin of the randomly generated LCPs. These edge areas were extracted in R using the ‘Extent’ function to select 1 km wide rectangular strips along the four edges of each sample unit [87]. Following this, using the ‘spsample’ function (‘sp’ package), random goal and origin points were generated within these 1 km long and 10 km wide strips [111–113]. Applying separate loops for north to south LCPs and east to west LCPs, this process was repeated 1000 times for each orientation and sample unit to obtain meaningful results (see S2 File for R code). A High-Performance Computing cluster at Freie Universität Berlin was used to calculate the random LCPs.

## Results

### Field observations

**Fig 4:**
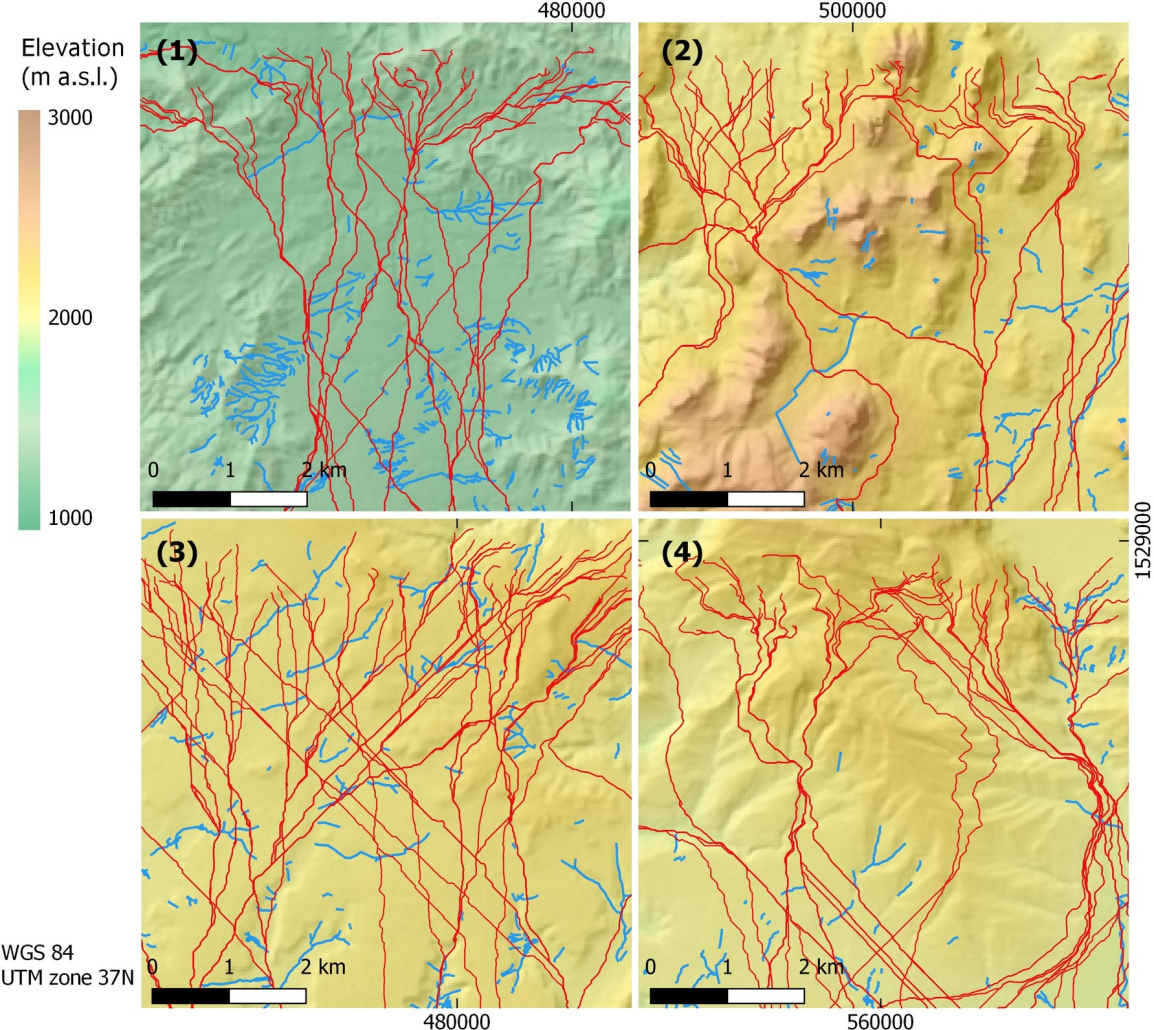
**Gullies (blue lines) and randomly distributed Least Cost Paths (red lines) within the four sample units: Rama (1), Yeha (2), Daragà (3) and Wuqro (4).** Figures are showing the north-central section of each unit for comprehensible view of LCPs’ starting points as well as the gullies’ distribution in the landscape. Color-coded hillshade maps based on 30 m SRTM DEM provided by USGS earth explorer [81–83].

Gully erosion occurs across all landscape types of the four sample units. Gully networks including first, second and third order gully channels occur more frequently close to perennial or periodical streams while isolated gullies occur commonly in agricultural fields and along slopes. V-shaped gullies start predominantly mid-slope and frequently expose the local bedrock at their base. These usually reach the foot-slope and at times continue within the plains. In the plains, box-shaped gullies are the more typical form, though few v-shaped, usually narrower than the box shaped gullies, also occur. Where vegetation cover occurs, gullies are less frequent; this observation was especially made for the v-shaped gullies occurring along slopes. In the Wuqro sample unit (4) some gullies observed in upslope areas formed along fault lines. Gully distribution was not uniform across the sample units with the Rama (1) and Daragà (3) sample units exhibiting the highest number of mapped gullies (Table 2). Regarding possible overall patterns, it is evident that gullies’ lengths and locations vary between and within the sample units in both network and isolated forms. Many gullies exhibit a NW to SE direction. However, low mean resultant length values indicate a lack of unidirectional distribution pattern (Table 2). Some gullies are confined by valleys and seem to be predominantly shaped by topography, while others occur on the plains and along slopes (Fig 4). In the Daragà sample unit (3) the highest absolute number of holloways was recorded, composing 11% of its gullies, a ratio similar to the Wuqro (4) sample unit (Table 2).

**Table 2:** Distribution of gullies and holloways in the study area.

Sample Unit	Total number of gullies	Total length of gullies	Gully Density	Mean average**	Mean resultant length**	Total number of holloways	Holloways share in gullies
		[m]	[m^2^]*				[%]
1 Rama	880	163,775	4.2^−6^	64.47°	0.112	8	0.9
2 Yeha	553	86,484	1.56^−6^	107.29°	0.125	22	4
3 Daragà	905	199,230	3.37^−6^	106.01°	0.182	107	11
4 Wuqro	368	77,733	1.48^−6^	91.25°	0.311	41	11

*Gully density was calculated after mapping and transforming vectors into pixels (‘Rasterize’) for each sample unit. Following which, the amount of gully pixels was divided by the sum of all pixels of that sample unit [QGIS; 37, 84, 92].

** Mean average (degrees east of north) and mean resultant length where calculated using circular statistics [‘circular’ package; 87–89]. Mean resultant length is a precision evaluation between 0 and 1, where 0 indicates that the spread of gullies’ orientations is wide (multidirectional) and 1 means that all gullies are oriented towards a single direction (unidirectional) [88, 89].

### Frequency Ratio (FR)

**Fig 5:**
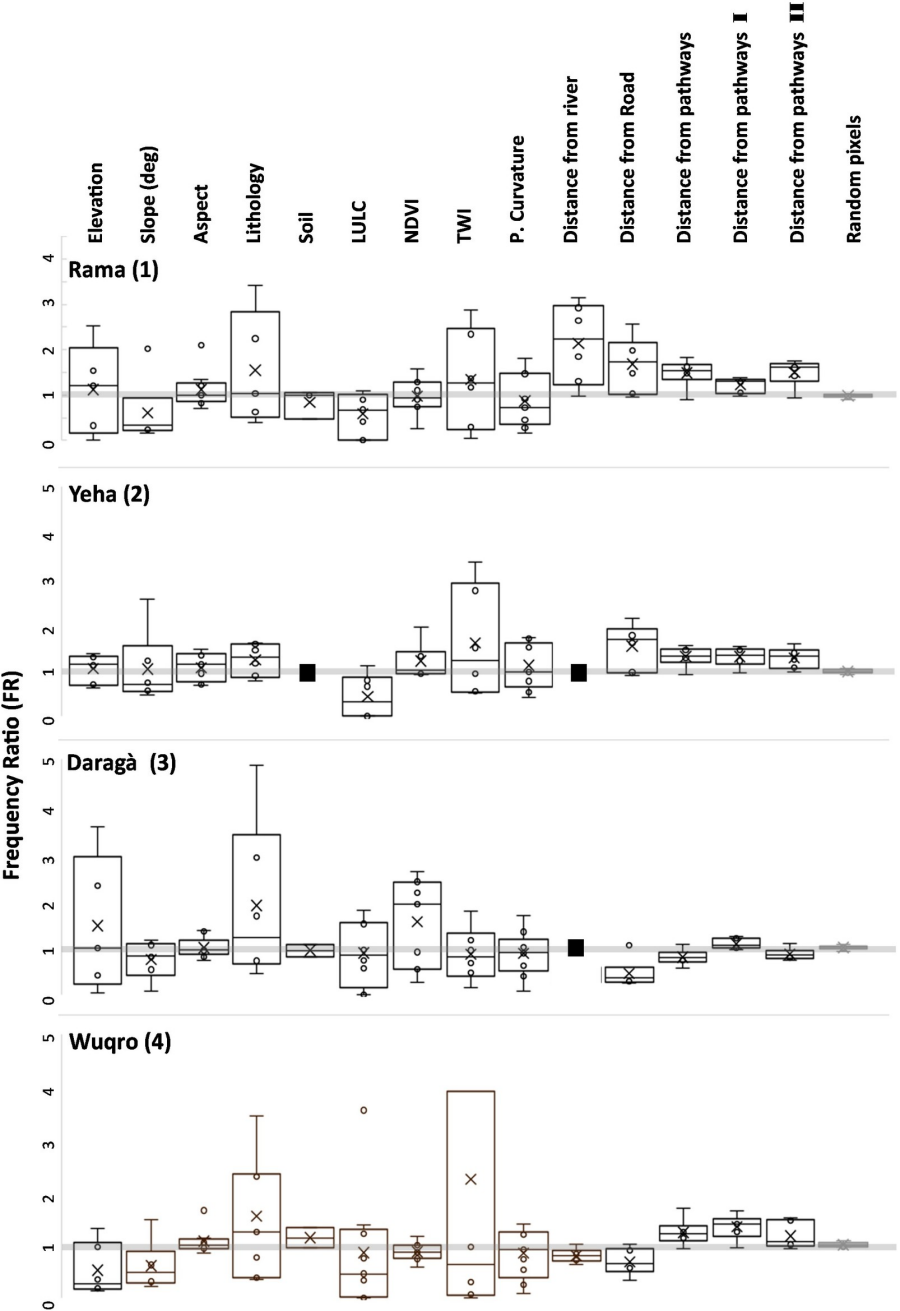
Frequency Ratio (FR) box plot diagrams of all classes within a given variable. Boxes represent 50% of FR distribution. Median is marked by a horizontal line while arithmetic mean marked by X. Circles represent the FR values whenever they were not used to generate the box plots borders itself. LULC—Land Use Land Cover, NDVI—Normalized Difference Vegetation Index, TWI—Topographic Wetness Index, distance from pathways I–based on Soviet topographic maps, distance from pathways II–based on CORONA satellite images. Black squares are due to irrelevance of the variable for a given sample unit, i.e., one soil type at Yeha sample unite (2) or no officially mapped rivers in the vicinity of the sample units (2) and (3). Random pixels tested for their relative frequency in the ‘distance from pathway’ variable are marked in light grey at the right edge of the plot. For TWI in the Wuqro sample unit (4), highest FR is 10.4 and is not presented due to graphic limitations (see S1 Table for full results).

Different natural and human related variables with a possible effect on determining the location of gullies were systematically collected and each of these variables were sub-divided into their own classes (Table 1). Fig 5 incorporates the Frequency Ratio (FR) of all classes of a given variable, i.e., one box plot per variable. As each class holds one FR value (e.g. a certain lithological unit is a class with FR = 2 while another unit composes another class with FR = 1.2). Notwithstanding the asymmetric nature of FR, all classes of a given variable were plotted together to illustrate the spread of values and the distance from a ‘non-affecting’ FR value of 1. The distribution of gullies across the different sample units differs from a non-variable-related FR of 1. Therefore, all variables are responsible for the location of gullies to various extents (Fig 5).

Due to the asymmetric nature of FR values, interpretation of deviations of the FR values from 1 could only be qualitatively and comparatively. Taking this limitation into account, it is evident that the four sample units show distinct FR value distributions which require a site-based analysis.

Among all variables, lithological characteristics dominate the location of gullies in the Rama (1) and Daragà (3) sample units. Topographic Wetness Index (TWI) is the most influential variable in the Yeha (2) and Wuqro (4) sample units. Slope gradient has little influence in the Daragà sample unit (3) while it has a relatively more dominant role in the Rama (1) and Yeha (2) sample units (Fig 5, S1 Table). For all sample units, the slope aspect exhibits only little influence on gully development, the same applies for the soil types. Some of the variables analysed, such as Land Use and Land Cover (LULC), show mainly FR values lower than 1, indicating that the amount of gully pixels in some classes is lower than expected from an equal distribution across space. However, the extent to which gullies are less likely to occur should not be over interpreted due to the asymmetric nature of FR. In the Wuqro sample unit (4), LULC has one class with a relatively higher FR value than the rest of the classes, indicating this class may have a more dominant role on determining the location of gullies (Fig 5). In general, movement related variables have weaker relationship with the occurrences of gullies than the non-movement related variables. However, random pixels equivalent to the number of gully pixels in each of the sample units were tested against the ‘proximity to pathways’ variable. This gives an estimation of the possible error size of a random distribution both for standard deviation and proximity to value 1. It is evident that movement related variables exceed the range of this distribution (Fig 5), indicating that the location of pathways and roads relates to the location of gullies. In the Rama (1), Yeha (2) and Wuqro (4) sample units, gullies are more likely to occur closer to pathways while in the Daragà sample unit (3), gullies are more likely to form outside the pathways’ proximity range (25 meters). Proximity to roads has a relatively stronger correlation with the location of gullies than the proximity to pathways in the Rama (1) and Yeha (2) sample units—while in the Wuqro sample unit (4) gullies are less likely to occur in proximity to roads (Fig 5). For pathways based on USSR maps from 1977, gullies are more likely to occur closer to pathways in all sample units (Fig 5).

### Least Cost Path analysis

**Fig 6:**
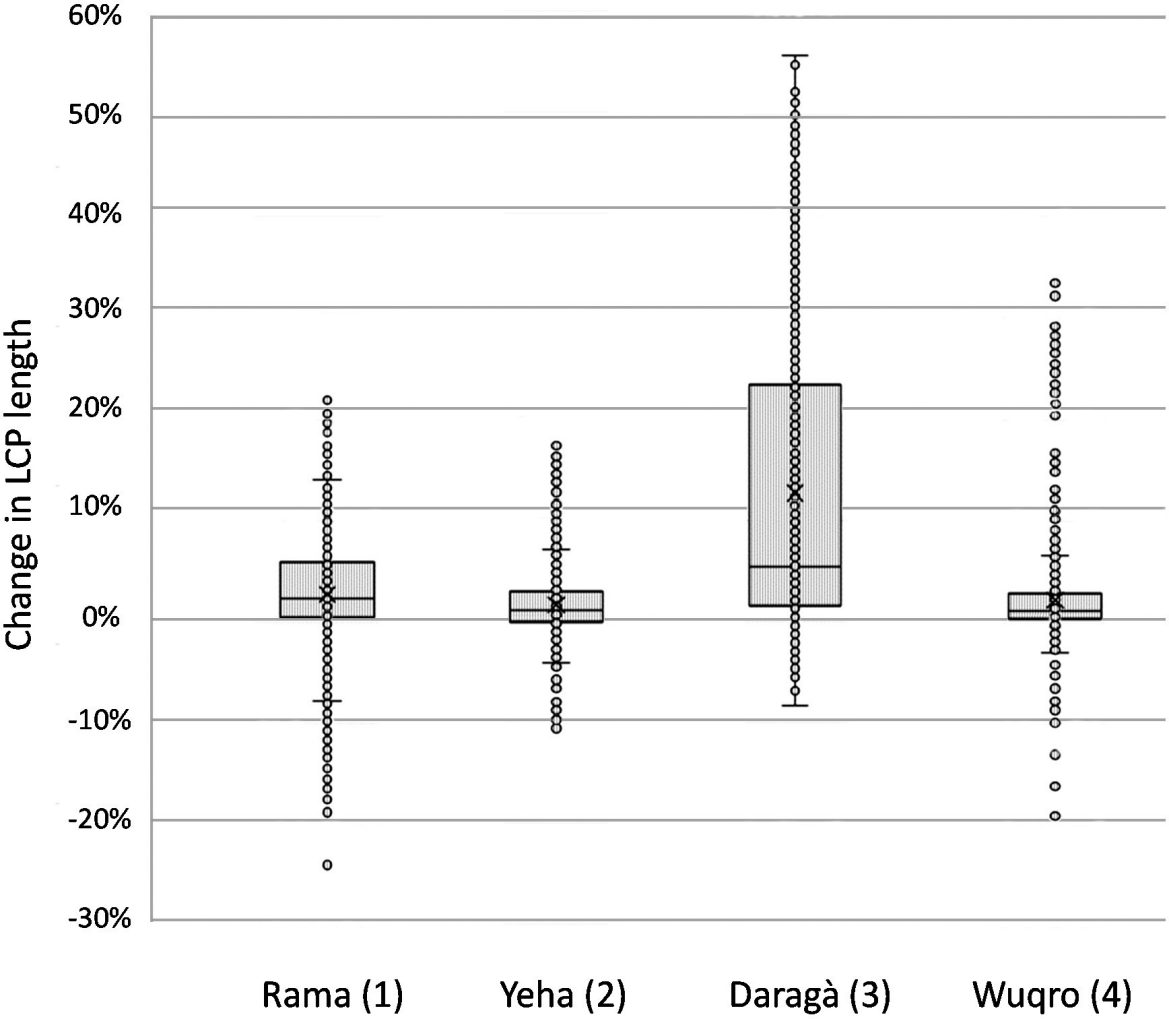
Changes in length of Least Cost Paths (LCPs) following the introduction of barriers along gully locations (‘modified DEM’). Change is expressed by differences in the length of Least Cost Paths generated between two random points across the four sample units. The length difference (LCP without gullies as barriers / LCP with gullies as barriers) is expressed in percentage along the Y axis. Box plot representing 50% of changed LCPs (median). Mean marked by X while circles represent each changed LCP (n = 2000 for each sample unit; See S2 Table for full results).

The randomly distributed Least Cost Paths cross gullies in multiple occasions as exhibited in the central-northern examples of the south to north LCP in all sample units (Fig 4). Changes in costs of movement, expressed here as changes in Least Cost Path lengths, are assessed after the implementation of high cost barriers at the location of gullies (Fig 6). Changed LCPs show a range of both increase as well as reduction in length from the unmodified LCP: The highest values reflect a 55% length increase compared to the unmodified DEM in the Daragà sample unit (3), following 34% increase in the Wuqro sample unit (4), 22% in the Rama sample unit (1) and a 17% increase in the Yeha (2) sample unit. In the Daragà sample unit (3), half of LCPs witnessed an increase of between 1.7–23.4%, while for the Rama (1), Wuqro (4) and Yeha (2) sample units, half of the LCPs were longer by 0.5–6%, 0.4–2.9% and -0.1–2.8% accordingly.

Although most of the LCPs got longer when integrating gully barriers into the DEM, some LCPs exhibited even shorter paths after being ‘blocked’ by gullies (negative values in Fig 6). LCPs that became shorter account for 22% of the Daragà sample unit (3), 38% of the Rama sample unit (1), 25% of the Wuqro sample unit (4) and 55% of the LCPs in the Yeha sample unit (2). The most extreme path length shortening was witnessed in the Rama sample unit (1) with a maximum reduction of 24% of the path length compared to the unmodified DEM. In the Wuqro sample unit (4), maximum length reduction values is 19% while in the Yeha (2) and Daragà (3) sample units, the extreme values for LCPs length reduction were 11% and 8% accordingly (Fig 6). After assessing the validity of the algorithm, it was evident that a topographic threshold, that was too high to cross before the introduction of gullies, has been considered as worth crossing due to the ‘blocking’ of other possible routes by the uncrossable gullies. This was due to either equal values in the cell’s ‘neighbourhood’, causing the selection of cells whose cumulated length is not optimal (though their conductance is) or to a change in the transition matrix used as input for the shortest path algorithm.

**Fig 7:**
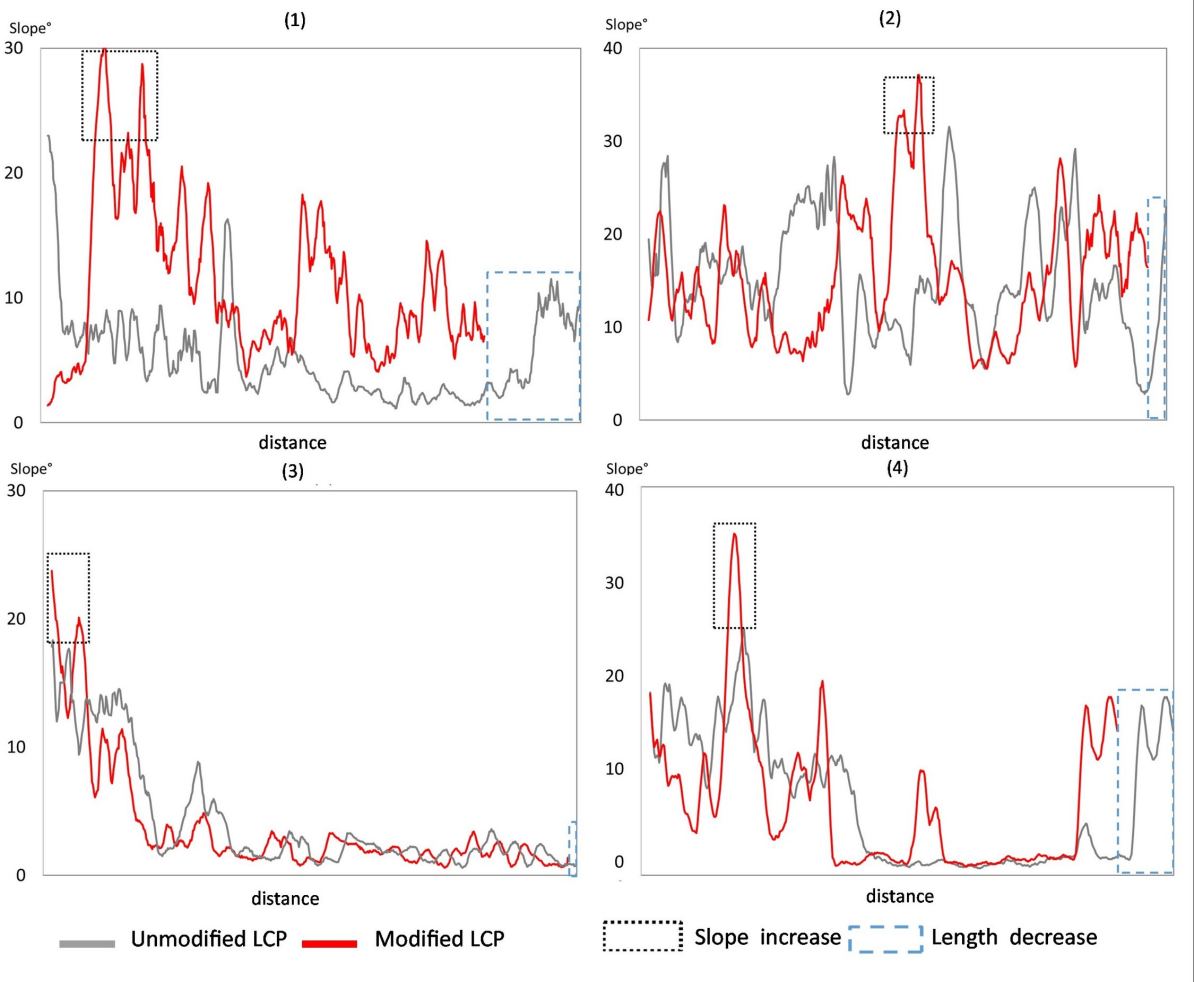
**Comparison of slope values along Least Cost Paths (LCPs) based on the unmodified and modified DEM.** Unmodified (original) LCPs appear as grey lines while modified LCPs appear as red lines. Selected for display were only the LCPs with the largest observed length reduction. Notice the black fine-dashed rectangles around the higher slope angles values of the LCP with gullies as barriers, as well as the dashed blue rectangles around the longer segments on the path-distance axis in the same modified LCP.

**Fig 8:**
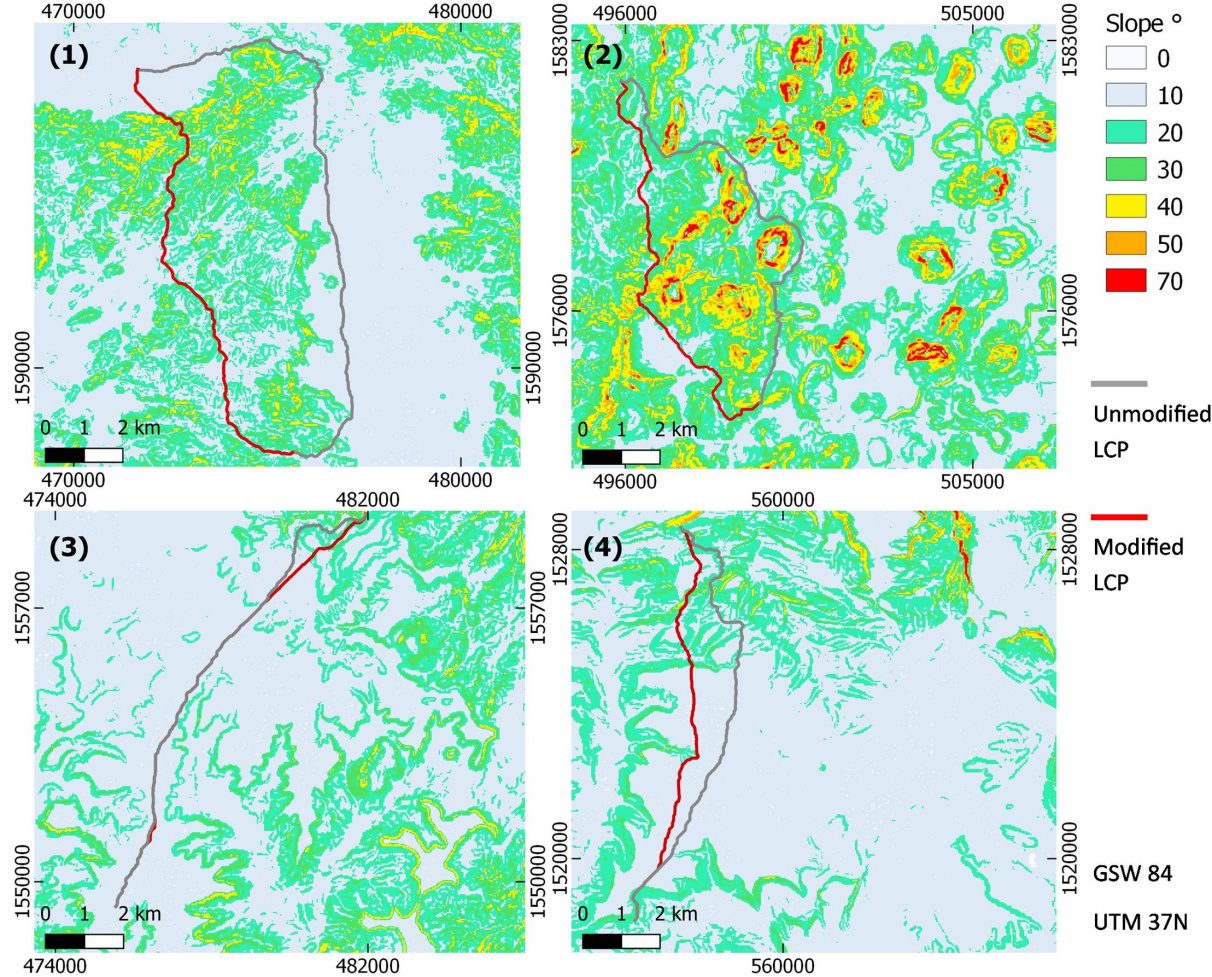
Spatial distribution of the Least Cost Paths (LCPs) with the largest observed length reduction presented in Fig 7. LCPs presented from north to south points. Unmodified LCPs appear as grey lines while modified LCPs appear as red lines. Numbers correspond to sample units. Notice areas with relatively higher slope angles. Slope maps based on 30 m SRTM DEM provided by USGS earth explorer [81–83].

In order to understand the possible mechanism responsible for LCPs length shortening, LCPs of the extreme cases on the south to north LCPs axis, for each sample unit, both applied on the unmodified and the modified DEM, are presented along the slopes’ gradients these LCPs cross. In all four sample units, the shorter S-N LCPs after gully blocking, also lead along steeper slopes than the unmodified LCP in at least one section of the pathway (Figs 7 and 8).

## Discussion

### The effect of pathways and roads on gully erosion

The process of gully erosion and the corresponding distribution of gullies is controlled by an intricate set of environmental and human induced factors [28, 38]. As interpretation of the exact values of FR is limited, addressing basic variables referred to in the literature was therefore the supporting basis to evaluate the relative possible influence of pathways and roads on the formation of gullies. Based on FR values of these variables, various authors have successfully generated susceptibility maps for gullies, flooded areas, and landslides with high prediction performances (76–91.7%) following data validation [102–106]. For the four sample units analysed, it is evident that lithology and Topographic Wetness Index (TWI) are the most influential variables for the occurrence of gullies (Fig 5). Lithology incorporates all rock characteristics determined mainly by minerals and their interconnections. At the surface, these result in bedrock density, pore volume or typical weathering products which influence erosional processes [114]. The other dominant variable, TWI, reflects a pixel’s control on hydrologic processes as it includes the parameters slope and the upslope drainage tributary to it [115]. Consequently, high influence of TWI on gully development results from its high explanatory power on the pixel’s relative moisture status [116]. In order to evaluate the effect of pathways on the location of gullies, random pixels, equal to the number of pixels generated by gully mapping for each sample unit, were tested for their Frequency Ratio (FR) within the classes of the ‘distance from pathways’ variable. Results for actual gully pixels within the ‘distance from pathways’ variable, goes well beyond these random pixels (Fig 5), proving that the location of gullies is related to the location of pathways. Results also show that the general effect of pathways and roads on the location of gullies is not as dominant as the effects of other environmental factors such as lithology, slope gradient and elevation which are traditionally considered [28, 38].

For the relationship between the occurrence of gullies and pathways, based both on modern and the CORONA (1960s) satellite images, the Daragà sample unit (3) shows FR values mostly less than 1 (Fig 5). However, it should be stressed that lower and higher than the expected random distribution (1) FR values, are not symmetric, i.e., negative correlation (lower than 1 values) within a variable’s class can go only as far as zero, while positive correlation (above 1) is infinite. Therefore, the exact extent to which gullies are less likely to occur (i.e., FR values 0–1) cannot be determined exclusively based on FR values. However, negative correlation based on FR values have been used to qualitatively evaluate the relative impact of different classes within a variable on the occurrences of gullies [38, 102–106]. Following these interpretations, results suggest gullies are less likely to occur next to pathways in the Daragà sample unit (3). However, for the pathways mapped on the basis of the USSR topographic maps, scale 1:200.000 published in the 1970s, a weak but positive relationship to the occurrence of today’s gullies can be observed (Fig 5). Hence, for the Daragà sample unit (3), more of the current gullies occur next to pathways known in the early 1970s than they do next to modern pathways and roads. It should be considered that due to the coarse scale of the USSR topographic maps only a part of the existing pathways and roads might have been recorded, causing a bias in the statistics based on these data. One reason for the negative relation between the occurrence of gullies and pathways in the Daragà sample unit (3), and gullies and roads in the Wuqro sample unit (4) (Fig 5), might be due to the implementation of Soil and Water Conservation (SWC) measures in Tigray during the past decades [22]. The SWC treatments in the study areas may have been more frequently implemented close to pathways and roads due to accessibility, producing a bias resulting in a negative relationship between pathways and gullies. Another reason for the negative relation between pathways and gullies in the Daragà sample unit (3) may be due to the occurrences of holloways. Holloways were considered neither in FR nor in LCP based on a demarcation problem: (1) Holloways are themselves pathways and hence could not be isolated from the movement variables, and (2) holloways in the sample units are usually only a few decimetres deep and do not exceed a depth of 0.5 m and, therefore, do not form steep obstacles for movement necessary to be considered in the LCP analysis. Pathways used for the FR calculation were mapped regardless of their possible holloway character. In the Daragà sample unit (3), the highest absolute number of holloways of all sample units was observed (Table 2). Consequently, it has to be considered that in the landscape of the Daragà sample unit (3), holloways—which serve both as pathways and shallow channels—are causing an increased “channel” density. By doing so, holloways affect the thresholds of concentrated runoff, which must be exceeded in order to start gully erosion. This would result in a relatively low density of gullies around pathways turned holloways, compared to the other areas within the sample unit.

The FR results for roads’ importance in determining the locations of gullies are in agreement with Nyssen et al. (2002) who observed for the Ethiopian Highlands that the location of roads on a slope affects runoff generation and as a result the formation of gullies further downslope [30]. Furthermore, results of the present work may suggest that the drainage of pathways and holloways could be considered as an additional criterion to consider while performing soil conservation measures or water harvesting in Ethiopia and elsewhere [62].

### The effect of gully erosion on human movement

The underlying assumption behind the calculation of movement cost is that gullies form barriers to movement. Field observations made clear that many gullies encountered were at least 2–3 m wide and 2–3 m deep, while only few gullies were smaller in width and depth and, thus, crossable by humans (Fig 3). Notwithstanding these properties, the geometry of gullies is highly variable along their course, perhaps to the extent that some are crossable at certain points. While some of the available satellite images have a horizontal resolution of 1 m x 1 m, reliable mapping of gullies on this basis was difficult. This was due to the poor ratio of the geometry of the forms to be mapped and the given image resolution. Additionally, gullies were impeded by low colour contrast of the images, i.e., gullies’ surfaces and their surroundings had similar colours. As a result, gullies which were neither deeper nor wider than 2–3 m, could not be distinguished from other linear forms, resulting from ploughing or vegetation changes along irrigation lines. Therefore, reliable detection of gullies from satellite images was possible mainly when the gullies were deep enough and wider than 2–3 m, thus generating visible shade. This observation is confirmed by comparison of satellite images with field data. Field validation clearly showed that shallow gullies that are less than 3 m in width were undetectable in the available satellite images. This partial bias resulted in the fact that smaller, crossable gullies were unrecognizable while mapping from satellite images. To further assure that mapped gullies for Least Cost Path (LCP) analysis were in fact at least 2 m wide, regular measurements of the width of the gullies were conducted using QGIS measure line while mapping the gullies using satellite images. These frequent width validations showed additionally that most measured gullies are wider than 5 m. Therefore, it is safe to assume that gullies mapped pose a barrier for movement during dry periods and even more so during heavy rains as it was observed that many gullies carry water during these events. Evaluation of the change of “Cost” in this study is presented as path length, rather than calories or time, to avoid assumptions regarding the type of mobility, which has changed over the millennia, whether it be for migration, trade or daily movement across Tigray or the Ethiopian Highlands [16–18].

Results of LCP analysis point out the effect of the gullies on the LCPs modelled for crossing the respective sample units following a south to north as well as an east to west axis. The extent of the elongations of LCPs is dependent upon the number of gullies they have to cross and local topography, concentrating the LCPs in one ‘bottle neck’ with gullies or forcing them to take deviations. Density and amounts of gullies are higher in plains as occurring in the Rama and Daragà sample units, than in well-drained areas dominated by steeper slopes or with perennial rivers such as in Yeha and Wuqro sample units (Table 2, Fig 2). TWI is a variable related directly to drainage and is proven to be important for controlling the location of gullies for these areas (Fig 5). Therefore, the differences in gully density between the sample units can be explained by difference in flow dynamics. Slope and high drainage density in sample units (2) and (4) generates a decrease of peak flow in the respective concentrated surface runoff, causing the threshold of surface runoff required for gully erosion not to be crossed [26, 29]. In all sample units, some Least Cost Paths lengthen by at least 17%, reaching an elongation of even 55% in the Daragà sample unit (3), while median changes of LCPs lengths range between 0.9%-5.4% (Fig 6, S2 Table). These elongations of pathways are in general due to detours taken to avoid the barrier. An unexpected result is the observation that some of the modelled LCPs get curtailed due to the development of gullies. The shortening of pathways following implementation of gullies as barriers is most dominant in the Yeha (2) and Rama (1) sample units with 55% and 38% of LCPs becoming shorter accordingly. This phenomenon is explained by the occurrence of other topographic barriers that are avoided in the absence of gullies, yet once the gullies appeared it became costlier to cross the gully than the other topographic barrier (Figs 6 and 7). This also implies that although shorter, these changed pathways are usually more difficult due to lower topographic conductivity. This may be attributed to least-cost algorithm whose local 8x8 neighbourhood of pixels determines available paths and might cause the paths to remain in less conductive areas that are eventually shorter in terms of distance [16–18]. However, it should be considered that after gully development has started, humans crossing the Ethiopian Highlands may have decided to take a steeper slope but shorter way due to gullies denying them crossing. Hence, this unexpected anomaly could be interpreted as another form of real-life path planning that considers historical contingency. In any event, further research has to elaborate on this.

While examining the differences between the lower medians and higher extreme values it should be considered that in the FR analysis it has been established that gullies occur often in proximity to pathways (Fig 5). Gullies, in this case obstacles for LCPs, are more likely to be around pathways and therefore paths people use in reality. For the random LCP analysis, the more gullies a LCP encounters after gullies were introduced, the longer it became. This implies that LCPs, which extended due to gullies crossing their original route, may better reflect movement related realities than that of a median average value for all randomly generated LCPs. In consequence, we assume that in the Ethiopian Highlands a lengthening of LCPs due to the development of barriers resulting from gully erosion reliably amounts up to 17–55%. Therefore, strictly using the median average results may downplay our understanding of the change in LCPs.

### Gully erosion in the Ethiopian Highlands

The northern Ethiopian Highlands are part of the main African divide with the Tekeze River draining into Nile River (Fig 1). The formation of the Ethiopian Highlands is part of the Tertiary formation of the Ethiopian–Yemen Plateau as well as the Rift formation [117]. Due to the strong relief, the drainage network is deeply incised into the Ethiopian Highlands, forming the base level for erosion. Today, gullies are a permanent feature in the Ethiopian Highlands [22, 27]. In order to understand historically how long gullies have formed in the Ethiopian Highlands, it can be assumed that the geological conditions were largely stable during settlement history. Paleo-environmental studies show that during much of the Holocene and in particular since mid-Holocene (ca. 5.6 cal kyr BP), climate in the Ethiopian Highlands was comparable to the current climate. Predominating climate since mid-Holocene had several semi-arid to dry-sub-humid phases with disperse vegetation cover under which gully erosion is more likely to take place [56, 57, 118, 119].

Gullies can form under natural conditions especially considering disperse vegetation, slope gradient, and parent material [e.g. 120]. However, their formation is accelerated under human impact on landscapes [28]. Gully erosion is initiated during heavy rainfall events when the shear stress on the parent material of a gully head is smaller than the shear stress caused by the running water [28]. In contrast, during moderate rainfall events, runoff is not sufficient to transport the sediments to the outlet; consequently, channels of gullies are characterized by alternating sections with strong channel bed accumulation and channel bed erosion. Resulting channel length profiles show typical riffle-pool sequences with decreasing frequency during channel development and concurrent formation of a concavity. Over time, these processes result in a levelling of the gully topography with the shape of older gullies characterized by a flattened gully head and banks and lower riffle-pool frequency compared to a new gully [121, 122]. In the Ethiopian Highlands, various generations of gullies appear in parallel [22, 27, 119].

Based on the past climatic and environmental reconstructions and the tendency of gullies to develop under semi-arid to dry-sub humid climate, both older and more recent types of gullies in the Ethiopian Highlands may have been affected by pathways and in turn formed obstacles for human movement (Figs 5 and 6). It is therefore highly probable that the proposed gullying-pathway mechanism occurred throughout much of human settlement history in the Ethiopian Highlands. Therefore, implications of the gully-pathway mechanism should be considered in archaeological and historical contexts as well [11, 119, 123].

### Historical movement cost implications of gully erosion

Material culture shows that long distance exchanges are dated well into the 1^st^ Millennium BC with the presence of Arabian architects, stonemasons, and merchants in Tigray [11, 44, 69]. For the later Aksumite period (2^nd^ century BCE-9^th^ century CE), most recent investigations on salt trade between the Ethiopian Highlands and locations across the Horn of Africa, emphasise the importance of inter-regional movement due to trade during the Aksumite period [124]. Additionally, Harrower and D’Andrea (2014) investigated possible routes of exchanges and travel between Aksum and the port city of Adulis using LCP analysis. Results of the latter work generally support a 1^st^ century CE documented account, the *Periplus Maris Erythraei*, suggesting an approximately eight-day travel time from Adulis to Aksum [11]. By considering a 15% change of path length due to the development of gullies (Fig 6) and converting this into time units, we may infer that there was an average of 1.2 days of additional travel time during the 1^st^ century CE compared to today. Weighing the effects of gully formation on the transport of goods depends on the way in which transport in the Ethiopian Highlands took place. Using pack animals such as donkeys has been suggested to take place in the Horn of Africa for the past 6000 years [125]. While wheeled wagons were in partial use for 4000 years in Egypt and Mesopotamia and later vastly in ancient Rome [126], whether due to lack of use or preservation bias, there is currently no archaeological evidence for wagon-based transport of goods in Tigray during pre-Aksumite and Aksumite times [64, 68, 127]. Travelers through the Ethiopian Highlands most likely preferring pack animals for transportation because, at least before the invention of sealed roads at the beginning of the 21^st^ century, resistance for the use of simple wagons on the inclined and most likely badly maintained gravel roads was too high. This is even more so during rainy seasons when some depressions and valleys become flooded [11, 12]. In recent decades, the use of animal-drawn carts became more common in some parts of the Ethiopian Highlands mainly due to road modernization providing easier access to different areas. From the aspect of carrying capacity, it has been suggested that in Ethiopia today, carts are able to carry up to ten times the load of pack animals [128]. The effect of gullies on transportation would slow both wheeled and animal-packed goods transport but would also highly vary between these two transport types. Moreover, as dominant features along and cutting through pathways, gullies may have affected decisions regarding which transportation to use in the dissected Ethiopian Highlands landscape. Since the Aksumite period until recently, ancient caravan activities using donkeys and camels as pack animals illustrate that crossing the Ethiopian Highlands is something large groups of humans did on a regular basis for millennia [64, 124]. Based on archaeological evidence, the extent of international trade indicates commerce relations with Egypt and other Arab regions occurred at the end of the Aksumite period [129]. During the post Aksumite period (ca. 10th-18th century CE), European travellers’ descriptions and maps provide input on interregional traveling and Ethiopian urban centres. Some of these medieval travellers describe commuting by foot, donkey or mule between various locations in the North Ethiopian Highlands extending to Eritrea, with Aksum being a dominant locality [129, 130]. A recent LCP analysis by Nyssen et al. (2020) investigated the role both topography and some post-Aksumite centres possibly played on pathway selection. These trajectories indicate that several LCPs have repeated in the same areas, making them ‘highways’ for travellers as suggested by the authors [12]. Our results suggest such intensive pathway networks would promote gully erosion in the vicinity of these pathways that may form barriers and raise the cost of movement during the post Aksumite period.

### Additional social implications of gully erosion–An outlook

The dissection of the Ethiopian Highlands by gullies may have had an additional long-term effect on societies occupying the region. Based on archaeological evidence, it is suggested that during the middle to late Holocene forager groups co-existed in the Ethiopian Highlands alongside pastoral farmers [44, 64, 68]. Transition to complex societies appeared in the Ethiopian Highlands far later than in neighbouring present-day Egyptian and Sudanese territories, mostly along the Nile valley [11]. When complex social structures are evident, from the pre-Aksumite period onwards, the persistence of local traditions over external and multi-regional influences, at least in Tigray, lays evidence of a strong local culture [64, 67, 68]. From a socio-ecological perspective it has been argued that topography influences social structures by defining cultural boundaries through physical obstacles [131]. It is also suggested that streams should be interpreted as natural properties as well as socio-cultural dimensions [132]. Following these concepts, it is worth noting that both naturally occurring and human accelerated gullies may have influenced the landscape on a socio-evolutionary scale, contributing to more dissected Ethiopian Highlands with topographically-bounded micro-environments. It is suggested that gullies of different ages, together with other topographic features, influenced the perception of territoriality. Such a dissected environment probably would impede the rooting of external or multi-regional cultural trends and innovations [e.g. 133, 134]. This in turn would have encouraged more regional diversification, allowing different groups and practices to co-exist during the Holocene, as well as strengthening the local culture over the regional one, as both aspects are portrayed in the archaeological record.

Moreover, since historical times, wars have been deeply affected by the ability to quickly cross large areas with full-sized armies and in most cases with wheeled vehicles [126]. The millennium-long Aksumite independence (1^st^ millennium CE), despite more dominant rival neighbouring kingdoms, is uncommon in East African history [44, 68]. However, the Ethiopian Highlands produced a further example of unique resistance when, in 1896, the Italian army suffered a defeat in the battle of Adwa, a city located in Tigray (between the Yeha (2) and Daragà (3) sample units). Following a short, unstable occupation (1936–1941), an important and relevant feature left from the Italian rule are a network of roads across the Ethiopian Highlands which were constructed mainly to assist in the occupation of Ethiopia [135, 136]. The long Aksumite independence and the failure of the Italian army to occupy the highlands in the late 19^th^ century each have a complex set of causes [44, 68, 137]. That being stated, gullies, deterring large scale movement across the Ethiopian Highlands, should certainly not be disregarded as a significant physical factor affecting these processes and events.

## Conclusions

Human movement and gully erosion are related to each other. Results of this study suggest that, for the Ethiopian Highlands, the more pathways develop, the more likely gullies are to occur in an area. If the cross profile of these gullies reaches dimensions of minimum 2 m depth and 2–3 m width, they obtain the characteristics of obstacles for movement of people. Consequently, humans will search for another pathway to continue moving and reach their destination while keeping the costs of movement as low as possible. This process will make their way longer when going around one or several topographical obstacles. When gullies result in deflection of pathways, this may lead to the creation of new gullies, subsequently forming a feedback mechanism between the two. Moreover, former decisions for least cost pathways on the basis of given topography might be revised when new topographical obstacles develop, potentially resulting in the decision to include formerly avoided topographical barriers into the new pathway. This may possibly result in a shorter but more difficult pathway than the preceding one. In the Ethiopian Highlands, this relationship potentially impacted commerce and the exchange of goods, as well as large scale accessibility for military and other purposes. From a socio-cultural perspective, gullies may have contributed to a more diversified and sub-divided population in the Ethiopian Highlands, one that is less susceptible to external influences and trends as compared with nearby areas. As concepts of physical movement changed dramatically in recent decades, the gully-movement feedback mechanism in Tigray serves as an early example of the effect humans may have had on their natural environment.

## Supporting information

S1 File (DOCX)

S2 File (DOCX)

S3 File (PDF)

S1 Table Full results of Frequency Ratio calculations for all sample units. (DOCX)

S2 Table Least Cost Paths (LCPs) for all sample units 1–4. (DOCX)
