# Supplementary material for: Human movement and gully erosion: Investigating feedback mechanisms using Frequency Ratio and Least Cost Path analysis in Tigray, Ethiopia
Source: PLoS One. 2021 Feb 5;16(2):e0245248. doi: 10.1371/journal.pone.0245248 (PMC7864406; doi:10.1371/journal.pone.0245248)
Supplement: S1 File — (DOCX) [file pone.0245248.s001.docx]

R script 1.

**## Frequency Ratio (FR) calculation**

## R script for all reclassified variables (elevation, slope aspect, slope angle, TWI, P.curvature, NDVI, DFS, DFR,DRP ,DFPSM , SFPCI) and non-reclassified (LULC, lithology, soil).

 ## For LULC the natural break alone was used while for lithology and soil types, classifications were based on the spatial units in the according maps

## The script uses elevation as an example. Based on data in table 1, this could be applied to all other variables

library(raster)

## Loading the 5 m x 5 m DEMs

Elevation_ sample_unit_1<-raster("elevation_ sample_unit_1.tif")

Elevation_ sample_unit_2<-raster("elevation_ sample_unit_2.tif")

Elevation_ sample_unit_3<-raster("elevation_ sample_unit_3.tif")

Elevation_ sample_unit_4<-raster("elevation_ sample_unit_4.tif")

## Loading the gullies and matching them with the DEMs’ rows and columns for later calculations

Gullies_ sample_unit_1<-resample(raster("gullies_ sample_unit_1.tif"),raster("elevation_ sample_unit_1.tif"),method="ngb")

Gullies_ sample_unit_2<-resample(raster("gullies_ sample_unit_2.tif"),raster("elevation_ sample_unit_2.tif"),method="ngb")

Gullies_ sample_unit_3<-resample(raster("gullies_ sample_unit_3.tif"),raster("elevation_ sample_unit_2.tif"),method="ngb")

Gullies_ sample_unit_4<-resample(raster("gullies_ sample_unit_4.tif"),raster("elevation_ sample_unit_2.tif"),method="ngb")

## Evaluating the natural distribution of the elevation in the sample units

hist(Elevation_ sample_unit_1)

hist(Elevation_ sample_unit_2)

hist(Elevation_ sample_unit_3)

hist(Elevation_ sample_unit_4)

## Reclassify in same size categories according to the common natural distribution of the sample units for generating similar data sets (elevation, slope aspect, slope angle, TWI, P.curvature, NDVI).

class <- c(1800, 1900, 1,

           1900, 2000, 2,

           2000, 2100, 3,

           2100, 2200, 4,

           2200, 2300, 5,

           2300, 2900, 6)

 ## For variable elevation only: Sample unit 1 which is different in elevation has a different classification. All other reclassified variables in all sample units were sub-categorized together.

class <- c(1100, 1200, 1,

           1200, 1300, 2,

           1300, 1400, 3,

           1400, 1500, 4,

           1500, 1600, 5,

           1600, 2300, 6)

## Reclassification for all distance-based variables (rivers, roads, pathways, soviet mapped pathways, CORONA image based mapped pathways). The following example is in the case of streams and roads (distance in meters)

class <- c( 0, 50, 1,

50, 100, 2,

100, 150, 3,

150, 200, 4,

200, 250, 5,

250, 50000, 6)

 ## For all reclassified variables: Reshape the object into a matrix with columns and rows

[rcl.sl](http://rcl.sl/) <- matrix(class,

                 ncol=3,

                 byrow=TRUE)

## For all reclassified variables: Reclassify the variable using the reclass object - rcl.m

Elevation_reclas_sample_unit_1 <- reclassify(Elevation_ sample_unit_1,  [rcl.sl](http://rcl.sl/))

Elevation_reclas_sample_unit_1[Elevation_reclas_sample_unit_1[]==-1] <- NA

Elevation_reclas_sample_unit_2 <- reclassify(Elevation_ sample_unit_2,  [rcl.sl](http://rcl.sl/))

Elevation_reclas_sample_unit_2[Elevation_reclas_sample_unit_2[]==-1] <- NA

Elevation_reclas_sample_unit_3 <- reclassify(Elevation_ sample_unit_3,  [rcl.sl](http://rcl.sl/))

Elevation_reclas_sample_unit_3[Elevation_reclas_sample_unit_3[]==-1] <- NA

Elevation_reclas_sample_unit_4 <- reclassify(Elevation_ sample_unit_4,  [rcl.sl](http://rcl.sl/))

Elevation_reclas_sample_unit_4[Elevation_reclas_sample_unit_4[]==-1] <- NA

**## For all variables: Calculation of overlap - gullies pixels for each variable**

Elevation_Gullies_sample_unit_<-Elevation_reclas_sample_unit_1* Gullies_ sample_unit_1

Elevation_Gullies_sample_unit_2<-Elevation_reclas_sample_unit_2* Gullies_ sample_unit_2

Elevation_Gullies_sample_unit_3<-Elevation_reclas_sample_unit_3* Gullies_ sample_unit_2

Elevation_Gullies_sample_unit_4<-Elevation_reclas_sample_unit_4* Gullies_ sample_unit_2

### Exporting spatial data for documentation

writeRaster(Elevation_Gullies_ sample_unit,"Elevation_Gullies_ sample_unit.tif",overwrite=TRUE)

## FR calculation

## Gully and variable pixel overlap = **g**

tmp1 <- hist(Elevation_Gullies_sample_unit, density = NULL, axes = TRUE, plot = TRUE)

## variable pixel = **C**

tmp2 <- hist(Elevation_reclas_sample_unit_2,

density = NULL, col = "red",

main = paste(" elevation in the sample_unit_2"),

xlab = "Elevations", ylab="Gullies (in Pixel frequency)",

axes = TRUE, plot = TRUE)

## Output total gully pixels = **G**

tmp3 <- hist(Gullies_sample_unit_2,

density = NULL, col = "red",

main = paste("Gullies by elevation in the sample_unit_2"), xlab = "Elevations", ylab="Gullies (in Pixel frequency)",

axes = TRUE, plot = TRUE)

## Output sample unit pixel = **S**

tmp4 <-hist(Elevation_sample_unit_2,plot=FALSE)

## Producing data frames

g.df<-data.frame(counts=tmp1$counts)
C.df<-data.frame(counts=tmp2$counts)
G.df<-data.frame(counts=tmp3$counts)
S.df<-data.frame(counts=tmp4$counts)

## Retrieving the relevant numbers from each data frame

names(g.df)[names(g.df) == "counts"] <- "gullies"
names(C.df)[names(C.df) == "counts"] <- "category"

G<-sum(G.df)
S<-sum(S.df)

g.df[g.df[]==0] <-NA
C.df[C.df[]==0] <-NA

g<-na.omit(g.df)
C<-na.omit(C.df)

## Binding the four elements into one object

FR_data<-cbind(g,C,G,S)

## **Calculating the Frequency Ratios of the classes for a given variable**
FR_data_Elevation_sample_unit<-(FR_data$gullies/FR_data$category)/(FR_data$G/FR_data$S)

## Export the data

write.csv(FR_data_Elevation_sample_unit," FR_data_Elevation_sample_unit.csv")
