# Supplementary material for: Human movement and gully erosion: Investigating feedback mechanisms using Frequency Ratio and Least Cost Path analysis in Tigray, Ethiopia
Source: PLoS One. 2021 Feb 5;16(2):e0245248. doi: 10.1371/journal.pone.0245248 (PMC7864406; doi:10.1371/journal.pone.0245248)
Supplement: S2 File — (DOCX) [file pone.0245248.s002.docx]

R script 2.

**## Least Cost Path Analysis**

library(raster)

library(gdistance)

library(rgdal)

## general

altDiff <- function(x){x[2] - x[1]}

## wheel critical slope cost function; the slope is requested as rise/run by the cost function;

## sl.crit (=critical slope, in percent) is "the transition where switchbacks become more effective than direct uphill or downhill paths" and typically is in the range 8-16

sl.crit <- 12

## DEM

dem <- raster("./sample_unit_DEM_res.tif")

hd <- transition(dem, altDiff, 8, symm=FALSE)

slope <- geoCorrection(hd)

adj <- adjacent(dem, cells=1:ncell(dem), pairs=TRUE, directions = 8)

speed <- slope

## wheel critical slope

speed[adj] <- 1 / (1 + ((abs(slope[adj])*100) / sl.crit)^2)

Conductance <- geoCorrection(speed)

## Gullies as barriers

gullies_raw <- raster("./sample_unit_gullies_res.tif")

dem_gullies <- dem

dem_gullies[gullies_raw] <- 99999

hd2 <- transition(dem_gullies, altDiff, 8, symm=FALSE)

slope2 <- geoCorrection(hd2)

adj2 <- adjacent(dem_gullies, cells=1:ncell(dem_gullies), pairs=TRUE, directions = 8)

speed2 <- slope2

## wheel critical slope

speed2[adj2] <- 1 / (1 + ((abs(slope2[adj2])*100) / sl.crit)^2)

Conductance_gullies <- geoCorrection(speed2)

## Areas for random points

N_R<- extent(dem)

N_R[3] <- N_R[4] - 1000

S_R<- extent(dem)

S_R[4] <- S_R[3] + 1000

poly_sample_unit_N<-as(N_R, "SpatialPolygons")

poly_sample_unit_S<-as(S_R, "SpatialPolygons")

##Raster Values

sample_unit_ng_con<-raster(Conductance)

sample_unit_g_con<-raster(Conductance_gullies)

## results files

my_results <- c()

my_length_ng <- c()

my_length_g <- c()

my_conduc_results_mean_ng <- c()

my_conduc_results_mean_g <- c()

my_conduc_results_median_ng <- c()

my_conduc_results_median_g <- c()

## the loop:

for (i in 1:1000) {

origin <- spsample(poly_sample_unit_N[1,], 1, type = 'random')

goal <- spsample(poly_sample_unit_S[1,], 1, type='random')

sample_unit_ng_lcp <- shortestPath(Conductance, origin, goal,

output="SpatialLines")

sample_unit_g_lcp <- shortestPath(Conductance_gullies, origin, goal,

output="SpatialLines")

## Extract the lines

sample_unit_ng <- SpatialLinesDataFrame(sample_unit_ng_lcp, data.frame(id=1:length(sample_unit_ng_lcp)))

sample_unit_g <- SpatialLinesDataFrame(sample_unit_g_lcp, data.frame(id=1:length(sample_unit_g_lcp)))

dir.create("ng")

dir.create("g")

writeOGR(sample_unit_ng, dsn=paste0("ng/","lcp_ng_",i,".gpkg"), layer = "new_ng",

driver = "GPKG")

writeOGR(sample_unit_g, dsn=paste0("g/","lcp_g_",i,".gpkg"), layer = "new_g",

driver = "GPKG")

ngl<-SpatialLinesLengths(sample_unit_ng)

gl<-SpatialLinesLengths(sample_unit_g)

my_length_ng [i] <-ngl

my_length_g [i] <-gl

a<-((1-(ngl[1]/gl[1]))*100)

my_results [i] <-a

## Extracting conductivity data

sample_unit_extract_ng<-extract(sample_unit_ng_con,sample_unit_ng, along = TRUE,

cellnumbers = FALSE)

sample_unit_extract_g<-extract(sample_unit_g_con,sample_unit_g, along = TRUE,

cellnumbers = FALSE)

a1<-mean.default(unlist(sample_unit_extract_ng))

a2<-mean.default(unlist(sample_unit_extract_g))

my_conduc_results_mean_ng [i] <- a1

my_conduc_results_mean_g [i] <- a2

b1<-median(unlist(sample_unit_extract_ng))

b2<-median(unlist(sample_unit_extract_g))

my_conduc_results_median_ng [i] <- b1

my_conduc_results_median_g [i] <- b2}

## Writing the output file

total<-data.frame(calculation=my_results, length_ng=my_length_ng, length_g=my_length_g, cond_mean_ng=my_conduc_results_mean_ng,

cond_mean_g=my_conduc_results_mean_g, cond_med_ng=my_conduc_results_median_ng, cond_med_g=my_conduc_results_median_g)

write.csv(total,"sample_unit__LCP_total_data.csv")

##East to west LCPs calculations differs in:

## Areas for random points

E_R<- extent(dem)

E_R[1] <- E_R[2] - 1000

W_R<- extent(dem)

W_R[2] <- W_R[1] + 1000

poly_sample_unit_E<-as(E_R, "SpatialPolygons")

poly_sample_unit_W<-as(W_R, "SpatialPolygons")

## the loop:

for (i in 1:1000) {

origin <- spsample(poly_sample_unit_E[1,], 1, type = 'random')

goal <- spsample(poly_sample_unit_W[1,], 1, type='random')

sample_unit_ng_lcp <- shortestPath(Conductance, origin, goal,

output="SpatialLines")

sample_unit_g_lcp <- shortestPath(Conductance_gullies, origin, goal,

output="SpatialLines")
