# Supplementary material for: Human movement and gully erosion: Investigating feedback mechanisms using Frequency Ratio and Least Cost Path analysis in Tigray, Ethiopia
Source: PLoS One. 2021 Feb 5;16(2):e0245248. doi: 10.1371/journal.pone.0245248 (PMC7864406; doi:10.1371/journal.pone.0245248)

Mapped gullies Rama sample unit (1)

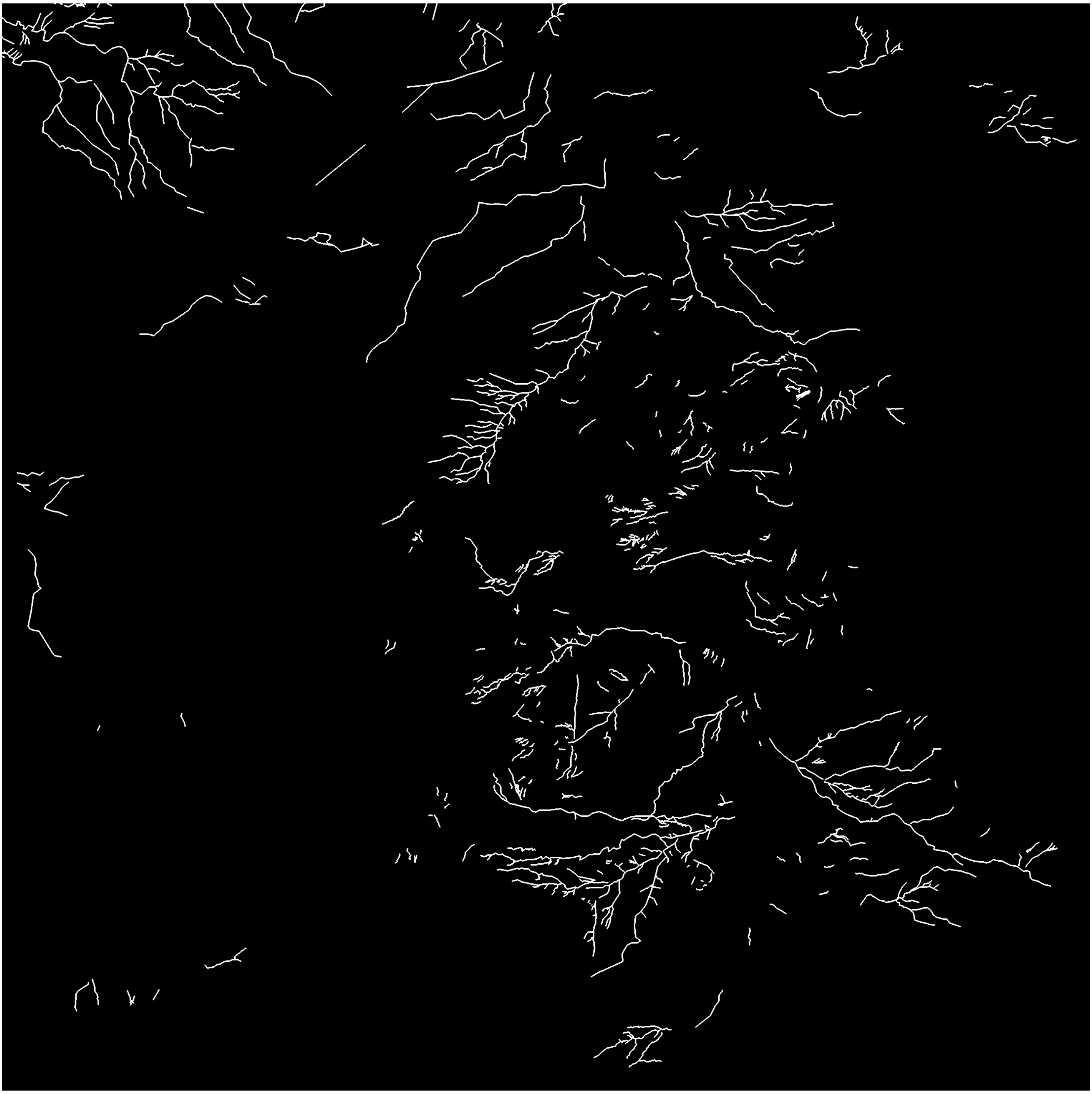

Mapped gullies Yeha sample unit (2)

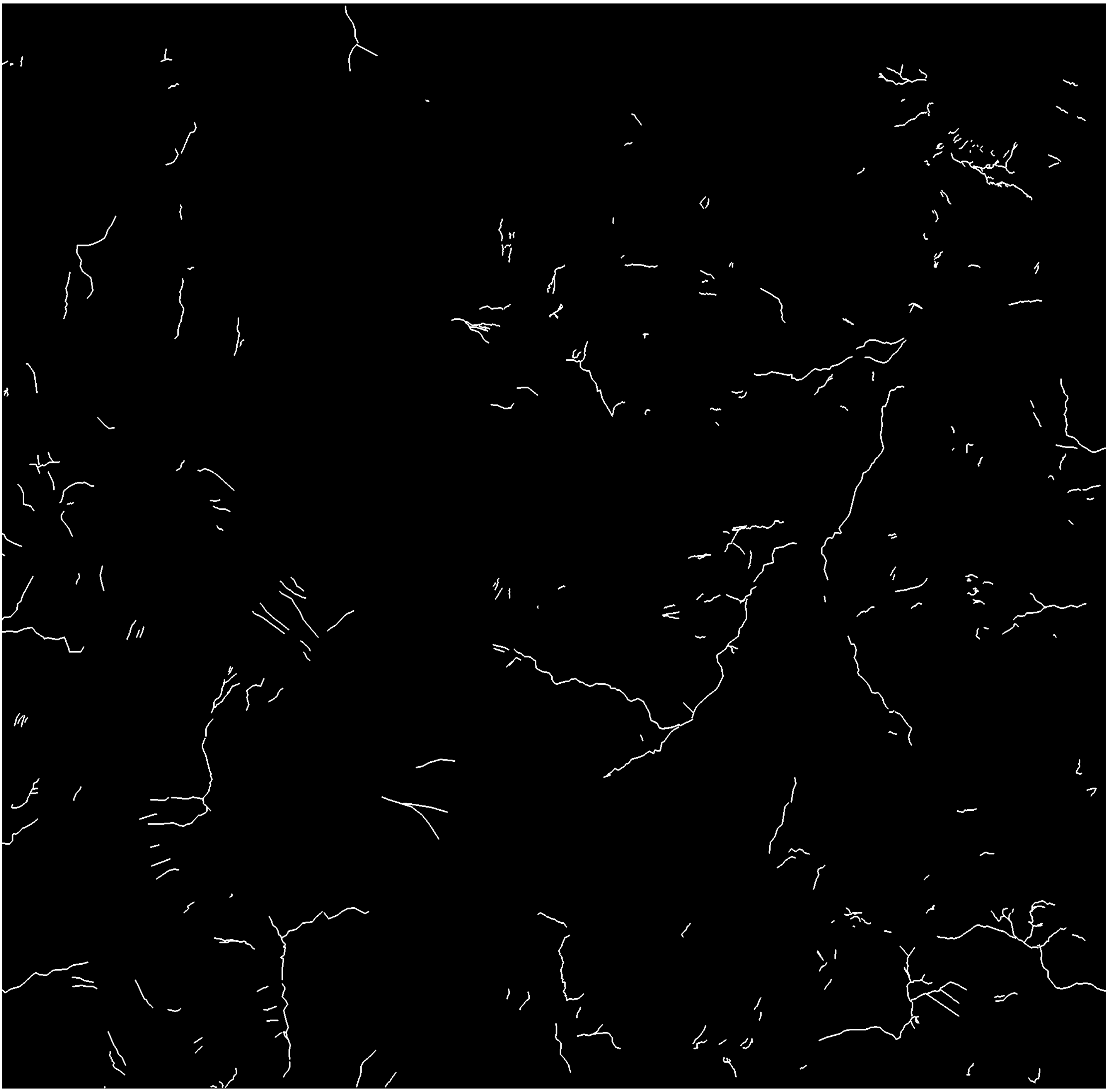

Mapped gullies Melazo sample unit (3)

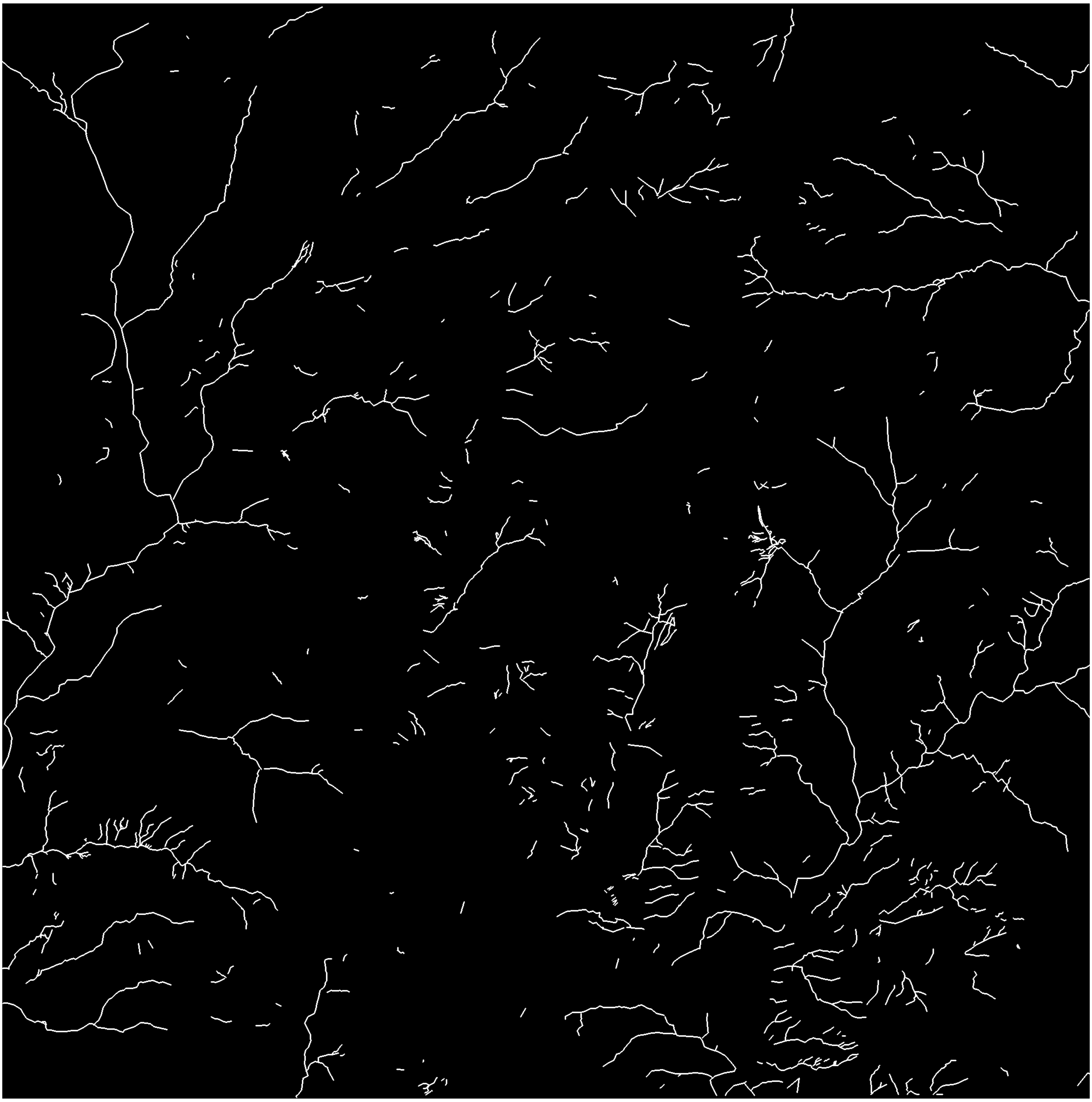

Mapped gullies Wuqro sample unit (4)

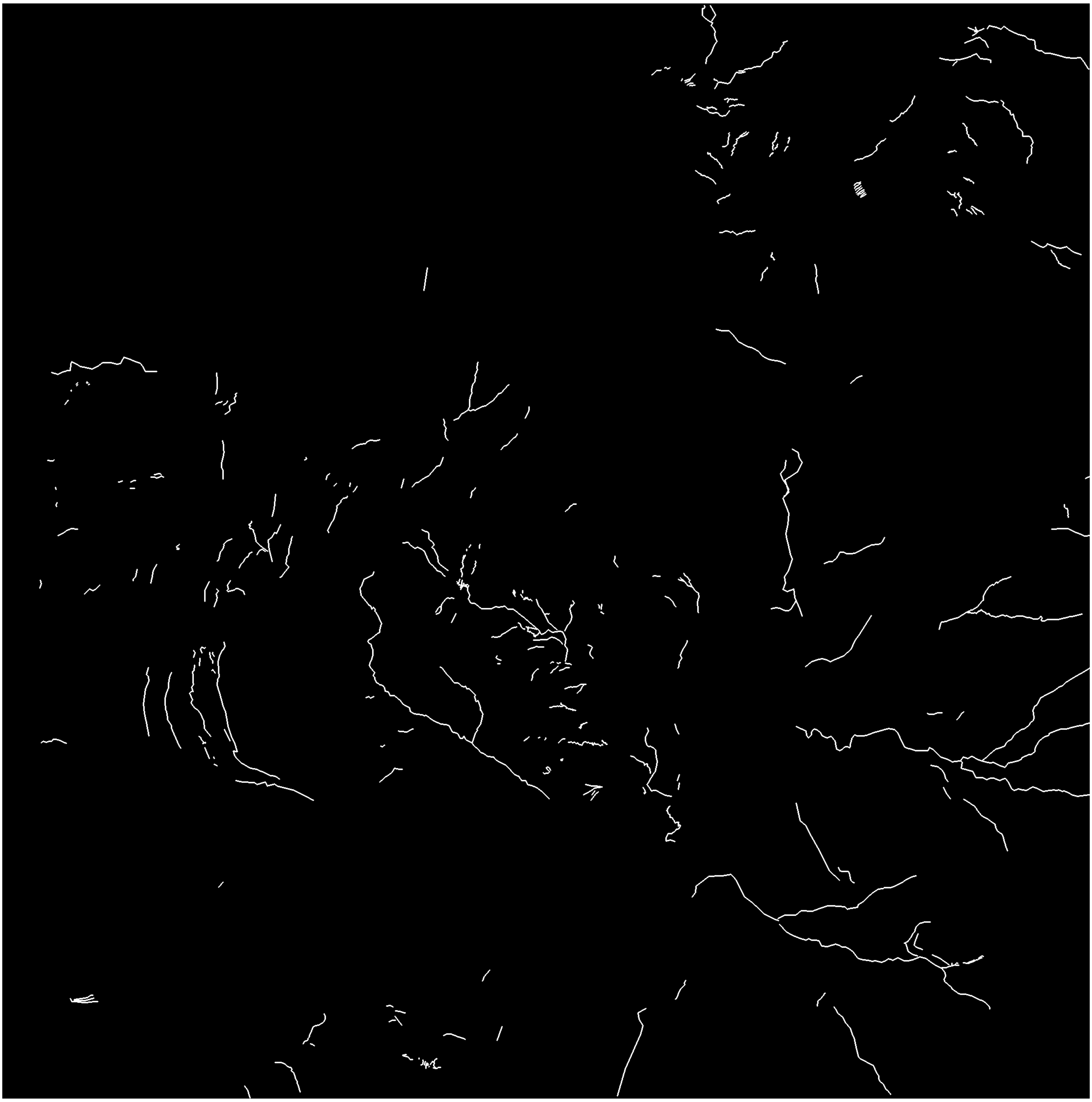

Supplement: S3 File — (PDF) [file pone.0245248.s003.pdf]
