# Supplementary material for: Human movement and gully erosion: Investigating feedback mechanisms using Frequency Ratio and Least Cost Path analysis in Tigray, Ethiopia
Source: PLoS One. 2021 Feb 5;16(2):e0245248. doi: 10.1371/journal.pone.0245248 (PMC7864406; doi:10.1371/journal.pone.0245248)
Supplement: S1 Table — (DOCX) [file pone.0245248.s004.docx]

S1 Table. Full results of Frequency Ratio calculations for all sample units. Notice the standard deviation (STDEV.P in Excel) for each variable represents its importance in each sample unit, with average for each variable at the third column

|  | Classes | Classes / STDEV Avrage | Rama Sample unit 1* | Yeha Sample unite 2 | Melazo Sample unite 3 | Wuqro Sample unit 4 |
| --- | --- | --- | --- | --- | --- | --- |
| Elevation | 1800-1900 | 1100-1200* | 0.327650991 | 1.178949358 | 3.559972565 | 0.347309561 |
|  | 1900-2000 | 1200-1300* | 1.204959512 | 1.107998572 | 2.311899375 | 1.317896606 |
|  | 2000-2100 | 1300-1400* | 1.53932905 | 1.365105937 | 0.99042216 | 0.963366382 |
|  | 2100-2200 | 1400-1500* | 2.529565265 | 1.294959134 | 0.40255142 | 0.199035724 |
|  | 2200-2300 | 1500-1600* | 0 | 0.697253642 | 0.03273538 | 0.186317532 |
|  | >2300 | >1600* |  | 0.620647026 |  | 0.131389596 |
|  | STDEV | 0.735639759 | 0.90023838 | 0.285148293 | 1.304714627 | 0.452457737 |
| Slope (deg) | 0-5 |  | 2.02425268 | 2.551061073 | 1.05265088 | 1.602580457 |
|  | 5-10 |  | 0.581143405 | 1.20901514 | 1.160332039 | 1.46819881 |
|  | 10-15 |  | 0.30024664 | 0.678946319 | 0.850946278 | 1.228521653 |
|  | 15-20 |  | 0.245873158 | 0.472068934 | 0.801094783 | 0.59823107 |
|  | 20-25 |  | 0.360923893 | 0.566205305 | 0.525373908 | 0.445414769 |
|  | >25 |  | 0.153098403 | 0.735633425 | 0.060894911 | 0.328282988 |
|  | STDEV | 0.558228793 | 0.645532301 | 0.716679314 | 0.364622652 | 0.506080905 |
| Aspect | -1- 0 | Flat | 2.100755374 | 1.45504519 | 0.940223692 | 2.117856091 |
|  | 0-22.5 / 337.5-360 | N | 0.911798544 | 0.678789024 | 0.722624497 | 1.168009509 |
|  | 22.5- 67.5 | NE | 1.203032565 | 0.787975915 | 0.800314518 | 1.427216139 |
|  | 67.5 - 112.5 | E | 1.34155916 | 1.129733935 | 0.896886157 | 1.287243121 |
|  | 112.5 - 157.5 | SE | 0.91747478 | 1.12978745 | 0.941374206 | 1.201284573 |
|  | 157.5 - 202.5 | S | 0.701034635 | 1.412237089 | 0.983307709 | 1.077845348 |
|  | 202.5 - 247.5 | SW | 0.822896676 | 1.326306897 | 1.334184359 | 1.439818162 |
|  | 247.5 - 292.5 | W | 0.998074886 | 0.935621019 | 1.359882454 | 1.336956568 |
|  | 292.5 - 337.5 | NW | 1.089327552 | 0.704567824 | 0.960546001 | 1.201972927 |
|  | STDEV | 0.292469776 | 0.391838011 | 0.283207911 | 0.204688146 | 0.290145038 |
| Lithology | Mapped spatial unit |  | 0.401447022 | 1.155675133 | 0.714960625 | 1.246303379 |
|  | Mapped spatial unit |  | 0.62692276 | 1.590054485 | 0.753703883 | 0.378540173 |
|  | Mapped spatial unit |  | 2.24597452 | 0.773104573 | 1.667711019 | 0.35754423 |
|  | Mapped spatial unit |  | 1.035057884 | 0.88801128 | 0.445138206 | 0.767007755 |
|  | Mapped spatial unit |  | 3.424365854 | 1.575630367 | 2.917824483 | 3.448299383 |
|  | Mapped spatial unit |  |  | 1.445597188 | 4.873058111 | 2.299674009 |
|  | Mapped spatial unit |  |  |  |  | 2.359362736 |
|  | STDEV | 1.029089589 | 1.134370848 | 0.323117745 | 1.568641043 | 1.090228719 |
| Soil | Mapped spatial unit |  | 0.476452357 | NA | 0.806781142 | 0.94822016 |
|  | Mapped spatial unit |  | 0.998879975 |  | 1.065870684 | 1.332941381 |
|  | Mapped spatial unit |  | 1.055162057 |  |  |  |
|  | STDEV | 0.194153676 | 0.260555647 |  | 0.129544771 | 0.192360611 |
| LULC | Tree cover |  | 0.672808682 | 0.88243035 | 0.726648246 | 1.386269248 |
|  | Shurbs |  | 0.903874044 | 0.648347272 | 1.50000418 | 0.331424924 |
|  | Fields |  | 1.100603351 | 0.785872866 | 1.793856551 | 0.458606881 |
|  | Cropland |  | 1.022886746 | 1.101088955 | 0.941235866 | 1.220937193 |
|  | Vegetation aquatic or regularly flooded | |  | 0 | 0.551522678 | 0 |
|  | Sparse Vegetation |  | 0 | 0 | 0 | 0 |
|  | Bare areas |  | 0.414071463 | 0 | 1.538585171 | 3.559977152 |
|  | Buit up |  | 0 | 0 | 0 | 0.02895131 |
|  | open water |  |  |  | 0 | 0.749468874 |
|  | STDEV | 0.652590238 | 0.42771758 | 0.442847702 | 0.668987224 | 1.070808448 |
| NDVI | -0.2- 0.075 | Water/Bare area | 0.744071808 | 1.414063667 | 0.262398053 | 0.578811678 |
|  | 0.075- 0.125 | Bare area | 0.945247508 | 1.310527462 | 0.536883885 | 0.999308449 |
|  | 0.125- 0.175 | Grassland | 1.103128261 | 0.909008927 | 0.902871347 | 1.16799128 |
|  | 0.175- 0.225 | Grassland | 1.577113465 | 0.93253075 | 1.926263718 | 0.919194155 |
|  | 0.225-0.275 | Fields/Shrubs | 1.291660563 | 0.946946808 | 2.395405872 | 0.743015953 |
|  | 0.275-0.325 | shurbs | 0.909504074 | 0.997801485 | 2.166363175 | 0.86450695 |
|  | 0.325-0.875 | Sparse vegetation | 0.253121784 | 1.933074084 | 2.606343151 | 0.875327509 |
|  | STDEV | 0.44849681 | 0.388925648 | 0.349754396 | 0.882750569 | 0.172556627 |
| TWI | 5--7 |  | 0.04022655 | 0.541473821 | 0.149369478 | 10.45885373 |
|  | 7--9 |  | 0.292474781 | 0.498200887 | 0.457349217 | 1.741049547 |
|  | 9--11 |  | 1.167445639 | 1.493148212 | 0.938867263 | 0.971344511 |
|  | 11--13 |  | 2.340531833 | 2.734626389 | 1.772121145 | 0.062879075 |
|  | 13--15 |  | 2.877878393 | 3.36358395 | 1.156991084 | 0.300637211 |
|  | 15--17.5 |  | 1.367974516 | 0.933334908 | 0.667494856 | 0 |
|  | STDEV | 1.587146824 | 1.015139623 | 1.094508443 | 0.521299259 | 3.717639973 |


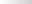


|  | Classes | Classes / STDEV Avrage | Rama Sample unit 1* | Yeha Sample unite 2 | Melazo Sample unite 3 | Wuqro Sample unit 4 |
| --- | --- | --- | --- | --- | --- | --- |
| P.Curvature | -100 - -10 |  | 0.510964513 | 0.766993535 | 0.386237487 | 1.279993091 |
|  | -10 - -3 |  | 0.454168273 | 0.970614876 | 0.9856321 | 0.535580461 |
|  | -3 - -2 |  | 0.91004939 | 1.515652193 | 1.675893756 | 0.731935006 |
|  | -2 - -1 |  | 1.468086821 | 1.697082943 | 1.332671722 | 1.203837747 |
|  | -1 - 0 |  | 1.810177905 | 1.71870393 | 1.010097185 | 1.404950615 |
|  | 0 - 1 |  | 1.480944799 | 1.503554833 | 0.899663514 | 1.238261299 |
|  | 1 - 2 |  | 0.71740497 | 0.873150833 | 0.872620936 | 0.915560379 |
|  | 2 - 9 |  | 0.271967112 | 0.411871324 | 0.617491976 | 0.246130231 |
|  | 9 - 100 |  | 0.157419542 | 0.528291522 | 0.07561412 | 0.0916171 |
|  | STDEV | 0.484243041 | 0.558445963 | 0.477676284 | 0.450453524 | 0.450396393 |
| Distance from river | 0-50m |  | 1.311062351 | NA | NA | 0.839615255 |
|  | 50- 100m |  | 2.642596751 |  |  | 0.756838393 |
|  | 100- 150m |  | 2.923811186 |  |  | 0.633825104 |
|  | 150- 200m |  | 3.142312108 |  |  | 0.855728038 |
|  | 200- 250m |  | 1.845715768 |  |  | 0.720074502 |
|  | 250< |  | 0.983536569 |  |  | 1.024164414 |
|  | STDEV | 0.468819602 | 0.81473185 |  |  | 0.122907353 |
| Distance from roads | 0-50m |  | 1.034916772 | 0.891108749 | 0.331171224 | 0.341668086 |
|  | 50-100m |  | 2.573178445 | 1.595693536 | 0.377646388 | 0.563884934 |
|  | 100-150m |  | 2.027249421 | 2.134849029 | 0.236909455 | 0.897929988 |
|  | 150-200m |  | 1.989403466 | 1.82428389 | 0.272239497 | 0.65948799 |
|  | 200-250m |  | 1.479000019 | 1.762229685 | 0.408994253 | 0.633513939 |
|  | >250m |  | 0.964391074 | 0.955750903 | 1.042986072 | 1.021496521 |
|  | STDEV | 0.381563292 | 0.574845842 | 0.45617843 | 0.273716739 | 0.221512155 |
| Distance from pathway | 0-25m |  | 1.835359483 | 1.280032366 | 0.556235109 | 1.69422746 |
|  | 25-50m |  | 1.496141592 | 1.347039168 | 0.772680714 | 1.246400077 |
|  | 50-75m |  | 1.483149279 | 1.451092666 | 0.726117536 | 1.180310637 |
|  | 75-100m |  | 1.597053613 | 1.531540924 | 0.827625101 | 1.128306319 |
|  | 100-125m |  | 1.624082034 | 1.274494244 | 0.772322802 | 1.267100221 |
|  | >125m |  | 0.895681993 | 0.915260495 | 1.059192348 | 0.929555102 |
|  | STDEV | 0.215944424 | 0.289251348 | 0.194818961 | 0.148945424 | 0.230761963 |
| Distance from pathways USS | 0-50m |  | 1.339100379 | 1.206409252 | 0.95616316 | 1.460575918 |
|  | 50-100m |  | 1.385025583 | 1.449226378 | 1.220485513 | 1.250770363 |
|  | 100-150m |  | 1.289131197 | 1.445435368 | 1.192950659 | 1.655425315 |
|  | 150-200m |  | 1.059107957 | 1.516525951 | 1.022360624 | 1.397146257 |
|  | 200-250m |  | 1.30584647 | 1.211801132 | 1.051309158 | 1.411386361 |
|  | >250m |  | 0.972429323 | 0.952343134 | 0.983973529 | 0.951802096 |
|  | STDEV | 0.166102807 | 0.153073018 | 0.194786826 | 0.100608357 | 0.215943027 |
| Distance from pathways CORON | 0-25m |  | 1.423346774 | 1.064229547 | 0.77306228 | 1.099178596 |
|  | 25-50m |  | 1.680213391 | 1.584671139 | 0.725498487 | 1.467211559 |
|  | 50-75m |  | 1.610995431 | 1.232318551 | 0.873334652 | 1.510309223 |
|  | 75-100m |  | 1.741378323 | 1.376936795 | 0.827256207 | 1.038958305 |
|  | 100-125m |  | 1.605688229 | 1.394658225 | 0.82649275 | 1.009086346 |
|  | >125m |  | 0.941104572 | 0.966489348 | 1.077209549 | 0.935781005 |
|  | STDEV | 0.203766804 | 0.26847375 | 0.208915087 | 0.111547627 | 0.226130753 |
| DFP Random pixels | 0-25m |  | 0.894423913 | 0.911318641 | 0.910925289 | 0.974257382 |
|  | 25-50m |  | 0.96812577 | 1.010279376 | 0.980409275 | 1.029549907 |
|  | 50-75m |  | 1.011409215 | 0.948933372 | 1.00664351 | 1.004476689 |
|  | 75-100m |  | 0.992142503 | 1.03392835 | 1.023641572 | 0.962877949 |
|  | 100-125m |  | 1.019287379 | 0.956794229 | 1.053889629 | 1.089883407 |
|  | >125m |  | 1.004360756 | 0.986725706 | 1.001787627 | 0.998042526 |
|  | STDEV | 0.04221789 | 0.04226769 | 0.040661676 | 0.044239016 | 0.041703176 |
