# Supplementary material for: Human movement and gully erosion: Investigating feedback mechanisms using Frequency Ratio and Least Cost Path analysis in Tigray, Ethiopia
Source: PLoS One. 2021 Feb 5;16(2):e0245248. doi: 10.1371/journal.pone.0245248 (PMC7864406; doi:10.1371/journal.pone.0245248)
Supplement: S2 Table — (DOCX) [file pone.0245248.s005.docx]

S2 Table. Least Cost Paths (LCPs) for all sample units 1-4. N-S n=1000, E-W n=1000 (LCPs lengths: **UM L** = unmodified DEM, **M L** modified DEM; mean average conductivity for a LCP: **UM C** = unmodified DEM, **M C** = modified DEM; L Ch % = Length changes following DEM modification)

|  | Rama sample unite (1) N-S | | | | | Yeha sample unite (2) N-S | | | | |
| --- | --- | --- | --- | --- | --- | --- | --- | --- | --- | --- |
|  | UM L | M L | UM C | M C | L Ch % | UM L | M L | UM C | M C | L Ch % |
| 1 | 12849.11 | 13141.36 | 0.092 | 0.094 | 2.2239 | 13159.65 | 13956.21 | 0.114 | 0.12 | 5.7076 |
| 2 | 12565.03 | 12667.16 | 0.092 | 0.094 | 0.8063 | 15876.61 | 15953.8 | 0.103 | 0.108 | 0.4839 |
| 3 | 12547.18 | 13332.49 | 0.094 | 0.1 | 5.8902 | 11777.86 | 13001.78 | 0.1 | 0.084 | 9.4135 |
| 4 | 12223.19 | 12938.29 | 0.095 | 0.102 | 5.527 | 13168.58 | 13219.5 | 0.079 | 0.078 | 0.3852 |
| 5 | 13671.36 | 13962.91 | 0.096 | 0.096 | 2.088 | 12013.81 | 12013.4 | 0.088 | 0.096 | -0.0035 |
| 6 | 13285.3 | 13387.43 | 0.096 | 0.098 | 0.7629 | 14360.9 | 14325.04 | 0.083 | 0.084 | -0.2503 |
| 7 | 13645 | 13969.98 | 0.096 | 0.097 | 2.3262 | 15906.58 | 15897.29 | 0.099 | 0.103 | -0.0584 |
| 8 | 11801.06 | 12081.6 | 0.097 | 0.104 | 2.322 | 11316.28 | 11241.8 | 0.106 | 0.106 | -0.6625 |
| 9 | 12150.97 | 13012.05 | 0.097 | 0.108 | 6.6175 | 15917.04 | 16398.93 | 0.119 | 0.114 | 2.9385 |
| 10 | 12695.92 | 13026.13 | 0.098 | 0.1 | 2.535 | 11329.53 | 11881.61 | 0.122 | 0.121 | 4.6465 |
| 11 | 12524.97 | 13501.1 | 0.098 | 0.109 | 7.2301 | 11221.32 | 11383.36 | 0.123 | 0.122 | 1.4235 |
| 12 | 13525.33 | 13400.48 | 0.099 | 0.099 | -0.9317 | 13039.84 | 13149.46 | 0.117 | 0.122 | 0.8336 |
| 13 | 12095.71 | 11917.69 | 0.1 | 0.095 | -1.4938 | 11984.19 | 11984.19 | 0.077 | 0.077 | 0 |
| 14 | 11967.07 | 11920.5 | 0.1 | 0.1 | -0.3907 | 12880.13 | 13389.09 | 0.127 | 0.121 | 3.8013 |
| 15 | 13114.06 | 13015.77 | 0.1 | 0.101 | -0.7551 | 12011.62 | 12119.11 | 0.073 | 0.084 | 0.8869 |
| 16 | 12439.77 | 12393.2 | 0.1 | 0.1 | -0.3758 | 11400.57 | 12888.28 | 0.111 | 0.109 | 11.543 |
| 17 | 13305.75 | 13217.46 | 0.1 | 0.1 | -0.6679 | 11509.43 | 12505.53 | 0.069 | 0.072 | 7.9653 |
| 18 | 12371.21 | 12213.73 | 0.1 | 0.102 | -1.2894 | 10003.25 | 10193.76 | 0.128 | 0.128 | 1.8688 |
| 19 | 13033.75 | 13493.41 | 0.1 | 0.105 | 3.4065 | 11225.81 | 11208.74 | 0.093 | 0.09 | -0.1523 |
| 20 | 11727.15 | 11787.06 | 0.1 | 0.101 | 0.5083 | 13076.78 | 13861.55 | 0.075 | 0.08 | 5.6615 |
| 21 | 12334.5 | 12287.93 | 0.1 | 0.1 | -0.379 | 13023.75 | 13137.51 | 0.109 | 0.114 | 0.8659 |
| 22 | 13306.33 | 13978.29 | 0.1 | 0.099 | 4.8072 | 12344.34 | 12446.39 | 0.108 | 0.113 | 0.8199 |
| 23 | 12583.82 | 12537.26 | 0.1 | 0.101 | -0.3714 | 10949.28 | 11437.15 | 0.131 | 0.127 | 4.2656 |
| 24 | 12320.62 | 12716.72 | 0.1 | 0.107 | 3.1148 | 11993.54 | 11979.11 | 0.084 | 0.084 | -0.1205 |
| 25 | 13119.17 | 13340.38 | 0.1 | 0.103 | 1.6582 | 12070.54 | 12175.52 | 0.1 | 0.106 | 0.8622 |
| 26 | 13889.52 | 14040.18 | 0.1 | 0.098 | 1.0731 | 11539.22 | 11641.27 | 0.11 | 0.116 | 0.8766 |
| 27 | 12583.56 | 12546.69 | 0.1 | 0.102 | -0.2938 | 15025.33 | 15131.52 | 0.107 | 0.112 | 0.7018 |
| 28 | 12821.95 | 12157.9 | 0.101 | 0.098 | -5.4619 | 13289.14 | 13272.49 | 0.092 | 0.093 | -0.1255 |
| 29 | 13939.45 | 13851.16 | 0.101 | 0.101 | -0.6374 | 9589.615 | 9784.798 | 0.122 | 0.12 | 1.9948 |
| 30 | 12716.36 | 12770.09 | 0.101 | 0.095 | 0.4207 | 13784.85 | 14201.04 | 0.068 | 0.071 | 2.9307 |
| 31 | 12740.6 | 12757.97 | 0.101 | 0.101 | 0.1361 | 15244.6 | 15675.84 | 0.099 | 0.106 | 2.751 |
| 32 | 13830.39 | 13705.54 | 0.101 | 0.101 | -0.911 | 15967.75 | 16474.29 | 0.112 | 0.11 | 3.0747 |
| 33 | 12727.93 | 12960.69 | 0.101 | 0.101 | 1.7959 | 14643.62 | 14623.62 | 0.083 | 0.081 | -0.1368 |
| 34 | 13147.32 | 13549.03 | 0.101 | 0.101 | 2.9649 | 12109.22 | 12095.29 | 0.097 | 0.101 | -0.1152 |
| 35 | 13346.4 | 13999.57 | 0.101 | 0.105 | 4.6657 | 12159.12 | 12142.13 | 0.08 | 0.088 | -0.1399 |
| 36 | 12836.04 | 12981.48 | 0.101 | 0.101 | 1.1204 | 13001.6 | 13447.4 | 0.098 | 0.107 | 3.3152 |
| 37 | 13426.89 | 13930.06 | 0.101 | 0.1 | 3.6121 | 16005.21 | 16561.74 | 0.121 | 0.117 | 3.3604 |
| 38 | 12852.19 | 12826.3 | 0.101 | 0.097 | -0.2019 | 12148.47 | 12361.44 | 0.109 | 0.109 | 1.7228 |
| 39 | 13693.94 | 14758.41 | 0.101 | 0.101 | 7.2126 | 15594.34 | 15783.42 | 0.112 | 0.114 | 1.198 |
| 40 | 12433.14 | 12412.43 | 0.101 | 0.102 | -0.1669 | 10621.9 | 10493.83 | 0.108 | 0.115 | -1.2205 |
| 41 | 12658.52 | 12972.49 | 0.102 | 0.109 | 2.4203 | 11183.21 | 11943.27 | 0.12 | 0.12 | 6.3638 |
| 42 | 12769.62 | 12871.72 | 0.102 | 0.098 | 0.7932 | 15519.87 | 15897.27 | 0.1 | 0.106 | 2.374 |
| 43 | 13418.14 | 13007.01 | 0.102 | 0.097 | -3.1608 | 12688.21 | 12689.93 | 0.102 | 0.104 | 0.0135 |
| 44 | 12561.95 | 12926.42 | 0.102 | 0.109 | 2.8196 | 12094.28 | 12447.16 | 0.1 | 0.109 | 2.835 |
| 45 | 13796.13 | 13444 | 0.102 | 0.098 | -2.6193 | 10017.62 | 10350.42 | 0.131 | 0.123 | 3.2154 |
| 46 | 12841.63 | 12955.86 | 0.102 | 0.101 | 0.8817 | 17518.28 | 17575.23 | 0.11 | 0.111 | 0.324 |
| 47 | 13323.97 | 13645.1 | 0.102 | 0.102 | 2.3534 | 16113.06 | 16082.56 | 0.101 | 0.105 | -0.1897 |
| 48 | 11981 | 12111.92 | 0.102 | 0.105 | 1.0809 | 12245.68 | 13400.24 | 0.073 | 0.081 | 8.616 |

| 49 | 13614.71 | 13872.78 | 0.102 | 0.105 | 1.8603 | 11441.61 | 11452.65 | 0.105 | 0.114 | 0.0964 |
| --- | --- | --- | --- | --- | --- | --- | --- | --- | --- | --- |
| 50 | 12624.46 | 13221.57 | 0.103 | 0.108 | 4.5162 | 12386.62 | 12952.51 | 0.068 | 0.072 | 4.369 |
| 51 | 12590.48 | 12783.62 | 0.103 | 0.104 | 1.5108 | 14842.19 | 15272.06 | 0.093 | 0.103 | 2.8147 |
| 52 | 12562.87 | 12932.19 | 0.103 | 0.11 | 2.8559 | 9929.087 | 10043.14 | 0.127 | 0.122 | 1.1357 |
| 53 | 12922.58 | 13065.42 | 0.103 | 0.102 | 1.0933 | 10043.61 | 10055.33 | 0.134 | 0.13 | 0.1165 |
| 54 | 13136.4 | 13143.76 | 0.104 | 0.103 | 0.056 | 10931.71 | 11489.58 | 0.13 | 0.127 | 4.8554 |
| 55 | 13700.1 | 13815.87 | 0.104 | 0.104 | 0.838 | 13713.56 | 14610.66 | 0.114 | 0.11 | 6.1401 |
| 56 | 13038.35 | 12852.99 | 0.105 | 0.105 | -1.4421 | 15820.13 | 15976.24 | 0.102 | 0.111 | 0.9771 |
| 57 | 12607.04 | 12901.93 | 0.105 | 0.11 | 2.2856 | 12491.85 | 13573.05 | 0.094 | 0.081 | 7.9658 |
| 58 | 14145.07 | 14098.5 | 0.105 | 0.105 | -0.3303 | 11882.42 | 11958.48 | 0.093 | 0.094 | 0.6361 |
| 59 | 13049.29 | 13329.83 | 0.105 | 0.112 | 2.1046 | 13755.5 | 13447.13 | 0.065 | 0.067 | -2.2932 |
| 60 | 12512.18 | 13623.14 | 0.105 | 0.104 | 8.1549 | 12691.14 | 12662.36 | 0.095 | 0.092 | -0.2273 |
| 61 | 12118.96 | 12420 | 0.105 | 0.112 | 2.4238 | 15478.24 | 15871.11 | 0.094 | 0.101 | 2.4754 |
| 62 | 12659.92 | 12571.63 | 0.105 | 0.106 | -0.7022 | 12178.46 | 12155.74 | 0.086 | 0.087 | -0.1869 |
| 63 | 13015.98 | 12927.7 | 0.105 | 0.106 | -0.6829 | 13042.72 | 13423.89 | 0.105 | 0.118 | 2.8394 |
| 64 | 12283.94 | 12234.23 | 0.106 | 0.108 | -0.4063 | 12193.73 | 12139.29 | 0.091 | 0.092 | -0.4484 |
| 65 | 11922.71 | 12232.04 | 0.106 | 0.113 | 2.5288 | 12112.07 | 12186.72 | 0.129 | 0.127 | 0.6125 |
| 66 | 13542.49 | 13524.71 | 0.106 | 0.107 | -0.1315 | 12641.5 | 12739.4 | 0.117 | 0.122 | 0.7685 |
| 67 | 14183.77 | 14688.21 | 0.106 | 0.105 | 3.4343 | 14111.9 | 14277.13 | 0.085 | 0.093 | 1.1573 |
| 68 | 13423.17 | 13492.26 | 0.106 | 0.104 | 0.512 | 15705.13 | 16128.9 | 0.101 | 0.11 | 2.6273 |
| 69 | 12726.95 | 12884.65 | 0.106 | 0.103 | 1.2239 | 12975.24 | 13828.11 | 0.067 | 0.073 | 6.1677 |
| 70 | 13624.83 | 13954.45 | 0.106 | 0.112 | 2.3621 | 13229.62 | 13166.9 | 0.09 | 0.091 | -0.4764 |
| 71 | 12596.92 | 12963.85 | 0.106 | 0.109 | 2.8304 | 12599.44 | 12576.72 | 0.092 | 0.093 | -0.1807 |
| 72 | 13096.42 | 12947.72 | 0.107 | 0.108 | -1.1485 | 10295.28 | 11034.44 | 0.128 | 0.128 | 6.6987 |
| 73 | 13718.49 | 13872.55 | 0.107 | 0.104 | 1.1105 | 12913.46 | 13015.5 | 0.11 | 0.115 | 0.784 |
| 74 | 13347.25 | 14102.26 | 0.107 | 0.109 | 5.3538 | 16838.76 | 17007.13 | 0.105 | 0.112 | 0.99 |
| 75 | 13535.15 | 13772.05 | 0.107 | 0.102 | 1.7201 | 12981.34 | 13999.2 | 0.077 | 0.095 | 7.2709 |
| 76 | 13322.04 | 13719.06 | 0.107 | 0.11 | 2.8939 | 11825.94 | 11904.72 | 0.08 | 0.084 | 0.6618 |
| 77 | 13636.63 | 13569.98 | 0.107 | 0.106 | -0.4912 | 11209.34 | 11571.09 | 0.111 | 0.115 | 3.1263 |
| 78 | 12343.21 | 12802.16 | 0.107 | 0.11 | 3.5849 | 11749.05 | 11686.33 | 0.093 | 0.093 | -0.5367 |
| 79 | 12884.08 | 13684.41 | 0.107 | 0.107 | 5.8485 | 13183.14 | 13120.42 | 0.092 | 0.092 | -0.478 |
| 80 | 12039.64 | 12846.36 | 0.107 | 0.114 | 6.2798 | 15297.82 | 15672.29 | 0.1 | 0.106 | 2.3894 |
| 81 | 12927.93 | 13302.87 | 0.107 | 0.099 | 2.8185 | 11230.62 | 11207.9 | 0.092 | 0.093 | -0.2027 |
| 82 | 12985.18 | 12987.4 | 0.107 | 0.107 | 0.0171 | 13802.22 | 13904.27 | 0.111 | 0.116 | 0.7339 |
| 83 | 11947.18 | 12248.23 | 0.107 | 0.114 | 2.4578 | 13036.77 | 13138.81 | 0.118 | 0.123 | 0.7767 |
| 84 | 12894.88 | 12884.5 | 0.107 | 0.103 | -0.0806 | 13049.56 | 13282.58 | 0.077 | 0.087 | 1.7543 |
| 85 | 13305.8 | 13300.44 | 0.107 | 0.108 | -0.0403 | 13079.38 | 13287.07 | 0.109 | 0.116 | 1.5631 |
| 86 | 12938.64 | 13816.34 | 0.107 | 0.106 | 6.3526 | 11428.53 | 11439.58 | 0.107 | 0.116 | 0.0965 |
| 87 | 13642.25 | 14097.88 | 0.108 | 0.109 | 3.2319 | 10648.29 | 10964.01 | 0.124 | 0.127 | 2.8796 |
| 88 | 12786.27 | 13935.12 | 0.108 | 0.111 | 8.2443 | 10797.97 | 10810.4 | 0.12 | 0.118 | 0.1149 |
| 89 | 14015.27 | 14186.1 | 0.108 | 0.104 | 1.2042 | 16898.72 | 17097.51 | 0.111 | 0.111 | 1.1627 |
| 90 | 13392.76 | 13577.91 | 0.108 | 0.109 | 1.3636 | 14601.26 | 15037.41 | 0.115 | 0.117 | 2.9004 |
| 91 | 13300.27 | 13237.76 | 0.108 | 0.107 | -0.4722 | 13922.8 | 14857.12 | 0.118 | 0.113 | 6.2887 |
| 92 | 12684.83 | 12697.84 | 0.108 | 0.106 | 0.1025 | 17281.57 | 16714.08 | 0.109 | 0.11 | -3.3953 |
| 93 | 13603.14 | 14008.62 | 0.108 | 0.114 | 2.8945 | 12274.52 | 12223.02 | 0.09 | 0.091 | -0.4214 |
| 94 | 13416.69 | 13337.44 | 0.108 | 0.103 | -0.5942 | 12958.73 | 13060.77 | 0.117 | 0.122 | 0.7813 |
| 95 | 13293.97 | 13267.91 | 0.108 | 0.108 | -0.1965 | 12431.56 | 13579.92 | 0.076 | 0.083 | 8.4563 |
| 96 | 12537.98 | 12813.58 | 0.108 | 0.114 | 2.1508 | 12169.82 | 12267.6 | 0.078 | 0.078 | 0.7971 |
| 97 | 13145.57 | 13557.61 | 0.108 | 0.105 | 3.0392 | 11520.73 | 11902.39 | 0.097 | 0.106 | 3.2066 |
| 98 | 13869.77 | 13969.68 | 0.108 | 0.106 | 0.7152 | 12893.49 | 13056.51 | 0.086 | 0.095 | 1.2485 |

| 99 | 13224.46 | 14101.61 | 0.109 | 0.111 | 6.2202 | 16307.29 | 16172.85 | 0.104 | 0.107 | -0.8312 |
| --- | --- | --- | --- | --- | --- | --- | --- | --- | --- | --- |
| 100 | 13721.1 | 14744.87 | 0.109 | 0.108 | 6.9432 | 11681 | 11859.99 | 0.076 | 0.081 | 1.5092 |
| 101 | 13902.11 | 14517.55 | 0.109 | 0.105 | 4.2393 | 11969.32 | 11932.33 | 0.09 | 0.095 | -0.31 |
| 102 | 12626.3 | 12887.84 | 0.109 | 0.108 | 2.0294 | 14106.03 | 14027.66 | 0.097 | 0.103 | -0.5587 |
| 103 | 14042.34 | 13914.3 | 0.109 | 0.105 | -0.9202 | 14096.55 | 14025.54 | 0.09 | 0.089 | -0.5063 |
| 104 | 13840.18 | 13834.12 | 0.109 | 0.109 | -0.0438 | 15704.72 | 15511.29 | 0.103 | 0.108 | -1.247 |
| 105 | 13711.84 | 13694.06 | 0.109 | 0.109 | -0.1299 | 15866.29 | 15319.63 | 0.082 | 0.084 | -3.5683 |
| 106 | 12483.67 | 12967.01 | 0.109 | 0.116 | 3.7275 | 13427.17 | 13797.46 | 0.113 | 0.116 | 2.6838 |
| 107 | 14252.76 | 14552.59 | 0.109 | 0.106 | 2.0603 | 13168.79 | 13270.83 | 0.117 | 0.122 | 0.7689 |
| 108 | 13146.4 | 13045.77 | 0.109 | 0.11 | -0.7713 | 12154.05 | 12140.11 | 0.115 | 0.119 | -0.1148 |
| 109 | 13044.83 | 13080.06 | 0.109 | 0.106 | 0.2694 | 14403.17 | 14393.38 | 0.106 | 0.109 | -0.068 |
| 110 | 13445.25 | 13551.9 | 0.109 | 0.107 | 0.787 | 15534.51 | 15528.27 | 0.104 | 0.109 | -0.0402 |
| 111 | 12827.75 | 13141.21 | 0.109 | 0.116 | 2.3854 | 11984.39 | 11943.64 | 0.12 | 0.121 | -0.3412 |
| 112 | 13404.39 | 13514.68 | 0.109 | 0.11 | 0.8161 | 14126.28 | 14138.91 | 0.106 | 0.109 | 0.0894 |
| 113 | 14148.03 | 14768.03 | 0.109 | 0.106 | 4.1983 | 12086.71 | 12118.63 | 0.088 | 0.086 | 0.2634 |
| 114 | 13299.39 | 13337.26 | 0.11 | 0.104 | 0.2839 | 10592.8 | 10881.55 | 0.117 | 0.115 | 2.6536 |
| 115 | 13018.41 | 13026.48 | 0.11 | 0.11 | 0.062 | 14469.53 | 13379.95 | 0.081 | 0.086 | -8.1434 |
| 116 | 13778.8 | 14287.79 | 0.11 | 0.112 | 3.5624 | 10777.48 | 11457.82 | 0.11 | 0.109 | 5.9378 |
| 117 | 12948.58 | 13437.78 | 0.11 | 0.117 | 3.6405 | 11618.42 | 11650.85 | 0.087 | 0.087 | 0.2783 |
| 118 | 14433.03 | 15355.58 | 0.111 | 0.108 | 6.0079 | 14448.16 | 14145.58 | 0.117 | 0.114 | -2.1391 |
| 119 | 14381.7 | 15626.05 | 0.111 | 0.109 | 7.9633 | 12776.15 | 13143.43 | 0.122 | 0.126 | 2.7944 |
| 120 | 13210.36 | 14742.82 | 0.111 | 0.115 | 10.395 | 18069.56 | 18284.91 | 0.11 | 0.11 | 1.1778 |
| 121 | 14154.07 | 13671.28 | 0.115 | 0.099 | -3.5314 | 12428.9 | 12603.67 | 0.072 | 0.082 | 1.3866 |
| 122 | 13845.12 | 13452.99 | 0.115 | 0.101 | -2.9148 | 14181.89 | 14719.63 | 0.118 | 0.113 | 3.6533 |
| 123 | 12980.87 | 13075.31 | 0.116 | 0.114 | 0.7223 | 11068.5 | 11234.18 | 0.113 | 0.112 | 1.4748 |
| 124 | 14249.22 | 13593.7 | 0.116 | 0.101 | -4.8222 | 12282.39 | 12341.6 | 0.084 | 0.086 | 0.4797 |
| 125 | 13323.32 | 12667.81 | 0.117 | 0.1 | -5.1746 | 13330.27 | 13454.33 | 0.077 | 0.075 | 0.9221 |
| 126 | 15514.46 | 15752.54 | 0.118 | 0.117 | 1.5114 | 14482.61 | 14860.93 | 0.108 | 0.111 | 2.5457 |
| 127 | 12941.9 | 12577.6 | 0.118 | 0.102 | -2.8964 | 13742.55 | 13728.62 | 0.108 | 0.112 | -0.1015 |
| 128 | 15361.64 | 14121.69 | 0.118 | 0.099 | -8.7805 | 11496.83 | 11943.78 | 0.128 | 0.123 | 3.7421 |
| 129 | 13046.55 | 12653.96 | 0.118 | 0.103 | -3.1025 | 13964.27 | 13912.76 | 0.086 | 0.086 | -0.3702 |
| 130 | 16642.15 | 16892.66 | 0.118 | 0.118 | 1.4829 | 10043.25 | 10303.71 | 0.127 | 0.125 | 2.5278 |
| 131 | 17766.77 | 18066.98 | 0.119 | 0.117 | 1.6616 | 13477.33 | 14088.5 | 0.117 | 0.112 | 4.3381 |
| 132 | 14450.07 | 14494.8 | 0.119 | 0.119 | 0.3086 | 9902.91 | 10383.79 | 0.13 | 0.121 | 4.6311 |
| 133 | 13455.98 | 13116.66 | 0.119 | 0.103 | -2.587 | 10968.06 | 11070.1 | 0.102 | 0.109 | 0.9218 |
| 134 | 14649.55 | 14900.05 | 0.119 | 0.119 | 1.6812 | 11405.49 | 11466.79 | 0.069 | 0.082 | 0.5346 |
| 135 | 13549.79 | 13718.7 | 0.119 | 0.122 | 1.2312 | 12360.29 | 12270.7 | 0.103 | 0.111 | -0.7301 |
| 136 | 16041.53 | 16292.03 | 0.119 | 0.119 | 1.5376 | 10845.31 | 11097.15 | 0.078 | 0.089 | 2.2694 |
| 137 | 12858.23 | 12553.46 | 0.119 | 0.103 | -2.4278 | 14141.48 | 13958.05 | 0.108 | 0.113 | -1.3142 |
| 138 | 13502.51 | 14575.87 | 0.12 | 0.124 | 7.364 | 12501.41 | 12904.08 | 0.105 | 0.109 | 3.1205 |
| 139 | 14155.83 | 14230.43 | 0.12 | 0.111 | 0.5242 | 13673.11 | 14029.8 | 0.103 | 0.107 | 2.5424 |
| 140 | 17008.87 | 17263.72 | 0.12 | 0.119 | 1.4762 | 14191.04 | 14128.32 | 0.09 | 0.09 | -0.4439 |
| 141 | 13467.7 | 13785.18 | 0.12 | 0.122 | 2.3031 | 13202.64 | 13414.94 | 0.084 | 0.092 | 1.5826 |
| 142 | 15522.54 | 15801.82 | 0.12 | 0.119 | 1.7674 | 11026.16 | 11337.32 | 0.124 | 0.122 | 2.7446 |
| 143 | 16782.66 | 17082.87 | 0.12 | 0.119 | 1.7574 | 11960.95 | 11992.16 | 0.084 | 0.084 | 0.2603 |
| 144 | 14192.78 | 15270.55 | 0.12 | 0.116 | 7.0578 | 11559.63 | 11665.81 | 0.103 | 0.109 | 0.9102 |
| 145 | 16266.89 | 16517.39 | 0.12 | 0.12 | 1.5166 | 11422.59 | 11433.63 | 0.11 | 0.119 | 0.0966 |
| 146 | 14301.58 | 14540.37 | 0.12 | 0.119 | 1.6422 | 12397.25 | 13309.38 | 0.079 | 0.086 | 6.8533 |
| 147 | 14352.14 | 14590.93 | 0.12 | 0.12 | 1.6365 | 16219.66 | 16185.55 | 0.105 | 0.109 | -0.2107 |
| 148 | 15963.52 | 16456.03 | 0.12 | 0.121 | 2.9929 | 12846.66 | 12850.38 | 0.091 | 0.096 | 0.029 |

| 149 | 12480.36 | 12211.24 | 0.12 | 0.104 | -2.2038 | 12530.17 | 12632.22 | 0.111 | 0.116 | 0.8078 |
| --- | --- | --- | --- | --- | --- | --- | --- | --- | --- | --- |
| 150 | 14579.69 | 14784.84 | 0.12 | 0.12 | 1.3876 | 15319.06 | 15717.29 | 0.101 | 0.108 | 2.5337 |
| 151 | 13947.44 | 14152.59 | 0.12 | 0.12 | 1.4495 | 13249.04 | 13635.07 | 0.127 | 0.123 | 2.8312 |
| 152 | 13077.84 | 12738.52 | 0.12 | 0.103 | -2.6638 | 12519.67 | 12512.6 | 0.084 | 0.084 | -0.0565 |
| 153 | 14088.62 | 15495.05 | 0.121 | 0.121 | 9.0767 | 12925.24 | 13027.28 | 0.11 | 0.115 | 0.7833 |
| 154 | 14049.62 | 14862.92 | 0.121 | 0.11 | 5.472 | 14441.21 | 14839.45 | 0.101 | 0.108 | 2.6836 |
| 155 | 15878.75 | 16145.11 | 0.121 | 0.12 | 1.6498 | 11825.88 | 11837.9 | 0.089 | 0.095 | 0.1015 |
| 156 | 12774.5 | 13071.99 | 0.121 | 0.123 | 2.2758 | 12482.39 | 12455.53 | 0.087 | 0.088 | -0.2157 |
| 157 | 12971.47 | 13960.9 | 0.121 | 0.125 | 7.0871 | 11876.98 | 11979.02 | 0.115 | 0.121 | 0.8519 |
| 158 | 17052.62 | 17220.11 | 0.121 | 0.12 | 0.9726 | 10261.37 | 10272.41 | 0.104 | 0.114 | 0.1075 |
| 159 | 16188.95 | 16563.75 | 0.121 | 0.119 | 2.2628 | 10911.7 | 11504.12 | 0.106 | 0.113 | 5.1497 |
| 160 | 14322.88 | 14400.66 | 0.121 | 0.12 | 0.5401 | 11105.43 | 11078.57 | 0.09 | 0.09 | -0.2425 |
| 161 | 15214.99 | 15459.04 | 0.121 | 0.12 | 1.5787 | 9947.372 | 10022.64 | 0.127 | 0.125 | 0.751 |
| 162 | 15275.99 | 15526.49 | 0.121 | 0.12 | 1.6134 | 15201.35 | 14908.12 | 0.081 | 0.085 | -1.9669 |
| 163 | 16442.89 | 16743.1 | 0.121 | 0.119 | 1.793 | 12438.1 | 12776.98 | 0.123 | 0.118 | 2.6522 |
| 164 | 12242.37 | 12184.55 | 0.121 | 0.103 | -0.4745 | 11697.7 | 11931.38 | 0.109 | 0.108 | 1.9585 |
| 165 | 16799.67 | 17148.87 | 0.121 | 0.12 | 2.0363 | 11898.24 | 12384.43 | 0.095 | 0.103 | 3.9258 |
| 166 | 12984.5 | 13428.35 | 0.121 | 0.123 | 3.3053 | 12643.73 | 12745.77 | 0.115 | 0.12 | 0.8006 |
| 167 | 13931.04 | 13604.01 | 0.121 | 0.115 | -2.404 | 12873.93 | 13805.09 | 0.094 | 0.105 | 6.7451 |
| 168 | 14065.77 | 15143.54 | 0.121 | 0.116 | 7.117 | 15375.05 | 15723.63 | 0.115 | 0.117 | 2.2169 |
| 169 | 14051.58 | 14302.08 | 0.121 | 0.12 | 1.7515 | 12681.01 | 12723.02 | 0.084 | 0.091 | 0.3302 |
| 170 | 14955.45 | 13392.49 | 0.121 | 0.1 | -11.67 | 16163.88 | 16701.42 | 0.116 | 0.112 | 3.2185 |
| 171 | 14510.84 | 14743.06 | 0.121 | 0.12 | 1.5751 | 13853.9 | 13622.73 | 0.118 | 0.116 | -1.6969 |
| 172 | 14411.94 | 14662.44 | 0.121 | 0.121 | 1.7085 | 12427.16 | 12897.99 | 0.068 | 0.077 | 3.6504 |
| 173 | 14618.86 | 14869.36 | 0.121 | 0.12 | 1.6847 | 11890.54 | 11901.59 | 0.107 | 0.116 | 0.0928 |
| 174 | 13369.29 | 14095.93 | 0.121 | 0.121 | 5.155 | 10989.69 | 11184.87 | 0.123 | 0.121 | 1.7451 |
| 175 | 12978.56 | 12639.23 | 0.121 | 0.104 | -2.6847 | 10748.21 | 11076.88 | 0.122 | 0.117 | 2.9671 |
| 176 | 16482.68 | 16610.17 | 0.121 | 0.12 | 0.7675 | 11094.39 | 11344.01 | 0.073 | 0.081 | 2.2005 |
| 177 | 13486.42 | 13581.94 | 0.121 | 0.113 | 0.7032 | 13110.44 | 13933.82 | 0.073 | 0.083 | 5.9092 |
| 178 | 12802.16 | 12946.42 | 0.121 | 0.11 | 1.1143 | 15992.98 | 15861.97 | 0.083 | 0.084 | -0.8259 |
| 179 | 14889.93 | 15140.43 | 0.121 | 0.121 | 1.6545 | 12395.54 | 12622.7 | 0.12 | 0.12 | 1.7996 |
| 180 | 15927.77 | 16174.34 | 0.121 | 0.12 | 1.5244 | 12598.99 | 12712.75 | 0.117 | 0.122 | 0.8949 |
| 181 | 14526.51 | 15429.63 | 0.121 | 0.117 | 5.8532 | 10994.28 | 10550.85 | 0.104 | 0.091 | -4.2028 |
| 182 | 16737.62 | 16905.11 | 0.121 | 0.12 | 0.9908 | 12763.61 | 12829.17 | 0.092 | 0.094 | 0.5111 |
| 183 | 13594.73 | 14868.44 | 0.121 | 0.123 | 8.5665 | 14382.67 | 14359.95 | 0.084 | 0.085 | -0.1582 |
| 184 | 15440.04 | 14834.34 | 0.121 | 0.104 | -4.0831 | 12007.07 | 12025.95 | 0.084 | 0.094 | 0.1569 |
| 185 | 13439.62 | 13557.4 | 0.121 | 0.121 | 0.8688 | 13633.43 | 13735.48 | 0.111 | 0.116 | 0.7429 |
| 186 | 15184.75 | 15435.26 | 0.121 | 0.121 | 1.6229 | 15785.43 | 15771.49 | 0.101 | 0.106 | -0.0883 |
| 187 | 13446.84 | 14567.71 | 0.121 | 0.121 | 7.6942 | 11564.39 | 11732.88 | 0.096 | 0.101 | 1.4361 |
| 188 | 13027.84 | 12688.52 | 0.121 | 0.104 | -2.6743 | 11933.45 | 11957.09 | 0.123 | 0.115 | 0.1977 |
| 189 | 13507.93 | 14585.7 | 0.122 | 0.117 | 7.3892 | 13535.77 | 13637.82 | 0.11 | 0.115 | 0.7483 |
| 190 | 14548.05 | 15821.76 | 0.122 | 0.123 | 8.0504 | 11278.63 | 11609.67 | 0.113 | 0.117 | 2.8514 |
| 191 | 17585.88 | 17753.37 | 0.122 | 0.121 | 0.9434 | 12628.4 | 13010.06 | 0.099 | 0.108 | 2.9336 |
| 192 | 15159.89 | 13985.58 | 0.122 | 0.106 | -8.3966 | 11272.33 | 11249.61 | 0.082 | 0.083 | -0.202 |
| 193 | 13911.15 | 14648.71 | 0.122 | 0.121 | 5.035 | 10458.2 | 10553.47 | 0.129 | 0.131 | 0.9027 |
| 194 | 13666.48 | 14118.32 | 0.122 | 0.124 | 3.2004 | 10774.21 | 10936.26 | 0.119 | 0.117 | 1.4817 |
| 195 | 16552.89 | 17067.03 | 0.122 | 0.121 | 3.0125 | 13182.31 | 13284.35 | 0.108 | 0.113 | 0.7682 |
| 196 | 13007.72 | 13190.57 | 0.122 | 0.125 | 1.3862 | 9720.803 | 9853.351 | 0.127 | 0.125 | 1.3452 |
| 197 | 15658 | 15825.49 | 0.122 | 0.121 | 1.0583 | 12138.8 | 12275.12 | 0.118 | 0.119 | 1.1106 |
| 198 | 13519.62 | 13684.06 | 0.122 | 0.123 | 1.2017 | 13029.35 | 13702.07 | 0.114 | 0.119 | 4.9096 |

| 199 | 13729.24 | 13979.74 | 0.122 | 0.121 | 1.7919 | 13641.81 | 13701.9 | 0.085 | 0.094 | 0.4385 |
| --- | --- | --- | --- | --- | --- | --- | --- | --- | --- | --- |
| 200 | 14440.78 | 14715.64 | 0.122 | 0.121 | 1.8678 | 14518.97 | 14909.01 | 0.107 | 0.111 | 2.6161 |
| 201 | 14692.91 | 15966.62 | 0.122 | 0.123 | 7.9773 | 13821.57 | 14603.03 | 0.067 | 0.07 | 5.3513 |
| 202 | 17846.3 | 18013.78 | 0.122 | 0.121 | 0.9298 | 11488.23 | 11629.73 | 0.126 | 0.126 | 1.2168 |
| 203 | 12438.73 | 11907.98 | 0.122 | 0.104 | -4.4571 | 12818.4 | 12953.46 | 0.084 | 0.086 | 1.0427 |
| 204 | 13567.22 | 14644.99 | 0.122 | 0.117 | 7.3593 | 14983 | 14852 | 0.083 | 0.084 | -0.8821 |
| 205 | 15348.06 | 15506.85 | 0.122 | 0.124 | 1.024 | 13068.52 | 13182.28 | 0.117 | 0.122 | 0.863 |
| 206 | 11985.03 | 13161.75 | 0.122 | 0.124 | 8.9405 | 13361.48 | 13307.05 | 0.087 | 0.088 | -0.4091 |
| 207 | 14201.39 | 13377.16 | 0.122 | 0.1 | -6.1615 | 14631.84 | 14162.76 | 0.096 | 0.078 | -3.3121 |
| 208 | 14728.5 | 14967.29 | 0.122 | 0.122 | 1.5954 | 10864.24 | 10964.45 | 0.09 | 0.089 | 0.9139 |
| 209 | 14500.51 | 14618.3 | 0.122 | 0.122 | 0.8057 | 11833.24 | 12813.48 | 0.073 | 0.076 | 7.6501 |
| 210 | 15574.69 | 15692.47 | 0.123 | 0.122 | 0.7506 | 11454.72 | 12178.4 | 0.068 | 0.073 | 5.9423 |
| 211 | 14018.35 | 14843.48 | 0.123 | 0.122 | 5.5589 | 12534.91 | 12887.78 | 0.1 | 0.109 | 2.7381 |
| 212 | 11978.87 | 11758.04 | 0.123 | 0.101 | -1.8781 | 12847.77 | 12778.19 | 0.101 | 0.108 | -0.5446 |
| 213 | 13059.2 | 13176.99 | 0.123 | 0.123 | 0.8938 | 11619.84 | 11704.49 | 0.092 | 0.094 | 0.7232 |
| 214 | 14063.91 | 14909.55 | 0.123 | 0.123 | 5.6718 | 11958.58 | 12086.86 | 0.083 | 0.088 | 1.0614 |
| 215 | 15719.13 | 15846.62 | 0.123 | 0.121 | 0.8045 | 9990.803 | 10138 | 0.123 | 0.119 | 1.4519 |
| 216 | 13401.3 | 14272.29 | 0.123 | 0.122 | 6.1027 | 15090.93 | 14959.93 | 0.084 | 0.085 | -0.8757 |
| 217 | 15543.68 | 14379.48 | 0.123 | 0.106 | -8.0962 | 12534.11 | 12675.03 | 0.127 | 0.129 | 1.1118 |
| 218 | 13666.45 | 14644.22 | 0.123 | 0.118 | 6.6768 | 11326.62 | 11303.9 | 0.092 | 0.092 | -0.201 |
| 219 | 13668.47 | 14010.39 | 0.123 | 0.121 | 2.4405 | 11681.08 | 12022.75 | 0.104 | 0.113 | 2.8418 |
| 220 | 12588.14 | 12705.92 | 0.123 | 0.123 | 0.927 | 12845.63 | 13003.7 | 0.12 | 0.119 | 1.2156 |
| 221 | 14763.91 | 13648.8 | 0.123 | 0.105 | -8.17 | 11410.84 | 11421.88 | 0.104 | 0.114 | 0.0967 |
| 222 | 12282.6 | 13558.74 | 0.123 | 0.123 | 9.4119 | 13907.13 | 13884.71 | 0.085 | 0.083 | -0.1615 |
| 223 | 15495.13 | 15622.62 | 0.123 | 0.121 | 0.816 | 14158.62 | 14377.23 | 0.085 | 0.088 | 1.5206 |
| 224 | 12973.49 | 13091.28 | 0.123 | 0.123 | 0.8997 | 13821.07 | 13906.63 | 0.072 | 0.076 | 0.6153 |
| 225 | 14305.63 | 13916.79 | 0.123 | 0.114 | -2.794 | 11393.1 | 12526.38 | 0.116 | 0.119 | 9.0471 |
| 226 | 17441.65 | 17761.86 | 0.123 | 0.122 | 1.8028 | 12193.23 | 12170.5 | 0.087 | 0.088 | -0.1867 |
| 227 | 13908.99 | 14811.1 | 0.123 | 0.119 | 6.0908 | 11065.9 | 11057.62 | 0.093 | 0.09 | -0.0749 |
| 228 | 13028.43 | 14271.43 | 0.123 | 0.124 | 8.7097 | 10938.78 | 11692.54 | 0.1 | 0.083 | 6.4465 |
| 229 | 13223.35 | 13790.87 | 0.123 | 0.124 | 4.1152 | 12504.52 | 12623.43 | 0.086 | 0.085 | 0.942 |
| 230 | 14708.71 | 15358.04 | 0.123 | 0.12 | 4.2279 | 13517.72 | 14101.19 | 0.113 | 0.11 | 4.1377 |
| 231 | 14489.74 | 14607.52 | 0.123 | 0.123 | 0.8063 | 10620.63 | 10782.68 | 0.123 | 0.12 | 1.5028 |
| 232 | 14065.21 | 13477.88 | 0.123 | 0.104 | -4.3577 | 11163.18 | 11195.1 | 0.095 | 0.093 | 0.2852 |
| 233 | 13606.37 | 13866.88 | 0.123 | 0.122 | 1.8786 | 11588.13 | 11566.92 | 0.076 | 0.076 | -0.1834 |
| 234 | 13167.87 | 14401.08 | 0.123 | 0.118 | 8.5633 | 10114.86 | 10398.29 | 0.126 | 0.12 | 2.7258 |
| 235 | 12912.31 | 13139 | 0.123 | 0.126 | 1.7253 | 13504.71 | 13586.63 | 0.072 | 0.076 | 0.603 |
| 236 | 15210.18 | 14188.71 | 0.123 | 0.106 | -7.1992 | 13000.92 | 12614.35 | 0.084 | 0.089 | -3.0645 |
| 237 | 12089.79 | 11826.83 | 0.123 | 0.106 | -2.2235 | 13396.57 | 14100.71 | 0.114 | 0.12 | 4.9937 |
| 238 | 13981.08 | 14098.86 | 0.123 | 0.123 | 0.8354 | 10430.92 | 10441.97 | 0.106 | 0.116 | 0.1057 |
| 239 | 11430.64 | 11727.83 | 0.123 | 0.127 | 2.5341 | 13579.77 | 13565.83 | 0.114 | 0.118 | -0.1027 |
| 240 | 15485.93 | 15653.42 | 0.123 | 0.122 | 1.07 | 12832.64 | 12809.92 | 0.088 | 0.089 | -0.1774 |
| 241 | 16149.78 | 16317.27 | 0.123 | 0.123 | 1.0264 | 11926.8 | 11830.65 | 0.087 | 0.086 | -0.8127 |
| 242 | 13069.02 | 13807.59 | 0.123 | 0.122 | 5.349 | 14632.82 | 14591.02 | 0.106 | 0.11 | -0.2865 |
| 243 | 13309.12 | 13386.9 | 0.124 | 0.123 | 0.581 | 11843.41 | 11945.46 | 0.102 | 0.107 | 0.8543 |
| 244 | 13093.47 | 13359.83 | 0.124 | 0.123 | 1.9937 | 16264.34 | 16258.1 | 0.104 | 0.109 | -0.0384 |
| 245 | 13215.18 | 13481.04 | 0.124 | 0.123 | 1.9721 | 12415.3 | 12392.58 | 0.086 | 0.087 | -0.1833 |
| 246 | 15433 | 15600.49 | 0.124 | 0.123 | 1.0736 | 12203.59 | 12856.19 | 0.121 | 0.116 | 5.0761 |
| 247 | 12429.97 | 13563.89 | 0.124 | 0.126 | 8.3598 | 12766.74 | 13548.2 | 0.064 | 0.068 | 5.768 |
| 248 | 12394.26 | 13087.46 | 0.124 | 0.124 | 5.2967 | 11510.69 | 11571.61 | 0.123 | 0.121 | 0.5265 |

| 249 | 14570.07 | 14947.14 | 0.124 | 0.123 | 2.5227 | 12065.05 | 12167.1 | 0.113 | 0.119 | 0.8387 |
| --- | --- | --- | --- | --- | --- | --- | --- | --- | --- | --- |
| 250 | 16056.79 | 16224.28 | 0.124 | 0.123 | 1.0323 | 17142.18 | 17734.85 | 0.1 | 0.103 | 3.3418 |
| 251 | 15858.27 | 16025.76 | 0.124 | 0.123 | 1.0451 | 10388.06 | 10583.24 | 0.125 | 0.123 | 1.8443 |
| 252 | 13309.56 | 14376.29 | 0.124 | 0.124 | 7.42 | 13064.5 | 13159.47 | 0.13 | 0.125 | 0.7217 |
| 253 | 13214.68 | 13549.53 | 0.124 | 0.123 | 2.4713 | 13122.16 | 13224.2 | 0.109 | 0.114 | 0.7717 |
| 254 | 13562.13 | 13679.92 | 0.124 | 0.124 | 0.861 | 15831.49 | 16403.42 | 0.108 | 0.111 | 3.4866 |
| 255 | 12102.17 | 12729.35 | 0.124 | 0.124 | 4.927 | 12353.19 | 12455.24 | 0.115 | 0.121 | 0.8193 |
| 256 | 11899.61 | 13232.91 | 0.124 | 0.127 | 10.076 | 14006.49 | 14103.44 | 0.116 | 0.119 | 0.6874 |
| 257 | 11862.39 | 13108.41 | 0.124 | 0.128 | 9.5055 | 13352.26 | 13136.61 | 0.086 | 0.09 | -1.6416 |
| 258 | 15864.25 | 16270.91 | 0.124 | 0.123 | 2.4993 | 11445.11 | 11857.12 | 0.131 | 0.126 | 3.4748 |
| 259 | 15988.71 | 16156.2 | 0.124 | 0.123 | 1.0367 | 12288.9 | 12377.98 | 0.087 | 0.095 | 0.7197 |
| 260 | 15814.55 | 16105.76 | 0.124 | 0.123 | 1.8081 | 13008.08 | 12985.36 | 0.08 | 0.081 | -0.175 |
| 261 | 14030.36 | 15108.12 | 0.124 | 0.119 | 7.1337 | 18306.8 | 18292.87 | 0.097 | 0.101 | -0.0762 |
| 262 | 13753.97 | 13871.75 | 0.124 | 0.124 | 0.8491 | 16396.41 | 15897.54 | 0.103 | 0.083 | -3.1381 |
| 263 | 13900 | 14977.77 | 0.124 | 0.119 | 7.1958 | 11839.24 | 12454.56 | 0.123 | 0.119 | 4.9405 |
| 264 | 16196.49 | 16363.98 | 0.124 | 0.123 | 1.0235 | 11850.55 | 12053.78 | 0.073 | 0.077 | 1.686 |
| 265 | 13634.27 | 13752.05 | 0.124 | 0.124 | 0.8565 | 15246.04 | 14872.73 | 0.103 | 0.112 | -2.51 |
| 266 | 15395.81 | 15563.3 | 0.124 | 0.123 | 1.0762 | 16009.42 | 15961.85 | 0.081 | 0.082 | -0.298 |
| 267 | 11466.06 | 11418.99 | 0.124 | 0.107 | -0.4122 | 13392.84 | 13738.53 | 0.105 | 0.111 | 2.5162 |
| 268 | 12611.51 | 12729.29 | 0.124 | 0.124 | 0.9253 | 13091.68 | 13193.73 | 0.117 | 0.122 | 0.7734 |
| 269 | 14198.37 | 15493.42 | 0.124 | 0.118 | 8.3587 | 12053.78 | 12155.82 | 0.115 | 0.12 | 0.8395 |
| 270 | 12938.54 | 13916.31 | 0.124 | 0.119 | 7.026 | 12487.04 | 12589.08 | 0.118 | 0.123 | 0.8106 |
| 271 | 15082.82 | 15250.31 | 0.125 | 0.123 | 1.0983 | 12305.68 | 12407.72 | 0.112 | 0.117 | 0.8224 |
| 272 | 15062.73 | 15253.15 | 0.125 | 0.124 | 1.2484 | 11447.83 | 11761.8 | 0.11 | 0.115 | 2.6694 |
| 273 | 13741.33 | 12928.31 | 0.125 | 0.101 | -6.2886 | 13431.73 | 13459.34 | 0.104 | 0.105 | 0.2051 |
| 274 | 13795.69 | 13913.47 | 0.125 | 0.125 | 0.8465 | 12350.17 | 12229.38 | 0.095 | 0.094 | -0.9878 |
| 275 | 13617.82 | 13735.6 | 0.125 | 0.125 | 0.8575 | 12400.04 | 13332.18 | 0.104 | 0.107 | 6.9917 |
| 276 | 14678.8 | 15047.5 | 0.125 | 0.124 | 2.4502 | 17867.01 | 18082.36 | 0.111 | 0.11 | 1.191 |
| 277 | 12774.13 | 13647.26 | 0.125 | 0.118 | 6.3978 | 14088.49 | 14205.06 | 0.076 | 0.076 | 0.8206 |
| 278 | 13564.91 | 14472.88 | 0.125 | 0.12 | 6.2736 | 15413.74 | 15528.21 | 0.106 | 0.111 | 0.7372 |
| 279 | 13902.54 | 14804.66 | 0.125 | 0.119 | 6.0935 | 11891.77 | 12273.43 | 0.099 | 0.108 | 3.1097 |
| 280 | 14770.63 | 15938.1 | 0.125 | 0.119 | 7.325 | 12099.99 | 12444.59 | 0.102 | 0.111 | 2.769 |
| 281 | 12625.42 | 12743.2 | 0.125 | 0.125 | 0.9243 | 12783.93 | 12656.56 | 0.1 | 0.106 | -1.0063 |
| 282 | 14063.74 | 14181.52 | 0.125 | 0.125 | 0.8305 | 10638.06 | 10749.39 | 0.123 | 0.122 | 1.0357 |
| 283 | 13137.66 | 14442.08 | 0.125 | 0.126 | 9.0321 | 16207.98 | 17082.87 | 0.099 | 0.105 | 5.1214 |
| 284 | 13298.89 | 13376.67 | 0.125 | 0.124 | 0.5815 | 13993.91 | 13979.98 | 0.108 | 0.112 | -0.0997 |
| 285 | 16355.05 | 16522.54 | 0.125 | 0.124 | 1.0137 | 13093.52 | 14647.58 | 0.081 | 0.09 | 10.61 |
| 286 | 13469.23 | 14761.73 | 0.125 | 0.127 | 8.7557 | 12305.62 | 12642.31 | 0.093 | 0.096 | 2.6632 |
| 287 | 13243.74 | 13136.04 | 0.125 | 0.128 | -0.8198 | 11626.24 | 12774.02 | 0.075 | 0.091 | 8.9853 |
| 288 | 14218.11 | 14335.9 | 0.125 | 0.125 | 0.8216 | 11822.21 | 11714.93 | 0.126 | 0.121 | -0.9157 |
| 289 | 15618.09 | 15785.58 | 0.125 | 0.124 | 1.061 | 11527.9 | 11546.47 | 0.073 | 0.086 | 0.1609 |
| 290 | 12325.65 | 12443.43 | 0.125 | 0.125 | 0.9465 | 11659.01 | 11633.15 | 0.117 | 0.121 | -0.2223 |
| 291 | 14379.07 | 14649.57 | 0.125 | 0.125 | 1.8465 | 12422.96 | 12525 | 0.116 | 0.122 | 0.8147 |
| 292 | 12160.97 | 13096.4 | 0.125 | 0.125 | 7.1426 | 15889.05 | 16351.65 | 0.118 | 0.115 | 2.8291 |
| 293 | 13532.34 | 13650.12 | 0.125 | 0.125 | 0.8629 | 13195.11 | 13582.85 | 0.127 | 0.121 | 2.8547 |
| 294 | 13037.81 | 14115.58 | 0.125 | 0.12 | 7.6353 | 11084.59 | 11181.66 | 0.129 | 0.126 | 0.8681 |
| 295 | 13042.75 | 14031.02 | 0.125 | 0.12 | 7.0435 | 15055.81 | 14843.59 | 0.101 | 0.106 | -1.4297 |
| 296 | 13309.44 | 14593.44 | 0.125 | 0.12 | 8.7985 | 11102.71 | 11625.94 | 0.133 | 0.128 | 4.5005 |
| 297 | 13342.4 | 13460.18 | 0.125 | 0.125 | 0.875 | 12640.44 | 12952.7 | 0.108 | 0.112 | 2.4107 |
| 298 | 12246.79 | 13642.97 | 0.125 | 0.124 | 10.234 | 11111.86 | 12247.44 | 0.108 | 0.108 | 9.272 |

| 299 | 13358.76 | 13476.55 | 0.125 | 0.125 | 0.874 | 11450.05 | 11942.86 | 0.126 | 0.113 | 4.1264 |
| --- | --- | --- | --- | --- | --- | --- | --- | --- | --- | --- |
| 300 | 12782.61 | 13048.97 | 0.125 | 0.125 | 2.0412 | 11938.81 | 11913.87 | 0.083 | 0.083 | -0.2093 |
| 301 | 13585.18 | 13702.97 | 0.125 | 0.125 | 0.8595 | 14267.84 | 13798.56 | 0.098 | 0.079 | -3.401 |
| 302 | 11906.97 | 12341.01 | 0.125 | 0.126 | 3.5171 | 13675.57 | 14534.39 | 0.113 | 0.11 | 5.9089 |
| 303 | 13124.23 | 14379.95 | 0.125 | 0.126 | 8.7324 | 12040.82 | 12018.1 | 0.088 | 0.088 | -0.1891 |
| 304 | 12119.11 | 13555.25 | 0.125 | 0.126 | 10.595 | 15789.24 | 16285.05 | 0.092 | 0.106 | 3.0446 |
| 305 | 11778.69 | 12170.27 | 0.125 | 0.126 | 3.2175 | 12750.55 | 12852.6 | 0.116 | 0.122 | 0.794 |
| 306 | 13115.22 | 14105.83 | 0.125 | 0.119 | 7.0227 | 14903.44 | 14889.51 | 0.107 | 0.111 | -0.0936 |
| 307 | 13714.23 | 15055.64 | 0.125 | 0.12 | 8.9097 | 13455.97 | 13565.59 | 0.116 | 0.121 | 0.8081 |
| 308 | 13966.78 | 15264.22 | 0.125 | 0.121 | 8.4999 | 11678.8 | 12151.19 | 0.126 | 0.121 | 3.8876 |
| 309 | 12706.19 | 12565.06 | 0.125 | 0.128 | -1.1232 | 11030.91 | 11103.63 | 0.074 | 0.094 | 0.6549 |
| 310 | 12694.77 | 12812.55 | 0.126 | 0.125 | 0.9193 | 13927.91 | 13905.18 | 0.081 | 0.081 | -0.1634 |
| 311 | 12394.11 | 13294.68 | 0.126 | 0.125 | 6.7739 | 9992.813 | 10146.57 | 0.122 | 0.119 | 1.5154 |
| 312 | 13501.83 | 13139.89 | 0.126 | 0.102 | -2.7545 | 13347.84 | 13426.63 | 0.072 | 0.076 | 0.5868 |
| 313 | 11517.27 | 11966.84 | 0.126 | 0.126 | 3.7568 | 11087.13 | 12123.21 | 0.115 | 0.113 | 8.5463 |
| 314 | 11484.72 | 11971.86 | 0.126 | 0.125 | 4.0691 | 12307.63 | 12409.67 | 0.115 | 0.12 | 0.8223 |
| 315 | 12950.38 | 13683.89 | 0.126 | 0.127 | 5.3603 | 11060.54 | 11028.83 | 0.1 | 0.103 | -0.2876 |
| 316 | 13247.22 | 14524.15 | 0.126 | 0.12 | 8.7918 | 14084 | 14027.01 | 0.092 | 0.094 | -0.4063 |
| 317 | 14010.27 | 14128.05 | 0.126 | 0.125 | 0.8337 | 15799.63 | 15624.48 | 0.086 | 0.086 | -1.121 |
| 318 | 11928.28 | 12989.56 | 0.126 | 0.127 | 8.1703 | 12061.56 | 12165.2 | 0.079 | 0.083 | 0.8519 |
| 319 | 14353.5 | 14938.15 | 0.126 | 0.125 | 3.9138 | 14377.7 | 14512.76 | 0.077 | 0.079 | 0.9306 |
| 320 | 13981.81 | 14120.31 | 0.126 | 0.125 | 0.9808 | 11573.93 | 11719.7 | 0.127 | 0.13 | 1.2438 |
| 321 | 12711.59 | 13594 | 0.126 | 0.119 | 6.4912 | 11525.94 | 11598.36 | 0.078 | 0.083 | 0.6245 |
| 322 | 13720.27 | 13838.05 | 0.126 | 0.126 | 0.8511 | 12302.06 | 12514.7 | 0.079 | 0.077 | 1.6991 |
| 323 | 12514.97 | 13131.61 | 0.126 | 0.124 | 4.6958 | 11878.78 | 11980.82 | 0.11 | 0.115 | 0.8517 |
| 324 | 13760.57 | 15058 | 0.126 | 0.121 | 8.6163 | 11525.85 | 11904.34 | 0.073 | 0.077 | 3.1794 |
| 325 | 13064.71 | 13316.55 | 0.126 | 0.126 | 1.8912 | 10111.57 | 10193.91 | 0.12 | 0.116 | 0.8077 |
| 326 | 13803.67 | 15102.85 | 0.126 | 0.119 | 8.6023 | 12064.08 | 12654.93 | 0.109 | 0.115 | 4.6689 |
| 327 | 12745.14 | 13653.11 | 0.126 | 0.12 | 6.6503 | 12296.12 | 12633.14 | 0.104 | 0.112 | 2.6677 |
| 328 | 11267.09 | 11926.86 | 0.126 | 0.126 | 5.5319 | 11459.17 | 11554.61 | 0.128 | 0.129 | 0.826 |
| 329 | 12506.53 | 13584.3 | 0.126 | 0.12 | 7.9339 | 11149.3 | 11752.38 | 0.11 | 0.11 | 5.1316 |
| 330 | 12009.88 | 12127.66 | 0.126 | 0.126 | 0.9712 | 13232.49 | 14770.93 | 0.066 | 0.069 | 10.415 |
| 331 | 12192.33 | 13164.83 | 0.126 | 0.127 | 7.3871 | 11574.54 | 11597.3 | 0.11 | 0.119 | 0.1962 |
| 332 | 16473.21 | 16640.7 | 0.126 | 0.125 | 1.0065 | 10158.29 | 10269.63 | 0.124 | 0.122 | 1.0841 |
| 333 | 14263 | 14533.5 | 0.126 | 0.125 | 1.8612 | 12812.01 | 13076.15 | 0.081 | 0.093 | 2.02 |
| 334 | 12227.48 | 13305.25 | 0.126 | 0.12 | 8.1003 | 10308.65 | 10399.77 | 0.127 | 0.129 | 0.8762 |
| 335 | 12996.27 | 12980.27 | 0.126 | 0.097 | -0.1232 | 13538.64 | 13764.12 | 0.089 | 0.087 | 1.6382 |
| 336 | 14329.33 | 14496.81 | 0.126 | 0.125 | 1.1553 | 11041.88 | 11041.88 | 0.074 | 0.074 | 0 |
| 337 | 13752.32 | 14719.13 | 0.126 | 0.122 | 6.5684 | 11533.66 | 11533.66 | 0.076 | 0.076 | 0 |
| 338 | 11608.07 | 12035.3 | 0.126 | 0.127 | 3.5498 | 13820.18 | 13818.68 | 0.112 | 0.116 | -0.0109 |
| 339 | 13962.75 | 13299.27 | 0.126 | 0.103 | -4.9889 | 12431.24 | 12380.95 | 0.089 | 0.089 | -0.4062 |
| 340 | 13911.77 | 14791.46 | 0.126 | 0.121 | 5.9473 | 10933.28 | 11406.29 | 0.133 | 0.129 | 4.147 |
| 341 | 13200.81 | 13273.48 | 0.127 | 0.125 | 0.5475 | 18136.3 | 18075.08 | 0.098 | 0.101 | -0.3387 |
| 342 | 12739.23 | 12857.01 | 0.127 | 0.126 | 0.9161 | 12388.86 | 12397.15 | 0.096 | 0.093 | 0.0668 |
| 343 | 12476.47 | 13118.59 | 0.127 | 0.126 | 4.8947 | 16612.68 | 16490.46 | 0.1 | 0.104 | -0.7411 |
| 344 | 11983.63 | 12410.86 | 0.127 | 0.128 | 3.4424 | 12658.87 | 12607.16 | 0.086 | 0.086 | -0.4102 |
| 345 | 12383.14 | 12309.59 | 0.127 | 0.128 | -0.5975 | 13859.47 | 13836.75 | 0.087 | 0.087 | -0.1642 |
| 346 | 11314.84 | 11388.99 | 0.127 | 0.127 | 0.651 | 12271.8 | 13553.61 | 0.077 | 0.094 | 9.4573 |
| 347 | 14263.46 | 13390.98 | 0.127 | 0.103 | -6.5154 | 11076.73 | 11072.59 | 0.076 | 0.076 | -0.0374 |
| 348 | 14171.25 | 14272.46 | 0.127 | 0.126 | 0.7092 | 14257.05 | 14303.62 | 0.115 | 0.117 | 0.3256 |

| 349 | 12396.62 | 13281.25 | 0.127 | 0.127 | 6.6607 | 13375.27 | 13385.86 | 0.084 | 0.093 | 0.0791 |
| --- | --- | --- | --- | --- | --- | --- | --- | --- | --- | --- |
| 350 | 13309.12 | 13426.9 | 0.127 | 0.127 | 0.8772 | 12168.54 | 12647.71 | 0.119 | 0.113 | 3.7886 |
| 351 | 13263.26 | 13341.04 | 0.127 | 0.126 | 0.583 | 12332.29 | 12323.76 | 0.117 | 0.119 | -0.0692 |
| 352 | 13237.51 | 13555.63 | 0.127 | 0.131 | 2.3467 | 10751.68 | 11238.46 | 0.126 | 0.116 | 4.3313 |
| 353 | 14213.76 | 14381.25 | 0.127 | 0.126 | 1.1646 | 12363.02 | 12357.07 | 0.082 | 0.084 | -0.0481 |
| 354 | 16685.63 | 17292.72 | 0.127 | 0.117 | 3.5107 | 15705.28 | 18074.35 | 0.064 | 0.102 | 13.107 |
| 355 | 12766.5 | 13668.62 | 0.127 | 0.121 | 6.5999 | 10192.87 | 10294.92 | 0.1 | 0.107 | 0.9912 |
| 356 | 12261.26 | 13143.67 | 0.127 | 0.12 | 6.7136 | 13559.56 | 13559.56 | 0.069 | 0.069 | 0 |
| 357 | 12700.76 | 13164.27 | 0.127 | 0.127 | 3.5209 | 11564.58 | 11556.29 | 0.092 | 0.089 | -0.0717 |
| 358 | 14860.21 | 16027.68 | 0.127 | 0.121 | 7.2841 | 17706.42 | 17968.64 | 0.11 | 0.11 | 1.4593 |
| 359 | 14391.82 | 15803.83 | 0.127 | 0.128 | 8.9346 | 12730.35 | 13042.6 | 0.115 | 0.121 | 2.3941 |
| 360 | 13539.08 | 13951.75 | 0.127 | 0.13 | 2.9578 | 10960.45 | 11271.61 | 0.123 | 0.121 | 2.7606 |
| 361 | 12704.58 | 13582.55 | 0.127 | 0.121 | 6.464 | 14030.25 | 14183.26 | 0.084 | 0.093 | 1.0788 |
| 362 | 12556.56 | 13080.98 | 0.127 | 0.127 | 4.009 | 13418.96 | 13383.1 | 0.09 | 0.086 | -0.2679 |
| 363 | 12286.8 | 12404.59 | 0.127 | 0.127 | 0.9495 | 17553.45 | 17329.31 | 0.098 | 0.103 | -1.2934 |
| 364 | 13000.47 | 14307.23 | 0.127 | 0.121 | 9.1336 | 15733.43 | 16885.94 | 0.118 | 0.112 | 6.8253 |
| 365 | 11589.17 | 11706.95 | 0.128 | 0.127 | 1.0061 | 12848.96 | 12811.89 | 0.117 | 0.12 | -0.2893 |
| 366 | 13339.61 | 14416.29 | 0.128 | 0.121 | 7.4685 | 15825.31 | 15902.5 | 0.103 | 0.108 | 0.4854 |
| 367 | 14002.66 | 14526.2 | 0.128 | 0.117 | 3.6041 | 12621.9 | 12592.49 | 0.081 | 0.088 | -0.2336 |
| 368 | 12338.23 | 12456.01 | 0.128 | 0.127 | 0.9456 | 11107.98 | 11665.85 | 0.127 | 0.124 | 4.7821 |
| 369 | 13438.69 | 13853.11 | 0.128 | 0.105 | 2.9915 | 17782.19 | 18053.2 | 0.109 | 0.11 | 1.5011 |
| 370 | 12298.73 | 12416.51 | 0.128 | 0.127 | 0.9486 | 11393.95 | 11521.94 | 0.092 | 0.09 | 1.1108 |
| 371 | 13346.98 | 13585 | 0.128 | 0.131 | 1.7521 | 12397.81 | 12499.11 | 0.074 | 0.086 | 0.8105 |
| 372 | 12356.14 | 15486.58 | 0.128 | 0.13 | 20.214 | 11961.68 | 12298.79 | 0.11 | 0.115 | 2.741 |
| 373 | 13290.92 | 13737.7 | 0.128 | 0.126 | 3.2522 | 14364.77 | 14377.4 | 0.106 | 0.109 | 0.0879 |
| 374 | 13275.68 | 14503.95 | 0.128 | 0.13 | 8.4685 | 16748 | 17186.95 | 0.095 | 0.102 | 2.5539 |
| 375 | 15314.97 | 17207.45 | 0.128 | 0.129 | 10.998 | 11947.28 | 12109.5 | 0.123 | 0.123 | 1.3396 |
| 376 | 13723.42 | 14549.27 | 0.128 | 0.122 | 5.6762 | 13991.4 | 14136.13 | 0.087 | 0.095 | 1.0238 |
| 377 | 12334.65 | 12452.43 | 0.128 | 0.128 | 0.9459 | 9562.519 | 9666.072 | 0.132 | 0.134 | 1.0713 |
| 378 | 12480.44 | 12598.23 | 0.128 | 0.128 | 0.9349 | 13154.88 | 13967.51 | 0.117 | 0.123 | 5.818 |
| 379 | 14348.4 | 13082.66 | 0.128 | 0.102 | -9.6749 | 12351.6 | 12312.1 | 0.071 | 0.071 | -0.3208 |
| 380 | 12392.22 | 12510 | 0.128 | 0.127 | 0.9415 | 12927.06 | 13029.11 | 0.115 | 0.121 | 0.7832 |
| 381 | 12573.99 | 13193.68 | 0.128 | 0.127 | 4.6969 | 12264.64 | 12250.7 | 0.096 | 0.101 | -0.1137 |
| 382 | 13167.34 | 13285.12 | 0.128 | 0.128 | 0.8866 | 13050.41 | 13044.55 | 0.1 | 0.102 | -0.0449 |
| 383 | 12433.15 | 12696.15 | 0.128 | 0.092 | 2.0715 | 17099.8 | 17434.62 | 0.111 | 0.109 | 1.9204 |
| 384 | 11789.82 | 11907.6 | 0.128 | 0.128 | 0.9891 | 12613.04 | 12890.24 | 0.075 | 0.082 | 2.1504 |
| 385 | 11699.07 | 12570.06 | 0.128 | 0.128 | 6.9291 | 15610.56 | 15590.11 | 0.108 | 0.112 | -0.1312 |
| 386 | 13714.41 | 13881.9 | 0.128 | 0.127 | 1.2065 | 11906.83 | 12008.87 | 0.116 | 0.121 | 0.8498 |
| 387 | 13725.81 | 14502.25 | 0.128 | 0.12 | 5.3539 | 14125.6 | 14045.31 | 0.087 | 0.087 | -0.5717 |
| 388 | 12205.22 | 15198 | 0.128 | 0.131 | 19.692 | 16777.18 | 17220.32 | 0.105 | 0.11 | 2.5733 |
| 389 | 12332.42 | 13057.55 | 0.128 | 0.127 | 5.5534 | 16152.06 | 16573.31 | 0.101 | 0.116 | 2.5417 |
| 390 | 15084.76 | 14085.78 | 0.128 | 0.102 | -7.0921 | 12648.21 | 13758.2 | 0.092 | 0.079 | 8.0678 |
| 391 | 14141.86 | 16778.01 | 0.129 | 0.126 | 15.712 | 14952.5 | 15084.51 | 0.118 | 0.119 | 0.8751 |
| 392 | 13290.7 | 14328.59 | 0.129 | 0.122 | 7.2435 | 14964.22 | 15124.51 | 0.118 | 0.119 | 1.0598 |
| 393 | 11839.07 | 12357.43 | 0.129 | 0.128 | 4.1947 | 11919.99 | 11937.06 | 0.093 | 0.093 | 0.143 |
| 394 | 14588.13 | 16480.61 | 0.129 | 0.13 | 11.483 | 12193.85 | 12350 | 0.125 | 0.126 | 1.2644 |
| 395 | 12658.21 | 13377.82 | 0.129 | 0.091 | 5.3791 | 11885.61 | 11908.03 | 0.095 | 0.093 | 0.1883 |
| 396 | 13165.31 | 14047.72 | 0.129 | 0.122 | 6.2815 | 10444.19 | 10999.26 | 0.123 | 0.126 | 5.0464 |
| 397 | 12388.04 | 13685.48 | 0.129 | 0.124 | 9.4804 | 18807.09 | 18374.14 | 0.102 | 0.092 | -2.3563 |
| 398 | 16621.05 | 17228.99 | 0.129 | 0.127 | 3.5286 | 11526.57 | 11725.69 | 0.102 | 0.108 | 1.6981 |

| 399 | 12494.29 | 12612.07 | 0.129 | 0.128 | 0.9339 | 13160.18 | 12790.98 | 0.082 | 0.086 | -2.8864 |
| --- | --- | --- | --- | --- | --- | --- | --- | --- | --- | --- |
| 400 | 14820.59 | 16194.84 | 0.129 | 0.129 | 8.4857 | 15840.64 | 15842.06 | 0.105 | 0.108 | 0.009 |
| 401 | 15139.02 | 16744.69 | 0.129 | 0.13 | 9.5891 | 11648.36 | 12616.77 | 0.078 | 0.076 | 7.6755 |
| 402 | 13963.78 | 15668.81 | 0.129 | 0.128 | 10.882 | 11971.2 | 11607.15 | 0.128 | 0.122 | -3.1365 |
| 403 | 11989.32 | 12107.1 | 0.129 | 0.128 | 0.9728 | 12660.77 | 12319.94 | 0.083 | 0.091 | -2.7665 |
| 404 | 16910.86 | 14613.24 | 0.129 | 0.105 | -15.723 | 11928.73 | 12358.15 | 0.11 | 0.116 | 3.4748 |
| 405 | 12669.93 | 15623.71 | 0.129 | 0.131 | 18.906 | 16339.28 | 16333.04 | 0.104 | 0.108 | -0.0382 |
| 406 | 13626.68 | 17271.8 | 0.129 | 0.128 | 21.104 | 11191.2 | 11408.48 | 0.072 | 0.088 | 1.9045 |
| 407 | 13427.03 | 15296.08 | 0.129 | 0.129 | 12.219 | 10478.03 | 10473.89 | 0.076 | 0.076 | -0.0395 |
| 408 | 12751.03 | 13615.92 | 0.129 | 0.131 | 6.352 | 15740.34 | 15612.56 | 0.085 | 0.086 | -0.8185 |
| 409 | 14243.46 | 13921.52 | 0.129 | 0.106 | -2.3125 | 11545.88 | 12598.36 | 0.114 | 0.117 | 8.3541 |
| 410 | 16011.51 | 16618.6 | 0.129 | 0.119 | 3.6531 | 10495.13 | 10640.9 | 0.127 | 0.13 | 1.3699 |
| 411 | 15057.87 | 16692.33 | 0.129 | 0.13 | 9.7917 | 10955.29 | 11005.79 | 0.09 | 0.098 | 0.4589 |
| 412 | 12495.94 | 12504.59 | 0.129 | 0.093 | 0.0692 | 12557.07 | 12534.35 | 0.087 | 0.088 | -0.1813 |
| 413 | 14148.63 | 14683.42 | 0.129 | 0.122 | 3.6421 | 13650.65 | 13752.7 | 0.11 | 0.115 | 0.742 |
| 414 | 13033.54 | 14201.02 | 0.129 | 0.122 | 8.221 | 10516.01 | 10527.05 | 0.108 | 0.118 | 0.1049 |
| 415 | 12058.03 | 14724.95 | 0.129 | 0.132 | 18.112 | 11035.81 | 11348.86 | 0.117 | 0.122 | 2.7584 |
| 416 | 14128.93 | 14838.57 | 0.129 | 0.124 | 4.7824 | 14596.16 | 15131.47 | 0.103 | 0.115 | 3.5377 |
| 417 | 13322.67 | 14823.76 | 0.129 | 0.129 | 10.126 | 13884.62 | 13466.46 | 0.082 | 0.087 | -3.1052 |
| 418 | 15497.47 | 16507.47 | 0.129 | 0.128 | 6.1184 | 15892.65 | 15886.41 | 0.105 | 0.11 | -0.0393 |
| 419 | 15345.82 | 16989.43 | 0.129 | 0.132 | 9.6743 | 14461.79 | 14614.8 | 0.082 | 0.091 | 1.047 |
| 420 | 12410.41 | 12735.21 | 0.13 | 0.133 | 2.5504 | 15637.9 | 15785.44 | 0.109 | 0.11 | 0.9346 |
| 421 | 12461.4 | 13236.62 | 0.13 | 0.122 | 5.8566 | 16153.48 | 16035.4 | 0.1 | 0.104 | -0.7363 |
| 422 | 12394.69 | 13101.12 | 0.13 | 0.122 | 5.3921 | 12125.37 | 12663.11 | 0.126 | 0.12 | 4.2466 |
| 423 | 12688.39 | 13612.72 | 0.13 | 0.121 | 6.7902 | 17120.61 | 17429.85 | 0.103 | 0.107 | 1.7742 |
| 424 | 16295.67 | 17111.27 | 0.13 | 0.129 | 4.7664 | 12427.27 | 12456.27 | 0.091 | 0.089 | 0.2328 |
| 425 | 15656.51 | 16107.33 | 0.13 | 0.121 | 2.7988 | 11533.31 | 11386.53 | 0.114 | 0.114 | -1.289 |
| 426 | 11998.39 | 12880.5 | 0.13 | 0.123 | 6.8485 | 10813.77 | 11445.66 | 0.128 | 0.13 | 5.5208 |
| 427 | 12742.74 | 13027.99 | 0.13 | 0.099 | 2.1895 | 11835.05 | 11850.11 | 0.091 | 0.092 | 0.1271 |
| 428 | 13588.45 | 13835.04 | 0.13 | 0.123 | 1.7824 | 17737.64 | 18332.67 | 0.111 | 0.107 | 3.2457 |
| 429 | 15414.76 | 17184.14 | 0.13 | 0.132 | 10.297 | 11897.21 | 11862.56 | 0.11 | 0.115 | -0.2921 |
| 430 | 13574.17 | 14741.64 | 0.13 | 0.123 | 7.9196 | 11447.95 | 11789.61 | 0.106 | 0.115 | 2.898 |
| 431 | 14490.76 | 15246.85 | 0.13 | 0.12 | 4.959 | 14582.17 | 15257.52 | 0.109 | 0.114 | 4.4264 |
| 432 | 15605.47 | 14457.17 | 0.13 | 0.1 | -7.9428 | 15069.24 | 15002.38 | 0.086 | 0.087 | -0.4457 |
| 433 | 13388.33 | 14247.6 | 0.13 | 0.122 | 6.031 | 13674.96 | 13472.59 | 0.115 | 0.113 | -1.5021 |
| 434 | 12788.6 | 13691.51 | 0.13 | 0.123 | 6.5947 | 13507.93 | 13483.79 | 0.083 | 0.08 | -0.179 |
| 435 | 15316.95 | 15924.04 | 0.13 | 0.12 | 3.8124 | 11834.76 | 11954.55 | 0.082 | 0.091 | 1.0021 |
| 436 | 14710.55 | 16591.82 | 0.13 | 0.131 | 11.339 | 12520.65 | 12497.93 | 0.085 | 0.085 | -0.1818 |
| 437 | 12565.87 | 15308.57 | 0.13 | 0.132 | 17.916 | 11851.59 | 12312.42 | 0.08 | 0.09 | 3.7428 |
| 438 | 13279.99 | 14654.24 | 0.13 | 0.131 | 9.3778 | 11194.55 | 11657.1 | 0.069 | 0.079 | 3.968 |
| 439 | 18443.85 | 17326.13 | 0.13 | 0.109 | -6.451 | 14908.44 | 15048.65 | 0.109 | 0.109 | 0.9317 |
| 440 | 14374.78 | 16089.52 | 0.131 | 0.132 | 10.657 | 17060.98 | 17318.85 | 0.107 | 0.114 | 1.4889 |
| 441 | 12767.44 | 13468.1 | 0.131 | 0.122 | 5.2024 | 12177.86 | 11779.73 | 0.13 | 0.129 | -3.3798 |
| 442 | 17815.92 | 14386.67 | 0.131 | 0.107 | -23.836 | 12260.7 | 12602.37 | 0.107 | 0.115 | 2.7111 |
| 443 | 16333.67 | 14132.14 | 0.131 | 0.106 | -15.578 | 10947.21 | 11571.35 | 0.126 | 0.122 | 5.3939 |
| 444 | 14712.36 | 16183.54 | 0.131 | 0.134 | 9.0906 | 11627.33 | 11604.61 | 0.085 | 0.086 | -0.1958 |
| 445 | 14427.21 | 15147.77 | 0.131 | 0.122 | 4.7569 | 14569.98 | 14582.61 | 0.107 | 0.11 | 0.0866 |
| 446 | 16885.44 | 14633.68 | 0.131 | 0.107 | -15.388 | 11423.89 | 11318.95 | 0.1 | 0.108 | -0.9271 |
| 447 | 13206.44 | 14937.46 | 0.131 | 0.132 | 11.588 | 12435.32 | 12688.18 | 0.118 | 0.117 | 1.9929 |
| 448 | 17939 | 15840.91 | 0.131 | 0.107 | -13.245 | 15520.72 | 15527.5 | 0.103 | 0.107 | 0.0436 |

| 449 | 15871.18 | 14134.63 | 0.131 | 0.104 | -12.286 | 11126.95 | 11330.68 | 0.128 | 0.129 | 1.798 |
| --- | --- | --- | --- | --- | --- | --- | --- | --- | --- | --- |
| 450 | 17127.37 | 14588.82 | 0.131 | 0.111 | -17.401 | 10527.1 | 11015.9 | 0.109 | 0.112 | 4.4372 |
| 451 | 17717.83 | 17013.67 | 0.131 | 0.108 | -4.1388 | 12812.39 | 12804.11 | 0.089 | 0.086 | -0.0647 |
| 452 | 12189.82 | 13482.61 | 0.131 | 0.125 | 9.5886 | 10437.43 | 11131.52 | 0.114 | 0.114 | 6.2354 |
| 453 | 12891.23 | 14387.91 | 0.131 | 0.132 | 10.402 | 16283.53 | 16889.56 | 0.112 | 0.113 | 3.5882 |
| 454 | 15114.84 | 16728.16 | 0.131 | 0.134 | 9.6443 | 13323.63 | 13209.49 | 0.102 | 0.1 | -0.8641 |
| 455 | 16469.52 | 17159.73 | 0.131 | 0.127 | 4.0223 | 11109.62 | 11669.59 | 0.13 | 0.123 | 4.7985 |
| 456 | 12199.01 | 15191.21 | 0.131 | 0.129 | 19.697 | 12128.45 | 12234.93 | 0.095 | 0.102 | 0.8703 |
| 457 | 12924.87 | 15718.98 | 0.131 | 0.132 | 17.775 | 13874.47 | 15340.75 | 0.071 | 0.081 | 9.558 |
| 458 | 14921.89 | 14733.09 | 0.131 | 0.122 | -1.2815 | 14882.88 | 14768.74 | 0.11 | 0.108 | -0.7729 |
| 459 | 13079.93 | 15122.51 | 0.131 | 0.133 | 13.507 | 16239.28 | 16251.91 | 0.101 | 0.104 | 0.0777 |
| 460 | 12761.82 | 15766.69 | 0.131 | 0.131 | 19.058 | 12391.73 | 12938.76 | 0.127 | 0.122 | 4.2279 |
| 461 | 13016.72 | 14448.1 | 0.131 | 0.134 | 9.9071 | 12661.65 | 12449.43 | 0.096 | 0.102 | -1.7046 |
| 462 | 12168.19 | 13867.06 | 0.131 | 0.132 | 12.251 | 13542.34 | 13479.62 | 0.088 | 0.088 | -0.4653 |
| 463 | 11314.77 | 13932.91 | 0.131 | 0.134 | 18.791 | 15186.9 | 15700.58 | 0.106 | 0.109 | 3.2717 |
| 464 | 13567.36 | 13277.34 | 0.131 | 0.099 | -2.1843 | 11271.86 | 11413.37 | 0.124 | 0.124 | 1.2398 |
| 465 | 13907.72 | 15612.74 | 0.131 | 0.13 | 10.921 | 13107.06 | 13246.89 | 0.115 | 0.12 | 1.0556 |
| 466 | 11959.48 | 13764.47 | 0.131 | 0.135 | 13.113 | 12782.37 | 12768.43 | 0.113 | 0.117 | -0.1091 |
| 467 | 12760.74 | 13372.58 | 0.131 | 0.134 | 4.5753 | 12163.67 | 12100.95 | 0.088 | 0.089 | -0.5183 |
| 468 | 17168.28 | 17010.87 | 0.131 | 0.108 | -0.9254 | 16301.37 | 16381.49 | 0.103 | 0.108 | 0.4891 |
| 469 | 13002.7 | 14269.97 | 0.131 | 0.133 | 8.8806 | 11730.53 | 11741.12 | 0.087 | 0.097 | 0.0902 |
| 470 | 12685.08 | 14329.12 | 0.131 | 0.133 | 11.473 | 10786.97 | 10932.74 | 0.127 | 0.13 | 1.3333 |
| 471 | 15164.67 | 15771.76 | 0.131 | 0.12 | 3.8492 | 12780.38 | 12882.43 | 0.116 | 0.121 | 0.7921 |
| 472 | 14671.2 | 16400.38 | 0.131 | 0.132 | 10.543 | 11345.14 | 11499.19 | 0.086 | 0.082 | 1.3397 |
| 473 | 13134.39 | 12456.51 | 0.131 | 0.126 | -5.442 | 10812.5 | 11600.37 | 0.117 | 0.114 | 6.7917 |
| 474 | 15282.12 | 17304.35 | 0.131 | 0.132 | 11.686 | 12540.62 | 12661.33 | 0.095 | 0.094 | 0.9534 |
| 475 | 15432.39 | 13788.62 | 0.131 | 0.1 | -11.921 | 14224.88 | 14818.69 | 0.118 | 0.116 | 4.0072 |
| 476 | 12177.23 | 15135.78 | 0.131 | 0.134 | 19.547 | 13916.38 | 14374.13 | 0.119 | 0.115 | 3.1845 |
| 477 | 12579.12 | 13333.63 | 0.131 | 0.123 | 5.6587 | 17822.39 | 17783.6 | 0.095 | 0.099 | -0.2181 |
| 478 | 15848.61 | 14900.64 | 0.131 | 0.103 | -6.3619 | 12024.4 | 11949.26 | 0.095 | 0.095 | -0.6289 |
| 479 | 16610.53 | 14009.6 | 0.131 | 0.105 | -18.565 | 12468.02 | 12496.8 | 0.082 | 0.084 | 0.2304 |
| 480 | 13276.97 | 14806.64 | 0.131 | 0.129 | 10.331 | 14347.67 | 14054.95 | 0.084 | 0.088 | -2.0827 |
| 481 | 12577.49 | 13460.41 | 0.131 | 0.124 | 6.5593 | 17109.31 | 16987.09 | 0.099 | 0.103 | -0.7195 |
| 482 | 14952.77 | 13474.47 | 0.131 | 0.099 | -10.971 | 13840.45 | 14492.38 | 0.089 | 0.084 | 4.4984 |
| 483 | 14623.28 | 16529.9 | 0.131 | 0.132 | 11.534 | 10972.48 | 11089.64 | 0.086 | 0.094 | 1.0565 |
| 484 | 16056.08 | 16819.04 | 0.131 | 0.129 | 4.5363 | 12612.48 | 12883.61 | 0.088 | 0.09 | 2.1044 |
| 485 | 12503.93 | 12733.67 | 0.131 | 0.135 | 1.8042 | 12292.33 | 12324.26 | 0.087 | 0.087 | 0.259 |
| 486 | 14072.27 | 14868.36 | 0.132 | 0.121 | 5.3542 | 11668.52 | 11659.82 | 0.086 | 0.094 | -0.0746 |
| 487 | 11878.28 | 12433.16 | 0.132 | 0.137 | 4.463 | 12548.64 | 12525.92 | 0.091 | 0.091 | -0.1814 |
| 488 | 19063.64 | 18362.83 | 0.132 | 0.107 | -3.8165 | 11229.93 | 11289.13 | 0.083 | 0.081 | 0.5244 |
| 489 | 13906.79 | 14417.23 | 0.132 | 0.12 | 3.5405 | 16633.28 | 16915.79 | 0.108 | 0.115 | 1.6701 |
| 490 | 11770.49 | 13945.26 | 0.132 | 0.132 | 15.595 | 11630.41 | 12221.41 | 0.123 | 0.119 | 4.8358 |
| 491 | 14121.06 | 14295.11 | 0.132 | 0.12 | 1.2176 | 11432.68 | 12369.66 | 0.069 | 0.07 | 7.5749 |
| 492 | 12821.53 | 13453.87 | 0.132 | 0.135 | 4.7001 | 12438.52 | 12540.57 | 0.117 | 0.122 | 0.8137 |
| 493 | 12213.45 | 14643.56 | 0.132 | 0.132 | 16.595 | 10434.8 | 10918.32 | 0.132 | 0.129 | 4.4285 |
| 494 | 13670.35 | 13439.41 | 0.132 | 0.099 | -1.7183 | 12213.97 | 12196.99 | 0.079 | 0.086 | -0.1393 |
| 495 | 11968.18 | 13335.44 | 0.132 | 0.125 | 10.253 | 13724.27 | 14266.52 | 0.104 | 0.108 | 3.8009 |
| 496 | 17511.24 | 18500.3 | 0.132 | 0.126 | 5.3462 | 14854.43 | 15308.54 | 0.114 | 0.112 | 2.9664 |
| 497 | 16031.82 | 16621.82 | 0.132 | 0.13 | 3.5496 | 11575.82 | 12063.19 | 0.128 | 0.124 | 4.0401 |
| 498 | 14795.64 | 15812.71 | 0.132 | 0.13 | 6.432 | 10478.5 | 10441.43 | 0.103 | 0.107 | -0.355 |

| 499 | 11770.9 | 12664.14 | 0.132 | 0.099 | 7.0533 | 11120.84 | 11115.4 | 0.121 | 0.12 | -0.049 |
| --- | --- | --- | --- | --- | --- | --- | --- | --- | --- | --- |
| 500 | 13351 | 14311.73 | 0.132 | 0.121 | 6.7129 | 12840.86 | 13301.69 | 0.07 | 0.079 | 3.4645 |
| 501 | 11932.17 | 12919.94 | 0.132 | 0.135 | 7.6453 | 15292.61 | 15220.72 | 0.107 | 0.11 | -0.4723 |
| 502 | 12527.26 | 13092.84 | 0.132 | 0.126 | 4.3198 | 10649.43 | 10645.29 | 0.078 | 0.078 | -0.0389 |
| 503 | 13330.4 | 12816.07 | 0.132 | 0.125 | -4.0132 | 15766.47 | 15969.4 | 0.115 | 0.115 | 1.2707 |
| 504 | 12563.7 | 13683.29 | 0.132 | 0.132 | 8.1821 | 12808.99 | 11854.07 | 0.106 | 0.106 | -8.0556 |
| 505 | 13394.99 | 16337.68 | 0.132 | 0.133 | 18.012 | 14003.56 | 13993.47 | 0.104 | 0.111 | -0.0721 |
| 506 | 11795.96 | 14600.58 | 0.132 | 0.133 | 19.209 | 10846.5 | 10957.63 | 0.085 | 0.083 | 1.0142 |
| 507 | 16082.45 | 13921.1 | 0.132 | 0.1 | -15.526 | 13834.35 | 13816.28 | 0.11 | 0.114 | -0.1308 |
| 508 | 17141.39 | 15536.76 | 0.132 | 0.108 | -10.328 | 11627.75 | 12529.67 | 0.1 | 0.092 | 7.1983 |
| 509 | 13934.64 | 14664.15 | 0.132 | 0.121 | 4.9748 | 13352.55 | 13497.28 | 0.085 | 0.093 | 1.0723 |
| 510 | 13017.56 | 12749.02 | 0.132 | 0.105 | -2.1064 | 11499.26 | 11813.23 | 0.107 | 0.112 | 2.6578 |
| 511 | 13259.66 | 14728.24 | 0.132 | 0.132 | 9.9712 | 11446.77 | 11388.19 | 0.091 | 0.092 | -0.5144 |
| 512 | 12504.45 | 13505.36 | 0.132 | 0.135 | 7.4112 | 12702.66 | 12804.71 | 0.118 | 0.123 | 0.7969 |
| 513 | 14897.92 | 15928.42 | 0.132 | 0.132 | 6.4696 | 12188.31 | 12649.14 | 0.07 | 0.079 | 3.6432 |
| 514 | 13609.02 | 16485.88 | 0.132 | 0.127 | 17.45 | 13845.77 | 13855.27 | 0.089 | 0.089 | 0.0685 |
| 515 | 17729.08 | 15511.79 | 0.132 | 0.107 | -14.294 | 14421.9 | 15192.14 | 0.065 | 0.068 | 5.07 |
| 516 | 17349 | 14620.96 | 0.132 | 0.109 | -18.658 | 11137.58 | 11398.03 | 0.122 | 0.121 | 2.2851 |
| 517 | 17406.3 | 15717.71 | 0.132 | 0.108 | -10.743 | 12553.87 | 12553.87 | 0.068 | 0.068 | 0 |
| 518 | 12669.51 | 16038.06 | 0.132 | 0.131 | 21.003 | 11489.91 | 12162.63 | 0.108 | 0.112 | 5.531 |
| 519 | 16027.27 | 16538.6 | 0.132 | 0.127 | 3.0918 | 13537.34 | 13514.62 | 0.086 | 0.087 | -0.1681 |
| 520 | 13581.38 | 14074.24 | 0.132 | 0.12 | 3.5019 | 16687.29 | 16764.48 | 0.102 | 0.107 | 0.4605 |
| 521 | 12522.73 | 13759.78 | 0.132 | 0.133 | 8.9904 | 11130 | 10733.73 | 0.108 | 0.109 | -3.6919 |
| 522 | 13055.2 | 14396.52 | 0.132 | 0.133 | 9.317 | 13803.05 | 13740.33 | 0.089 | 0.09 | -0.4565 |
| 523 | 11971.08 | 13513.7 | 0.132 | 0.135 | 11.415 | 9671.905 | 10014 | 0.13 | 0.122 | 3.4162 |
| 524 | 14586 | 13158.49 | 0.132 | 0.099 | -10.849 | 16089.91 | 16632.8 | 0.121 | 0.117 | 3.264 |
| 525 | 12180.87 | 12414.46 | 0.132 | 0.136 | 1.8816 | 14865.01 | 14701.08 | 0.084 | 0.085 | -1.1151 |
| 526 | 16488.81 | 16964.29 | 0.132 | 0.127 | 2.8028 | 13273.46 | 13375.5 | 0.107 | 0.113 | 0.7629 |
| 527 | 12746.2 | 14918.2 | 0.132 | 0.133 | 14.559 | 13379.7 | 13257.27 | 0.102 | 0.1 | -0.9235 |
| 528 | 15286.06 | 14039.18 | 0.132 | 0.101 | -8.8814 | 10263.25 | 10607.55 | 0.122 | 0.121 | 3.2458 |
| 529 | 13737.04 | 15852.34 | 0.132 | 0.131 | 13.344 | 10279.59 | 10273.52 | 0.128 | 0.126 | -0.059 |
| 530 | 12370.73 | 13513.14 | 0.132 | 0.135 | 8.4541 | 12863.81 | 12849.88 | 0.113 | 0.117 | -0.1084 |
| 531 | 17744.08 | 16895.33 | 0.132 | 0.112 | -5.0236 | 15862.32 | 16449.13 | 0.106 | 0.109 | 3.5674 |
| 532 | 16303.75 | 17269.73 | 0.132 | 0.13 | 5.5935 | 12986.78 | 12935.27 | 0.086 | 0.086 | -0.3982 |
| 533 | 13620.31 | 14674.39 | 0.132 | 0.133 | 7.1831 | 11002.23 | 11383.9 | 0.101 | 0.111 | 3.3527 |
| 534 | 13787.5 | 14303.8 | 0.132 | 0.121 | 3.6095 | 11616.64 | 11829.6 | 0.109 | 0.109 | 1.8003 |
| 535 | 18306.42 | 16833.86 | 0.132 | 0.107 | -8.7476 | 12721.93 | 13126.36 | 0.13 | 0.129 | 3.0811 |
| 536 | 14376.73 | 15211.49 | 0.132 | 0.129 | 5.4877 | 13900.86 | 14002.91 | 0.108 | 0.113 | 0.7287 |
| 537 | 15245.41 | 13559.53 | 0.132 | 0.1 | -12.433 | 11082.32 | 11269.51 | 0.123 | 0.122 | 1.6611 |
| 538 | 11404.09 | 12295.29 | 0.132 | 0.125 | 7.2483 | 12930.63 | 12589.79 | 0.083 | 0.09 | -2.7072 |
| 539 | 16374.61 | 15135.64 | 0.133 | 0.113 | -8.1858 | 11854.52 | 12216.28 | 0.107 | 0.111 | 2.9612 |
| 540 | 13148.18 | 14202.26 | 0.133 | 0.133 | 7.4219 | 11427.95 | 11156.32 | 0.13 | 0.123 | -2.4348 |
| 541 | 12792.8 | 12885.64 | 0.133 | 0.12 | 0.7205 | 15788.38 | 15865.58 | 0.099 | 0.104 | 0.4865 |
| 542 | 15279.53 | 15569.15 | 0.133 | 0.13 | 1.8602 | 11706.47 | 12129.65 | 0.108 | 0.112 | 3.4887 |
| 543 | 12936.72 | 13314.33 | 0.133 | 0.132 | 2.8361 | 12027.45 | 12052.72 | 0.083 | 0.085 | 0.2097 |
| 544 | 12049.1 | 13209.29 | 0.133 | 0.136 | 8.7832 | 15196.06 | 15615.4 | 0.11 | 0.116 | 2.6854 |
| 545 | 12756.44 | 15843.7 | 0.133 | 0.13 | 19.486 | 11389.84 | 11832.72 | 0.112 | 0.126 | 3.7428 |
| 546 | 16039.19 | 15270.22 | 0.133 | 0.113 | -5.0358 | 15651.17 | 15809.25 | 0.116 | 0.116 | 0.9999 |
| 547 | 15755.72 | 16275.67 | 0.133 | 0.131 | 3.1946 | 11745.24 | 12249.31 | 0.109 | 0.115 | 4.1151 |
| 548 | 14678.36 | 14342.56 | 0.133 | 0.096 | -2.3413 | 11257.11 | 11268.15 | 0.103 | 0.113 | 0.098 |

| 549 | 14474.55 | 14903.15 | 0.133 | 0.122 | 2.8759 | 11556.73 | 11576.23 | 0.093 | 0.093 | 0.1684 |
| --- | --- | --- | --- | --- | --- | --- | --- | --- | --- | --- |
| 550 | 10998.58 | 11753.09 | 0.133 | 0.123 | 6.4197 | 12077.9 | 12195.68 | 0.089 | 0.094 | 0.9658 |
| 551 | 12762.17 | 14174.18 | 0.133 | 0.132 | 9.9618 | 11966.33 | 11929.26 | 0.118 | 0.121 | -0.3108 |
| 552 | 12346.47 | 13503.95 | 0.133 | 0.125 | 8.5714 | 14003.14 | 14105.18 | 0.112 | 0.117 | 0.7235 |
| 553 | 16706.47 | 14778.21 | 0.133 | 0.108 | -13.048 | 13818.35 | 14354.95 | 0.104 | 0.108 | 3.7381 |
| 554 | 17225.3 | 14876.58 | 0.133 | 0.103 | -15.788 | 11743.34 | 11782.75 | 0.09 | 0.091 | 0.3345 |
| 555 | 15855.14 | 14734.45 | 0.133 | 0.102 | -7.6059 | 14682.59 | 14551.58 | 0.084 | 0.085 | -0.9003 |
| 556 | 12414.38 | 13640.11 | 0.133 | 0.132 | 8.9863 | 13933.14 | 14690.96 | 0.113 | 0.11 | 5.1584 |
| 557 | 11791.23 | 11861.27 | 0.133 | 0.136 | 0.5905 | 11810.58 | 12353.34 | 0.131 | 0.133 | 4.3936 |
| 558 | 15432.77 | 14153.8 | 0.133 | 0.101 | -9.0363 | 12308.61 | 12421.86 | 0.116 | 0.121 | 0.9118 |
| 559 | 12016.64 | 13107.99 | 0.133 | 0.134 | 8.3259 | 13902.01 | 13882.01 | 0.088 | 0.084 | -0.1441 |
| 560 | 13488.15 | 13598.26 | 0.133 | 0.1 | 0.8097 | 13502.13 | 13492.34 | 0.108 | 0.112 | -0.0726 |
| 561 | 13059.87 | 12938.05 | 0.133 | 0.1 | -0.9415 | 13136.12 | 13275.44 | 0.117 | 0.122 | 1.0495 |
| 562 | 15573.63 | 14618.3 | 0.133 | 0.113 | -6.5352 | 13317.29 | 13300.31 | 0.079 | 0.086 | -0.1277 |
| 563 | 18427.03 | 16983.38 | 0.133 | 0.109 | -8.5004 | 16392.35 | 16787.33 | 0.098 | 0.103 | 2.3528 |
| 564 | 12641.7 | 13695.77 | 0.133 | 0.133 | 7.6964 | 12548.63 | 13521.93 | 0.076 | 0.084 | 7.1979 |
| 565 | 13488.09 | 15643.32 | 0.133 | 0.129 | 13.777 | 11062.47 | 11169.96 | 0.131 | 0.135 | 0.9623 |
| 566 | 13067.12 | 14834.78 | 0.133 | 0.131 | 11.916 | 12287.74 | 12389.78 | 0.098 | 0.104 | 0.8236 |
| 567 | 14375.87 | 16294.08 | 0.133 | 0.132 | 11.772 | 12937.56 | 13492.88 | 0.122 | 0.117 | 4.1156 |
| 568 | 12726.39 | 13358.73 | 0.133 | 0.136 | 4.7335 | 12104.32 | 12437.28 | 0.11 | 0.115 | 2.6771 |
| 569 | 13711.5 | 14436.88 | 0.133 | 0.122 | 5.0245 | 11511.98 | 12532.18 | 0.078 | 0.075 | 8.1407 |
| 570 | 14634.37 | 13615.9 | 0.133 | 0.101 | -7.48 | 12016.35 | 12647.1 | 0.072 | 0.077 | 4.9873 |
| 571 | 12225.1 | 13046.01 | 0.133 | 0.136 | 6.2924 | 13393.08 | 13495.12 | 0.112 | 0.117 | 0.7562 |
| 572 | 11855.2 | 12272.51 | 0.133 | 0.136 | 3.4004 | 11056.37 | 11054.44 | 0.126 | 0.125 | -0.0174 |
| 573 | 11920.25 | 12857.51 | 0.133 | 0.136 | 7.2896 | 12483.75 | 12488.07 | 0.109 | 0.109 | 0.0345 |
| 574 | 17955.61 | 16477.61 | 0.133 | 0.111 | -8.9698 | 13469.29 | 13592.64 | 0.109 | 0.113 | 0.9074 |
| 575 | 11689.6 | 12312.34 | 0.133 | 0.092 | 5.0579 | 11248.74 | 11339.45 | 0.075 | 0.08 | 0.8 |
| 576 | 13293.16 | 15184.93 | 0.133 | 0.134 | 12.458 | 10661.34 | 10512.76 | 0.116 | 0.115 | -1.4133 |
| 577 | 12848.4 | 14458.97 | 0.133 | 0.135 | 11.139 | 12634.44 | 12611.72 | 0.081 | 0.082 | -0.1802 |
| 578 | 15317.56 | 16394.13 | 0.133 | 0.132 | 6.5668 | 12562.25 | 12699.23 | 0.082 | 0.08 | 1.0787 |
| 579 | 13397.59 | 13925.6 | 0.133 | 0.121 | 3.7917 | 14395.33 | 15659.3 | 0.109 | 0.108 | 8.0717 |
| 580 | 12959.49 | 14922.16 | 0.133 | 0.135 | 13.153 | 11946.74 | 11888.16 | 0.093 | 0.093 | -0.4927 |
| 581 | 13887.35 | 15364.51 | 0.133 | 0.133 | 9.6141 | 11305.07 | 11316.11 | 0.106 | 0.115 | 0.0976 |
| 582 | 13623.85 | 13958.81 | 0.133 | 0.122 | 2.3996 | 12482.49 | 12469.23 | 0.081 | 0.089 | -0.1063 |
| 583 | 12944.22 | 14810.9 | 0.133 | 0.134 | 12.603 | 14494.95 | 14363.95 | 0.083 | 0.084 | -0.912 |
| 584 | 16856.74 | 14882.41 | 0.133 | 0.11 | -13.266 | 10968.07 | 10945.35 | 0.091 | 0.092 | -0.2076 |
| 585 | 12583.53 | 12970.92 | 0.133 | 0.097 | 2.9866 | 15235.96 | 15229.72 | 0.102 | 0.107 | -0.041 |
| 586 | 13152.14 | 13495.38 | 0.133 | 0.121 | 2.5434 | 13903.74 | 14057.67 | 0.12 | 0.12 | 1.095 |
| 587 | 16104.93 | 16992.57 | 0.133 | 0.127 | 5.2237 | 10275.07 | 10386.4 | 0.125 | 0.123 | 1.0719 |
| 588 | 13485.51 | 14970.75 | 0.133 | 0.133 | 9.9209 | 12270.26 | 12261.98 | 0.093 | 0.09 | -0.0676 |
| 589 | 16802.1 | 14767.77 | 0.133 | 0.11 | -13.775 | 11535.35 | 11970.7 | 0.124 | 0.123 | 3.6368 |
| 590 | 18672.63 | 17310.57 | 0.133 | 0.112 | -7.8684 | 12870.3 | 13147.37 | 0.078 | 0.08 | 2.1074 |
| 591 | 13011.72 | 13338.4 | 0.133 | 0.12 | 2.4491 | 10665.04 | 10810.81 | 0.128 | 0.131 | 1.3484 |
| 592 | 13499.92 | 13694.05 | 0.134 | 0.12 | 1.4176 | 11727.56 | 12603.18 | 0.12 | 0.123 | 6.9476 |
| 593 | 17775.14 | 16641.96 | 0.134 | 0.11 | -6.8092 | 12221.53 | 12198.81 | 0.082 | 0.082 | -0.1863 |
| 594 | 15097.98 | 16117.46 | 0.134 | 0.134 | 6.3253 | 12909.02 | 13101.57 | 0.11 | 0.115 | 1.4697 |
| 595 | 16746.47 | 17693.2 | 0.134 | 0.128 | 5.3508 | 15787.56 | 15620.7 | 0.085 | 0.085 | -1.0682 |
| 596 | 13247.64 | 15082.58 | 0.134 | 0.131 | 12.166 | 11754.49 | 13316.86 | 0.071 | 0.072 | 11.732 |
| 597 | 15777.61 | 16333.21 | 0.134 | 0.13 | 3.4017 | 11630.26 | 11952.01 | 0.114 | 0.118 | 2.692 |
| 598 | 18235.86 | 16585.01 | 0.134 | 0.108 | -9.9539 | 11266.38 | 11635.49 | 0.079 | 0.089 | 3.1723 |

| 599 | 12315.91 | 14104.11 | 0.134 | 0.133 | 12.679 | 15920.37 | 15997.56 | 0.1 | 0.105 | 0.4825 |
| --- | --- | --- | --- | --- | --- | --- | --- | --- | --- | --- |
| 600 | 10955.45 | 11809.37 | 0.134 | 0.125 | 7.2309 | 11272.55 | 11374.6 | 0.097 | 0.103 | 0.8971 |
| 601 | 11505.48 | 14231.61 | 0.134 | 0.135 | 19.155 | 10986.17 | 11457 | 0.077 | 0.087 | 4.1096 |
| 602 | 12598.8 | 14416.9 | 0.134 | 0.134 | 12.611 | 16709.9 | 16663.54 | 0.103 | 0.103 | -0.2782 |
| 603 | 15530.49 | 14399.8 | 0.134 | 0.102 | -7.8521 | 13286.31 | 13261.04 | 0.081 | 0.088 | -0.1906 |
| 604 | 16130.17 | 16838.04 | 0.134 | 0.131 | 4.204 | 11630.94 | 11966.74 | 0.101 | 0.11 | 2.8062 |
| 605 | 13945.27 | 14670.96 | 0.134 | 0.131 | 4.9464 | 12046.18 | 14275.54 | 0.073 | 0.076 | 15.617 |
| 606 | 18137.18 | 16629.38 | 0.134 | 0.109 | -9.0671 | 12401.23 | 12442.49 | 0.119 | 0.119 | 0.3316 |
| 607 | 16395.97 | 14609.13 | 0.134 | 0.11 | -12.231 | 18427.36 | 18256.86 | 0.096 | 0.101 | -0.9339 |
| 608 | 12381.76 | 14963.41 | 0.134 | 0.133 | 17.253 | 12901.93 | 12867.28 | 0.11 | 0.113 | -0.2692 |
| 609 | 14100.28 | 14422.81 | 0.134 | 0.123 | 2.2363 | 12253.48 | 12351.77 | 0.083 | 0.083 | 0.7957 |
| 610 | 11067.43 | 12225.91 | 0.134 | 0.137 | 9.4756 | 15064.12 | 15170.31 | 0.108 | 0.112 | 0.7 |
| 611 | 13208.61 | 15033.37 | 0.134 | 0.133 | 12.138 | 12190.38 | 12153.31 | 0.119 | 0.122 | -0.305 |
| 612 | 12317.05 | 14214.68 | 0.134 | 0.134 | 13.35 | 10447.17 | 10504.96 | 0.13 | 0.132 | 0.55 |
| 613 | 17658.45 | 18953.08 | 0.134 | 0.129 | 6.8307 | 12348.54 | 12469.67 | 0.082 | 0.08 | 0.9714 |
| 614 | 13199.33 | 14253.41 | 0.134 | 0.134 | 7.3953 | 11449.97 | 11450.05 | 0.128 | 0.132 | 0.0008 |
| 615 | 13004.1 | 13496.96 | 0.134 | 0.121 | 3.6517 | 12123.84 | 12096.98 | 0.092 | 0.093 | -0.2221 |
| 616 | 12219.21 | 13557.77 | 0.134 | 0.134 | 9.873 | 10116.66 | 10145.66 | 0.125 | 0.123 | 0.2858 |
| 617 | 13109.98 | 13143.48 | 0.134 | 0.121 | 0.2549 | 11475.96 | 11462.03 | 0.095 | 0.1 | -0.1216 |
| 618 | 12301.19 | 12999.05 | 0.134 | 0.121 | 5.3685 | 11806.59 | 12544.82 | 0.101 | 0.114 | 5.8848 |
| 619 | 11230.1 | 12630.09 | 0.134 | 0.137 | 11.085 | 12810.76 | 12912.81 | 0.116 | 0.121 | 0.7903 |
| 620 | 17165.05 | 16292.09 | 0.134 | 0.11 | -5.3582 | 15022.23 | 15033.15 | 0.107 | 0.111 | 0.0726 |
| 621 | 15521 | 17029.22 | 0.134 | 0.131 | 8.8567 | 12357.45 | 12305.95 | 0.093 | 0.094 | -0.4186 |
| 622 | 13134.66 | 14330.66 | 0.134 | 0.134 | 8.3457 | 13822.4 | 13811.4 | 0.086 | 0.086 | -0.0797 |
| 623 | 13674.99 | 13808.74 | 0.134 | 0.123 | 0.9686 | 11145.35 | 13021.53 | 0.078 | 0.072 | 14.408 |
| 624 | 12376.9 | 13426.04 | 0.134 | 0.136 | 7.8142 | 13052.6 | 13108.58 | 0.11 | 0.116 | 0.427 |
| 625 | 13192.17 | 14246.25 | 0.134 | 0.134 | 7.399 | 12498.53 | 12678.36 | 0.111 | 0.109 | 1.4184 |
| 626 | 12850.06 | 14424.08 | 0.134 | 0.133 | 10.912 | 17141.32 | 17053.96 | 0.097 | 0.101 | -0.5123 |
| 627 | 16085.2 | 15117.47 | 0.134 | 0.115 | -6.4014 | 12675.97 | 13705.83 | 0.074 | 0.082 | 7.5141 |
| 628 | 13943.47 | 14671.99 | 0.134 | 0.122 | 4.9653 | 15157.63 | 15611.03 | 0.107 | 0.108 | 2.9043 |
| 629 | 13397.62 | 15136.67 | 0.134 | 0.135 | 11.489 | 13201.45 | 13303.49 | 0.111 | 0.116 | 0.7671 |
| 630 | 12555.25 | 13705.48 | 0.134 | 0.134 | 8.3925 | 11933.81 | 12783.02 | 0.13 | 0.127 | 6.6432 |
| 631 | 12629.48 | 13709.83 | 0.134 | 0.135 | 7.8801 | 12321.71 | 12216.77 | 0.1 | 0.108 | -0.859 |
| 632 | 13191.06 | 14955.22 | 0.134 | 0.132 | 11.796 | 11764.11 | 11741.39 | 0.084 | 0.085 | -0.1935 |
| 633 | 17141.82 | 18010.27 | 0.134 | 0.128 | 4.8219 | 13236.95 | 13339 | 0.108 | 0.113 | 0.765 |
| 634 | 14702 | 15356.42 | 0.134 | 0.135 | 4.2616 | 15990.13 | 16315.32 | 0.101 | 0.105 | 1.9931 |
| 635 | 12660.46 | 14543.44 | 0.134 | 0.134 | 12.947 | 10656.55 | 11220.14 | 0.099 | 0.106 | 5.023 |
| 636 | 13209.74 | 13903.59 | 0.134 | 0.132 | 4.9904 | 11574.27 | 11539.63 | 0.097 | 0.101 | -0.3002 |
| 637 | 12954.51 | 14831.31 | 0.134 | 0.134 | 12.654 | 15292.98 | 15451.05 | 0.116 | 0.116 | 1.0231 |
| 638 | 12322.7 | 13376.78 | 0.134 | 0.134 | 7.8799 | 11853.1 | 11830.38 | 0.086 | 0.086 | -0.1921 |
| 639 | 13206.44 | 15017.29 | 0.134 | 0.134 | 12.058 | 12975.53 | 13077.58 | 0.111 | 0.116 | 0.7803 |
| 640 | 16356.34 | 15477.71 | 0.134 | 0.112 | -5.6767 | 11719.46 | 11863.1 | 0.075 | 0.093 | 1.2108 |
| 641 | 14070.1 | 14576.78 | 0.134 | 0.122 | 3.4759 | 12853.52 | 12830.8 | 0.086 | 0.087 | -0.1771 |
| 642 | 12743.81 | 14527.16 | 0.134 | 0.134 | 12.276 | 11730.9 | 11690.73 | 0.112 | 0.112 | -0.3436 |
| 643 | 17239.44 | 15580.59 | 0.134 | 0.113 | -10.647 | 14223.11 | 15051.43 | 0.115 | 0.111 | 5.5033 |
| 644 | 15966.06 | 15613.36 | 0.135 | 0.115 | -2.259 | 13894.2 | 14372.82 | 0.112 | 0.109 | 3.33 |
| 645 | 11955.71 | 13440.95 | 0.135 | 0.134 | 11.05 | 14886.52 | 15255.13 | 0.101 | 0.107 | 2.4163 |
| 646 | 13049.67 | 14941.43 | 0.135 | 0.135 | 12.661 | 10617.62 | 11007.79 | 0.131 | 0.122 | 3.5445 |
| 647 | 16369.18 | 16830.72 | 0.135 | 0.131 | 2.7423 | 12663.9 | 12770.17 | 0.094 | 0.095 | 0.8322 |
| 648 | 12702.76 | 13783.11 | 0.135 | 0.136 | 7.8382 | 16011.85 | 16439.96 | 0.098 | 0.109 | 2.6041 |

| 649 | 14064.7 | 14773.5 | 0.135 | 0.103 | 4.7978 | 15118.49 | 15176.62 | 0.108 | 0.111 | 0.383 |
| --- | --- | --- | --- | --- | --- | --- | --- | --- | --- | --- |
| 650 | 16557.04 | 15336.89 | 0.135 | 0.115 | -7.9557 | 10429.12 | 10591.17 | 0.124 | 0.121 | 1.53 |
| 651 | 12372.29 | 14009.74 | 0.135 | 0.132 | 11.688 | 15401.79 | 15836.17 | 0.1 | 0.104 | 2.743 |
| 652 | 13439.64 | 13536.1 | 0.135 | 0.124 | 0.7127 | 9910.164 | 9897.321 | 0.13 | 0.134 | -0.1298 |
| 653 | 12659.33 | 13759.68 | 0.135 | 0.135 | 7.9969 | 18117.99 | 18061.89 | 0.111 | 0.093 | -0.3106 |
| 654 | 17972.74 | 19052.43 | 0.135 | 0.128 | 5.6669 | 15356.1 | 16333.04 | 0.106 | 0.109 | 5.9813 |
| 655 | 15815.31 | 16215.13 | 0.135 | 0.13 | 2.4658 | 11704.26 | 12514.79 | 0.105 | 0.088 | 6.4766 |
| 656 | 11887.47 | 12391.63 | 0.135 | 0.127 | 4.0686 | 12646.48 | 12702.43 | 0.084 | 0.093 | 0.4404 |
| 657 | 14393.61 | 15209.95 | 0.135 | 0.131 | 5.3672 | 11207.82 | 11651.71 | 0.099 | 0.105 | 3.8096 |
| 658 | 14774.34 | 15450.77 | 0.135 | 0.131 | 4.378 | 11935.11 | 12358.29 | 0.107 | 0.111 | 3.4242 |
| 659 | 16423.24 | 15719.67 | 0.135 | 0.112 | -4.4757 | 11111.28 | 11213.33 | 0.1 | 0.106 | 0.91 |
| 660 | 17733.6 | 17019.15 | 0.135 | 0.112 | -4.1979 | 11309.98 | 11404.12 | 0.119 | 0.117 | 0.8255 |
| 661 | 17237.05 | 16243.6 | 0.135 | 0.113 | -6.1159 | 13048.75 | 13887.49 | 0.067 | 0.073 | 6.0395 |
| 662 | 12787.17 | 13968.11 | 0.135 | 0.135 | 8.4545 | 10724.28 | 11922.82 | 0.115 | 0.112 | 10.053 |
| 663 | 14639.28 | 15239.14 | 0.135 | 0.134 | 3.9363 | 10231.18 | 10587.2 | 0.125 | 0.123 | 3.3627 |
| 664 | 12401.97 | 13875.09 | 0.135 | 0.136 | 10.617 | 15788.25 | 15969.04 | 0.111 | 0.117 | 1.1322 |
| 665 | 13038.77 | 14765.39 | 0.135 | 0.136 | 11.694 | 12540.38 | 12477.66 | 0.089 | 0.09 | -0.5027 |
| 666 | 12993.77 | 14047.84 | 0.135 | 0.135 | 7.5035 | 10602.64 | 10613.68 | 0.105 | 0.115 | 0.104 |
| 667 | 15507.44 | 14407.46 | 0.135 | 0.103 | -7.6348 | 12239.73 | 12318.94 | 0.092 | 0.093 | 0.6429 |
| 668 | 12002.14 | 13082.49 | 0.135 | 0.136 | 8.258 | 10707.43 | 10718.47 | 0.107 | 0.117 | 0.103 |
| 669 | 17195.13 | 15870.73 | 0.135 | 0.112 | -8.3449 | 11301.61 | 11747.64 | 0.128 | 0.123 | 3.7968 |
| 670 | 14912.39 | 15408.2 | 0.135 | 0.131 | 3.2178 | 13856.07 | 13954.98 | 0.066 | 0.069 | 0.7088 |
| 671 | 12532.68 | 16064.03 | 0.135 | 0.131 | 21.983 | 12759.85 | 12828.73 | 0.076 | 0.088 | 0.5369 |
| 672 | 12148.74 | 13389.94 | 0.135 | 0.137 | 9.2696 | 9457.605 | 9578.229 | 0.129 | 0.125 | 1.2594 |
| 673 | 11462.26 | 13925.71 | 0.135 | 0.134 | 17.69 | 12693.52 | 14520.96 | 0.075 | 0.077 | 12.585 |
| 674 | 12506.7 | 13587.05 | 0.135 | 0.136 | 7.9513 | 15709.66 | 15758.15 | 0.083 | 0.084 | 0.3077 |
| 675 | 15604.41 | 16510.76 | 0.135 | 0.132 | 5.4894 | 12972.16 | 12943.87 | 0.092 | 0.094 | -0.2185 |
| 676 | 15746.8 | 16479.6 | 0.135 | 0.131 | 4.4467 | 12585.19 | 12798.15 | 0.107 | 0.106 | 1.664 |
| 677 | 11955.61 | 12377.84 | 0.135 | 0.126 | 3.4112 | 11441.35 | 11562.36 | 0.085 | 0.087 | 1.0465 |
| 678 | 11451.13 | 12693.75 | 0.135 | 0.138 | 9.7892 | 9851.123 | 10111.57 | 0.127 | 0.125 | 2.5758 |
| 679 | 16170.99 | 17080.14 | 0.135 | 0.129 | 5.3229 | 14972.76 | 14966.52 | 0.105 | 0.11 | -0.0417 |
| 680 | 16675.91 | 16879.74 | 0.135 | 0.11 | 1.2076 | 13067.66 | 13049.59 | 0.112 | 0.116 | -0.1385 |
| 681 | 12429.85 | 13737.51 | 0.135 | 0.134 | 9.5189 | 14360.25 | 14378.24 | 0.108 | 0.112 | 0.1251 |
| 682 | 12715.19 | 13948.73 | 0.135 | 0.136 | 8.8434 | 13009.71 | 12726.99 | 0.085 | 0.088 | -2.2214 |
| 683 | 17435.67 | 18515.92 | 0.135 | 0.131 | 5.8341 | 11316.28 | 11418.33 | 0.104 | 0.11 | 0.8937 |
| 684 | 13015.25 | 14388.08 | 0.135 | 0.134 | 9.5414 | 12008.82 | 12164.98 | 0.126 | 0.123 | 1.2836 |
| 685 | 13190.9 | 13350.68 | 0.135 | 0.122 | 1.1968 | 12684.2 | 12746.01 | 0.075 | 0.086 | 0.4849 |
| 686 | 13359.23 | 15250.99 | 0.135 | 0.135 | 12.404 | 17900.02 | 18271.27 | 0.1 | 0.103 | 2.0319 |
| 687 | 15227.54 | 15869.41 | 0.135 | 0.13 | 4.0447 | 10362.49 | 10464.54 | 0.103 | 0.109 | 0.9752 |
| 688 | 15338.31 | 16095.45 | 0.135 | 0.131 | 4.7041 | 15114.33 | 15126.96 | 0.107 | 0.11 | 0.0835 |
| 689 | 13125.4 | 12851.81 | 0.135 | 0.124 | -2.1287 | 11878.3 | 12086.41 | 0.122 | 0.12 | 1.7219 |
| 690 | 13001.32 | 14897.73 | 0.135 | 0.136 | 12.73 | 12029.49 | 13628.02 | 0.076 | 0.075 | 11.73 |
| 691 | 11985.57 | 12970.91 | 0.135 | 0.136 | 7.5965 | 11892.95 | 12616.38 | 0.103 | 0.11 | 5.7341 |
| 692 | 12313.19 | 12945.53 | 0.135 | 0.139 | 4.8846 | 12357.55 | 12541.78 | 0.121 | 0.121 | 1.4689 |
| 693 | 13544.77 | 14320.25 | 0.135 | 0.131 | 5.4153 | 13188.61 | 14175.54 | 0.095 | 0.08 | 6.9622 |
| 694 | 15647.07 | 16181.46 | 0.135 | 0.13 | 3.3025 | 13341.24 | 13877.31 | 0.118 | 0.122 | 3.8629 |
| 695 | 15519.79 | 16053.47 | 0.135 | 0.131 | 3.3244 | 13697.13 | 13648.43 | 0.091 | 0.094 | -0.3568 |
| 696 | 16050 | 17015.84 | 0.135 | 0.13 | 5.6761 | 12581.19 | 12556.21 | 0.082 | 0.09 | -0.1989 |
| 697 | 17732.88 | 15935.38 | 0.135 | 0.11 | -11.28 | 13241.42 | 13218.7 | 0.085 | 0.086 | -0.1719 |
| 698 | 12702.28 | 14052.25 | 0.135 | 0.131 | 9.6068 | 10501.84 | 10588.83 | 0.131 | 0.133 | 0.8215 |

| 699 | 11504.72 | 11917.39 | 0.135 | 0.138 | 3.4628 | 11517.11 | 11529.54 | 0.115 | 0.113 | 0.1078 |
| --- | --- | --- | --- | --- | --- | --- | --- | --- | --- | --- |
| 700 | 12526.82 | 13714.59 | 0.135 | 0.137 | 8.6606 | 11534.38 | 12689.07 | 0.114 | 0.118 | 9.0999 |
| 701 | 14558.39 | 14312.59 | 0.135 | 0.102 | -1.7174 | 12894.17 | 13317.34 | 0.104 | 0.109 | 3.1776 |
| 702 | 12858.94 | 13637.55 | 0.135 | 0.134 | 5.7093 | 12030.22 | 12016.29 | 0.096 | 0.101 | -0.116 |
| 703 | 17469.77 | 16652.3 | 0.135 | 0.109 | -4.909 | 11602.71 | 12606.77 | 0.077 | 0.089 | 7.9644 |
| 704 | 14233.5 | 15259.36 | 0.135 | 0.135 | 6.7228 | 14000.68 | 14819 | 0.097 | 0.093 | 5.5221 |
| 705 | 11900.86 | 13100.09 | 0.135 | 0.136 | 9.1543 | 11165.81 | 11518.69 | 0.101 | 0.111 | 3.0635 |
| 706 | 12219.86 | 13273.94 | 0.135 | 0.136 | 7.941 | 10821.35 | 11200.68 | 0.11 | 0.115 | 3.3866 |
| 707 | 16270.68 | 15585.51 | 0.135 | 0.111 | -4.3961 | 13649.39 | 13586.67 | 0.091 | 0.091 | -0.4616 |
| 708 | 10666.4 | 11963.04 | 0.136 | 0.137 | 10.839 | 11820.1 | 11911.23 | 0.124 | 0.126 | 0.7651 |
| 709 | 12593.53 | 14208.24 | 0.136 | 0.136 | 11.365 | 12129.67 | 12115.74 | 0.105 | 0.109 | -0.115 |
| 710 | 12308.49 | 13141.34 | 0.136 | 0.135 | 6.3376 | 12393.29 | 13886.21 | 0.074 | 0.092 | 10.751 |
| 711 | 17243.9 | 18404.05 | 0.136 | 0.132 | 6.3038 | 16966.6 | 17697.19 | 0.112 | 0.108 | 4.1283 |
| 712 | 14121.23 | 14986.49 | 0.136 | 0.133 | 5.7737 | 11729.48 | 11740.52 | 0.108 | 0.117 | 0.094 |
| 713 | 11594.54 | 11852.77 | 0.136 | 0.139 | 2.1787 | 14849.8 | 14827.08 | 0.087 | 0.087 | -0.1532 |
| 714 | 14597.15 | 15403.96 | 0.136 | 0.133 | 5.2377 | 12576.07 | 12513.35 | 0.091 | 0.091 | -0.5012 |
| 715 | 12035.07 | 13185.3 | 0.136 | 0.135 | 8.7236 | 12547.96 | 12650 | 0.111 | 0.117 | 0.8067 |
| 716 | 12092.27 | 13492.64 | 0.136 | 0.139 | 10.379 | 14757.72 | 15150.61 | 0.116 | 0.114 | 2.5932 |
| 717 | 15255.62 | 15731.72 | 0.136 | 0.13 | 3.0264 | 15741.16 | 15734.93 | 0.104 | 0.109 | -0.0396 |
| 718 | 14875.85 | 15461.86 | 0.136 | 0.131 | 3.7901 | 14389.48 | 14659.78 | 0.117 | 0.118 | 1.8438 |
| 719 | 17072.26 | 16210.32 | 0.136 | 0.112 | -5.3172 | 11358.39 | 11699.72 | 0.081 | 0.091 | 2.9175 |
| 720 | 12182.41 | 13236.48 | 0.136 | 0.136 | 7.9634 | 9837.199 | 9956.661 | 0.124 | 0.123 | 1.1998 |
| 721 | 11776.93 | 12969.13 | 0.136 | 0.136 | 9.1926 | 15331.75 | 15408.95 | 0.101 | 0.106 | 0.501 |
| 722 | 12174.83 | 13296.28 | 0.136 | 0.135 | 8.4343 | 12278.4 | 12268.61 | 0.116 | 0.12 | -0.0798 |
| 723 | 12892.41 | 14253.59 | 0.136 | 0.135 | 9.5498 | 13493.29 | 12987.76 | 0.095 | 0.077 | -3.8923 |
| 724 | 13271.2 | 13972.75 | 0.136 | 0.134 | 5.0208 | 13047.07 | 13408.82 | 0.106 | 0.11 | 2.6979 |
| 725 | 16453.54 | 15606.11 | 0.136 | 0.116 | -5.4301 | 10630.3 | 10732.35 | 0.099 | 0.105 | 0.9508 |
| 726 | 15907.02 | 16797.72 | 0.136 | 0.13 | 5.3025 | 15270.81 | 14683.53 | 0.096 | 0.078 | -3.9996 |
| 727 | 10638.5 | 13406.93 | 0.136 | 0.134 | 20.649 | 12184.7 | 12434.32 | 0.071 | 0.078 | 2.0075 |
| 728 | 11263.06 | 13244.41 | 0.136 | 0.136 | 14.96 | 17641 | 17279.29 | 0.099 | 0.104 | -2.0933 |
| 729 | 16397.5 | 15416.05 | 0.136 | 0.115 | -6.3664 | 15578.91 | 15726.44 | 0.109 | 0.11 | 0.9382 |
| 730 | 12704.5 | 14369.44 | 0.136 | 0.135 | 11.587 | 11454.82 | 11556.33 | 0.129 | 0.129 | 0.8784 |
| 731 | 14353.15 | 15243.31 | 0.136 | 0.133 | 5.8397 | 12710.92 | 12467.7 | 0.084 | 0.088 | -1.9508 |
| 732 | 11618.38 | 11560.25 | 0.136 | 0.122 | -0.5028 | 14069.2 | 14462.08 | 0.098 | 0.105 | 2.7166 |
| 733 | 11576.94 | 13274.59 | 0.136 | 0.138 | 12.789 | 11923.99 | 12305.65 | 0.101 | 0.11 | 3.1015 |
| 734 | 11644.98 | 12847.1 | 0.136 | 0.138 | 9.3571 | 17421.24 | 17382.45 | 0.099 | 0.103 | -0.2231 |
| 735 | 16440.65 | 17247.5 | 0.136 | 0.132 | 4.6781 | 10913.41 | 11015.46 | 0.106 | 0.113 | 0.9264 |
| 736 | 11537.27 | 12160.82 | 0.136 | 0.139 | 5.1276 | 16835.05 | 16972.33 | 0.099 | 0.103 | 0.8088 |
| 737 | 12764.93 | 14464.83 | 0.136 | 0.136 | 11.752 | 15711.27 | 16620.56 | 0.109 | 0.105 | 5.4709 |
| 738 | 11586.22 | 12562.93 | 0.136 | 0.136 | 7.7746 | 16887.62 | 16848.83 | 0.098 | 0.103 | -0.2302 |
| 739 | 11696.67 | 13798.96 | 0.136 | 0.136 | 15.235 | 12966.91 | 13103.24 | 0.114 | 0.115 | 1.0404 |
| 740 | 17273.98 | 15789.62 | 0.136 | 0.11 | -9.4009 | 12831.66 | 14340.86 | 0.075 | 0.086 | 10.524 |
| 741 | 12831.26 | 13885.33 | 0.136 | 0.136 | 7.5913 | 12140.62 | 12077.9 | 0.094 | 0.095 | -0.5193 |
| 742 | 17486.55 | 16624.94 | 0.136 | 0.114 | -5.1826 | 13803.23 | 13916.99 | 0.11 | 0.115 | 0.8174 |
| 743 | 14032.98 | 14813.23 | 0.136 | 0.133 | 5.2672 | 11392.2 | 11403.24 | 0.108 | 0.117 | 0.0968 |
| 744 | 12208.35 | 13528.63 | 0.136 | 0.137 | 9.7592 | 11897.69 | 11874.97 | 0.088 | 0.088 | -0.1913 |
| 745 | 11032.47 | 12108.52 | 0.136 | 0.139 | 8.8867 | 12390.94 | 12758.04 | 0.13 | 0.132 | 2.8775 |
| 746 | 17002.34 | 16415.88 | 0.136 | 0.111 | -3.5725 | 15826.24 | 17127.15 | 0.08 | 0.086 | 7.5957 |
| 747 | 16466.2 | 17817.65 | 0.136 | 0.132 | 7.5849 | 13515.71 | 13493.29 | 0.085 | 0.082 | -0.1662 |
| 748 | 13590.67 | 14387.48 | 0.136 | 0.134 | 5.5382 | 10127.79 | 10295.69 | 0.122 | 0.12 | 1.6308 |

| 749 | 15502 | 16514.74 | 0.136 | 0.131 | 6.1323 | 12153.63 | 12145.35 | 0.089 | 0.087 | -0.0682 |
| --- | --- | --- | --- | --- | --- | --- | --- | --- | --- | --- |
| 750 | 16364.38 | 17288.42 | 0.136 | 0.132 | 5.3449 | 12755.97 | 12858.02 | 0.115 | 0.12 | 0.7936 |
| 751 | 13145.96 | 13438.24 | 0.136 | 0.125 | 2.1749 | 13193.79 | 13131.07 | 0.09 | 0.091 | -0.4777 |
| 752 | 14676.73 | 15774.53 | 0.136 | 0.134 | 6.9593 | 12071.62 | 12513.21 | 0.116 | 0.111 | 3.529 |
| 753 | 15965.88 | 16932.35 | 0.136 | 0.131 | 5.7078 | 14334.92 | 14303.2 | 0.087 | 0.084 | -0.2217 |
| 754 | 11147.35 | 12486.92 | 0.136 | 0.137 | 10.728 | 14585.23 | 14718.63 | 0.113 | 0.114 | 0.9063 |
| 755 | 13875.31 | 14658.65 | 0.136 | 0.133 | 5.3439 | 12745.86 | 12723.14 | 0.088 | 0.088 | -0.1786 |
| 756 | 11941.43 | 12524 | 0.136 | 0.127 | 4.6516 | 12661.66 | 12570.74 | 0.107 | 0.104 | -0.7233 |
| 757 | 10823.29 | 13008.49 | 0.137 | 0.137 | 16.798 | 15034.09 | 15349.07 | 0.101 | 0.107 | 2.0521 |
| 758 | 14235.52 | 15069.61 | 0.137 | 0.133 | 5.5349 | 11565.71 | 11707.22 | 0.123 | 0.123 | 1.2087 |
| 759 | 12433.85 | 13646.27 | 0.137 | 0.137 | 8.8846 | 12291.6 | 12714.77 | 0.105 | 0.109 | 3.3282 |
| 760 | 13763.53 | 13513.09 | 0.137 | 0.125 | -1.8534 | 11677.77 | 12161.08 | 0.118 | 0.112 | 3.9742 |
| 761 | 17296.34 | 15880.55 | 0.137 | 0.111 | -8.9152 | 11461.71 | 11870.87 | 0.124 | 0.118 | 3.4468 |
| 762 | 11785.28 | 12033.09 | 0.137 | 0.135 | 2.0595 | 11156.13 | 11335.96 | 0.115 | 0.114 | 1.5863 |
| 763 | 14724.72 | 15659.06 | 0.137 | 0.134 | 5.9667 | 13211.45 | 13593.11 | 0.097 | 0.105 | 2.8078 |
| 764 | 14559.37 | 15537.34 | 0.137 | 0.133 | 6.2944 | 17725.14 | 17408.78 | 0.099 | 0.103 | -1.8172 |
| 765 | 11721.37 | 12775.44 | 0.137 | 0.136 | 8.2508 | 11557.3 | 11574.37 | 0.091 | 0.089 | 0.1475 |
| 766 | 15956.61 | 16761.53 | 0.137 | 0.13 | 4.8022 | 12857.38 | 12840.39 | 0.08 | 0.087 | -0.1323 |
| 767 | 12117.02 | 13045.37 | 0.137 | 0.136 | 7.1164 | 14927.68 | 15543.33 | 0.09 | 0.086 | 3.9609 |
| 768 | 16878.34 | 16382.45 | 0.137 | 0.117 | -3.0269 | 17791.71 | 17756.86 | 0.103 | 0.105 | -0.1963 |
| 769 | 11912.43 | 13062.66 | 0.137 | 0.137 | 8.8055 | 15152.19 | 15563.65 | 0.093 | 0.103 | 2.6437 |
| 770 | 12192.64 | 13246.72 | 0.137 | 0.137 | 7.9573 | 12386.47 | 12418.4 | 0.097 | 0.094 | 0.2571 |
| 771 | 16691.86 | 17615.7 | 0.137 | 0.131 | 5.2444 | 10889.48 | 10805.84 | 0.122 | 0.114 | -0.774 |
| 772 | 15160.32 | 15941.78 | 0.137 | 0.133 | 4.9019 | 15089.68 | 15166.88 | 0.102 | 0.107 | 0.509 |
| 773 | 12200.01 | 13222.87 | 0.137 | 0.137 | 7.7356 | 16640.32 | 16542.95 | 0.101 | 0.105 | -0.5886 |
| 774 | 12695.78 | 14627.29 | 0.137 | 0.133 | 13.205 | 13014.67 | 13116.72 | 0.115 | 0.12 | 0.778 |
| 775 | 12133.14 | 13325.35 | 0.137 | 0.137 | 8.9469 | 13185.09 | 13214.79 | 0.112 | 0.117 | 0.2248 |
| 776 | 12494.77 | 13424.13 | 0.137 | 0.135 | 6.9231 | 13216.07 | 13921.93 | 0.114 | 0.12 | 5.0701 |
| 777 | 12422.85 | 13593.34 | 0.137 | 0.137 | 8.6107 | 13701.84 | 14076.02 | 0.108 | 0.112 | 2.6583 |
| 778 | 12131.43 | 13674.08 | 0.137 | 0.137 | 11.282 | 11946.36 | 12135.65 | 0.127 | 0.127 | 1.5598 |
| 779 | 11596.01 | 12506.86 | 0.137 | 0.137 | 7.2828 | 11880.8 | 11886.74 | 0.083 | 0.091 | 0.05 |
| 780 | 13212.05 | 13749.45 | 0.137 | 0.134 | 3.9085 | 13234.11 | 13347.87 | 0.116 | 0.121 | 0.8523 |
| 781 | 11743.33 | 13474.65 | 0.137 | 0.137 | 12.849 | 12294.07 | 12740.1 | 0.123 | 0.118 | 3.501 |
| 782 | 11666.2 | 12293.9 | 0.137 | 0.14 | 5.1057 | 12158.67 | 12135.95 | 0.084 | 0.085 | -0.1872 |
| 783 | 11567.05 | 13219.33 | 0.137 | 0.127 | 12.499 | 12351.61 | 12465.37 | 0.103 | 0.108 | 0.9126 |
| 784 | 12662.26 | 14135.57 | 0.137 | 0.135 | 10.423 | 12970.3 | 12956.36 | 0.113 | 0.117 | -0.1075 |
| 785 | 16960.47 | 15804.08 | 0.137 | 0.117 | -7.317 | 14224 | 14337.26 | 0.111 | 0.116 | 0.79 |
| 786 | 12468.56 | 13953.79 | 0.137 | 0.137 | 10.644 | 10641.23 | 10529.77 | 0.117 | 0.116 | -1.0585 |
| 787 | 11640.86 | 11297.76 | 0.137 | 0.122 | -3.0369 | 13954.94 | 13923.23 | 0.096 | 0.093 | -0.2278 |
| 788 | 17428.38 | 16576.57 | 0.137 | 0.114 | -5.1387 | 11025.92 | 11077.35 | 0.114 | 0.109 | 0.4642 |
| 789 | 11849.72 | 12704.77 | 0.137 | 0.129 | 6.7301 | 12714.9 | 13235.37 | 0.121 | 0.116 | 3.9324 |
| 790 | 15472.5 | 16506.46 | 0.137 | 0.132 | 6.2639 | 13141.09 | 13522.76 | 0.097 | 0.105 | 2.8224 |
| 791 | 12012.28 | 13497.51 | 0.137 | 0.137 | 11.004 | 14833.89 | 14771.16 | 0.089 | 0.089 | -0.4246 |
| 792 | 11132.32 | 13264.82 | 0.137 | 0.137 | 16.076 | 12944.55 | 13058.31 | 0.117 | 0.122 | 0.8712 |
| 793 | 12923.06 | 14280.1 | 0.137 | 0.137 | 9.503 | 12399.82 | 13499.47 | 0.063 | 0.072 | 8.1459 |
| 794 | 10908.53 | 11223.13 | 0.137 | 0.14 | 2.8031 | 11768.47 | 11874.66 | 0.102 | 0.107 | 0.8942 |
| 795 | 11115.37 | 11461.35 | 0.137 | 0.141 | 3.0187 | 14762.17 | 14773.09 | 0.106 | 0.11 | 0.0739 |
| 796 | 12476.22 | 14816.4 | 0.137 | 0.136 | 15.795 | 13060.21 | 14191.84 | 0.069 | 0.076 | 7.9738 |
| 797 | 11754.17 | 12192.98 | 0.138 | 0.127 | 3.5989 | 10983.03 | 10857.38 | 0.102 | 0.108 | -1.1573 |
| 798 | 12390.73 | 12656.83 | 0.138 | 0.135 | 2.1024 | 14994.83 | 15007.46 | 0.107 | 0.11 | 0.0842 |

| 799 | 11613.85 | 12667.93 | 0.138 | 0.137 | 8.3208 | 13085.95 | 13199.71 | 0.117 | 0.122 | 0.8618 |
| --- | --- | --- | --- | --- | --- | --- | --- | --- | --- | --- |
| 800 | 11504.33 | 12245.2 | 0.138 | 0.129 | 6.0503 | 12090.26 | 11478.8 | 0.069 | 0.074 | -5.3268 |
| 801 | 12160.41 | 12866.6 | 0.138 | 0.134 | 5.4885 | 12409.97 | 12819.64 | 0.122 | 0.116 | 3.1956 |
| 802 | 11998.79 | 13191 | 0.138 | 0.138 | 9.038 | 16757.83 | 16622.68 | 0.098 | 0.102 | -0.813 |
| 803 | 11579.21 | 12202.99 | 0.138 | 0.127 | 5.1117 | 13750.18 | 16201.97 | 0.068 | 0.07 | 15.133 |
| 804 | 16743.19 | 17682.59 | 0.138 | 0.132 | 5.3126 | 15994.93 | 15914.42 | 0.083 | 0.084 | -0.5058 |
| 805 | 16673.13 | 17716.08 | 0.138 | 0.132 | 5.887 | 12488.75 | 12613.23 | 0.113 | 0.119 | 0.9868 |
| 806 | 12521.6 | 13179.2 | 0.138 | 0.135 | 4.9897 | 13287.39 | 13258.61 | 0.089 | 0.086 | -0.2171 |
| 807 | 12095.72 | 13409.41 | 0.138 | 0.136 | 9.7968 | 10967.14 | 11495.87 | 0.114 | 0.114 | 4.5994 |
| 808 | 11950.13 | 14179.39 | 0.138 | 0.135 | 15.722 | 11616.35 | 11998.02 | 0.101 | 0.11 | 3.1811 |
| 809 | 11803.73 | 12690.95 | 0.138 | 0.138 | 6.9909 | 12853.43 | 12830.71 | 0.082 | 0.083 | -0.1771 |
| 810 | 13104 | 13818.97 | 0.138 | 0.134 | 5.1739 | 11761.53 | 11820.95 | 0.085 | 0.087 | 0.5026 |
| 811 | 11898.17 | 12258.15 | 0.138 | 0.124 | 2.9367 | 12660.95 | 12647.01 | 0.112 | 0.117 | -0.1102 |
| 812 | 17344.79 | 16515.4 | 0.138 | 0.115 | -5.0219 | 14645.17 | 15107.77 | 0.121 | 0.117 | 3.062 |
| 813 | 11382.81 | 12505.97 | 0.138 | 0.138 | 8.981 | 11388.19 | 11365.47 | 0.087 | 0.088 | -0.1999 |
| 814 | 12416.05 | 13470.12 | 0.138 | 0.138 | 7.8253 | 12496.74 | 12781.93 | 0.11 | 0.115 | 2.2311 |
| 815 | 12084.38 | 13427.3 | 0.138 | 0.138 | 10.001 | 12615.01 | 13201.55 | 0.124 | 0.119 | 4.4429 |
| 816 | 11392.15 | 12024.49 | 0.138 | 0.141 | 5.2588 | 12712.51 | 12689.79 | 0.085 | 0.085 | -0.179 |
| 817 | 12010.53 | 12634.46 | 0.138 | 0.141 | 4.9383 | 13001.6 | 13103.64 | 0.118 | 0.123 | 0.7788 |
| 818 | 11507.69 | 11987.47 | 0.138 | 0.137 | 4.0023 | 10372.08 | 10383.12 | 0.102 | 0.112 | 0.1063 |
| 819 | 12586.61 | 12743.09 | 0.138 | 0.136 | 1.228 | 11364.6 | 11793.42 | 0.069 | 0.076 | 3.6361 |
| 820 | 11345.72 | 12918.49 | 0.138 | 0.137 | 12.175 | 10595.58 | 11215.58 | 0.127 | 0.123 | 5.528 |
| 821 | 11679.71 | 12923.67 | 0.138 | 0.139 | 9.6254 | 13655.86 | 13715.06 | 0.108 | 0.112 | 0.4317 |
| 822 | 11948.04 | 13641.47 | 0.138 | 0.138 | 12.414 | 11924.02 | 11861.3 | 0.087 | 0.088 | -0.5288 |
| 823 | 12260.78 | 13764.47 | 0.138 | 0.138 | 10.924 | 12147.97 | 12352.65 | 0.111 | 0.11 | 1.657 |
| 824 | 11818.29 | 12872.37 | 0.138 | 0.138 | 8.1887 | 11795.29 | 14274.14 | 0.076 | 0.078 | 17.366 |
| 825 | 12464.59 | 13057.64 | 0.138 | 0.136 | 4.5418 | 12080.47 | 12182.51 | 0.117 | 0.123 | 0.8376 |
| 826 | 11763.82 | 13150.58 | 0.138 | 0.138 | 10.545 | 11969.13 | 11969.13 | 0.072 | 0.072 | 0 |
| 827 | 11169.44 | 12999.79 | 0.138 | 0.139 | 14.08 | 16886.68 | 16764.46 | 0.099 | 0.103 | -0.729 |
| 828 | 12019.42 | 12394.93 | 0.138 | 0.137 | 3.0296 | 11334.34 | 11311.62 | 0.089 | 0.09 | -0.2009 |
| 829 | 11429.82 | 12027.8 | 0.138 | 0.137 | 4.9716 | 10665.55 | 10734.93 | 0.087 | 0.098 | 0.6463 |
| 830 | 10714.97 | 11123.12 | 0.138 | 0.141 | 3.6694 | 11890.41 | 11705.14 | 0.124 | 0.124 | -1.5828 |
| 831 | 12787.29 | 14503.87 | 0.138 | 0.137 | 11.835 | 16243.57 | 16178.83 | 0.085 | 0.086 | -0.4001 |
| 832 | 10842.76 | 11918.24 | 0.138 | 0.139 | 9.0238 | 15399.15 | 15409.87 | 0.102 | 0.106 | 0.0695 |
| 833 | 11111.18 | 12385.02 | 0.138 | 0.138 | 10.285 | 11482.11 | 11584.16 | 0.099 | 0.105 | 0.8809 |
| 834 | 12216.93 | 13700.12 | 0.138 | 0.138 | 10.826 | 12658.06 | 12926.1 | 0.118 | 0.118 | 2.0736 |
| 835 | 11406.12 | 12270.41 | 0.138 | 0.139 | 7.0437 | 11260.02 | 11909.76 | 0.096 | 0.103 | 5.4555 |
| 836 | 11997.91 | 13845.75 | 0.138 | 0.138 | 13.346 | 11511.27 | 11511.27 | 0.083 | 0.083 | 0 |
| 837 | 11296.27 | 12893.63 | 0.138 | 0.138 | 12.389 | 11336.84 | 11365.84 | 0.123 | 0.121 | 0.2551 |
| 838 | 11412.69 | 12639.96 | 0.138 | 0.139 | 9.7094 | 10263.6 | 10463.42 | 0.114 | 0.12 | 1.9098 |
| 839 | 11867.31 | 12776.45 | 0.138 | 0.14 | 7.1157 | 13281.42 | 13522.84 | 0.088 | 0.089 | 1.7853 |
| 840 | 11546.72 | 12505.24 | 0.138 | 0.141 | 7.6649 | 11059.81 | 11227.5 | 0.109 | 0.113 | 1.4936 |
| 841 | 11129.69 | 13236.12 | 0.138 | 0.138 | 15.914 | 11438.82 | 11890.71 | 0.13 | 0.125 | 3.8003 |
| 842 | 12174.18 | 12453.21 | 0.138 | 0.137 | 2.2406 | 13703.11 | 14069.3 | 0.104 | 0.108 | 2.6027 |
| 843 | 12729 | 14229.52 | 0.139 | 0.137 | 10.545 | 13988.7 | 14002.84 | 0.078 | 0.079 | 0.101 |
| 844 | 11041.01 | 12037.77 | 0.139 | 0.14 | 8.2803 | 12193.79 | 12573.21 | 0.108 | 0.115 | 3.0177 |
| 845 | 12172.03 | 12465.2 | 0.139 | 0.136 | 2.3519 | 10295.92 | 10325.92 | 0.119 | 0.114 | 0.2905 |
| 846 | 11072.4 | 11971.79 | 0.139 | 0.139 | 7.5126 | 12857.92 | 13796.24 | 0.1 | 0.097 | 6.8013 |
| 847 | 11201.31 | 13015.21 | 0.139 | 0.139 | 13.937 | 11350.73 | 11732.39 | 0.1 | 0.109 | 3.2531 |
| 848 | 12302.59 | 12898.78 | 0.139 | 0.137 | 4.622 | 11935.7 | 11952.77 | 0.086 | 0.086 | 0.1428 |
| 849 | 13132.64 | 13670.04 | 0.139 | 0.135 | 3.9312 | 14897.46 | 14849.89 | 0.083 | 0.085 | -0.3204 |

| 850 | 13155.27 | 13990.04 | 0.139 | 0.135 | 5.9669 | 14107.11 | 14093.17 | 0.107 | 0.111 | -0.0989 |
| --- | --- | --- | --- | --- | --- | --- | --- | --- | --- | --- |
| 851 | 11621.72 | 12507.56 | 0.139 | 0.14 | 7.0825 | 12720.17 | 12657.45 | 0.096 | 0.097 | -0.4955 |
| 852 | 11554 | 13249.2 | 0.139 | 0.138 | 12.795 | 11354.56 | 11460.75 | 0.104 | 0.11 | 0.9265 |
| 853 | 11339.43 | 13059.43 | 0.139 | 0.138 | 13.171 | 11024.7 | 11494.78 | 0.131 | 0.128 | 4.0896 |
| 854 | 11528.33 | 11772 | 0.139 | 0.137 | 2.07 | 9419.59 | 9581.636 | 0.126 | 0.123 | 1.6912 |
| 855 | 10916.07 | 12114.64 | 0.139 | 0.14 | 9.8935 | 12860.82 | 12962.87 | 0.106 | 0.112 | 0.7872 |
| 856 | 12302.7 | 13600.85 | 0.139 | 0.138 | 9.5446 | 12617.51 | 12612.96 | 0.088 | 0.095 | -0.0361 |
| 857 | 11683.35 | 12986.51 | 0.139 | 0.14 | 10.035 | 12286.55 | 12482.7 | 0.121 | 0.122 | 1.5714 |
| 858 | 12888.02 | 13516.21 | 0.139 | 0.135 | 4.6477 | 12049.11 | 12026.39 | 0.089 | 0.089 | -0.1889 |
| 859 | 11133.73 | 11621.79 | 0.139 | 0.138 | 4.1995 | 11722.74 | 11702.74 | 0.096 | 0.093 | -0.1709 |
| 860 | 11745.42 | 12644.07 | 0.139 | 0.137 | 7.1073 | 13748.91 | 13686.19 | 0.087 | 0.087 | -0.4583 |
| 861 | 13117.1 | 13832.78 | 0.139 | 0.136 | 5.1738 | 15109.64 | 15495.79 | 0.112 | 0.11 | 2.492 |
| 862 | 11726.11 | 12005.14 | 0.139 | 0.138 | 2.3243 | 12332.06 | 12312.06 | 0.089 | 0.086 | -0.1624 |
| 863 | 11734.95 | 13104.55 | 0.139 | 0.137 | 10.451 | 11644.66 | 11552.15 | 0.102 | 0.111 | -0.8008 |
| 864 | 10642.84 | 11664.96 | 0.139 | 0.14 | 8.7623 | 14898.56 | 16044.48 | 0.1 | 0.108 | 7.1422 |
| 865 | 11782.19 | 12662.71 | 0.139 | 0.139 | 6.9537 | 12552.16 | 12533.37 | 0.091 | 0.094 | -0.1499 |
| 866 | 12851.21 | 13368.11 | 0.139 | 0.136 | 3.8667 | 12446.31 | 13200.47 | 0.111 | 0.113 | 5.7131 |
| 867 | 12326.37 | 13349.23 | 0.139 | 0.139 | 7.6623 | 12128.21 | 12206.71 | 0.127 | 0.13 | 0.643 |
| 868 | 11117.16 | 13504.2 | 0.139 | 0.137 | 17.676 | 12596.14 | 13133.89 | 0.124 | 0.118 | 4.0943 |
| 869 | 11500.83 | 12352.69 | 0.139 | 0.141 | 6.8961 | 11687.33 | 11624.61 | 0.091 | 0.092 | -0.5396 |
| 870 | 11102.2 | 11345.87 | 0.139 | 0.138 | 2.1477 | 11897.77 | 11926.77 | 0.092 | 0.089 | 0.2431 |
| 871 | 10994.38 | 12158.37 | 0.139 | 0.139 | 9.5736 | 10375.1 | 11825.94 | 0.081 | 0.074 | 12.268 |
| 872 | 10984.41 | 12003.6 | 0.139 | 0.139 | 8.4907 | 13334.59 | 13431.15 | 0.071 | 0.075 | 0.719 |
| 873 | 11150.59 | 11375.69 | 0.139 | 0.143 | 1.9788 | 11252.39 | 11311.8 | 0.091 | 0.093 | 0.5252 |
| 874 | 12404.92 | 12779.22 | 0.139 | 0.137 | 2.929 | 12525.73 | 13087.9 | 0.121 | 0.12 | 4.2953 |
| 875 | 11175.85 | 11424.38 | 0.14 | 0.14 | 2.1754 | 14556.94 | 14254.93 | 0.081 | 0.085 | -2.1186 |
| 876 | 10942.86 | 11473.07 | 0.14 | 0.143 | 4.6213 | 10112.32 | 10296.76 | 0.123 | 0.126 | 1.7912 |
| 877 | 11446.09 | 12477.01 | 0.14 | 0.137 | 8.2625 | 14888.17 | 14916.67 | 0.083 | 0.084 | 0.191 |
| 878 | 10853.11 | 11616.06 | 0.14 | 0.14 | 6.5681 | 11036.29 | 11050.61 | 0.117 | 0.116 | 0.1295 |
| 879 | 11803.71 | 12793.07 | 0.14 | 0.137 | 7.7336 | 15497.5 | 15559.13 | 0.084 | 0.086 | 0.3961 |
| 880 | 11729.36 | 12887.87 | 0.14 | 0.139 | 8.9892 | 15584.87 | 15104.78 | 0.085 | 0.088 | -3.1784 |
| 881 | 11849.71 | 13497.13 | 0.14 | 0.139 | 12.206 | 11238.34 | 11513.33 | 0.112 | 0.118 | 2.3884 |
| 882 | 11270.74 | 12184.45 | 0.14 | 0.139 | 7.499 | 16521.19 | 16674.08 | 0.111 | 0.112 | 0.917 |
| 883 | 12592.36 | 13216.62 | 0.14 | 0.138 | 4.7233 | 12566.71 | 13039.38 | 0.097 | 0.103 | 3.6249 |
| 884 | 11339.88 | 12365.68 | 0.14 | 0.14 | 8.2955 | 15628.64 | 16097.09 | 0.12 | 0.116 | 2.9102 |
| 885 | 11947.19 | 12362.91 | 0.14 | 0.137 | 3.3626 | 12437.34 | 12574.71 | 0.121 | 0.121 | 1.0924 |
| 886 | 11721.45 | 12095.75 | 0.14 | 0.138 | 3.0945 | 11077.79 | 11242.26 | 0.112 | 0.111 | 1.463 |
| 887 | 11926.89 | 12929.81 | 0.14 | 0.139 | 7.7566 | 10398.95 | 10814.69 | 0.131 | 0.121 | 3.8442 |
| 888 | 10709.91 | 11695.75 | 0.14 | 0.14 | 8.4291 | 16582.58 | 17036.98 | 0.113 | 0.113 | 2.6671 |
| 889 | 11858.44 | 12106.26 | 0.14 | 0.138 | 2.047 | 10214.75 | 10637.92 | 0.111 | 0.116 | 3.978 |
| 890 | 10472.69 | 11679.94 | 0.14 | 0.141 | 10.336 | 13244.38 | 14290.1 | 0.091 | 0.077 | 7.3178 |
| 891 | 10667.13 | 11621.41 | 0.14 | 0.14 | 8.2114 | 11683.01 | 11648.36 | 0.118 | 0.123 | -0.2974 |
| 892 | 12068.23 | 12660.86 | 0.14 | 0.14 | 4.6808 | 15439.61 | 15976.47 | 0.112 | 0.119 | 3.3603 |
| 893 | 11468.23 | 11807.17 | 0.14 | 0.138 | 2.8707 | 10682 | 10853.21 | 0.07 | 0.088 | 1.5775 |
| 894 | 11332.76 | 11576.44 | 0.14 | 0.139 | 2.1049 | 11062.44 | 12186.08 | 0.069 | 0.066 | 9.2207 |
| 895 | 10959.53 | 11559.22 | 0.14 | 0.139 | 5.188 | 14130.98 | 13922.91 | 0.107 | 0.112 | -1.4945 |
| 896 | 10761.1 | 12300.03 | 0.14 | 0.139 | 12.512 | 15825.84 | 15743.33 | 0.085 | 0.085 | -0.5241 |
| 897 | 12034.08 | 12626.72 | 0.14 | 0.14 | 4.6935 | 15796.98 | 16691.09 | 0.112 | 0.11 | 5.3568 |
| 898 | 11673.41 | 11917.09 | 0.14 | 0.139 | 2.0448 | 15420.39 | 15497.59 | 0.1 | 0.105 | 0.4981 |
| 899 | 11430.2 | 12238.09 | 0.14 | 0.139 | 6.6014 | 15756.16 | 15785.78 | 0.105 | 0.11 | 0.1876 |

| 900 | 10269.55 | 11224.51 | 0.14 | 0.143 | 8.5078 | 15861.49 | 15241.7 | 0.101 | 0.109 | -4.0664 |
| --- | --- | --- | --- | --- | --- | --- | --- | --- | --- | --- |
| 901 | 12463.58 | 12980.48 | 0.14 | 0.136 | 3.9821 | 11480.82 | 11757.51 | 0.108 | 0.113 | 2.3533 |
| 902 | 10885.41 | 11776.06 | 0.14 | 0.141 | 7.5632 | 14970.33 | 14938.62 | 0.086 | 0.082 | -0.2123 |
| 903 | 12566.8 | 13061.28 | 0.14 | 0.138 | 3.7858 | 17376.03 | 17806.77 | 0.099 | 0.103 | 2.419 |
| 904 | 10942.52 | 12003.39 | 0.14 | 0.14 | 8.8381 | 12165.52 | 12169.37 | 0.097 | 0.101 | 0.0316 |
| 905 | 11264.11 | 12192.47 | 0.14 | 0.14 | 7.6142 | 12245.21 | 11898.73 | 0.083 | 0.091 | -2.9119 |
| 906 | 12646.11 | 13442.92 | 0.14 | 0.137 | 5.9274 | 14125.5 | 14227.55 | 0.111 | 0.115 | 0.7172 |
| 907 | 11470.61 | 11714.28 | 0.141 | 0.139 | 2.0802 | 13820.92 | 13702.64 | 0.114 | 0.113 | -0.8632 |
| 908 | 11379.8 | 12610.74 | 0.141 | 0.142 | 9.761 | 12300.11 | 12390.82 | 0.078 | 0.082 | 0.7321 |
| 909 | 11641.18 | 12621.88 | 0.141 | 0.139 | 7.7698 | 12261.33 | 12363.37 | 0.113 | 0.118 | 0.8254 |
| 910 | 11259.85 | 12196.02 | 0.141 | 0.14 | 7.6761 | 10501.73 | 10584.75 | 0.121 | 0.125 | 0.7843 |
| 911 | 10940.89 | 11961.83 | 0.141 | 0.139 | 8.535 | 11162.03 | 11057.09 | 0.105 | 0.113 | -0.9491 |
| 912 | 11523.49 | 12912.58 | 0.141 | 0.138 | 10.758 | 15032.08 | 15466.47 | 0.101 | 0.105 | 2.8086 |
| 913 | 10983.4 | 12195.61 | 0.141 | 0.14 | 9.9397 | 13272.37 | 14604.03 | 0.065 | 0.067 | 9.1185 |
| 914 | 12273.41 | 13182.86 | 0.141 | 0.137 | 6.8987 | 14042.76 | 14028.82 | 0.108 | 0.112 | -0.0993 |
| 915 | 11554.56 | 11833.6 | 0.141 | 0.139 | 2.358 | 11622.53 | 11612.74 | 0.096 | 0.1 | -0.0843 |
| 916 | 11200.3 | 11801 | 0.141 | 0.14 | 5.0902 | 10691.86 | 10752.75 | 0.128 | 0.129 | 0.5662 |
| 917 | 12900.05 | 13609.67 | 0.141 | 0.138 | 5.2141 | 11155.85 | 11880.29 | 0.109 | 0.114 | 6.0978 |
| 918 | 11820.15 | 12938.23 | 0.141 | 0.139 | 8.6416 | 12027.11 | 12049.87 | 0.107 | 0.116 | 0.1889 |
| 919 | 11288.7 | 12184.19 | 0.141 | 0.141 | 7.3497 | 14939.83 | 15486.94 | 0.095 | 0.107 | 3.5327 |
| 920 | 10340.62 | 11281.18 | 0.141 | 0.141 | 8.3374 | 12077.31 | 12077.31 | 0.08 | 0.08 | 0 |
| 921 | 10995.77 | 12045.84 | 0.141 | 0.14 | 8.7173 | 15582.29 | 15659.48 | 0.104 | 0.109 | 0.4929 |
| 922 | 10170.62 | 11339.72 | 0.141 | 0.141 | 10.31 | 17202.23 | 17824.62 | 0.11 | 0.106 | 3.4917 |
| 923 | 11066.33 | 11440.63 | 0.141 | 0.139 | 3.2717 | 15575.61 | 15919.55 | 0.111 | 0.117 | 2.1605 |
| 924 | 11965.82 | 13619.05 | 0.141 | 0.139 | 12.139 | 14956 | 15637.39 | 0.113 | 0.111 | 4.3574 |
| 925 | 10744.73 | 11831.98 | 0.141 | 0.14 | 9.1891 | 14130.01 | 14430.51 | 0.119 | 0.12 | 2.0824 |
| 926 | 12758.63 | 13183.81 | 0.141 | 0.138 | 3.225 | 12409.59 | 12342.22 | 0.086 | 0.086 | -0.5458 |
| 927 | 10340.41 | 10811.9 | 0.141 | 0.14 | 4.3609 | 15128.53 | 14237.32 | 0.095 | 0.075 | -6.2597 |
| 928 | 11501.28 | 12508.8 | 0.141 | 0.138 | 8.0545 | 15622.44 | 16129.25 | 0.099 | 0.109 | 3.1422 |
| 929 | 11678.83 | 11922.5 | 0.141 | 0.14 | 2.0438 | 15899.15 | 16323.33 | 0.097 | 0.103 | 2.5986 |
| 930 | 11952.35 | 12785.22 | 0.141 | 0.137 | 6.5144 | 11361.76 | 12245.35 | 0.099 | 0.11 | 7.2157 |
| 931 | 12622.98 | 13041.3 | 0.141 | 0.138 | 3.2077 | 13319.71 | 13703.68 | 0.104 | 0.109 | 2.8019 |
| 932 | 11130.36 | 11409.39 | 0.141 | 0.14 | 2.4456 | 11406.74 | 11599.67 | 0.128 | 0.127 | 1.6632 |
| 933 | 10859.11 | 11382.53 | 0.142 | 0.14 | 4.5984 | 12783.16 | 13430.24 | 0.11 | 0.116 | 4.818 |
| 934 | 11151.75 | 12088.18 | 0.142 | 0.141 | 7.7467 | 13255.92 | 13320.06 | 0.068 | 0.073 | 0.4815 |
| 935 | 11419.32 | 11902.03 | 0.142 | 0.14 | 4.0557 | 11176.34 | 11223.12 | 0.121 | 0.122 | 0.4168 |
| 936 | 11800.59 | 12508.58 | 0.142 | 0.14 | 5.66 | 12258.36 | 13195.21 | 0.096 | 0.083 | 7.0999 |
| 937 | 11266.37 | 11545.4 | 0.142 | 0.14 | 2.4168 | 12718.81 | 12628.61 | 0.108 | 0.104 | -0.7143 |
| 938 | 11697.49 | 12202.8 | 0.142 | 0.14 | 4.1409 | 11288.93 | 11777.5 | 0.133 | 0.128 | 4.1484 |
| 939 | 11126.83 | 11501.13 | 0.142 | 0.139 | 3.2545 | 10428.46 | 10688.91 | 0.125 | 0.124 | 2.4367 |
| 940 | 11633.9 | 12822.42 | 0.142 | 0.139 | 9.269 | 11374.76 | 11503.54 | 0.086 | 0.092 | 1.1195 |
| 941 | 12376.27 | 12784.08 | 0.142 | 0.139 | 3.19 | 11694.87 | 11714.37 | 0.077 | 0.078 | 0.1664 |
| 942 | 12040.58 | 12510.91 | 0.142 | 0.14 | 3.7594 | 11451.85 | 11553.9 | 0.115 | 0.121 | 0.8832 |
| 943 | 11122.84 | 12018.34 | 0.142 | 0.142 | 7.4511 | 11960.17 | 11937.45 | 0.08 | 0.081 | -0.1903 |
| 944 | 11667.69 | 12818.48 | 0.142 | 0.136 | 8.9776 | 15973.65 | 16417.03 | 0.091 | 0.105 | 2.7007 |
| 945 | 11928.6 | 12398.93 | 0.142 | 0.14 | 3.7933 | 11820.26 | 11882.39 | 0.085 | 0.082 | 0.5229 |
| 946 | 11842 | 12462.63 | 0.142 | 0.14 | 4.9799 | 10305.3 | 10413 | 0.131 | 0.132 | 1.0342 |
| 947 | 11064.11 | 11438.41 | 0.142 | 0.14 | 3.2723 | 14846.44 | 15007.62 | 0.113 | 0.114 | 1.074 |
| 948 | 11418.99 | 11793.29 | 0.142 | 0.14 | 3.1738 | 10058.6 | 10351.94 | 0.126 | 0.126 | 2.8337 |
| 949 | 10814.65 | 11093.68 | 0.142 | 0.14 | 2.5152 | 10451.64 | 10684.69 | 0.117 | 0.115 | 2.1812 |
| 950 | 11345.06 | 12233.86 | 0.142 | 0.142 | 7.2651 | 12759.84 | 13784.06 | 0.094 | 0.08 | 7.4304 |

| 951 | 11610.99 | 11890.02 | 0.142 | 0.14 | 2.3468 | 11800.05 | 12722.18 | 0.074 | 0.074 | 7.2482 |
| --- | --- | --- | --- | --- | --- | --- | --- | --- | --- | --- |
| 952 | 10730 | 11617.35 | 0.142 | 0.141 | 7.6382 | 13752.11 | 13697.67 | 0.089 | 0.09 | -0.3974 |
| 953 | 11106.72 | 11556.46 | 0.142 | 0.14 | 3.8917 | 11959.66 | 13032.58 | 0.098 | 0.084 | 8.2326 |
| 954 | 11956.22 | 12299.31 | 0.142 | 0.14 | 2.7895 | 12398.31 | 12335.59 | 0.096 | 0.097 | -0.5085 |
| 955 | 11517.33 | 12626.15 | 0.142 | 0.138 | 8.7819 | 15533.77 | 15402.77 | 0.084 | 0.084 | -0.8505 |
| 956 | 11018.58 | 11269.33 | 0.142 | 0.141 | 2.225 | 12538.57 | 12477.36 | 0.115 | 0.115 | -0.4906 |
| 957 | 11581.07 | 11956.59 | 0.142 | 0.141 | 3.1406 | 15506.73 | 15643.63 | 0.105 | 0.109 | 0.8751 |
| 958 | 10769.76 | 11735.97 | 0.142 | 0.141 | 8.2329 | 11683.2 | 11886.46 | 0.102 | 0.108 | 1.71 |
| 959 | 10841.12 | 11642.56 | 0.142 | 0.142 | 6.8837 | 10716 | 10774.99 | 0.116 | 0.114 | 0.5475 |
| 960 | 10988.9 | 12400.59 | 0.143 | 0.142 | 11.384 | 12885.43 | 13558.74 | 0.118 | 0.113 | 4.9659 |
| 961 | 12346.84 | 13296.71 | 0.143 | 0.138 | 7.1436 | 12760.25 | 12812 | 0.107 | 0.108 | 0.4039 |
| 962 | 11699.34 | 12107.16 | 0.143 | 0.139 | 3.3684 | 12617.93 | 12597.81 | 0.084 | 0.092 | -0.1597 |
| 963 | 12973.72 | 13669.91 | 0.143 | 0.138 | 5.0929 | 12402.33 | 12511.92 | 0.086 | 0.095 | 0.8758 |
| 964 | 10902.57 | 11766.85 | 0.143 | 0.143 | 7.3451 | 12026.24 | 11770.55 | 0.124 | 0.125 | -2.1722 |
| 965 | 11120.98 | 12148.9 | 0.143 | 0.141 | 8.4611 | 15666.79 | 15619.22 | 0.082 | 0.084 | -0.3046 |
| 966 | 11585.86 | 12008.53 | 0.143 | 0.14 | 3.5197 | 10281.37 | 10461.19 | 0.118 | 0.116 | 1.719 |
| 967 | 11768.29 | 12036.82 | 0.143 | 0.14 | 2.2309 | 14413.68 | 14504.39 | 0.071 | 0.075 | 0.6254 |
| 968 | 11125.62 | 11935.35 | 0.143 | 0.143 | 6.7843 | 15781.08 | 16390.02 | 0.1 | 0.117 | 3.7153 |
| 969 | 10386.39 | 10847.14 | 0.143 | 0.14 | 4.2476 | 10959.83 | 10970.87 | 0.107 | 0.117 | 0.1006 |
| 970 | 11924.63 | 12369.22 | 0.143 | 0.141 | 3.5944 | 12490.59 | 13027.19 | 0.105 | 0.109 | 4.1191 |
| 971 | 10944.85 | 11458.77 | 0.143 | 0.141 | 4.4849 | 12119.82 | 12479.23 | 0.086 | 0.086 | 2.8801 |
| 972 | 11158.49 | 12277.54 | 0.144 | 0.141 | 9.1146 | 14064.2 | 14799.06 | 0.115 | 0.121 | 4.9655 |
| 973 | 11126.57 | 11708.03 | 0.144 | 0.141 | 4.9663 | 16381.7 | 16655.59 | 0.098 | 0.104 | 1.6444 |
| 974 | 11350.83 | 11725.13 | 0.144 | 0.141 | 3.1923 | 11893.28 | 13645.53 | 0.082 | 0.074 | 12.841 |
| 975 | 11161.37 | 11977.09 | 0.144 | 0.141 | 6.8107 | 16135.88 | 16348.6 | 0.116 | 0.117 | 1.3012 |
| 976 | 11393.65 | 12106.61 | 0.144 | 0.139 | 5.8891 | 12191.64 | 12819.39 | 0.121 | 0.116 | 4.8968 |
| 977 | 11134.47 | 11473.41 | 0.144 | 0.142 | 2.9542 | 14554.39 | 14835.22 | 0.104 | 0.109 | 1.893 |
| 978 | 11942.31 | 12495.81 | 0.144 | 0.142 | 4.4295 | 17224.16 | 17122.66 | 0.099 | 0.103 | -0.5928 |
| 979 | 10959.86 | 11944.16 | 0.144 | 0.141 | 8.2408 | 12237.81 | 12175.09 | 0.092 | 0.092 | -0.5152 |
| 980 | 11341.51 | 11630.04 | 0.144 | 0.14 | 2.4809 | 12519.78 | 13063.89 | 0.122 | 0.117 | 4.165 |
| 981 | 10817.16 | 11382.26 | 0.144 | 0.141 | 4.9647 | 15338.53 | 15716.26 | 0.107 | 0.118 | 2.4034 |
| 982 | 11150.06 | 11648.62 | 0.144 | 0.143 | 4.28 | 11796.79 | 14236.69 | 0.073 | 0.073 | 17.138 |
| 983 | 10576.63 | 11100.04 | 0.144 | 0.142 | 4.7154 | 13295.8 | 13677.46 | 0.098 | 0.106 | 2.7905 |
| 984 | 11221.45 | 11596.97 | 0.144 | 0.142 | 3.238 | 10857.56 | 10940.58 | 0.122 | 0.126 | 0.7588 |
| 985 | 10889.82 | 11229.77 | 0.144 | 0.142 | 3.0272 | 12079.5 | 12580.22 | 0.107 | 0.109 | 3.9803 |
| 986 | 10918.44 | 11118.18 | 0.144 | 0.142 | 1.7965 | 16338.62 | 15307.14 | 0.097 | 0.073 | -6.7385 |
| 987 | 10831.5 | 11740.81 | 0.144 | 0.143 | 7.7449 | 14410.58 | 14359.07 | 0.09 | 0.09 | -0.3587 |
| 988 | 11463.9 | 11838.2 | 0.144 | 0.142 | 3.1618 | 11599.33 | 11676.73 | 0.11 | 0.119 | 0.6629 |
| 989 | 11656.39 | 12361.14 | 0.144 | 0.14 | 5.7013 | 11600.76 | 11613.19 | 0.084 | 0.084 | 0.107 |
| 990 | 10999.03 | 11398.77 | 0.144 | 0.141 | 3.5069 | 11173.56 | 11380.25 | 0.115 | 0.116 | 1.8162 |
| 991 | 10985.39 | 11828.47 | 0.144 | 0.141 | 7.1276 | 10913.52 | 11371.26 | 0.121 | 0.12 | 4.0253 |
| 992 | 11616.37 | 12273.09 | 0.144 | 0.141 | 5.3509 | 11938.31 | 12291.78 | 0.106 | 0.111 | 2.8756 |
| 993 | 9905.793 | 10412.64 | 0.145 | 0.142 | 4.8676 | 10988.7 | 11074.02 | 0.125 | 0.127 | 0.7704 |
| 994 | 11057.52 | 11503.03 | 0.145 | 0.143 | 3.873 | 12319.23 | 12296.51 | 0.089 | 0.089 | -0.1848 |
| 995 | 11138.38 | 12117.32 | 0.145 | 0.142 | 8.0789 | 17245.76 | 17617.01 | 0.099 | 0.102 | 2.1073 |
| 996 | 10096.94 | 10603.79 | 0.145 | 0.142 | 4.7799 | 11965.64 | 12079.78 | 0.084 | 0.084 | 0.9449 |
| 997 | 10555.24 | 11046.4 | 0.145 | 0.141 | 4.4464 | 9656.981 | 9969.859 | 0.125 | 0.123 | 3.1382 |
| 998 | 10985.5 | 11511.84 | 0.145 | 0.143 | 4.5722 | 11279.39 | 11258.68 | 0.119 | 0.117 | -0.184 |
| 999 | 10997.9 | 11526.76 | 0.145 | 0.141 | 4.5881 | 12116.83 | 12089.97 | 0.087 | 0.088 | -0.2222 |
| 1000 | 9995.584 | 10338.67 | 0.147 | 0.145 | 3.3185 | 14180.83 | 14035.98 | 0.115 | 0.114 | -1.032 |

|  | Daraga sample unite (3) N-S | | | | | Wuqro sample unite (4) N-S | | | | |
| --- | --- | --- | --- | --- | --- | --- | --- | --- | --- | --- |
|  | UM L | M L | UM C | M C | L Ch % | UM L | M L | UM C | M C | L Ch % |
| 1 | 11734.12 | 12194.07 | 0.149 | 0.147 | 3.7719 | 11322.5 | 11363.21 | 0.125 | 0.122 | 0.3583 |
| 2 | 13153.53 | 13222.94 | 0.14 | 0.14 | 0.5249 | 10794.69 | 10924.27 | 0.124 | 0.123 | 1.1862 |
| 3 | 12463.04 | 12530.41 | 0.138 | 0.137 | 0.5376 | 11964.24 | 12089.01 | 0.124 | 0.124 | 1.0321 |
| 4 | 14012.87 | 16096.76 | 0.134 | 0.135 | 12.946 | 14898.07 | 15047.66 | 0.129 | 0.128 | 0.9941 |
| 5 | 14612.57 | 14876.68 | 0.121 | 0.127 | 1.7753 | 11916.77 | 12476.51 | 0.13 | 0.129 | 4.4864 |
| 6 | 15328.17 | 15617.67 | 0.131 | 0.126 | 1.8537 | 11798.4 | 11989.44 | 0.127 | 0.126 | 1.5934 |
| 7 | 11564.84 | 13000.79 | 0.118 | 0.12 | 11.045 | 12730.63 | 12981.96 | 0.128 | 0.129 | 1.936 |
| 8 | 11601.9 | 11934.42 | 0.121 | 0.124 | 2.7862 | 12146.52 | 12048.53 | 0.125 | 0.123 | -0.813 |
| 9 | 12069.96 | 12819.19 | 0.146 | 0.147 | 5.8447 | 11258.86 | 11377.06 | 0.127 | 0.128 | 1.0389 |
| 10 | 12656.18 | 12633.75 | 0.14 | 0.142 | -0.178 | 11206.33 | 11302.6 | 0.12 | 0.121 | 0.8518 |
| 11 | 12033.66 | 13761.04 | 0.12 | 0.139 | 12.553 | 11847.64 | 13522.22 | 0.14 | 0.139 | 12.384 |
| 12 | 11153.62 | 11992.95 | 0.144 | 0.145 | 6.9985 | 11587.87 | 12113.93 | 0.122 | 0.122 | 4.3425 |
| 13 | 11452.42 | 15615.42 | 0.143 | 0.14 | 26.66 | 12093.4 | 12238.21 | 0.122 | 0.118 | 1.1833 |
| 14 | 12514.28 | 12400.61 | 0.115 | 0.123 | -0.917 | 12689.84 | 12782.69 | 0.13 | 0.129 | 0.7263 |
| 15 | 12254.87 | 13149.22 | 0.114 | 0.122 | 6.8015 | 11293.95 | 11499.72 | 0.139 | 0.136 | 1.7894 |
| 16 | 10859 | 11158.15 | 0.124 | 0.127 | 2.681 | 12327.48 | 12633.79 | 0.13 | 0.13 | 2.4245 |
| 17 | 11804.75 | 12199.05 | 0.148 | 0.146 | 3.2322 | 12635.29 | 12738.21 | 0.124 | 0.121 | 0.808 |
| 18 | 13873.2 | 13967.56 | 0.128 | 0.133 | 0.6756 | 13866.81 | 14005.69 | 0.118 | 0.118 | 0.9915 |
| 19 | 10665.21 | 14303.64 | 0.125 | 0.141 | 25.437 | 12002.03 | 12365.29 | 0.124 | 0.122 | 2.9377 |
| 20 | 11678.41 | 11757.11 | 0.131 | 0.129 | 0.6694 | 14220.53 | 14001.53 | 0.112 | 0.117 | -1.564 |
| 21 | 15187.62 | 14843.11 | 0.108 | 0.111 | -2.321 | 15619.65 | 15726.81 | 0.128 | 0.127 | 0.6814 |
| 22 | 12887.12 | 12422.86 | 0.107 | 0.118 | -3.737 | 12122.02 | 12113.24 | 0.129 | 0.129 | -0.073 |
| 23 | 11858.84 | 11902.77 | 0.142 | 0.142 | 0.3691 | 14123.41 | 14311.47 | 0.128 | 0.121 | 1.3141 |
| 24 | 11038.18 | 10971.7 | 0.117 | 0.124 | -0.606 | 10577.26 | 10766.88 | 0.127 | 0.122 | 1.7611 |
| 25 | 12814.46 | 12838.31 | 0.14 | 0.141 | 0.1858 | 13541.37 | 13584.09 | 0.122 | 0.123 | 0.3145 |
| 26 | 14716.63 | 17048.1 | 0.127 | 0.134 | 13.676 | 16098.59 | 17608.64 | 0.134 | 0.137 | 8.5756 |
| 27 | 11934.14 | 13029.71 | 0.135 | 0.134 | 8.4082 | 12290.35 | 12567.45 | 0.118 | 0.122 | 2.205 |
| 28 | 11998.78 | 13283.25 | 0.143 | 0.148 | 9.6699 | 15335.51 | 15596.39 | 0.128 | 0.128 | 1.6727 |
| 29 | 13985.31 | 14002.88 | 0.139 | 0.139 | 0.1255 | 14025.11 | 13946.16 | 0.123 | 0.122 | -0.566 |
| 30 | 11502.29 | 12430.64 | 0.127 | 0.128 | 7.4683 | 11023.21 | 11302.42 | 0.135 | 0.134 | 2.4703 |
| 31 | 12939.91 | 13606.3 | 0.146 | 0.147 | 4.8977 | 11095.51 | 11318.47 | 0.131 | 0.13 | 1.9699 |
| 32 | 11321.71 | 11903.93 | 0.139 | 0.136 | 4.891 | 12751.44 | 12776.59 | 0.125 | 0.126 | 0.1968 |
| 33 | 12513.81 | 12025.49 | 0.102 | 0.118 | -4.061 | 10248.85 | 11453.76 | 0.137 | 0.134 | 10.52 |
| 34 | 15312.1 | 15491.77 | 0.124 | 0.13 | 1.1598 | 10942.26 | 11264.43 | 0.12 | 0.127 | 2.86 |
| 35 | 13486.02 | 14227.18 | 0.117 | 0.13 | 5.2095 | 12359.44 | 12434.79 | 0.124 | 0.124 | 0.606 |
| 36 | 14272.39 | 14805.94 | 0.132 | 0.135 | 3.6036 | 12295.05 | 12791.31 | 0.129 | 0.124 | 3.8797 |
| 37 | 12812.69 | 12879.64 | 0.112 | 0.121 | 0.5198 | 15486.9 | 16436.74 | 0.119 | 0.124 | 5.7788 |
| 38 | 14310.7 | 15269.66 | 0.128 | 0.127 | 6.2802 | 13279.26 | 13221.06 | 0.119 | 0.113 | -0.44 |
| 39 | 11828.54 | 11886.53 | 0.141 | 0.143 | 0.4879 | 11505.59 | 11615.59 | 0.136 | 0.134 | 0.947 |
| 40 | 12214.82 | 12280.88 | 0.138 | 0.14 | 0.538 | 13971.77 | 14322.24 | 0.129 | 0.127 | 2.447 |
| 41 | 12906.3 | 13172.66 | 0.136 | 0.134 | 2.0221 | 12121.88 | 12372.12 | 0.131 | 0.128 | 2.0226 |
| 42 | 11945.16 | 13658.91 | 0.123 | 0.121 | 12.547 | 15999.42 | 16132.94 | 0.128 | 0.127 | 0.8276 |
| 43 | 14442.68 | 14572.98 | 0.137 | 0.14 | 0.8941 | 13161.79 | 13226.85 | 0.118 | 0.119 | 0.4919 |
| 44 | 13071.2 | 13414.01 | 0.131 | 0.138 | 2.5556 | 10692.41 | 10747.76 | 0.129 | 0.128 | 0.515 |
| 45 | 14511.96 | 14747.02 | 0.13 | 0.128 | 1.594 | 12324.09 | 12538.11 | 0.121 | 0.118 | 1.707 |
| 46 | 12802.65 | 13346.74 | 0.135 | 0.133 | 4.0766 | 11306.07 | 11297.79 | 0.125 | 0.125 | -0.073 |
| 47 | 12944.35 | 12912.56 | 0.119 | 0.119 | -0.246 | 12101.71 | 12217.06 | 0.12 | 0.117 | 0.9442 |
| 48 | 11428.91 | 12066.27 | 0.125 | 0.121 | 5.2821 | 13078.51 | 13143.57 | 0.121 | 0.121 | 0.495 |

| 49 | 11402.34 | 12109.56 | 0.146 | 0.143 | 5.8402 | 13038.16 | 13189.46 | 0.124 | 0.123 | 1.1471 |
| --- | --- | --- | --- | --- | --- | --- | --- | --- | --- | --- |
| 50 | 11975.09 | 11979.23 | 0.143 | 0.143 | 0.0346 | 16754.33 | 16864.42 | 0.127 | 0.127 | 0.6528 |
| 51 | 13739.38 | 13432.18 | 0.106 | 0.113 | -2.287 | 14630.74 | 14891.12 | 0.131 | 0.13 | 1.7486 |
| 52 | 10829.21 | 11179.45 | 0.137 | 0.132 | 3.1329 | 12484.97 | 12894.41 | 0.12 | 0.123 | 3.1754 |
| 53 | 11316.38 | 11761.85 | 0.136 | 0.14 | 3.7875 | 10565.53 | 11541.74 | 0.135 | 0.138 | 8.4581 |
| 54 | 12605.7 | 12683.86 | 0.112 | 0.12 | 0.6162 | 10275.28 | 11509.92 | 0.133 | 0.134 | 10.727 |
| 55 | 11712.63 | 12239.37 | 0.108 | 0.121 | 4.3037 | 12205.11 | 12304.61 | 0.125 | 0.12 | 0.8086 |
| 56 | 14425.88 | 14839.52 | 0.131 | 0.136 | 2.7874 | 13322.31 | 13409.97 | 0.116 | 0.113 | 0.6537 |
| 57 | 12260.31 | 12922.56 | 0.148 | 0.148 | 5.1248 | 12584.81 | 12597.12 | 0.126 | 0.123 | 0.0977 |
| 58 | 10787.73 | 12123.28 | 0.136 | 0.133 | 11.016 | 13527.68 | 13429.69 | 0.123 | 0.121 | -0.73 |
| 59 | 11700.52 | 11927.17 | 0.117 | 0.12 | 1.9003 | 13007.51 | 13142.25 | 0.12 | 0.12 | 1.0252 |
| 60 | 11626.59 | 12544.32 | 0.134 | 0.132 | 7.3159 | 11970.33 | 12423.47 | 0.126 | 0.126 | 3.6474 |
| 61 | 12444.08 | 14674.29 | 0.14 | 0.145 | 15.198 | 13423.97 | 13527.11 | 0.116 | 0.119 | 0.7624 |
| 62 | 13872.46 | 14049.53 | 0.134 | 0.13 | 1.2603 | 14555.29 | 15808.06 | 0.131 | 0.131 | 7.9249 |
| 63 | 10895.28 | 11334.72 | 0.13 | 0.125 | 3.877 | 11980.71 | 11962.93 | 0.123 | 0.121 | -0.149 |
| 64 | 13054.08 | 16030.9 | 0.112 | 0.109 | 18.569 | 11358.07 | 11492.8 | 0.129 | 0.129 | 1.1723 |
| 65 | 14982.35 | 14971.68 | 0.127 | 0.135 | -0.071 | 14272.62 | 15678.74 | 0.126 | 0.129 | 8.9683 |
| 66 | 12807.93 | 12765.8 | 0.142 | 0.142 | -0.33 | 12584.73 | 12775.77 | 0.123 | 0.122 | 1.4953 |
| 67 | 11358.24 | 11712.17 | 0.138 | 0.135 | 3.0219 | 11382.62 | 11447.68 | 0.123 | 0.124 | 0.5683 |
| 68 | 13220.34 | 13224.48 | 0.138 | 0.139 | 0.0313 | 12953.91 | 13092.78 | 0.121 | 0.12 | 1.0607 |
| 69 | 10745.77 | 10903.67 | 0.147 | 0.147 | 1.4482 | 15211.93 | 15453.53 | 0.124 | 0.123 | 1.5634 |
| 70 | 10300.14 | 10581.1 | 0.147 | 0.145 | 2.6552 | 12092.15 | 12210.35 | 0.124 | 0.124 | 0.968 |
| 71 | 11517.23 | 11556.23 | 0.144 | 0.144 | 0.3374 | 11461.27 | 11777.87 | 0.131 | 0.127 | 2.6881 |
| 72 | 10792.94 | 11099.96 | 0.149 | 0.146 | 2.766 | 13478.66 | 13253.13 | 0.114 | 0.113 | -1.702 |
| 73 | 12279.33 | 13808.69 | 0.128 | 0.124 | 11.075 | 13022.12 | 13293.63 | 0.134 | 0.135 | 2.0424 |
| 74 | 11671.34 | 12192 | 0.145 | 0.146 | 4.2705 | 12152.27 | 12270.47 | 0.123 | 0.123 | 0.9633 |
| 75 | 13513.1 | 13596.78 | 0.133 | 0.14 | 0.6154 | 11399.12 | 12006.14 | 0.128 | 0.131 | 5.0559 |
| 76 | 10828.61 | 11201.7 | 0.146 | 0.145 | 3.3306 | 10846.61 | 11016.52 | 0.138 | 0.139 | 1.5424 |
| 77 | 10937.64 | 11037.05 | 0.141 | 0.144 | 0.9007 | 11205.57 | 12908.23 | 0.139 | 0.137 | 13.19 |
| 78 | 11056.22 | 11382.74 | 0.126 | 0.127 | 2.8685 | 13782.21 | 12889.92 | 0.115 | 0.117 | -6.922 |
| 79 | 12968.25 | 13170.68 | 0.136 | 0.134 | 1.5369 | 13326.32 | 13401.76 | 0.119 | 0.121 | 0.5629 |
| 80 | 11956.29 | 11956.29 | 0.139 | 0.139 | 0 | 11564 | 11537.93 | 0.121 | 0.119 | -0.226 |
| 81 | 11245.73 | 11534.97 | 0.146 | 0.144 | 2.5075 | 11801.95 | 12015.21 | 0.129 | 0.127 | 1.7749 |
| 82 | 13309.85 | 13371.9 | 0.131 | 0.137 | 0.464 | 13450.22 | 13546.49 | 0.123 | 0.125 | 0.7107 |
| 83 | 11795.14 | 13704.67 | 0.143 | 0.148 | 13.933 | 10924.04 | 11166.29 | 0.122 | 0.126 | 2.1695 |
| 84 | 13872.82 | 13889.39 | 0.134 | 0.133 | 0.1193 | 13749.74 | 13824.6 | 0.12 | 0.116 | 0.5414 |
| 85 | 12008.75 | 13628.25 | 0.143 | 0.146 | 11.883 | 12936.09 | 12975.59 | 0.128 | 0.127 | 0.3044 |
| 86 | 11705.37 | 13989.79 | 0.124 | 0.142 | 16.329 | 12731.37 | 12890.96 | 0.118 | 0.119 | 1.238 |
| 87 | 12847.66 | 14048.2 | 0.121 | 0.114 | 8.5459 | 11816.14 | 11569.16 | 0.123 | 0.122 | -2.135 |
| 88 | 12918.85 | 14529.01 | 0.122 | 0.111 | 11.082 | 12877.32 | 12794.89 | 0.123 | 0.121 | -0.644 |
| 89 | 14560.31 | 14917.5 | 0.13 | 0.126 | 2.3945 | 13386.86 | 13582.64 | 0.138 | 0.137 | 1.4413 |
| 90 | 12204.77 | 13127.75 | 0.125 | 0.128 | 7.0308 | 11269.4 | 11408.28 | 0.128 | 0.127 | 1.2173 |
| 91 | 12357.1 | 13264.13 | 0.129 | 0.139 | 6.8383 | 11821.24 | 11931.24 | 0.135 | 0.133 | 0.9219 |
| 92 | 11447.55 | 11658.51 | 0.132 | 0.13 | 1.8094 | 13043.91 | 13208.43 | 0.122 | 0.119 | 1.2456 |
| 93 | 12178.29 | 12386.66 | 0.137 | 0.141 | 1.6822 | 12440.2 | 12770.24 | 0.131 | 0.129 | 2.5844 |
| 94 | 12051.88 | 14247.34 | 0.125 | 0.137 | 15.41 | 12030.05 | 12474.07 | 0.135 | 0.12 | 3.5595 |
| 95 | 14635.13 | 14756.85 | 0.134 | 0.137 | 0.8248 | 11957.74 | 12060.67 | 0.127 | 0.123 | 0.8534 |
| 96 | 14901.31 | 14625.99 | 0.132 | 0.14 | -1.882 | 12875.65 | 13305.39 | 0.126 | 0.125 | 3.2298 |
| 97 | 11666.9 | 14726.04 | 0.131 | 0.144 | 20.774 | 12089.66 | 12077.23 | 0.12 | 0.12 | -0.103 |
| 98 | 17259.56 | 17797.04 | 0.125 | 0.12 | 3.0201 | 11011.31 | 11123.65 | 0.133 | 0.133 | 1.0099 |

| 99 | 11173.68 | 11857.65 | 0.146 | 0.146 | 5.7682 | 12346.06 | 12390.58 | 0.12 | 0.12 | 0.3593 |
| --- | --- | --- | --- | --- | --- | --- | --- | --- | --- | --- |
| 100 | 12429.84 | 12455.91 | 0.142 | 0.143 | 0.2093 | 13614.51 | 13769.24 | 0.125 | 0.119 | 1.1237 |
| 101 | 12962.21 | 15056.64 | 0.133 | 0.136 | 13.91 | 12985.62 | 13217.58 | 0.115 | 0.122 | 1.7549 |
| 102 | 13340.47 | 13231.77 | 0.137 | 0.136 | -0.822 | 14777.25 | 15131.69 | 0.122 | 0.123 | 2.3423 |
| 103 | 13691.25 | 13213.21 | 0.136 | 0.139 | -3.618 | 11575.58 | 12316.88 | 0.129 | 0.129 | 6.0186 |
| 104 | 11845.79 | 12422.23 | 0.112 | 0.121 | 4.6404 | 14987.72 | 15324.49 | 0.134 | 0.129 | 2.1976 |
| 105 | 12430.94 | 12639.01 | 0.112 | 0.123 | 1.6463 | 12576.4 | 12675.48 | 0.127 | 0.12 | 0.7817 |
| 106 | 12465.73 | 13036.6 | 0.136 | 0.137 | 4.379 | 10585.68 | 11868.37 | 0.135 | 0.134 | 10.808 |
| 107 | 11682.33 | 12359.23 | 0.135 | 0.134 | 5.4769 | 16249.54 | 16741.01 | 0.132 | 0.129 | 2.9357 |
| 108 | 11554.3 | 11868.6 | 0.146 | 0.145 | 2.6482 | 11082.94 | 11058.08 | 0.12 | 0.12 | -0.225 |
| 109 | 13021.55 | 13389.97 | 0.127 | 0.138 | 2.7515 | 13252.76 | 13443.8 | 0.124 | 0.123 | 1.421 |
| 110 | 11740.26 | 13319.46 | 0.115 | 0.115 | 11.856 | 12058.02 | 12157.51 | 0.125 | 0.12 | 0.8184 |
| 111 | 11805.25 | 12481.89 | 0.123 | 0.118 | 5.421 | 11393 | 11624.33 | 0.138 | 0.141 | 1.9901 |
| 112 | 14724.97 | 15115.73 | 0.122 | 0.128 | 2.5851 | 12960.87 | 13115.6 | 0.125 | 0.119 | 1.1797 |
| 113 | 11947.72 | 11951.86 | 0.141 | 0.141 | 0.0347 | 11615.96 | 11668.6 | 0.119 | 0.119 | 0.4511 |
| 114 | 15176.61 | 15743.8 | 0.132 | 0.126 | 3.6026 | 12366.28 | 12781.96 | 0.128 | 0.121 | 3.2521 |
| 115 | 12166.4 | 13623.64 | 0.122 | 0.125 | 10.696 | 10951.39 | 12070.08 | 0.123 | 0.122 | 9.2683 |
| 116 | 12874.82 | 14404.94 | 0.127 | 0.134 | 10.622 | 13125.77 | 13329.74 | 0.129 | 0.128 | 1.5302 |
| 117 | 11949.47 | 12234.06 | 0.141 | 0.14 | 2.3262 | 11206.17 | 11244.66 | 0.132 | 0.131 | 0.3423 |
| 118 | 12372.65 | 12817.45 | 0.145 | 0.147 | 3.4703 | 11356.17 | 11766.53 | 0.123 | 0.127 | 3.4876 |
| 119 | 13320.33 | 13188.08 | 0.114 | 0.121 | -1.003 | 12551.12 | 13165.11 | 0.127 | 0.127 | 4.6637 |
| 120 | 11621.02 | 11623.95 | 0.146 | 0.146 | 0.0252 | 12426.96 | 13234.92 | 0.125 | 0.125 | 6.1047 |
| 121 | 13131.14 | 13129.42 | 0.142 | 0.142 | -0.013 | 11707.98 | 11810.91 | 0.121 | 0.117 | 0.8715 |
| 122 | 13425.1 | 14829.12 | 0.118 | 0.123 | 9.468 | 11905.17 | 12013.46 | 0.121 | 0.123 | 0.9014 |
| 123 | 12478.99 | 12613.42 | 0.111 | 0.117 | 1.0658 | 15129.35 | 16402.41 | 0.127 | 0.128 | 7.7615 |
| 124 | 13789.24 | 14216.81 | 0.136 | 0.133 | 3.0075 | 12155.81 | 12489.58 | 0.135 | 0.137 | 2.6723 |
| 125 | 11920.04 | 12623.25 | 0.131 | 0.122 | 5.5707 | 10638.71 | 10791.97 | 0.13 | 0.129 | 1.4201 |
| 126 | 11325.1 | 12078.9 | 0.123 | 0.123 | 6.2406 | 11660.81 | 12466.59 | 0.133 | 0.133 | 6.4635 |
| 127 | 12846.17 | 17625.08 | 0.138 | 0.132 | 27.114 | 12584.99 | 12864.61 | 0.132 | 0.136 | 2.1736 |
| 128 | 13596.67 | 15330.81 | 0.138 | 0.132 | 11.311 | 12996.07 | 13200.04 | 0.129 | 0.127 | 1.5452 |
| 129 | 15805.8 | 15996.69 | 0.123 | 0.129 | 1.1933 | 10741.55 | 11783.49 | 0.125 | 0.121 | 8.8424 |
| 130 | 11991.56 | 11964.28 | 0.143 | 0.142 | -0.228 | 12421.53 | 12648.52 | 0.133 | 0.132 | 1.7946 |
| 131 | 14084.31 | 14480.43 | 0.119 | 0.125 | 2.7355 | 11099.77 | 11164.83 | 0.124 | 0.124 | 0.5827 |
| 132 | 11391.96 | 11836.26 | 0.148 | 0.149 | 3.7537 | 14012.06 | 14073.77 | 0.12 | 0.124 | 0.4385 |
| 133 | 11809.92 | 12676.11 | 0.145 | 0.146 | 6.8332 | 11943.83 | 11996.46 | 0.121 | 0.121 | 0.4388 |
| 134 | 15333.39 | 15694.4 | 0.129 | 0.127 | 2.3002 | 12531.41 | 12371.59 | 0.122 | 0.117 | -1.292 |
| 135 | 12278.31 | 12290.74 | 0.145 | 0.145 | 0.1011 | 11223.12 | 11380.52 | 0.13 | 0.131 | 1.3831 |
| 136 | 12093.58 | 12250.99 | 0.146 | 0.146 | 1.2848 | 12142.13 | 12116.07 | 0.124 | 0.122 | -0.215 |
| 137 | 11394.18 | 13072 | 0.144 | 0.149 | 12.835 | 11734.85 | 11708.79 | 0.126 | 0.124 | -0.223 |
| 138 | 11879.15 | 16237.85 | 0.133 | 0.136 | 26.843 | 11666 | 11865.82 | 0.128 | 0.128 | 1.6841 |
| 139 | 14926.35 | 14425.77 | 0.107 | 0.111 | -3.47 | 11452.91 | 11867.59 | 0.132 | 0.13 | 3.4942 |
| 140 | 11915.51 | 12344.66 | 0.142 | 0.14 | 3.4764 | 11035.73 | 11051.59 | 0.124 | 0.125 | 0.1435 |
| 141 | 12556.27 | 12810.03 | 0.133 | 0.135 | 1.981 | 13462.76 | 13562.26 | 0.121 | 0.117 | 0.7336 |
| 142 | 14116.02 | 13683.05 | 0.117 | 0.121 | -3.164 | 15222.88 | 16329.29 | 0.137 | 0.143 | 6.7756 |
| 143 | 10843.85 | 11633.51 | 0.124 | 0.126 | 6.7878 | 13719.96 | 13621.97 | 0.123 | 0.122 | -0.719 |
| 144 | 13330.65 | 15596.29 | 0.121 | 0.114 | 14.527 | 14156.1 | 14356.48 | 0.128 | 0.125 | 1.3958 |
| 145 | 11711.38 | 13684.76 | 0.143 | 0.148 | 14.42 | 10874.44 | 11492.8 | 0.131 | 0.13 | 5.3804 |
| 146 | 13546.04 | 16251.35 | 0.119 | 0.124 | 16.647 | 15547.64 | 15647.23 | 0.129 | 0.126 | 0.6364 |
| 147 | 13157.64 | 13343.29 | 0.116 | 0.127 | 1.3913 | 12880.77 | 13071.81 | 0.127 | 0.126 | 1.4615 |
| 148 | 11317.83 | 13765.95 | 0.143 | 0.147 | 17.784 | 13923.57 | 14039.01 | 0.122 | 0.121 | 0.8223 |

| 149 | 11428.47 | 11928.34 | 0.128 | 0.124 | 4.1906 | 10666.02 | 10553.98 | 0.122 | 0.116 | -1.062 |
| --- | --- | --- | --- | --- | --- | --- | --- | --- | --- | --- |
| 150 | 12267.67 | 12283.53 | 0.142 | 0.141 | 0.1291 | 15143.02 | 16084.37 | 0.125 | 0.128 | 5.8526 |
| 151 | 11900.19 | 12664.19 | 0.137 | 0.131 | 6.0328 | 11437.05 | 11502.11 | 0.122 | 0.123 | 0.5656 |
| 152 | 11515.78 | 11739.96 | 0.145 | 0.144 | 1.9095 | 12592.65 | 12963.79 | 0.124 | 0.122 | 2.8629 |
| 153 | 11674.27 | 12014.43 | 0.147 | 0.145 | 2.8312 | 15351.48 | 15273.28 | 0.116 | 0.119 | -0.512 |
| 154 | 15384.08 | 15388.22 | 0.129 | 0.13 | 0.0269 | 11037.46 | 11676.52 | 0.139 | 0.138 | 5.4731 |
| 155 | 11643.91 | 13015.36 | 0.135 | 0.131 | 10.537 | 12015.91 | 12134.11 | 0.12 | 0.121 | 0.9741 |
| 156 | 12258.41 | 12752.5 | 0.148 | 0.148 | 3.8745 | 13073.49 | 13172.99 | 0.125 | 0.121 | 0.7553 |
| 157 | 11474.66 | 11333.83 | 0.115 | 0.124 | -1.243 | 11634.37 | 11796.71 | 0.124 | 0.124 | 1.3761 |
| 158 | 11170.25 | 12711.62 | 0.138 | 0.132 | 12.126 | 15670.61 | 15755.55 | 0.125 | 0.124 | 0.5391 |
| 159 | 12670.22 | 12674.36 | 0.14 | 0.141 | 0.0327 | 12188.24 | 12253.3 | 0.121 | 0.121 | 0.531 |
| 160 | 12459.59 | 13129.88 | 0.115 | 0.124 | 5.1051 | 12305.76 | 12612.07 | 0.13 | 0.13 | 2.4287 |
| 161 | 14797.84 | 15136.34 | 0.132 | 0.13 | 2.2363 | 13708.72 | 15331.25 | 0.134 | 0.139 | 10.583 |
| 162 | 13372.97 | 15036.57 | 0.116 | 0.114 | 11.064 | 12191.51 | 12291.01 | 0.124 | 0.119 | 0.8095 |
| 163 | 11954.01 | 12422.66 | 0.122 | 0.12 | 3.7725 | 13081.09 | 13788.8 | 0.12 | 0.12 | 5.1325 |
| 164 | 15170.37 | 15702.5 | 0.129 | 0.124 | 3.3888 | 13971.96 | 14138.91 | 0.12 | 0.117 | 1.1808 |
| 165 | 15395.32 | 16254.61 | 0.129 | 0.128 | 5.2864 | 10607.49 | 10748.83 | 0.129 | 0.13 | 1.3149 |
| 166 | 11459.39 | 13183.61 | 0.143 | 0.141 | 13.078 | 12790.57 | 12981.61 | 0.126 | 0.126 | 1.4716 |
| 167 | 12198.76 | 12788.81 | 0.127 | 0.132 | 4.6138 | 12662.46 | 12761.55 | 0.126 | 0.12 | 0.7764 |
| 168 | 12765.36 | 12867.37 | 0.115 | 0.127 | 0.7928 | 13958.55 | 13970.27 | 0.126 | 0.125 | 0.0839 |
| 169 | 10918.11 | 11308.27 | 0.146 | 0.143 | 3.4502 | 12798.64 | 13197.97 | 0.13 | 0.128 | 3.0257 |
| 170 | 12157.98 | 12965.14 | 0.11 | 0.119 | 6.2256 | 14088.82 | 15070.25 | 0.138 | 0.138 | 6.5123 |
| 171 | 12480.59 | 12538.58 | 0.141 | 0.143 | 0.4625 | 14121.51 | 14272.8 | 0.124 | 0.122 | 1.0601 |
| 172 | 12041.94 | 12272.36 | 0.117 | 0.127 | 1.8775 | 14181.6 | 14269.47 | 0.128 | 0.127 | 0.6158 |
| 173 | 11129.54 | 14699.77 | 0.129 | 0.142 | 24.288 | 11513.99 | 11577.83 | 0.126 | 0.123 | 0.5515 |
| 174 | 12194.82 | 11998.01 | 0.123 | 0.129 | -1.64 | 11748.63 | 12435.91 | 0.135 | 0.134 | 5.5266 |
| 175 | 11440.19 | 11499.89 | 0.14 | 0.14 | 0.5192 | 14088.36 | 14151.91 | 0.121 | 0.127 | 0.4491 |
| 176 | 13503.74 | 13600.98 | 0.119 | 0.121 | 0.715 | 11681.23 | 11725.79 | 0.13 | 0.13 | 0.38 |
| 177 | 11588.95 | 11676.61 | 0.115 | 0.121 | 0.7507 | 12016.66 | 12116.15 | 0.125 | 0.121 | 0.8212 |
| 178 | 12040.7 | 13133.42 | 0.126 | 0.135 | 8.3202 | 12503.95 | 12790.51 | 0.127 | 0.128 | 2.2405 |
| 179 | 11533.45 | 12097.68 | 0.116 | 0.129 | 4.6639 | 10971.39 | 12085.59 | 0.139 | 0.14 | 9.2192 |
| 180 | 12160.46 | 12310.08 | 0.14 | 0.14 | 1.2154 | 11846.06 | 12502.3 | 0.125 | 0.12 | 5.2489 |
| 181 | 11048.13 | 10728.47 | 0.126 | 0.132 | -2.98 | 11360.47 | 11447.42 | 0.129 | 0.126 | 0.7596 |
| 182 | 12078.95 | 12625.76 | 0.149 | 0.148 | 4.3309 | 11590.96 | 11854.09 | 0.125 | 0.127 | 2.2198 |
| 183 | 11991.42 | 12510.59 | 0.135 | 0.134 | 4.1498 | 11653.8 | 11820.96 | 0.126 | 0.124 | 1.4141 |
| 184 | 12421.72 | 14361.21 | 0.141 | 0.144 | 13.505 | 11536.59 | 11875.62 | 0.133 | 0.131 | 2.8548 |
| 185 | 11186.37 | 11430.75 | 0.146 | 0.145 | 2.138 | 11490.46 | 11657.62 | 0.126 | 0.123 | 1.4339 |
| 186 | 11212.97 | 11701.59 | 0.128 | 0.131 | 4.1756 | 13848.65 | 14196.05 | 0.125 | 0.12 | 2.4472 |
| 187 | 12931.19 | 13057.34 | 0.137 | 0.14 | 0.9661 | 12308.89 | 12649.3 | 0.126 | 0.128 | 2.6912 |
| 188 | 13768.25 | 14827.08 | 0.128 | 0.129 | 7.1412 | 12394.67 | 12644 | 0.12 | 0.123 | 1.9719 |
| 189 | 12652.31 | 12740.55 | 0.123 | 0.128 | 0.6927 | 11724.68 | 12109 | 0.12 | 0.12 | 3.1738 |
| 190 | 12165.91 | 12223.78 | 0.14 | 0.139 | 0.4734 | 11481.4 | 11468.98 | 0.123 | 0.123 | -0.108 |
| 191 | 10944.71 | 11313.95 | 0.13 | 0.126 | 3.2636 | 11406.23 | 11443.51 | 0.131 | 0.13 | 0.3258 |
| 192 | 11344.51 | 12068.73 | 0.124 | 0.125 | 6.0007 | 11305.92 | 11080.4 | 0.12 | 0.119 | -2.035 |
| 193 | 13511.42 | 13784.82 | 0.104 | 0.119 | 1.9833 | 12113.7 | 12145.33 | 0.125 | 0.124 | 0.2604 |
| 194 | 11731.44 | 13867.71 | 0.145 | 0.149 | 15.405 | 10518.2 | 10582.55 | 0.13 | 0.129 | 0.6081 |
| 195 | 11147.58 | 12258.36 | 0.136 | 0.133 | 9.0614 | 12420.29 | 12867.22 | 0.122 | 0.126 | 3.4734 |
| 196 | 12849.83 | 13124.27 | 0.115 | 0.122 | 2.0911 | 15023.2 | 14879.89 | 0.128 | 0.128 | -0.963 |
| 197 | 11675.63 | 12150.4 | 0.133 | 0.129 | 3.9074 | 10973.24 | 11123.15 | 0.125 | 0.124 | 1.3478 |
| 198 | 14584.24 | 15047.22 | 0.125 | 0.132 | 3.0768 | 10847.62 | 10871.26 | 0.125 | 0.127 | 0.2175 |

| 199 | 11561.24 | 11838.06 | 0.142 | 0.14 | 2.3383 | 11625.34 | 11690.4 | 0.123 | 0.123 | 0.5565 |
| --- | --- | --- | --- | --- | --- | --- | --- | --- | --- | --- |
| 200 | 12879.14 | 14129.39 | 0.122 | 0.111 | 8.8485 | 14600.59 | 14522.51 | 0.114 | 0.121 | -0.538 |
| 201 | 11715.16 | 13782.22 | 0.129 | 0.14 | 14.998 | 12126.19 | 12100.12 | 0.124 | 0.122 | -0.215 |
| 202 | 13532.74 | 13936.26 | 0.13 | 0.131 | 2.8955 | 14058.72 | 16087.11 | 0.134 | 0.138 | 12.609 |
| 203 | 12748.61 | 13894.46 | 0.128 | 0.142 | 8.2469 | 10753.3 | 10856.23 | 0.123 | 0.119 | 0.9481 |
| 204 | 15678.77 | 15988.77 | 0.129 | 0.127 | 1.9389 | 12022.22 | 13120.03 | 0.133 | 0.134 | 8.3674 |
| 205 | 11151.38 | 13384.43 | 0.142 | 0.146 | 16.684 | 15152.1 | 16759.23 | 0.136 | 0.138 | 9.5895 |
| 206 | 11270.04 | 11290.75 | 0.141 | 0.144 | 0.1834 | 10286.37 | 10564.27 | 0.128 | 0.129 | 2.6306 |
| 207 | 12703.28 | 13562.27 | 0.113 | 0.122 | 6.3337 | 11531.59 | 11565.02 | 0.127 | 0.128 | 0.2891 |
| 208 | 11100.09 | 11485.1 | 0.149 | 0.148 | 3.3523 | 12460.34 | 12719.51 | 0.132 | 0.13 | 2.0376 |
| 209 | 13334.83 | 12676.37 | 0.136 | 0.14 | -5.194 | 12351.33 | 12486.06 | 0.122 | 0.122 | 1.0791 |
| 210 | 16167.93 | 16693.73 | 0.124 | 0.134 | 3.1497 | 13282.75 | 13035.76 | 0.118 | 0.117 | -1.895 |
| 211 | 10937.16 | 11065.36 | 0.135 | 0.131 | 1.1586 | 11735.79 | 11853.99 | 0.12 | 0.121 | 0.9971 |
| 212 | 12094.07 | 12293.36 | 0.136 | 0.133 | 1.6211 | 13032.29 | 13678.32 | 0.127 | 0.13 | 4.723 |
| 213 | 11355.31 | 11809.11 | 0.145 | 0.145 | 3.8428 | 12639.18 | 12413.66 | 0.118 | 0.117 | -1.817 |
| 214 | 12259.93 | 12179.72 | 0.115 | 0.124 | -0.659 | 10869.92 | 13085.71 | 0.131 | 0.137 | 16.933 |
| 215 | 11322.95 | 11507.88 | 0.114 | 0.122 | 1.6071 | 12842.97 | 12944.8 | 0.126 | 0.123 | 0.7867 |
| 216 | 12064.61 | 11731.94 | 0.118 | 0.124 | -2.836 | 15033.78 | 16244.34 | 0.129 | 0.129 | 7.4522 |
| 217 | 15358.13 | 15763.99 | 0.129 | 0.133 | 2.5746 | 10965.42 | 11441.73 | 0.128 | 0.13 | 4.1629 |
| 218 | 12770.75 | 12772.46 | 0.142 | 0.142 | 0.0134 | 10521.6 | 11500.03 | 0.137 | 0.138 | 8.508 |
| 219 | 12798.56 | 14781.9 | 0.139 | 0.132 | 13.417 | 11488.01 | 12107.33 | 0.132 | 0.13 | 5.1153 |
| 220 | 13390.1 | 13373.53 | 0.14 | 0.14 | -0.124 | 10750.47 | 11919.28 | 0.138 | 0.137 | 9.806 |
| 221 | 12856.69 | 14311.3 | 0.129 | 0.138 | 10.164 | 12991.23 | 12765.7 | 0.117 | 0.116 | -1.767 |
| 222 | 12010.44 | 11895.76 | 0.119 | 0.125 | -0.964 | 13241.62 | 13586.13 | 0.129 | 0.127 | 2.5357 |
| 223 | 11787 | 12555.65 | 0.138 | 0.132 | 6.1219 | 13255.77 | 13530.96 | 0.128 | 0.128 | 2.0337 |
| 224 | 11819.66 | 11874.18 | 0.117 | 0.124 | 0.4592 | 11815.02 | 12241.74 | 0.124 | 0.128 | 3.4858 |
| 225 | 13617.72 | 13965 | 0.135 | 0.133 | 2.4868 | 14907.6 | 16198.24 | 0.124 | 0.126 | 7.9678 |
| 226 | 11630.46 | 15149.09 | 0.124 | 0.143 | 23.227 | 11505.69 | 11647.03 | 0.123 | 0.124 | 1.2135 |
| 227 | 12138.94 | 12758.23 | 0.139 | 0.137 | 4.854 | 14448.25 | 14939.58 | 0.135 | 0.129 | 3.2888 |
| 228 | 11754.81 | 12228.36 | 0.137 | 0.132 | 3.8726 | 11991.4 | 12302.74 | 0.134 | 0.136 | 2.5306 |
| 229 | 11885.26 | 13764 | 0.125 | 0.12 | 13.65 | 10053.67 | 11299.59 | 0.138 | 0.135 | 11.026 |
| 230 | 12702.22 | 14299.41 | 0.108 | 0.111 | 11.17 | 12211.27 | 12329.46 | 0.122 | 0.123 | 0.9587 |
| 231 | 12548.58 | 12628.46 | 0.118 | 0.123 | 0.6325 | 11922.03 | 12070.22 | 0.127 | 0.132 | 1.2278 |
| 232 | 12026.97 | 12448.84 | 0.128 | 0.125 | 3.3889 | 13742.39 | 12763.86 | 0.126 | 0.129 | -7.666 |
| 233 | 13022.31 | 15410.07 | 0.123 | 0.114 | 15.495 | 12706.28 | 12680.21 | 0.123 | 0.121 | -0.206 |
| 234 | 11749.87 | 12345.1 | 0.14 | 0.142 | 4.8216 | 14780.88 | 16154.78 | 0.131 | 0.132 | 8.5046 |
| 235 | 14157.46 | 13486.79 | 0.137 | 0.141 | -4.973 | 12350.21 | 12449.71 | 0.123 | 0.119 | 0.7992 |
| 236 | 11593.3 | 11910.11 | 0.127 | 0.128 | 2.66 | 12452.18 | 12668.21 | 0.12 | 0.113 | 1.7053 |
| 237 | 15427.22 | 15974.62 | 0.13 | 0.133 | 3.4267 | 12874.19 | 12992.39 | 0.116 | 0.117 | 0.9097 |
| 238 | 14446.46 | 14400.51 | 0.129 | 0.131 | -0.319 | 12468.85 | 12660.9 | 0.127 | 0.129 | 1.5168 |
| 239 | 12039.76 | 13851.18 | 0.145 | 0.148 | 13.078 | 11794.93 | 12026.47 | 0.128 | 0.128 | 1.9253 |
| 240 | 11756.82 | 12270.91 | 0.135 | 0.133 | 4.1895 | 11773.5 | 11547.97 | 0.122 | 0.121 | -1.953 |
| 241 | 10634.86 | 14687.34 | 0.126 | 0.141 | 27.592 | 11284.96 | 11148.06 | 0.127 | 0.121 | -1.228 |
| 242 | 11414.53 | 11601.43 | 0.144 | 0.144 | 1.611 | 11770.85 | 12042.31 | 0.123 | 0.126 | 2.2542 |
| 243 | 12160.43 | 14111.39 | 0.139 | 0.146 | 13.825 | 16318.79 | 17614.2 | 0.131 | 0.134 | 7.3543 |
| 244 | 12154.52 | 13144.06 | 0.138 | 0.133 | 7.5284 | 12694.35 | 12885.39 | 0.128 | 0.127 | 1.4826 |
| 245 | 12150.49 | 12468.78 | 0.138 | 0.135 | 2.5527 | 10913.39 | 10964.01 | 0.129 | 0.127 | 0.4617 |
| 246 | 11469.39 | 11879.05 | 0.131 | 0.129 | 3.4486 | 14624.83 | 14683.4 | 0.123 | 0.123 | 0.3989 |
| 247 | 12505.94 | 12466.73 | 0.144 | 0.143 | -0.314 | 11714.82 | 11868.58 | 0.126 | 0.127 | 1.2955 |
| 248 | 12122.03 | 12491.47 | 0.137 | 0.139 | 2.9576 | 11149.6 | 11554.99 | 0.12 | 0.124 | 3.5084 |

| 249 | 13670.75 | 14336.4 | 0.133 | 0.137 | 4.6431 | 12229.34 | 12206.49 | 0.126 | 0.123 | -0.187 |
| --- | --- | --- | --- | --- | --- | --- | --- | --- | --- | --- |
| 250 | 12990.69 | 13131.24 | 0.115 | 0.114 | 1.0704 | 15090.41 | 15952.6 | 0.128 | 0.132 | 5.4047 |
| 251 | 11337.62 | 12393.3 | 0.148 | 0.146 | 8.5182 | 14257.3 | 14332.95 | 0.118 | 0.122 | 0.5278 |
| 252 | 11988.47 | 11953.62 | 0.143 | 0.144 | -0.292 | 13141.58 | 13126.73 | 0.121 | 0.127 | -0.113 |
| 253 | 11045.53 | 11435.69 | 0.15 | 0.148 | 3.4118 | 12296.56 | 12424.46 | 0.13 | 0.13 | 1.0295 |
| 254 | 14294.61 | 14728.25 | 0.132 | 0.131 | 2.9443 | 10933.45 | 10804.83 | 0.122 | 0.115 | -1.19 |
| 255 | 16643.34 | 17275.47 | 0.124 | 0.123 | 3.6591 | 14011.81 | 13459.37 | 0.126 | 0.121 | -4.105 |
| 256 | 14352.38 | 15560.12 | 0.113 | 0.114 | 7.7618 | 11877.71 | 12174.73 | 0.13 | 0.129 | 2.4396 |
| 257 | 11567.4 | 11766.72 | 0.147 | 0.146 | 1.694 | 13057.28 | 13196.15 | 0.12 | 0.12 | 1.0524 |
| 258 | 13155.21 | 13337.51 | 0.137 | 0.138 | 1.3669 | 12568.04 | 12842.22 | 0.119 | 0.124 | 2.135 |
| 259 | 12993.52 | 15280.25 | 0.115 | 0.112 | 14.965 | 12401.97 | 12749.51 | 0.126 | 0.124 | 2.7259 |
| 260 | 13228.82 | 14996.73 | 0.139 | 0.132 | 11.789 | 12584.44 | 12338.67 | 0.124 | 0.124 | -1.992 |
| 261 | 12238.24 | 12878.69 | 0.122 | 0.123 | 4.973 | 12381.18 | 12582.27 | 0.124 | 0.121 | 1.5982 |
| 262 | 12919.36 | 14870.58 | 0.135 | 0.135 | 13.121 | 12297.59 | 12367.17 | 0.114 | 0.115 | 0.5626 |
| 263 | 11632.62 | 12306.59 | 0.147 | 0.147 | 5.4765 | 11816.3 | 11926.3 | 0.134 | 0.132 | 0.9223 |
| 264 | 11552.91 | 11965.87 | 0.131 | 0.124 | 3.4512 | 13111.44 | 12885.91 | 0.116 | 0.115 | -1.75 |
| 265 | 15306.71 | 15791.06 | 0.132 | 0.133 | 3.0672 | 13101.45 | 13236.18 | 0.12 | 0.12 | 1.0179 |
| 266 | 11494.24 | 11939.05 | 0.146 | 0.147 | 3.7256 | 12765.07 | 12644.99 | 0.126 | 0.128 | -0.95 |
| 267 | 11807.55 | 12699.55 | 0.131 | 0.122 | 7.0238 | 13749.95 | 12667.56 | 0.12 | 0.129 | -8.545 |
| 268 | 11072.62 | 12668.51 | 0.141 | 0.147 | 12.597 | 13766.06 | 13801.8 | 0.128 | 0.125 | 0.2589 |
| 269 | 12029.97 | 12874.06 | 0.138 | 0.133 | 6.5565 | 14924.58 | 15767.98 | 0.134 | 0.137 | 5.3488 |
| 270 | 11081.99 | 11675.74 | 0.129 | 0.138 | 5.0853 | 14763.32 | 14821.9 | 0.123 | 0.123 | 0.3952 |
| 271 | 11707.42 | 11770.55 | 0.139 | 0.14 | 0.5364 | 11968.53 | 12379.07 | 0.132 | 0.13 | 3.3164 |
| 272 | 12836.99 | 14429.03 | 0.137 | 0.131 | 11.034 | 11212.77 | 11398.75 | 0.12 | 0.126 | 1.6316 |
| 273 | 11202.7 | 14834.89 | 0.131 | 0.145 | 24.484 | 11114.12 | 11255.46 | 0.128 | 0.13 | 1.2557 |
| 274 | 11856.71 | 11905.79 | 0.145 | 0.144 | 0.4122 | 12355.42 | 12454.92 | 0.126 | 0.122 | 0.7989 |
| 275 | 13606.88 | 13662.02 | 0.136 | 0.137 | 0.4037 | 12994.99 | 13364.41 | 0.122 | 0.12 | 2.7643 |
| 276 | 11955.4 | 12542.33 | 0.126 | 0.123 | 4.6796 | 10541.38 | 11131.67 | 0.132 | 0.111 | 5.3028 |
| 277 | 12526.33 | 12468.84 | 0.142 | 0.141 | -0.461 | 11448.06 | 11161.28 | 0.125 | 0.128 | -2.569 |
| 278 | 11037.17 | 13067.04 | 0.125 | 0.14 | 15.534 | 14417.59 | 14621.56 | 0.125 | 0.124 | 1.395 |
| 279 | 14195.59 | 14565.13 | 0.123 | 0.13 | 2.5372 | 11526.71 | 12093.02 | 0.134 | 0.127 | 4.6829 |
| 280 | 11954.87 | 12570.09 | 0.122 | 0.119 | 4.8943 | 11719.42 | 12220.08 | 0.133 | 0.133 | 4.097 |
| 281 | 12780.21 | 13845.1 | 0.122 | 0.115 | 7.6914 | 12293.75 | 12422.66 | 0.134 | 0.133 | 1.0377 |
| 282 | 13561.73 | 13610.13 | 0.136 | 0.137 | 0.3557 | 11003.03 | 11140.52 | 0.122 | 0.121 | 1.2341 |
| 283 | 12606.48 | 12597.7 | 0.143 | 0.143 | -0.07 | 11112.91 | 11114.12 | 0.123 | 0.124 | 0.0109 |
| 284 | 10373.05 | 10754.92 | 0.149 | 0.147 | 3.5507 | 11090.28 | 11142.91 | 0.12 | 0.121 | 0.4724 |
| 285 | 12410.34 | 14088.05 | 0.136 | 0.139 | 11.909 | 12349.16 | 12336.73 | 0.12 | 0.12 | -0.101 |
| 286 | 12594.99 | 12509.93 | 0.132 | 0.134 | -0.68 | 12287.48 | 12295.05 | 0.122 | 0.123 | 0.0616 |
| 287 | 11391.83 | 11827.97 | 0.146 | 0.144 | 3.6873 | 13473.91 | 13447.84 | 0.122 | 0.121 | -0.194 |
| 288 | 15275.04 | 15716.58 | 0.131 | 0.126 | 2.8094 | 11211.8 | 12126.73 | 0.12 | 0.12 | 7.5447 |
| 289 | 12697.04 | 12661.47 | 0.137 | 0.137 | -0.281 | 11020.72 | 11008.29 | 0.126 | 0.126 | -0.113 |
| 290 | 13903.58 | 13278.05 | 0.136 | 0.139 | -4.711 | 11022.86 | 11196.62 | 0.125 | 0.125 | 1.5519 |
| 291 | 11608.06 | 12073.36 | 0.123 | 0.124 | 3.854 | 11658.1 | 11732.83 | 0.127 | 0.125 | 0.6369 |
| 292 | 12660.28 | 12627.94 | 0.128 | 0.13 | -0.256 | 11187.94 | 11286.64 | 0.132 | 0.134 | 0.8745 |
| 293 | 11611.76 | 11909.99 | 0.146 | 0.145 | 2.5041 | 14731.45 | 15054.76 | 0.122 | 0.123 | 2.1476 |
| 294 | 12989.11 | 13314.67 | 0.131 | 0.133 | 2.4451 | 15252.87 | 15255 | 0.116 | 0.115 | 0.014 |
| 295 | 16449.08 | 16866.57 | 0.13 | 0.125 | 2.4752 | 12596.51 | 13475.59 | 0.135 | 0.134 | 6.5235 |
| 296 | 13541.84 | 15591.41 | 0.119 | 0.122 | 13.146 | 12285.61 | 12060.08 | 0.119 | 0.118 | -1.87 |
| 297 | 11855.36 | 11895.36 | 0.143 | 0.144 | 0.3363 | 11952.38 | 12039.16 | 0.128 | 0.128 | 0.7208 |
| 298 | 12252.42 | 14126.77 | 0.145 | 0.148 | 13.268 | 14304.58 | 13264.13 | 0.125 | 0.126 | -7.844 |

| 299 | 10547.43 | 10727.05 | 0.143 | 0.141 | 1.6745 | 11935.31 | 12000.37 | 0.118 | 0.119 | 0.5422 |
| --- | --- | --- | --- | --- | --- | --- | --- | --- | --- | --- |
| 300 | 12117.81 | 12038.86 | 0.121 | 0.131 | -0.656 | 15096.52 | 15566.57 | 0.129 | 0.128 | 3.0196 |
| 301 | 11709.39 | 13389.05 | 0.138 | 0.132 | 12.545 | 11939.54 | 11759.89 | 0.129 | 0.122 | -1.528 |
| 302 | 11897.98 | 13266.68 | 0.126 | 0.133 | 10.317 | 11842.81 | 11934.52 | 0.115 | 0.118 | 0.7685 |
| 303 | 11098.11 | 11400.78 | 0.144 | 0.143 | 2.6548 | 13985.13 | 14859.83 | 0.12 | 0.126 | 5.8863 |
| 304 | 12788.27 | 13553.31 | 0.124 | 0.119 | 5.6447 | 12180.45 | 12102.17 | 0.124 | 0.123 | -0.647 |
| 305 | 14482 | 15239 | 0.109 | 0.115 | 4.9675 | 11542.42 | 11582.12 | 0.126 | 0.123 | 0.3428 |
| 306 | 16606.21 | 16489.26 | 0.128 | 0.133 | -0.709 | 14473.87 | 15838.98 | 0.124 | 0.126 | 8.6187 |
| 307 | 10755.36 | 11441.55 | 0.132 | 0.131 | 5.9973 | 15795.01 | 16007.81 | 0.127 | 0.129 | 1.3294 |
| 308 | 10701.06 | 11270.01 | 0.147 | 0.146 | 5.0483 | 12459.45 | 12746.02 | 0.121 | 0.122 | 2.2483 |
| 309 | 11830.31 | 11687.92 | 0.138 | 0.138 | -1.218 | 11043.6 | 11200.29 | 0.128 | 0.123 | 1.399 |
| 310 | 15582.73 | 16244.28 | 0.131 | 0.125 | 4.0725 | 10797.7 | 10850.34 | 0.118 | 0.119 | 0.4851 |
| 311 | 12948.51 | 13125.62 | 0.14 | 0.139 | 1.3493 | 10822.44 | 10945.08 | 0.138 | 0.135 | 1.1205 |
| 312 | 12745.07 | 13199.11 | 0.126 | 0.127 | 3.4399 | 12705.06 | 12789.71 | 0.126 | 0.127 | 0.6618 |
| 313 | 11818.96 | 11826 | 0.121 | 0.128 | 0.0595 | 10923.02 | 10981.01 | 0.132 | 0.132 | 0.5281 |
| 314 | 14552.39 | 14265.07 | 0.121 | 0.127 | -2.014 | 12358.58 | 12525.53 | 0.127 | 0.123 | 1.3329 |
| 315 | 11336.12 | 11612.94 | 0.147 | 0.145 | 2.3837 | 14242.56 | 14557.33 | 0.12 | 0.125 | 2.1623 |
| 316 | 14600.39 | 14836.96 | 0.128 | 0.125 | 1.5945 | 15579.74 | 16530.21 | 0.137 | 0.139 | 5.7499 |
| 317 | 15744.16 | 16165.79 | 0.129 | 0.124 | 2.6082 | 11481.32 | 11620.73 | 0.124 | 0.123 | 1.1997 |
| 318 | 12693.65 | 12648.59 | 0.132 | 0.134 | -0.356 | 11377.53 | 11876.94 | 0.125 | 0.124 | 4.2049 |
| 319 | 14942.6 | 14979.96 | 0.135 | 0.135 | 0.2494 | 11939.26 | 11957.04 | 0.121 | 0.121 | 0.1487 |
| 320 | 14314.27 | 14134.8 | 0.13 | 0.14 | -1.27 | 15698 | 15878.38 | 0.129 | 0.128 | 1.136 |
| 321 | 10965.12 | 11266.07 | 0.145 | 0.144 | 2.6713 | 9990.828 | 10645.16 | 0.141 | 0.138 | 6.1468 |
| 322 | 12001.14 | 12372.56 | 0.137 | 0.133 | 3.002 | 12672.12 | 13142.07 | 0.13 | 0.128 | 3.5759 |
| 323 | 12613.38 | 13028.46 | 0.127 | 0.132 | 3.1859 | 12578.75 | 12591.05 | 0.126 | 0.123 | 0.0977 |
| 324 | 11992.45 | 13160.98 | 0.124 | 0.113 | 8.8787 | 14460.23 | 14365.88 | 0.122 | 0.119 | -0.657 |
| 325 | 11094.65 | 11488.95 | 0.149 | 0.147 | 3.432 | 11884.95 | 11876.16 | 0.129 | 0.129 | -0.074 |
| 326 | 14913.64 | 14619.29 | 0.125 | 0.133 | -2.013 | 11936.17 | 11988.8 | 0.119 | 0.119 | 0.439 |
| 327 | 10934.04 | 10934.04 | 0.145 | 0.145 | 0 | 12872.55 | 12846.48 | 0.127 | 0.125 | -0.203 |
| 328 | 11917.33 | 13875.65 | 0.139 | 0.148 | 14.113 | 12013.79 | 12202.91 | 0.113 | 0.119 | 1.5498 |
| 329 | 12245.4 | 14985.75 | 0.125 | 0.145 | 18.286 | 14267.18 | 14447.09 | 0.129 | 0.131 | 1.2453 |
| 330 | 11530.19 | 15436.4 | 0.125 | 0.144 | 25.305 | 10900.81 | 11003.74 | 0.125 | 0.121 | 0.9354 |
| 331 | 12401.34 | 12485.77 | 0.144 | 0.144 | 0.6763 | 11209.18 | 11963.31 | 0.13 | 0.127 | 6.3037 |
| 332 | 12722.72 | 12819.93 | 0.116 | 0.126 | 0.7583 | 11196.55 | 11235.04 | 0.127 | 0.126 | 0.3426 |
| 333 | 13756.43 | 14908.95 | 0.118 | 0.122 | 7.7303 | 11741.56 | 11758.42 | 0.129 | 0.129 | 0.1434 |
| 334 | 12424.29 | 12568.1 | 0.117 | 0.127 | 1.1443 | 12088.19 | 12861.03 | 0.126 | 0.128 | 6.0092 |
| 335 | 13353.97 | 15097.52 | 0.139 | 0.133 | 11.549 | 12023.19 | 12623.47 | 0.132 | 0.13 | 4.7553 |
| 336 | 13909.74 | 14157.73 | 0.132 | 0.137 | 1.7516 | 11343.3 | 11897.65 | 0.122 | 0.121 | 4.6593 |
| 337 | 12087.15 | 11949.04 | 0.114 | 0.12 | -1.156 | 13985.02 | 13939.46 | 0.117 | 0.12 | -0.327 |
| 338 | 11876.41 | 11991.56 | 0.142 | 0.142 | 0.9602 | 12748.85 | 12955.04 | 0.128 | 0.13 | 1.5916 |
| 339 | 11078.8 | 11375.16 | 0.112 | 0.121 | 2.6053 | 12511.4 | 12660.27 | 0.119 | 0.118 | 1.1759 |
| 340 | 11627.33 | 12350.38 | 0.124 | 0.122 | 5.8545 | 11087.27 | 11285.88 | 0.131 | 0.131 | 1.7598 |
| 341 | 12582.25 | 12628.4 | 0.142 | 0.142 | 0.3655 | 14777.63 | 14974.08 | 0.13 | 0.129 | 1.3119 |
| 342 | 11917.62 | 13792.45 | 0.122 | 0.122 | 13.593 | 12876.91 | 13685.54 | 0.126 | 0.129 | 5.9086 |
| 343 | 12286.65 | 14294.92 | 0.128 | 0.134 | 14.049 | 11571.49 | 12503.85 | 0.135 | 0.136 | 7.4566 |
| 344 | 12079.35 | 14087.22 | 0.143 | 0.147 | 14.253 | 11113.83 | 11280.52 | 0.124 | 0.122 | 1.4777 |
| 345 | 12501.78 | 13188.61 | 0.118 | 0.119 | 5.2077 | 11671.41 | 12426.51 | 0.123 | 0.128 | 6.0765 |
| 346 | 10900.15 | 11288.6 | 0.149 | 0.147 | 3.441 | 11096.32 | 11426.06 | 0.123 | 0.127 | 2.8859 |
| 347 | 11698.08 | 11979.04 | 0.145 | 0.144 | 2.3454 | 12528.47 | 12526.55 | 0.116 | 0.116 | -0.015 |
| 348 | 12352.75 | 14012.45 | 0.115 | 0.117 | 11.845 | 13679.84 | 13726.49 | 0.118 | 0.119 | 0.3399 |

| 349 | 12889.38 | 13151.8 | 0.138 | 0.135 | 1.9954 | 11316.57 | 11664.31 | 0.132 | 0.131 | 2.9812 |
| --- | --- | --- | --- | --- | --- | --- | --- | --- | --- | --- |
| 350 | 11440.21 | 11604.69 | 0.145 | 0.146 | 1.4173 | 12771.57 | 12531.83 | 0.123 | 0.12 | -1.913 |
| 351 | 13749.53 | 14629.2 | 0.118 | 0.115 | 6.0131 | 12625.43 | 12871.37 | 0.125 | 0.127 | 1.9108 |
| 352 | 13871.81 | 13753.96 | 0.115 | 0.113 | -0.857 | 11334.72 | 11532.33 | 0.13 | 0.127 | 1.7135 |
| 353 | 11858.47 | 12271.56 | 0.142 | 0.143 | 3.3662 | 11789.54 | 12055.43 | 0.126 | 0.126 | 2.2056 |
| 354 | 16541.36 | 16855.92 | 0.12 | 0.127 | 1.8662 | 13460.26 | 13525.32 | 0.118 | 0.118 | 0.481 |
| 355 | 12209.31 | 12603.78 | 0.123 | 0.129 | 3.1298 | 11405.86 | 12639.37 | 0.14 | 0.139 | 9.7592 |
| 356 | 13369.51 | 14191.78 | 0.121 | 0.127 | 5.794 | 10492.08 | 10633.41 | 0.126 | 0.127 | 1.3292 |
| 357 | 12187.36 | 12729.82 | 0.134 | 0.134 | 4.2613 | 10882.76 | 10906.4 | 0.125 | 0.126 | 0.2167 |
| 358 | 11720.63 | 12160.37 | 0.129 | 0.128 | 3.6162 | 12939.3 | 13323.62 | 0.118 | 0.118 | 2.8845 |
| 359 | 12700.05 | 12995.08 | 0.113 | 0.121 | 2.2703 | 12266.04 | 12365.12 | 0.126 | 0.12 | 0.8013 |
| 360 | 11886.24 | 11897.66 | 0.14 | 0.141 | 0.096 | 11153.83 | 11295.16 | 0.125 | 0.126 | 1.2513 |
| 361 | 13349.83 | 13361.55 | 0.14 | 0.139 | 0.0877 | 15301.9 | 16278.81 | 0.117 | 0.121 | 6.0011 |
| 362 | 11313.47 | 11488.03 | 0.12 | 0.126 | 1.5195 | 11743.23 | 11859.59 | 0.117 | 0.117 | 0.9812 |
| 363 | 12555.7 | 13524.4 | 0.114 | 0.124 | 7.1626 | 12179.43 | 12502.1 | 0.121 | 0.125 | 2.5809 |
| 364 | 13186.53 | 13344.19 | 0.132 | 0.133 | 1.1815 | 11723.93 | 12035.09 | 0.125 | 0.128 | 2.5855 |
| 365 | 12965.53 | 12310.71 | 0.138 | 0.142 | -5.319 | 15193.59 | 15633.85 | 0.129 | 0.128 | 2.8161 |
| 366 | 11070.36 | 12317.71 | 0.133 | 0.131 | 10.126 | 11638.74 | 11691.38 | 0.118 | 0.118 | 0.4502 |
| 367 | 15926.03 | 15722.13 | 0.123 | 0.132 | -1.297 | 11599.28 | 11734.01 | 0.127 | 0.127 | 1.1482 |
| 368 | 16001.3 | 16423.43 | 0.131 | 0.125 | 2.5703 | 12425.85 | 12544.05 | 0.121 | 0.122 | 0.9423 |
| 369 | 12850.47 | 12386.57 | 0.137 | 0.14 | -3.745 | 12199.58 | 12414.97 | 0.129 | 0.126 | 1.7349 |
| 370 | 12640.29 | 12868.61 | 0.139 | 0.139 | 1.7742 | 12568.62 | 12542.55 | 0.124 | 0.122 | -0.208 |
| 371 | 12771.12 | 12934.17 | 0.134 | 0.134 | 1.2606 | 11481.27 | 11580.76 | 0.129 | 0.124 | 0.8592 |
| 372 | 12082.88 | 13858.78 | 0.142 | 0.147 | 12.814 | 11700.58 | 12182.04 | 0.131 | 0.13 | 3.9522 |
| 373 | 12734.98 | 16454.13 | 0.142 | 0.141 | 22.603 | 11038.47 | 11197.35 | 0.126 | 0.125 | 1.4188 |
| 374 | 10116.5 | 10464.95 | 0.148 | 0.145 | 3.3296 | 12051.85 | 11852.36 | 0.12 | 0.118 | -1.683 |
| 375 | 11168.83 | 11405.94 | 0.145 | 0.144 | 2.0788 | 13976.37 | 14068.8 | 0.128 | 0.129 | 0.657 |
| 376 | 13510.65 | 13650.18 | 0.134 | 0.139 | 1.0222 | 12098.41 | 12085.99 | 0.122 | 0.122 | -0.103 |
| 377 | 12404.27 | 12612.17 | 0.138 | 0.135 | 1.6484 | 12818.07 | 12980.41 | 0.119 | 0.119 | 1.2507 |
| 378 | 11799.22 | 11820.94 | 0.145 | 0.145 | 0.1837 | 12058.06 | 11832.53 | 0.121 | 0.12 | -1.906 |
| 379 | 13733.98 | 14338.85 | 0.121 | 0.123 | 4.2184 | 14314.56 | 14717.03 | 0.122 | 0.122 | 2.7348 |
| 380 | 12083.92 | 15973.12 | 0.135 | 0.132 | 24.348 | 12864.41 | 12609.85 | 0.123 | 0.12 | -2.019 |
| 381 | 12196 | 12920.97 | 0.147 | 0.147 | 5.6108 | 11907 | 12025.2 | 0.119 | 0.12 | 0.9829 |
| 382 | 13954.94 | 15600.72 | 0.135 | 0.137 | 10.549 | 10242.75 | 10743.53 | 0.135 | 0.129 | 4.6612 |
| 383 | 13961.4 | 15433.44 | 0.138 | 0.134 | 9.538 | 12434.38 | 12573.25 | 0.121 | 0.121 | 1.1045 |
| 384 | 13943.53 | 15611.56 | 0.12 | 0.11 | 10.685 | 11281.46 | 11721.41 | 0.132 | 0.13 | 3.7534 |
| 385 | 11037.37 | 11352.47 | 0.145 | 0.145 | 2.7756 | 13570.14 | 13967.47 | 0.125 | 0.123 | 2.8447 |
| 386 | 13282.76 | 13587.75 | 0.118 | 0.113 | 2.2446 | 14275.82 | 14181.97 | 0.122 | 0.12 | -0.662 |
| 387 | 11320.67 | 11621.46 | 0.141 | 0.139 | 2.5883 | 11483.6 | 11525.31 | 0.127 | 0.127 | 0.3619 |
| 388 | 12432.69 | 13465.98 | 0.114 | 0.119 | 7.6734 | 12532.98 | 14198.05 | 0.134 | 0.138 | 11.727 |
| 389 | 11501.82 | 13092.22 | 0.125 | 0.122 | 12.148 | 10581.01 | 10838.62 | 0.131 | 0.131 | 2.3768 |
| 390 | 10557.41 | 13849.23 | 0.126 | 0.141 | 23.769 | 13118.22 | 13825.93 | 0.119 | 0.119 | 5.1187 |
| 391 | 14130.56 | 16426.97 | 0.13 | 0.137 | 13.98 | 11575.69 | 11766.23 | 0.127 | 0.128 | 1.6194 |
| 392 | 11567.86 | 12346.77 | 0.138 | 0.133 | 6.3086 | 10491.96 | 11096.26 | 0.133 | 0.133 | 5.446 |
| 393 | 15882.78 | 15886.92 | 0.129 | 0.129 | 0.0261 | 10131.6 | 10634.98 | 0.131 | 0.132 | 4.7333 |
| 394 | 12406.1 | 13125.53 | 0.127 | 0.13 | 5.4811 | 14551.65 | 14500.02 | 0.118 | 0.122 | -0.356 |
| 395 | 14119.44 | 14862.45 | 0.107 | 0.115 | 4.9993 | 14525.25 | 14564.74 | 0.138 | 0.137 | 0.2712 |
| 396 | 13146.16 | 13692.19 | 0.139 | 0.137 | 3.9879 | 11847.38 | 11685.84 | 0.125 | 0.122 | -1.382 |
| 397 | 13017.53 | 13183.72 | 0.142 | 0.142 | 1.2606 | 15821.37 | 16795.35 | 0.117 | 0.121 | 5.7991 |
| 398 | 13171.41 | 16292.62 | 0.136 | 0.134 | 19.157 | 13362.55 | 13344.77 | 0.12 | 0.118 | -0.133 |

| 399 | 13367.61 | 13581.3 | 0.115 | 0.111 | 1.5734 | 10620.66 | 11729.22 | 0.128 | 0.128 | 9.4513 |
| --- | --- | --- | --- | --- | --- | --- | --- | --- | --- | --- |
| 400 | 10488.25 | 10778.2 | 0.146 | 0.145 | 2.6901 | 10682.2 | 10901.73 | 0.132 | 0.132 | 2.0137 |
| 401 | 10872.85 | 11210.16 | 0.122 | 0.126 | 3.009 | 11650.41 | 12603.75 | 0.136 | 0.135 | 7.564 |
| 402 | 11899.12 | 12082.47 | 0.133 | 0.129 | 1.5174 | 13906.19 | 14045.06 | 0.118 | 0.118 | 0.9888 |
| 403 | 12767.08 | 12771.23 | 0.14 | 0.141 | 0.0324 | 11447.77 | 11306.73 | 0.123 | 0.116 | -1.247 |
| 404 | 12135.08 | 13099.08 | 0.123 | 0.118 | 7.3593 | 12196.94 | 12476.18 | 0.131 | 0.128 | 2.2382 |
| 405 | 13321.08 | 14182.72 | 0.126 | 0.124 | 6.0753 | 13950.28 | 14048.19 | 0.122 | 0.128 | 0.6969 |
| 406 | 13569.9 | 16259.07 | 0.136 | 0.134 | 16.539 | 12016.47 | 12501.29 | 0.125 | 0.119 | 3.8781 |
| 407 | 11636.24 | 12254.89 | 0.143 | 0.14 | 5.0482 | 12115.16 | 12585.74 | 0.127 | 0.126 | 3.7389 |
| 408 | 12790.47 | 13781.77 | 0.129 | 0.135 | 7.1928 | 11083.71 | 11532.53 | 0.131 | 0.131 | 3.8918 |
| 409 | 12204.52 | 12194.37 | 0.12 | 0.129 | -0.083 | 10867.14 | 10854.71 | 0.121 | 0.121 | -0.114 |
| 410 | 10203.7 | 10523.14 | 0.149 | 0.147 | 3.0357 | 13005.09 | 13067.72 | 0.139 | 0.138 | 0.4793 |
| 411 | 11388.27 | 11585.16 | 0.141 | 0.141 | 1.6996 | 12683.07 | 13068.08 | 0.129 | 0.126 | 2.9462 |
| 412 | 11913.6 | 12618.81 | 0.131 | 0.127 | 5.5886 | 12521.88 | 12428.03 | 0.126 | 0.124 | -0.755 |
| 413 | 14465.11 | 14615.91 | 0.132 | 0.136 | 1.0317 | 11392.51 | 11500.92 | 0.127 | 0.122 | 0.9426 |
| 414 | 13559.13 | 13861.56 | 0.132 | 0.139 | 2.1818 | 10557.97 | 10716.84 | 0.125 | 0.124 | 1.4825 |
| 415 | 16009.64 | 16621.65 | 0.124 | 0.121 | 3.682 | 14709.35 | 14733.37 | 0.122 | 0.119 | 0.163 |
| 416 | 13104.29 | 14517.97 | 0.123 | 0.115 | 9.7374 | 11688.36 | 11938.9 | 0.122 | 0.126 | 2.0985 |
| 417 | 12523.25 | 14119.47 | 0.123 | 0.118 | 11.305 | 10539 | 11009.33 | 0.129 | 0.129 | 4.2721 |
| 418 | 11736.19 | 12183.21 | 0.122 | 0.124 | 3.6692 | 11190.69 | 11255.75 | 0.127 | 0.127 | 0.578 |
| 419 | 12634.19 | 13389.64 | 0.107 | 0.116 | 5.642 | 11655.43 | 11732.42 | 0.123 | 0.12 | 0.6562 |
| 420 | 11849.72 | 12757.58 | 0.121 | 0.115 | 7.1162 | 11438.77 | 11213.24 | 0.122 | 0.121 | -2.011 |
| 421 | 14124.49 | 14928.86 | 0.126 | 0.127 | 5.388 | 11135.63 | 11415.46 | 0.13 | 0.13 | 2.4513 |
| 422 | 11263.41 | 12047.88 | 0.145 | 0.147 | 6.5113 | 12953.67 | 13114.79 | 0.119 | 0.119 | 1.2286 |
| 423 | 12968.49 | 13112.93 | 0.139 | 0.142 | 1.1015 | 12736.34 | 12812.19 | 0.125 | 0.126 | 0.5921 |
| 424 | 13789.63 | 13805.49 | 0.14 | 0.14 | 0.1149 | 12431.2 | 12674.3 | 0.126 | 0.125 | 1.9181 |
| 425 | 11298.29 | 11765.61 | 0.131 | 0.13 | 3.9719 | 11822.71 | 11940.91 | 0.119 | 0.119 | 0.9899 |
| 426 | 10995.3 | 12313.94 | 0.136 | 0.129 | 10.708 | 15796.96 | 17256.84 | 0.137 | 0.134 | 8.4597 |
| 427 | 11588.56 | 11416.17 | 0.138 | 0.14 | -1.51 | 12052.59 | 11933.03 | 0.128 | 0.122 | -1.002 |
| 428 | 12249.42 | 16316.82 | 0.137 | 0.131 | 24.928 | 11294.84 | 11302.42 | 0.124 | 0.125 | 0.067 |
| 429 | 10673.2 | 10942.95 | 0.118 | 0.124 | 2.465 | 15308.56 | 15571.66 | 0.127 | 0.126 | 1.6896 |
| 430 | 12309.37 | 12763.87 | 0.117 | 0.114 | 3.5609 | 13677.24 | 13652.18 | 0.123 | 0.12 | -0.184 |
| 431 | 12025.97 | 12121.24 | 0.135 | 0.139 | 0.786 | 11186.17 | 11258.8 | 0.128 | 0.129 | 0.6451 |
| 432 | 13013.52 | 16148.74 | 0.113 | 0.111 | 19.415 | 13504.83 | 13643.7 | 0.118 | 0.117 | 1.0179 |
| 433 | 11848.24 | 13391.99 | 0.125 | 0.139 | 11.527 | 11747.54 | 12073.14 | 0.122 | 0.125 | 2.6969 |
| 434 | 11825.97 | 11947.65 | 0.123 | 0.129 | 1.0184 | 10619.21 | 11646.4 | 0.134 | 0.134 | 8.8198 |
| 435 | 11446 | 11497.17 | 0.122 | 0.129 | 0.4451 | 12584.69 | 13021.52 | 0.124 | 0.124 | 3.3547 |
| 436 | 13747.28 | 13643.1 | 0.104 | 0.115 | -0.764 | 11805.1 | 11981.08 | 0.125 | 0.131 | 1.4688 |
| 437 | 15957.27 | 15819.61 | 0.128 | 0.134 | -0.87 | 12546.48 | 12767.91 | 0.128 | 0.127 | 1.7342 |
| 438 | 11966.92 | 13432.06 | 0.117 | 0.12 | 10.908 | 10992.97 | 11167.74 | 0.128 | 0.128 | 1.5649 |
| 439 | 12981.12 | 13724.13 | 0.108 | 0.118 | 5.4139 | 12786.65 | 12851.71 | 0.121 | 0.121 | 0.5062 |
| 440 | 17085.28 | 17212.31 | 0.127 | 0.134 | 0.738 | 13561.97 | 13907.54 | 0.127 | 0.129 | 2.4847 |
| 441 | 12878.31 | 13803.13 | 0.106 | 0.118 | 6.7001 | 11437.03 | 11685.55 | 0.128 | 0.127 | 2.1268 |
| 442 | 11838.99 | 12392.8 | 0.107 | 0.121 | 4.4688 | 12608.09 | 12599.3 | 0.129 | 0.129 | -0.07 |
| 443 | 10814.89 | 11076.4 | 0.144 | 0.141 | 2.3609 | 11090.81 | 11212.74 | 0.127 | 0.124 | 1.0874 |
| 444 | 13069.89 | 13196.04 | 0.138 | 0.141 | 0.956 | 12618.08 | 12761.89 | 0.121 | 0.117 | 1.1269 |
| 445 | 12655.5 | 14478.43 | 0.115 | 0.117 | 12.591 | 11761.03 | 11836.39 | 0.125 | 0.125 | 0.6366 |
| 446 | 12690.78 | 13478.37 | 0.13 | 0.133 | 5.8433 | 13300.25 | 13584.72 | 0.119 | 0.123 | 2.0941 |
| 447 | 11973.27 | 12529.11 | 0.134 | 0.131 | 4.4364 | 12444.42 | 12533.3 | 0.122 | 0.125 | 0.7091 |
| 448 | 11624.01 | 12566.89 | 0.124 | 0.122 | 7.5029 | 11959.04 | 12524.76 | 0.133 | 0.134 | 4.5168 |

| 449 | 12490.05 | 12521.77 | 0.14 | 0.14 | 0.2533 | 11279.15 | 12043.61 | 0.136 | 0.136 | 6.3474 |
| --- | --- | --- | --- | --- | --- | --- | --- | --- | --- | --- |
| 450 | 12203.21 | 12683.3 | 0.123 | 0.126 | 3.7852 | 12150.31 | 12436.88 | 0.123 | 0.125 | 2.3042 |
| 451 | 11698.83 | 12290.2 | 0.145 | 0.139 | 4.8117 | 11820.81 | 12574.94 | 0.127 | 0.126 | 5.9971 |
| 452 | 11519.51 | 12040.47 | 0.146 | 0.146 | 4.3267 | 11098.77 | 11163.83 | 0.123 | 0.123 | 0.5828 |
| 453 | 12827.1 | 14221.69 | 0.14 | 0.132 | 9.8061 | 10551.78 | 11023.45 | 0.133 | 0.131 | 4.2787 |
| 454 | 12624.4 | 12955.86 | 0.136 | 0.133 | 2.5584 | 12610.9 | 13891.78 | 0.138 | 0.137 | 9.2204 |
| 455 | 11028.29 | 11643.84 | 0.134 | 0.131 | 5.2865 | 12939.98 | 13227.88 | 0.119 | 0.123 | 2.1765 |
| 456 | 11616.84 | 12440.19 | 0.137 | 0.13 | 6.6184 | 11442.8 | 11725.35 | 0.133 | 0.132 | 2.4097 |
| 457 | 13505.33 | 13784.71 | 0.133 | 0.135 | 2.0267 | 14083.71 | 14272.92 | 0.121 | 0.122 | 1.3256 |
| 458 | 11200.16 | 13053.84 | 0.13 | 0.139 | 14.2 | 10957.44 | 11283.13 | 0.139 | 0.135 | 2.8865 |
| 459 | 12824.46 | 12808.4 | 0.14 | 0.14 | -0.125 | 10888.65 | 11125.75 | 0.132 | 0.132 | 2.1312 |
| 460 | 14285.65 | 14690.97 | 0.122 | 0.128 | 2.759 | 13184.55 | 13302.75 | 0.116 | 0.117 | 0.8885 |
| 461 | 11430.13 | 12080.67 | 0.149 | 0.149 | 5.385 | 10940.28 | 10946.13 | 0.129 | 0.129 | 0.0535 |
| 462 | 11937.77 | 12752.04 | 0.126 | 0.138 | 6.3854 | 13119.41 | 13301.96 | 0.128 | 0.13 | 1.3723 |
| 463 | 12564.01 | 12876.02 | 0.127 | 0.131 | 2.4232 | 10962.73 | 11620.04 | 0.132 | 0.132 | 5.6567 |
| 464 | 10972.07 | 11324.65 | 0.148 | 0.145 | 3.1134 | 15579.04 | 15904.65 | 0.129 | 0.127 | 2.0473 |
| 465 | 13927.47 | 13854.51 | 0.123 | 0.131 | -0.527 | 11553.46 | 11652.96 | 0.127 | 0.122 | 0.8538 |
| 466 | 13064.6 | 13392.33 | 0.141 | 0.14 | 2.4472 | 14730 | 14713.72 | 0.132 | 0.133 | -0.111 |
| 467 | 11187.22 | 12134.75 | 0.132 | 0.127 | 7.8083 | 11831.99 | 11998.97 | 0.125 | 0.126 | 1.3917 |
| 468 | 10572.19 | 10958.2 | 0.15 | 0.148 | 3.5226 | 14857.79 | 15190.89 | 0.128 | 0.126 | 2.1928 |
| 469 | 12345.8 | 13411.86 | 0.136 | 0.136 | 7.9487 | 14815.89 | 15824.61 | 0.138 | 0.143 | 6.3743 |
| 470 | 11017.6 | 11403.91 | 0.145 | 0.149 | 3.3875 | 13421.01 | 12255.7 | 0.116 | 0.119 | -9.508 |
| 471 | 12398.57 | 12199.37 | 0.113 | 0.12 | -1.633 | 12023.51 | 12422.25 | 0.133 | 0.132 | 3.2099 |
| 472 | 13101.64 | 13223.36 | 0.136 | 0.14 | 0.9205 | 11065.86 | 11053.44 | 0.123 | 0.123 | -0.112 |
| 473 | 11157.87 | 12523.42 | 0.126 | 0.126 | 10.904 | 13300.82 | 14955.18 | 0.134 | 0.138 | 11.062 |
| 474 | 11284.63 | 12514.45 | 0.142 | 0.148 | 9.8273 | 11419.97 | 11516.24 | 0.124 | 0.126 | 0.836 |
| 475 | 11385.42 | 11622.53 | 0.132 | 0.131 | 2.0401 | 14652.33 | 17213.12 | 0.124 | 0.124 | 14.877 |
| 476 | 12524.26 | 12851.5 | 0.111 | 0.122 | 2.5463 | 12204.97 | 12529.85 | 0.132 | 0.131 | 2.5929 |
| 477 | 15958.1 | 16321.95 | 0.13 | 0.124 | 2.2292 | 13340.4 | 13793.04 | 0.12 | 0.124 | 3.2816 |
| 478 | 13655.37 | 13773.44 | 0.13 | 0.134 | 0.8573 | 13096.42 | 13373.66 | 0.127 | 0.126 | 2.0731 |
| 479 | 12380.91 | 13188.07 | 0.11 | 0.119 | 6.1204 | 12180.92 | 12398.11 | 0.116 | 0.121 | 1.7518 |
| 480 | 12047.47 | 13620.76 | 0.129 | 0.126 | 11.551 | 11745.3 | 12084.83 | 0.13 | 0.124 | 2.8096 |
| 481 | 13252.85 | 14313.79 | 0.126 | 0.13 | 7.412 | 12798.13 | 13216.57 | 0.129 | 0.126 | 3.166 |
| 482 | 12413.41 | 12984.29 | 0.137 | 0.135 | 4.3967 | 15222.34 | 16605.73 | 0.131 | 0.132 | 8.3308 |
| 483 | 12343.21 | 13768.52 | 0.138 | 0.133 | 10.352 | 11091.65 | 11109.43 | 0.12 | 0.121 | 0.1601 |
| 484 | 16395.51 | 16871.92 | 0.123 | 0.129 | 2.8237 | 12992.01 | 12749.17 | 0.119 | 0.117 | -1.905 |
| 485 | 13836.7 | 13840.84 | 0.133 | 0.134 | 0.0299 | 12991.7 | 12929.62 | 0.125 | 0.123 | -0.48 |
| 486 | 11892.11 | 11896.25 | 0.143 | 0.144 | 0.0348 | 13222.02 | 13315.78 | 0.123 | 0.127 | 0.7041 |
| 487 | 12531.15 | 12385.68 | 0.119 | 0.125 | -1.175 | 11586.09 | 11642.16 | 0.121 | 0.123 | 0.4816 |
| 488 | 12295.03 | 13966.36 | 0.112 | 0.109 | 11.967 | 13406.46 | 13498.89 | 0.124 | 0.126 | 0.6847 |
| 489 | 13060.06 | 12978.43 | 0.134 | 0.136 | -0.629 | 12788.28 | 12853.34 | 0.117 | 0.117 | 0.5062 |
| 490 | 13505.77 | 14272.46 | 0.136 | 0.132 | 5.3718 | 10920.57 | 11138.89 | 0.132 | 0.129 | 1.96 |
| 491 | 13599.06 | 15583.65 | 0.138 | 0.13 | 12.735 | 13912.51 | 14135.73 | 0.126 | 0.124 | 1.5791 |
| 492 | 12354.27 | 12677.58 | 0.135 | 0.139 | 2.5502 | 16150.51 | 17229.56 | 0.129 | 0.131 | 6.2628 |
| 493 | 15135.56 | 15488.66 | 0.131 | 0.133 | 2.2797 | 12078.31 | 12181.24 | 0.124 | 0.12 | 0.845 |
| 494 | 12592.75 | 13393.84 | 0.109 | 0.119 | 5.981 | 14496.75 | 14508.46 | 0.122 | 0.121 | 0.0808 |
| 495 | 12662.96 | 14166.21 | 0.116 | 0.117 | 10.612 | 11735.64 | 11861.2 | 0.121 | 0.121 | 1.0586 |
| 496 | 13138.2 | 14804.18 | 0.141 | 0.145 | 11.253 | 12558.21 | 12756.12 | 0.124 | 0.128 | 1.5514 |
| 497 | 11123.06 | 11354.22 | 0.123 | 0.126 | 2.0359 | 11783.46 | 11882.96 | 0.122 | 0.117 | 0.8373 |
| 498 | 15852.89 | 15536.65 | 0.125 | 0.131 | -2.035 | 12280.03 | 12464 | 0.119 | 0.122 | 1.476 |

| 499 | 12709.35 | 13205.21 | 0.134 | 0.139 | 3.755 | 13323.93 | 13388.99 | 0.122 | 0.122 | 0.4859 |
| --- | --- | --- | --- | --- | --- | --- | --- | --- | --- | --- |
| 500 | 12172.71 | 12375.64 | 0.136 | 0.134 | 1.6397 | 10898.34 | 11883.21 | 0.14 | 0.139 | 8.2879 |
| 501 | 11090.42 | 11528.86 | 0.134 | 0.129 | 3.803 | 11421 | 11718.52 | 0.124 | 0.128 | 2.5389 |
| 502 | 14484.13 | 15997.56 | 0.127 | 0.131 | 9.4604 | 10688.94 | 11137.88 | 0.131 | 0.134 | 4.0308 |
| 503 | 11011.31 | 11430.75 | 0.148 | 0.148 | 3.6695 | 15130.41 | 16170.59 | 0.128 | 0.134 | 6.4325 |
| 504 | 13481.52 | 14113.82 | 0.117 | 0.123 | 4.48 | 14039.33 | 14078.82 | 0.133 | 0.132 | 0.2805 |
| 505 | 12124.48 | 12376.44 | 0.141 | 0.141 | 2.0358 | 12862.92 | 12927.98 | 0.121 | 0.122 | 0.5033 |
| 506 | 11167.56 | 15131.36 | 0.143 | 0.141 | 26.196 | 11026.03 | 11649.92 | 0.12 | 0.119 | 5.3553 |
| 507 | 16177.7 | 16633.4 | 0.124 | 0.129 | 2.7397 | 10793.42 | 10868.78 | 0.126 | 0.127 | 0.6933 |
| 508 | 10443.64 | 10724.59 | 0.145 | 0.143 | 2.6197 | 14918.93 | 14898.72 | 0.114 | 0.118 | -0.136 |
| 509 | 11402.49 | 12082.32 | 0.149 | 0.149 | 5.6266 | 11237.76 | 11332.53 | 0.128 | 0.128 | 0.8362 |
| 510 | 13448.16 | 13453.19 | 0.107 | 0.117 | 0.0374 | 11904.64 | 12022.83 | 0.12 | 0.12 | 0.9831 |
| 511 | 17595.27 | 18197.81 | 0.127 | 0.122 | 3.3111 | 10396.72 | 11514.24 | 0.128 | 0.129 | 9.7056 |
| 512 | 16754.94 | 16762.25 | 0.12 | 0.131 | 0.0436 | 12534.5 | 12725.54 | 0.128 | 0.127 | 1.5012 |
| 513 | 10608.91 | 10677.49 | 0.115 | 0.122 | 0.6423 | 12131.53 | 12294.93 | 0.12 | 0.113 | 1.329 |
| 514 | 11599.64 | 11613.28 | 0.142 | 0.144 | 0.1174 | 15995.33 | 17092.81 | 0.132 | 0.13 | 6.4208 |
| 515 | 13404.17 | 13884.59 | 0.135 | 0.132 | 3.4601 | 11446.27 | 12222.66 | 0.135 | 0.132 | 6.3521 |
| 516 | 12404.56 | 12430.14 | 0.117 | 0.117 | 0.2058 | 12562.13 | 12598.91 | 0.122 | 0.119 | 0.2919 |
| 517 | 13863.87 | 14370.82 | 0.131 | 0.133 | 3.5276 | 13008.08 | 13779.71 | 0.139 | 0.138 | 5.5998 |
| 518 | 14536.59 | 15277.01 | 0.13 | 0.133 | 4.8466 | 12187.51 | 12201.24 | 0.123 | 0.117 | 0.1125 |
| 519 | 13333.95 | 14574.2 | 0.122 | 0.125 | 8.51 | 11867.62 | 12102.44 | 0.121 | 0.124 | 1.9402 |
| 520 | 13850.48 | 15050.95 | 0.113 | 0.117 | 7.976 | 12234.93 | 12353.13 | 0.118 | 0.119 | 0.9568 |
| 521 | 12657.72 | 13460.95 | 0.115 | 0.125 | 5.9671 | 11575.9 | 11717.23 | 0.129 | 0.13 | 1.2062 |
| 522 | 11511.56 | 11979.34 | 0.14 | 0.136 | 3.9049 | 12249.45 | 12189.87 | 0.127 | 0.129 | -0.489 |
| 523 | 13074.93 | 13241.58 | 0.132 | 0.137 | 1.2586 | 11948.71 | 11723.18 | 0.121 | 0.12 | -1.924 |
| 524 | 11959.22 | 12401.09 | 0.144 | 0.145 | 3.5632 | 11803.07 | 11922.98 | 0.126 | 0.125 | 1.0057 |
| 525 | 12673.66 | 13347.63 | 0.145 | 0.146 | 5.0494 | 13372.88 | 13415.6 | 0.121 | 0.122 | 0.3184 |
| 526 | 12285.45 | 13055.88 | 0.131 | 0.139 | 5.901 | 13622.4 | 13826.37 | 0.128 | 0.127 | 1.4752 |
| 527 | 12247.04 | 13463.02 | 0.141 | 0.148 | 9.032 | 12803.23 | 12902.72 | 0.123 | 0.119 | 0.7711 |
| 528 | 13915.14 | 15830.01 | 0.136 | 0.138 | 12.096 | 11458.45 | 11921.5 | 0.14 | 0.136 | 3.8842 |
| 529 | 16773.32 | 17549.59 | 0.122 | 0.131 | 4.4233 | 11509.75 | 11562.38 | 0.118 | 0.119 | 0.4552 |
| 530 | 11529.77 | 12266.38 | 0.148 | 0.148 | 6.0051 | 14363.64 | 14583.52 | 0.128 | 0.128 | 1.5077 |
| 531 | 12056.26 | 13080.38 | 0.122 | 0.121 | 7.8295 | 16234.84 | 16584.3 | 0.131 | 0.129 | 2.1072 |
| 532 | 12629.79 | 14706.36 | 0.139 | 0.144 | 14.12 | 13415.4 | 13405.11 | 0.116 | 0.122 | -0.077 |
| 533 | 14180.59 | 14714.02 | 0.132 | 0.134 | 3.6253 | 12543.23 | 12411.34 | 0.124 | 0.121 | -1.063 |
| 534 | 12655.4 | 16652.89 | 0.135 | 0.131 | 24.005 | 11083.54 | 11183.04 | 0.127 | 0.123 | 0.8897 |
| 535 | 12700.71 | 13116.24 | 0.126 | 0.126 | 3.168 | 11343.53 | 11432.53 | 0.127 | 0.127 | 0.7784 |
| 536 | 11687.7 | 14299.06 | 0.128 | 0.141 | 18.262 | 11250.73 | 11289.72 | 0.126 | 0.121 | 0.3454 |
| 537 | 13784.15 | 13764.66 | 0.136 | 0.136 | -0.142 | 11931.85 | 11942.65 | 0.124 | 0.122 | 0.0904 |
| 538 | 11592.6 | 12832.6 | 0.139 | 0.137 | 9.6629 | 11231.9 | 11284.54 | 0.122 | 0.122 | 0.4664 |
| 539 | 13232.67 | 13195.24 | 0.114 | 0.112 | -0.284 | 11726.63 | 11893.62 | 0.124 | 0.125 | 1.404 |
| 540 | 12499.66 | 12503.8 | 0.142 | 0.142 | 0.0331 | 12500.6 | 12930.72 | 0.124 | 0.125 | 3.3264 |
| 541 | 13321.23 | 13439.3 | 0.134 | 0.138 | 0.8786 | 12852 | 12925.01 | 0.122 | 0.124 | 0.5649 |
| 542 | 11107.85 | 11547 | 0.124 | 0.12 | 3.8032 | 12302.77 | 12545.03 | 0.121 | 0.125 | 1.9311 |
| 543 | 12792.07 | 12867.13 | 0.142 | 0.142 | 0.5834 | 12672.01 | 12911.21 | 0.134 | 0.133 | 1.8527 |
| 544 | 12534.95 | 12542.82 | 0.127 | 0.128 | 0.0627 | 11883.26 | 11912.26 | 0.125 | 0.125 | 0.2434 |
| 545 | 11834.64 | 12641.79 | 0.111 | 0.121 | 6.3848 | 15097.63 | 16126.47 | 0.119 | 0.124 | 6.3798 |
| 546 | 12618.43 | 13003.96 | 0.135 | 0.137 | 2.9647 | 12010.43 | 12172.77 | 0.121 | 0.12 | 1.3336 |
| 547 | 15060.65 | 15618.72 | 0.128 | 0.13 | 3.5731 | 12523.61 | 12765.03 | 0.13 | 0.129 | 1.8913 |
| 548 | 10967.94 | 14276.86 | 0.127 | 0.14 | 23.177 | 15028.26 | 15199.05 | 0.129 | 0.129 | 1.1237 |

| 549 | 16348.64 | 16784.29 | 0.125 | 0.123 | 2.5956 | 16356.28 | 16385.57 | 0.129 | 0.128 | 0.1788 |
| --- | --- | --- | --- | --- | --- | --- | --- | --- | --- | --- |
| 550 | 12174.08 | 13134.46 | 0.116 | 0.125 | 7.3119 | 10953.88 | 11103.79 | 0.134 | 0.134 | 1.3501 |
| 551 | 11430.85 | 12833.99 | 0.115 | 0.117 | 10.933 | 11576.5 | 11665.91 | 0.126 | 0.116 | 0.7664 |
| 552 | 11772.7 | 12129.51 | 0.145 | 0.146 | 2.9417 | 10335.89 | 11046.38 | 0.141 | 0.139 | 6.4319 |
| 553 | 12534.72 | 12907.06 | 0.113 | 0.124 | 2.8848 | 12828.97 | 12871.69 | 0.123 | 0.123 | 0.3319 |
| 554 | 14778.46 | 14862.36 | 0.121 | 0.128 | 0.5645 | 15499.2 | 15404.97 | 0.125 | 0.128 | -0.612 |
| 555 | 11805.41 | 11805.41 | 0.144 | 0.144 | 0 | 11911.74 | 12118.23 | 0.121 | 0.126 | 1.7039 |
| 556 | 11968.4 | 13141.66 | 0.123 | 0.12 | 8.9278 | 10368.11 | 10779.48 | 0.14 | 0.14 | 3.8162 |
| 557 | 13522.17 | 14126.73 | 0.132 | 0.138 | 4.2795 | 11995.6 | 12177.11 | 0.123 | 0.125 | 1.4906 |
| 558 | 10681.15 | 11004.03 | 0.147 | 0.145 | 2.9342 | 11082.29 | 12173.35 | 0.139 | 0.139 | 8.9627 |
| 559 | 11468.35 | 12429.97 | 0.135 | 0.123 | 7.7363 | 11287.91 | 11532.38 | 0.137 | 0.139 | 2.1199 |
| 560 | 14338.04 | 14433.48 | 0.133 | 0.136 | 0.6613 | 11552.92 | 11671.12 | 0.122 | 0.123 | 1.0127 |
| 561 | 11356.39 | 11633.2 | 0.142 | 0.14 | 2.3795 | 10920.86 | 11078.77 | 0.131 | 0.132 | 1.4253 |
| 562 | 15785.8 | 16055.27 | 0.125 | 0.132 | 1.6783 | 13261.56 | 13607.12 | 0.126 | 0.129 | 2.5396 |
| 563 | 11176.22 | 11482.74 | 0.146 | 0.144 | 2.6694 | 11557.5 | 11675.7 | 0.122 | 0.123 | 1.0123 |
| 564 | 11682.33 | 13495.8 | 0.115 | 0.112 | 13.437 | 9907.128 | 11284.81 | 0.138 | 0.138 | 12.208 |
| 565 | 11393.2 | 11955.29 | 0.123 | 0.129 | 4.7015 | 11969.2 | 12081.72 | 0.127 | 0.125 | 0.9313 |
| 566 | 15887.84 | 16149.05 | 0.133 | 0.134 | 1.6175 | 12107.38 | 12423.9 | 0.132 | 0.129 | 2.5477 |
| 567 | 12281.23 | 12602.36 | 0.136 | 0.131 | 2.5482 | 13663 | 13817.73 | 0.126 | 0.12 | 1.1198 |
| 568 | 12791.89 | 14425.65 | 0.142 | 0.145 | 11.325 | 11415.82 | 11741.42 | 0.123 | 0.127 | 2.7731 |
| 569 | 11824.54 | 12503.86 | 0.148 | 0.147 | 5.4329 | 12483.1 | 12610.09 | 0.139 | 0.137 | 1.007 |
| 570 | 15881.89 | 16621.68 | 0.129 | 0.129 | 4.4508 | 10804.12 | 11244.07 | 0.134 | 0.131 | 3.9127 |
| 571 | 11066.28 | 12275.4 | 0.142 | 0.149 | 9.8499 | 11878.15 | 12015.64 | 0.121 | 0.12 | 1.1442 |
| 572 | 12993.58 | 13007.22 | 0.137 | 0.139 | 0.1049 | 11188.18 | 11253.24 | 0.121 | 0.122 | 0.5782 |
| 573 | 17040.41 | 17719.32 | 0.124 | 0.122 | 3.8315 | 14207.94 | 14281.91 | 0.12 | 0.124 | 0.5179 |
| 574 | 12456.03 | 15349.92 | 0.114 | 0.111 | 18.853 | 12672.33 | 12912.45 | 0.121 | 0.124 | 1.8596 |
| 575 | 12328.72 | 12852.31 | 0.121 | 0.118 | 4.0739 | 14731.4 | 14837.17 | 0.139 | 0.137 | 0.7129 |
| 576 | 11613.62 | 12107.3 | 0.137 | 0.134 | 4.0775 | 11578.01 | 12069.88 | 0.12 | 0.127 | 4.0752 |
| 577 | 12527.63 | 13013.43 | 0.14 | 0.142 | 3.7331 | 11028.83 | 11891.24 | 0.127 | 0.124 | 7.2525 |
| 578 | 11270.73 | 12303.99 | 0.136 | 0.132 | 8.3978 | 14099.95 | 14140.87 | 0.12 | 0.118 | 0.2894 |
| 579 | 11743.14 | 15355.25 | 0.135 | 0.14 | 23.524 | 14219.48 | 14334.93 | 0.123 | 0.122 | 0.8053 |
| 580 | 12329.38 | 12888.49 | 0.134 | 0.131 | 4.3381 | 14205.37 | 14442.47 | 0.125 | 0.123 | 1.6417 |
| 581 | 13812.78 | 14435.97 | 0.124 | 0.131 | 4.3169 | 10872.22 | 11106.19 | 0.131 | 0.13 | 2.1067 |
| 582 | 11540.48 | 12919.55 | 0.132 | 0.131 | 10.674 | 9966.747 | 10308.91 | 0.127 | 0.129 | 3.3191 |
| 583 | 13419.41 | 14057.83 | 0.124 | 0.139 | 4.5414 | 11586.45 | 11725.32 | 0.125 | 0.124 | 1.1844 |
| 584 | 11749.96 | 11951.79 | 0.136 | 0.132 | 1.6888 | 12757.56 | 13121.33 | 0.126 | 0.126 | 2.7723 |
| 585 | 12765.78 | 15882.06 | 0.135 | 0.136 | 19.621 | 11666.24 | 11690.68 | 0.128 | 0.127 | 0.209 |
| 586 | 13943.04 | 14079.19 | 0.137 | 0.141 | 0.967 | 15160.55 | 15373.01 | 0.122 | 0.121 | 1.382 |
| 587 | 11820.24 | 13832.25 | 0.143 | 0.148 | 14.546 | 12470.39 | 12569.47 | 0.127 | 0.121 | 0.7883 |
| 588 | 11523.77 | 12393.04 | 0.133 | 0.124 | 7.0142 | 12082.7 | 12182.19 | 0.124 | 0.12 | 0.8167 |
| 589 | 17123.05 | 17672.75 | 0.124 | 0.122 | 3.1105 | 12203.72 | 12470.53 | 0.13 | 0.128 | 2.1395 |
| 590 | 12560.09 | 14663.05 | 0.108 | 0.111 | 14.342 | 10584.48 | 10743.35 | 0.127 | 0.126 | 1.4788 |
| 591 | 12994.18 | 13209.17 | 0.113 | 0.11 | 1.6276 | 12373.52 | 13081.23 | 0.122 | 0.123 | 5.4101 |
| 592 | 11942.19 | 12199.68 | 0.143 | 0.144 | 2.1106 | 11542.67 | 12575.74 | 0.141 | 0.14 | 8.2148 |
| 593 | 11957.69 | 11999.11 | 0.144 | 0.145 | 0.3452 | 15272.94 | 15748.85 | 0.132 | 0.129 | 3.0219 |
| 594 | 12943.41 | 13280.62 | 0.127 | 0.139 | 2.5391 | 11340.46 | 11554.64 | 0.131 | 0.13 | 1.8536 |
| 595 | 12075.3 | 12473.24 | 0.143 | 0.141 | 3.1903 | 12654.82 | 12757.75 | 0.123 | 0.119 | 0.8068 |
| 596 | 11302.69 | 11779.92 | 0.15 | 0.147 | 4.0512 | 10209.09 | 10431.13 | 0.137 | 0.141 | 2.1287 |
| 597 | 14320.07 | 14922.88 | 0.134 | 0.133 | 4.0395 | 11406.07 | 11381.22 | 0.123 | 0.123 | -0.218 |
| 598 | 13920.26 | 14693.29 | 0.121 | 0.123 | 5.2611 | 11760.03 | 11528.57 | 0.124 | 0.12 | -2.008 |

| 599 | 13174.79 | 13586.04 | 0.135 | 0.132 | 3.027 | 12757.41 | 13176.29 | 0.131 | 0.128 | 3.179 |
| --- | --- | --- | --- | --- | --- | --- | --- | --- | --- | --- |
| 600 | 13202.07 | 14766.37 | 0.123 | 0.116 | 10.594 | 11652.3 | 11618.15 | 0.116 | 0.116 | -0.294 |
| 601 | 10772.14 | 10784.56 | 0.143 | 0.143 | 0.1152 | 15957.5 | 16134.95 | 0.13 | 0.129 | 1.0998 |
| 602 | 16461.06 | 17148.55 | 0.124 | 0.12 | 4.009 | 12401.79 | 12640.87 | 0.126 | 0.128 | 1.8913 |
| 603 | 11751.55 | 12470.37 | 0.145 | 0.145 | 5.7642 | 12356.84 | 12396.75 | 0.127 | 0.127 | 0.322 |
| 604 | 12328.33 | 13079.78 | 0.141 | 0.142 | 5.7452 | 15041.95 | 16286.43 | 0.125 | 0.128 | 7.6412 |
| 605 | 12753.06 | 13075.49 | 0.132 | 0.133 | 2.4659 | 12413.48 | 12692.12 | 0.121 | 0.116 | 2.1954 |
| 606 | 13297.69 | 13451.33 | 0.113 | 0.123 | 1.1422 | 13256.19 | 13230.12 | 0.122 | 0.121 | -0.197 |
| 607 | 10697.35 | 14241.63 | 0.128 | 0.141 | 24.887 | 14362.5 | 15744.47 | 0.127 | 0.129 | 8.7775 |
| 608 | 12369.06 | 12369.06 | 0.139 | 0.139 | 0 | 11871.99 | 12005 | 0.13 | 0.129 | 1.108 |
| 609 | 14536.84 | 14301.32 | 0.124 | 0.132 | -1.647 | 11567.64 | 11542.79 | 0.124 | 0.124 | -0.215 |
| 610 | 11351.9 | 11590.4 | 0.12 | 0.129 | 2.0577 | 11094.93 | 11217.23 | 0.123 | 0.123 | 1.0903 |
| 611 | 13374.72 | 14767.6 | 0.118 | 0.126 | 9.432 | 11534.01 | 12187.27 | 0.131 | 0.129 | 5.3602 |
| 612 | 10462.63 | 10852.79 | 0.15 | 0.148 | 3.595 | 11154.04 | 11234.24 | 0.129 | 0.129 | 0.714 |
| 613 | 13199.83 | 13268.08 | 0.123 | 0.125 | 0.5144 | 12046.05 | 12218.8 | 0.13 | 0.129 | 1.4139 |
| 614 | 13005.8 | 15058.89 | 0.12 | 0.116 | 13.634 | 13482.62 | 13737.27 | 0.126 | 0.129 | 1.8537 |
| 615 | 11390.37 | 13494.17 | 0.125 | 0.143 | 15.59 | 10458.85 | 10756.46 | 0.137 | 0.14 | 2.7668 |
| 616 | 13583.11 | 13583.11 | 0.14 | 0.14 | 0 | 12312.48 | 12549.72 | 0.122 | 0.121 | 1.8904 |
| 617 | 14803.26 | 14834.94 | 0.124 | 0.132 | 0.2136 | 14499.61 | 14501.82 | 0.121 | 0.127 | 0.0153 |
| 618 | 11490.02 | 13010.68 | 0.138 | 0.133 | 11.688 | 11974.07 | 12103.66 | 0.124 | 0.122 | 1.0706 |
| 619 | 12044.17 | 11898.48 | 0.121 | 0.13 | -1.224 | 13983.11 | 13914.75 | 0.125 | 0.12 | -0.491 |
| 620 | 12887.06 | 12781.71 | 0.113 | 0.119 | -0.824 | 11329.37 | 11576.68 | 0.128 | 0.128 | 2.1363 |
| 621 | 11671.46 | 13531.38 | 0.136 | 0.146 | 13.745 | 15411.19 | 16664.93 | 0.128 | 0.137 | 7.5232 |
| 622 | 14594.56 | 15126.03 | 0.122 | 0.127 | 3.5136 | 10503.27 | 11438.1 | 0.129 | 0.124 | 8.173 |
| 623 | 13591.79 | 14907.52 | 0.134 | 0.135 | 8.826 | 12313.47 | 12087.95 | 0.118 | 0.117 | -1.866 |
| 624 | 12669.15 | 12940.66 | 0.13 | 0.13 | 2.0981 | 13485.58 | 13652.56 | 0.126 | 0.127 | 1.2231 |
| 625 | 12534.61 | 14921.88 | 0.124 | 0.117 | 15.998 | 11745.99 | 12072.59 | 0.133 | 0.132 | 2.7053 |
| 626 | 11454.43 | 11461.5 | 0.144 | 0.144 | 0.0617 | 12712.18 | 13151.52 | 0.13 | 0.128 | 3.3406 |
| 627 | 11189.37 | 12549.91 | 0.131 | 0.14 | 10.841 | 10952.77 | 11237.45 | 0.124 | 0.123 | 2.5333 |
| 628 | 13171.39 | 13564.4 | 0.131 | 0.133 | 2.8974 | 11639.7 | 12049.14 | 0.121 | 0.124 | 3.3981 |
| 629 | 10897.42 | 11233.44 | 0.15 | 0.147 | 2.9912 | 14882.3 | 16039.93 | 0.117 | 0.121 | 7.2171 |
| 630 | 11240.75 | 11283.68 | 0.12 | 0.125 | 0.3805 | 10578.68 | 10923.15 | 0.132 | 0.131 | 3.1536 |
| 631 | 13848.73 | 13186.63 | 0.136 | 0.139 | -5.021 | 12095.64 | 12198.69 | 0.118 | 0.12 | 0.8448 |
| 632 | 12646.8 | 13472.78 | 0.14 | 0.135 | 6.1307 | 12842.1 | 12701.06 | 0.122 | 0.115 | -1.11 |
| 633 | 11643.19 | 11778.13 | 0.122 | 0.126 | 1.1457 | 11656.47 | 11773.04 | 0.117 | 0.119 | 0.9901 |
| 634 | 11066.16 | 15656.37 | 0.135 | 0.144 | 29.319 | 14060.11 | 14296.47 | 0.132 | 0.135 | 1.6533 |
| 635 | 12946.69 | 14700.3 | 0.115 | 0.117 | 11.929 | 12780.83 | 13265.23 | 0.128 | 0.124 | 3.6517 |
| 636 | 13905.45 | 13185.58 | 0.132 | 0.136 | -5.46 | 12701.09 | 13005.57 | 0.117 | 0.116 | 2.3411 |
| 637 | 11201.88 | 11393.01 | 0.128 | 0.129 | 1.6776 | 12871.29 | 13151.33 | 0.118 | 0.119 | 2.1293 |
| 638 | 11000.89 | 11403.89 | 0.145 | 0.144 | 3.5339 | 12089.05 | 12178.96 | 0.128 | 0.125 | 0.7383 |
| 639 | 14606.74 | 14737.03 | 0.133 | 0.137 | 0.8841 | 12844.76 | 12962.96 | 0.12 | 0.12 | 0.9118 |
| 640 | 10844.12 | 12349.51 | 0.14 | 0.147 | 12.19 | 12883.77 | 13142.94 | 0.134 | 0.132 | 1.9719 |
| 641 | 11897.3 | 12507.21 | 0.137 | 0.133 | 4.8765 | 14340.08 | 15634.57 | 0.131 | 0.131 | 8.2796 |
| 642 | 11211.26 | 11539.75 | 0.119 | 0.127 | 2.8466 | 14117.19 | 14128.91 | 0.126 | 0.126 | 0.0829 |
| 643 | 13201.18 | 13364.23 | 0.137 | 0.137 | 1.2201 | 11285.31 | 11090.34 | 0.121 | 0.118 | -1.758 |
| 644 | 12222.56 | 13660.38 | 0.136 | 0.141 | 10.525 | 9968.524 | 9986.808 | 0.132 | 0.134 | 0.1831 |
| 645 | 14321.62 | 14325.76 | 0.137 | 0.138 | 0.0289 | 14981.1 | 15068.97 | 0.12 | 0.118 | 0.5831 |
| 646 | 12563.62 | 12362.87 | 0.12 | 0.125 | -1.624 | 11551.34 | 11538.91 | 0.121 | 0.121 | -0.108 |
| 647 | 13089.77 | 13187.48 | 0.118 | 0.115 | 0.7409 | 14419.23 | 15744.42 | 0.131 | 0.131 | 8.4169 |
| 648 | 12192.3 | 12337.03 | 0.109 | 0.121 | 1.1731 | 12672.58 | 12877.76 | 0.129 | 0.128 | 1.5933 |

| 649 | 13259.85 | 14577.67 | 0.121 | 0.117 | 9.04 | 11846.88 | 12011.35 | 0.126 | 0.126 | 1.3693 |
| --- | --- | --- | --- | --- | --- | --- | --- | --- | --- | --- |
| 650 | 11209.87 | 11611.88 | 0.111 | 0.121 | 3.4621 | 12397.55 | 12214.19 | 0.128 | 0.121 | -1.501 |
| 651 | 11036.9 | 11498.78 | 0.143 | 0.143 | 4.0167 | 15076.53 | 15056.74 | 0.117 | 0.117 | -0.131 |
| 652 | 10849.71 | 11003.14 | 0.119 | 0.127 | 1.3944 | 16189.47 | 17163.96 | 0.113 | 0.117 | 5.6775 |
| 653 | 12601.56 | 12437.38 | 0.117 | 0.124 | -1.32 | 11175.11 | 11274.61 | 0.124 | 0.119 | 0.8825 |
| 654 | 14861.96 | 15013.89 | 0.13 | 0.129 | 1.0119 | 11922.26 | 12010.54 | 0.125 | 0.123 | 0.7351 |
| 655 | 12266.39 | 12694.2 | 0.136 | 0.133 | 3.3702 | 12687.16 | 13406.78 | 0.128 | 0.128 | 5.3676 |
| 656 | 10747.42 | 11236.37 | 0.141 | 0.144 | 4.3514 | 12701.14 | 13059.08 | 0.128 | 0.125 | 2.7409 |
| 657 | 12345.24 | 14010.88 | 0.111 | 0.114 | 11.888 | 12997.17 | 13097.97 | 0.122 | 0.12 | 0.7696 |
| 658 | 12342.04 | 13998.61 | 0.144 | 0.147 | 11.834 | 16898.89 | 17076.34 | 0.129 | 0.129 | 1.0392 |
| 659 | 12429.82 | 12442.25 | 0.143 | 0.143 | 0.0999 | 13707.97 | 13900.01 | 0.128 | 0.13 | 1.3816 |
| 660 | 11946.6 | 11959.02 | 0.141 | 0.14 | 0.1039 | 12587.76 | 12561.69 | 0.12 | 0.118 | -0.208 |
| 661 | 11108.91 | 12546.09 | 0.136 | 0.132 | 11.455 | 13554.64 | 13867.03 | 0.126 | 0.124 | 2.2528 |
| 662 | 12110.13 | 12359.16 | 0.137 | 0.138 | 2.0149 | 11523.24 | 11588.3 | 0.123 | 0.123 | 0.5614 |
| 663 | 11566.84 | 11843.53 | 0.133 | 0.128 | 2.3362 | 11624.98 | 11399.45 | 0.122 | 0.121 | -1.978 |
| 664 | 13709.68 | 14157.76 | 0.133 | 0.137 | 3.1649 | 11700.92 | 11969.45 | 0.132 | 0.133 | 2.2434 |
| 665 | 12267.38 | 12657.63 | 0.137 | 0.142 | 3.0831 | 11526.85 | 12564.43 | 0.132 | 0.13 | 8.258 |
| 666 | 12182.5 | 12331.59 | 0.113 | 0.116 | 1.2089 | 10958.91 | 11572.04 | 0.123 | 0.12 | 5.2983 |
| 667 | 13528.95 | 13425.15 | 0.112 | 0.114 | -0.773 | 13255.02 | 13962.73 | 0.121 | 0.122 | 5.0686 |
| 668 | 12210.46 | 13794.97 | 0.137 | 0.131 | 11.486 | 13115.04 | 13250.98 | 0.125 | 0.125 | 1.0259 |
| 669 | 11266.61 | 14434.62 | 0.127 | 0.145 | 21.947 | 13115.63 | 13285.5 | 0.122 | 0.119 | 1.2787 |
| 670 | 12712.66 | 13956.63 | 0.122 | 0.115 | 8.9131 | 13161.3 | 13269.59 | 0.116 | 0.117 | 0.816 |
| 671 | 14312.61 | 13653.44 | 0.135 | 0.139 | -4.828 | 12343.74 | 12249.89 | 0.128 | 0.126 | -0.766 |
| 672 | 12955.83 | 13604 | 0.14 | 0.138 | 4.7645 | 11010.58 | 11018.15 | 0.124 | 0.125 | 0.0687 |
| 673 | 12744.5 | 12326.98 | 0.113 | 0.125 | -3.387 | 12076.47 | 12485.92 | 0.12 | 0.124 | 3.2793 |
| 674 | 12874.76 | 13819.11 | 0.112 | 0.12 | 6.8337 | 15192.13 | 16028.96 | 0.134 | 0.137 | 5.2207 |
| 675 | 10899.21 | 11647.36 | 0.135 | 0.131 | 6.4233 | 12432.18 | 12706.36 | 0.119 | 0.124 | 2.1578 |
| 676 | 11218.43 | 11858.79 | 0.147 | 0.143 | 5.3999 | 10862.31 | 11019.71 | 0.134 | 0.134 | 1.4284 |
| 677 | 10536.66 | 11123.47 | 0.146 | 0.147 | 5.2754 | 13233.31 | 13346.33 | 0.117 | 0.114 | 0.8468 |
| 678 | 11316.55 | 11785.2 | 0.123 | 0.121 | 3.9766 | 14949.01 | 15005.82 | 0.125 | 0.123 | 0.3786 |
| 679 | 12879.72 | 12922.86 | 0.12 | 0.121 | 0.3338 | 11514.14 | 11496.36 | 0.123 | 0.12 | -0.155 |
| 680 | 11996.46 | 12142.95 | 0.14 | 0.138 | 1.2063 | 10898.68 | 11272.18 | 0.12 | 0.126 | 3.3135 |
| 681 | 12476.66 | 13956.1 | 0.119 | 0.128 | 10.601 | 12682.91 | 12971.52 | 0.119 | 0.124 | 2.225 |
| 682 | 11687.97 | 13307.25 | 0.124 | 0.124 | 12.168 | 12890.53 | 13401.08 | 0.125 | 0.126 | 3.8098 |
| 683 | 13105.42 | 13654.71 | 0.131 | 0.132 | 4.0227 | 14120.77 | 14199.05 | 0.123 | 0.124 | 0.5513 |
| 684 | 11412.64 | 11832.09 | 0.146 | 0.146 | 3.545 | 11552.38 | 11639.96 | 0.119 | 0.121 | 0.7524 |
| 685 | 10874.33 | 14346.36 | 0.127 | 0.144 | 24.201 | 11288.79 | 11263.94 | 0.12 | 0.12 | -0.221 |
| 686 | 12292.55 | 13385.24 | 0.121 | 0.129 | 8.1634 | 11762.92 | 11878.28 | 0.122 | 0.119 | 0.9711 |
| 687 | 11416.52 | 11768.99 | 0.137 | 0.133 | 2.9948 | 12847.69 | 12965.88 | 0.119 | 0.12 | 0.9116 |
| 688 | 11930.81 | 12395.11 | 0.137 | 0.132 | 3.7458 | 11495.73 | 11662.88 | 0.126 | 0.124 | 1.4332 |
| 689 | 12452.85 | 12624.18 | 0.138 | 0.136 | 1.3572 | 10361.72 | 10503.06 | 0.126 | 0.127 | 1.3457 |
| 690 | 12651.99 | 15216.88 | 0.136 | 0.136 | 16.856 | 12078.77 | 11575.04 | 0.123 | 0.125 | -4.352 |
| 691 | 11439.12 | 12442.54 | 0.133 | 0.128 | 8.0644 | 11697.76 | 11941.52 | 0.126 | 0.124 | 2.0413 |
| 692 | 12207.93 | 11949.91 | 0.128 | 0.13 | -2.159 | 11969.13 | 12002.56 | 0.128 | 0.128 | 0.2785 |
| 693 | 11697.51 | 11483.04 | 0.125 | 0.129 | -1.868 | 12029.8 | 12431.02 | 0.127 | 0.128 | 3.2275 |
| 694 | 13480.51 | 13564.36 | 0.14 | 0.141 | 0.6181 | 11120.25 | 11237.95 | 0.131 | 0.129 | 1.0473 |
| 695 | 11945.48 | 12320.7 | 0.145 | 0.143 | 3.0454 | 12530.75 | 12703.59 | 0.126 | 0.127 | 1.3606 |
| 696 | 13438.63 | 13454.48 | 0.137 | 0.136 | 0.1179 | 12920.81 | 13019.89 | 0.126 | 0.12 | 0.761 |
| 697 | 12156.71 | 12920.38 | 0.142 | 0.142 | 5.9106 | 12597.14 | 13019.45 | 0.125 | 0.119 | 3.2436 |
| 698 | 14987.32 | 14625.93 | 0.127 | 0.137 | -2.471 | 12569.29 | 12708.16 | 0.122 | 0.121 | 1.0928 |

| 699 | 12607.72 | 13967.05 | 0.119 | 0.115 | 9.7324 | 11582.63 | 11796.23 | 0.123 | 0.115 | 1.8108 |
| --- | --- | --- | --- | --- | --- | --- | --- | --- | --- | --- |
| 700 | 13121.14 | 12727.97 | 0.127 | 0.13 | -3.089 | 11268.71 | 11321.34 | 0.121 | 0.122 | 0.4649 |
| 701 | 12415.36 | 14526.66 | 0.144 | 0.148 | 14.534 | 12061.64 | 12213.41 | 0.118 | 0.114 | 1.2426 |
| 702 | 13372.11 | 13776.84 | 0.113 | 0.122 | 2.9378 | 11337.75 | 11471.09 | 0.12 | 0.118 | 1.1624 |
| 703 | 11437.53 | 11519.12 | 0.12 | 0.126 | 0.7083 | 14829.58 | 14905.14 | 0.129 | 0.132 | 0.507 |
| 704 | 13920.61 | 14761.42 | 0.123 | 0.123 | 5.696 | 11603.6 | 12173.63 | 0.126 | 0.124 | 4.6825 |
| 705 | 11501.76 | 11902.26 | 0.118 | 0.123 | 3.3649 | 11560.64 | 11984.99 | 0.122 | 0.122 | 3.5407 |
| 706 | 11053.38 | 11347.47 | 0.147 | 0.146 | 2.5917 | 13189.15 | 13344.51 | 0.12 | 0.124 | 1.1642 |
| 707 | 11357.33 | 11527.63 | 0.121 | 0.125 | 1.4773 | 13244.72 | 13629.03 | 0.119 | 0.119 | 2.8198 |
| 708 | 12704.92 | 12575.88 | 0.118 | 0.124 | -1.026 | 12074.97 | 12415.42 | 0.116 | 0.121 | 2.7422 |
| 709 | 13927.51 | 15649.85 | 0.139 | 0.142 | 11.005 | 11249.19 | 11349.19 | 0.136 | 0.134 | 0.8811 |
| 710 | 12407.56 | 14219.92 | 0.134 | 0.136 | 12.745 | 14967.04 | 15116.63 | 0.127 | 0.127 | 0.9895 |
| 711 | 11286.72 | 12677.68 | 0.131 | 0.129 | 10.972 | 11067.45 | 11126.45 | 0.12 | 0.123 | 0.5302 |
| 712 | 12336 | 12730.94 | 0.117 | 0.126 | 3.1022 | 12423.61 | 12833.05 | 0.119 | 0.122 | 3.1906 |
| 713 | 15390.11 | 15704.25 | 0.129 | 0.127 | 2.0004 | 10743.95 | 10745.96 | 0.12 | 0.124 | 0.0187 |
| 714 | 10967.91 | 11382 | 0.133 | 0.128 | 3.6381 | 12958.33 | 13219.42 | 0.125 | 0.123 | 1.9751 |
| 715 | 12236.11 | 12845.35 | 0.146 | 0.145 | 4.7429 | 14301.17 | 13439.22 | 0.123 | 0.121 | -6.414 |
| 716 | 13931.82 | 14181.49 | 0.121 | 0.129 | 1.7605 | 11069.95 | 11291.28 | 0.132 | 0.134 | 1.9602 |
| 717 | 11385.87 | 11319.39 | 0.114 | 0.121 | -0.587 | 10739.31 | 10857.5 | 0.126 | 0.127 | 1.0886 |
| 718 | 12099.22 | 12199.93 | 0.109 | 0.118 | 0.8255 | 11661.05 | 12275.85 | 0.128 | 0.13 | 5.0082 |
| 719 | 11527.84 | 12149.7 | 0.128 | 0.131 | 5.1183 | 12547.97 | 12469.68 | 0.123 | 0.122 | -0.628 |
| 720 | 10164.22 | 10317.99 | 0.146 | 0.147 | 1.4902 | 13973.17 | 14391.8 | 0.13 | 0.128 | 2.9088 |
| 721 | 12812.51 | 13983.05 | 0.136 | 0.143 | 8.3711 | 13107.18 | 13156.76 | 0.119 | 0.122 | 0.3769 |
| 722 | 11547.11 | 11941.41 | 0.145 | 0.143 | 3.302 | 12734.94 | 12861.93 | 0.139 | 0.137 | 0.9873 |
| 723 | 11991.55 | 12718.66 | 0.146 | 0.145 | 5.7169 | 10485.45 | 10460.59 | 0.119 | 0.119 | -0.238 |
| 724 | 11821.68 | 12995.44 | 0.124 | 0.122 | 9.0321 | 11591.5 | 11641.2 | 0.125 | 0.12 | 0.427 |
| 725 | 14051.86 | 15032.29 | 0.131 | 0.132 | 6.5222 | 14603.76 | 15051.38 | 0.127 | 0.126 | 2.974 |
| 726 | 13125.77 | 14466.43 | 0.121 | 0.113 | 9.2674 | 11846.11 | 12135.94 | 0.126 | 0.128 | 2.3882 |
| 727 | 13241.33 | 13461.12 | 0.112 | 0.121 | 1.6328 | 11580.46 | 11816.44 | 0.125 | 0.128 | 1.997 |
| 728 | 11140.81 | 11166.97 | 0.144 | 0.143 | 0.2342 | 11554.93 | 11670.29 | 0.121 | 0.117 | 0.9885 |
| 729 | 10823.44 | 11226.02 | 0.147 | 0.147 | 3.5862 | 11648.63 | 11674.7 | 0.126 | 0.128 | 0.2233 |
| 730 | 12201.86 | 12777.1 | 0.115 | 0.125 | 4.5021 | 13348.86 | 13255.01 | 0.123 | 0.122 | -0.708 |
| 731 | 11274.72 | 11564.93 | 0.137 | 0.136 | 2.5094 | 11368.27 | 11550.52 | 0.132 | 0.129 | 1.5779 |
| 732 | 11269.63 | 13924.08 | 0.126 | 0.141 | 19.064 | 13601.21 | 13824.31 | 0.115 | 0.117 | 1.6138 |
| 733 | 12909.33 | 13041.34 | 0.139 | 0.142 | 1.0122 | 11561.19 | 11667.68 | 0.129 | 0.128 | 0.9126 |
| 734 | 12335.97 | 15567.76 | 0.113 | 0.112 | 20.759 | 11301.22 | 13390.36 | 0.139 | 0.14 | 15.602 |
| 735 | 11243.63 | 14997.22 | 0.144 | 0.141 | 25.029 | 12147.33 | 12006.29 | 0.126 | 0.118 | -1.175 |
| 736 | 13807.71 | 14410.26 | 0.133 | 0.134 | 4.1814 | 13272.95 | 13193.37 | 0.126 | 0.129 | -0.603 |
| 737 | 14947.29 | 15365.4 | 0.131 | 0.127 | 2.7211 | 13389.29 | 13849.34 | 0.128 | 0.126 | 3.3218 |
| 738 | 13526.2 | 13657.59 | 0.126 | 0.13 | 0.962 | 11613.57 | 11923.74 | 0.12 | 0.116 | 2.6013 |
| 739 | 10929.55 | 11210.51 | 0.146 | 0.144 | 2.5062 | 14671.24 | 14571.15 | 0.127 | 0.129 | -0.687 |
| 740 | 14084.4 | 14226.58 | 0.123 | 0.13 | 0.9994 | 11878.19 | 11981.12 | 0.12 | 0.116 | 0.8591 |
| 741 | 12171.12 | 14852.61 | 0.113 | 0.112 | 18.054 | 11118.27 | 11115.84 | 0.129 | 0.128 | -0.022 |
| 742 | 12750.84 | 13225.28 | 0.133 | 0.132 | 3.5873 | 10218.19 | 11057.17 | 0.127 | 0.126 | 7.5877 |
| 743 | 12537.07 | 13923.47 | 0.133 | 0.135 | 9.9573 | 12778.14 | 12969.18 | 0.126 | 0.126 | 1.473 |
| 744 | 13766.81 | 14285.06 | 0.114 | 0.115 | 3.6279 | 11651.56 | 11818.72 | 0.122 | 0.12 | 1.4143 |
| 745 | 12445.37 | 12788.13 | 0.131 | 0.133 | 2.6803 | 11437.91 | 11568.03 | 0.123 | 0.122 | 1.1248 |
| 746 | 12928.7 | 12950.88 | 0.116 | 0.127 | 0.1713 | 12869.3 | 13201.94 | 0.127 | 0.128 | 2.5196 |
| 747 | 14618.93 | 14928.81 | 0.13 | 0.131 | 2.0757 | 11270.84 | 11412.17 | 0.127 | 0.128 | 1.2385 |
| 748 | 11412.11 | 11982.36 | 0.146 | 0.145 | 4.759 | 11552.64 | 11526.57 | 0.122 | 0.12 | -0.226 |

| 749 | 11634.4 | 12338.34 | 0.122 | 0.125 | 5.7053 | 10904.43 | 10592.17 | 0.121 | 0.122 | -2.948 |
| --- | --- | --- | --- | --- | --- | --- | --- | --- | --- | --- |
| 750 | 14470.9 | 14193.65 | 0.132 | 0.14 | -1.953 | 11507.49 | 11740.16 | 0.123 | 0.125 | 1.9818 |
| 751 | 9867.742 | 10144.55 | 0.148 | 0.146 | 2.7287 | 13353.01 | 13158.99 | 0.128 | 0.128 | -1.474 |
| 752 | 12211.45 | 14026.72 | 0.113 | 0.112 | 12.942 | 11511.08 | 11947.31 | 0.123 | 0.128 | 3.6512 |
| 753 | 11570.61 | 12039.26 | 0.122 | 0.12 | 3.8927 | 12359.75 | 12347.32 | 0.121 | 0.121 | -0.101 |
| 754 | 12777.58 | 12803.64 | 0.14 | 0.14 | 0.2036 | 12437.01 | 12563.46 | 0.118 | 0.118 | 1.0065 |
| 755 | 13414.1 | 14236.25 | 0.127 | 0.124 | 5.775 | 10515.8 | 10795.04 | 0.132 | 0.131 | 2.5867 |
| 756 | 11588.3 | 13769.38 | 0.129 | 0.14 | 15.84 | 11897.77 | 12067.98 | 0.135 | 0.132 | 1.4104 |
| 757 | 12759 | 14963.59 | 0.138 | 0.132 | 14.733 | 10986.35 | 11012.42 | 0.125 | 0.126 | 0.2367 |
| 758 | 11848.62 | 12377.06 | 0.142 | 0.136 | 4.2695 | 11563.43 | 11830.83 | 0.12 | 0.125 | 2.2602 |
| 759 | 12591.36 | 12639.32 | 0.118 | 0.123 | 0.3794 | 13561.27 | 13750.53 | 0.121 | 0.123 | 1.3763 |
| 760 | 11083.94 | 12221 | 0.125 | 0.125 | 9.3041 | 14737.79 | 14828.59 | 0.125 | 0.122 | 0.6123 |
| 761 | 11828.95 | 13857.01 | 0.123 | 0.124 | 14.636 | 13138.72 | 13126.29 | 0.121 | 0.12 | -0.095 |
| 762 | 12376.33 | 12434.96 | 0.112 | 0.125 | 0.4715 | 13265.22 | 13263.8 | 0.12 | 0.126 | -0.011 |
| 763 | 10357.1 | 10554 | 0.145 | 0.146 | 1.8656 | 12113.51 | 12328.9 | 0.131 | 0.13 | 1.747 |
| 764 | 12612.2 | 14231.93 | 0.125 | 0.122 | 11.381 | 15692.2 | 16026.31 | 0.129 | 0.127 | 2.0847 |
| 765 | 12763.09 | 13880.63 | 0.136 | 0.135 | 8.0511 | 11067.6 | 11643.29 | 0.125 | 0.118 | 4.9444 |
| 766 | 12611.51 | 13758.04 | 0.127 | 0.135 | 8.3335 | 11608.45 | 11745.14 | 0.129 | 0.126 | 1.1638 |
| 767 | 14942.83 | 15205.55 | 0.133 | 0.129 | 1.7278 | 13778.22 | 13782.95 | 0.125 | 0.123 | 0.0343 |
| 768 | 15454.68 | 15173.71 | 0.125 | 0.135 | -1.852 | 12692.25 | 12806.34 | 0.12 | 0.124 | 0.8909 |
| 769 | 10833.97 | 11066.93 | 0.147 | 0.146 | 2.1051 | 10710.36 | 11650.3 | 0.127 | 0.135 | 8.0679 |
| 770 | 11656.31 | 11966.46 | 0.146 | 0.145 | 2.5919 | 11340.29 | 11848.9 | 0.12 | 0.125 | 4.2925 |
| 771 | 12326.74 | 12673.07 | 0.109 | 0.121 | 2.7328 | 11571.92 | 11525.94 | 0.124 | 0.117 | -0.399 |
| 772 | 12736.99 | 13103.01 | 0.144 | 0.142 | 2.7934 | 14232.48 | 14651.11 | 0.13 | 0.128 | 2.8573 |
| 773 | 14487.92 | 14612.27 | 0.129 | 0.129 | 0.851 | 10626.32 | 10696.4 | 0.124 | 0.123 | 0.6552 |
| 774 | 16316.77 | 17074.05 | 0.125 | 0.123 | 4.4353 | 11110.34 | 11225.69 | 0.125 | 0.121 | 1.0276 |
| 775 | 11100.33 | 11429.27 | 0.147 | 0.145 | 2.8781 | 13089.79 | 13959.2 | 0.139 | 0.138 | 6.2282 |
| 776 | 11599.77 | 11771.11 | 0.14 | 0.139 | 1.4556 | 15334.51 | 15849.91 | 0.129 | 0.128 | 3.2518 |
| 777 | 12076.23 | 12472.75 | 0.131 | 0.128 | 3.1791 | 11838.63 | 12352.31 | 0.117 | 0.125 | 4.1585 |
| 778 | 13349.27 | 14689.93 | 0.12 | 0.112 | 9.1264 | 14456.45 | 14499.18 | 0.127 | 0.127 | 0.2946 |
| 779 | 15269.52 | 15399.81 | 0.136 | 0.139 | 0.8461 | 13157.45 | 13138.25 | 0.129 | 0.126 | -0.146 |
| 780 | 11598.72 | 11685.7 | 0.14 | 0.142 | 0.7444 | 11350.45 | 13492.6 | 0.139 | 0.141 | 15.877 |
| 781 | 11090.72 | 11339.24 | 0.145 | 0.145 | 2.1918 | 11387.3 | 11882.48 | 0.123 | 0.126 | 4.1673 |
| 782 | 12045.64 | 12538.15 | 0.126 | 0.138 | 3.9281 | 11761.95 | 11897.9 | 0.13 | 0.128 | 1.1426 |
| 783 | 12923.85 | 12819.81 | 0.12 | 0.128 | -0.812 | 10766.22 | 11472.82 | 0.133 | 0.134 | 6.1589 |
| 784 | 11986.52 | 12825.55 | 0.123 | 0.124 | 6.5419 | 11682.01 | 11781.51 | 0.127 | 0.123 | 0.8445 |
| 785 | 13118.46 | 13231.8 | 0.13 | 0.13 | 0.8566 | 13335 | 13332.99 | 0.138 | 0.137 | -0.015 |
| 786 | 13867 | 13541.73 | 0.12 | 0.128 | -2.402 | 13261.95 | 13653.92 | 0.123 | 0.124 | 2.8708 |
| 787 | 12298.15 | 13728.31 | 0.138 | 0.13 | 10.418 | 12443.69 | 12370.35 | 0.122 | 0.12 | -0.593 |
| 788 | 14274.66 | 17067.03 | 0.139 | 0.132 | 16.361 | 13103.26 | 13307.23 | 0.125 | 0.124 | 1.5328 |
| 789 | 12148.76 | 12546.75 | 0.126 | 0.13 | 3.1721 | 13705.67 | 13832.83 | 0.122 | 0.121 | 0.9192 |
| 790 | 16084.24 | 17002.61 | 0.126 | 0.132 | 5.4014 | 14063.59 | 14103.09 | 0.138 | 0.137 | 0.2801 |
| 791 | 11542.55 | 12223.8 | 0.12 | 0.122 | 5.5731 | 12526.24 | 12794.56 | 0.122 | 0.127 | 2.0971 |
| 792 | 13068.05 | 12543.46 | 0.111 | 0.123 | -4.182 | 14446 | 14893.63 | 0.131 | 0.129 | 3.0055 |
| 793 | 12961.08 | 13205.47 | 0.143 | 0.142 | 1.8506 | 11750.64 | 11915.2 | 0.135 | 0.135 | 1.3811 |
| 794 | 11502.27 | 11758.3 | 0.112 | 0.12 | 2.1774 | 10590.39 | 10931.05 | 0.131 | 0.133 | 3.1164 |
| 795 | 12439.78 | 12602.83 | 0.14 | 0.139 | 1.2938 | 13262.17 | 13429.15 | 0.126 | 0.127 | 1.2435 |
| 796 | 11593.27 | 14019.06 | 0.129 | 0.14 | 17.304 | 11189.21 | 11979.54 | 0.138 | 0.141 | 6.5973 |
| 797 | 12589.45 | 12870.66 | 0.126 | 0.131 | 2.1849 | 12815.25 | 12951.19 | 0.125 | 0.123 | 1.0497 |
| 798 | 13386.34 | 14068.23 | 0.113 | 0.122 | 4.847 | 12420.71 | 12394.65 | 0.124 | 0.122 | -0.21 |

| 799 | 14141.74 | 14351.51 | 0.124 | 0.131 | 1.4617 | 11962.74 | 12254.4 | 0.126 | 0.127 | 2.3801 |
| --- | --- | --- | --- | --- | --- | --- | --- | --- | --- | --- |
| 800 | 13240.06 | 14001.15 | 0.115 | 0.123 | 5.4359 | 11503.16 | 12141.66 | 0.127 | 0.128 | 5.2587 |
| 801 | 12729.02 | 13005.8 | 0.137 | 0.134 | 2.1281 | 10856.06 | 10994.93 | 0.13 | 0.13 | 1.2631 |
| 802 | 13429.6 | 14099.27 | 0.117 | 0.124 | 4.7497 | 13453.34 | 13792.09 | 0.128 | 0.126 | 2.4561 |
| 803 | 12663.61 | 15095.57 | 0.141 | 0.145 | 16.11 | 11202.45 | 11253.37 | 0.126 | 0.125 | 0.4525 |
| 804 | 13863.09 | 13690.21 | 0.118 | 0.121 | -1.263 | 11620.96 | 12960.1 | 0.119 | 0.12 | 10.333 |
| 805 | 11515.68 | 11868.27 | 0.143 | 0.14 | 2.9708 | 10744.59 | 10987.76 | 0.127 | 0.127 | 2.2131 |
| 806 | 12284.23 | 12605.53 | 0.133 | 0.138 | 2.5489 | 14368.9 | 14516.68 | 0.13 | 0.132 | 1.018 |
| 807 | 13634.57 | 13165.16 | 0.12 | 0.128 | -3.566 | 15062.56 | 15281.8 | 0.124 | 0.124 | 1.4346 |
| 808 | 13385.07 | 14199.34 | 0.119 | 0.128 | 5.7345 | 12188.17 | 11916.82 | 0.123 | 0.12 | -2.277 |
| 809 | 11104.86 | 11463.6 | 0.144 | 0.145 | 3.1294 | 10058.49 | 10896.97 | 0.142 | 0.14 | 7.6946 |
| 810 | 14717.11 | 15284.8 | 0.131 | 0.125 | 3.7141 | 11697.98 | 11778.9 | 0.129 | 0.125 | 0.687 |
| 811 | 11442.11 | 11989.05 | 0.129 | 0.125 | 4.562 | 13629.68 | 13569.72 | 0.124 | 0.117 | -0.442 |
| 812 | 13055.75 | 13681.19 | 0.12 | 0.124 | 4.5715 | 13843.68 | 14047.65 | 0.124 | 0.123 | 1.452 |
| 813 | 11803.35 | 12245.94 | 0.144 | 0.145 | 3.6141 | 15018.39 | 16420.07 | 0.126 | 0.127 | 8.5364 |
| 814 | 11132.54 | 11505.63 | 0.147 | 0.146 | 3.2426 | 11178.38 | 11153.52 | 0.124 | 0.124 | -0.223 |
| 815 | 10913.2 | 11307.5 | 0.149 | 0.147 | 3.4871 | 16041.42 | 17448.25 | 0.122 | 0.124 | 8.0629 |
| 816 | 12986.69 | 13146.99 | 0.136 | 0.142 | 1.2192 | 10638.08 | 10613.23 | 0.12 | 0.12 | -0.234 |
| 817 | 15497.9 | 15857.57 | 0.131 | 0.132 | 2.2681 | 10769.37 | 10904.1 | 0.128 | 0.127 | 1.2356 |
| 818 | 12279.09 | 12547.67 | 0.129 | 0.13 | 2.1405 | 11396.99 | 11595.69 | 0.129 | 0.132 | 1.7136 |
| 819 | 15767.87 | 16121.21 | 0.128 | 0.126 | 2.1918 | 13659.94 | 13856.26 | 0.127 | 0.129 | 1.4169 |
| 820 | 10426.3 | 10846.66 | 0.15 | 0.149 | 3.8755 | 12930.8 | 13030.3 | 0.122 | 0.119 | 0.7636 |
| 821 | 13979.6 | 14144.74 | 0.135 | 0.137 | 1.1676 | 13191.36 | 13488.6 | 0.129 | 0.125 | 2.2037 |
| 822 | 15492.24 | 15831.15 | 0.129 | 0.125 | 2.1408 | 13720.25 | 13893.56 | 0.12 | 0.116 | 1.2474 |
| 823 | 14287.54 | 14291.68 | 0.134 | 0.135 | 0.029 | 12399.52 | 12512.58 | 0.119 | 0.122 | 0.9035 |
| 824 | 11101.61 | 13344.52 | 0.126 | 0.14 | 16.808 | 11807.65 | 11933.21 | 0.118 | 0.119 | 1.0522 |
| 825 | 12819.38 | 12907.78 | 0.139 | 0.14 | 0.6849 | 11049.6 | 11309.34 | 0.131 | 0.131 | 2.2967 |
| 826 | 12755.15 | 12897.2 | 0.14 | 0.14 | 1.1014 | 10991.07 | 11204.83 | 0.138 | 0.141 | 1.9078 |
| 827 | 16395.94 | 16685.23 | 0.128 | 0.126 | 1.7338 | 10429.62 | 11365.54 | 0.123 | 0.125 | 8.2348 |
| 828 | 11679.19 | 11775.14 | 0.141 | 0.14 | 0.8148 | 12195.64 | 12336.47 | 0.129 | 0.126 | 1.1416 |
| 829 | 14505.17 | 14509.31 | 0.137 | 0.137 | 0.0285 | 11717.03 | 11846.23 | 0.123 | 0.124 | 1.0907 |
| 830 | 13703.37 | 14122.41 | 0.122 | 0.128 | 2.9672 | 12090.05 | 12213.81 | 0.12 | 0.121 | 1.0133 |
| 831 | 11472.46 | 11919.69 | 0.147 | 0.147 | 3.752 | 12386.39 | 12494.67 | 0.12 | 0.121 | 0.8666 |
| 832 | 13505.06 | 13647.78 | 0.138 | 0.141 | 1.0457 | 12336.95 | 12433.23 | 0.119 | 0.121 | 0.7743 |
| 833 | 13150.5 | 13693.23 | 0.131 | 0.132 | 3.9634 | 14134.35 | 14590.26 | 0.129 | 0.127 | 3.1247 |
| 834 | 12797.58 | 13091.35 | 0.104 | 0.12 | 2.244 | 11533.86 | 12004.11 | 0.126 | 0.127 | 3.9174 |
| 835 | 12130.15 | 12117.72 | 0.141 | 0.14 | -0.103 | 13027.14 | 12929.15 | 0.125 | 0.123 | -0.758 |
| 836 | 12013.63 | 12725.88 | 0.147 | 0.148 | 5.5969 | 10854.78 | 10970.14 | 0.122 | 0.119 | 1.0515 |
| 837 | 15117.45 | 15121.59 | 0.135 | 0.135 | 0.0274 | 11048.65 | 11764.49 | 0.128 | 0.127 | 6.0848 |
| 838 | 12949.18 | 13500.82 | 0.115 | 0.116 | 4.086 | 11667.31 | 12001.78 | 0.129 | 0.126 | 2.7869 |
| 839 | 10867.53 | 12401.79 | 0.139 | 0.146 | 12.371 | 14359.68 | 14660.35 | 0.126 | 0.124 | 2.0509 |
| 840 | 11285.63 | 11817.59 | 0.145 | 0.146 | 4.5014 | 15558.5 | 15643.44 | 0.121 | 0.12 | 0.543 |
| 841 | 11420.02 | 12782.6 | 0.129 | 0.142 | 10.66 | 14368.26 | 14175.37 | 0.124 | 0.119 | -1.361 |
| 842 | 12486.15 | 13795.69 | 0.122 | 0.115 | 9.4923 | 12231.46 | 13126.92 | 0.121 | 0.116 | 6.8215 |
| 843 | 11619.26 | 11611.47 | 0.143 | 0.143 | -0.067 | 12316.12 | 12434.32 | 0.125 | 0.126 | 0.9506 |
| 844 | 11008.27 | 11245.92 | 0.119 | 0.123 | 2.1133 | 11378.27 | 11516.17 | 0.129 | 0.128 | 1.1975 |
| 845 | 11824.91 | 13814.23 | 0.112 | 0.113 | 14.401 | 11502.71 | 11601.62 | 0.121 | 0.122 | 0.8525 |
| 846 | 11779.28 | 12057.82 | 0.111 | 0.119 | 2.3101 | 15705.13 | 15844.8 | 0.131 | 0.13 | 0.8815 |
| 847 | 12830.53 | 15034.95 | 0.123 | 0.114 | 14.662 | 11473.58 | 11573.08 | 0.121 | 0.117 | 0.8597 |
| 848 | 13018.2 | 13439.29 | 0.126 | 0.124 | 3.1333 | 12075.92 | 12175.42 | 0.125 | 0.121 | 0.8172 |
| 849 | 12456.46 | 12918.87 | 0.123 | 0.12 | 3.5793 | 15046.37 | 15246.75 | 0.125 | 0.126 | 1.3143 |

| 850 | 13125.48 | 14893.97 | 0.143 | 0.147 | 11.874 | 11877.71 | 11993.19 | 0.118 | 0.119 | 0.9629 |
| --- | --- | --- | --- | --- | --- | --- | --- | --- | --- | --- |
| 851 | 12898.85 | 12446.89 | 0.111 | 0.124 | -3.631 | 11957.86 | 11710.87 | 0.122 | 0.12 | -2.109 |
| 852 | 12426.78 | 13238.87 | 0.115 | 0.125 | 6.1342 | 12378.66 | 12443.72 | 0.123 | 0.123 | 0.5228 |
| 853 | 10445.89 | 10810.69 | 0.143 | 0.142 | 3.3745 | 14660.88 | 14560.79 | 0.127 | 0.129 | -0.687 |
| 854 | 11909.04 | 13298.86 | 0.144 | 0.148 | 10.451 | 13230.68 | 13337.87 | 0.119 | 0.12 | 0.8037 |
| 855 | 12582.78 | 12637.93 | 0.14 | 0.14 | 0.4364 | 11493.48 | 11891.51 | 0.123 | 0.128 | 3.3471 |
| 856 | 13165.69 | 13144.4 | 0.119 | 0.118 | -0.162 | 11937.65 | 12152.12 | 0.123 | 0.128 | 1.7649 |
| 857 | 10482.05 | 12328.36 | 0.142 | 0.148 | 14.976 | 12054.36 | 13141.86 | 0.133 | 0.134 | 8.2751 |
| 858 | 10980.84 | 11753.54 | 0.136 | 0.133 | 6.5742 | 12222.3 | 12359.78 | 0.12 | 0.119 | 1.1124 |
| 859 | 11781.62 | 12002.38 | 0.11 | 0.121 | 1.8393 | 13318 | 14213.16 | 0.121 | 0.116 | 6.2981 |
| 860 | 12485.12 | 14413.91 | 0.143 | 0.147 | 13.381 | 12609.2 | 11994.39 | 0.124 | 0.129 | -5.126 |
| 861 | 11803.46 | 13522.66 | 0.139 | 0.146 | 12.713 | 15512.03 | 15617.51 | 0.124 | 0.122 | 0.6754 |
| 862 | 12453.33 | 15273.68 | 0.126 | 0.144 | 18.465 | 11986 | 11849.1 | 0.125 | 0.119 | -1.155 |
| 863 | 13587.98 | 13136.02 | 0.126 | 0.127 | -3.441 | 10839.59 | 11807.51 | 0.135 | 0.137 | 8.1975 |
| 864 | 13161.6 | 13558.25 | 0.106 | 0.122 | 2.9256 | 12603.81 | 12914.77 | 0.126 | 0.128 | 2.4077 |
| 865 | 14608.5 | 15359.72 | 0.135 | 0.134 | 4.8908 | 12019.76 | 12467.8 | 0.119 | 0.116 | 3.5936 |
| 866 | 12183.6 | 13361.5 | 0.139 | 0.145 | 8.8157 | 13660.28 | 13567.56 | 0.115 | 0.119 | -0.683 |
| 867 | 13091.78 | 13071.45 | 0.12 | 0.125 | -0.156 | 14047.18 | 13928.31 | 0.126 | 0.128 | -0.853 |
| 868 | 11796.19 | 11939.75 | 0.133 | 0.128 | 1.2023 | 13098.64 | 13563.54 | 0.131 | 0.126 | 3.4276 |
| 869 | 12825.66 | 13830.03 | 0.125 | 0.13 | 7.2622 | 12762.59 | 12827.65 | 0.121 | 0.121 | 0.5072 |
| 870 | 13258.75 | 13447.63 | 0.108 | 0.118 | 1.4045 | 12312.36 | 12430.55 | 0.122 | 0.123 | 0.9509 |
| 871 | 12280.04 | 13067.95 | 0.133 | 0.128 | 6.0293 | 11303.41 | 11077.88 | 0.122 | 0.121 | -2.036 |
| 872 | 12028.45 | 12277.36 | 0.117 | 0.123 | 2.0274 | 12119.87 | 11563.8 | 0.118 | 0.115 | -4.809 |
| 873 | 16127.08 | 16603.49 | 0.124 | 0.13 | 2.8693 | 10469.41 | 11170.02 | 0.14 | 0.139 | 6.2722 |
| 874 | 14911.43 | 15165.78 | 0.13 | 0.126 | 1.6771 | 13216.25 | 13190.18 | 0.119 | 0.117 | -0.198 |
| 875 | 12719.91 | 13394.34 | 0.109 | 0.121 | 5.0352 | 10712.76 | 11379.96 | 0.125 | 0.13 | 5.8629 |
| 876 | 13552.89 | 17075.02 | 0.133 | 0.131 | 20.627 | 15176.41 | 15121.59 | 0.122 | 0.117 | -0.363 |
| 877 | 15390.86 | 15639.64 | 0.134 | 0.135 | 1.5907 | 11134.38 | 11557.14 | 0.137 | 0.124 | 3.658 |
| 878 | 11820.76 | 13269.55 | 0.117 | 0.115 | 10.918 | 12004.23 | 12104.23 | 0.134 | 0.133 | 0.8262 |
| 879 | 12280.15 | 12335.68 | 0.119 | 0.127 | 0.4501 | 13036.34 | 13203.32 | 0.12 | 0.121 | 1.2647 |
| 880 | 13744.52 | 13992.06 | 0.124 | 0.131 | 1.7691 | 12857.97 | 13040.31 | 0.124 | 0.125 | 1.3983 |
| 881 | 11334.63 | 11716.79 | 0.14 | 0.141 | 3.2617 | 14633.46 | 14791.84 | 0.124 | 0.124 | 1.0707 |
| 882 | 11651.88 | 11615.1 | 0.142 | 0.142 | -0.317 | 11574.64 | 11582.21 | 0.125 | 0.126 | 0.0654 |
| 883 | 12313.23 | 12775.41 | 0.115 | 0.125 | 3.6178 | 13268.15 | 13192.79 | 0.122 | 0.121 | -0.571 |
| 884 | 14425.49 | 14508.25 | 0.13 | 0.136 | 0.5704 | 11593.36 | 11700.14 | 0.123 | 0.119 | 0.9126 |
| 885 | 12050.19 | 12452.56 | 0.126 | 0.122 | 3.2313 | 11617.11 | 11682.17 | 0.124 | 0.125 | 0.5569 |
| 886 | 12287.44 | 12167.32 | 0.118 | 0.125 | -0.987 | 13358.78 | 13423.84 | 0.117 | 0.118 | 0.4847 |
| 887 | 15004.61 | 14629.37 | 0.128 | 0.134 | -2.565 | 13901.32 | 13785.38 | 0.126 | 0.128 | -0.841 |
| 888 | 12074.51 | 12527.06 | 0.116 | 0.127 | 3.6126 | 10076.1 | 10385.13 | 0.138 | 0.14 | 2.9757 |
| 889 | 11258.48 | 13009.52 | 0.136 | 0.143 | 13.46 | 11514.94 | 11965.77 | 0.13 | 0.124 | 3.7677 |
| 890 | 12849.77 | 12955.17 | 0.121 | 0.128 | 0.8136 | 11606.71 | 11751.52 | 0.122 | 0.119 | 1.2323 |
| 891 | 13104.11 | 13970.75 | 0.12 | 0.118 | 6.2032 | 11341 | 11347.56 | 0.126 | 0.122 | 0.0579 |
| 892 | 13683.85 | 14849.74 | 0.12 | 0.114 | 7.8513 | 11650.4 | 11715.46 | 0.121 | 0.121 | 0.5553 |
| 893 | 13326.76 | 13607.71 | 0.137 | 0.136 | 2.0647 | 12345.06 | 12549.03 | 0.127 | 0.125 | 1.6254 |
| 894 | 13862.91 | 13354.24 | 0.133 | 0.136 | -3.809 | 12594.59 | 12694.08 | 0.123 | 0.119 | 0.7838 |
| 895 | 15055.58 | 14880.64 | 0.126 | 0.132 | -1.176 | 14808.97 | 14844.2 | 0.124 | 0.121 | 0.2374 |
| 896 | 11744.54 | 12123.57 | 0.136 | 0.131 | 3.1264 | 12302 | 12840.65 | 0.127 | 0.127 | 4.1949 |
| 897 | 11320.86 | 11762.74 | 0.147 | 0.149 | 3.7566 | 14731.36 | 15610.11 | 0.136 | 0.138 | 5.6294 |
| 898 | 13105.6 | 13109.74 | 0.138 | 0.139 | 0.0316 | 11844.64 | 12014.81 | 0.122 | 0.119 | 1.4164 |
| 899 | 11535.35 | 13578.1 | 0.113 | 0.116 | 15.044 | 11168.14 | 11197.13 | 0.124 | 0.124 | 0.2589 |

| 900 | 12256.18 | 13388.52 | 0.124 | 0.12 | 8.4575 | 12373.79 | 12488.73 | 0.125 | 0.125 | 0.9203 |
| --- | --- | --- | --- | --- | --- | --- | --- | --- | --- | --- |
| 901 | 13649.74 | 14510.33 | 0.117 | 0.119 | 5.9309 | 14492.68 | 15824.95 | 0.124 | 0.125 | 8.4188 |
| 902 | 13486.34 | 13733.91 | 0.132 | 0.13 | 1.8026 | 14014.74 | 15400.86 | 0.127 | 0.129 | 9.0003 |
| 903 | 13119.79 | 13015.49 | 0.101 | 0.118 | -0.801 | 11329.63 | 11632.3 | 0.13 | 0.128 | 2.602 |
| 904 | 13673.95 | 13649.44 | 0.113 | 0.118 | -0.18 | 11303.51 | 11421.71 | 0.124 | 0.125 | 1.0349 |
| 905 | 13003.54 | 13181.62 | 0.135 | 0.133 | 1.3509 | 11854.64 | 12128.81 | 0.119 | 0.125 | 2.2605 |
| 906 | 10925.71 | 11454.56 | 0.151 | 0.147 | 4.617 | 13644.17 | 13762.37 | 0.116 | 0.117 | 0.8588 |
| 907 | 11906.18 | 12252.91 | 0.144 | 0.141 | 2.8297 | 12905.46 | 12679.93 | 0.12 | 0.119 | -1.779 |
| 908 | 11430.46 | 12252.75 | 0.137 | 0.132 | 6.7111 | 15655.86 | 16843.36 | 0.122 | 0.122 | 7.0503 |
| 909 | 16897.13 | 17187.46 | 0.121 | 0.132 | 1.6892 | 13682.57 | 14107.98 | 0.123 | 0.123 | 3.0154 |
| 910 | 12219.6 | 12378.51 | 0.135 | 0.134 | 1.2837 | 11227.41 | 11301.26 | 0.129 | 0.129 | 0.6534 |
| 911 | 11508.45 | 11991.88 | 0.111 | 0.123 | 4.0313 | 14066.33 | 14484.96 | 0.131 | 0.129 | 2.8901 |
| 912 | 13989.77 | 13974.92 | 0.135 | 0.135 | -0.106 | 14053.91 | 14416.09 | 0.125 | 0.123 | 2.5123 |
| 913 | 11767.76 | 13068.29 | 0.135 | 0.133 | 9.9518 | 12196.83 | 12721.55 | 0.131 | 0.129 | 4.1246 |
| 914 | 12074.4 | 12728.54 | 0.14 | 0.136 | 5.1392 | 13373.4 | 13491.6 | 0.119 | 0.12 | 0.8761 |
| 915 | 9992.803 | 10185.56 | 0.147 | 0.147 | 1.8924 | 13353.87 | 14307.16 | 0.121 | 0.113 | 6.663 |
| 916 | 16094.34 | 16247.8 | 0.131 | 0.133 | 0.9445 | 10677.52 | 11274.54 | 0.135 | 0.138 | 5.2953 |
| 917 | 11846.44 | 11850.67 | 0.114 | 0.118 | 0.0357 | 11752.8 | 11817.86 | 0.123 | 0.123 | 0.5505 |
| 918 | 12092.17 | 13429.07 | 0.139 | 0.146 | 9.9553 | 12391.01 | 12546.24 | 0.127 | 0.124 | 1.2373 |
| 919 | 15402.69 | 15963.91 | 0.132 | 0.134 | 3.5155 | 12390.48 | 12992.52 | 0.129 | 0.122 | 4.6338 |
| 920 | 12504.82 | 13643.85 | 0.139 | 0.134 | 8.3483 | 12503.34 | 12789.44 | 0.129 | 0.126 | 2.237 |
| 921 | 11708.51 | 12234.7 | 0.136 | 0.133 | 4.3008 | 13642.67 | 13741.75 | 0.126 | 0.12 | 0.721 |
| 922 | 15347.88 | 15196.54 | 0.126 | 0.133 | -0.996 | 12743.85 | 12874.27 | 0.126 | 0.129 | 1.013 |
| 923 | 13155.6 | 13989.79 | 0.126 | 0.132 | 5.9629 | 13328.39 | 13557.77 | 0.127 | 0.128 | 1.6918 |
| 924 | 14782.19 | 15275.17 | 0.124 | 0.13 | 3.2273 | 11943.36 | 12080.85 | 0.12 | 0.119 | 1.1381 |
| 925 | 13028.63 | 14284.52 | 0.141 | 0.145 | 8.792 | 11944.04 | 11931.61 | 0.119 | 0.119 | -0.104 |
| 926 | 11862.5 | 13074.05 | 0.142 | 0.147 | 9.2668 | 12364.87 | 12808.59 | 0.133 | 0.129 | 3.4643 |
| 927 | 12348.28 | 12735.35 | 0.108 | 0.119 | 3.0393 | 11819.87 | 11995.85 | 0.13 | 0.129 | 1.467 |
| 928 | 13342.23 | 14291.19 | 0.119 | 0.123 | 6.6402 | 10351.22 | 10492.55 | 0.126 | 0.127 | 1.347 |
| 929 | 12034.19 | 13602.1 | 0.126 | 0.133 | 11.527 | 11144.42 | 11285.75 | 0.125 | 0.126 | 1.2523 |
| 930 | 13264.68 | 12879.57 | 0.136 | 0.139 | -2.99 | 13481.89 | 13895.28 | 0.121 | 0.121 | 2.9751 |
| 931 | 11206.28 | 11615.22 | 0.124 | 0.126 | 3.5208 | 14856.39 | 15948.16 | 0.135 | 0.135 | 6.8457 |
| 932 | 13958.94 | 13516.84 | 0.135 | 0.138 | -3.271 | 11943.85 | 12307.65 | 0.132 | 0.134 | 2.9559 |
| 933 | 11396.59 | 11414.37 | 0.142 | 0.144 | 0.1558 | 12312.09 | 12253.89 | 0.122 | 0.115 | -0.475 |
| 934 | 12549.92 | 13212.01 | 0.117 | 0.125 | 5.0113 | 14140.76 | 15459.68 | 0.126 | 0.128 | 8.5314 |
| 935 | 12054.33 | 12343.62 | 0.129 | 0.131 | 2.3436 | 11949.46 | 12026.74 | 0.119 | 0.118 | 0.6426 |
| 936 | 12751.6 | 14865.15 | 0.141 | 0.145 | 14.218 | 10462.52 | 10437.67 | 0.119 | 0.119 | -0.238 |
| 937 | 12118.52 | 12401.4 | 0.139 | 0.138 | 2.281 | 11477.17 | 11542.23 | 0.122 | 0.122 | 0.5637 |
| 938 | 11411.43 | 11965.52 | 0.145 | 0.143 | 4.6307 | 15184.35 | 15933.9 | 0.137 | 0.139 | 4.7041 |
| 939 | 12765.7 | 12865.7 | 0.107 | 0.115 | 0.7773 | 11579.79 | 11712.81 | 0.13 | 0.129 | 1.1356 |
| 940 | 15755.88 | 16191.15 | 0.125 | 0.128 | 2.6883 | 12644.99 | 12491.51 | 0.126 | 0.12 | -1.229 |
| 941 | 14508.24 | 14415.22 | 0.126 | 0.129 | -0.645 | 14273.01 | 14496.24 | 0.126 | 0.124 | 1.5399 |
| 942 | 11933.73 | 15457.82 | 0.14 | 0.139 | 22.798 | 15689.76 | 15939.56 | 0.132 | 0.132 | 1.5671 |
| 943 | 12770.7 | 16008.06 | 0.136 | 0.134 | 20.223 | 12453.67 | 13511.95 | 0.134 | 0.133 | 7.8322 |
| 944 | 12446.3 | 13744.41 | 0.124 | 0.117 | 9.4447 | 11548.33 | 11677.95 | 0.13 | 0.129 | 1.11 |
| 945 | 15568.5 | 15990.77 | 0.123 | 0.129 | 2.6407 | 12578.58 | 12728.7 | 0.128 | 0.129 | 1.1794 |
| 946 | 11393.07 | 11571.69 | 0.147 | 0.147 | 1.5435 | 13492.7 | 13797.38 | 0.131 | 0.129 | 2.2082 |
| 947 | 10963.02 | 12053.18 | 0.132 | 0.13 | 9.0446 | 10394.21 | 11290.99 | 0.132 | 0.133 | 7.9424 |
| 948 | 12340.05 | 13420.54 | 0.121 | 0.117 | 8.051 | 14345.32 | 15168.09 | 0.132 | 0.135 | 5.4244 |
| 949 | 11059.58 | 13231.41 | 0.139 | 0.147 | 16.414 | 11096.82 | 11124.6 | 0.124 | 0.125 | 0.2497 |
| 950 | 11559.71 | 11994.51 | 0.147 | 0.147 | 3.625 | 15620.72 | 15858.17 | 0.128 | 0.127 | 1.4973 |

| 951 | 11949.51 | 12011.65 | 0.143 | 0.144 | 0.5173 | 11504.88 | 11643.75 | 0.125 | 0.125 | 1.1927 |
| --- | --- | --- | --- | --- | --- | --- | --- | --- | --- | --- |
| 952 | 11212.23 | 13019.88 | 0.13 | 0.141 | 13.884 | 13133.25 | 13035.26 | 0.125 | 0.124 | -0.752 |
| 953 | 15452.19 | 15019.05 | 0.126 | 0.133 | -2.884 | 15619.74 | 16561.21 | 0.132 | 0.132 | 5.6848 |
| 954 | 13440.38 | 14166.83 | 0.106 | 0.115 | 5.1278 | 14692.78 | 14837.51 | 0.122 | 0.121 | 0.9754 |
| 955 | 12653.08 | 12100.2 | 0.112 | 0.124 | -4.569 | 11803.21 | 11906.14 | 0.12 | 0.116 | 0.8645 |
| 956 | 10130.98 | 10272.31 | 0.147 | 0.147 | 1.3759 | 15441.15 | 16839.36 | 0.124 | 0.124 | 8.3032 |
| 957 | 13022.13 | 14604.01 | 0.121 | 0.111 | 10.832 | 13450.42 | 13207.58 | 0.116 | 0.114 | -1.839 |
| 958 | 10933.13 | 15265.92 | 0.143 | 0.141 | 28.382 | 11321.79 | 11282.88 | 0.125 | 0.119 | -0.345 |
| 959 | 12010.07 | 12406.88 | 0.138 | 0.141 | 3.1983 | 13543.93 | 13896.91 | 0.132 | 0.128 | 2.54 |
| 960 | 13432.98 | 13663.61 | 0.113 | 0.123 | 1.6879 | 12754.82 | 12944.94 | 0.127 | 0.126 | 1.4687 |
| 961 | 12107.15 | 12338.07 | 0.11 | 0.122 | 1.8716 | 12660.4 | 13097.23 | 0.124 | 0.123 | 3.3353 |
| 962 | 11947.38 | 12403.9 | 0.146 | 0.148 | 3.6804 | 13853.48 | 13755.49 | 0.121 | 0.119 | -0.712 |
| 963 | 12807.82 | 12806.61 | 0.141 | 0.141 | -0.009 | 12034.7 | 12179.51 | 0.125 | 0.121 | 1.189 |
| 964 | 12185.55 | 12164.34 | 0.144 | 0.144 | -0.174 | 11179.36 | 11610.22 | 0.133 | 0.133 | 3.7111 |
| 965 | 11023.11 | 11487.11 | 0.146 | 0.143 | 4.0394 | 15624.05 | 16274.56 | 0.129 | 0.13 | 3.9971 |
| 966 | 14304.72 | 14563.21 | 0.13 | 0.126 | 1.775 | 12481.59 | 12789.63 | 0.122 | 0.124 | 2.4085 |
| 967 | 12763.37 | 12719.11 | 0.104 | 0.114 | -0.348 | 11981.26 | 11997.53 | 0.127 | 0.127 | 0.1356 |
| 968 | 12900.17 | 12996.74 | 0.138 | 0.139 | 0.743 | 11650.8 | 13018.02 | 0.14 | 0.14 | 10.502 |
| 969 | 10767.99 | 11196.93 | 0.151 | 0.147 | 3.8309 | 11845.28 | 11832.85 | 0.122 | 0.122 | -0.105 |
| 970 | 11121.57 | 11382.23 | 0.13 | 0.132 | 2.2901 | 13548.48 | 14393.59 | 0.128 | 0.13 | 5.8714 |
| 971 | 11986.61 | 12162.09 | 0.136 | 0.133 | 1.4428 | 12172.45 | 12253.37 | 0.138 | 0.137 | 0.6604 |
| 972 | 11963.16 | 12082.25 | 0.141 | 0.14 | 0.9856 | 13099.88 | 13268.75 | 0.125 | 0.124 | 1.2727 |
| 973 | 12079.08 | 12076.38 | 0.12 | 0.122 | -0.022 | 13316.79 | 13353.65 | 0.12 | 0.125 | 0.2761 |
| 974 | 11766.88 | 12378.04 | 0.135 | 0.133 | 4.9375 | 10846.33 | 12520.87 | 0.139 | 0.14 | 13.374 |
| 975 | 12064.33 | 12570.88 | 0.126 | 0.123 | 4.0296 | 11248.09 | 12674.52 | 0.131 | 0.129 | 11.254 |
| 976 | 13657.27 | 15502.79 | 0.134 | 0.136 | 11.904 | 11680.46 | 12128.69 | 0.127 | 0.128 | 3.6956 |
| 977 | 12190.52 | 12659.17 | 0.12 | 0.118 | 3.7021 | 12816.52 | 12912.79 | 0.121 | 0.123 | 0.7456 |
| 978 | 12105.75 | 15170.33 | 0.125 | 0.141 | 20.201 | 13525.02 | 13985.07 | 0.131 | 0.129 | 3.2896 |
| 979 | 12383.94 | 12745.03 | 0.135 | 0.136 | 2.8332 | 12855.05 | 13291.07 | 0.129 | 0.126 | 3.2805 |
| 980 | 12114.52 | 13302.13 | 0.125 | 0.118 | 8.928 | 11896.76 | 11995.46 | 0.13 | 0.132 | 0.8228 |
| 981 | 12510.43 | 13459.84 | 0.113 | 0.123 | 7.0537 | 10666.07 | 10731.13 | 0.129 | 0.129 | 0.6063 |
| 982 | 11440.79 | 15129.47 | 0.105 | 0.113 | 24.381 | 11242.35 | 11217.49 | 0.119 | 0.119 | -0.222 |
| 983 | 13937.28 | 17544.52 | 0.136 | 0.132 | 20.561 | 15204.81 | 15660.79 | 0.124 | 0.123 | 2.9116 |
| 984 | 12510.48 | 12791.44 | 0.144 | 0.143 | 2.1964 | 14622.02 | 14664.74 | 0.126 | 0.126 | 0.2913 |
| 985 | 13151.56 | 13100.81 | 0.123 | 0.131 | -0.387 | 10179.03 | 10611.61 | 0.138 | 0.139 | 4.0765 |
| 986 | 14344.75 | 14363.33 | 0.13 | 0.135 | 0.1293 | 10978.62 | 11153.98 | 0.124 | 0.123 | 1.5721 |
| 987 | 12437.96 | 12252.86 | 0.119 | 0.123 | -1.511 | 11153.13 | 11228.48 | 0.126 | 0.127 | 0.6711 |
| 988 | 15547.88 | 16105 | 0.125 | 0.131 | 3.4593 | 12993.16 | 13406.98 | 0.118 | 0.119 | 3.0865 |
| 989 | 12827.17 | 13121.56 | 0.14 | 0.14 | 2.2435 | 14553.59 | 14596.31 | 0.127 | 0.127 | 0.2927 |
| 990 | 12018.48 | 14079.32 | 0.117 | 0.117 | 14.637 | 15029.35 | 16239.31 | 0.137 | 0.135 | 7.4508 |
| 991 | 12911.74 | 13754.2 | 0.12 | 0.119 | 6.1251 | 15278.06 | 15406 | 0.126 | 0.124 | 0.8305 |
| 992 | 15572.3 | 16821.89 | 0.127 | 0.129 | 7.4283 | 12537.46 | 12638.26 | 0.123 | 0.121 | 0.7976 |
| 993 | 12650.68 | 12797.04 | 0.137 | 0.139 | 1.1437 | 15282.37 | 15492.55 | 0.132 | 0.13 | 1.3566 |
| 994 | 13217.43 | 13762.28 | 0.13 | 0.132 | 3.959 | 11080.14 | 11170.14 | 0.134 | 0.133 | 0.8057 |
| 995 | 12221.13 | 12355.48 | 0.141 | 0.142 | 1.0874 | 12610.49 | 12839.87 | 0.126 | 0.128 | 1.7864 |
| 996 | 11210.34 | 12244.55 | 0.133 | 0.127 | 8.4463 | 12299.4 | 12619.44 | 0.12 | 0.123 | 2.5361 |
| 997 | 14895.05 | 15085.05 | 0.133 | 0.13 | 1.2595 | 11135.04 | 11597.33 | 0.132 | 0.131 | 3.9862 |
| 998 | 12035.2 | 13104.52 | 0.147 | 0.146 | 8.16 | 14893.72 | 16247.83 | 0.131 | 0.131 | 8.3341 |
| 999 | 12371.93 | 12505.77 | 0.139 | 0.14 | 1.0703 | 10738.88 | 11148.83 | 0.126 | 0.125 | 3.6771 |
| 1000 | 13187.54 | 12744.85 | 0.136 | 0.139 | -3.473 | 12386.15 | 12606.99 | 0.121 | 0.125 | 1.7517 |

|  | Rama sample unite (1) E-W | | | | | Yeha sample unite (2) E-W | | | | |
| --- | --- | --- | --- | --- | --- | --- | --- | --- | --- | --- |
|  | UM L | M L | UM C | M C | L Ch % | UM L | M L | UM C | M C | L Ch % |
| 1 | 15085.54 | 16622.45 | 0.117 | 0.123 | 9.246 | 16035.97 | 15635.67 | 0.0938 | 0.0987 | -2.56 |
| 2 | 14590.11 | 14667.77 | 0.116 | 0.103 | 0.5295 | 18190.44 | 18212.15 | 0.1017 | 0.1044 | 0.1192 |
| 3 | 17899.53 | 16012.93 | 0.1281 | 0.107 | -11.782 | 15058.89 | 14812.23 | 0.0958 | 0.0955 | -1.665 |
| 4 | 12320.97 | 12258.54 | 0.1099 | 0.093 | -0.5092 | 16805.61 | 16810.26 | 0.0961 | 0.1016 | 0.0276 |
| 5 | 12223.86 | 12378.84 | 0.1197 | 0.119 | 1.2519 | 12079.05 | 12333.31 | 0.0997 | 0.0987 | 2.0616 |
| 6 | 14504.19 | 15180.31 | 0.1087 | 0.109 | 4.4539 | 12802.31 | 12903.02 | 0.1109 | 0.1135 | 0.7805 |
| 7 | 13563.92 | 14776.09 | 0.1169 | 0.118 | 8.2036 | 13256.86 | 14968.03 | 0.1074 | 0.0993 | 11.432 |
| 8 | 12778.53 | 12846.31 | 0.119 | 0.119 | 0.5276 | 12942.86 | 15678.91 | 0.0992 | 0.0968 | 17.451 |
| 9 | 13164.72 | 13207.44 | 0.1142 | 0.118 | 0.3235 | 14087.92 | 13975.49 | 0.1029 | 0.1023 | -0.804 |
| 10 | 12188.36 | 12326.77 | 0.1243 | 0.124 | 1.1228 | 15018.92 | 14999.55 | 0.0978 | 0.0962 | -0.129 |
| 11 | 12213.96 | 12808.73 | 0.1149 | 0.109 | 4.6434 | 14741.95 | 14709.81 | 0.0994 | 0.0994 | -0.218 |
| 12 | 11771.59 | 11916.56 | 0.0989 | 0.103 | 1.2166 | 15010.44 | 15041.44 | 0.0985 | 0.0978 | 0.2061 |
| 13 | 12390.85 | 12645.11 | 0.1216 | 0.122 | 2.0108 | 14521.47 | 14518.33 | 0.1006 | 0.0984 | -0.022 |
| 14 | 15463.46 | 17085.02 | 0.1148 | 0.121 | 9.4911 | 15902.92 | 15956.64 | 0.0994 | 0.103 | 0.3367 |
| 15 | 17015.35 | 15387.28 | 0.1327 | 0.108 | -10.581 | 15099.83 | 15087.4 | 0.0925 | 0.0919 | -0.082 |
| 16 | 11790.37 | 11928.78 | 0.1226 | 0.122 | 1.1603 | 12916.63 | 13141.28 | 0.1084 | 0.11 | 1.7095 |
| 17 | 12562.4 | 12690.77 | 0.1244 | 0.126 | 1.0115 | 16246.47 | 16553.21 | 0.1022 | 0.0993 | 1.8531 |
| 18 | 11957.01 | 12330.03 | 0.126 | 0.13 | 3.0253 | 16151.43 | 16301.72 | 0.0913 | 0.0872 | 0.922 |
| 19 | 10388.05 | 11167.95 | 0.1292 | 0.126 | 6.9834 | 13311.13 | 13586.1 | 0.1003 | 0.0981 | 2.0239 |
| 20 | 14373.14 | 14824.09 | 0.1206 | 0.124 | 3.042 | 13179.66 | 13328.95 | 0.1084 | 0.1076 | 1.12 |
| 21 | 14606.58 | 16182.01 | 0.1203 | 0.107 | 9.7357 | 15138.71 | 15767.5 | 0.0935 | 0.095 | 3.9879 |
| 22 | 11340.69 | 11325.34 | 0.1277 | 0.128 | -0.1356 | 15628.71 | 15863.36 | 0.1008 | 0.1054 | 1.4792 |
| 23 | 12069.28 | 12328.69 | 0.1188 | 0.12 | 2.1041 | 13651.19 | 14765.33 | 0.0975 | 0.099 | 7.5457 |
| 24 | 12418.48 | 13031.03 | 0.1133 | 0.108 | 4.7007 | 16797.13 | 15444.75 | 0.1028 | 0.0809 | -8.756 |
| 25 | 12181.41 | 12470.53 | 0.1256 | 0.126 | 2.3184 | 13558.15 | 13627.44 | 0.1096 | 0.1089 | 0.5085 |
| 26 | 16300.34 | 17866.33 | 0.1117 | 0.118 | 8.7651 | 15228.2 | 15890.83 | 0.0955 | 0.0968 | 4.1699 |
| 27 | 14984.05 | 15089.49 | 0.1247 | 0.123 | 0.6988 | 14085.72 | 14352.59 | 0.1032 | 0.1038 | 1.8593 |
| 28 | 12835.32 | 12973.73 | 0.1188 | 0.118 | 1.0668 | 13302.49 | 13641.99 | 0.0955 | 0.1055 | 2.4886 |
| 29 | 15150.82 | 15098.31 | 0.1183 | 0.102 | -0.3478 | 12910.98 | 12918.26 | 0.1067 | 0.1044 | 0.0563 |
| 30 | 10982.47 | 13245.71 | 0.1225 | 0.113 | 17.087 | 13599.36 | 13673.47 | 0.1058 | 0.1071 | 0.542 |
| 31 | 12616.24 | 12766.95 | 0.1206 | 0.102 | 1.1805 | 14621.05 | 14664.87 | 0.1 | 0.1022 | 0.2988 |
| 32 | 15735.36 | 15973.53 | 0.1185 | 0.116 | 1.491 | 15090.82 | 15412.92 | 0.1077 | 0.1071 | 2.0898 |
| 33 | 13294.28 | 13579.65 | 0.107 | 0.099 | 2.1015 | 13353.57 | 13450.72 | 0.1026 | 0.1044 | 0.7223 |
| 34 | 12421.66 | 12504.5 | 0.1145 | 0.113 | 0.6625 | 16087.21 | 16294.69 | 0.1016 | 0.1011 | 1.2733 |
| 35 | 13496.34 | 13901.31 | 0.1276 | 0.132 | 2.9132 | 16194.52 | 14765.58 | 0.1063 | 0.0837 | -9.678 |
| 36 | 11692.83 | 12255.59 | 0.1321 | 0.126 | 4.5918 | 16377.44 | 17222.21 | 0.0958 | 0.0969 | 4.9051 |
| 37 | 14611.62 | 14679.4 | 0.1146 | 0.115 | 0.4617 | 13588.71 | 14008.09 | 0.1071 | 0.1031 | 2.9938 |
| 38 | 13568.48 | 13621.41 | 0.1164 | 0.118 | 0.3886 | 13755.63 | 13930.18 | 0.1033 | 0.0992 | 1.2531 |
| 39 | 14301.92 | 13849.37 | 0.1165 | 0.114 | -3.2676 | 13740.87 | 13827.44 | 0.1121 | 0.1119 | 0.6261 |
| 40 | 13596.49 | 13664.28 | 0.1147 | 0.115 | 0.4961 | 14879.07 | 16620.4 | 0.0984 | 0.0957 | 10.477 |
| 41 | 12500.85 | 13285.24 | 0.1229 | 0.124 | 5.9042 | 14315.22 | 14612 | 0.1154 | 0.1031 | 2.031 |
| 42 | 13422.65 | 14062.82 | 0.1187 | 0.12 | 4.5522 | 13233.03 | 13244.53 | 0.0987 | 0.0983 | 0.0869 |
| 43 | 17585.5 | 16370.89 | 0.1333 | 0.108 | -7.4193 | 13414.56 | 14012.46 | 0.1037 | 0.0992 | 4.2669 |
| 44 | 12957.69 | 13096.09 | 0.1173 | 0.117 | 1.0569 | 13404.3 | 13958.53 | 0.1148 | 0.1107 | 3.9705 |
| 45 | 12999.01 | 13853.69 | 0.1194 | 0.121 | 6.1693 | 15092.02 | 15814.66 | 0.0918 | 0.0933 | 4.5694 |
| 46 | 13700.3 | 14463.89 | 0.1195 | 0.121 | 5.2793 | 14964.01 | 14959.87 | 0.0959 | 0.0947 | -0.028 |
| 47 | 13831.44 | 13837.68 | 0.1236 | 0.116 | 0.0451 | 14945.99 | 15626.2 | 0.0983 | 0.0981 | 4.353 |
| 48 | 11412.97 | 11397.62 | 0.1223 | 0.123 | -0.1347 | 12961.25 | 13124.47 | 0.1015 | 0.1034 | 1.2437 |
| 49 | 12707.64 | 12747.1 | 0.1112 | 0.099 | 0.3096 | 16822.3 | 15877.99 | 0.1038 | 0.0971 | -5.947 |
| 50 | 12349.64 | 12494.61 | 0.095 | 0.099 | 1.1603 | 14473.86 | 15158.5 | 0.1017 | 0.0994 | 4.5166 |
| 51 | 13124.78 | 14242.09 | 0.1192 | 0.12 | 7.8452 | 13721.29 | 13808.95 | 0.0968 | 0.0989 | 0.6348 |
| 52 | 12013.45 | 12561.56 | 0.1196 | 0.118 | 4.3634 | 16522.33 | 17518.52 | 0.0983 | 0.1015 | 5.6865 |
| 53 | 11718.06 | 13609.79 | 0.121 | 0.112 | 13.9 | 16784.19 | 17240.43 | 0.0949 | 0.0922 | 2.6463 |
| 54 | 12278.31 | 12548.73 | 0.1293 | 0.132 | 2.1549 | 14188.44 | 14868.65 | 0.0997 | 0.0995 | 4.5748 |
| 55 | 11970.79 | 11993.21 | 0.1236 | 0.124 | 0.187 | 14849.4 | 13736.94 | 0.1068 | 0.084 | -8.098 |
| 56 | 11326.61 | 11863.51 | 0.1275 | 0.125 | 4.5256 | 14618.97 | 14544.53 | 0.0978 | 0.098 | -0.512 |
| 57 | 14161.62 | 12407.28 | 0.1205 | 0.105 | -14.14 | 16552.77 | 16340.05 | 0.0994 | 0.1043 | -1.302 |
| 58 | 12306.01 | 12599.65 | 0.1177 | 0.103 | 2.3305 | 13413.38 | 13417.82 | 0.1039 | 0.1052 | 0.0331 |
| 59 | 12398.79 | 12471.06 | 0.1237 | 0.123 | 0.5795 | 13036.48 | 13316.48 | 0.1085 | 0.1103 | 2.1027 |
| 60 | 13903.07 | 14161.24 | 0.1254 | 0.123 | 1.823 | 15098.92 | 15724.78 | 0.0974 | 0.0986 | 3.9801 |
| 61 | 12459.05 | 12787.16 | 0.1302 | 0.13 | 2.5659 | 14606.61 | 15400.25 | 0.0932 | 0.0946 | 5.1534 |
| 62 | 11785.52 | 12345.14 | 0.1318 | 0.127 | 4.5331 | 15865.73 | 14483.86 | 0.1065 | 0.0842 | -9.541 |
| 63 | 17522.17 | 15782.75 | 0.1323 | 0.109 | -11.021 | 13297.11 | 13502.26 | 0.1091 | 0.1107 | 1.5194 |
| 64 | 11447.59 | 11938.39 | 0.1195 | 0.117 | 4.1111 | 16834.25 | 17302.45 | 0.1039 | 0.1007 | 2.706 |
| 65 | 13891.97 | 13476.29 | 0.1236 | 0.105 | -3.0846 | 13646.81 | 13923.17 | 0.1086 | 0.11 | 1.9849 |
| 66 | 12963.94 | 13916.52 | 0.1172 | 0.12 | 6.845 | 13436.73 | 13324.3 | 0.1036 | 0.1029 | -0.844 |
| 67 | 12352.23 | 12357.59 | 0.1272 | 0.128 | 0.0433 | 15100.58 | 15729.36 | 0.0971 | 0.0984 | 3.9975 |
| 68 | 13744.45 | 14629.63 | 0.1201 | 0.109 | 6.0506 | 13828.33 | 13859.34 | 0.1006 | 0.0998 | 0.2237 |
| 69 | 12735.39 | 12731.25 | 0.1118 | 0.11 | -0.0325 | 12034.19 | 12288.46 | 0.0999 | 0.0988 | 2.0691 |
| 70 | 12040.82 | 12665.09 | 0.1146 | 0.109 | 4.929 | 13829.35 | 13752.28 | 0.0946 | 0.0942 | -0.56 |
| 71 | 13079.84 | 13185.28 | 0.1292 | 0.127 | 0.7997 | 15576.32 | 15590.84 | 0.1028 | 0.0986 | 0.0931 |
| 72 | 16359.21 | 18042.9 | 0.1146 | 0.119 | 9.3316 | 15755.13 | 16090.4 | 0.1061 | 0.0827 | 2.0837 |
| 73 | 13139.36 | 13469.36 | 0.114 | 0.119 | 2.45 | 13650.96 | 13386.02 | 0.1119 | 0.1033 | -1.979 |
| 74 | 11750.7 | 11849.19 | 0.1275 | 0.127 | 0.8312 | 13776.1 | 13945.1 | 0.0931 | 0.1007 | 1.2119 |
| 75 | 14039.16 | 14123.6 | 0.1185 | 0.118 | 0.5978 | 14793 | 14915.93 | 0.1002 | 0.0986 | 0.8241 |
| 76 | 13691.98 | 13808.25 | 0.1216 | 0.124 | 0.8421 | 14101.15 | 14767.22 | 0.0979 | 0.0991 | 4.5104 |
| 77 | 12557.05 | 12529.11 | 0.127 | 0.124 | -0.223 | 15131.05 | 15835.4 | 0.0966 | 0.0964 | 4.4479 |
| 78 | 12427.58 | 12510.42 | 0.1151 | 0.114 | 0.6622 | 12709.08 | 12637.87 | 0.1023 | 0.104 | -0.563 |
| 79 | 12721.63 | 13172.79 | 0.1271 | 0.122 | 3.425 | 17173.65 | 17375.66 | 0.1041 | 0.1009 | 1.1626 |
| 80 | 13948.95 | 14410.37 | 0.112 | 0.116 | 3.202 | 13031.42 | 13733.76 | 0.1185 | 0.1092 | 5.114 |
| 81 | 12794.01 | 13648.69 | 0.1194 | 0.121 | 6.262 | 11964.91 | 13260.21 | 0.0975 | 0.0955 | 9.7684 |
| 82 | 11719.51 | 11741.94 | 0.1235 | 0.124 | 0.191 | 15041.24 | 15092.54 | 0.1002 | 0.1005 | 0.3399 |
| 83 | 12184.7 | 12650.17 | 0.1134 | 0.11 | 3.6796 | 13236.4 | 13709.78 | 0.0963 | 0.1027 | 3.4529 |
| 84 | 14349.59 | 14556.28 | 0.1185 | 0.121 | 1.4199 | 12718.43 | 12705.3 | 0.1049 | 0.107 | -0.103 |
| 85 | 12875.1 | 13311.37 | 0.1054 | 0.102 | 3.2775 | 15384.42 | 16013.21 | 0.0927 | 0.0941 | 3.9267 |
| 86 | 12577.81 | 12431.45 | 0.1283 | 0.126 | -1.1773 | 15237.44 | 15406.23 | 0.1054 | 0.1057 | 1.0956 |
| 87 | 13329.93 | 13705.83 | 0.1143 | 0.111 | 2.7427 | 15719.09 | 15895.74 | 0.0987 | 0.0953 | 1.1113 |
| 88 | 11228.98 | 11383.95 | 0.1238 | 0.123 | 1.3613 | 13653.49 | 13739.14 | 0.1051 | 0.1072 | 0.6234 |
| 89 | 13708.38 | 13748.38 | 0.1094 | 0.109 | 0.2909 | 15097.28 | 16438.02 | 0.0953 | 0.0889 | 8.1564 |
| 90 | 13827.95 | 13917.74 | 0.1121 | 0.112 | 0.6452 | 13970.84 | 14058.71 | 0.1085 | 0.1077 | 0.625 |
| 91 | 11202.32 | 11761.94 | 0.1288 | 0.124 | 4.7579 | 15022.35 | 15224.69 | 0.1048 | 0.1043 | 1.329 |
| 92 | 11513.39 | 11767.65 | 0.1207 | 0.121 | 2.1607 | 13960.64 | 14374.45 | 0.1085 | 0.1064 | 2.8788 |
| 93 | 17458.07 | 17758.28 | 0.1202 | 0.119 | 1.6905 | 14867.03 | 15010.97 | 0.1042 | 0.1035 | 0.9589 |
| 94 | 13460.32 | 13482.75 | 0.1247 | 0.125 | 0.1663 | 14260.61 | 14453.92 | 0.1093 | 0.1051 | 1.3374 |
| 95 | 13966.68 | 14072.12 | 0.1284 | 0.126 | 0.7493 | 12281.89 | 12263.4 | 0.1067 | 0.1085 | -0.151 |
| 96 | 12134.17 | 11997.06 | 0.1222 | 0.101 | -1.1428 | 13817.55 | 13688.05 | 0.1054 | 0.1059 | -0.946 |
| 97 | 15260.44 | 15320.44 | 0.1137 | 0.113 | 0.3916 | 15576.44 | 15780.76 | 0.1071 | 0.1056 | 1.2947 |
| 98 | 12187.78 | 12333.05 | 0.114 | 0.112 | 1.1779 | 13593.68 | 13629.03 | 0.1068 | 0.1046 | 0.2594 |
| 99 | 15947.5 | 16396.82 | 0.1295 | 0.115 | 2.7403 | 17700.3 | 17855.95 | 0.1069 | 0.1052 | 0.8717 |
| 100 | 15831.01 | 15826.96 | 0.1166 | 0.12 | -0.0256 | 16760.89 | 17282.99 | 0.1151 | 0.1116 | 3.0209 |
| 101 | 13758.06 | 13825.84 | 0.1149 | 0.115 | 0.4903 | 14543.68 | 15181.46 | 0.0937 | 0.0954 | 4.2011 |
| 102 | 16709.16 | 17063.72 | 0.12 | 0.119 | 2.0778 | 12652.93 | 12886.07 | 0.1172 | 0.1153 | 1.8092 |
| 103 | 13826.16 | 14795.82 | 0.1126 | 0.115 | 6.5536 | 15374.93 | 15864.3 | 0.0987 | 0.0952 | 3.0848 |
| 104 | 12079 | 12762.1 | 0.1315 | 0.134 | 5.3526 | 13474.98 | 13759.95 | 0.1104 | 0.108 | 2.071 |
| 105 | 11509.34 | 11779.76 | 0.1269 | 0.13 | 2.2956 | 15491.64 | 15686.62 | 0.1053 | 0.0973 | 1.2429 |
| 106 | 12334.93 | 12500.24 | 0.1194 | 0.11 | 1.3224 | 16002.92 | 16076.26 | 0.107 | 0.1106 | 0.4562 |
| 107 | 11480.7 | 11521 | 0.1195 | 0.101 | 0.3497 | 13596.28 | 13871.25 | 0.0996 | 0.0974 | 1.9823 |
| 108 | 13320.77 | 14865.49 | 0.1267 | 0.112 | 10.391 | 13511.63 | 14271.55 | 0.0997 | 0.0972 | 5.3247 |
| 109 | 15986.66 | 17341.95 | 0.1148 | 0.12 | 7.8151 | 14354.6 | 14983.38 | 0.0969 | 0.0983 | 4.1966 |
| 110 | 12384.12 | 13390.8 | 0.1242 | 0.123 | 7.5177 | 14615.64 | 14395.6 | 0.0996 | 0.0978 | -1.528 |
| 111 | 17840.79 | 17166.19 | 0.1333 | 0.113 | -3.9299 | 14731.12 | 14881.42 | 0.0948 | 0.0904 | 1.0099 |
| 112 | 11845.21 | 12221.12 | 0.1213 | 0.117 | 3.0759 | 15037.24 | 15163.39 | 0.0974 | 0.0999 | 0.832 |
| 113 | 13311.79 | 13994.89 | 0.131 | 0.133 | 4.8811 | 13958.58 | 16099.15 | 0.0943 | 0.0945 | 13.296 |
| 114 | 15573.79 | 17133.93 | 0.1126 | 0.119 | 9.1055 | 14656.91 | 14910.43 | 0.1038 | 0.0988 | 1.7003 |
| 115 | 13578.97 | 14531.56 | 0.1115 | 0.114 | 6.5553 | 17023.96 | 17008.81 | 0.1027 | 0.1044 | -0.089 |
| 116 | 14012.33 | 13796.05 | 0.1212 | 0.109 | -1.5677 | 17074.4 | 17002.89 | 0.0966 | 0.1 | -0.421 |
| 117 | 16506.38 | 16285.05 | 0.1256 | 0.11 | -1.3591 | 13903.7 | 13888.14 | 0.0987 | 0.1002 | -0.112 |
| 118 | 13417.73 | 13537.82 | 0.1221 | 0.123 | 0.887 | 14511.4 | 14705.04 | 0.1017 | 0.101 | 1.3168 |
| 119 | 12898.75 | 13153.02 | 0.1235 | 0.124 | 1.9331 | 15987.79 | 16644.61 | 0.1032 | 0.1004 | 3.9461 |
| 120 | 12185.95 | 12128.79 | 0.1121 | 0.11 | -0.4713 | 14940.49 | 13546.9 | 0.1057 | 0.0823 | -10.29 |
| 121 | 17436.57 | 17691.21 | 0.1191 | 0.118 | 1.4394 | 14680.15 | 15342.78 | 0.0956 | 0.097 | 4.3189 |
| 122 | 13335.99 | 14251.94 | 0.1252 | 0.129 | 6.4268 | 16854.75 | 16858.19 | 0.0961 | 0.1018 | 0.0204 |
| 123 | 17998.1 | 17901.01 | 0.1364 | 0.112 | -0.5423 | 14768.8 | 14531.02 | 0.103 | 0.1008 | -1.636 |
| 124 | 14384.57 | 15423.31 | 0.1178 | 0.103 | 6.7348 | 14371.13 | 14159.92 | 0.0967 | 0.094 | -1.492 |
| 125 | 11235.96 | 11374.37 | 0.1249 | 0.124 | 1.2168 | 16358.02 | 16560.03 | 0.1063 | 0.1029 | 1.2199 |
| 126 | 15968.73 | 17329.29 | 0.1126 | 0.121 | 7.8512 | 13828.23 | 14502.07 | 0.097 | 0.0979 | 4.6466 |
| 127 | 18243.69 | 19052.75 | 0.1324 | 0.127 | 4.2465 | 16431.04 | 16398.91 | 0.0955 | 0.0955 | -0.196 |
| 128 | 12433.36 | 13093.69 | 0.1253 | 0.128 | 5.0431 | 13082.49 | 12890.06 | 0.1061 | 0.1063 | -1.493 |
| 129 | 11583.35 | 12636.72 | 0.1212 | 0.124 | 8.3358 | 15973.89 | 16908.9 | 0.0954 | 0.1046 | 5.5297 |
| 130 | 13674.36 | 13742.14 | 0.1099 | 0.11 | 0.4932 | 18220.56 | 18114.7 | 0.0951 | 0.0992 | -0.584 |
| 131 | 12692.02 | 12951.23 | 0.1249 | 0.124 | 2.0014 | 13702.84 | 13586.28 | 0.104 | 0.1043 | -0.858 |
| 132 | 14035.76 | 14154.55 | 0.1151 | 0.117 | 0.8392 | 13832.83 | 13800.7 | 0.1023 | 0.1023 | -0.233 |
| 133 | 13017.28 | 13580.07 | 0.1098 | 0.103 | 4.1443 | 13381.79 | 13678.03 | 0.1089 | 0.1053 | 2.1658 |
| 134 | 13580.34 | 13648.12 | 0.1161 | 0.117 | 0.4966 | 12064.11 | 12070.47 | 0.1057 | 0.1079 | 0.0527 |
| 135 | 17646.51 | 18246.92 | 0.1229 | 0.122 | 3.2905 | 15350.4 | 15823.21 | 0.1022 | 0.0999 | 2.9881 |
| 136 | 11539.51 | 11828.63 | 0.1242 | 0.124 | 2.4442 | 13934.35 | 13880.92 | 0.1048 | 0.1042 | -0.385 |
| 137 | 12879.24 | 12930.12 | 0.1204 | 0.121 | 0.3935 | 14413.36 | 15311.56 | 0.0924 | 0.0937 | 5.8661 |
| 138 | 11779.32 | 12078.69 | 0.1273 | 0.122 | 2.4785 | 13318.95 | 14145.6 | 0.1056 | 0.1082 | 5.8439 |
| 139 | 13353.38 | 14314.01 | 0.12 | 0.109 | 6.7111 | 15751.59 | 14475.23 | 0.1059 | 0.092 | -8.818 |
| 140 | 11654.58 | 11799.55 | 0.0988 | 0.103 | 1.2286 | 13597.66 | 16254.51 | 0.0981 | 0.0965 | 16.345 |
| 141 | 13693.19 | 13780.55 | 0.1161 | 0.115 | 0.634 | 13978.11 | 13841.37 | 0.1057 | 0.099 | -0.988 |
| 142 | 12966.92 | 13120.97 | 0.1256 | 0.128 | 1.1741 | 12845.15 | 13480.92 | 0.1047 | 0.0998 | 4.7161 |
| 143 | 13268.46 | 13406.86 | 0.1257 | 0.125 | 1.0324 | 14186.64 | 14182.71 | 0.1039 | 0.1019 | -0.028 |
| 144 | 13419.52 | 13708.64 | 0.115 | 0.115 | 2.109 | 17163.15 | 16767.39 | 0.1019 | 0.0903 | -2.36 |
| 145 | 11726.17 | 12133.99 | 0.1274 | 0.128 | 3.361 | 13433.59 | 13638.74 | 0.11 | 0.1116 | 1.5042 |
| 146 | 14189.81 | 14274.25 | 0.1173 | 0.117 | 0.5915 | 16061.2 | 15921.2 | 0.1024 | 0.1057 | -0.879 |
| 147 | 13280.8 | 13682.81 | 0.1155 | 0.116 | 2.9381 | 13723.35 | 13732.43 | 0.1036 | 0.1059 | 0.0661 |
| 148 | 12774.2 | 13710.01 | 0.1157 | 0.121 | 6.8257 | 17512.66 | 17222.94 | 0.1021 | 0.0868 | -1.682 |
| 149 | 11234.78 | 11643.07 | 0.1246 | 0.119 | 3.5067 | 12619.51 | 12857.42 | 0.097 | 0.0959 | 1.8503 |
| 150 | 11821.85 | 11960.26 | 0.1253 | 0.124 | 1.1572 | 14669.4 | 14795.55 | 0.0976 | 0.1002 | 0.8526 |
| 151 | 12327.21 | 12768.96 | 0.124 | 0.126 | 3.4596 | 14215.08 | 14422.01 | 0.0941 | 0.0932 | 1.4348 |
| 152 | 12317.81 | 12375.8 | 0.0981 | 0.101 | 0.4686 | 17027.04 | 17280.47 | 0.1086 | 0.1063 | 1.4666 |
| 153 | 13730.78 | 14030.71 | 0.1097 | 0.097 | 2.1377 | 17682.24 | 18036.59 | 0.099 | 0.1016 | 1.9646 |
| 154 | 12619.77 | 13115.45 | 0.1236 | 0.109 | 3.7794 | 15831.74 | 15885.08 | 0.1145 | 0.1057 | 0.3358 |
| 155 | 12102.69 | 12646.48 | 0.1285 | 0.123 | 4.3 | 11081.38 | 11725.35 | 0.1079 | 0.1039 | 5.4921 |
| 156 | 12489.66 | 13383.37 | 0.1196 | 0.104 | 6.6778 | 15842.51 | 14636.79 | 0.097 | 0.0777 | -8.238 |
| 157 | 14135.59 | 14153.66 | 0.1137 | 0.115 | 0.1277 | 14356.43 | 14682.17 | 0.1038 | 0.1005 | 2.2186 |
| 158 | 11744.29 | 12424.46 | 0.1279 | 0.131 | 5.4745 | 16415.57 | 16581.52 | 0.0972 | 0.0937 | 1.0008 |
| 159 | 13233.57 | 14349.46 | 0.118 | 0.122 | 7.7766 | 14869.8 | 15151.85 | 0.1094 | 0.106 | 1.8615 |
| 160 | 12656.9 | 12548.79 | 0.1193 | 0.121 | -0.8615 | 15485.78 | 16797.62 | 0.0957 | 0.0942 | 7.8097 |
| 161 | 14844.16 | 15698.85 | 0.1134 | 0.115 | 5.4442 | 13653.8 | 14059.45 | 0.1052 | 0.1047 | 2.8852 |
| 162 | 12396.4 | 12486.19 | 0.1258 | 0.125 | 0.7191 | 12840.06 | 12981.28 | 0.1092 | 0.1111 | 1.0878 |
| 163 | 15038.19 | 15623.87 | 0.1153 | 0.101 | 3.7487 | 13163.29 | 13147.22 | 0.1053 | 0.1074 | -0.122 |
| 164 | 12993.77 | 12466.98 | 0.1223 | 0.12 | -4.2255 | 12614.59 | 12606.21 | 0.1068 | 0.1068 | -0.066 |
| 165 | 12140.5 | 12186.07 | 0.1154 | 0.113 | 0.3739 | 17923.22 | 17586.86 | 0.097 | 0.1018 | -1.913 |
| 166 | 12861.45 | 13558.64 | 0.1135 | 0.11 | 5.1421 | 15848.06 | 17106.89 | 0.0991 | 0.0964 | 7.3586 |
| 167 | 15222.46 | 17082.3 | 0.1156 | 0.12 | 10.888 | 14899.26 | 15055.21 | 0.1026 | 0.0993 | 1.0358 |
| 168 | 15155.17 | 15222.95 | 0.1134 | 0.114 | 0.4453 | 13373.49 | 13586.84 | 0.0886 | 0.0965 | 1.5702 |
| 169 | 12153.1 | 11753.28 | 0.1174 | 0.099 | -3.4018 | 13039.56 | 13731.9 | 0.1166 | 0.1066 | 5.0418 |
| 170 | 11631.02 | 11687.09 | 0.1279 | 0.127 | 0.4797 | 17265.53 | 17270.17 | 0.0951 | 0.1005 | 0.0269 |
| 171 | 11804.16 | 11942.56 | 0.1218 | 0.121 | 1.1589 | 12885.5 | 13083.79 | 0.1065 | 0.1078 | 1.5155 |
| 172 | 12791.69 | 13474.79 | 0.1313 | 0.134 | 5.0695 | 14384.6 | 15016.23 | 0.0976 | 0.099 | 4.2063 |
| 173 | 11334.16 | 12177.04 | 0.1231 | 0.105 | 6.9219 | 14152.07 | 14795.21 | 0.0969 | 0.0978 | 4.3469 |
| 174 | 14307.45 | 14397.24 | 0.1195 | 0.119 | 0.6237 | 15495.05 | 15659.48 | 0.0996 | 0.0985 | 1.0501 |
| 175 | 11402.09 | 11406.23 | 0.1225 | 0.122 | 0.0363 | 14551.2 | 14623.42 | 0.1033 | 0.1038 | 0.4939 |
| 176 | 14600.73 | 14837.6 | 0.1165 | 0.117 | 1.5964 | 14941.44 | 15087.01 | 0.1092 | 0.1062 | 0.9648 |
| 177 | 12658.73 | 13254.71 | 0.1118 | 0.106 | 4.4964 | 16644.57 | 16389.38 | 0.1001 | 0.1003 | -1.557 |
| 178 | 12876.65 | 12879.07 | 0.1236 | 0.124 | 0.0188 | 12138.02 | 12362.66 | 0.1098 | 0.1115 | 1.8171 |
| 179 | 15511.31 | 15606.75 | 0.1162 | 0.12 | 0.6115 | 14785.38 | 14860.11 | 0.1011 | 0.1032 | 0.5029 |
| 180 | 12124.64 | 12630.65 | 0.1125 | 0.106 | 4.0063 | 14231.05 | 14262.06 | 0.1007 | 0.0999 | 0.2174 |
| 181 | 14845.94 | 14918.66 | 0.1146 | 0.115 | 0.4874 | 12446.28 | 12651.42 | 0.1092 | 0.1109 | 1.6215 |
| 182 | 13675.55 | 14530.23 | 0.1159 | 0.117 | 5.8821 | 13379.89 | 13623.95 | 0.1046 | 0.1043 | 1.7914 |
| 183 | 13826 | 13931.44 | 0.1167 | 0.114 | 0.7569 | 12926.48 | 13137.49 | 0.1098 | 0.1117 | 1.6061 |
| 184 | 11960.49 | 12204.55 | 0.1284 | 0.13 | 1.9997 | 14633.89 | 15262.67 | 0.0945 | 0.096 | 4.1198 |
| 185 | 12766.85 | 12751.5 | 0.1231 | 0.123 | -0.1204 | 16813.19 | 16679.25 | 0.0963 | 0.0995 | -0.803 |
| 186 | 13839.96 | 13929.75 | 0.1198 | 0.119 | 0.6446 | 12855.78 | 12823.65 | 0.1043 | 0.1043 | -0.251 |
| 187 | 14462.08 | 15550.02 | 0.1203 | 0.108 | 6.9964 | 13469.24 | 13527.82 | 0.1041 | 0.105 | 0.433 |
| 188 | 11922.6 | 12193.02 | 0.1298 | 0.133 | 2.2178 | 13654.89 | 13823.89 | 0.0943 | 0.1019 | 1.2225 |
| 189 | 11313.03 | 11716.74 | 0.1038 | 0.105 | 3.4456 | 13516.48 | 13647.4 | 0.107 | 0.1086 | 0.9593 |
| 190 | 14285.99 | 14245.78 | 0.1116 | 0.103 | -0.2822 | 13266.08 | 13297.08 | 0.1032 | 0.1023 | 0.2332 |
| 191 | 11995.22 | 12518.28 | 0.1272 | 0.124 | 4.1783 | 14774.95 | 15403.74 | 0.0953 | 0.0966 | 4.082 |
| 192 | 13554.48 | 14409.16 | 0.1163 | 0.118 | 5.9315 | 13240.53 | 13167.6 | 0.0991 | 0.0991 | -0.554 |
| 193 | 11382.41 | 11622.23 | 0.1248 | 0.127 | 2.0635 | 14212.37 | 15538.38 | 0.1008 | 0.0909 | 8.5338 |
| 194 | 12555.14 | 12539.78 | 0.1254 | 0.126 | -0.1225 | 14498.04 | 14711.76 | 0.099 | 0.101 | 1.4528 |
| 195 | 12113.54 | 12809.73 | 0.1134 | 0.109 | 5.4348 | 12538.58 | 13021.01 | 0.096 | 0.1096 | 3.705 |
| 196 | 13331.9 | 13425.33 | 0.1097 | 0.109 | 0.6959 | 15347.17 | 15311.52 | 0.101 | 0.1046 | -0.233 |
| 197 | 11365.25 | 12007.54 | 0.1222 | 0.12 | 5.3491 | 17218.16 | 17725.35 | 0.101 | 0.101 | 2.8614 |
| 198 | 11801.5 | 12055.76 | 0.1187 | 0.119 | 2.1091 | 16720.84 | 16898 | 0.099 | 0.0955 | 1.0484 |
| 199 | 12233.18 | 12868.1 | 0.1207 | 0.118 | 4.9341 | 17398.97 | 17589.47 | 0.1066 | 0.1054 | 1.083 |
| 200 | 11380.96 | 11365.61 | 0.1232 | 0.124 | -0.1351 | 15307.28 | 15472.42 | 0.0925 | 0.0906 | 1.0674 |
| 201 | 11849.51 | 11921.65 | 0.1237 | 0.125 | 0.6051 | 14101.34 | 14068.2 | 0.1055 | 0.1061 | -0.236 |
| 202 | 11995.52 | 12067.65 | 0.1228 | 0.124 | 0.5977 | 14108.44 | 14333.09 | 0.1092 | 0.1106 | 1.5673 |
| 203 | 11911.59 | 12024.31 | 0.1268 | 0.127 | 0.9374 | 16160.08 | 14720.5 | 0.1048 | 0.091 | -9.779 |
| 204 | 15118.17 | 15738.83 | 0.1292 | 0.125 | 3.9435 | 11895.24 | 12154.44 | 0.0987 | 0.1091 | 2.1326 |
| 205 | 13138.35 | 13652.32 | 0.127 | 0.11 | 3.7647 | 15510.4 | 15843.96 | 0.1088 | 0.1066 | 2.1052 |
| 206 | 12445.05 | 12601.33 | 0.1192 | 0.102 | 1.2401 | 14705.5 | 14817.72 | 0.0942 | 0.0923 | 0.7573 |
| 207 | 11728.19 | 11998.61 | 0.1287 | 0.132 | 2.2537 | 16912.89 | 16936.24 | 0.0967 | 0.1007 | 0.1378 |
| 208 | 12690.33 | 12758.11 | 0.1177 | 0.118 | 0.5313 | 13649.56 | 13649.85 | 0.1047 | 0.1069 | 0.0022 |
| 209 | 13278.42 | 13368.21 | 0.1223 | 0.122 | 0.6717 | 14441.85 | 14848.33 | 0.095 | 0.0974 | 2.7376 |
| 210 | 11774.58 | 13568.11 | 0.1191 | 0.11 | 13.219 | 15643.33 | 16011.74 | 0.1045 | 0.0813 | 2.3009 |
| 211 | 14355.62 | 14593.78 | 0.1208 | 0.118 | 1.6319 | 17314.81 | 17115.73 | 0.0976 | 0.1023 | -1.163 |
| 212 | 12290.49 | 12428.9 | 0.1217 | 0.121 | 1.1136 | 14470.36 | 15135.92 | 0.0971 | 0.0985 | 4.3972 |
| 213 | 11867.47 | 13580.8 | 0.1202 | 0.105 | 12.616 | 14792.66 | 14899.02 | 0.0918 | 0.09 | 0.7139 |
| 214 | 12270.25 | 11518.46 | 0.1273 | 0.107 | -6.5268 | 16453.46 | 16655.47 | 0.104 | 0.1007 | 1.2129 |
| 215 | 14083.49 | 14319.47 | 0.1231 | 0.127 | 1.648 | 17007.58 | 16406.26 | 0.1022 | 0.0928 | -3.665 |
| 216 | 12177.82 | 13039.65 | 0.1203 | 0.118 | 6.6093 | 14926.4 | 17264.04 | 0.0947 | 0.0947 | 13.541 |
| 217 | 12647 | 14000.2 | 0.1227 | 0.124 | 9.6655 | 13917.35 | 14231.67 | 0.1097 | 0.1093 | 2.2086 |
| 218 | 15381.65 | 15449.43 | 0.1122 | 0.113 | 0.4387 | 14699.28 | 15382.41 | 0.0987 | 0.0986 | 4.441 |
| 219 | 15369.49 | 15147.95 | 0.1172 | 0.109 | -1.4625 | 13685.58 | 13801.23 | 0.1057 | 0.1042 | 0.838 |
| 220 | 14252.84 | 14491 | 0.123 | 0.12 | 1.6435 | 15735.37 | 15598.71 | 0.0968 | 0.0949 | -0.876 |
| 221 | 12769.72 | 13886.82 | 0.1195 | 0.124 | 8.0444 | 14802.68 | 15191.43 | 0.1105 | 0.1093 | 2.559 |
| 222 | 12812.69 | 13218.84 | 0.121 | 0.12 | 3.0725 | 18172.66 | 17982.95 | 0.1015 | 0.1015 | -1.055 |
| 223 | 11086.02 | 11489.73 | 0.1081 | 0.109 | 3.5137 | 12649.35 | 12882.49 | 0.1185 | 0.1165 | 1.8097 |
| 224 | 11626.73 | 11587.74 | 0.1233 | 0.123 | -0.3365 | 13832.49 | 14091.69 | 0.0969 | 0.0972 | 1.8394 |
| 225 | 12747.15 | 14328.85 | 0.1226 | 0.109 | 11.039 | 12050.11 | 12433.16 | 0.0994 | 0.0985 | 3.0809 |
| 226 | 14274.24 | 12577.39 | 0.1138 | 0.097 | -13.491 | 17303.94 | 17785.54 | 0.1116 | 0.1074 | 2.7078 |
| 227 | 11747.83 | 11785.62 | 0.1248 | 0.124 | 0.3206 | 14455.91 | 14507.21 | 0.1007 | 0.101 | 0.3536 |
| 228 | 14587.1 | 14367.66 | 0.1113 | 0.108 | -1.5274 | 16439.93 | 17461.59 | 0.1055 | 0.1004 | 5.8509 |
| 229 | 13117.42 | 14474.75 | 0.1217 | 0.123 | 9.3773 | 12325.38 | 12545.8 | 0.0939 | 0.0951 | 1.7569 |
| 230 | 14235.31 | 15183.12 | 0.1216 | 0.111 | 6.2426 | 16745.26 | 16610.61 | 0.0967 | 0.1008 | -0.811 |
| 231 | 12961.88 | 12309.08 | 0.1301 | 0.109 | -5.3033 | 14426.42 | 15069.56 | 0.097 | 0.0979 | 4.2678 |
| 232 | 14335.05 | 14402.83 | 0.1144 | 0.115 | 0.4706 | 14666.26 | 15014.22 | 0.1072 | 0.1045 | 2.3175 |
| 233 | 12947.82 | 13704.44 | 0.1123 | 0.102 | 5.521 | 16843.53 | 17038.46 | 0.103 | 0.1087 | 1.1441 |
| 234 | 14648.66 | 14885.73 | 0.1171 | 0.117 | 1.5926 | 13756.01 | 13780.06 | 0.1019 | 0.1005 | 0.1746 |
| 235 | 14137.69 | 14375.86 | 0.1222 | 0.12 | 1.6567 | 13821.78 | 15174.66 | 0.1041 | 0.0984 | 8.9154 |
| 236 | 16399.42 | 16496.77 | 0.1358 | 0.111 | 0.5901 | 14074.83 | 14083.91 | 0.1028 | 0.105 | 0.0645 |
| 237 | 12080.46 | 12110.96 | 0.1193 | 0.12 | 0.2519 | 16994.76 | 17149.49 | 0.1088 | 0.1099 | 0.9022 |
| 238 | 13198.04 | 13303.48 | 0.1286 | 0.126 | 0.7926 | 13083.67 | 13010.74 | 0.1009 | 0.1009 | -0.561 |
| 239 | 19500.84 | 16785.03 | 0.1309 | 0.108 | -16.18 | 15952.5 | 15880.99 | 0.0975 | 0.1012 | -0.45 |
| 240 | 12634.38 | 12693.87 | 0.1173 | 0.123 | 0.4687 | 14214.93 | 14612.79 | 0.1098 | 0.1049 | 2.7227 |
| 241 | 16830.91 | 16080.94 | 0.1347 | 0.113 | -4.6637 | 12356.6 | 12306.8 | 0.1101 | 0.1083 | -0.405 |
| 242 | 13787.74 | 14234.36 | 0.1176 | 0.12 | 3.1376 | 14505.08 | 14620.23 | 0.0967 | 0.0949 | 0.7876 |
| 243 | 12420.05 | 12697.83 | 0.126 | 0.123 | 2.1876 | 15963.98 | 16589.84 | 0.0919 | 0.0933 | 3.7725 |
| 244 | 18361.42 | 17347.94 | 0.1335 | 0.11 | -5.8421 | 14759.01 | 15640.93 | 0.096 | 0.0972 | 5.6386 |
| 245 | 16638.06 | 15933.87 | 0.1261 | 0.115 | -4.4195 | 13250.12 | 13436.9 | 0.0939 | 0.0954 | 1.39 |
| 246 | 12581.62 | 12714.67 | 0.1172 | 0.116 | 1.0464 | 15997.51 | 16217.98 | 0.1064 | 0.1056 | 1.3594 |
| 247 | 16749.88 | 17050.08 | 0.1202 | 0.119 | 1.7607 | 13704.39 | 13915.39 | 0.1096 | 0.1113 | 1.5163 |
| 248 | 13779.48 | 13360.66 | 0.1246 | 0.107 | -3.1347 | 15799.37 | 16177.12 | 0.1057 | 0.1051 | 2.3351 |
| 249 | 15788.5 | 15955.99 | 0.1261 | 0.125 | 1.0497 | 15004.31 | 15152.68 | 0.0999 | 0.1019 | 0.9792 |
| 250 | 14332.37 | 14757.93 | 0.116 | 0.119 | 2.8836 | 12395.5 | 12618.14 | 0.1003 | 0.1088 | 1.7644 |
| 251 | 13063.89 | 13183.97 | 0.1212 | 0.122 | 0.9109 | 16932.15 | 15987.84 | 0.1039 | 0.0972 | -5.906 |
| 252 | 12141.09 | 12499.92 | 0.1266 | 0.12 | 2.8706 | 13142.58 | 13072.78 | 0.1061 | 0.1054 | -0.534 |
| 253 | 12208.64 | 13237.76 | 0.1256 | 0.114 | 7.7741 | 16356.56 | 16285.05 | 0.0972 | 0.1007 | -0.439 |
| 254 | 13138.94 | 13417.64 | 0.127 | 0.129 | 2.0771 | 13525.04 | 13740.39 | 0.0981 | 0.1088 | 1.5673 |
| 255 | 11782.05 | 11903.89 | 0.1197 | 0.12 | 1.0235 | 15809.19 | 15763.75 | 0.1159 | 0.1073 | -0.288 |
| 256 | 12535.6 | 12625.39 | 0.1244 | 0.124 | 0.7112 | 12581.24 | 13081.66 | 0.1136 | 0.1048 | 3.8253 |
| 257 | 12861.18 | 13115.44 | 0.119 | 0.119 | 1.9387 | 14410.66 | 16198.69 | 0.0997 | 0.0961 | 11.038 |
| 258 | 12742.04 | 12880.44 | 0.1211 | 0.12 | 1.0745 | 14165.81 | 14334.6 | 0.1091 | 0.1093 | 1.1775 |
| 259 | 15429.45 | 15674.01 | 0.1188 | 0.119 | 1.5603 | 14353.17 | 14521.96 | 0.1076 | 0.1078 | 1.1623 |
| 260 | 12433.38 | 12523.17 | 0.1253 | 0.125 | 0.717 | 15075.99 | 15704.78 | 0.0953 | 0.0967 | 4.0038 |
| 261 | 13039.64 | 13246.33 | 0.1236 | 0.127 | 1.5604 | 11248.35 | 11685.46 | 0.105 | 0.1035 | 3.7406 |
| 262 | 11449.96 | 11772.16 | 0.11 | 0.106 | 2.737 | 13088.32 | 13084.39 | 0.1051 | 0.1029 | -0.03 |
| 263 | 13157.35 | 13247.14 | 0.1203 | 0.12 | 0.6778 | 15757.51 | 15788.52 | 0.0968 | 0.0961 | 0.1964 |
| 264 | 14330.91 | 14398.69 | 0.1162 | 0.117 | 0.4707 | 15695.51 | 15736.73 | 0.0941 | 0.0925 | 0.2619 |
| 265 | 11798.04 | 12068.46 | 0.1286 | 0.131 | 2.2407 | 14925.21 | 16231.31 | 0.0944 | 0.089 | 8.0468 |
| 266 | 12592.31 | 13151.25 | 0.1093 | 0.103 | 4.2501 | 15927.46 | 17166.78 | 0.094 | 0.0883 | 7.2193 |
| 267 | 11111.99 | 11450.52 | 0.126 | 0.127 | 2.9564 | 13402.78 | 13526.42 | 0.1033 | 0.1017 | 0.9141 |
| 268 | 11924.2 | 11815.71 | 0.1178 | 0.113 | -0.9182 | 13375.1 | 13460.45 | 0.1116 | 0.1115 | 0.6341 |
| 269 | 12171.92 | 12254.13 | 0.1214 | 0.123 | 0.6709 | 16951.81 | 17209.68 | 0.1036 | 0.1083 | 1.4984 |
| 270 | 14189.34 | 13999.63 | 0.1197 | 0.108 | -1.3551 | 13329.83 | 13540.83 | 0.1084 | 0.1102 | 1.5583 |
| 271 | 12500.11 | 12800.82 | 0.1264 | 0.123 | 2.3492 | 14218.48 | 14373.63 | 0.1054 | 0.1049 | 1.0794 |
| 272 | 13875.77 | 12323.4 | 0.1086 | 0.1 | -12.597 | 15875.39 | 16070.62 | 0.1015 | 0.1079 | 1.2148 |
| 273 | 13109.01 | 14195.82 | 0.1176 | 0.118 | 7.6559 | 15221.92 | 16333.17 | 0.0926 | 0.0895 | 6.8036 |
| 274 | 11104.22 | 11382.92 | 0.1261 | 0.129 | 2.4484 | 13126.07 | 12922.72 | 0.1107 | 0.108 | -1.574 |
| 275 | 12990.03 | 12932.04 | 0.1221 | 0.124 | -0.4484 | 13416.94 | 13532.59 | 0.1086 | 0.1104 | 0.8546 |
| 276 | 17165.14 | 17465.35 | 0.1193 | 0.118 | 1.7189 | 15293.19 | 15976.32 | 0.0983 | 0.0981 | 4.2759 |
| 277 | 12931.31 | 12459.35 | 0.1272 | 0.108 | -3.788 | 13526.48 | 15757.94 | 0.0883 | 0.0943 | 14.161 |
| 278 | 16009.85 | 15792.28 | 0.1082 | 0.107 | -1.3777 | 12186.83 | 12207.04 | 0.1095 | 0.1107 | 0.1655 |
| 279 | 14148.12 | 14215.91 | 0.1152 | 0.116 | 0.4768 | 14602.71 | 14652.08 | 0.101 | 0.0997 | 0.337 |
| 280 | 17567.87 | 17068.61 | 0.1266 | 0.11 | -2.925 | 15385.26 | 15399.4 | 0.1095 | 0.1025 | 0.0918 |
| 281 | 12776.68 | 12772.63 | 0.1236 | 0.123 | -0.0318 | 13378.58 | 13401.3 | 0.1043 | 0.1063 | 0.1695 |
| 282 | 13590.6 | 13658.38 | 0.1182 | 0.119 | 0.4963 | 15139.79 | 15170.79 | 0.0984 | 0.0976 | 0.2044 |
| 283 | 15112.47 | 15849.04 | 0.1184 | 0.124 | 4.6474 | 16184.99 | 16062.77 | 0.0989 | 0.1031 | -0.761 |
| 284 | 11464.81 | 12915.99 | 0.1207 | 0.123 | 11.236 | 13076.72 | 12960.15 | 0.1054 | 0.1058 | -0.899 |
| 285 | 12777.71 | 13012.48 | 0.1221 | 0.125 | 1.8042 | 15928.21 | 16505.2 | 0.0941 | 0.0918 | 3.4958 |
| 286 | 12849.7 | 14219.46 | 0.1227 | 0.124 | 9.633 | 13642.91 | 14326.34 | 0.1033 | 0.1019 | 4.7705 |
| 287 | 13765.86 | 13871.3 | 0.1235 | 0.121 | 0.7601 | 13060.96 | 12948.53 | 0.1052 | 0.1046 | -0.868 |
| 288 | 11390.4 | 11878.3 | 0.1259 | 0.126 | 4.1075 | 14293.57 | 14555.7 | 0.1032 | 0.1093 | 1.8009 |
| 289 | 14461.2 | 13678.65 | 0.1121 | 0.112 | -5.7209 | 15474.9 | 14081.31 | 0.1043 | 0.0815 | -9.897 |
| 290 | 13762.59 | 13860.37 | 0.1139 | 0.114 | 0.7055 | 14222.47 | 14382.06 | 0.0986 | 0.1026 | 1.1096 |
| 291 | 13365.2 | 13900.17 | 0.1233 | 0.125 | 3.8487 | 17493.52 | 17358.87 | 0.0981 | 0.102 | -0.776 |
| 292 | 12105.64 | 12598.4 | 0.1259 | 0.123 | 3.9113 | 14759.98 | 14724.33 | 0.0926 | 0.0942 | -0.242 |
| 293 | 15997.63 | 15788.13 | 0.1121 | 0.111 | -1.3269 | 13256.84 | 13487.34 | 0.1088 | 0.1104 | 1.709 |
| 294 | 11711.55 | 12208.57 | 0.1272 | 0.122 | 4.0711 | 14642.29 | 14630.78 | 0.099 | 0.098 | -0.079 |
| 295 | 12738.64 | 12839.06 | 0.1108 | 0.11 | 0.7821 | 14481.26 | 14690.76 | 0.1063 | 0.1053 | 1.426 |
| 296 | 12679.26 | 13023.73 | 0.121 | 0.103 | 2.645 | 14559.3 | 14549.89 | 0.1077 | 0.1092 | -0.065 |
| 297 | 12967.94 | 13058.91 | 0.1257 | 0.124 | 0.6966 | 13397.58 | 13420.3 | 0.1064 | 0.1085 | 0.1693 |
| 298 | 17623.97 | 15665.73 | 0.1255 | 0.104 | -12.5 | 14437.02 | 14785.43 | 0.1066 | 0.1069 | 2.3564 |
| 299 | 12968.19 | 13073.63 | 0.1284 | 0.126 | 0.8065 | 18945.1 | 18360.06 | 0.099 | 0.09 | -3.187 |
| 300 | 14227.54 | 14347.62 | 0.1145 | 0.116 | 0.837 | 14196.96 | 14552.73 | 0.1044 | 0.1045 | 2.4447 |
| 301 | 14758.85 | 14864.29 | 0.1091 | 0.107 | 0.7094 | 13252.65 | 13359.8 | 0.1012 | 0.103 | 0.8021 |
| 302 | 11102.97 | 11639.87 | 0.1237 | 0.121 | 4.6126 | 13224.73 | 13246.36 | 0.1024 | 0.1024 | 0.1633 |
| 303 | 13070.84 | 13155.28 | 0.1194 | 0.119 | 0.6418 | 16230.44 | 14874.93 | 0.1029 | 0.081 | -9.113 |
| 304 | 12803.79 | 12518.9 | 0.1227 | 0.125 | -2.2757 | 14384.51 | 14776.1 | 0.1026 | 0.0996 | 2.6502 |
| 305 | 11675.87 | 13107.65 | 0.1199 | 0.122 | 10.923 | 15667.56 | 16033.3 | 0.0971 | 0.0933 | 2.2811 |
| 306 | 11649.2 | 12080.26 | 0.1272 | 0.125 | 3.5683 | 14365.04 | 14248.47 | 0.1049 | 0.1052 | -0.818 |
| 307 | 14581.61 | 15345.78 | 0.1304 | 0.115 | 4.9797 | 14233.68 | 14059.03 | 0.1022 | 0.0991 | -1.242 |
| 308 | 11819.88 | 12652.93 | 0.1279 | 0.115 | 6.5839 | 13875.72 | 13961.08 | 0.1101 | 0.1099 | 0.6114 |
| 309 | 12698.21 | 12720.64 | 0.1223 | 0.122 | 0.1763 | 11567.47 | 11869.01 | 0.103 | 0.1022 | 2.5406 |
| 310 | 12927.79 | 13123.96 | 0.1101 | 0.103 | 1.4947 | 15246.04 | 15365.83 | 0.0948 | 0.0929 | 0.7796 |
| 311 | 19377.2 | 16673.82 | 0.1328 | 0.109 | -16.213 | 15709.19 | 15817.65 | 0.1009 | 0.0979 | 0.6857 |
| 312 | 16846.92 | 14990.7 | 0.1282 | 0.099 | -12.383 | 14289.89 | 14184.33 | 0.1027 | 0.1023 | -0.744 |
| 313 | 12448.72 | 13882.41 | 0.1201 | 0.124 | 10.327 | 13139.29 | 13349.5 | 0.1036 | 0.1015 | 1.5747 |
| 314 | 12499.27 | 12589.06 | 0.1242 | 0.124 | 0.7133 | 12574.52 | 12597.25 | 0.1062 | 0.1084 | 0.1804 |
| 315 | 11733.78 | 12836.86 | 0.1209 | 0.105 | 8.5931 | 16191.86 | 16376.76 | 0.1071 | 0.1007 | 1.1291 |
| 316 | 12143.84 | 12381.53 | 0.1285 | 0.129 | 1.9198 | 15330.7 | 15959.48 | 0.0957 | 0.0971 | 3.9399 |
| 317 | 14027.61 | 14299.74 | 0.1138 | 0.117 | 1.9031 | 12750.81 | 12941.1 | 0.1095 | 0.1108 | 1.4705 |
| 318 | 16065.38 | 16748.48 | 0.113 | 0.116 | 4.0786 | 15698.92 | 16407.21 | 0.097 | 0.0978 | 4.3169 |
| 319 | 12991.13 | 13687.54 | 0.1054 | 0.111 | 5.0879 | 14535.64 | 14531.7 | 0.1033 | 0.1014 | -0.027 |
| 320 | 12149.12 | 12167.19 | 0.1154 | 0.117 | 0.1486 | 14576.28 | 14715.57 | 0.0914 | 0.0957 | 0.9465 |
| 321 | 13216.14 | 13303.42 | 0.1188 | 0.119 | 0.6561 | 14175.43 | 14493.59 | 0.1031 | 0.0992 | 2.1952 |
| 322 | 15301.77 | 15078.43 | 0.1197 | 0.109 | -1.4812 | 15892.83 | 16575.97 | 0.0962 | 0.0962 | 4.1213 |
| 323 | 12587.33 | 12228.75 | 0.1242 | 0.118 | -2.9323 | 15600.05 | 16283.19 | 0.0973 | 0.0972 | 4.1954 |
| 324 | 11520.85 | 12286.53 | 0.1152 | 0.109 | 6.2319 | 13485.1 | 13710.87 | 0.0951 | 0.1008 | 1.6467 |
| 325 | 11837.77 | 12071.33 | 0.1293 | 0.131 | 1.9348 | 17234.26 | 17424.76 | 0.1072 | 0.106 | 1.0933 |
| 326 | 12182.39 | 13108.41 | 0.1201 | 0.122 | 7.0643 | 12188.4 | 12371.24 | 0.1022 | 0.1038 | 1.478 |
| 327 | 12488.99 | 12743.25 | 0.1204 | 0.121 | 1.9953 | 15840.16 | 16016.81 | 0.0999 | 0.0964 | 1.1029 |
| 328 | 14564.67 | 14309.36 | 0.1234 | 0.121 | -1.7842 | 16042.27 | 16314.57 | 0.0967 | 0.093 | 1.6691 |
| 329 | 13518.76 | 13974.07 | 0.1254 | 0.12 | 3.2582 | 14361.85 | 14373.36 | 0.0961 | 0.0958 | 0.0801 |
| 330 | 17080.89 | 15319.14 | 0.1318 | 0.107 | -11.5 | 13733.92 | 14157.44 | 0.1066 | 0.1026 | 2.9915 |
| 331 | 12942.45 | 13080.86 | 0.1164 | 0.116 | 1.0581 | 13022.58 | 13151.57 | 0.104 | 0.1035 | 0.9808 |
| 332 | 16183.25 | 13916.86 | 0.1228 | 0.098 | -16.285 | 13694.83 | 13942.02 | 0.1128 | 0.1116 | 1.773 |
| 333 | 12131.23 | 12203.36 | 0.1262 | 0.128 | 0.5911 | 14586.1 | 14402.97 | 0.1037 | 0.1019 | -1.272 |
| 334 | 14462.99 | 14886.67 | 0.1183 | 0.12 | 2.846 | 16210.74 | 17881.4 | 0.0943 | 0.0869 | 9.343 |
| 335 | 12699.98 | 12767.76 | 0.1177 | 0.118 | 0.5309 | 14445.58 | 14517.79 | 0.1038 | 0.1043 | 0.4974 |
| 336 | 14877.54 | 15560.64 | 0.1176 | 0.12 | 4.3899 | 16719.24 | 16921.25 | 0.1057 | 0.1024 | 1.1938 |
| 337 | 11763.51 | 11726.23 | 0.1252 | 0.124 | -0.3179 | 14841.54 | 14508.02 | 0.1005 | 0.0954 | -2.299 |
| 338 | 11507.95 | 11646.35 | 0.125 | 0.124 | 1.1884 | 12027.18 | 12019.91 | 0.1069 | 0.1088 | -0.061 |
| 339 | 12379.51 | 13523.69 | 0.12 | 0.122 | 8.4605 | 13264.44 | 13290.09 | 0.1066 | 0.1087 | 0.193 |
| 340 | 11565.87 | 11588.3 | 0.1247 | 0.125 | 0.1935 | 13235.18 | 13544.18 | 0.0957 | 0.1058 | 2.2814 |
| 341 | 13689.06 | 14007.71 | 0.1043 | 0.098 | 2.2748 | 15831.08 | 15846.64 | 0.1022 | 0.1055 | 0.0982 |
| 342 | 13396.02 | 13507.52 | 0.1187 | 0.119 | 0.8255 | 12568.96 | 12583.81 | 0.1109 | 0.1094 | 0.118 |
| 343 | 11684.72 | 11873.21 | 0.1259 | 0.123 | 1.5875 | 12865.12 | 12850.48 | 0.106 | 0.104 | -0.114 |
| 344 | 12676.65 | 12673.72 | 0.1232 | 0.123 | -0.0231 | 18217.7 | 17594.37 | 0.0997 | 0.0907 | -3.543 |
| 345 | 13828.42 | 13948.5 | 0.1181 | 0.119 | 0.8609 | 12624.67 | 12676.09 | 0.1007 | 0.0989 | 0.4057 |
| 346 | 18954.65 | 16543.32 | 0.1337 | 0.106 | -14.576 | 14121.41 | 14228.57 | 0.0999 | 0.1016 | 0.7531 |
| 347 | 15345.93 | 16069.69 | 0.1188 | 0.122 | 4.5039 | 14346.75 | 15376.67 | 0.0982 | 0.0965 | 6.6979 |
| 348 | 11824.43 | 11962.83 | 0.1214 | 0.121 | 1.157 | 14821.94 | 14837 | 0.103 | 0.0803 | 0.1015 |
| 349 | 12217.65 | 12754.55 | 0.1256 | 0.123 | 4.2095 | 14225.15 | 16386.94 | 0.0917 | 0.0931 | 13.192 |
| 350 | 13108.97 | 12193.25 | 0.1108 | 0.1 | -7.5101 | 14770.53 | 14855.26 | 0.0995 | 0.1012 | 0.5704 |
| 351 | 12345.88 | 12202.21 | 0.1224 | 0.121 | -1.1775 | 13488.2 | 14084.39 | 0.1037 | 0.0997 | 4.233 |
| 352 | 11985.58 | 12543.61 | 0.1207 | 0.103 | 4.4487 | 15564.81 | 14062.73 | 0.1073 | 0.0841 | -10.68 |
| 353 | 14806.93 | 15751.17 | 0.1272 | 0.109 | 5.9948 | 14388.03 | 15013.89 | 0.0962 | 0.0976 | 4.1685 |
| 354 | 15441.19 | 16921.24 | 0.1182 | 0.123 | 8.7467 | 13824.28 | 13937.88 | 0.1081 | 0.1074 | 0.8151 |
| 355 | 13525.45 | 13521.31 | 0.1107 | 0.109 | -0.0306 | 14727.12 | 15035.58 | 0.1063 | 0.1059 | 2.0515 |
| 356 | 12314.68 | 12997.78 | 0.1315 | 0.134 | 5.2555 | 13251.61 | 13426.96 | 0.1087 | 0.1072 | 1.306 |
| 357 | 13861.41 | 13929.19 | 0.1171 | 0.117 | 0.4866 | 13950.43 | 14295.87 | 0.1128 | 0.1081 | 2.4164 |
| 358 | 12152.98 | 12223.4 | 0.0981 | 0.1 | 0.5761 | 14313.24 | 14942.02 | 0.0965 | 0.0979 | 4.2082 |
| 359 | 11488.83 | 11485.9 | 0.1212 | 0.121 | -0.0255 | 15013.85 | 15090.21 | 0.0925 | 0.0909 | 0.506 |
| 360 | 11635.82 | 12015.44 | 0.1215 | 0.107 | 3.1594 | 14158.03 | 14433 | 0.0995 | 0.0974 | 1.9052 |
| 361 | 17607.31 | 15612.01 | 0.1289 | 0.107 | -12.781 | 14162.47 | 14522.85 | 0.108 | 0.105 | 2.4815 |
| 362 | 11406.4 | 11880.79 | 0.123 | 0.127 | 3.9929 | 13590.77 | 15883.15 | 0.0995 | 0.0986 | 14.433 |
| 363 | 13529.76 | 14039.38 | 0.1227 | 0.124 | 3.6299 | 15821.42 | 15975.53 | 0.1082 | 0.1049 | 0.9646 |
| 364 | 14543.66 | 15092.41 | 0.1153 | 0.119 | 3.6359 | 15500.34 | 16126.2 | 0.0946 | 0.0959 | 3.881 |
| 365 | 18940.42 | 16500.06 | 0.1329 | 0.107 | -14.79 | 16665.05 | 17048.9 | 0.1095 | 0.1022 | 2.2515 |
| 366 | 11324.04 | 11966.33 | 0.1226 | 0.12 | 5.3675 | 13529.94 | 15282.02 | 0.1012 | 0.0976 | 11.465 |
| 367 | 12412 | 12424.43 | 0.1268 | 0.129 | 0.1 | 13903.36 | 14220.01 | 0.1126 | 0.1105 | 2.2268 |
| 368 | 16633.91 | 16108.6 | 0.1215 | 0.106 | -3.261 | 15934.07 | 16400.05 | 0.1022 | 0.1007 | 2.8413 |
| 369 | 15240.39 | 15489.51 | 0.1112 | 0.113 | 1.6083 | 14129.42 | 14555.16 | 0.1077 | 0.1068 | 2.925 |
| 370 | 12344.6 | 12106.33 | 0.1235 | 0.122 | -1.9681 | 14558.79 | 14341.21 | 0.0991 | 0.0988 | -1.517 |
| 371 | 12582.46 | 11918.46 | 0.1272 | 0.108 | -5.5712 | 18136.57 | 18040.92 | 0.0982 | 0.1017 | -0.53 |
| 372 | 12302.25 | 12527.39 | 0.1223 | 0.103 | 1.7972 | 12902.97 | 13129.24 | 0.1043 | 0.1107 | 1.7234 |
| 373 | 12293.72 | 12325.64 | 0.1299 | 0.131 | 0.259 | 17141.44 | 16980.8 | 0.1025 | 0.0879 | -0.946 |
| 374 | 11549.39 | 11736.17 | 0.1226 | 0.12 | 1.5915 | 13203.02 | 13550.71 | 0.0993 | 0.0972 | 2.5659 |
| 375 | 14087.21 | 13912.14 | 0.1187 | 0.109 | -1.2583 | 13107.11 | 13325.69 | 0.1046 | 0.1049 | 1.6403 |
| 376 | 11674.99 | 12188.34 | 0.1218 | 0.108 | 4.2118 | 13492.38 | 13725.01 | 0.1042 | 0.1021 | 1.695 |
| 377 | 13401.08 | 13468.86 | 0.1167 | 0.117 | 0.5032 | 12537.96 | 12812.1 | 0.1123 | 0.1065 | 2.1397 |
| 378 | 18141.04 | 15679.88 | 0.1326 | 0.107 | -15.696 | 12677.51 | 12824.29 | 0.1085 | 0.1063 | 1.1445 |
| 379 | 13268.86 | 13286.94 | 0.1138 | 0.115 | 0.136 | 14802.14 | 14818.5 | 0.1032 | 0.1024 | 0.1104 |
| 380 | 15331.53 | 15399.31 | 0.1138 | 0.114 | 0.4402 | 17453.6 | 16946.24 | 0.0957 | 0.1015 | -2.994 |
| 381 | 12877.76 | 12967.55 | 0.1277 | 0.127 | 0.6924 | 14072.79 | 14221.29 | 0.1077 | 0.1068 | 1.0442 |
| 382 | 13907.8 | 14013.25 | 0.1258 | 0.124 | 0.7524 | 14150.31 | 14178.09 | 0.1042 | 0.1022 | 0.1959 |
| 383 | 12903.68 | 13586.78 | 0.1283 | 0.131 | 5.0277 | 15842.28 | 15893.58 | 0.0983 | 0.0986 | 0.3228 |
| 384 | 11940.63 | 12922.66 | 0.1244 | 0.126 | 7.5993 | 13299.5 | 13256.66 | 0.0995 | 0.1007 | -0.323 |
| 385 | 12942.35 | 13032.14 | 0.1162 | 0.116 | 0.689 | 12731.3 | 12740.38 | 0.1063 | 0.1088 | 0.0713 |
| 386 | 13753.26 | 14203.92 | 0.1057 | 0.1 | 3.1728 | 12800.88 | 12862.81 | 0.1117 | 0.1115 | 0.4814 |
| 387 | 13018.89 | 13169.6 | 0.1138 | 0.114 | 1.1444 | 15303.75 | 14801.74 | 0.1097 | 0.0999 | -3.392 |
| 388 | 13570.31 | 13714.57 | 0.1242 | 0.106 | 1.0519 | 17345.02 | 17249.37 | 0.0976 | 0.1012 | -0.555 |
| 389 | 11854.67 | 11892.45 | 0.1244 | 0.124 | 0.3177 | 16179.45 | 16850.87 | 0.0956 | 0.0967 | 3.9845 |
| 390 | 12592.36 | 12737.33 | 0.0936 | 0.097 | 1.1382 | 13127.39 | 13829.52 | 0.1019 | 0.1027 | 5.0771 |
| 391 | 12785.98 | 12868.82 | 0.1174 | 0.116 | 0.6437 | 14533.12 | 15057.65 | 0.1054 | 0.1048 | 3.4834 |
| 392 | 13351.29 | 12445.65 | 0.1245 | 0.105 | -7.2767 | 16130.23 | 14737.14 | 0.1051 | 0.0832 | -9.453 |
| 393 | 13030.26 | 13052.69 | 0.1264 | 0.126 | 0.1718 | 15049.28 | 15678.06 | 0.0963 | 0.0977 | 4.0106 |
| 394 | 12097.63 | 12344.11 | 0.1291 | 0.13 | 1.9968 | 16334.46 | 16963.25 | 0.094 | 0.0953 | 3.7068 |
| 395 | 12341.3 | 13281.46 | 0.1172 | 0.119 | 7.0787 | 13275.51 | 13332.88 | 0.1019 | 0.1024 | 0.4303 |
| 396 | 13543.09 | 12423.23 | 0.1243 | 0.105 | -9.0143 | 16210.61 | 16994.75 | 0.0951 | 0.0956 | 4.614 |
| 397 | 12144.82 | 11469.22 | 0.1156 | 0.104 | -5.8905 | 14166.08 | 14308.5 | 0.1026 | 0.1005 | 0.9954 |
| 398 | 14033.33 | 14153.42 | 0.1181 | 0.119 | 0.8485 | 14206.4 | 14335.39 | 0.1013 | 0.1009 | 0.8998 |
| 399 | 12508.49 | 12419.38 | 0.1221 | 0.123 | -0.7176 | 17001.53 | 16866.89 | 0.096 | 0.1 | -0.798 |
| 400 | 11910.82 | 12278.61 | 0.1181 | 0.12 | 2.9953 | 15409.81 | 14066.02 | 0.1054 | 0.0839 | -9.554 |
| 401 | 18836.01 | 16385.89 | 0.1334 | 0.106 | -14.953 | 13742.38 | 13827.73 | 0.1114 | 0.1112 | 0.6173 |
| 402 | 15111.03 | 15053.66 | 0.1171 | 0.1 | -0.3811 | 12177.54 | 12130.97 | 0.1047 | 0.1039 | -0.384 |
| 403 | 13552.73 | 13691.02 | 0.1167 | 0.117 | 1.01 | 16335.73 | 14999.93 | 0.1048 | 0.0832 | -8.905 |
| 404 | 13043.23 | 13651.63 | 0.1111 | 0.106 | 4.4567 | 13096.86 | 13139.2 | 0.1004 | 0.0993 | 0.3222 |
| 405 | 13492.09 | 13597.54 | 0.1281 | 0.126 | 0.7754 | 17227.74 | 17211.68 | 0.0954 | 0.1006 | -0.093 |
| 406 | 12712.54 | 12850.95 | 0.1165 | 0.116 | 1.077 | 14602.29 | 14775.72 | 0.0997 | 0.0985 | 1.1738 |
| 407 | 12667.82 | 13084.6 | 0.1283 | 0.128 | 3.1852 | 13459.88 | 13386.95 | 0.1015 | 0.1015 | -0.545 |
| 408 | 11913.16 | 13040.98 | 0.1212 | 0.119 | 8.6483 | 15086.91 | 15770.05 | 0.0967 | 0.0966 | 4.3319 |
| 409 | 14315.32 | 12898.17 | 0.1165 | 0.101 | -10.987 | 14831.73 | 15460.51 | 0.0947 | 0.0961 | 4.067 |
| 410 | 12169.37 | 11724.31 | 0.1259 | 0.12 | -3.7961 | 14114.95 | 14183.44 | 0.1078 | 0.102 | 0.4829 |
| 411 | 18715.68 | 17046.72 | 0.1336 | 0.109 | -9.7905 | 15040.76 | 14601.68 | 0.109 | 0.1 | -3.007 |
| 412 | 12511.42 | 12929.24 | 0.1277 | 0.11 | 3.2316 | 13662.92 | 13812.21 | 0.1099 | 0.1091 | 1.0809 |
| 413 | 13587.93 | 14196.34 | 0.1093 | 0.105 | 4.2857 | 12110.32 | 12163.75 | 0.1061 | 0.1067 | 0.4393 |
| 414 | 12981.15 | 13455.96 | 0.1285 | 0.112 | 3.5286 | 14707.21 | 14757 | 0.1032 | 0.1039 | 0.3374 |
| 415 | 13699.61 | 13789.4 | 0.1209 | 0.121 | 0.6512 | 17394.67 | 17323.16 | 0.0935 | 0.0968 | -0.413 |
| 416 | 13485.99 | 14585.28 | 0.1253 | 0.129 | 7.537 | 13560.16 | 13861.55 | 0.1093 | 0.1087 | 2.1743 |
| 417 | 14797.21 | 13390.06 | 0.1125 | 0.099 | -10.509 | 14480.49 | 14413.42 | 0.1035 | 0.1024 | -0.465 |
| 418 | 11636.14 | 11890.41 | 0.1243 | 0.125 | 2.1384 | 14147.61 | 14810.04 | 0.0961 | 0.0972 | 4.4728 |
| 419 | 10877.53 | 11163.72 | 0.1252 | 0.127 | 2.5636 | 12253.61 | 12311.68 | 0.1024 | 0.1063 | 0.4717 |
| 420 | 12972.29 | 13254.73 | 0.1129 | 0.104 | 2.1309 | 13128.32 | 13235.18 | 0.1052 | 0.1054 | 0.8074 |
| 421 | 12382.99 | 12831.2 | 0.1154 | 0.104 | 3.4931 | 13439.56 | 13322.99 | 0.1059 | 0.1063 | -0.875 |
| 422 | 16503.39 | 16621.17 | 0.1223 | 0.122 | 0.7086 | 16558.48 | 16558.48 | 0.101 | 0.1058 | 0 |
| 423 | 13087.23 | 13105.31 | 0.1158 | 0.117 | 0.1379 | 13816.11 | 13783.98 | 0.1018 | 0.1018 | -0.233 |
| 424 | 12233.34 | 12712.78 | 0.1138 | 0.106 | 3.7714 | 13960.83 | 14589.62 | 0.0951 | 0.0965 | 4.3098 |
| 425 | 12995.27 | 14132.17 | 0.1161 | 0.116 | 8.0448 | 15808.42 | 15868.42 | 0.0942 | 0.0924 | 0.3781 |
| 426 | 11914.31 | 13486.6 | 0.114 | 0.106 | 11.658 | 13144.14 | 13364.77 | 0.0978 | 0.0972 | 1.6508 |
| 427 | 14753.31 | 14821.09 | 0.1147 | 0.115 | 0.4573 | 13236.09 | 13501.07 | 0.0957 | 0.0952 | 1.9626 |
| 428 | 16040.62 | 16195.89 | 0.1109 | 0.107 | 0.9587 | 13977.49 | 15440.37 | 0.1052 | 0.0963 | 9.4744 |
| 429 | 12443.75 | 13763.29 | 0.1155 | 0.106 | 9.5873 | 12391.18 | 13103.43 | 0.1093 | 0.1035 | 5.4356 |
| 430 | 13606.35 | 13903.35 | 0.1081 | 0.095 | 2.1362 | 15068.57 | 15039.57 | 0.0995 | 0.0993 | -0.193 |
| 431 | 12678.1 | 12816.51 | 0.1202 | 0.119 | 1.0799 | 15205.7 | 15368.42 | 0.1027 | 0.1018 | 1.0588 |
| 432 | 12990.95 | 13269.65 | 0.1276 | 0.13 | 2.1003 | 16691.97 | 17368.07 | 0.1025 | 0.0999 | 3.8928 |
| 433 | 10718.73 | 11589.01 | 0.1328 | 0.123 | 7.5095 | 14917.08 | 15557.08 | 0.0971 | 0.0973 | 4.1139 |
| 434 | 13714.66 | 13670.72 | 0.1104 | 0.111 | -0.3214 | 12989.02 | 12967.31 | 0.1049 | 0.1043 | -0.167 |
| 435 | 12233.61 | 12876.15 | 0.1133 | 0.109 | 4.9902 | 13375.43 | 13406.43 | 0.1026 | 0.1018 | 0.2313 |
| 436 | 10775.63 | 10772.7 | 0.1308 | 0.13 | -0.0272 | 14927.56 | 15610.7 | 0.0995 | 0.0993 | 4.3761 |
| 437 | 13782.02 | 12996.55 | 0.1105 | 0.111 | -6.0437 | 14235.61 | 14323.27 | 0.099 | 0.1008 | 0.612 |
| 438 | 12009.07 | 11954.31 | 0.1198 | 0.115 | -0.4581 | 12964.27 | 12890.83 | 0.1045 | 0.1017 | -0.57 |
| 439 | 12017.27 | 12423.42 | 0.1215 | 0.121 | 3.2692 | 12881.55 | 12953.76 | 0.1045 | 0.105 | 0.5575 |
| 440 | 12082.44 | 12286.59 | 0.1261 | 0.128 | 1.6615 | 16504.68 | 15581.94 | 0.1022 | 0.0923 | -5.922 |
| 441 | 11943.36 | 12015.49 | 0.1198 | 0.121 | 0.6003 | 12399.82 | 12664.79 | 0.098 | 0.0974 | 2.0922 |
| 442 | 12548.73 | 12491.33 | 0.1205 | 0.101 | -0.4595 | 17679.73 | 17726.51 | 0.1001 | 0.1035 | 0.2639 |
| 443 | 16537.98 | 17817.39 | 0.1249 | 0.125 | 7.1807 | 15219.28 | 15848.06 | 0.0973 | 0.0986 | 3.9676 |
| 444 | 14792.26 | 15491.58 | 0.1143 | 0.116 | 4.5142 | 13795.48 | 13930.58 | 0.1052 | 0.0992 | 0.9698 |
| 445 | 12596.51 | 12660.82 | 0.1105 | 0.099 | 0.508 | 14534.74 | 15244.24 | 0.1045 | 0.0972 | 4.6542 |
| 446 | 12856.99 | 12924.77 | 0.1104 | 0.111 | 0.5244 | 14336.11 | 14303.98 | 0.1012 | 0.1012 | -0.225 |
| 447 | 13173.61 | 13144.7 | 0.1249 | 0.122 | -0.2199 | 14634.45 | 15472.73 | 0.0945 | 0.0962 | 5.4178 |
| 448 | 13695.36 | 13895.77 | 0.1213 | 0.124 | 1.4423 | 14105.92 | 14326.55 | 0.0956 | 0.0951 | 1.54 |
| 449 | 16152.74 | 15791.58 | 0.1283 | 0.113 | -2.2871 | 15113.71 | 15742.5 | 0.0937 | 0.0951 | 3.9942 |
| 450 | 14158.28 | 14403.72 | 0.1212 | 0.118 | 1.704 | 15563.39 | 15903.77 | 0.1059 | 0.1028 | 2.1403 |
| 451 | 12017.79 | 12123.24 | 0.1311 | 0.128 | 0.8697 | 16617.22 | 15227.77 | 0.1035 | 0.0821 | -9.124 |
| 452 | 14976.9 | 15215.06 | 0.1229 | 0.12 | 1.5653 | 15525.28 | 16339.42 | 0.0945 | 0.0951 | 4.9827 |
| 453 | 12593.57 | 12665.7 | 0.1209 | 0.122 | 0.5695 | 13594.89 | 14094.6 | 0.1137 | 0.1077 | 3.5454 |
| 454 | 16277.85 | 16812.24 | 0.1255 | 0.111 | 3.1786 | 14164.98 | 14111.55 | 0.104 | 0.1034 | -0.379 |
| 455 | 12469.58 | 12492 | 0.1231 | 0.123 | 0.1795 | 16768.55 | 17427.88 | 0.1049 | 0.0953 | 3.7832 |
| 456 | 11653.09 | 11650.16 | 0.125 | 0.125 | -0.0251 | 14577.27 | 14899.36 | 0.1101 | 0.1094 | 2.1618 |
| 457 | 14513.23 | 14719.92 | 0.1181 | 0.121 | 1.4042 | 15235.41 | 16112.81 | 0.0927 | 0.0905 | 5.4454 |
| 458 | 11901.27 | 12184.11 | 0.1294 | 0.132 | 2.3214 | 13390 | 14425.18 | 0.1106 | 0.0971 | 7.1762 |
| 459 | 12363.28 | 12310.64 | 0.1138 | 0.115 | -0.4276 | 15153.12 | 15833.33 | 0.0979 | 0.0977 | 4.2961 |
| 460 | 11666.52 | 12089.49 | 0.1265 | 0.127 | 3.4986 | 16143.57 | 16754.31 | 0.1042 | 0.1013 | 3.6453 |
| 461 | 12224.82 | 12495.24 | 0.128 | 0.131 | 2.1642 | 16611.97 | 16552.89 | 0.0978 | 0.1012 | -0.357 |
| 462 | 12751.81 | 12786.46 | 0.1143 | 0.115 | 0.2709 | 14952.39 | 14804.48 | 0.0991 | 0.099 | -0.999 |
| 463 | 14207.28 | 14519.53 | 0.1141 | 0.116 | 2.1506 | 13728.09 | 13813.44 | 0.11 | 0.1099 | 0.6179 |
| 464 | 12216.56 | 12354.97 | 0.1189 | 0.118 | 1.1202 | 13324.47 | 13255.69 | 0.0995 | 0.0985 | -0.519 |
| 465 | 16446.13 | 18233.34 | 0.1159 | 0.121 | 9.8019 | 13669.39 | 13871.61 | 0.1093 | 0.1109 | 1.4578 |
| 466 | 15439.74 | 17227.36 | 0.1136 | 0.119 | 10.377 | 15127.92 | 15756.7 | 0.0966 | 0.0979 | 3.9906 |
| 467 | 12784.03 | 12052.96 | 0.1259 | 0.107 | -6.0655 | 12754.29 | 12934.5 | 0.1109 | 0.1097 | 1.3932 |
| 468 | 13149.23 | 12720.24 | 0.1235 | 0.121 | -3.3725 | 15762.92 | 16388.77 | 0.0933 | 0.0946 | 3.8188 |
| 469 | 12080.65 | 12351.07 | 0.1298 | 0.133 | 2.1894 | 14329.16 | 14297.03 | 0.1008 | 0.1007 | -0.225 |
| 470 | 14321.63 | 15038.74 | 0.1189 | 0.12 | 4.7684 | 12849.29 | 13018.29 | 0.0909 | 0.099 | 1.2981 |
| 471 | 16356.85 | 17913.34 | 0.1136 | 0.119 | 8.689 | 12519.41 | 12696.28 | 0.1056 | 0.1054 | 1.393 |
| 472 | 14706.65 | 14435.32 | 0.1146 | 0.106 | -1.8797 | 15041.64 | 15670.93 | 0.0958 | 0.097 | 4.0156 |
| 473 | 16413.69 | 15418.37 | 0.1299 | 0.107 | -6.4554 | 13268.73 | 13489.35 | 0.0964 | 0.0959 | 1.6355 |
| 474 | 13348.19 | 13432.62 | 0.1115 | 0.111 | 0.6286 | 14133.25 | 14096.97 | 0.1011 | 0.101 | -0.257 |
| 475 | 14187.94 | 12741.8 | 0.1133 | 0.099 | -11.35 | 12787.75 | 13914.68 | 0.1038 | 0.0977 | 8.0989 |
| 476 | 13682.94 | 14136.08 | 0.1145 | 0.118 | 3.2055 | 15453.77 | 16082.56 | 0.0938 | 0.0952 | 3.9097 |
| 477 | 13832.87 | 13938.31 | 0.1237 | 0.121 | 0.7565 | 14445.64 | 14437.35 | 0.1091 | 0.1017 | -0.057 |
| 478 | 12821.3 | 13467.45 | 0.1196 | 0.115 | 4.7979 | 12885.09 | 12863.37 | 0.107 | 0.1063 | -0.169 |
| 479 | 14717.85 | 13327.07 | 0.1122 | 0.098 | -10.436 | 13653.75 | 13704.25 | 0.0954 | 0.0914 | 0.3685 |
| 480 | 12663.05 | 13628.57 | 0.1145 | 0.117 | 7.0845 | 15035.78 | 14739.84 | 0.1139 | 0.102 | -2.008 |
| 481 | 11361.95 | 12478.14 | 0.1214 | 0.12 | 8.9451 | 15165.81 | 15365.31 | 0.096 | 0.0979 | 1.2984 |
| 482 | 12776.62 | 12882.06 | 0.1278 | 0.125 | 0.8185 | 17308.66 | 17338.58 | 0.1002 | 0.1004 | 0.1725 |
| 483 | 13520.28 | 13610.07 | 0.1243 | 0.124 | 0.6597 | 15290.64 | 15919.42 | 0.0933 | 0.0947 | 3.9498 |
| 484 | 10886.66 | 11923.46 | 0.128 | 0.128 | 8.6954 | 15283.19 | 15776.7 | 0.1022 | 0.0998 | 3.1281 |
| 485 | 13421.47 | 12677 | 0.1237 | 0.118 | -5.8726 | 13972.24 | 14215.67 | 0.0951 | 0.0933 | 1.7124 |
| 486 | 12061 | 12199.4 | 0.1162 | 0.115 | 1.1345 | 14182.05 | 14253.68 | 0.101 | 0.0969 | 0.5025 |
| 487 | 11831.38 | 11874.51 | 0.1274 | 0.125 | 0.3633 | 16581.17 | 16541.56 | 0.0914 | 0.0908 | -0.24 |
| 488 | 12825.36 | 13899.15 | 0.115 | 0.117 | 7.7256 | 15883.06 | 15848.87 | 0.0963 | 0.0999 | -0.216 |
| 489 | 13003.87 | 13964.03 | 0.1189 | 0.121 | 6.8759 | 14944.96 | 13615.81 | 0.1083 | 0.0853 | -9.762 |
| 490 | 12129.19 | 12096.06 | 0.1225 | 0.125 | -0.2739 | 16370.84 | 16417.62 | 0.1025 | 0.1061 | 0.2849 |
| 491 | 12940.96 | 13030.75 | 0.1267 | 0.126 | 0.6891 | 14431.43 | 14649.42 | 0.1053 | 0.1096 | 1.488 |
| 492 | 14599.96 | 14104.87 | 0.1212 | 0.103 | -3.5101 | 14798.63 | 14848 | 0.0996 | 0.1008 | 0.3325 |
| 493 | 13119.95 | 11995.95 | 0.1215 | 0.105 | -9.3699 | 15064.25 | 15045.84 | 0.1055 | 0.1037 | -0.122 |
| 494 | 12696.12 | 12834.52 | 0.1187 | 0.118 | 1.0784 | 13852.23 | 13883.44 | 0.1036 | 0.1046 | 0.2248 |
| 495 | 13617.52 | 13685.31 | 0.1159 | 0.116 | 0.4953 | 17904.86 | 18095.03 | 0.1124 | 0.1117 | 1.051 |
| 496 | 13282.76 | 12595.65 | 0.1153 | 0.092 | -5.4551 | 17022.33 | 17131.03 | 0.1038 | 0.1076 | 0.6345 |
| 497 | 12961.14 | 13578.04 | 0.1255 | 0.128 | 4.5434 | 15089.73 | 15177.39 | 0.098 | 0.0996 | 0.5776 |
| 498 | 12212.06 | 12270.55 | 0.1216 | 0.126 | 0.4767 | 16111.92 | 14686.62 | 0.106 | 0.0841 | -9.705 |
| 499 | 12254.26 | 12909.73 | 0.1158 | 0.111 | 5.0774 | 16231.39 | 17348.79 | 0.0931 | 0.0901 | 6.4408 |
| 500 | 13849.52 | 13745.34 | 0.123 | 0.118 | -0.7579 | 12112.3 | 12406.27 | 0.102 | 0.1008 | 2.3695 |
| 501 | 14537.33 | 14605.11 | 0.115 | 0.115 | 0.4641 | 13333.64 | 14157.91 | 0.0998 | 0.0972 | 5.8219 |
| 502 | 11264.83 | 11249.48 | 0.1243 | 0.125 | -0.1365 | 12878.73 | 12873.16 | 0.1064 | 0.108 | -0.043 |
| 503 | 12002.5 | 12045.64 | 0.1295 | 0.127 | 0.3581 | 14598.6 | 14685.05 | 0.1 | 0.1014 | 0.5887 |
| 504 | 14677.5 | 13194.5 | 0.1115 | 0.096 | -11.24 | 17058.98 | 17249.77 | 0.0963 | 0.0931 | 1.1061 |
| 505 | 11833.8 | 12486.98 | 0.1231 | 0.123 | 5.2308 | 15553.3 | 15578.86 | 0.1048 | 0.1078 | 0.1641 |
| 506 | 16975.86 | 14935.83 | 0.1252 | 0.098 | -13.659 | 12968.56 | 13148.76 | 0.1123 | 0.1111 | 1.3705 |
| 507 | 11569.98 | 11637.97 | 0.1184 | 0.117 | 0.5842 | 13956.97 | 13924.84 | 0.1012 | 0.1012 | -0.231 |
| 508 | 11998.34 | 11953.45 | 0.1201 | 0.118 | -0.3755 | 16028.97 | 15106.23 | 0.1039 | 0.0938 | -6.108 |
| 509 | 14324.61 | 14459.85 | 0.108 | 0.102 | 0.9353 | 14677.01 | 16155.03 | 0.0961 | 0.0888 | 9.149 |
| 510 | 12477.75 | 12097.83 | 0.114 | 0.098 | -3.1403 | 15459.07 | 17054.67 | 0.1027 | 0.0983 | 9.3558 |
| 511 | 13058.48 | 13076.56 | 0.1148 | 0.116 | 0.1382 | 13862.88 | 13787.06 | 0.1002 | 0.1033 | -0.55 |
| 512 | 13745.34 | 13850.78 | 0.1213 | 0.119 | 0.7613 | 13502.49 | 13385.92 | 0.1066 | 0.1069 | -0.871 |
| 513 | 13263.66 | 13369.1 | 0.1276 | 0.125 | 0.7887 | 14126.81 | 14089.95 | 0.0998 | 0.0987 | -0.262 |
| 514 | 15089.43 | 14708.39 | 0.1136 | 0.107 | -2.5906 | 14375.49 | 14308.42 | 0.1041 | 0.1029 | -0.469 |
| 515 | 12101.07 | 12096.93 | 0.115 | 0.113 | -0.0342 | 15008.45 | 15161.17 | 0.099 | 0.1005 | 1.0073 |
| 516 | 13390.47 | 13929.59 | 0.1226 | 0.124 | 3.8703 | 15600.4 | 16229.19 | 0.0973 | 0.0985 | 3.8744 |
| 517 | 14802.45 | 13750.87 | 0.1219 | 0.103 | -7.6474 | 14513.42 | 14627.77 | 0.112 | 0.1102 | 0.7817 |
| 518 | 11155.43 | 11326.47 | 0.0925 | 0.091 | 1.5101 | 13136.86 | 13810.42 | 0.1042 | 0.1013 | 4.8771 |
| 519 | 15751.27 | 15533.7 | 0.1083 | 0.107 | -1.4007 | 11980.41 | 11831.12 | 0.1085 | 0.1046 | -1.262 |
| 520 | 13501.86 | 13734.88 | 0.123 | 0.12 | 1.6965 | 13561.75 | 13620.75 | 0.1066 | 0.1063 | 0.4331 |
| 521 | 11942.27 | 12080.68 | 0.1215 | 0.121 | 1.1457 | 14017.82 | 14871.96 | 0.0949 | 0.097 | 5.7433 |
| 522 | 12983.82 | 12979.68 | 0.1121 | 0.11 | -0.0319 | 16155.58 | 16417 | 0.1004 | 0.1017 | 1.5924 |
| 523 | 11994.76 | 12548.26 | 0.1304 | 0.124 | 4.411 | 11784.91 | 11772.48 | 0.1118 | 0.1111 | -0.106 |
| 524 | 13268.86 | 14088.39 | 0.1183 | 0.12 | 5.8171 | 14748.53 | 17551.24 | 0.0992 | 0.0976 | 15.969 |
| 525 | 17713.85 | 16242.96 | 0.1327 | 0.107 | -9.0555 | 13797.55 | 13680.98 | 0.1062 | 0.1064 | -0.852 |
| 526 | 13998.16 | 14555.77 | 0.1191 | 0.122 | 3.8308 | 13069.57 | 13226.23 | 0.099 | 0.1034 | 1.1844 |
| 527 | 11849.01 | 11871.44 | 0.131 | 0.131 | 0.1889 | 14215.54 | 14433.24 | 0.0981 | 0.0973 | 1.5083 |
| 528 | 12749.56 | 13517.9 | 0.1066 | 0.11 | 5.6838 | 15281.91 | 15298.27 | 0.1017 | 0.1009 | 0.1069 |
| 529 | 12516.65 | 12501.29 | 0.1251 | 0.125 | -0.1228 | 14595.6 | 14870.58 | 0.0982 | 0.0961 | 1.8491 |
| 530 | 12496.85 | 12481.5 | 0.1173 | 0.118 | -0.123 | 16256.89 | 16885.67 | 0.0947 | 0.0961 | 3.7238 |
| 531 | 12271.27 | 12407.25 | 0.1159 | 0.115 | 1.096 | 13106.08 | 13193.74 | 0.1007 | 0.1026 | 0.6644 |
| 532 | 12620.9 | 12688.68 | 0.1168 | 0.117 | 0.5342 | 14911.56 | 15595.2 | 0.0987 | 0.0983 | 4.3837 |
| 533 | 14383.54 | 13042.26 | 0.1226 | 0.105 | -10.284 | 17379.23 | 17684.46 | 0.0973 | 0.0926 | 1.726 |
| 534 | 15704.26 | 15365.94 | 0.1149 | 0.109 | -2.2018 | 17974.51 | 18752.67 | 0.1081 | 0.1045 | 4.1496 |
| 535 | 17567.52 | 15448.16 | 0.1277 | 0.105 | -13.719 | 13313.94 | 13464.56 | 0.1148 | 0.1128 | 1.1187 |
| 536 | 13212.52 | 12448.23 | 0.1209 | 0.105 | -6.1398 | 14030.72 | 14030.6 | 0.1056 | 0.1041 | -9E-04 |
| 537 | 13016.94 | 13101.37 | 0.1179 | 0.117 | 0.6445 | 12448.23 | 13011.78 | 0.0957 | 0.1009 | 4.3311 |
| 538 | 13270.93 | 13355.37 | 0.119 | 0.118 | 0.6322 | 13228.26 | 13416.96 | 0.1185 | 0.1178 | 1.4064 |
| 539 | 14614.61 | 15617.92 | 0.1263 | 0.13 | 6.4241 | 13252.99 | 13099.35 | 0.1065 | 0.1065 | -1.173 |
| 540 | 11901.59 | 12543.87 | 0.1226 | 0.12 | 5.1203 | 14638.96 | 14593.9 | 0.0998 | 0.0999 | -0.309 |
| 541 | 16043.25 | 15318.82 | 0.1249 | 0.109 | -4.729 | 15598.45 | 15989.84 | 0.1084 | 0.1066 | 2.4477 |
| 542 | 17182.95 | 16623.91 | 0.1332 | 0.112 | -3.3629 | 14948.59 | 15169.22 | 0.0961 | 0.0956 | 1.4544 |
| 543 | 18789.51 | 16218.85 | 0.1314 | 0.107 | -15.85 | 18009.39 | 17137.74 | 0.0986 | 0.0903 | -5.086 |
| 544 | 12947.81 | 13217.93 | 0.1277 | 0.13 | 2.0436 | 14638.29 | 15306.78 | 0.0958 | 0.097 | 4.3673 |
| 545 | 11822.5 | 11718.53 | 0.1255 | 0.125 | -0.8872 | 15207.92 | 15823.77 | 0.0984 | 0.0995 | 3.892 |
| 546 | 13166.4 | 13533.17 | 0.1282 | 0.127 | 2.7102 | 17645.44 | 17556.65 | 0.1019 | 0.1038 | -0.506 |
| 547 | 13181.4 | 13585.78 | 0.1219 | 0.115 | 2.9765 | 13951.75 | 14080.75 | 0.1051 | 0.1047 | 0.9161 |
| 548 | 11516.26 | 11538.68 | 0.1261 | 0.126 | 0.1944 | 13101.13 | 13109 | 0.1071 | 0.109 | 0.06 |
| 549 | 13262.18 | 13960.3 | 0.1221 | 0.125 | 5.0007 | 15851.14 | 15716.49 | 0.0974 | 0.1018 | -0.857 |
| 550 | 13580.09 | 13850.12 | 0.1211 | 0.122 | 1.9497 | 14794.51 | 17640.86 | 0.098 | 0.0942 | 16.135 |
| 551 | 12636.55 | 12610.86 | 0.1191 | 0.12 | -0.2037 | 13668.58 | 16136.73 | 0.0989 | 0.0967 | 15.295 |
| 552 | 13805.55 | 13903.33 | 0.115 | 0.115 | 0.7033 | 18386.99 | 18982.02 | 0.1113 | 0.1072 | 3.1347 |
| 553 | 11988.03 | 12029.45 | 0.1157 | 0.115 | 0.3443 | 14154.6 | 14277.52 | 0.1005 | 0.0988 | 0.861 |
| 554 | 13039.56 | 12884.92 | 0.1274 | 0.125 | -1.2002 | 14597.06 | 15103.3 | 0.1045 | 0.1034 | 3.3518 |
| 555 | 12784.82 | 13207.37 | 0.1219 | 0.123 | 3.1993 | 14239.74 | 14868.53 | 0.0964 | 0.0977 | 4.229 |
| 556 | 12794.01 | 13469.03 | 0.1278 | 0.13 | 5.0117 | 13994.95 | 13938.09 | 0.0983 | 0.0978 | -0.408 |
| 557 | 14359.1 | 14940.58 | 0.1209 | 0.119 | 3.8919 | 15708.42 | 14789.46 | 0.1077 | 0.1009 | -6.214 |
| 558 | 16921.27 | 15431.06 | 0.1333 | 0.109 | -9.6572 | 12783.87 | 12902.37 | 0.0984 | 0.1 | 0.9184 |
| 559 | 12583.86 | 12612.15 | 0.1239 | 0.126 | 0.2243 | 13590.45 | 13703.89 | 0.1042 | 0.1051 | 0.8277 |
| 560 | 16571.47 | 16027.78 | 0.1346 | 0.113 | -3.3922 | 14938.98 | 14874.04 | 0.1089 | 0.0994 | -0.437 |
| 561 | 12787.34 | 12949.89 | 0.1275 | 0.11 | 1.2552 | 13988.62 | 14292.97 | 0.0905 | 0.1013 | 2.1294 |
| 562 | 11794.01 | 12378.69 | 0.1303 | 0.131 | 4.7233 | 14669.22 | 14975.96 | 0.1066 | 0.1032 | 2.0482 |
| 563 | 13706.33 | 13863.31 | 0.1218 | 0.124 | 1.1324 | 17304.53 | 15888.72 | 0.1042 | 0.084 | -8.911 |
| 564 | 13277.49 | 13508.11 | 0.1074 | 0.108 | 1.7073 | 12853.08 | 13014.29 | 0.104 | 0.1037 | 1.2387 |
| 565 | 12149.1 | 12886.66 | 0.1198 | 0.121 | 5.7234 | 12952.19 | 13131.9 | 0.113 | 0.1102 | 1.3685 |
| 566 | 10892.03 | 11451.65 | 0.1318 | 0.126 | 4.8868 | 14804.22 | 15159.4 | 0.109 | 0.1051 | 2.343 |
| 567 | 16877.87 | 16226.27 | 0.1314 | 0.11 | -4.0157 | 13356.04 | 13558.26 | 0.1086 | 0.1103 | 1.4915 |
| 568 | 12044.61 | 12306.07 | 0.0922 | 0.095 | 2.1246 | 17253.89 | 17430.55 | 0.0966 | 0.0934 | 1.0135 |
| 569 | 12574.08 | 13693.24 | 0.1189 | 0.116 | 8.173 | 14398.12 | 14644.07 | 0.1049 | 0.1014 | 1.6795 |
| 570 | 11346.43 | 12207.54 | 0.1287 | 0.129 | 7.0539 | 16145.32 | 16656.53 | 0.1049 | 0.1024 | 3.0691 |
| 571 | 13770 | 14024.27 | 0.1131 | 0.114 | 1.813 | 13017.16 | 13105.44 | 0.1056 | 0.1076 | 0.6736 |
| 572 | 12449.98 | 12539.77 | 0.1239 | 0.123 | 0.7161 | 13868.7 | 13815.27 | 0.1047 | 0.1041 | -0.387 |
| 573 | 12823.15 | 12912.94 | 0.1256 | 0.125 | 0.6954 | 13330.87 | 13826.4 | 0.1076 | 0.1063 | 3.5839 |
| 574 | 13464.03 | 14617.71 | 0.1201 | 0.117 | 7.8923 | 12576.03 | 12660.09 | 0.1156 | 0.1134 | 0.6639 |
| 575 | 13106.63 | 13144.41 | 0.1206 | 0.12 | 0.2874 | 15514.19 | 16198.54 | 0.0992 | 0.0969 | 4.2248 |
| 576 | 14152 | 13793.38 | 0.1248 | 0.108 | -2.5999 | 14398.9 | 14483.63 | 0.1006 | 0.1024 | 0.585 |
| 577 | 17880.94 | 17131.9 | 0.1339 | 0.113 | -4.3722 | 13292.97 | 13433.17 | 0.1097 | 0.1077 | 1.0437 |
| 578 | 11745.95 | 11794.94 | 0.1151 | 0.113 | 0.4154 | 17580 | 17748.87 | 0.0934 | 0.1009 | 0.9515 |
| 579 | 11926.18 | 12552.87 | 0.1189 | 0.105 | 4.9924 | 18291.65 | 18240.44 | 0.0933 | 0.0974 | -0.281 |
| 580 | 12923.76 | 12991.55 | 0.1132 | 0.114 | 0.5217 | 15814.46 | 14420.87 | 0.1039 | 0.0816 | -9.664 |
| 581 | 12671.41 | 13004.38 | 0.1279 | 0.13 | 2.5604 | 12869 | 12876.99 | 0.1082 | 0.1086 | 0.062 |
| 582 | 14346.53 | 14466.62 | 0.1143 | 0.115 | 0.8301 | 12627.51 | 12861.86 | 0.0988 | 0.0973 | 1.8221 |
| 583 | 18457.51 | 19625.95 | 0.1356 | 0.132 | 5.9536 | 16522.12 | 16626.97 | 0.1005 | 0.1007 | 0.6306 |
| 584 | 12622.49 | 13494.5 | 0.1294 | 0.132 | 6.462 | 15743.06 | 16368.92 | 0.0934 | 0.0947 | 3.8235 |
| 585 | 14643.58 | 16254.55 | 0.1145 | 0.12 | 9.9109 | 12047.86 | 12282.42 | 0.0936 | 0.1022 | 1.9097 |
| 586 | 12732.33 | 12504.97 | 0.1214 | 0.117 | -1.8182 | 14316.47 | 14423.63 | 0.0994 | 0.1011 | 0.7429 |
| 587 | 11168.03 | 11202.38 | 0.1233 | 0.121 | 0.3066 | 13689.24 | 13355.48 | 0.1091 | 0.1075 | -2.499 |
| 588 | 13798.43 | 14052.7 | 0.122 | 0.122 | 1.8094 | 12965.86 | 12721.01 | 0.1048 | 0.1023 | -1.925 |
| 589 | 14837.67 | 14934.24 | 0.1273 | 0.117 | 0.6466 | 15105.46 | 15417.89 | 0.0957 | 0.0959 | 2.0264 |
| 590 | 17012.72 | 17312.93 | 0.1195 | 0.118 | 1.734 | 15586.76 | 16212.62 | 0.0954 | 0.0967 | 3.8603 |
| 591 | 12137.48 | 13156.21 | 0.1175 | 0.105 | 7.7434 | 13292.28 | 13530.06 | 0.103 | 0.1012 | 1.7574 |
| 592 | 13661.63 | 13964.77 | 0.1083 | 0.097 | 2.1707 | 16194.75 | 16405.26 | 0.0937 | 0.0924 | 1.2831 |
| 593 | 16699.31 | 16302.4 | 0.1349 | 0.113 | -2.4347 | 12966.21 | 12920.57 | 0.1081 | 0.1065 | -0.353 |
| 594 | 11797.44 | 11840.58 | 0.1312 | 0.129 | 0.3643 | 13122.61 | 13183.11 | 0.1085 | 0.1037 | 0.4589 |
| 595 | 13530.11 | 13635.55 | 0.1276 | 0.125 | 0.7733 | 13254.65 | 13212.72 | 0.1074 | 0.105 | -0.317 |
| 596 | 14053.38 | 15351.84 | 0.1022 | 0.115 | 8.458 | 14170.27 | 14137.13 | 0.1039 | 0.1045 | -0.234 |
| 597 | 16191.03 | 16196.05 | 0.1274 | 0.113 | 0.031 | 15643.12 | 16271.91 | 0.0952 | 0.0966 | 3.8642 |
| 598 | 12493.13 | 12565.26 | 0.117 | 0.119 | 0.5741 | 14913.93 | 14881.8 | 0.101 | 0.101 | -0.216 |
| 599 | 11701.9 | 12428.67 | 0.1223 | 0.122 | 5.8475 | 14460.04 | 14653.68 | 0.101 | 0.1003 | 1.3214 |
| 600 | 14363.65 | 15482.6 | 0.1197 | 0.105 | 7.2271 | 15201.17 | 15263.9 | 0.0979 | 0.097 | 0.4109 |
| 601 | 12521.15 | 12799.85 | 0.1214 | 0.124 | 2.1774 | 16335.17 | 16093.37 | 0.0971 | 0.0993 | -1.502 |
| 602 | 13140.95 | 12109.58 | 0.1192 | 0.111 | -8.517 | 17638.72 | 17577.01 | 0.1039 | 0.1065 | -0.351 |
| 603 | 11086.44 | 11365.14 | 0.1296 | 0.132 | 2.4522 | 13738.97 | 14367.76 | 0.0985 | 0.0999 | 4.3764 |
| 604 | 13806.29 | 13926.37 | 0.1183 | 0.119 | 0.8623 | 13317.17 | 13452.02 | 0.1047 | 0.102 | 1.0025 |
| 605 | 14262.51 | 14609 | 0.1201 | 0.121 | 2.3717 | 13516.34 | 14183.91 | 0.1038 | 0.1021 | 4.7066 |
| 606 | 12412.18 | 12849.59 | 0.1284 | 0.133 | 3.404 | 15958.02 | 16292.37 | 0.0929 | 0.091 | 2.0522 |
| 607 | 12153.52 | 12608.82 | 0.1277 | 0.123 | 3.611 | 14205.58 | 14264.15 | 0.102 | 0.1028 | 0.4107 |
| 608 | 16774.4 | 16941.89 | 0.1216 | 0.121 | 0.9886 | 14232.26 | 15676.64 | 0.0995 | 0.0965 | 9.2136 |
| 609 | 13872.06 | 14205.66 | 0.1192 | 0.119 | 2.3484 | 14463.9 | 14548.63 | 0.1012 | 0.1029 | 0.5824 |
| 610 | 12988.46 | 12615.62 | 0.1115 | 0.096 | -2.9554 | 15759.58 | 14331.38 | 0.1076 | 0.0938 | -9.966 |
| 611 | 17140.24 | 15179.08 | 0.1273 | 0.104 | -12.92 | 14475.37 | 14463.86 | 0.0992 | 0.0982 | -0.08 |
| 612 | 12787.9 | 12746.47 | 0.1199 | 0.122 | -0.325 | 15281.75 | 15490.62 | 0.1035 | 0.1073 | 1.3484 |
| 613 | 13538.33 | 13622.77 | 0.1193 | 0.119 | 0.6198 | 14485.58 | 14634.07 | 0.1055 | 0.1046 | 1.0147 |
| 614 | 12620.7 | 12695.35 | 0.1193 | 0.12 | 0.588 | 16790.38 | 17425.02 | 0.0935 | 0.0946 | 3.6421 |
| 615 | 12542.39 | 13647.91 | 0.1292 | 0.116 | 8.1002 | 15933.39 | 16562.68 | 0.0942 | 0.0953 | 3.7994 |
| 616 | 12876.41 | 12948.54 | 0.1201 | 0.122 | 0.5571 | 14850.99 | 15077.44 | 0.1034 | 0.1009 | 1.5019 |
| 617 | 12649.78 | 12721.92 | 0.1248 | 0.126 | 0.567 | 13434.22 | 13552.59 | 0.1019 | 0.1036 | 0.8734 |
| 618 | 12991.19 | 13827.43 | 0.13 | 0.133 | 6.0477 | 15931.66 | 16108.32 | 0.0969 | 0.0936 | 1.0967 |
| 619 | 14001.53 | 13350.96 | 0.1223 | 0.104 | -4.8729 | 16708.69 | 16782.19 | 0.104 | 0.0955 | 0.438 |
| 620 | 12293.78 | 14182.74 | 0.1175 | 0.124 | 13.319 | 15421.53 | 14054.3 | 0.104 | 0.0822 | -9.728 |
| 621 | 11677.56 | 11771.92 | 0.126 | 0.125 | 0.8015 | 14743.03 | 16106.29 | 0.0964 | 0.0953 | 8.4641 |
| 622 | 12427.63 | 13211.01 | 0.1213 | 0.103 | 5.9298 | 13868.77 | 14055.72 | 0.1075 | 0.1042 | 1.3301 |
| 623 | 15630.06 | 15408.52 | 0.1167 | 0.109 | -1.4378 | 12325.59 | 12957.93 | 0.0953 | 0.1012 | 4.8799 |
| 624 | 17697.57 | 16281.04 | 0.1348 | 0.109 | -8.7005 | 13831.37 | 14154.55 | 0.0971 | 0.1037 | 2.2832 |
| 625 | 12412.77 | 12914.65 | 0.1121 | 0.105 | 3.8861 | 14178.83 | 14452.65 | 0.1096 | 0.1064 | 1.8945 |
| 626 | 13893.69 | 14421.89 | 0.1142 | 0.114 | 3.6625 | 15879.79 | 14492.09 | 0.1064 | 0.0941 | -9.576 |
| 627 | 12320.54 | 12338.62 | 0.117 | 0.118 | 0.1465 | 15629.26 | 15831.27 | 0.1052 | 0.1018 | 1.276 |
| 628 | 12046.23 | 11526.82 | 0.1259 | 0.122 | -4.5061 | 12510.18 | 14059.18 | 0.105 | 0.1089 | 11.018 |
| 629 | 12752.72 | 13319.59 | 0.1165 | 0.117 | 4.2559 | 17548.78 | 16615.68 | 0.1011 | 0.0947 | -5.616 |
| 630 | 17036.12 | 15095.17 | 0.1302 | 0.108 | -12.858 | 13121.86 | 14389.6 | 0.1056 | 0.0977 | 8.8101 |
| 631 | 11402.36 | 12178.75 | 0.1192 | 0.113 | 6.375 | 14408.27 | 14523.92 | 0.1047 | 0.1032 | 0.7963 |
| 632 | 13684.95 | 12919.68 | 0.1135 | 0.114 | -5.9233 | 13476.53 | 14142.1 | 0.097 | 0.0985 | 4.7063 |
| 633 | 12788.26 | 13457.38 | 0.1169 | 0.115 | 4.9721 | 13402.52 | 13474.74 | 0.1041 | 0.1046 | 0.536 |
| 634 | 11158.39 | 11473.57 | 0.1255 | 0.126 | 2.747 | 13339.41 | 13965.27 | 0.0978 | 0.0992 | 4.4815 |
| 635 | 11911.28 | 13114.23 | 0.1278 | 0.123 | 9.1729 | 13486.9 | 13227.49 | 0.1076 | 0.109 | -1.961 |
| 636 | 14023.48 | 15044.71 | 0.1084 | 0.106 | 6.788 | 17771.7 | 16986.74 | 0.1011 | 0.0922 | -4.621 |
| 637 | 11851.67 | 11923.8 | 0.1239 | 0.125 | 0.6049 | 17034.71 | 17131.07 | 0.0889 | 0.0876 | 0.5625 |
| 638 | 16622.36 | 15534.88 | 0.1233 | 0.102 | -7.0003 | 15482.13 | 15657.78 | 0.1009 | 0.0967 | 1.1218 |
| 639 | 14788.86 | 14523.76 | 0.1288 | 0.113 | -1.8253 | 15630.4 | 14396.68 | 0.1022 | 0.0976 | -8.57 |
| 640 | 13284.56 | 13573.09 | 0.1074 | 0.102 | 2.1257 | 13580.43 | 13990.51 | 0.1067 | 0.1036 | 2.9312 |
| 641 | 12271.52 | 12367.6 | 0.1266 | 0.125 | 0.7769 | 13768.85 | 16409.42 | 0.0945 | 0.0942 | 16.092 |
| 642 | 11534.78 | 11813.48 | 0.1269 | 0.13 | 2.3592 | 13037.07 | 13229.5 | 0.1059 | 0.1061 | 1.4545 |
| 643 | 14834.14 | 15289.12 | 0.1173 | 0.119 | 2.9758 | 18068.16 | 18055.94 | 0.0959 | 0.0997 | -0.068 |
| 644 | 12942.54 | 13047.98 | 0.1272 | 0.125 | 0.8081 | 14310.6 | 14939.39 | 0.0943 | 0.0957 | 4.2089 |
| 645 | 12834.35 | 12976.07 | 0.1274 | 0.113 | 1.0921 | 15789.75 | 15778.25 | 0.0969 | 0.096 | -0.073 |
| 646 | 12169.24 | 12219.45 | 0.1234 | 0.122 | 0.4109 | 14571.07 | 15294.62 | 0.0944 | 0.097 | 4.7308 |
| 647 | 16760.73 | 17277.01 | 0.1208 | 0.119 | 2.9882 | 16554.99 | 16495.91 | 0.0973 | 0.1007 | -0.358 |
| 648 | 12809.57 | 13287.7 | 0.1269 | 0.125 | 3.5983 | 14966.24 | 15073.39 | 0.0992 | 0.1008 | 0.7109 |
| 649 | 18336.86 | 16684.97 | 0.1349 | 0.11 | -9.9005 | 14621.23 | 14801.64 | 0.0995 | 0.0983 | 1.2189 |
| 650 | 12393.31 | 12476.15 | 0.1123 | 0.111 | 0.664 | 12637.6 | 12702.45 | 0.1124 | 0.1119 | 0.5106 |
| 651 | 14556.63 | 14814.79 | 0.1246 | 0.122 | 1.7426 | 13506.13 | 13389.56 | 0.1033 | 0.1037 | -0.871 |
| 652 | 12667.88 | 12735.66 | 0.1159 | 0.116 | 0.5322 | 13245.39 | 13330.75 | 0.1126 | 0.1124 | 0.6403 |
| 653 | 12321.23 | 11737.68 | 0.1257 | 0.121 | -4.9716 | 15972.72 | 14628.92 | 0.1052 | 0.0844 | -9.186 |
| 654 | 11027.32 | 11316.44 | 0.1273 | 0.127 | 2.5548 | 13411.93 | 13722.34 | 0.1032 | 0.1004 | 2.2621 |
| 655 | 11462.53 | 11447.17 | 0.122 | 0.122 | -0.1341 | 12750.38 | 12701.09 | 0.1076 | 0.1072 | -0.388 |
| 656 | 12767.11 | 13828.44 | 0.1231 | 0.112 | 7.675 | 17053 | 16537 | 0.1042 | 0.093 | -3.12 |
| 657 | 14176.34 | 14992.94 | 0.1217 | 0.125 | 5.4466 | 16070.88 | 16754.02 | 0.096 | 0.096 | 4.0775 |
| 658 | 11097.22 | 12141.6 | 0.1223 | 0.125 | 8.6016 | 15309.12 | 15296.69 | 0.0911 | 0.0904 | -0.081 |
| 659 | 12592.42 | 14044.71 | 0.1182 | 0.108 | 10.34 | 12776.44 | 13033.84 | 0.0978 | 0.0972 | 1.9749 |
| 660 | 14058.16 | 14680.54 | 0.1315 | 0.127 | 4.2395 | 16327.06 | 16330.49 | 0.0984 | 0.1043 | 0.021 |
| 661 | 11348.68 | 11939.1 | 0.1201 | 0.124 | 4.9452 | 16798.33 | 16849.55 | 0.0961 | 0.1016 | 0.3039 |
| 662 | 12122.86 | 13719.41 | 0.1221 | 0.11 | 11.637 | 17146.55 | 17323.21 | 0.097 | 0.0938 | 1.0198 |
| 663 | 11980.1 | 12064.53 | 0.1225 | 0.122 | 0.6999 | 13858.11 | 13603.26 | 0.1033 | 0.101 | -1.873 |
| 664 | 13039.83 | 13090.71 | 0.121 | 0.122 | 0.3887 | 15205.22 | 16075.01 | 0.0956 | 0.0972 | 5.4108 |
| 665 | 13387.21 | 13684.2 | 0.1077 | 0.095 | 2.1704 | 12597.31 | 13005 | 0.1121 | 0.1065 | 3.1349 |
| 666 | 13962.76 | 14599.95 | 0.1239 | 0.127 | 4.3644 | 12949.98 | 12960.18 | 0.1066 | 0.103 | 0.0788 |
| 667 | 12427.75 | 12807.37 | 0.1248 | 0.127 | 2.9641 | 14843.39 | 14984.9 | 0.0997 | 0.1011 | 0.9443 |
| 668 | 14093.77 | 14161.56 | 0.1151 | 0.116 | 0.4786 | 13132.4 | 13306.55 | 0.1063 | 0.1038 | 1.3087 |
| 669 | 12415.18 | 12916.4 | 0.1246 | 0.111 | 3.8804 | 15275.55 | 15553.45 | 0.1075 | 0.1056 | 1.7868 |
| 670 | 13406.91 | 13524.07 | 0.1198 | 0.121 | 0.8663 | 13169.27 | 13402.4 | 0.1146 | 0.1128 | 1.7395 |
| 671 | 13296.29 | 13175.58 | 0.1254 | 0.11 | -0.9162 | 13730.77 | 14390.27 | 0.0963 | 0.0973 | 4.5829 |
| 672 | 12963.63 | 13387.39 | 0.1253 | 0.127 | 3.1654 | 12873.16 | 12996.21 | 0.1079 | 0.1056 | 0.9468 |
| 673 | 11635.4 | 12372.96 | 0.1211 | 0.122 | 5.9611 | 16888.25 | 16811.38 | 0.0997 | 0.1022 | -0.457 |
| 674 | 11573.37 | 11868.1 | 0.1293 | 0.132 | 2.4834 | 13808.57 | 13932.17 | 0.1063 | 0.1059 | 0.8872 |
| 675 | 12555.77 | 12467.19 | 0.1307 | 0.111 | -0.7105 | 17064.25 | 17037.89 | 0.0979 | 0.1014 | -0.155 |
| 676 | 12791.2 | 14632.02 | 0.1168 | 0.108 | 12.581 | 13644.18 | 13792.67 | 0.1086 | 0.1076 | 1.0766 |
| 677 | 10685.39 | 11497.21 | 0.13 | 0.127 | 7.061 | 13740.81 | 14006.67 | 0.1096 | 0.1115 | 1.8981 |
| 678 | 12682.23 | 12802.32 | 0.1197 | 0.121 | 0.938 | 11865.32 | 12347.16 | 0.097 | 0.1038 | 3.9024 |
| 679 | 13069.49 | 13273.25 | 0.1234 | 0.127 | 1.5351 | 13278.21 | 15940.63 | 0.0965 | 0.0951 | 16.702 |
| 680 | 13094.99 | 13199.42 | 0.1157 | 0.115 | 0.7912 | 16923.25 | 16914.25 | 0.0969 | 0.1024 | -0.053 |
| 681 | 14556.03 | 15365.61 | 0.1229 | 0.127 | 5.2688 | 13241.39 | 13940.59 | 0.1011 | 0.1019 | 5.0156 |
| 682 | 13930.21 | 14035.65 | 0.116 | 0.114 | 0.7512 | 14275.25 | 14550.22 | 0.0985 | 0.0964 | 1.8898 |
| 683 | 15434.07 | 16166.41 | 0.129 | 0.118 | 4.53 | 14989.55 | 15030.22 | 0.1024 | 0.1023 | 0.2706 |
| 684 | 13295.11 | 12859.76 | 0.1216 | 0.118 | -3.3854 | 16210.49 | 16322.71 | 0.1006 | 0.1027 | 0.6875 |
| 685 | 13011.01 | 13605.77 | 0.113 | 0.107 | 4.3714 | 17326.86 | 17728.45 | 0.0986 | 0.0951 | 2.2652 |
| 686 | 10771.7 | 10910.1 | 0.1238 | 0.123 | 1.2686 | 15104.92 | 15776.34 | 0.0962 | 0.0974 | 4.2559 |
| 687 | 11851.29 | 11885.64 | 0.1233 | 0.121 | 0.289 | 15307.47 | 15987.68 | 0.0973 | 0.0972 | 4.2546 |
| 688 | 12160.72 | 12266.16 | 0.1305 | 0.128 | 0.8596 | 14785.31 | 15795.72 | 0.0983 | 0.097 | 6.3968 |
| 689 | 19030.72 | 16219.18 | 0.1311 | 0.105 | -17.335 | 15952.08 | 16128.73 | 0.1008 | 0.0974 | 1.0953 |
| 690 | 13237.44 | 13255.51 | 0.1164 | 0.118 | 0.1364 | 14437.68 | 14645.64 | 0.1094 | 0.1051 | 1.4199 |
| 691 | 17506.1 | 14941.47 | 0.1255 | 0.103 | -17.165 | 16520.97 | 16522.25 | 0.1059 | 0.0908 | 0.0078 |
| 692 | 13024.28 | 12255.92 | 0.124 | 0.104 | -6.2693 | 16853.9 | 16930.38 | 0.0996 | 0.1014 | 0.4517 |
| 693 | 12320.08 | 12458.48 | 0.1209 | 0.12 | 1.1109 | 15233.6 | 15557.74 | 0.0959 | 0.0961 | 2.0835 |
| 694 | 13061.21 | 13063.23 | 0.1078 | 0.107 | 0.0154 | 16854.35 | 17537.48 | 0.0962 | 0.0962 | 3.8953 |
| 695 | 11743.9 | 11876 | 0.122 | 0.12 | 1.1123 | 18120.44 | 18138.01 | 0.1014 | 0.1039 | 0.0969 |
| 696 | 13961.14 | 14778.8 | 0.1231 | 0.127 | 5.5327 | 16596.62 | 16620.76 | 0.1006 | 0.1047 | 0.1453 |
| 697 | 12563.35 | 12862.72 | 0.1252 | 0.12 | 2.3275 | 12684.91 | 13361.68 | 0.0996 | 0.1006 | 5.0651 |
| 698 | 14090.57 | 14734.03 | 0.1183 | 0.119 | 4.3672 | 15594.69 | 15422.3 | 0.116 | 0.1056 | -1.118 |
| 699 | 13291.12 | 12685.35 | 0.1208 | 0.115 | -4.7754 | 16804.24 | 16321.49 | 0.1018 | 0.0915 | -2.958 |
| 700 | 11822.65 | 12139.05 | 0.1259 | 0.128 | 2.6064 | 15167.71 | 15621.43 | 0.0986 | 0.0957 | 2.9045 |
| 701 | 16839.08 | 14502.49 | 0.1232 | 0.097 | -16.112 | 14568.52 | 14536.38 | 0.1032 | 0.1032 | -0.221 |
| 702 | 11029.71 | 11075.28 | 0.1236 | 0.123 | 0.4114 | 14060.92 | 14252.05 | 0.0989 | 0.0976 | 1.341 |
| 703 | 11950.04 | 12623.54 | 0.125 | 0.124 | 5.3353 | 14262.32 | 14403.24 | 0.1032 | 0.1032 | 0.9784 |
| 704 | 11527.15 | 11665.55 | 0.1228 | 0.122 | 1.1865 | 16382.56 | 16824.69 | 0.1027 | 0.1 | 2.6279 |
| 705 | 15555.44 | 15390.59 | 0.1083 | 0.108 | -1.0711 | 14118.53 | 14690.07 | 0.0998 | 0.1045 | 3.8907 |
| 706 | 12259.14 | 12634.92 | 0.1169 | 0.103 | 2.9741 | 16263.98 | 16128.12 | 0.1028 | 0.1055 | -0.842 |
| 707 | 11442.97 | 11427.62 | 0.1291 | 0.129 | -0.1344 | 15711.24 | 14323.5 | 0.103 | 0.0806 | -9.688 |
| 708 | 12094.89 | 13770.15 | 0.1218 | 0.11 | 12.166 | 15724.85 | 15692.72 | 0.0971 | 0.0971 | -0.205 |
| 709 | 11734.87 | 11989.13 | 0.1185 | 0.119 | 2.1208 | 17414.73 | 17328.37 | 0.0983 | 0.1016 | -0.498 |
| 710 | 11377.7 | 12012.63 | 0.1241 | 0.121 | 5.2855 | 16504.7 | 16556 | 0.0968 | 0.0971 | 0.3099 |
| 711 | 14529.67 | 13491.73 | 0.1169 | 0.1 | -7.6932 | 16220.78 | 16235.13 | 0.0936 | 0.0909 | 0.0884 |
| 712 | 12721.7 | 12827.14 | 0.1305 | 0.128 | 0.822 | 13482.51 | 14449.62 | 0.1018 | 0.099 | 6.693 |
| 713 | 12760.81 | 13458.56 | 0.1285 | 0.131 | 5.1844 | 14531.56 | 15055.37 | 0.1064 | 0.1056 | 3.4792 |
| 714 | 14015.35 | 14207.66 | 0.1222 | 0.12 | 1.3535 | 16955.53 | 16896.44 | 0.098 | 0.1013 | -0.35 |
| 715 | 13754.69 | 14037.13 | 0.1098 | 0.101 | 2.0121 | 17548.01 | 17469.73 | 0.1021 | 0.1044 | -0.448 |
| 716 | 13913.16 | 14018.6 | 0.1258 | 0.124 | 0.7522 | 13836.2 | 13975.78 | 0.1023 | 0.1044 | 0.9988 |
| 717 | 11804.72 | 12126.93 | 0.1093 | 0.105 | 2.6569 | 15616.64 | 15326.17 | 0.0941 | 0.0957 | -1.895 |
| 718 | 13564.48 | 13632.27 | 0.1172 | 0.118 | 0.4972 | 12720.71 | 13013.64 | 0.0981 | 0.1093 | 2.2509 |
| 719 | 13220.6 | 13977.55 | 0.1268 | 0.129 | 5.4155 | 13032.94 | 13152.82 | 0.1006 | 0.1043 | 0.9114 |
| 720 | 12136.21 | 12219.06 | 0.1161 | 0.114 | 0.678 | 15519.67 | 15570.97 | 0.0986 | 0.0989 | 0.3295 |
| 721 | 11942.03 | 13697.14 | 0.1213 | 0.127 | 12.814 | 16725.7 | 16691.26 | 0.1069 | 0.1097 | -0.206 |
| 722 | 16106.66 | 16465.74 | 0.1172 | 0.112 | 2.1808 | 14995.58 | 15092.85 | 0.1035 | 0.0811 | 0.6445 |
| 723 | 13495.97 | 13601.41 | 0.1279 | 0.126 | 0.7752 | 17072.6 | 17049.46 | 0.1007 | 0.1055 | -0.136 |
| 724 | 13451.16 | 13732.64 | 0.1284 | 0.126 | 2.0497 | 15655.91 | 15967.24 | 0.1075 | 0.1062 | 1.9498 |
| 725 | 18050.01 | 15519.73 | 0.1268 | 0.104 | -16.304 | 17907.47 | 17259.75 | 0.1051 | 0.0938 | -3.753 |
| 726 | 13303.61 | 13375.74 | 0.1218 | 0.123 | 0.5393 | 13514.09 | 13521.46 | 0.1012 | 0.1024 | 0.0545 |
| 727 | 12361.85 | 12467.29 | 0.1289 | 0.126 | 0.8457 | 14931.31 | 15560.1 | 0.0948 | 0.0961 | 4.041 |
| 728 | 12755.41 | 12988.25 | 0.1198 | 0.119 | 1.7927 | 14317.79 | 14243.24 | 0.1083 | 0.111 | -0.523 |
| 729 | 13621.68 | 13727.12 | 0.1268 | 0.124 | 0.7681 | 18128.83 | 17455.97 | 0.1059 | 0.0963 | -3.855 |
| 730 | 12757.31 | 11762.5 | 0.1087 | 0.098 | -8.4574 | 16040.54 | 16965.42 | 0.1056 | 0.0928 | 5.4516 |
| 731 | 11628.15 | 11776.06 | 0.0969 | 0.101 | 1.256 | 13153.41 | 13238.76 | 0.1108 | 0.1107 | 0.6447 |
| 732 | 11863.72 | 12052.21 | 0.1232 | 0.12 | 1.564 | 15034.13 | 15029.48 | 0.11 | 0.0993 | -0.031 |
| 733 | 13564.92 | 14323.95 | 0.1236 | 0.129 | 5.299 | 16352.83 | 16412.83 | 0.0944 | 0.0927 | 0.3656 |
| 734 | 11629.7 | 12766.68 | 0.1239 | 0.121 | 8.9059 | 15885.05 | 16370.53 | 0.1046 | 0.0817 | 2.9656 |
| 735 | 14892.19 | 15157.46 | 0.1307 | 0.115 | 1.7501 | 13830.16 | 13826.23 | 0.1037 | 0.1017 | -0.028 |
| 736 | 16496.89 | 15982.66 | 0.1228 | 0.103 | -3.2174 | 12764.17 | 12776.6 | 0.1102 | 0.1093 | 0.0973 |
| 737 | 17444.94 | 15822.55 | 0.1362 | 0.112 | -10.254 | 16102.35 | 16030.84 | 0.0977 | 0.1013 | -0.446 |
| 738 | 13452.66 | 13690.82 | 0.1234 | 0.121 | 1.7396 | 14784.34 | 14582.41 | 0.1115 | 0.1023 | -1.385 |
| 739 | 13055.53 | 13051.47 | 0.1242 | 0.124 | -0.0311 | 14393.53 | 14587.17 | 0.103 | 0.1023 | 1.3275 |
| 740 | 16576.02 | 17817.6 | 0.1245 | 0.113 | 6.9683 | 14714.72 | 15611.29 | 0.0966 | 0.0977 | 5.7431 |
| 741 | 16466.92 | 14279.19 | 0.134 | 0.107 | -15.321 | 12594.85 | 12794.85 | 0.1076 | 0.1098 | 1.5631 |
| 742 | 12066.88 | 12272.65 | 0.1257 | 0.128 | 1.6767 | 15069.97 | 15225.92 | 0.1051 | 0.1018 | 1.0242 |
| 743 | 11484.64 | 11476.44 | 0.1252 | 0.125 | -0.0714 | 17079.31 | 17086.89 | 0.102 | 0.1033 | 0.0443 |
| 744 | 11474.23 | 12588.73 | 0.1319 | 0.125 | 8.8531 | 13933.48 | 14018.21 | 0.1016 | 0.1033 | 0.6044 |
| 745 | 16128.69 | 16419.19 | 0.1258 | 0.115 | 1.7693 | 14105.18 | 14380.16 | 0.0976 | 0.0955 | 1.9122 |
| 746 | 13485.58 | 12556.93 | 0.1248 | 0.105 | -7.3955 | 13463.91 | 13443.11 | 0.0985 | 0.0983 | -0.155 |
| 747 | 15143.38 | 15842.71 | 0.1142 | 0.116 | 4.4142 | 14439.63 | 14761.73 | 0.1105 | 0.1098 | 2.182 |
| 748 | 11545.99 | 11587.41 | 0.1195 | 0.118 | 0.3575 | 13209.79 | 14027.78 | 0.109 | 0.103 | 5.8312 |
| 749 | 17045.18 | 14990.59 | 0.1351 | 0.108 | -13.706 | 16950.66 | 17446.64 | 0.1045 | 0.0937 | 2.8428 |
| 750 | 12395.81 | 13134.56 | 0.1305 | 0.132 | 5.6245 | 13841.64 | 14197.38 | 0.1071 | 0.1059 | 2.5056 |
| 751 | 12682.42 | 12994.67 | 0.1214 | 0.123 | 2.4029 | 12905.3 | 13060.86 | 0.1071 | 0.1098 | 1.1911 |
| 752 | 12010.1 | 12068.09 | 0.1156 | 0.115 | 0.4805 | 14553.54 | 14660.7 | 0.101 | 0.1026 | 0.7309 |
| 753 | 12205.1 | 13158.85 | 0.1158 | 0.118 | 7.2479 | 13017.84 | 13284.62 | 0.1168 | 0.1157 | 2.0082 |
| 754 | 17251.82 | 16946.12 | 0.1257 | 0.108 | -1.804 | 15285.53 | 15655.91 | 0.1043 | 0.1019 | 2.3658 |
| 755 | 13430.45 | 14382.26 | 0.129 | 0.131 | 6.6179 | 13648.05 | 13531.48 | 0.1058 | 0.1061 | -0.861 |
| 756 | 14069.25 | 14265.58 | 0.1195 | 0.118 | 1.3762 | 13481.81 | 14076.16 | 0.1121 | 0.1092 | 4.2224 |
| 757 | 16583.15 | 18055.21 | 0.1149 | 0.12 | 8.1531 | 17577.15 | 17442.51 | 0.0959 | 0.0998 | -0.772 |
| 758 | 16418.45 | 16718.66 | 0.1206 | 0.119 | 1.7956 | 15403.23 | 15594.03 | 0.0995 | 0.096 | 1.2235 |
| 759 | 16291.38 | 15613.52 | 0.1291 | 0.111 | -4.3415 | 13547.26 | 13632.61 | 0.1106 | 0.1105 | 0.6261 |
| 760 | 12418.44 | 12395.53 | 0.1191 | 0.117 | -0.1849 | 15446.43 | 16221.79 | 0.0975 | 0.0976 | 4.7797 |
| 761 | 13355 | 13861.02 | 0.1101 | 0.104 | 3.6506 | 14144.18 | 14770.04 | 0.0957 | 0.0971 | 4.2373 |
| 762 | 11884.13 | 12206.34 | 0.1088 | 0.105 | 2.6396 | 13735.98 | 13554.27 | 0.1029 | 0.0997 | -1.341 |
| 763 | 14595.6 | 15534.3 | 0.1295 | 0.118 | 6.0428 | 13319.17 | 13965.74 | 0.0981 | 0.099 | 4.6297 |
| 764 | 17059.43 | 15682.07 | 0.1298 | 0.107 | -8.7831 | 16187.47 | 17201.15 | 0.1033 | 0.1013 | 5.8931 |
| 765 | 11164.93 | 12424.46 | 0.1209 | 0.109 | 10.138 | 13618.64 | 13839.27 | 0.0947 | 0.0942 | 1.5942 |
| 766 | 14055.86 | 14344.98 | 0.113 | 0.113 | 2.0155 | 14891.94 | 15203.27 | 0.1087 | 0.1074 | 2.0478 |
| 767 | 15417.69 | 16509.42 | 0.1111 | 0.115 | 6.6128 | 13760.3 | 13687.37 | 0.098 | 0.098 | -0.533 |
| 768 | 11918.6 | 12594.82 | 0.1221 | 0.105 | 5.3691 | 15170.78 | 15458.69 | 0.1055 | 0.1043 | 1.8624 |
| 769 | 13637.36 | 13870.38 | 0.1217 | 0.119 | 1.6799 | 18167.01 | 18071.36 | 0.0963 | 0.0997 | -0.529 |
| 770 | 13523.83 | 13716.35 | 0.1164 | 0.116 | 1.4035 | 17179.98 | 17356.64 | 0.0971 | 0.0939 | 1.0178 |
| 771 | 12811.62 | 12944.67 | 0.1116 | 0.111 | 1.0278 | 16806.01 | 17354.97 | 0.1115 | 0.1073 | 3.1631 |
| 772 | 12467.04 | 12789.24 | 0.1079 | 0.104 | 2.5193 | 17095.36 | 16442.62 | 0.1022 | 0.0928 | -3.97 |
| 773 | 12952.65 | 12970.72 | 0.1155 | 0.117 | 0.1394 | 12626.03 | 12657.25 | 0.111 | 0.1097 | 0.2466 |
| 774 | 11474.54 | 13422.13 | 0.1242 | 0.112 | 14.51 | 16828.81 | 16813.96 | 0.0958 | 0.1014 | -0.088 |
| 775 | 12271.08 | 12573.08 | 0.1098 | 0.106 | 2.4019 | 13791.34 | 15480.58 | 0.1034 | 0.0988 | 10.912 |
| 776 | 11005.72 | 11881.39 | 0.1262 | 0.126 | 7.3701 | 12548.81 | 12636.3 | 0.1036 | 0.1026 | 0.6923 |
| 777 | 12284.84 | 12844.46 | 0.1239 | 0.12 | 4.3569 | 14480.37 | 15163.5 | 0.099 | 0.0988 | 4.5051 |
| 778 | 12773.65 | 12883.37 | 0.124 | 0.122 | 0.8516 | 14992.29 | 17187.18 | 0.1017 | 0.0999 | 12.77 |
| 779 | 16558.42 | 15800.38 | 0.126 | 0.115 | -4.7976 | 14584.57 | 14669.93 | 0.1062 | 0.1061 | 0.5818 |
| 780 | 11833.36 | 12021.85 | 0.1243 | 0.121 | 1.5679 | 15188.53 | 15155.39 | 0.0985 | 0.0991 | -0.219 |
| 781 | 14947.57 | 13794.07 | 0.1206 | 0.102 | -8.3623 | 14257.64 | 16529.42 | 0.0985 | 0.0972 | 13.744 |
| 782 | 16487.12 | 17566.86 | 0.1243 | 0.114 | 6.1465 | 15122.3 | 15153.31 | 0.0976 | 0.0969 | 0.2046 |
| 783 | 15585.86 | 15653.64 | 0.1126 | 0.113 | 0.433 | 13989.87 | 14062.08 | 0.1055 | 0.106 | 0.5136 |
| 784 | 18368.28 | 17563.21 | 0.1308 | 0.11 | -4.5839 | 14902.47 | 15168.71 | 0.1063 | 0.1044 | 1.7552 |
| 785 | 14739.1 | 14456.56 | 0.1088 | 0.106 | -1.9545 | 13047.87 | 13028.87 | 0.1012 | 0.105 | -0.146 |
| 786 | 13861.56 | 13929.34 | 0.1145 | 0.115 | 0.4866 | 12739.47 | 12848.76 | 0.1093 | 0.1092 | 0.8506 |
| 787 | 12436.94 | 12421.59 | 0.1191 | 0.119 | -0.1236 | 14112.29 | 14116.73 | 0.1029 | 0.1041 | 0.0314 |
| 788 | 18339.2 | 16505.72 | 0.1337 | 0.109 | -11.108 | 14818.57 | 15096.05 | 0.1042 | 0.1046 | 1.8381 |
| 789 | 11098.27 | 11120.69 | 0.1282 | 0.128 | 0.2017 | 13470.3 | 13535.15 | 0.1115 | 0.111 | 0.4791 |
| 790 | 13700.11 | 13791.03 | 0.1228 | 0.125 | 0.6593 | 13483.59 | 13726.43 | 0.1034 | 0.1099 | 1.7692 |
| 791 | 12625.51 | 12709.95 | 0.1221 | 0.121 | 0.6643 | 14390.51 | 14369.63 | 0.1095 | 0.1018 | -0.145 |
| 792 | 13368.77 | 13708.41 | 0.1245 | 0.126 | 2.4776 | 14381.3 | 14496.44 | 0.0954 | 0.0936 | 0.7943 |
| 793 | 17608.39 | 18850.68 | 0.1355 | 0.131 | 6.5902 | 13181.25 | 13298.82 | 0.1075 | 0.1075 | 0.8841 |
| 794 | 13192.82 | 13875.92 | 0.1296 | 0.132 | 4.9229 | 14879.46 | 14942.18 | 0.0982 | 0.0974 | 0.4198 |
| 795 | 17033.37 | 18224.91 | 0.1328 | 0.128 | 6.538 | 14894.2 | 15013.99 | 0.0951 | 0.0931 | 0.7979 |
| 796 | 12993.71 | 13078.15 | 0.1221 | 0.121 | 0.6456 | 13649.42 | 13749.42 | 0.0989 | 0.1005 | 0.7273 |
| 797 | 11647.03 | 12216.47 | 0.1232 | 0.124 | 4.6613 | 12852.81 | 13361.93 | 0.1071 | 0.1014 | 3.8102 |
| 798 | 13675.91 | 13970.57 | 0.1067 | 0.1 | 2.1091 | 13205.83 | 13058.56 | 0.1076 | 0.1078 | -1.128 |
| 799 | 11581.9 | 11703.74 | 0.1224 | 0.123 | 1.041 | 13518.89 | 13591.1 | 0.1047 | 0.1052 | 0.5314 |
| 800 | 13744.69 | 14599.37 | 0.1164 | 0.118 | 5.8542 | 17455.59 | 17440.73 | 0.0934 | 0.0988 | -0.085 |
| 801 | 15136.93 | 16355.87 | 0.1283 | 0.119 | 7.4526 | 13798.53 | 14033.59 | 0.116 | 0.1138 | 1.675 |
| 802 | 12396.06 | 12285.73 | 0.1299 | 0.127 | -0.898 | 15806.64 | 16101.49 | 0.0989 | 0.1056 | 1.8312 |
| 803 | 13838.6 | 14263.25 | 0.1111 | 0.114 | 2.9772 | 13489.13 | 15607.28 | 0.0949 | 0.0954 | 13.572 |
| 804 | 11164.6 | 12513.65 | 0.124 | 0.126 | 10.781 | 14916.32 | 14967.62 | 0.0999 | 0.1003 | 0.3427 |
| 805 | 13289.51 | 12791.48 | 0.1265 | 0.108 | -3.8934 | 15478 | 15836.37 | 0.0956 | 0.0924 | 2.263 |
| 806 | 12318.95 | 12303.6 | 0.1237 | 0.124 | -0.1248 | 14568.59 | 14330.81 | 0.1003 | 0.0981 | -1.659 |
| 807 | 12628.52 | 12666.3 | 0.1221 | 0.121 | 0.2983 | 13350.71 | 13385.86 | 0.1075 | 0.1076 | 0.2626 |
| 808 | 16177.5 | 16468.28 | 0.1301 | 0.108 | 1.7657 | 12818.52 | 13241.78 | 0.1047 | 0.1015 | 3.1964 |
| 809 | 13898.03 | 14858.27 | 0.1218 | 0.111 | 6.4627 | 15383.25 | 15498.9 | 0.1005 | 0.0992 | 0.7462 |
| 810 | 12241.4 | 12331.19 | 0.1174 | 0.117 | 0.7282 | 15061.11 | 14976.35 | 0.0986 | 0.0979 | -0.566 |
| 811 | 13874.19 | 14092.98 | 0.1174 | 0.117 | 1.5525 | 16057.08 | 16233.73 | 0.0978 | 0.0944 | 1.0882 |
| 812 | 15830.66 | 16080.24 | 0.1191 | 0.117 | 1.5521 | 13576.28 | 15219.15 | 0.102 | 0.0987 | 10.795 |
| 813 | 14197.41 | 14880.51 | 0.1306 | 0.133 | 4.5906 | 18157.64 | 18826.1 | 0.1094 | 0.1059 | 3.5507 |
| 814 | 13036.95 | 13363.3 | 0.1034 | 0.1 | 2.4421 | 14039.41 | 14936.9 | 0.099 | 0.0987 | 6.0085 |
| 815 | 11725.96 | 11706.46 | 0.1153 | 0.117 | -0.1666 | 13042.51 | 14521.96 | 0.1075 | 0.0986 | 10.188 |
| 816 | 12853.67 | 12992.07 | 0.1166 | 0.116 | 1.0653 | 18293.72 | 18242.51 | 0.0939 | 0.098 | -0.281 |
| 817 | 12337.28 | 13020.38 | 0.1219 | 0.125 | 5.2464 | 12600.24 | 12443.67 | 0.1058 | 0.1026 | -1.258 |
| 818 | 12744.47 | 12812.26 | 0.1162 | 0.117 | 0.529 | 13368.65 | 13475.81 | 0.1017 | 0.1034 | 0.7952 |
| 819 | 10638.06 | 11249.51 | 0.125 | 0.122 | 5.4354 | 14728.35 | 14655.42 | 0.0951 | 0.0951 | -0.498 |
| 820 | 13193.44 | 13617.72 | 0.1128 | 0.1 | 3.1156 | 15444.79 | 15311.74 | 0.1005 | 0.1025 | -0.869 |
| 821 | 12283.16 | 13380.86 | 0.1236 | 0.113 | 8.2035 | 13768.5 | 14094.74 | 0.1118 | 0.1077 | 2.3146 |
| 822 | 12565.54 | 12633.32 | 0.1185 | 0.119 | 0.5365 | 12779.27 | 12864.62 | 0.1094 | 0.1093 | 0.6635 |
| 823 | 18005.87 | 15395.29 | 0.1231 | 0.104 | -16.957 | 15170.97 | 13789.09 | 0.105 | 0.0821 | -10.02 |
| 824 | 13321.05 | 13245.99 | 0.1154 | 0.114 | -0.5667 | 14186.48 | 16295.13 | 0.0941 | 0.0945 | 12.94 |
| 825 | 17905.65 | 17172.88 | 0.1312 | 0.11 | -4.267 | 13085.03 | 12903.81 | 0.1003 | 0.0974 | -1.404 |
| 826 | 13173.86 | 13191.94 | 0.1153 | 0.117 | 0.137 | 15247.47 | 15258.69 | 0.1093 | 0.1023 | 0.0735 |
| 827 | 13451.12 | 13570.32 | 0.1234 | 0.125 | 0.8784 | 13380.28 | 13411.29 | 0.1016 | 0.1008 | 0.2312 |
| 828 | 13173.59 | 11963.61 | 0.1199 | 0.106 | -10.114 | 16959.55 | 17305.32 | 0.1035 | 0.1059 | 1.9981 |
| 829 | 14363.91 | 14602.07 | 0.1212 | 0.118 | 1.631 | 13649.06 | 13813.7 | 0.1037 | 0.1041 | 1.1919 |
| 830 | 13368.38 | 13277.97 | 0.1189 | 0.118 | -0.6809 | 14554.63 | 14982.21 | 0.1121 | 0.1109 | 2.8539 |
| 831 | 13129.03 | 13785.77 | 0.1293 | 0.131 | 4.7639 | 17535.94 | 17428.58 | 0.097 | 0.101 | -0.616 |
| 832 | 13784.52 | 13375.03 | 0.1188 | 0.116 | -3.0617 | 15740.28 | 16315.34 | 0.0964 | 0.1043 | 3.5247 |
| 833 | 17582.74 | 18759.47 | 0.1366 | 0.132 | 6.2727 | 12915.36 | 13000.92 | 0.1059 | 0.1044 | 0.6581 |
| 834 | 15989.13 | 16163.69 | 0.1248 | 0.124 | 1.0799 | 13660.93 | 13801.23 | 0.1012 | 0.103 | 1.0165 |
| 835 | 13277.11 | 13272.97 | 0.1119 | 0.11 | -0.0312 | 15112.67 | 16052.21 | 0.0977 | 0.0911 | 5.853 |
| 836 | 11291.97 | 11446.94 | 0.1239 | 0.123 | 1.3539 | 12046.52 | 12284.43 | 0.0999 | 0.0988 | 1.9366 |
| 837 | 11978.27 | 13038.2 | 0.1334 | 0.129 | 8.1295 | 13007.19 | 13092.55 | 0.1097 | 0.1096 | 0.6519 |
| 838 | 13500.04 | 14335.95 | 0.1042 | 0.109 | 5.8309 | 14138.24 | 13975.22 | 0.1104 | 0.1011 | -1.166 |
| 839 | 12084.26 | 12764.76 | 0.1221 | 0.118 | 5.3311 | 15326.91 | 14949.8 | 0.1006 | 0.0975 | -2.522 |
| 840 | 12754.45 | 12069.73 | 0.1281 | 0.109 | -5.673 | 14516.34 | 16478.42 | 0.0928 | 0.0934 | 11.907 |
| 841 | 15693.12 | 15941.7 | 0.1272 | 0.115 | 1.5593 | 14119.16 | 14238.45 | 0.0941 | 0.0921 | 0.8378 |
| 842 | 16133.42 | 16926.17 | 0.1265 | 0.114 | 4.6836 | 15356.32 | 13989.09 | 0.1058 | 0.0841 | -9.774 |
| 843 | 14829.23 | 14919.02 | 0.1203 | 0.12 | 0.6019 | 14212.59 | 14157.44 | 0.1104 | 0.1036 | -0.39 |
| 844 | 14863.39 | 15546.49 | 0.1181 | 0.121 | 4.3939 | 15210.7 | 15488.6 | 0.1061 | 0.1042 | 1.7942 |
| 845 | 15049.09 | 15335.07 | 0.1312 | 0.116 | 1.8649 | 15314.35 | 15429.49 | 0.0928 | 0.091 | 0.7463 |
| 846 | 11660.22 | 11798.63 | 0.1194 | 0.119 | 1.1731 | 13954.24 | 14229.22 | 0.0997 | 0.0976 | 1.9325 |
| 847 | 12417.56 | 12985.71 | 0.1176 | 0.115 | 4.3752 | 14893.27 | 14774.07 | 0.1032 | 0.1046 | -0.807 |
| 848 | 16963.37 | 16619.69 | 0.1238 | 0.108 | -2.0679 | 13042.84 | 13748.62 | 0.1157 | 0.106 | 5.1334 |
| 849 | 14044.61 | 14181.17 | 0.1155 | 0.116 | 0.963 | 12190.26 | 12476.53 | 0.1039 | 0.1059 | 2.2945 |
| 850 | 14171.11 | 14342.84 | 0.1051 | 0.096 | 1.1973 | 17409.41 | 16798.4 | 0.108 | 0.0988 | -3.637 |
| 851 | 12898.07 | 13013.34 | 0.1241 | 0.126 | 0.8858 | 13000.63 | 13259.12 | 0.1176 | 0.1167 | 1.9495 |
| 852 | 13950.16 | 14719.53 | 0.127 | 0.131 | 5.2269 | 16746.53 | 17117.92 | 0.0992 | 0.0961 | 2.1696 |
| 853 | 16128.77 | 16296.26 | 0.1247 | 0.124 | 1.0278 | 14179.07 | 14592.88 | 0.1096 | 0.1075 | 2.8357 |
| 854 | 16254.96 | 15420.94 | 0.1329 | 0.11 | -5.4084 | 13280.72 | 13672.11 | 0.1088 | 0.1066 | 2.8627 |
| 855 | 14680.29 | 14819.88 | 0.1146 | 0.116 | 0.9419 | 12871.4 | 12996.42 | 0.1123 | 0.11 | 0.962 |
| 856 | 11346.92 | 11010.52 | 0.119 | 0.101 | -3.0552 | 13832.51 | 14479.08 | 0.0975 | 0.0983 | 4.4655 |
| 857 | 12163.8 | 12148.45 | 0.1246 | 0.125 | -0.1264 | 15552.54 | 15862.92 | 0.1011 | 0.0969 | 1.9566 |
| 858 | 12824.79 | 12775.38 | 0.1084 | 0.108 | -0.3868 | 16091.79 | 16090.78 | 0.1063 | 0.1083 | -0.006 |
| 859 | 11830.52 | 11968.93 | 0.1173 | 0.117 | 1.1564 | 16951.62 | 16847.47 | 0.1088 | 0.1082 | -0.618 |
| 860 | 12638.63 | 12777.04 | 0.1199 | 0.119 | 1.0832 | 14501.7 | 14389.28 | 0.1011 | 0.1004 | -0.781 |
| 861 | 13083.84 | 13068.48 | 0.1174 | 0.118 | -0.1175 | 18347.93 | 18253.49 | 0.0952 | 0.0987 | -0.517 |
| 862 | 11766.05 | 11807.47 | 0.1203 | 0.119 | 0.3508 | 13766.4 | 13666.52 | 0.1186 | 0.1096 | -0.731 |
| 863 | 12499.91 | 13112.45 | 0.1131 | 0.108 | 4.6715 | 13747.17 | 13819.39 | 0.1027 | 0.1032 | 0.5226 |
| 864 | 13415.11 | 13504.9 | 0.1161 | 0.116 | 0.6649 | 13503.09 | 13514.6 | 0.0979 | 0.0975 | 0.0851 |
| 865 | 12300.62 | 12194.22 | 0.1242 | 0.123 | -0.8725 | 15168.37 | 15317.66 | 0.1051 | 0.1045 | 0.9746 |
| 866 | 12263.66 | 12286.08 | 0.1221 | 0.122 | 0.1825 | 13664.03 | 13930.81 | 0.1158 | 0.1148 | 1.915 |
| 867 | 13590.7 | 13378.86 | 0.1225 | 0.107 | -1.5834 | 15267.59 | 15267.29 | 0.1025 | 0.1022 | -0.002 |
| 868 | 12067.54 | 12393.61 | 0.1216 | 0.119 | 2.6309 | 13864.18 | 14050.96 | 0.0939 | 0.0953 | 1.3293 |
| 869 | 16904.18 | 15014.02 | 0.1272 | 0.105 | -12.589 | 14451.14 | 14455.58 | 0.1026 | 0.1038 | 0.0307 |
| 870 | 11692.53 | 12056.08 | 0.1203 | 0.113 | 3.0155 | 15761.68 | 15475.94 | 0.1097 | 0.101 | -1.846 |
| 871 | 12510.25 | 12578.03 | 0.1172 | 0.118 | 0.5389 | 13142.5 | 13173.5 | 0.1027 | 0.1018 | 0.2354 |
| 872 | 15569.9 | 13529.08 | 0.1257 | 0.101 | -15.085 | 14610.75 | 16711.62 | 0.0948 | 0.0956 | 12.571 |
| 873 | 15781.6 | 15662.9 | 0.1174 | 0.121 | -0.7578 | 18200.06 | 17892.48 | 0.0977 | 0.1024 | -1.719 |
| 874 | 11544.39 | 11616.52 | 0.1224 | 0.124 | 0.6209 | 13569.77 | 14254.41 | 0.1041 | 0.1023 | 4.803 |
| 875 | 12467.01 | 11639.19 | 0.1153 | 0.1 | -7.1123 | 14522.36 | 14641.65 | 0.0957 | 0.0938 | 0.8147 |
| 876 | 12536.2 | 12645.91 | 0.1205 | 0.123 | 0.8675 | 17891.46 | 17362.74 | 0.1025 | 0.0928 | -3.045 |
| 877 | 12417.4 | 12485.18 | 0.1159 | 0.116 | 0.5429 | 12804.24 | 13003.03 | 0.1091 | 0.1107 | 1.5288 |
| 878 | 12473.04 | 12468.99 | 0.1275 | 0.127 | -0.0325 | 14306.46 | 14285.66 | 0.0998 | 0.0997 | -0.146 |
| 879 | 11692.1 | 11774.94 | 0.1146 | 0.113 | 0.7036 | 15210.84 | 15373.57 | 0.1027 | 0.1017 | 1.0584 |
| 880 | 12867.32 | 13298.49 | 0.1287 | 0.127 | 3.2423 | 12462.75 | 12623.37 | 0.1068 | 0.1058 | 1.2724 |
| 881 | 15479.59 | 14808.05 | 0.1157 | 0.113 | -4.535 | 15482.74 | 15866.56 | 0.1053 | 0.1028 | 2.419 |
| 882 | 12604.58 | 12667.71 | 0.121 | 0.121 | 0.4984 | 14353.79 | 15052.99 | 0.0979 | 0.0988 | 4.6449 |
| 883 | 15013.16 | 15080.94 | 0.1156 | 0.116 | 0.4495 | 14335.51 | 14270.58 | 0.1098 | 0.1038 | -0.455 |
| 884 | 14160.11 | 14244.55 | 0.1162 | 0.116 | 0.5928 | 13491.9 | 13766.96 | 0.1135 | 0.1086 | 1.998 |
| 885 | 16257.14 | 15921.12 | 0.113 | 0.115 | -2.1105 | 14062.39 | 14150.05 | 0.099 | 0.1007 | 0.6195 |
| 886 | 12093.12 | 12385.37 | 0.1259 | 0.126 | 2.3597 | 16782.28 | 15438.48 | 0.1014 | 0.0814 | -8.704 |
| 887 | 12629.39 | 12719.18 | 0.1233 | 0.123 | 0.706 | 13032.86 | 13309.05 | 0.0962 | 0.0957 | 2.0752 |
| 888 | 12921.52 | 12120.36 | 0.123 | 0.105 | -6.6101 | 12383.04 | 12704.88 | 0.1076 | 0.1045 | 2.5332 |
| 889 | 13190.77 | 13205 | 0.1074 | 0.106 | 0.1077 | 13322.4 | 13275.04 | 0.1163 | 0.1062 | -0.357 |
| 890 | 12963.27 | 13047.71 | 0.1204 | 0.12 | 0.6471 | 14337.27 | 14701.49 | 0.1148 | 0.1117 | 2.4775 |
| 891 | 14448.41 | 15429.03 | 0.1281 | 0.118 | 6.3557 | 14139.3 | 14363.95 | 0.1084 | 0.1099 | 1.5639 |
| 892 | 11656.73 | 11801.71 | 0.0955 | 0.099 | 1.2284 | 13338.73 | 13361.45 | 0.1043 | 0.1064 | 0.17 |
| 893 | 12850.51 | 13226.42 | 0.1171 | 0.113 | 2.8421 | 13454.98 | 13679.62 | 0.1083 | 0.1098 | 1.6422 |
| 894 | 12655.38 | 13219.11 | 0.1192 | 0.115 | 4.2645 | 14271.94 | 14538.71 | 0.1135 | 0.1125 | 1.8349 |
| 895 | 12381.5 | 14055.7 | 0.1215 | 0.127 | 11.911 | 12991.84 | 13202.84 | 0.1093 | 0.1112 | 1.5982 |
| 896 | 13102.75 | 13425.21 | 0.1239 | 0.125 | 2.4019 | 15232.44 | 15861.23 | 0.0929 | 0.0944 | 3.9643 |
| 897 | 15171.52 | 15450.22 | 0.1122 | 0.115 | 1.8039 | 14748.52 | 14842.03 | 0.0989 | 0.1008 | 0.6301 |
| 898 | 13468.83 | 13558.63 | 0.1193 | 0.119 | 0.6622 | 16146.47 | 16548.06 | 0.1003 | 0.0964 | 2.4268 |
| 899 | 13290.22 | 13973.32 | 0.1289 | 0.131 | 4.8886 | 14925.32 | 15335.4 | 0.1055 | 0.1027 | 2.6741 |
| 900 | 13580.53 | 13685.97 | 0.1277 | 0.125 | 0.7704 | 15213.05 | 15325.27 | 0.0942 | 0.0924 | 0.7322 |
| 901 | 11988.8 | 11935.67 | 0.1211 | 0.12 | -0.4452 | 13649.22 | 13924.24 | 0.1078 | 0.1061 | 1.9752 |
| 902 | 13458.7 | 15204.89 | 0.1061 | 0.117 | 11.484 | 16037.39 | 16666.17 | 0.0959 | 0.0972 | 3.7728 |
| 903 | 14414.53 | 14827.79 | 0.1231 | 0.127 | 2.7871 | 14579.85 | 15242.49 | 0.0971 | 0.0984 | 4.3473 |
| 904 | 12859.19 | 12843.84 | 0.1197 | 0.12 | -0.1196 | 16063.95 | 16514.87 | 0.1017 | 0.0994 | 2.7304 |
| 905 | 12099.07 | 12454.76 | 0.1279 | 0.13 | 2.8558 | 15113.7 | 15329.85 | 0.1093 | 0.1056 | 1.41 |
| 906 | 13242.23 | 14143.15 | 0.12 | 0.108 | 6.37 | 14779.84 | 15212.44 | 0.1096 | 0.1086 | 2.8437 |
| 907 | 12342.81 | 12901.75 | 0.11 | 0.103 | 4.3323 | 16653.16 | 17002.33 | 0.0973 | 0.0948 | 2.0536 |
| 908 | 12706.39 | 12960.65 | 0.1208 | 0.121 | 1.9618 | 13258.7 | 13125.77 | 0.1045 | 0.1047 | -1.013 |
| 909 | 12334.22 | 12397.36 | 0.1205 | 0.121 | 0.5093 | 13735.48 | 13596.28 | 0.1024 | 0.1021 | -1.024 |
| 910 | 12685.08 | 13288.67 | 0.1199 | 0.112 | 4.5421 | 14846.02 | 15471.88 | 0.0949 | 0.0963 | 4.0451 |
| 911 | 12307.38 | 12397.17 | 0.1165 | 0.116 | 0.7243 | 14340.43 | 14559.01 | 0.104 | 0.1043 | 1.5013 |
| 912 | 13200.88 | 13662.18 | 0.1246 | 0.122 | 3.3765 | 13763.77 | 14438.04 | 0.1077 | 0.0983 | 4.6701 |
| 913 | 13281.21 | 13973.26 | 0.1112 | 0.107 | 4.9526 | 15050.73 | 15056.97 | 0.1003 | 0.1018 | 0.0414 |
| 914 | 12137.06 | 12574.17 | 0.1318 | 0.133 | 3.4762 | 12523.96 | 12627.6 | 0.1026 | 0.1063 | 0.8207 |
| 915 | 12913.36 | 13003.15 | 0.1149 | 0.115 | 0.6905 | 18460.9 | 17879.75 | 0.1047 | 0.0941 | -3.25 |
| 916 | 12171.4 | 12261.19 | 0.1234 | 0.123 | 0.7323 | 13477.32 | 13688.32 | 0.1066 | 0.1085 | 1.5415 |
| 917 | 11325.16 | 12987.14 | 0.1233 | 0.126 | 12.797 | 17744.29 | 17609.64 | 0.0959 | 0.0998 | -0.765 |
| 918 | 12922.21 | 13027.65 | 0.13 | 0.128 | 0.8094 | 12653.31 | 12776.45 | 0.0997 | 0.1013 | 0.9638 |
| 919 | 14293.39 | 14417.62 | 0.1162 | 0.118 | 0.8616 | 13730.31 | 13815.66 | 0.1095 | 0.1094 | 0.6178 |
| 920 | 11812.8 | 11835.22 | 0.1225 | 0.123 | 0.1895 | 15837.05 | 15828.76 | 0.0954 | 0.095 | -0.052 |
| 921 | 15353.59 | 16294.01 | 0.1255 | 0.116 | 5.7715 | 12749.52 | 12772.25 | 0.1059 | 0.1081 | 0.1779 |
| 922 | 13096.6 | 13418.8 | 0.1034 | 0.1 | 2.4011 | 13632.02 | 13768.26 | 0.1065 | 0.1046 | 0.9895 |
| 923 | 12196.73 | 12554.26 | 0.1211 | 0.121 | 2.8478 | 14520.93 | 15204.07 | 0.0995 | 0.0993 | 4.4931 |
| 924 | 12747.56 | 12853 | 0.1221 | 0.12 | 0.8204 | 14412.52 | 15096.46 | 0.1016 | 0.1005 | 4.5304 |
| 925 | 14936.03 | 14726.53 | 0.1126 | 0.111 | -1.4226 | 15093.33 | 15776.47 | 0.0971 | 0.097 | 4.3301 |
| 926 | 12955.69 | 13075.77 | 0.121 | 0.122 | 0.9184 | 16722.43 | 16899.09 | 0.0964 | 0.0932 | 1.0454 |
| 927 | 11190.07 | 11824.99 | 0.1246 | 0.121 | 5.3693 | 14711.82 | 14763.12 | 0.0998 | 0.1002 | 0.3475 |
| 928 | 17297.48 | 15865.27 | 0.128 | 0.106 | -9.0274 | 15328.36 | 15797.27 | 0.1017 | 0.1012 | 2.9683 |
| 929 | 12455.63 | 13531.01 | 0.1245 | 0.119 | 7.9475 | 15168.96 | 15133.39 | 0.1016 | 0.0989 | -0.235 |
| 930 | 12413.63 | 13792.18 | 0.1136 | 0.117 | 9.9951 | 16736.36 | 17419.49 | 0.095 | 0.095 | 3.9217 |
| 931 | 11670.52 | 12230.14 | 0.1283 | 0.123 | 4.5757 | 14287.65 | 14685.01 | 0.1016 | 0.0993 | 2.7059 |
| 932 | 13871.9 | 13741.69 | 0.1169 | 0.117 | -0.9475 | 16495.29 | 15101.7 | 0.1035 | 0.0822 | -9.228 |
| 933 | 16159.8 | 17392.74 | 0.113 | 0.116 | 7.0888 | 15573.58 | 15541.44 | 0.0993 | 0.0992 | -0.207 |
| 934 | 12786.48 | 13824.72 | 0.1269 | 0.113 | 7.51 | 15732.56 | 15914.78 | 0.0948 | 0.0927 | 1.145 |
| 935 | 12140.17 | 12595.48 | 0.1289 | 0.124 | 3.6148 | 16735.57 | 16824.86 | 0.0889 | 0.0875 | 0.5307 |
| 936 | 13085.2 | 13472.31 | 0.1201 | 0.12 | 2.8734 | 12314.79 | 12495 | 0.1119 | 0.1107 | 1.4422 |
| 937 | 14442.28 | 14831.1 | 0.1173 | 0.119 | 2.6217 | 17454.24 | 18164.11 | 0.1099 | 0.1063 | 3.9081 |
| 938 | 11928.13 | 13640.04 | 0.1174 | 0.109 | 12.551 | 13073.76 | 13159.12 | 0.1081 | 0.108 | 0.6486 |
| 939 | 16415.46 | 16685.88 | 0.1199 | 0.118 | 1.6206 | 14913.91 | 16016.96 | 0.097 | 0.0966 | 6.8868 |
| 940 | 11388.09 | 11410.52 | 0.1242 | 0.124 | 0.1965 | 13085.83 | 13234.33 | 0.1088 | 0.1078 | 1.122 |
| 941 | 15290.72 | 15458.21 | 0.1246 | 0.124 | 1.0835 | 14739.39 | 14572.94 | 0.1037 | 0.0958 | -1.142 |
| 942 | 16770.08 | 16043.13 | 0.1324 | 0.11 | -4.5312 | 15342.06 | 15971.35 | 0.0952 | 0.0964 | 3.9401 |
| 943 | 12129.16 | 13478.21 | 0.1252 | 0.127 | 10.009 | 14197.97 | 14463.82 | 0.1084 | 0.1103 | 1.8381 |
| 944 | 12702.16 | 12785 | 0.114 | 0.112 | 0.648 | 14093.3 | 14241.79 | 0.1069 | 0.1059 | 1.0427 |
| 945 | 13276.81 | 13105.27 | 0.1263 | 0.123 | -1.309 | 13926.1 | 13901.96 | 0.0996 | 0.0983 | -0.174 |
| 946 | 12883.78 | 12378.51 | 0.1225 | 0.118 | -4.0818 | 13206.01 | 13168.73 | 0.1049 | 0.107 | -0.283 |
| 947 | 12570.12 | 13690.45 | 0.1228 | 0.122 | 8.1833 | 16663.45 | 16989.19 | 0.0981 | 0.0944 | 1.9173 |
| 948 | 13623.19 | 13712.98 | 0.1222 | 0.122 | 0.6548 | 16611.47 | 16552.39 | 0.0987 | 0.102 | -0.357 |
| 949 | 12260.81 | 12306.38 | 0.1201 | 0.119 | 0.3702 | 14826.3 | 14794.16 | 0.102 | 0.102 | -0.217 |
| 950 | 17793.49 | 18093.7 | 0.1179 | 0.117 | 1.6592 | 15963.04 | 16174.34 | 0.1013 | 0.0998 | 1.3064 |
| 951 | 12248.46 | 12738.79 | 0.1188 | 0.105 | 3.8491 | 13587.46 | 13709.6 | 0.1049 | 0.1036 | 0.8909 |
| 952 | 12493.38 | 12580.75 | 0.1233 | 0.123 | 0.6944 | 12297.45 | 12709.29 | 0.0998 | 0.0995 | 3.2404 |
| 953 | 12730.81 | 12216.93 | 0.1269 | 0.108 | -4.2063 | 13947.99 | 13780 | 0.1013 | 0.101 | -1.219 |
| 954 | 13039 | 13014.23 | 0.1205 | 0.121 | -0.1903 | 12512.93 | 12531.51 | 0.1033 | 0.1052 | 0.1483 |
| 955 | 12679.05 | 13678.41 | 0.1186 | 0.104 | 7.3061 | 16169.58 | 16041.51 | 0.11 | 0.102 | -0.798 |
| 956 | 17875.39 | 15438.37 | 0.1336 | 0.107 | -15.785 | 13982.76 | 14611.55 | 0.0959 | 0.0973 | 4.3034 |
| 957 | 13473.45 | 13578.9 | 0.1248 | 0.122 | 0.7765 | 16768.71 | 16934.15 | 0.0973 | 0.0965 | 0.977 |
| 958 | 12384.32 | 12663.02 | 0.1241 | 0.127 | 2.2009 | 17864.25 | 17409.46 | 0.1042 | 0.0941 | -2.612 |
| 959 | 12307.13 | 12990.24 | 0.1315 | 0.134 | 5.2586 | 15284.13 | 15950.49 | 0.0961 | 0.0973 | 4.1777 |
| 960 | 13455.81 | 13575.9 | 0.1217 | 0.123 | 0.8846 | 16864.46 | 16888.6 | 0.0967 | 0.1019 | 0.1429 |
| 961 | 11834.04 | 12105.49 | 0.1183 | 0.124 | 2.2424 | 16807.13 | 17345.71 | 0.1167 | 0.1128 | 3.105 |
| 962 | 10990.25 | 11312.45 | 0.114 | 0.11 | 2.8482 | 13200.07 | 13424.22 | 0.1065 | 0.1082 | 1.6697 |
| 963 | 13151.66 | 13100.82 | 0.1196 | 0.118 | -0.388 | 17226.62 | 17422.18 | 0.0993 | 0.0988 | 1.1225 |
| 964 | 14426.27 | 14664.43 | 0.1152 | 0.113 | 1.6241 | 14032.12 | 14139.28 | 0.1014 | 0.1031 | 0.7579 |
| 965 | 12657.83 | 13217.45 | 0.1275 | 0.123 | 4.2339 | 17048.81 | 17387.51 | 0.1021 | 0.1046 | 1.948 |
| 966 | 10718.47 | 10665.84 | 0.1197 | 0.121 | -0.4935 | 12332.16 | 12886.96 | 0.1046 | 0.1104 | 4.3051 |
| 967 | 11510.16 | 13215.5 | 0.1195 | 0.109 | 12.904 | 13069.92 | 13280.92 | 0.1087 | 0.1107 | 1.5888 |
| 968 | 12872.49 | 12826.01 | 0.1062 | 0.105 | -0.3624 | 15779.78 | 15643.92 | 0.1021 | 0.1058 | -0.868 |
| 969 | 12037.9 | 12565.66 | 0.1144 | 0.119 | 4.2001 | 14420.23 | 14535.38 | 0.0952 | 0.0934 | 0.7922 |
| 970 | 15379.14 | 14998.39 | 0.1137 | 0.108 | -2.5386 | 15100.18 | 14737.16 | 0.1004 | 0.0953 | -2.463 |
| 971 | 13774.52 | 13879.96 | 0.1256 | 0.123 | 0.7597 | 13044.79 | 13231.57 | 0.0909 | 0.0925 | 1.4116 |
| 972 | 14179.52 | 14270.44 | 0.1222 | 0.124 | 0.6371 | 17529.31 | 17304.88 | 0.0973 | 0.1018 | -1.297 |
| 973 | 12261.6 | 12820.54 | 0.1105 | 0.104 | 4.3598 | 15060.13 | 15501.43 | 0.1026 | 0.0995 | 2.8468 |
| 974 | 13006.01 | 13465.45 | 0.1288 | 0.124 | 3.412 | 18299.36 | 18925.6 | 0.1127 | 0.109 | 3.3089 |
| 975 | 11478.74 | 11463.39 | 0.1285 | 0.129 | -0.134 | 12942.93 | 12826.36 | 0.1082 | 0.1085 | -0.909 |
| 976 | 12863.38 | 12953.17 | 0.1261 | 0.126 | 0.6932 | 17908.03 | 17372.95 | 0.1063 | 0.0968 | -3.08 |
| 977 | 12196.41 | 12736.95 | 0.1265 | 0.128 | 4.2439 | 16076.3 | 14685.64 | 0.1021 | 0.0806 | -9.47 |
| 978 | 15700.6 | 16301.76 | 0.1314 | 0.127 | 3.6877 | 18511.71 | 18529.29 | 0.1014 | 0.1038 | 0.0948 |
| 979 | 13506.46 | 14597.33 | 0.1125 | 0.115 | 7.4731 | 12251.18 | 12282.39 | 0.1133 | 0.1119 | 0.2541 |
| 980 | 13113.5 | 14170.61 | 0.1225 | 0.124 | 7.4599 | 15539.52 | 14154.72 | 0.1069 | 0.0841 | -9.783 |
| 981 | 11046.19 | 12971.24 | 0.1204 | 0.108 | 14.841 | 14194.5 | 14896.63 | 0.0983 | 0.0992 | 4.7134 |
| 982 | 11822.55 | 12576.8 | 0.1255 | 0.119 | 5.9971 | 13531.02 | 13742.94 | 0.1037 | 0.1013 | 1.5421 |
| 983 | 12254.28 | 12988.58 | 0.1174 | 0.11 | 5.6534 | 14479.95 | 14456.52 | 0.097 | 0.0956 | -0.162 |
| 984 | 14666.24 | 14684.31 | 0.1139 | 0.115 | 0.1231 | 15830.34 | 16286.58 | 0.0985 | 0.0956 | 2.8013 |
| 985 | 11716.97 | 12426.74 | 0.1225 | 0.122 | 5.7117 | 14917.38 | 17084.02 | 0.096 | 0.095 | 12.682 |
| 986 | 12341.74 | 13306.13 | 0.1282 | 0.116 | 7.2477 | 15031.43 | 15660.22 | 0.0948 | 0.0962 | 4.0152 |
| 987 | 12709.08 | 12773.4 | 0.1092 | 0.098 | 0.5035 | 15330.93 | 15103.48 | 0.0928 | 0.0937 | -1.506 |
| 988 | 13891.61 | 15223.2 | 0.1049 | 0.119 | 8.7471 | 13175.69 | 13380.83 | 0.109 | 0.1106 | 1.5331 |
| 989 | 12960.28 | 13050.07 | 0.125 | 0.125 | 0.6881 | 16301.71 | 14919.84 | 0.1063 | 0.0847 | -9.262 |
| 990 | 12950.57 | 13531.81 | 0.1274 | 0.111 | 4.2954 | 16030.67 | 15772.01 | 0.1096 | 0.1012 | -1.64 |
| 991 | 12322.95 | 12649.29 | 0.1074 | 0.104 | 2.58 | 13929.5 | 13955.15 | 0.097 | 0.0989 | 0.1838 |
| 992 | 10728.38 | 11231.97 | 0.1238 | 0.113 | 4.4835 | 16961.73 | 17303.81 | 0.0986 | 0.1029 | 1.9769 |
| 993 | 12085.02 | 12148.15 | 0.1222 | 0.122 | 0.5197 | 14467.17 | 15093.03 | 0.0956 | 0.097 | 4.1467 |
| 994 | 17668.28 | 17968.49 | 0.1184 | 0.117 | 1.6707 | 13208.75 | 13874.32 | 0.0993 | 0.1007 | 4.7971 |
| 995 | 12410.63 | 12478.41 | 0.1167 | 0.117 | 0.5432 | 14804.16 | 14772.03 | 0.102 | 0.1019 | -0.218 |
| 996 | 12666.03 | 12608.04 | 0.1184 | 0.121 | -0.4599 | 14313.15 | 14263.86 | 0.1003 | 0.0989 | -0.346 |
| 997 | 13299.67 | 13405.11 | 0.1272 | 0.125 | 0.7866 | 14341.82 | 14448.98 | 0.1023 | 0.104 | 0.7416 |
| 998 | 11803.3 | 11764.31 | 0.1232 | 0.122 | -0.3315 | 17168.6 | 16876.89 | 0.0981 | 0.1033 | -1.728 |
| 999 | 13306.43 | 13963.17 | 0.1296 | 0.132 | 4.7034 | 14216.61 | 16078.27 | 0.1009 | 0.0973 | 11.579 |
| 1000 | 13098.62 | 14248.15 | 0.1158 | 0.113 | 8.0679 | 11644.26 | 11523.54 | 0.107 | 0.1075 | -1.048 |
|  |  |  |  |  |  |  |  |  |  |  |

|  | Daraga sample unite (3) E-W | | | | | Wuqro sample unite (4) E-W | | | | |
| --- | --- | --- | --- | --- | --- | --- | --- | --- | --- | --- |
|  | UM L | M L | UM C | M C | L Ch % | UM L | M L | UM C | M C | L Ch % |
| 1 | 12565 | 21970.4 | 0.1265 | 0.1309 | 42.809 | 15315.4 | 16178.7 | 0.1255 | 0.1244 | 5.3361 |
| 2 | 13016 | 22460.6 | 0.1297 | 0.1319 | 42.05 | 12448 | 12503.9 | 0.115 | 0.1162 | 0.4467 |
| 3 | 12325.9 | 22561.8 | 0.1175 | 0.1344 | 45.368 | 13196.3 | 13216.8 | 0.131 | 0.1305 | 0.1551 |
| 4 | 11741.2 | 15556.5 | 0.1337 | 0.1235 | 24.525 | 14079.6 | 13992.4 | 0.1271 | 0.1304 | -0.623 |
| 5 | 11522.7 | 11526.6 | 0.1155 | 0.1142 | 0.0334 | 12090.6 | 12487.4 | 0.1371 | 0.1346 | 3.1777 |
| 6 | 11911.2 | 11660 | 0.1327 | 0.1278 | -2.154 | 12709 | 12442 | 0.1387 | 0.136 | -2.146 |
| 7 | 13660.2 | 24111.1 | 0.1211 | 0.1323 | 43.345 | 11531.3 | 11674.4 | 0.1426 | 0.1428 | 1.2253 |
| 8 | 11342.3 | 19035.5 | 0.1355 | 0.1346 | 40.415 | 15295.1 | 15319.7 | 0.1228 | 0.1252 | 0.1603 |
| 9 | 13945.6 | 13962.5 | 0.1277 | 0.1267 | 0.1208 | 11968.2 | 12216.2 | 0.1308 | 0.1324 | 2.0303 |
| 10 | 14488.1 | 26213.1 | 0.1218 | 0.126 | 44.729 | 11287.4 | 11398.5 | 0.1417 | 0.1409 | 0.9749 |
| 11 | 11921.5 | 12220.7 | 0.1321 | 0.1343 | 2.4486 | 11102 | 12500.8 | 0.1432 | 0.1379 | 11.189 |
| 12 | 13278.9 | 25179 | 0.1117 | 0.1253 | 47.262 | 13631.9 | 14061.7 | 0.1237 | 0.1253 | 3.0564 |
| 13 | 11418.5 | 11577.9 | 0.1165 | 0.1226 | 1.3769 | 12707.3 | 12849.4 | 0.1285 | 0.1329 | 1.1055 |
| 14 | 11916.1 | 19011.7 | 0.1217 | 0.1323 | 37.323 | 12169.4 | 12190.9 | 0.1376 | 0.1364 | 0.1764 |
| 15 | 14058.1 | 14299.2 | 0.1218 | 0.1243 | 1.686 | 10817.7 | 12103.7 | 0.1376 | 0.1335 | 10.625 |
| 16 | 15898.5 | 16721.4 | 0.1274 | 0.1259 | 4.9214 | 12769.5 | 12925.5 | 0.1448 | 0.1454 | 1.2068 |
| 17 | 13919.9 | 15825.8 | 0.1263 | 0.1339 | 12.043 | 13197.2 | 13172.8 | 0.1314 | 0.1307 | -0.186 |
| 18 | 12581.6 | 19612.5 | 0.12 | 0.1296 | 35.849 | 13268.7 | 13308.9 | 0.1287 | 0.1293 | 0.3021 |
| 19 | 12315.9 | 12346.6 | 0.1121 | 0.1181 | 0.2487 | 10163.2 | 10454.8 | 0.1388 | 0.1391 | 2.7898 |
| 20 | 11443.8 | 18388.2 | 0.1348 | 0.1309 | 37.766 | 13368.2 | 14043.4 | 0.1156 | 0.1154 | 4.8082 |
| 21 | 10977.8 | 14435.9 | 0.1362 | 0.1354 | 23.955 | 11474.4 | 11816.8 | 0.1341 | 0.1339 | 2.8978 |
| 22 | 13287.1 | 26440.4 | 0.1266 | 0.133 | 49.747 | 15182.1 | 15165.8 | 0.1285 | 0.1288 | -0.108 |
| 23 | 11054.6 | 15727.7 | 0.1346 | 0.1322 | 29.713 | 12185.3 | 12311.4 | 0.1279 | 0.1321 | 1.025 |
| 24 | 14466.8 | 19671.3 | 0.1324 | 0.1306 | 26.457 | 13158.3 | 13267.3 | 0.1141 | 0.1153 | 0.8222 |
| 25 | 12251.2 | 15505.3 | 0.1435 | 0.1398 | 20.987 | 13898.7 | 13889.9 | 0.131 | 0.1307 | -0.063 |
| 26 | 13358.3 | 14495 | 0.12 | 0.1155 | 7.842 | 14355 | 14412 | 0.125 | 0.1262 | 0.3954 |
| 27 | 13211.9 | 18771.9 | 0.1256 | 0.1325 | 29.619 | 11209.6 | 11434.5 | 0.1333 | 0.1339 | 1.9668 |
| 28 | 12246 | 12729.9 | 0.1318 | 0.1306 | 3.8016 | 12112.3 | 12165.7 | 0.1385 | 0.14 | 0.4385 |
| 29 | 11506.6 | 16208.3 | 0.1297 | 0.1343 | 29.008 | 11401.5 | 11450.4 | 0.1351 | 0.1351 | 0.4271 |
| 30 | 11803.9 | 16738.7 | 0.1455 | 0.1414 | 29.481 | 15235.4 | 19327.5 | 0.1267 | 0.1232 | 21.172 |
| 31 | 11368.5 | 11255.4 | 0.1294 | 0.1301 | -1.004 | 12685.7 | 12900.6 | 0.1265 | 0.1287 | 1.6657 |
| 32 | 13420.4 | 14351.2 | 0.1252 | 0.1228 | 6.4855 | 11183.9 | 11481.7 | 0.1336 | 0.1364 | 2.5938 |
| 33 | 15576.3 | 16825.7 | 0.1168 | 0.1233 | 7.4259 | 10794.7 | 10972.4 | 0.1424 | 0.1407 | 1.6195 |
| 34 | 12452.5 | 20762.2 | 0.123 | 0.1242 | 40.023 | 16170.5 | 16201.6 | 0.1262 | 0.1285 | 0.1921 |
| 35 | 13614.7 | 21819.6 | 0.1263 | 0.1321 | 37.603 | 11755.9 | 11979.6 | 0.1334 | 0.1328 | 1.8671 |
| 36 | 10583 | 16972.9 | 0.141 | 0.1399 | 37.648 | 11848.9 | 11739.5 | 0.1382 | 0.1388 | -0.932 |
| 37 | 13362.7 | 20108 | 0.1297 | 0.1296 | 33.546 | 12826.1 | 12976.4 | 0.1276 | 0.132 | 1.1585 |
| 38 | 14091.6 | 14104.3 | 0.1281 | 0.1273 | 0.0902 | 11602.5 | 11539 | 0.1351 | 0.1356 | -0.551 |
| 39 | 12421 | 19012.1 | 0.1259 | 0.1365 | 34.668 | 14299.7 | 14283.4 | 0.1315 | 0.1316 | -0.115 |
| 40 | 13507.4 | 24288.3 | 0.1118 | 0.1309 | 44.387 | 13150.5 | 13438.5 | 0.1388 | 0.1355 | 2.1428 |
| 41 | 15414.4 | 16715.5 | 0.1235 | 0.1298 | 7.7835 | 12355.2 | 13523.1 | 0.1268 | 0.1258 | 8.6358 |
| 42 | 11548 | 15491.9 | 0.1337 | 0.1344 | 25.458 | 12298.2 | 12520.6 | 0.1439 | 0.1446 | 1.7758 |
| 43 | 12362.2 | 25372.8 | 0.1159 | 0.1267 | 51.278 | 12956.1 | 13218.9 | 0.1151 | 0.1165 | 1.9884 |
| 44 | 12222.4 | 19999 | 0.1349 | 0.14 | 38.885 | 11892.8 | 12490.6 | 0.1411 | 0.1366 | 4.7858 |
| 45 | 11585.5 | 17308.8 | 0.1438 | 0.1417 | 33.065 | 12128.4 | 12076.6 | 0.1437 | 0.135 | -0.429 |
| 46 | 15679.7 | 16809.8 | 0.1317 | 0.1341 | 6.7228 | 12466.4 | 12552.3 | 0.1287 | 0.1266 | 0.685 |
| 47 | 11390.3 | 16576.2 | 0.1396 | 0.1295 | 31.285 | 10974.1 | 11160.5 | 0.1327 | 0.1319 | 1.6701 |
| 48 | 13440.1 | 24443.9 | 0.1298 | 0.132 | 45.016 | 11946.7 | 11951.4 | 0.1378 | 0.1365 | 0.0389 |
| 49 | 14098.3 | 14440.9 | 0.1259 | 0.1221 | 2.3721 | 11361.9 | 11485.1 | 0.13 | 0.1341 | 1.0732 |
| 50 | 11984.3 | 11734.7 | 0.1102 | 0.1168 | -2.127 | 13357.2 | 14910.8 | 0.1284 | 0.1272 | 10.419 |
| 51 | 11835.3 | 11536.2 | 0.1103 | 0.1134 | -2.593 | 13465.7 | 13456.9 | 0.1326 | 0.1323 | -0.065 |
| 52 | 11243.7 | 18624 | 0.1369 | 0.1396 | 39.628 | 13055.1 | 13154.7 | 0.1316 | 0.1293 | 0.757 |
| 53 | 13000.9 | 13361 | 0.1287 | 0.1282 | 2.6957 | 13599.8 | 13604 | 0.1179 | 0.118 | 0.0304 |
| 54 | 11981.8 | 14251.8 | 0.1421 | 0.1271 | 15.928 | 12965.5 | 12986 | 0.1315 | 0.131 | 0.1579 |
| 55 | 12334.3 | 22239.9 | 0.1346 | 0.1333 | 44.54 | 12848.3 | 16524 | 0.1309 | 0.1257 | 22.244 |
| 56 | 12465.7 | 14295 | 0.1257 | 0.1294 | 12.797 | 14271.6 | 14469.9 | 0.1266 | 0.128 | 1.3706 |
| 57 | 12333.6 | 23992.5 | 0.131 | 0.1309 | 48.594 | 10804.6 | 10927.7 | 0.1316 | 0.1308 | 1.126 |
| 58 | 11928.4 | 13470.4 | 0.1201 | 0.1301 | 11.447 | 11035.9 | 11311.1 | 0.1432 | 0.1423 | 2.4329 |
| 59 | 12275.2 | 19144.7 | 0.1342 | 0.1405 | 35.882 | 13985.1 | 14023.2 | 0.124 | 0.1233 | 0.2715 |
| 60 | 14166.5 | 14465.4 | 0.1273 | 0.12 | 2.0664 | 11647.8 | 11888 | 0.1341 | 0.1302 | 2.0209 |
| 61 | 11652.1 | 11745.2 | 0.1128 | 0.1125 | 0.793 | 10969.8 | 11315.9 | 0.1394 | 0.1381 | 3.0578 |
| 62 | 17001 | 17075.5 | 0.1286 | 0.1337 | 0.4364 | 11016.1 | 11041.5 | 0.1391 | 0.1367 | 0.2296 |
| 63 | 13100.6 | 13982.4 | 0.1208 | 0.1122 | 6.3065 | 11797.8 | 11969.5 | 0.1399 | 0.1367 | 1.4346 |
| 64 | 14438.6 | 14493.9 | 0.1298 | 0.1199 | 0.3813 | 12244.9 | 12249.1 | 0.1163 | 0.1162 | 0.0338 |
| 65 | 14825.2 | 16155.3 | 0.1231 | 0.1318 | 8.2327 | 12987.3 | 13869.1 | 0.1327 | 0.133 | 6.3578 |
| 66 | 12703.2 | 16623.7 | 0.1365 | 0.1386 | 23.584 | 11233.9 | 11386.3 | 0.1333 | 0.1308 | 1.3379 |
| 67 | 14132.7 | 19696.6 | 0.1313 | 0.1309 | 28.248 | 11672.5 | 11701.5 | 0.1122 | 0.1118 | 0.2478 |
| 68 | 12088.3 | 13650 | 0.1168 | 0.1116 | 11.441 | 12747.4 | 12751.5 | 0.1146 | 0.1144 | 0.0325 |
| 69 | 14502.8 | 15056.8 | 0.1244 | 0.1148 | 3.6792 | 11789.7 | 11796 | 0.1305 | 0.1307 | 0.0539 |
| 70 | 12473.3 | 15371.6 | 0.1437 | 0.1401 | 18.854 | 12280.6 | 12419 | 0.1347 | 0.1349 | 1.1142 |
| 71 | 13676.3 | 14292.7 | 0.1265 | 0.1251 | 4.3124 | 11026.5 | 11263.4 | 0.1392 | 0.1374 | 2.1033 |
| 72 | 11261 | 15041.1 | 0.144 | 0.1393 | 25.132 | 10285.4 | 10573.4 | 0.1411 | 0.1393 | 2.7241 |
| 73 | 12175.5 | 19437.7 | 0.1257 | 0.1329 | 37.361 | 11540 | 11494 | 0.1377 | 0.1358 | -0.401 |
| 74 | 12090.5 | 13716.8 | 0.1421 | 0.1285 | 11.856 | 11101.5 | 11164.7 | 0.1408 | 0.14 | 0.5655 |
| 75 | 12083.6 | 19683.8 | 0.135 | 0.14 | 38.611 | 10845.8 | 11132.8 | 0.1375 | 0.1367 | 2.5781 |
| 76 | 13210 | 13291.2 | 0.115 | 0.1147 | 0.611 | 13159.6 | 14259 | 0.1447 | 0.1388 | 7.7098 |
| 77 | 11568.4 | 13446.9 | 0.1398 | 0.1258 | 13.97 | 11329.8 | 11713.4 | 0.1336 | 0.1326 | 3.2748 |
| 78 | 11116.8 | 17377.1 | 0.1351 | 0.1314 | 36.026 | 12300.6 | 12411.7 | 0.1363 | 0.1356 | 0.8953 |
| 79 | 13884.1 | 14693 | 0.1261 | 0.1307 | 5.5054 | 14398.8 | 15563.7 | 0.1373 | 0.1348 | 7.4848 |
| 80 | 12527.2 | 12400.2 | 0.1179 | 0.1146 | -1.024 | 10864.5 | 11139.9 | 0.1316 | 0.1298 | 2.4721 |
| 81 | 11153.1 | 17949.2 | 0.1426 | 0.1416 | 37.863 | 14090.1 | 14128.2 | 0.122 | 0.1214 | 0.2695 |
| 82 | 12625.8 | 16570.6 | 0.1264 | 0.127 | 23.805 | 12791.9 | 12704.7 | 0.1296 | 0.1336 | -0.686 |
| 83 | 13091.6 | 13327.6 | 0.1323 | 0.1261 | 1.7709 | 10656.5 | 11245.5 | 0.1438 | 0.1428 | 5.2375 |
| 84 | 12021.2 | 12155.7 | 0.1246 | 0.1169 | 1.107 | 10771.6 | 11349.4 | 0.1369 | 0.1338 | 5.0912 |
| 85 | 13077.4 | 22367.1 | 0.138 | 0.1259 | 41.533 | 12826.7 | 12961.9 | 0.1246 | 0.1247 | 1.0429 |
| 86 | 14883 | 21787.4 | 0.1197 | 0.1309 | 31.69 | 10775.3 | 13938.8 | 0.1365 | 0.129 | 22.696 |
| 87 | 11047.7 | 17010.7 | 0.1317 | 0.1376 | 35.055 | 14514.9 | 14640.3 | 0.1241 | 0.1231 | 0.8562 |
| 88 | 12004.2 | 15995.6 | 0.1307 | 0.129 | 24.953 | 11228.1 | 11291.2 | 0.1416 | 0.1409 | 0.5592 |
| 89 | 11334.4 | 18324.6 | 0.1415 | 0.1427 | 38.147 | 11239.1 | 11596.1 | 0.1367 | 0.1382 | 3.0781 |
| 90 | 12460.9 | 19270 | 0.1237 | 0.1258 | 35.335 | 11380.3 | 11651.8 | 0.1396 | 0.1374 | 2.3297 |
| 91 | 13111.6 | 13721.6 | 0.1216 | 0.1144 | 4.4449 | 12721.9 | 13196 | 0.1398 | 0.1399 | 3.5931 |
| 92 | 12215 | 16822.3 | 0.1237 | 0.1226 | 27.388 | 13279.4 | 17063.7 | 0.128 | 0.1236 | 22.178 |
| 93 | 11211.8 | 14564 | 0.1433 | 0.129 | 23.017 | 12267.6 | 12130.3 | 0.1307 | 0.1412 | -1.132 |
| 94 | 13453.9 | 23845.1 | 0.1245 | 0.1304 | 43.578 | 14954.4 | 15974.2 | 0.1308 | 0.1299 | 6.384 |
| 95 | 14349 | 14040.9 | 0.1212 | 0.1163 | -2.194 | 10466.3 | 10508.4 | 0.1421 | 0.1396 | 0.4009 |
| 96 | 12802.7 | 18666.5 | 0.1216 | 0.1207 | 31.413 | 13259.1 | 13242.7 | 0.1344 | 0.1346 | -0.124 |
| 97 | 14107.2 | 19831.4 | 0.1323 | 0.1295 | 28.864 | 13230.2 | 13527.7 | 0.1364 | 0.1376 | 2.1988 |
| 98 | 13760.5 | 13422.7 | 0.1069 | 0.1104 | -2.516 | 13272.4 | 13509.4 | 0.1346 | 0.1359 | 1.7542 |
| 99 | 15470.3 | 20401.4 | 0.1263 | 0.131 | 24.171 | 11241.4 | 11338.6 | 0.141 | 0.1342 | 0.8572 |
| 100 | 13212.9 | 23846.7 | 0.1276 | 0.1311 | 44.592 | 11633.4 | 11797.1 | 0.1355 | 0.1347 | 1.3881 |
| 101 | 12203.3 | 19859.8 | 0.1323 | 0.1315 | 38.552 | 12107.6 | 12270.8 | 0.1426 | 0.1437 | 1.3305 |
| 102 | 12079.5 | 11966.4 | 0.1093 | 0.1154 | -0.945 | 13007.6 | 13219.3 | 0.1396 | 0.1393 | 1.6009 |
| 103 | 12041.3 | 12169.3 | 0.1347 | 0.139 | 1.0524 | 11549.1 | 11738.4 | 0.1336 | 0.1361 | 1.6129 |
| 104 | 11214.9 | 17034.5 | 0.1253 | 0.1344 | 34.164 | 12585.7 | 12871.7 | 0.1278 | 0.1293 | 2.2215 |
| 105 | 13088.1 | 12959.6 | 0.1358 | 0.1316 | -0.991 | 11645.2 | 11645.2 | 0.1151 | 0.1151 | 0 |
| 106 | 13719.6 | 23098.2 | 0.1202 | 0.133 | 40.603 | 12618 | 12982.1 | 0.1336 | 0.1393 | 2.8043 |
| 107 | 14933.5 | 16668 | 0.1245 | 0.1244 | 10.407 | 13145.9 | 13347.3 | 0.1139 | 0.1149 | 1.5091 |
| 108 | 12689.1 | 12693.6 | 0.1339 | 0.1283 | 0.035 | 12403.5 | 12407.7 | 0.1127 | 0.1125 | 0.0334 |
| 109 | 12645.9 | 15199.3 | 0.123 | 0.1299 | 16.8 | 10918.6 | 11163 | 0.1327 | 0.1326 | 2.1892 |
| 110 | 12917.6 | 13321 | 0.1458 | 0.1448 | 3.0288 | 13838.4 | 13751.2 | 0.1284 | 0.1317 | -0.634 |
| 111 | 12507.2 | 13108.3 | 0.1463 | 0.1455 | 4.5855 | 10878.4 | 11125 | 0.1418 | 0.141 | 2.2167 |
| 112 | 12991.8 | 14045 | 0.1213 | 0.1126 | 7.4989 | 12066.8 | 11979.6 | 0.1315 | 0.1352 | -0.728 |
| 113 | 12989.6 | 25345.1 | 0.1144 | 0.1267 | 48.749 | 14602.2 | 14640.3 | 0.121 | 0.1204 | 0.2601 |
| 114 | 13248.5 | 18243.2 | 0.1146 | 0.1231 | 27.378 | 14186.6 | 15104.2 | 0.1353 | 0.1363 | 6.0753 |
| 115 | 12509.7 | 18399.9 | 0.1218 | 0.1298 | 32.012 | 15526.3 | 15842.1 | 0.1278 | 0.1267 | 1.9936 |
| 116 | 11166.8 | 18301.9 | 0.1328 | 0.1349 | 38.986 | 12954.5 | 13221 | 0.1309 | 0.1334 | 2.0153 |
| 117 | 13194.7 | 22334.7 | 0.128 | 0.1309 | 40.923 | 12830.3 | 13484.1 | 0.1361 | 0.1351 | 4.8488 |
| 118 | 13674.4 | 14079.6 | 0.1309 | 0.1307 | 2.8782 | 12153.5 | 12331.6 | 0.1271 | 0.1266 | 1.4444 |
| 119 | 15770.1 | 19886.9 | 0.1267 | 0.1323 | 20.701 | 13564.7 | 13540.8 | 0.124 | 0.125 | -0.177 |
| 120 | 10717.1 | 14829.9 | 0.1375 | 0.1349 | 27.733 | 11299.9 | 11344.2 | 0.1279 | 0.1282 | 0.3909 |
| 121 | 12440.8 | 22699.8 | 0.137 | 0.1314 | 45.194 | 12641.6 | 12920 | 0.1455 | 0.1454 | 2.1548 |
| 122 | 13560.1 | 13347.1 | 0.1224 | 0.126 | -1.596 | 11602.6 | 12063.1 | 0.1354 | 0.1342 | 3.818 |
| 123 | 13757.3 | 20949.2 | 0.1273 | 0.1335 | 34.33 | 11300.2 | 11714.5 | 0.1387 | 0.1371 | 3.5366 |
| 124 | 13421.5 | 15143.2 | 0.1288 | 0.1315 | 11.369 | 13925 | 13796.2 | 0.1269 | 0.1321 | -0.934 |
| 125 | 13358.7 | 15482.2 | 0.1205 | 0.1299 | 13.716 | 14341.1 | 15050.9 | 0.1274 | 0.1261 | 4.7162 |
| 126 | 12909.3 | 13096.5 | 0.1285 | 0.1306 | 1.4293 | 11313 | 11603.7 | 0.1253 | 0.1247 | 2.5049 |
| 127 | 11736.3 | 15934.2 | 0.1189 | 0.1231 | 26.345 | 10178.1 | 10749.8 | 0.1367 | 0.1332 | 5.3182 |
| 128 | 11780.1 | 12270.1 | 0.1347 | 0.1392 | 3.9935 | 11673.1 | 12050.4 | 0.1345 | 0.1372 | 3.1304 |
| 129 | 12475.7 | 14435.1 | 0.1219 | 0.1071 | 13.573 | 11705.2 | 11956.4 | 0.1321 | 0.1339 | 2.1014 |
| 130 | 12930.2 | 22781.1 | 0.1187 | 0.1332 | 43.242 | 13787.8 | 14035.8 | 0.1239 | 0.1256 | 1.7671 |
| 131 | 10917.2 | 12716.5 | 0.1199 | 0.1109 | 14.149 | 11857.1 | 12733.3 | 0.1405 | 0.1372 | 6.8813 |
| 132 | 12992.2 | 23676.8 | 0.1136 | 0.1307 | 45.127 | 11765 | 11897.9 | 0.1387 | 0.1384 | 1.1172 |
| 133 | 12851.1 | 26053.2 | 0.1273 | 0.1333 | 50.674 | 10979.2 | 11222.6 | 0.1338 | 0.1331 | 2.1694 |
| 134 | 12365.4 | 20046.1 | 0.1328 | 0.1405 | 38.315 | 14387.5 | 14300.3 | 0.1271 | 0.1303 | -0.609 |
| 135 | 11379.8 | 12073.9 | 0.1444 | 0.1469 | 5.749 | 11609.5 | 11699.6 | 0.1288 | 0.127 | 0.7703 |
| 136 | 15386.4 | 15947.8 | 0.129 | 0.1337 | 3.5201 | 13504.9 | 13488.5 | 0.1322 | 0.1322 | -0.121 |
| 137 | 10530.9 | 15812.8 | 0.1352 | 0.1367 | 33.403 | 13069.2 | 13723 | 0.1348 | 0.1341 | 4.7644 |
| 138 | 11073.4 | 11085.2 | 0.1366 | 0.1395 | 0.1065 | 11668.5 | 11754.4 | 0.127 | 0.1296 | 0.7307 |
| 139 | 13417.5 | 14352.1 | 0.1301 | 0.1297 | 6.5119 | 11132 | 11360.6 | 0.1349 | 0.1424 | 2.0127 |
| 140 | 12475.1 | 12219.4 | 0.1135 | 0.1168 | -2.092 | 14535.8 | 14592.8 | 0.1206 | 0.1218 | 0.3905 |
| 141 | 14151.6 | 18897.5 | 0.1379 | 0.1309 | 25.114 | 11651.9 | 11684.3 | 0.1394 | 0.1374 | 0.2768 |
| 142 | 14022 | 15789.9 | 0.1282 | 0.1313 | 11.196 | 11467.2 | 11757 | 0.1383 | 0.1455 | 2.4641 |
| 143 | 15933.2 | 19269.4 | 0.1242 | 0.1296 | 17.313 | 10745.6 | 11030.2 | 0.1335 | 0.1331 | 2.5801 |
| 144 | 12186.2 | 13778.8 | 0.1122 | 0.1115 | 11.558 | 10798.7 | 10813.9 | 0.1393 | 0.1366 | 0.1401 |
| 145 | 12682.8 | 13341.7 | 0.1174 | 0.1139 | 4.9387 | 12042.5 | 12029.6 | 0.1413 | 0.1395 | -0.107 |
| 146 | 12307.9 | 12301.2 | 0.1147 | 0.1107 | -0.054 | 11419 | 11651.9 | 0.1415 | 0.1368 | 1.9994 |
| 147 | 13651.3 | 21244.7 | 0.1228 | 0.1245 | 35.742 | 12932.6 | 13058.8 | 0.131 | 0.1348 | 0.9663 |
| 148 | 12512.1 | 14296.2 | 0.114 | 0.1091 | 12.479 | 12419.3 | 12573.6 | 0.1473 | 0.1466 | 1.2276 |
| 149 | 11311.7 | 17632.2 | 0.1261 | 0.135 | 35.846 | 12004.1 | 11741.1 | 0.1414 | 0.1429 | -2.239 |
| 150 | 11751.2 | 17792.6 | 0.1278 | 0.1364 | 33.955 | 11642.4 | 11773.8 | 0.1422 | 0.1443 | 1.1155 |
| 151 | 11938.4 | 14421.5 | 0.1294 | 0.1305 | 17.218 | 11658.6 | 12117.3 | 0.1308 | 0.1297 | 3.7855 |
| 152 | 10988.1 | 17462.6 | 0.1431 | 0.1423 | 37.077 | 14954.2 | 14907.1 | 0.1279 | 0.1303 | -0.316 |
| 153 | 12324.4 | 17696.2 | 0.1207 | 0.1287 | 30.356 | 11777.6 | 11834.6 | 0.1263 | 0.1279 | 0.4815 |
| 154 | 16242.6 | 19974.3 | 0.1244 | 0.1303 | 18.683 | 12232.9 | 12393.7 | 0.1238 | 0.1286 | 1.297 |
| 155 | 15515.9 | 19103.5 | 0.1294 | 0.1313 | 18.78 | 13110.7 | 17406.2 | 0.1271 | 0.1236 | 24.678 |
| 156 | 11138.3 | 19087.6 | 0.1363 | 0.1295 | 41.647 | 11507.8 | 12975.5 | 0.1423 | 0.1393 | 11.312 |
| 157 | 15336.7 | 21776.2 | 0.1193 | 0.1304 | 29.572 | 11106.5 | 11078.7 | 0.139 | 0.1401 | -0.251 |
| 158 | 11436.5 | 20041.1 | 0.1311 | 0.1401 | 42.935 | 11314.5 | 11454.2 | 0.1446 | 0.1438 | 1.2197 |
| 159 | 12016.8 | 11994.3 | 0.1321 | 0.1292 | -0.188 | 11140.1 | 11385.5 | 0.1292 | 0.131 | 2.1553 |
| 160 | 14052.3 | 26195.7 | 0.1129 | 0.1246 | 46.357 | 10947.2 | 10951.4 | 0.1368 | 0.1351 | 0.0386 |
| 161 | 12915.7 | 13338.5 | 0.1198 | 0.1161 | 3.1701 | 13242.5 | 13513.5 | 0.1412 | 0.1418 | 2.0048 |
| 162 | 12755.5 | 14803 | 0.1412 | 0.1376 | 13.831 | 10805.3 | 11024.9 | 0.1342 | 0.1376 | 1.9913 |
| 163 | 11302.3 | 15432.9 | 0.1294 | 0.1325 | 26.765 | 11394.9 | 11703.5 | 0.1335 | 0.1319 | 2.6362 |
| 164 | 11485.9 | 11659.5 | 0.133 | 0.1369 | 1.4885 | 11273.3 | 11449.7 | 0.1321 | 0.1386 | 1.5406 |
| 165 | 12168.8 | 15891.8 | 0.1269 | 0.1258 | 23.427 | 15077.4 | 16008.8 | 0.1352 | 0.128 | 5.8177 |
| 166 | 12808.5 | 12690.5 | 0.1355 | 0.1307 | -0.93 | 12354.5 | 12749.2 | 0.1318 | 0.1302 | 3.0961 |
| 167 | 13355.9 | 24173.9 | 0.1122 | 0.1308 | 44.751 | 10893 | 10935.4 | 0.1387 | 0.137 | 0.388 |
| 168 | 13693.7 | 13706.6 | 0.1358 | 0.1307 | 0.0943 | 14255.4 | 14931.2 | 0.1248 | 0.1243 | 4.5265 |
| 169 | 12236.1 | 13775.1 | 0.1204 | 0.1112 | 11.173 | 11041 | 11145.5 | 0.1435 | 0.1408 | 0.9381 |
| 170 | 11001.1 | 15737.8 | 0.1476 | 0.1429 | 30.098 | 11838.8 | 12558.2 | 0.1291 | 0.1286 | 5.7279 |
| 171 | 12094.8 | 12459.7 | 0.1248 | 0.1183 | 2.9292 | 13321 | 13877 | 0.1267 | 0.1257 | 4.0067 |
| 172 | 12981.7 | 14818 | 0.1364 | 0.1241 | 12.392 | 15979.4 | 16987.2 | 0.1237 | 0.1238 | 5.9331 |
| 173 | 11660.3 | 13311.4 | 0.1224 | 0.1308 | 12.404 | 14601.7 | 15255.6 | 0.1306 | 0.1302 | 4.2857 |
| 174 | 12553.6 | 21591.8 | 0.123 | 0.1337 | 41.859 | 10409.9 | 10648.2 | 0.1394 | 0.1376 | 2.2381 |
| 175 | 11934.3 | 19688.5 | 0.1325 | 0.1255 | 39.384 | 11092.6 | 11402.3 | 0.1378 | 0.1362 | 2.7165 |
| 176 | 12490.7 | 22680.8 | 0.1366 | 0.1312 | 44.928 | 11602.5 | 11768.2 | 0.1409 | 0.1413 | 1.4086 |
| 177 | 14427.9 | 22112.6 | 0.1255 | 0.133 | 34.753 | 14500.9 | 14922.9 | 0.1252 | 0.1253 | 2.8278 |
| 178 | 12377.5 | 13466.6 | 0.1153 | 0.1119 | 8.0873 | 13748.3 | 13874.5 | 0.1287 | 0.1323 | 0.9095 |
| 179 | 13400.9 | 14596.4 | 0.1206 | 0.1322 | 8.1907 | 12934.2 | 13170.1 | 0.1279 | 0.1287 | 1.7915 |
| 180 | 13231.5 | 20745 | 0.1284 | 0.1305 | 36.218 | 12319 | 12558.7 | 0.1324 | 0.1292 | 1.909 |
| 181 | 12820.4 | 13391.5 | 0.144 | 0.1439 | 4.2642 | 12400.8 | 12449.5 | 0.1373 | 0.1364 | 0.3912 |
| 182 | 12252.5 | 20849.2 | 0.1371 | 0.1404 | 41.233 | 11897.5 | 15702.4 | 0.1353 | 0.1286 | 24.231 |
| 183 | 14933.5 | 14878.2 | 0.1216 | 0.1138 | -0.372 | 11518.6 | 11644.8 | 0.1288 | 0.1332 | 1.0836 |
| 184 | 11829 | 19096 | 0.1269 | 0.1342 | 38.055 | 12606.5 | 12663.5 | 0.1245 | 0.126 | 0.45 |
| 185 | 13346.6 | 19108 | 0.1296 | 0.1323 | 30.152 | 11780.4 | 12237.6 | 0.1315 | 0.1332 | 3.7356 |
| 186 | 14079.9 | 15726.6 | 0.1257 | 0.1317 | 10.471 | 11525.9 | 11524.2 | 0.1355 | 0.1335 | -0.014 |
| 187 | 12683.1 | 12651.8 | 0.1269 | 0.1269 | -0.247 | 15104.6 | 16147.5 | 0.1244 | 0.1258 | 6.4587 |
| 188 | 12115.5 | 12396.9 | 0.1166 | 0.1154 | 2.2701 | 11834 | 12062.6 | 0.1305 | 0.1295 | 1.8945 |
| 189 | 12446.4 | 13277.7 | 0.1115 | 0.113 | 6.2608 | 15100.6 | 15157.5 | 0.1182 | 0.1194 | 0.376 |
| 190 | 11752.1 | 19766.5 | 0.1363 | 0.1396 | 40.545 | 12912 | 12923.7 | 0.1347 | 0.1354 | 0.0907 |
| 191 | 11180.7 | 16127 | 0.1408 | 0.1301 | 30.671 | 10336.3 | 10463.5 | 0.1445 | 0.1455 | 1.2156 |
| 192 | 12042.5 | 26739.1 | 0.1303 | 0.1321 | 54.963 | 12125.7 | 12125.7 | 0.1189 | 0.1189 | 0 |
| 193 | 13017.7 | 12858 | 0.1343 | 0.1321 | -1.242 | 11220.4 | 11405.6 | 0.1398 | 0.1408 | 1.6236 |
| 194 | 12504.9 | 17810.9 | 0.124 | 0.133 | 29.791 | 12513.8 | 12456.1 | 0.1446 | 0.1369 | -0.463 |
| 195 | 12407.5 | 18115.5 | 0.1248 | 0.1239 | 31.509 | 15165.9 | 15301.1 | 0.1247 | 0.1247 | 0.8835 |
| 196 | 11891.4 | 18916.9 | 0.1415 | 0.1406 | 37.138 | 11819.3 | 11898.2 | 0.1362 | 0.136 | 0.6629 |
| 197 | 13926.1 | 21646.6 | 0.1233 | 0.1332 | 35.666 | 11703.7 | 11928.6 | 0.1355 | 0.1362 | 1.8853 |
| 198 | 15594.2 | 18955 | 0.1253 | 0.1311 | 17.73 | 11232.2 | 11371.9 | 0.1458 | 0.145 | 1.2285 |
| 199 | 14429.2 | 18145.4 | 0.1338 | 0.1332 | 20.48 | 11590.2 | 11691.7 | 0.1287 | 0.1336 | 0.8685 |
| 200 | 13147.7 | 22803.8 | 0.1384 | 0.1262 | 42.344 | 12372 | 16447.9 | 0.1276 | 0.1237 | 24.781 |
| 201 | 11062.9 | 17741.7 | 0.1445 | 0.1429 | 37.645 | 12261.4 | 12753.9 | 0.1454 | 0.1452 | 3.861 |
| 202 | 11647.4 | 11594.5 | 0.1178 | 0.1204 | -0.457 | 11278 | 11334.1 | 0.1462 | 0.1449 | 0.4947 |
| 203 | 12809.1 | 13705 | 0.1128 | 0.1144 | 6.5367 | 11141.1 | 11790.7 | 0.1345 | 0.1336 | 5.5099 |
| 204 | 13128.7 | 22472 | 0.1211 | 0.1332 | 41.578 | 13208 | 13462.7 | 0.1355 | 0.1309 | 1.8919 |
| 205 | 12846.3 | 23922.7 | 0.1257 | 0.1325 | 46.301 | 12368.6 | 12404.3 | 0.1373 | 0.137 | 0.2874 |
| 206 | 11596.6 | 16028.1 | 0.1398 | 0.136 | 27.648 | 16294.7 | 16420.1 | 0.1229 | 0.1221 | 0.7634 |
| 207 | 13284.5 | 13850.7 | 0.128 | 0.1321 | 4.0875 | 12362.5 | 12444.3 | 0.131 | 0.1309 | 0.6576 |
| 208 | 10991.5 | 17534.5 | 0.1367 | 0.1302 | 37.315 | 11936.8 | 12106.9 | 0.1312 | 0.1369 | 1.4052 |
| 209 | 12436.8 | 22608.1 | 0.114 | 0.133 | 44.989 | 13138.1 | 13279.1 | 0.1343 | 0.1317 | 1.0619 |
| 210 | 12845.2 | 14570.3 | 0.1286 | 0.133 | 11.839 | 13332.6 | 13391.8 | 0.1128 | 0.1138 | 0.4427 |
| 211 | 12662.4 | 19645.6 | 0.1199 | 0.1242 | 35.546 | 10711.7 | 11076 | 0.1353 | 0.1357 | 3.2891 |
| 212 | 12135.9 | 15804.4 | 0.1296 | 0.1345 | 23.212 | 13395.6 | 13812.3 | 0.138 | 0.1353 | 3.0169 |
| 213 | 12424.5 | 12103.1 | 0.1303 | 0.134 | -2.655 | 10243.1 | 10612.9 | 0.1379 | 0.135 | 3.485 |
| 214 | 11411.1 | 17811.8 | 0.1346 | 0.1364 | 35.935 | 11718.8 | 11722.2 | 0.1315 | 0.132 | 0.0293 |
| 215 | 10807.1 | 11597.1 | 0.1362 | 0.1263 | 6.8122 | 14877.2 | 15278.3 | 0.1315 | 0.1301 | 2.6252 |
| 216 | 13927.7 | 14568.8 | 0.1284 | 0.1315 | 4.4004 | 10823.3 | 11165.9 | 0.1354 | 0.1367 | 3.0689 |
| 217 | 11636.8 | 15407.2 | 0.1439 | 0.1275 | 24.472 | 14059.1 | 14042.7 | 0.1327 | 0.133 | -0.117 |
| 218 | 14580.9 | 16271.3 | 0.1232 | 0.126 | 10.389 | 13401.2 | 13384.8 | 0.1346 | 0.1348 | -0.122 |
| 219 | 13617.9 | 26647.1 | 0.1235 | 0.1245 | 48.895 | 11724.4 | 12025.5 | 0.1341 | 0.1341 | 2.5044 |
| 220 | 11947.5 | 11836.3 | 0.1362 | 0.1313 | -0.94 | 11497.9 | 15662.1 | 0.1373 | 0.1298 | 26.588 |
| 221 | 11386.6 | 13835.9 | 0.1371 | 0.1359 | 17.702 | 11089 | 11431.1 | 0.1328 | 0.1295 | 2.9933 |
| 222 | 11774.7 | 15862.1 | 0.1349 | 0.1332 | 25.769 | 11919.5 | 11976.2 | 0.1329 | 0.1315 | 0.4741 |
| 223 | 15816.6 | 16765.4 | 0.1259 | 0.1229 | 5.6592 | 13655.7 | 13884.2 | 0.1247 | 0.1239 | 1.646 |
| 224 | 11895.8 | 16029 | 0.1328 | 0.1302 | 25.786 | 14351.4 | 15497 | 0.1382 | 0.1348 | 7.3925 |
| 225 | 12254.1 | 19679.9 | 0.1246 | 0.1346 | 37.733 | 14688.2 | 15684.7 | 0.1365 | 0.1421 | 6.3533 |
| 226 | 12535.1 | 17098.3 | 0.1225 | 0.128 | 26.688 | 11655.2 | 15594.1 | 0.1382 | 0.1304 | 25.259 |
| 227 | 12530.9 | 23607.9 | 0.1342 | 0.1317 | 46.921 | 11219.8 | 11608.8 | 0.1362 | 0.1348 | 3.3512 |
| 228 | 13341.9 | 13925.2 | 0.1339 | 0.1321 | 4.1891 | 13001.3 | 13005.4 | 0.1148 | 0.1147 | 0.0318 |
| 229 | 15436.7 | 16169.8 | 0.1271 | 0.1247 | 4.5332 | 12759.9 | 12776.3 | 0.1331 | 0.1308 | 0.1281 |
| 230 | 12762.8 | 13311 | 0.1415 | 0.1403 | 4.1184 | 14449.6 | 15858.3 | 0.1221 | 0.1217 | 8.8833 |
| 231 | 13762.5 | 13636.9 | 0.1199 | 0.1223 | -0.921 | 16946 | 17048.6 | 0.13 | 0.1286 | 0.6013 |
| 232 | 13555.7 | 15938.1 | 0.1332 | 0.1293 | 14.948 | 15297.5 | 16393.9 | 0.1435 | 0.1387 | 6.6879 |
| 233 | 12262.8 | 14992.6 | 0.1366 | 0.1325 | 18.207 | 11622.9 | 11885.4 | 0.1459 | 0.146 | 2.209 |
| 234 | 11751 | 16624.5 | 0.1334 | 0.1252 | 29.315 | 12525 | 12525 | 0.1119 | 0.1119 | 0 |
| 235 | 14159 | 21991.3 | 0.1249 | 0.1331 | 35.616 | 12477.8 | 12477.8 | 0.1165 | 0.1166 | 0 |
| 236 | 11896.2 | 13534 | 0.1185 | 0.1282 | 12.102 | 12623.4 | 12820.6 | 0.1431 | 0.1444 | 1.5381 |
| 237 | 12517.4 | 17608.3 | 0.1304 | 0.1362 | 28.912 | 11690 | 11690 | 0.1153 | 0.1153 | 0 |
| 238 | 11547.6 | 11890.2 | 0.1349 | 0.1323 | 2.8819 | 12306 | 12526.1 | 0.1371 | 0.137 | 1.757 |
| 239 | 12288.4 | 19561.8 | 0.1277 | 0.133 | 37.181 | 11395.6 | 11468.2 | 0.1392 | 0.1378 | 0.6334 |
| 240 | 11415.5 | 11669.6 | 0.1406 | 0.1399 | 2.1771 | 13661.3 | 13645 | 0.1325 | 0.1327 | -0.12 |
| 241 | 13431.7 | 13501.3 | 0.1308 | 0.1277 | 0.5156 | 11242.3 | 11563.4 | 0.1471 | 0.1452 | 2.7771 |
| 242 | 12295.9 | 17960.5 | 0.1309 | 0.1289 | 31.539 | 12074.7 | 12078.9 | 0.1355 | 0.1341 | 0.035 |
| 243 | 12759.3 | 22257 | 0.1219 | 0.1361 | 42.673 | 12882.5 | 13767.2 | 0.1347 | 0.1349 | 6.4261 |
| 244 | 11454.9 | 18765.2 | 0.1376 | 0.1397 | 38.957 | 12216 | 12340.1 | 0.1372 | 0.1339 | 1.0053 |
| 245 | 16463.4 | 16785.9 | 0.127 | 0.1267 | 1.9208 | 10656.4 | 10859.3 | 0.1365 | 0.1364 | 1.869 |
| 246 | 12602 | 13173 | 0.1334 | 0.1314 | 4.334 | 11046.2 | 11079.4 | 0.1417 | 0.1416 | 0.2991 |
| 247 | 10081.1 | 16370.7 | 0.1457 | 0.1423 | 38.42 | 12298.7 | 12315.9 | 0.1397 | 0.1417 | 0.1396 |
| 248 | 10754.7 | 16353.8 | 0.1332 | 0.127 | 34.237 | 12776.1 | 12776.1 | 0.117 | 0.117 | 0 |
| 249 | 11269.8 | 11323.8 | 0.1117 | 0.1187 | 0.4766 | 11957.7 | 12050.6 | 0.1462 | 0.1427 | 0.7704 |
| 250 | 13674.9 | 20567.8 | 0.1235 | 0.1338 | 33.513 | 13810.1 | 13867.1 | 0.125 | 0.1263 | 0.4109 |
| 251 | 11587 | 20460.1 | 0.1309 | 0.1371 | 43.368 | 10633.6 | 12593.3 | 0.1447 | 0.1406 | 15.561 |
| 252 | 12175.3 | 12116.9 | 0.1186 | 0.1136 | -0.483 | 12325.5 | 12392.6 | 0.1255 | 0.1253 | 0.5412 |
| 253 | 11999.7 | 12054.5 | 0.1153 | 0.1149 | 0.455 | 15020.5 | 15674.3 | 0.1296 | 0.1294 | 4.1712 |
| 254 | 14095.5 | 21526.3 | 0.1272 | 0.1327 | 34.519 | 11661.1 | 11717.9 | 0.1275 | 0.1261 | 0.4845 |
| 255 | 17088.6 | 20262 | 0.1243 | 0.128 | 15.662 | 13464.3 | 13447.9 | 0.1308 | 0.1311 | -0.122 |
| 256 | 14408.8 | 16660 | 0.1243 | 0.1339 | 13.512 | 10689.2 | 10886.3 | 0.1349 | 0.1345 | 1.8106 |
| 257 | 14746.3 | 16047 | 0.1308 | 0.1308 | 8.1059 | 11928.3 | 12345.2 | 0.1318 | 0.1344 | 3.3773 |
| 258 | 15229.4 | 15161.9 | 0.1275 | 0.1268 | -0.445 | 11900.7 | 12139.9 | 0.1383 | 0.136 | 1.9704 |
| 259 | 12575.4 | 18081.2 | 0.1238 | 0.1226 | 30.451 | 10936.6 | 11115.5 | 0.1354 | 0.1364 | 1.6088 |
| 260 | 15379.2 | 17248 | 0.1248 | 0.1315 | 10.834 | 14825.1 | 15478.9 | 0.1308 | 0.1305 | 4.2239 |
| 261 | 13032 | 25431.3 | 0.1143 | 0.1261 | 48.756 | 11527.4 | 11775.2 | 0.1341 | 0.1295 | 2.1046 |
| 262 | 13571.1 | 14192.2 | 0.1353 | 0.1353 | 4.3763 | 12064 | 12307.9 | 0.1389 | 0.1386 | 1.9822 |
| 263 | 12145.4 | 20062.7 | 0.1312 | 0.1285 | 39.463 | 15316.5 | 15311.8 | 0.1248 | 0.1257 | -0.03 |
| 264 | 13201.2 | 13314.6 | 0.1149 | 0.1146 | 0.8513 | 11978.7 | 12048.6 | 0.1357 | 0.137 | 0.5803 |
| 265 | 16648 | 17545.2 | 0.1266 | 0.122 | 5.1136 | 12658.6 | 16556.2 | 0.1274 | 0.1231 | 23.541 |
| 266 | 14664.6 | 15019.5 | 0.1247 | 0.1174 | 2.3629 | 12464.7 | 12754.7 | 0.1314 | 0.1284 | 2.274 |
| 267 | 11248.6 | 11247.5 | 0.113 | 0.1134 | -0.01 | 13343.7 | 13443.3 | 0.1331 | 0.1307 | 0.7408 |
| 268 | 14153.6 | 16200 | 0.1282 | 0.131 | 12.633 | 12980 | 12892.9 | 0.1301 | 0.1338 | -0.676 |
| 269 | 14546.3 | 18209.2 | 0.1342 | 0.1333 | 20.116 | 14380.7 | 14418.8 | 0.1218 | 0.1211 | 0.2641 |
| 270 | 13573.6 | 21804.1 | 0.1257 | 0.1313 | 37.748 | 13170.8 | 13619.4 | 0.132 | 0.131 | 3.2934 |
| 271 | 11332.1 | 16907.4 | 0.126 | 0.1332 | 32.976 | 11927.4 | 11876.5 | 0.1289 | 0.1304 | -0.429 |
| 272 | 13682.3 | 22090.3 | 0.1344 | 0.1312 | 38.062 | 11055.3 | 11241 | 0.1355 | 0.1342 | 1.6519 |
| 273 | 11802 | 18324.1 | 0.1325 | 0.1399 | 35.593 | 15267.9 | 15474.1 | 0.1282 | 0.1291 | 1.3328 |
| 274 | 13123.7 | 21450.8 | 0.1297 | 0.1214 | 38.819 | 11376.4 | 11376.4 | 0.1117 | 0.1117 | 0 |
| 275 | 11480.1 | 15005.2 | 0.1433 | 0.1399 | 23.492 | 13079.9 | 13216.9 | 0.1329 | 0.1314 | 1.0371 |
| 276 | 13733.8 | 13442.2 | 0.1062 | 0.1124 | -2.169 | 14287.5 | 14465 | 0.1284 | 0.1292 | 1.2268 |
| 277 | 11326.7 | 11330.6 | 0.1134 | 0.1136 | 0.0347 | 11652.8 | 12646.3 | 0.1456 | 0.1399 | 7.8561 |
| 278 | 16255.1 | 19314.3 | 0.1247 | 0.1295 | 15.839 | 11780.3 | 12434.1 | 0.1353 | 0.1348 | 5.2582 |
| 279 | 11786.2 | 18632.8 | 0.1254 | 0.1327 | 36.745 | 10513.5 | 10734.3 | 0.1356 | 0.1377 | 2.0565 |
| 280 | 11812.6 | 18614.8 | 0.1236 | 0.1333 | 36.542 | 13760.6 | 14896.6 | 0.1377 | 0.1433 | 7.6253 |
| 281 | 12652.1 | 15413.2 | 0.1427 | 0.1404 | 17.914 | 12386.2 | 12386.2 | 0.1131 | 0.1131 | 0 |
| 282 | 13942.8 | 21667.8 | 0.1289 | 0.1295 | 35.652 | 11057.9 | 11115.1 | 0.1359 | 0.1355 | 0.5146 |
| 283 | 11911.5 | 15513.1 | 0.1423 | 0.1267 | 23.217 | 13723.3 | 13849.5 | 0.1298 | 0.1335 | 0.9111 |
| 284 | 11727.2 | 11812.8 | 0.1166 | 0.1198 | 0.7243 | 11230.3 | 11752.5 | 0.1379 | 0.1338 | 4.4433 |
| 285 | 11741.7 | 12092 | 0.1283 | 0.1267 | 2.8972 | 12448.2 | 12452.3 | 0.1161 | 0.116 | 0.0333 |
| 286 | 14210.8 | 21578.4 | 0.1195 | 0.1319 | 34.143 | 12532.7 | 12586.1 | 0.1353 | 0.1368 | 0.4238 |
| 287 | 11718.5 | 11741.3 | 0.1328 | 0.1382 | 0.1942 | 13139.1 | 13237.7 | 0.1298 | 0.1291 | 0.745 |
| 288 | 11866.1 | 18140.1 | 0.1385 | 0.1371 | 34.586 | 13128.2 | 13407.2 | 0.1428 | 0.1427 | 2.0809 |
| 289 | 13360.6 | 20516.2 | 0.1232 | 0.1213 | 34.878 | 14915.3 | 15569.1 | 0.1315 | 0.1312 | 4.1994 |
| 290 | 16972.3 | 17261.6 | 0.1227 | 0.1208 | 1.6759 | 10635.6 | 10807.8 | 0.1411 | 0.1383 | 1.5935 |
| 291 | 15837.4 | 16758.1 | 0.1263 | 0.1217 | 5.4936 | 11638.9 | 11761.1 | 0.1384 | 0.1397 | 1.0392 |
| 292 | 11144.5 | 11425.6 | 0.1332 | 0.1389 | 2.4608 | 11738.5 | 11830.2 | 0.1378 | 0.1372 | 0.7745 |
| 293 | 13767.1 | 21639.8 | 0.1322 | 0.129 | 36.381 | 11315.8 | 11331.3 | 0.1392 | 0.1459 | 0.1366 |
| 294 | 13593.3 | 20465 | 0.1383 | 0.1277 | 33.578 | 12222.5 | 12222.5 | 0.1171 | 0.1171 | 0 |
| 295 | 11517.7 | 16339.7 | 0.1413 | 0.1302 | 29.511 | 12619.8 | 12603.4 | 0.134 | 0.1342 | -0.13 |
| 296 | 11403.1 | 14175.2 | 0.1143 | 0.1107 | 19.556 | 10659.9 | 11163 | 0.1298 | 0.1306 | 4.507 |
| 297 | 13064.3 | 23891.1 | 0.1249 | 0.1299 | 45.317 | 13451.3 | 13641.5 | 0.1278 | 0.1291 | 1.3943 |
| 298 | 13283.7 | 23649.4 | 0.113 | 0.1298 | 43.831 | 10294.6 | 10594.8 | 0.1311 | 0.1295 | 2.8339 |
| 299 | 14138 | 16454.1 | 0.1226 | 0.1215 | 14.076 | 12970.1 | 13252.7 | 0.1272 | 0.1274 | 2.1327 |
| 300 | 11512 | 14221.6 | 0.1167 | 0.1082 | 19.052 | 12311 | 12406.7 | 0.1348 | 0.1321 | 0.771 |
| 301 | 15430.9 | 16412.8 | 0.1285 | 0.1271 | 5.9827 | 14274.5 | 15253.3 | 0.1261 | 0.1273 | 6.4171 |
| 302 | 14552.4 | 21947.6 | 0.1254 | 0.1303 | 33.695 | 11848.4 | 12227.6 | 0.137 | 0.1344 | 3.1015 |
| 303 | 13029.6 | 25197.2 | 0.1299 | 0.1327 | 48.29 | 11132.3 | 11482.5 | 0.1448 | 0.1421 | 3.0495 |
| 304 | 13303.7 | 20568.6 | 0.1352 | 0.1286 | 35.32 | 10983.4 | 11663.5 | 0.1403 | 0.1371 | 5.8311 |
| 305 | 15407.5 | 15844.7 | 0.1272 | 0.1265 | 2.759 | 11990.2 | 11994.3 | 0.1134 | 0.1133 | 0.0345 |
| 306 | 11847.7 | 11759.2 | 0.1182 | 0.1145 | -0.753 | 10878.3 | 16509.1 | 0.1375 | 0.1294 | 34.107 |
| 307 | 12683.4 | 23231.2 | 0.1167 | 0.1334 | 45.404 | 15618.4 | 15531.2 | 0.1283 | 0.1313 | -0.561 |
| 308 | 11469.3 | 15471.3 | 0.1461 | 0.1401 | 25.867 | 13650.4 | 13751.5 | 0.1303 | 0.1297 | 0.7348 |
| 309 | 12972.4 | 13580.5 | 0.1297 | 0.1286 | 4.4776 | 11434.1 | 11716.1 | 0.1408 | 0.1405 | 2.4066 |
| 310 | 13650.4 | 13539.9 | 0.1365 | 0.1319 | -0.816 | 12422.2 | 12568.8 | 0.1282 | 0.1266 | 1.1664 |
| 311 | 14525.9 | 14487 | 0.1263 | 0.1194 | -0.269 | 15415.1 | 16995.8 | 0.1264 | 0.1251 | 9.3008 |
| 312 | 12625.8 | 16999.5 | 0.1447 | 0.1402 | 25.729 | 13505.4 | 13613.5 | 0.1237 | 0.1273 | 0.7942 |
| 313 | 15459.1 | 19858 | 0.1291 | 0.1298 | 22.152 | 15124.5 | 16022.4 | 0.1278 | 0.1264 | 5.604 |
| 314 | 11167 | 11411.1 | 0.1404 | 0.141 | 2.1388 | 13520.2 | 13975.1 | 0.1339 | 0.1312 | 3.2551 |
| 315 | 10985.8 | 19175.1 | 0.1402 | 0.1408 | 42.708 | 11981.5 | 11994.4 | 0.1301 | 0.1326 | 0.1078 |
| 316 | 11529.3 | 11733.4 | 0.1143 | 0.1164 | 1.7398 | 11927.1 | 11970.3 | 0.1452 | 0.1453 | 0.3604 |
| 317 | 12080.3 | 18869.8 | 0.1241 | 0.1322 | 35.981 | 11771.4 | 12240.4 | 0.142 | 0.1423 | 3.8318 |
| 318 | 13501.2 | 13381 | 0.1329 | 0.1273 | -0.898 | 13073.1 | 13111.2 | 0.1242 | 0.1234 | 0.2904 |
| 319 | 11319 | 16347 | 0.137 | 0.1386 | 30.758 | 11392.9 | 11726 | 0.1334 | 0.1355 | 2.8406 |
| 320 | 12603.9 | 21196.1 | 0.134 | 0.1336 | 40.537 | 15089.5 | 15971.3 | 0.1315 | 0.1324 | 5.521 |
| 321 | 11703.7 | 15168.5 | 0.1322 | 0.1342 | 22.842 | 10563.1 | 10601.4 | 0.1414 | 0.1395 | 0.3611 |
| 322 | 12490.1 | 17737.4 | 0.1381 | 0.1394 | 29.583 | 14709.1 | 15727.2 | 0.1354 | 0.1404 | 6.4737 |
| 323 | 16127.9 | 16844.4 | 0.1258 | 0.1211 | 4.2533 | 12493.5 | 12597.5 | 0.1251 | 0.1283 | 0.8253 |
| 324 | 13548.8 | 24344.6 | 0.1142 | 0.1294 | 44.346 | 11152.1 | 11214.4 | 0.1335 | 0.1407 | 0.5559 |
| 325 | 12272.7 | 12091.7 | 0.1321 | 0.1331 | -1.496 | 14468.9 | 15350.7 | 0.1332 | 0.1335 | 5.7441 |
| 326 | 12713.2 | 13258.3 | 0.1138 | 0.114 | 4.1108 | 11931 | 12039.9 | 0.1287 | 0.131 | 0.9046 |
| 327 | 11934.2 | 16334.9 | 0.1315 | 0.1267 | 26.94 | 11129.2 | 11203.2 | 0.137 | 0.1376 | 0.661 |
| 328 | 13014.9 | 18191.9 | 0.1342 | 0.1305 | 28.458 | 13706 | 14393.2 | 0.1343 | 0.1339 | 4.7748 |
| 329 | 11900.3 | 12009.3 | 0.1126 | 0.1138 | 0.9076 | 14181.2 | 14852.1 | 0.1338 | 0.1347 | 4.5171 |
| 330 | 12593 | 19095.7 | 0.1361 | 0.1407 | 34.053 | 15805.3 | 13617.2 | 0.122 | 0.113 | -16.07 |
| 331 | 12562 | 12856.7 | 0.1178 | 0.1123 | 2.2924 | 11864.6 | 11976.9 | 0.1279 | 0.1262 | 0.9373 |
| 332 | 12635 | 20660.6 | 0.1349 | 0.139 | 38.845 | 12421.4 | 12708 | 0.1311 | 0.1308 | 2.255 |
| 333 | 12746.4 | 19209.1 | 0.1368 | 0.1413 | 33.644 | 11106.5 | 11097 | 0.1334 | 0.1351 | -0.086 |
| 334 | 12172.8 | 14579.3 | 0.1437 | 0.1294 | 16.507 | 14331.7 | 14560.2 | 0.1255 | 0.1248 | 1.5695 |
| 335 | 11554 | 16055 | 0.1283 | 0.1324 | 28.035 | 15098.8 | 15939.8 | 0.1398 | 0.1412 | 5.2759 |
| 336 | 14410.9 | 17080.9 | 0.1292 | 0.1303 | 15.631 | 11646.5 | 11935.1 | 0.1323 | 0.1336 | 2.4175 |
| 337 | 11545.4 | 18277.9 | 0.1322 | 0.1395 | 36.834 | 12007.7 | 16090 | 0.1345 | 0.1291 | 25.372 |
| 338 | 13715.3 | 13627.5 | 0.1257 | 0.1252 | -0.644 | 11976.5 | 11976.5 | 0.1122 | 0.1123 | 0 |
| 339 | 12392.4 | 12657.9 | 0.1133 | 0.1157 | 2.0977 | 13443.7 | 13901.5 | 0.1316 | 0.129 | 3.2934 |
| 340 | 13196.4 | 13798.9 | 0.1289 | 0.1308 | 4.3664 | 12761 | 12818 | 0.1238 | 0.1252 | 0.4446 |
| 341 | 11721.3 | 14906.4 | 0.1289 | 0.1309 | 21.367 | 13142 | 13199 | 0.1249 | 0.1263 | 0.4317 |
| 342 | 13124.6 | 13820.9 | 0.1279 | 0.1255 | 5.0384 | 13720.8 | 14401 | 0.1343 | 0.134 | 4.7231 |
| 343 | 12055.8 | 18127 | 0.1252 | 0.1335 | 33.492 | 14047.3 | 14084.5 | 0.1154 | 0.1151 | 0.2647 |
| 344 | 11833.5 | 14839.3 | 0.1452 | 0.1422 | 20.256 | 12037.1 | 12099.3 | 0.1294 | 0.1308 | 0.5135 |
| 345 | 11723.9 | 18980.5 | 0.1395 | 0.1424 | 38.232 | 11229.8 | 11356 | 0.129 | 0.1335 | 1.1112 |
| 346 | 14594 | 19977.3 | 0.1286 | 0.1318 | 26.947 | 13408.8 | 13495.3 | 0.1282 | 0.1286 | 0.6415 |
| 347 | 12349.1 | 12169.4 | 0.1092 | 0.1155 | -1.476 | 12267.2 | 12326.7 | 0.134 | 0.13 | 0.4827 |
| 348 | 12158.7 | 18599.1 | 0.124 | 0.1362 | 34.627 | 15060.7 | 16543.3 | 0.1267 | 0.1258 | 8.9623 |
| 349 | 12191.7 | 12724.1 | 0.11 | 0.1112 | 4.184 | 11084.8 | 11109.2 | 0.1286 | 0.1291 | 0.2192 |
| 350 | 13461.4 | 22682.5 | 0.1235 | 0.1327 | 40.653 | 10838.6 | 10862.3 | 0.136 | 0.1373 | 0.2176 |
| 351 | 15787.2 | 22157.8 | 0.1182 | 0.1296 | 28.751 | 11579.4 | 11875.2 | 0.1346 | 0.1371 | 2.491 |
| 352 | 14420.1 | 20165.3 | 0.1291 | 0.1314 | 28.49 | 13648.1 | 13844.4 | 0.1283 | 0.1286 | 1.4177 |
| 353 | 11328.2 | 12058.7 | 0.1332 | 0.1358 | 6.0578 | 12885.5 | 13058.7 | 0.1275 | 0.1299 | 1.3268 |
| 354 | 12397.9 | 19475.3 | 0.1366 | 0.1407 | 36.341 | 10450.1 | 10635.2 | 0.1449 | 0.1456 | 1.7412 |
| 355 | 13422.9 | 23407.6 | 0.1142 | 0.1331 | 42.656 | 11992.8 | 12061.6 | 0.1308 | 0.1338 | 0.5706 |
| 356 | 12750.9 | 14883.2 | 0.1216 | 0.132 | 14.327 | 13869.4 | 14287.2 | 0.1294 | 0.1305 | 2.9242 |
| 357 | 11360 | 19307.9 | 0.1358 | 0.1301 | 41.164 | 12103.5 | 12154.1 | 0.1307 | 0.1307 | 0.4165 |
| 358 | 14080.1 | 23150.9 | 0.1207 | 0.1331 | 39.181 | 11209.9 | 11375.6 | 0.1335 | 0.1371 | 1.4565 |
| 359 | 14192.3 | 16100.9 | 0.125 | 0.1307 | 11.854 | 14149 | 14377.3 | 0.1214 | 0.124 | 1.5878 |
| 360 | 12460.8 | 12459.9 | 0.1272 | 0.119 | -0.007 | 11973.7 | 11977.9 | 0.1156 | 0.1155 | 0.0346 |
| 361 | 11637.6 | 18561.2 | 0.1243 | 0.1337 | 37.301 | 13200.9 | 13257.9 | 0.1232 | 0.1245 | 0.4298 |
| 362 | 14681 | 20137.8 | 0.1267 | 0.1319 | 27.097 | 11456.4 | 11538.1 | 0.1309 | 0.1309 | 0.7075 |
| 363 | 12611.5 | 13253.6 | 0.1455 | 0.1468 | 4.8446 | 11151.9 | 11378.3 | 0.1332 | 0.1343 | 1.9897 |
| 364 | 13208.9 | 23142.5 | 0.1148 | 0.134 | 42.924 | 13244.9 | 13304.1 | 0.1293 | 0.1313 | 0.445 |
| 365 | 11953.8 | 13831.4 | 0.1155 | 0.1058 | 13.575 | 12434.2 | 12484.9 | 0.131 | 0.1312 | 0.4055 |
| 366 | 14430 | 14434.5 | 0.1299 | 0.1289 | 0.0307 | 11796.7 | 11847.3 | 0.1319 | 0.132 | 0.4273 |
| 367 | 13418.9 | 19207.7 | 0.1287 | 0.129 | 30.138 | 15247.6 | 15453.9 | 0.1275 | 0.1283 | 1.3345 |
| 368 | 14187.9 | 21148.9 | 0.1195 | 0.1329 | 32.914 | 11987.2 | 12163.2 | 0.1461 | 0.1461 | 1.4468 |
| 369 | 12752 | 13216.3 | 0.1253 | 0.1162 | 3.5132 | 12015.4 | 11914.4 | 0.1243 | 0.1242 | -0.847 |
| 370 | 11692.6 | 18118.4 | 0.1341 | 0.1321 | 35.466 | 13760.9 | 14406.8 | 0.1337 | 0.1302 | 4.4836 |
| 371 | 11747.8 | 19181.2 | 0.1248 | 0.1349 | 38.754 | 10933.7 | 11075.8 | 0.1327 | 0.138 | 1.2825 |
| 372 | 13062.8 | 23773.7 | 0.1157 | 0.1304 | 45.054 | 12538.9 | 12606.4 | 0.1354 | 0.1377 | 0.5353 |
| 373 | 12644.5 | 23541.4 | 0.116 | 0.132 | 46.288 | 11113.8 | 11156.3 | 0.1389 | 0.1354 | 0.3811 |
| 374 | 15093.3 | 20108.2 | 0.1283 | 0.1288 | 24.94 | 12778.7 | 13432.6 | 0.1337 | 0.1334 | 4.8674 |
| 375 | 16808.6 | 17478.8 | 0.1237 | 0.1228 | 3.8344 | 11985.4 | 12045.5 | 0.1391 | 0.1389 | 0.4991 |
| 376 | 12295.1 | 12244.6 | 0.1116 | 0.1108 | -0.412 | 13945 | 14192.5 | 0.1128 | 0.1127 | 1.7438 |
| 377 | 17100.2 | 17821.5 | 0.1256 | 0.1305 | 4.0474 | 10725.9 | 11039.7 | 0.138 | 0.1361 | 2.8432 |
| 378 | 11403.1 | 17889.9 | 0.1397 | 0.1378 | 36.259 | 11137.8 | 11219.6 | 0.1276 | 0.1286 | 0.7294 |
| 379 | 14265.7 | 21863.2 | 0.1272 | 0.1329 | 34.75 | 13427.2 | 14230.8 | 0.138 | 0.1408 | 5.647 |
| 380 | 12812.9 | 12993.1 | 0.1292 | 0.1245 | 1.3866 | 10862 | 16030.4 | 0.1366 | 0.1287 | 32.241 |
| 381 | 13669.3 | 15515.9 | 0.1308 | 0.1319 | 11.901 | 12725.6 | 13040.6 | 0.1361 | 0.1374 | 2.4157 |
| 382 | 13651.6 | 24758.3 | 0.1263 | 0.1303 | 44.861 | 10474.1 | 10826.4 | 0.138 | 0.1361 | 3.2537 |
| 383 | 13993.5 | 20212.5 | 0.1318 | 0.1302 | 30.768 | 13325.3 | 13666.9 | 0.1272 | 0.1283 | 2.4993 |
| 384 | 16173 | 20692.2 | 0.1248 | 0.1319 | 21.84 | 13822.7 | 14008.6 | 0.1269 | 0.1285 | 1.327 |
| 385 | 11663.8 | 11823 | 0.133 | 0.1303 | 1.3466 | 11488.8 | 11642.3 | 0.1313 | 0.1292 | 1.3189 |
| 386 | 11754.7 | 15196 | 0.1451 | 0.1292 | 22.646 | 12729 | 13124.8 | 0.1413 | 0.142 | 3.0161 |
| 387 | 13049.2 | 21842.2 | 0.1337 | 0.1312 | 40.257 | 10654.6 | 10868.3 | 0.1384 | 0.135 | 1.9668 |
| 388 | 11817.7 | 18443.8 | 0.1296 | 0.1338 | 35.926 | 12551.5 | 12716.1 | 0.1299 | 0.1299 | 1.2941 |
| 389 | 11823.8 | 18461.6 | 0.1369 | 0.1402 | 35.954 | 10887.6 | 10906.6 | 0.1408 | 0.1377 | 0.1742 |
| 390 | 12054.7 | 12058.7 | 0.1114 | 0.1116 | 0.0326 | 11392.1 | 11392.1 | 0.1129 | 0.1129 | 0 |
| 391 | 11191.1 | 14762.1 | 0.1413 | 0.1286 | 24.19 | 13850.9 | 14167.7 | 0.123 | 0.1237 | 2.2356 |
| 392 | 12446.2 | 16217.2 | 0.1203 | 0.1295 | 23.253 | 11891.1 | 11891.1 | 0.1161 | 0.1159 | 0 |
| 393 | 12517.6 | 18881.5 | 0.1336 | 0.132 | 33.705 | 12106.8 | 12306.4 | 0.1292 | 0.1316 | 1.6221 |
| 394 | 13515.4 | 20844.9 | 0.1343 | 0.1269 | 35.162 | 11969.7 | 12029.4 | 0.1431 | 0.1424 | 0.4963 |
| 395 | 11491.2 | 17284.2 | 0.1389 | 0.1297 | 33.516 | 14911.7 | 16706.8 | 0.1341 | 0.1336 | 10.745 |
| 396 | 11454.9 | 11579.9 | 0.1343 | 0.1319 | 1.08 | 12779.2 | 12642.2 | 0.1295 | 0.1341 | -1.083 |
| 397 | 14086.6 | 14122 | 0.133 | 0.1284 | 0.2504 | 11980 | 12225.3 | 0.1334 | 0.1333 | 2.0062 |
| 398 | 11908.2 | 12458 | 0.1442 | 0.1468 | 4.4137 | 11047.6 | 11308.8 | 0.1338 | 0.1345 | 2.3094 |
| 399 | 11793.7 | 17242.9 | 0.1252 | 0.1302 | 31.602 | 11509 | 11683 | 0.1347 | 0.1377 | 1.4891 |
| 400 | 12186.5 | 11961.4 | 0.1298 | 0.1299 | -1.882 | 13063.8 | 13181.7 | 0.1299 | 0.1338 | 0.8945 |
| 401 | 13191.9 | 13714.7 | 0.1274 | 0.126 | 3.812 | 12014.1 | 12014.1 | 0.1167 | 0.1168 | 0 |
| 402 | 11239 | 11525.2 | 0.1321 | 0.1276 | 2.4832 | 11305.7 | 15588.8 | 0.1341 | 0.1283 | 27.475 |
| 403 | 12332.7 | 18446.4 | 0.1265 | 0.1355 | 33.143 | 11993 | 12014.5 | 0.143 | 0.1419 | 0.179 |
| 404 | 12894.9 | 13337.9 | 0.1312 | 0.1307 | 3.3215 | 11711.8 | 11829.7 | 0.1273 | 0.1313 | 0.9967 |
| 405 | 13137.6 | 13688.1 | 0.1122 | 0.1134 | 4.0214 | 11653 | 11719.5 | 0.1297 | 0.1333 | 0.5673 |
| 406 | 11667.8 | 16528.9 | 0.136 | 0.1274 | 29.409 | 13616.2 | 14468.7 | 0.1144 | 0.1148 | 5.8921 |
| 407 | 11142.2 | 17407.1 | 0.1325 | 0.131 | 35.99 | 11769.5 | 12158.1 | 0.1349 | 0.1305 | 3.1956 |
| 408 | 14038.2 | 21842.9 | 0.1219 | 0.1317 | 35.731 | 13776.3 | 13805.6 | 0.1293 | 0.1286 | 0.2122 |
| 409 | 15117.7 | 14797.6 | 0.1343 | 0.1301 | -2.163 | 14901.8 | 15123 | 0.1303 | 0.1291 | 1.4625 |
| 410 | 14504.6 | 14796.9 | 0.1252 | 0.1183 | 1.9757 | 14237.4 | 14465.9 | 0.1272 | 0.1264 | 1.5798 |
| 411 | 13153.6 | 25652.9 | 0.1132 | 0.1272 | 48.725 | 15218 | 15984.4 | 0.1253 | 0.1243 | 4.7947 |
| 412 | 11437.1 | 11427.8 | 0.1307 | 0.1345 | -0.081 | 14678.8 | 14708.1 | 0.1311 | 0.1305 | 0.1991 |
| 413 | 11818.9 | 13257.4 | 0.123 | 0.1289 | 10.851 | 11289.9 | 11333.1 | 0.1441 | 0.1437 | 0.3806 |
| 414 | 13123.4 | 12911.3 | 0.1309 | 0.1257 | -1.643 | 13951 | 14204.1 | 0.1244 | 0.1287 | 1.7818 |
| 415 | 11818.8 | 15501.8 | 0.1394 | 0.1272 | 23.758 | 12845.5 | 13187 | 0.1224 | 0.1239 | 2.5903 |
| 416 | 14435.7 | 19681.7 | 0.1282 | 0.1323 | 26.654 | 10071.1 | 10687.3 | 0.1403 | 0.1398 | 5.7663 |
| 417 | 11870.4 | 11819.3 | 0.1146 | 0.119 | -0.432 | 10488 | 10551.2 | 0.1404 | 0.1395 | 0.5984 |
| 418 | 11850.1 | 18884.6 | 0.1423 | 0.1432 | 37.25 | 13779.8 | 14722.3 | 0.1148 | 0.1165 | 6.4014 |
| 419 | 12079.6 | 24736.5 | 0.1309 | 0.1325 | 51.167 | 15053.7 | 15037.4 | 0.1301 | 0.1304 | -0.109 |
| 420 | 10880.5 | 16361.9 | 0.1307 | 0.1365 | 33.501 | 11536.9 | 11988.4 | 0.1341 | 0.1326 | 3.7661 |
| 421 | 13541 | 24468.2 | 0.1129 | 0.1308 | 44.659 | 13804.9 | 14146.5 | 0.1239 | 0.125 | 2.4146 |
| 422 | 12470.1 | 14192.9 | 0.1469 | 0.1405 | 12.139 | 10976.8 | 13067.5 | 0.1416 | 0.1356 | 15.999 |
| 423 | 10822.3 | 11302.4 | 0.1409 | 0.1388 | 4.2472 | 13432.1 | 13710.5 | 0.1289 | 0.1297 | 2.0303 |
| 424 | 11720.1 | 15374.2 | 0.1199 | 0.1082 | 23.768 | 11502 | 11843.6 | 0.1255 | 0.127 | 2.8841 |
| 425 | 13962.4 | 22832.8 | 0.1206 | 0.1329 | 38.849 | 11394 | 11714.5 | 0.1362 | 0.1374 | 2.7355 |
| 426 | 14093.8 | 24106.2 | 0.1123 | 0.1322 | 41.535 | 12263.2 | 12959.4 | 0.1297 | 0.1279 | 5.3724 |
| 427 | 13454.9 | 13657 | 0.1224 | 0.1181 | 1.4798 | 13594.2 | 13638.5 | 0.1281 | 0.1286 | 0.3252 |
| 428 | 12846.2 | 13599 | 0.1245 | 0.1265 | 5.536 | 12070.5 | 12399 | 0.1371 | 0.1427 | 2.6493 |
| 429 | 11817.9 | 16178.7 | 0.1398 | 0.1282 | 26.954 | 12174.3 | 16003 | 0.1348 | 0.1288 | 23.925 |
| 430 | 12838.6 | 24199.1 | 0.1285 | 0.1313 | 46.946 | 13625.2 | 13838.3 | 0.1227 | 0.1244 | 1.5396 |
| 431 | 13100.9 | 13398.4 | 0.1241 | 0.1215 | 2.2203 | 14149.9 | 15246.3 | 0.1429 | 0.1374 | 7.1913 |
| 432 | 14010.2 | 15498.8 | 0.1234 | 0.1318 | 9.6048 | 14201.5 | 14252.1 | 0.1297 | 0.1297 | 0.3552 |
| 433 | 17064.5 | 17254.1 | 0.1289 | 0.1339 | 1.0988 | 12613 | 13407.1 | 0.141 | 0.1391 | 5.9227 |
| 434 | 12517.6 | 12400.4 | 0.1237 | 0.1245 | -0.945 | 11436.9 | 11516.5 | 0.1369 | 0.1363 | 0.6914 |
| 435 | 11599.5 | 11367.4 | 0.1197 | 0.1116 | -2.041 | 11756.5 | 11813.3 | 0.1306 | 0.1292 | 0.4806 |
| 436 | 12494.2 | 12217 | 0.125 | 0.1258 | -2.269 | 12690.7 | 12756.5 | 0.1393 | 0.1401 | 0.5156 |
| 437 | 12685.2 | 18833.7 | 0.1302 | 0.1279 | 32.646 | 14618.8 | 15116.8 | 0.1408 | 0.144 | 3.2941 |
| 438 | 12800.9 | 22480.3 | 0.1371 | 0.1299 | 43.057 | 11128.5 | 11586.7 | 0.1371 | 0.1367 | 3.9548 |
| 439 | 12219.8 | 16634.9 | 0.1457 | 0.1403 | 26.541 | 11958.5 | 11958.5 | 0.1136 | 0.1137 | 0 |
| 440 | 12106.9 | 17744.2 | 0.1209 | 0.1308 | 31.77 | 10936 | 11156.7 | 0.1329 | 0.138 | 1.9786 |
| 441 | 11635.5 | 18894.8 | 0.1408 | 0.1422 | 38.419 | 12420.8 | 12272.2 | 0.1306 | 0.1402 | -1.21 |
| 442 | 13171.1 | 18222 | 0.1338 | 0.1329 | 27.719 | 11715.8 | 12001.8 | 0.133 | 0.138 | 2.3831 |
| 443 | 12780.7 | 12809 | 0.1181 | 0.1135 | 0.2205 | 14456.1 | 14598 | 0.1249 | 0.1241 | 0.9722 |
| 444 | 12976.8 | 12963 | 0.115 | 0.1154 | -0.106 | 11376.6 | 11762.2 | 0.1452 | 0.1431 | 3.279 |
| 445 | 13807 | 12927 | 0.1316 | 0.1251 | -6.808 | 11021.6 | 11361.9 | 0.14 | 0.1358 | 2.9957 |
| 446 | 11891.9 | 17418.3 | 0.1325 | 0.1361 | 31.727 | 14827.6 | 16059.3 | 0.1344 | 0.1365 | 7.6694 |
| 447 | 12846.4 | 12901.2 | 0.1135 | 0.1131 | 0.4252 | 11951.7 | 12097.5 | 0.1309 | 0.129 | 1.205 |
| 448 | 13055.7 | 24059 | 0.1139 | 0.1314 | 45.735 | 14401.7 | 15451.9 | 0.1246 | 0.1243 | 6.7968 |
| 449 | 12877.7 | 12637.1 | 0.1121 | 0.1144 | -1.904 | 12055.1 | 12178.3 | 0.1308 | 0.1344 | 1.0121 |
| 450 | 12547.7 | 20562.8 | 0.1352 | 0.1237 | 38.979 | 14147.3 | 14273.4 | 0.1299 | 0.1333 | 0.8841 |
| 451 | 12102.6 | 18319.2 | 0.1258 | 0.1345 | 33.935 | 12792.4 | 12854.6 | 0.1145 | 0.1151 | 0.484 |
| 452 | 12268.7 | 13044.3 | 0.1107 | 0.1155 | 5.9462 | 11504.3 | 11504.3 | 0.1169 | 0.1169 | 0 |
| 453 | 12885.7 | 26707.5 | 0.1281 | 0.1304 | 51.752 | 14105.1 | 14313.6 | 0.1285 | 0.1294 | 1.4566 |
| 454 | 12837.6 | 24015 | 0.1119 | 0.1295 | 46.543 | 12510.2 | 12550.4 | 0.1352 | 0.1375 | 0.3197 |
| 455 | 13299 | 22890.4 | 0.1231 | 0.1328 | 41.901 | 11838.8 | 11885.3 | 0.1471 | 0.1442 | 0.3918 |
| 456 | 11443.3 | 12816.2 | 0.1374 | 0.1443 | 10.712 | 14950 | 15324.7 | 0.1349 | 0.1304 | 2.4453 |
| 457 | 11874.3 | 14638.3 | 0.1461 | 0.1428 | 18.882 | 14970 | 14983.3 | 0.1241 | 0.1259 | 0.0891 |
| 458 | 11782.7 | 11941.4 | 0.1137 | 0.1142 | 1.329 | 11774.2 | 11844.1 | 0.136 | 0.1374 | 0.5903 |
| 459 | 14256.8 | 22252.2 | 0.1202 | 0.1312 | 35.931 | 11217.6 | 11648.3 | 0.1358 | 0.1363 | 3.6979 |
| 460 | 10859.1 | 10753.7 | 0.1192 | 0.1122 | -0.98 | 14147.1 | 14909.5 | 0.1285 | 0.1273 | 5.1131 |
| 461 | 11453.5 | 11298.7 | 0.1344 | 0.1322 | -1.371 | 11204.3 | 11315.5 | 0.1424 | 0.1416 | 0.9821 |
| 462 | 12800 | 19724.3 | 0.1227 | 0.1286 | 35.105 | 11898.4 | 16104.7 | 0.1348 | 0.1288 | 26.119 |
| 463 | 12087.3 | 14536.2 | 0.1326 | 0.1361 | 16.847 | 12340.5 | 12553.5 | 0.1354 | 0.134 | 1.6969 |
| 464 | 13011.7 | 19177.7 | 0.1168 | 0.1231 | 32.152 | 13932.7 | 14409.1 | 0.133 | 0.1293 | 3.3062 |
| 465 | 13001.1 | 13201.8 | 0.1163 | 0.1117 | 1.5203 | 13528.8 | 11977.6 | 0.1199 | 0.1192 | -12.95 |
| 466 | 11447.6 | 11517.1 | 0.1122 | 0.1147 | 0.6034 | 13319.9 | 13602.6 | 0.1315 | 0.1319 | 2.0778 |
| 467 | 11211.1 | 19639 | 0.1392 | 0.1415 | 42.914 | 13143.4 | 13279.8 | 0.1225 | 0.1272 | 1.0271 |
| 468 | 12657.7 | 12743.3 | 0.1104 | 0.1131 | 0.6714 | 11760.5 | 11807.1 | 0.1416 | 0.1386 | 0.3944 |
| 469 | 13112.6 | 13551.2 | 0.1213 | 0.1128 | 3.2367 | 10768.2 | 11338.7 | 0.1415 | 0.1384 | 5.0313 |
| 470 | 14253.9 | 25922.7 | 0.1111 | 0.1248 | 45.014 | 13706 | 13744.1 | 0.1253 | 0.1245 | 0.277 |
| 471 | 13740.5 | 23797.4 | 0.1237 | 0.1326 | 42.261 | 11888.5 | 12023.1 | 0.1328 | 0.1307 | 1.1192 |
| 472 | 14430.2 | 16519.2 | 0.1285 | 0.1296 | 12.646 | 12455.6 | 12586.2 | 0.1388 | 0.1409 | 1.0372 |
| 473 | 14111.6 | 16050.5 | 0.128 | 0.1272 | 12.08 | 13755.8 | 13668.7 | 0.128 | 0.1318 | -0.638 |
| 474 | 12664.2 | 12601.6 | 0.1181 | 0.1132 | -0.497 | 14034.4 | 14093 | 0.1245 | 0.1245 | 0.4157 |
| 475 | 13357.8 | 13222.4 | 0.1366 | 0.1318 | -1.024 | 15618.6 | 15639.1 | 0.13 | 0.1297 | 0.1311 |
| 476 | 16278.1 | 16608.3 | 0.1301 | 0.1279 | 1.9882 | 12143.2 | 12427 | 0.1439 | 0.1447 | 2.2834 |
| 477 | 14264.2 | 16196.3 | 0.1252 | 0.1305 | 11.929 | 13270.3 | 13518.3 | 0.1255 | 0.1273 | 1.8347 |
| 478 | 12992 | 22206.5 | 0.1194 | 0.1344 | 41.495 | 13949.1 | 14271.3 | 0.1257 | 0.1264 | 2.2578 |
| 479 | 12385.5 | 17338.6 | 0.1367 | 0.1386 | 28.567 | 11674.2 | 11657.8 | 0.1363 | 0.1367 | -0.14 |
| 480 | 14422.8 | 14159.3 | 0.1361 | 0.1313 | -1.861 | 10808.6 | 12987.3 | 0.1448 | 0.1395 | 16.776 |
| 481 | 13932.4 | 15529.8 | 0.1255 | 0.1346 | 10.286 | 13048.8 | 13612.3 | 0.127 | 0.1265 | 4.1403 |
| 482 | 12550.5 | 12453.8 | 0.1254 | 0.1257 | -0.776 | 11602.8 | 12005.3 | 0.1268 | 0.128 | 3.3527 |
| 483 | 11535.4 | 11636.1 | 0.1143 | 0.1154 | 0.8655 | 11918.2 | 12152.9 | 0.1321 | 0.1337 | 1.9311 |
| 484 | 13379.9 | 13101.3 | 0.1133 | 0.117 | -2.126 | 15075.4 | 15854.2 | 0.1411 | 0.1433 | 4.9125 |
| 485 | 13770.5 | 23064.6 | 0.1179 | 0.132 | 40.296 | 13476.4 | 13505.7 | 0.1296 | 0.1292 | 0.2169 |
| 486 | 11915 | 15863.6 | 0.1442 | 0.1422 | 24.891 | 11370.9 | 11431.3 | 0.1356 | 0.1347 | 0.5285 |
| 487 | 13053 | 24595.7 | 0.1294 | 0.1309 | 46.93 | 13198.6 | 13255.5 | 0.122 | 0.1234 | 0.4299 |
| 488 | 12818.6 | 20786.7 | 0.1196 | 0.1236 | 38.333 | 12015 | 12015 | 0.1181 | 0.1181 | 0 |
| 489 | 10905.4 | 16973.9 | 0.1306 | 0.1374 | 35.752 | 12947.5 | 13398.3 | 0.13 | 0.1286 | 3.3646 |
| 490 | 13029.3 | 13422.9 | 0.1176 | 0.1143 | 2.9326 | 14015.5 | 14669.3 | 0.1354 | 0.1349 | 4.457 |
| 491 | 12452 | 13293.1 | 0.1293 | 0.1294 | 6.3275 | 10428.6 | 10437.4 | 0.1439 | 0.1425 | 0.0842 |
| 492 | 12531.1 | 12743.7 | 0.1137 | 0.1122 | 1.6685 | 11233.8 | 11137.8 | 0.1428 | 0.1378 | -0.863 |
| 493 | 14150.1 | 14262.1 | 0.1292 | 0.1202 | 0.7856 | 11982.8 | 12042.5 | 0.1416 | 0.1408 | 0.4958 |
| 494 | 16741.6 | 17450.4 | 0.1241 | 0.1212 | 4.0617 | 10516.9 | 10997.3 | 0.1388 | 0.1379 | 4.3688 |
| 495 | 12590.1 | 14266.5 | 0.1221 | 0.1291 | 11.751 | 12415.2 | 12736.1 | 0.1327 | 0.1404 | 2.5198 |
| 496 | 11612.7 | 15674.8 | 0.1375 | 0.1343 | 25.915 | 12586.3 | 12656 | 0.1339 | 0.1349 | 0.5508 |
| 497 | 12993.2 | 22960.7 | 0.1152 | 0.1334 | 43.411 | 12842.9 | 12847 | 0.1143 | 0.1142 | 0.0322 |
| 498 | 12097.4 | 17545.2 | 0.1266 | 0.1351 | 31.05 | 11002.9 | 10943.4 | 0.1362 | 0.1372 | -0.544 |
| 499 | 13031.9 | 13348.9 | 0.1305 | 0.1346 | 2.3746 | 12749.9 | 12749.9 | 0.122 | 0.1218 | 0 |
| 500 | 13573.3 | 22419.5 | 0.1206 | 0.1332 | 39.457 | 12954.8 | 13428.9 | 0.1443 | 0.1441 | 3.5308 |
| 501 | 10697 | 11579.4 | 0.132 | 0.1288 | 7.6205 | 12241.3 | 12262.8 | 0.1438 | 0.1428 | 0.1754 |
| 502 | 15007.1 | 16284.1 | 0.1281 | 0.1307 | 7.8424 | 14316.3 | 14366.9 | 0.1298 | 0.1297 | 0.3524 |
| 503 | 12011.7 | 14041.3 | 0.117 | 0.1081 | 14.455 | 11769.7 | 11832.8 | 0.1424 | 0.1416 | 0.5336 |
| 504 | 15132.4 | 16181.5 | 0.127 | 0.125 | 6.4837 | 12433.7 | 12652.7 | 0.1344 | 0.1398 | 1.7308 |
| 505 | 13000.8 | 14743.2 | 0.1155 | 0.1134 | 11.819 | 13493.5 | 14154 | 0.1418 | 0.1438 | 4.6663 |
| 506 | 10828.4 | 16298.4 | 0.139 | 0.1295 | 33.561 | 12600.3 | 12604.9 | 0.1292 | 0.1296 | 0.0368 |
| 507 | 14009.1 | 21598.2 | 0.1217 | 0.1337 | 35.138 | 15846.7 | 16620 | 0.1225 | 0.122 | 4.6529 |
| 508 | 11461.3 | 15211 | 0.1293 | 0.1321 | 24.652 | 14123.4 | 14174 | 0.1297 | 0.1296 | 0.3572 |
| 509 | 10989.4 | 15896.8 | 0.1459 | 0.1411 | 30.87 | 12071.5 | 12071.5 | 0.1149 | 0.115 | 0 |
| 510 | 12419.8 | 24031.9 | 0.1131 | 0.1302 | 48.32 | 10452 | 10747.8 | 0.1359 | 0.1389 | 2.7522 |
| 511 | 11797.5 | 15332.6 | 0.143 | 0.1408 | 23.056 | 12139 | 12256.9 | 0.1298 | 0.1336 | 0.9619 |
| 512 | 14073.4 | 16627.3 | 0.1264 | 0.1301 | 15.36 | 11776.9 | 11924.9 | 0.1325 | 0.1304 | 1.241 |
| 513 | 12888.8 | 13695.2 | 0.1368 | 0.1333 | 5.8884 | 15891.8 | 15921.1 | 0.1285 | 0.1282 | 0.184 |
| 514 | 13133.3 | 22079.4 | 0.124 | 0.1356 | 40.518 | 11910.5 | 12068 | 0.1402 | 0.1412 | 1.305 |
| 515 | 12340.1 | 12185.3 | 0.1337 | 0.131 | -1.271 | 13243.8 | 13361.8 | 0.1291 | 0.1328 | 0.8824 |
| 516 | 11545.6 | 15860 | 0.1475 | 0.1418 | 27.203 | 11411.4 | 11289.3 | 0.1364 | 0.1346 | -1.082 |
| 517 | 12813.8 | 18822.6 | 0.1232 | 0.1289 | 31.923 | 13723.4 | 13780.3 | 0.1236 | 0.1249 | 0.4135 |
| 518 | 13504.8 | 14009.5 | 0.1257 | 0.1245 | 3.6022 | 13649.7 | 15276.3 | 0.1262 | 0.1249 | 10.648 |
| 519 | 11680 | 11640 | 0.1163 | 0.1138 | -0.344 | 13694 | 13677.6 | 0.1309 | 0.1311 | -0.12 |
| 520 | 12663.7 | 12292.8 | 0.1071 | 0.1113 | -3.017 | 11637.7 | 11840.1 | 0.13 | 0.1317 | 1.71 |
| 521 | 13909.8 | 22277.2 | 0.1311 | 0.1323 | 37.56 | 11875.8 | 11929.9 | 0.1359 | 0.1371 | 0.4531 |
| 522 | 13138 | 23515.1 | 0.1147 | 0.1308 | 44.13 | 10105 | 10329.9 | 0.1384 | 0.1372 | 2.1771 |
| 523 | 14370.6 | 15654.7 | 0.1232 | 0.1331 | 8.2023 | 11843.7 | 11849.9 | 0.1316 | 0.1311 | 0.0519 |
| 524 | 11778.1 | 16168.8 | 0.1361 | 0.1363 | 27.156 | 11919.7 | 12003.7 | 0.1373 | 0.1449 | 0.6995 |
| 525 | 12140.1 | 24291.6 | 0.1298 | 0.1325 | 50.024 | 13771.5 | 13976.9 | 0.1328 | 0.1319 | 1.4692 |
| 526 | 13443.5 | 25894.2 | 0.1158 | 0.1268 | 48.083 | 11025.2 | 11144.4 | 0.133 | 0.1305 | 1.0696 |
| 527 | 14239 | 14946.8 | 0.1308 | 0.1305 | 4.7353 | 11680.7 | 11668.8 | 0.1343 | 0.1338 | -0.102 |
| 528 | 15176.8 | 15989 | 0.1284 | 0.1245 | 5.0798 | 12521.4 | 12524.8 | 0.1338 | 0.1343 | 0.0274 |
| 529 | 12096.5 | 24217.9 | 0.1316 | 0.1309 | 50.051 | 11398.1 | 11445.8 | 0.1314 | 0.1314 | 0.4167 |
| 530 | 13128.5 | 21060.9 | 0.1187 | 0.1236 | 37.664 | 13875.7 | 14144 | 0.1312 | 0.1298 | 1.8971 |
| 531 | 14041.6 | 14629.5 | 0.1283 | 0.129 | 4.019 | 11841.9 | 12370.9 | 0.1366 | 0.1368 | 4.2767 |
| 532 | 15346.5 | 18514.9 | 0.1309 | 0.1332 | 17.113 | 11506.9 | 16266.9 | 0.1385 | 0.1294 | 29.262 |
| 533 | 11505.2 | 20181.2 | 0.1356 | 0.1367 | 42.991 | 15874.6 | 15974.2 | 0.1285 | 0.1255 | 0.6234 |
| 534 | 12657.9 | 12375.2 | 0.1171 | 0.1095 | -2.284 | 14687.4 | 14985.5 | 0.1279 | 0.1252 | 1.9891 |
| 535 | 10554 | 14822.3 | 0.129 | 0.127 | 28.796 | 15630.9 | 15967.8 | 0.1306 | 0.1312 | 2.1094 |
| 536 | 12802.8 | 17832.9 | 0.1196 | 0.1314 | 28.207 | 13221 | 13249.8 | 0.1351 | 0.1341 | 0.2173 |
| 537 | 12866.3 | 23025.1 | 0.1202 | 0.1335 | 44.12 | 11955.8 | 12189.5 | 0.1321 | 0.1328 | 1.917 |
| 538 | 11953.6 | 15718.9 | 0.1268 | 0.1313 | 23.954 | 13167.2 | 13202 | 0.1244 | 0.1274 | 0.2633 |
| 539 | 12821.5 | 14160.1 | 0.1277 | 0.133 | 9.4534 | 10563.2 | 10873.2 | 0.1355 | 0.136 | 2.8506 |
| 540 | 12437.2 | 24448.2 | 0.1141 | 0.1268 | 49.129 | 13634.3 | 14265.3 | 0.1356 | 0.1375 | 4.4231 |
| 541 | 13878.6 | 16190.9 | 0.1276 | 0.1244 | 14.282 | 12085.4 | 12121.2 | 0.1331 | 0.1391 | 0.2947 |
| 542 | 12341.5 | 12843.7 | 0.1191 | 0.1139 | 3.9096 | 12313.7 | 12502.8 | 0.134 | 0.1421 | 1.5126 |
| 543 | 13294.2 | 21276.4 | 0.1262 | 0.1335 | 37.517 | 10465.9 | 12105.5 | 0.1403 | 0.1343 | 13.544 |
| 544 | 10545.4 | 18095.6 | 0.142 | 0.142 | 41.724 | 13450 | 13954.9 | 0.1167 | 0.1162 | 3.6178 |
| 545 | 12343.5 | 13899.9 | 0.1216 | 0.1288 | 11.197 | 11629.2 | 11670.6 | 0.1288 | 0.1291 | 0.3549 |
| 546 | 12879.9 | 20726 | 0.1208 | 0.1227 | 37.856 | 14711.1 | 15182.4 | 0.1307 | 0.1288 | 3.1042 |
| 547 | 13984.6 | 18904.9 | 0.1282 | 0.1311 | 26.026 | 12218.7 | 12218.7 | 0.1149 | 0.115 | 0 |
| 548 | 12412.5 | 16642.4 | 0.1308 | 0.1337 | 25.416 | 11551.2 | 11555.3 | 0.1134 | 0.1132 | 0.0358 |
| 549 | 15960.4 | 17483.8 | 0.1224 | 0.1244 | 8.7134 | 12437.6 | 12645.4 | 0.1316 | 0.1325 | 1.6431 |
| 550 | 11019.1 | 11134.3 | 0.1186 | 0.1212 | 1.0353 | 13153.8 | 13300.7 | 0.1161 | 0.1178 | 1.1042 |
| 551 | 11721 | 14998 | 0.1254 | 0.1314 | 21.85 | 13716.6 | 14359 | 0.1416 | 0.1419 | 4.4738 |
| 552 | 11540.8 | 11533.3 | 0.1113 | 0.1186 | -0.066 | 14905.3 | 16042.7 | 0.1408 | 0.1375 | 7.0899 |
| 553 | 11706.3 | 14895.6 | 0.1287 | 0.1314 | 21.411 | 14757 | 15638.8 | 0.1337 | 0.134 | 5.6383 |
| 554 | 15815.5 | 16854.3 | 0.1255 | 0.1252 | 6.1633 | 13938.5 | 13995.5 | 0.1237 | 0.125 | 0.4072 |
| 555 | 13151.4 | 13759.8 | 0.1305 | 0.123 | 4.4213 | 11206.1 | 11465.7 | 0.1327 | 0.1346 | 2.2636 |
| 556 | 13853 | 13946.8 | 0.1292 | 0.1204 | 0.6723 | 13398.2 | 14047 | 0.1266 | 0.1249 | 4.6189 |
| 557 | 12813.3 | 22901.6 | 0.1252 | 0.1311 | 44.051 | 11224.7 | 11262.7 | 0.1383 | 0.136 | 0.3373 |
| 558 | 12317.1 | 12321 | 0.1104 | 0.1106 | 0.0319 | 14341.1 | 19416.9 | 0.1237 | 0.1215 | 26.141 |
| 559 | 11331.2 | 12232.4 | 0.1366 | 0.1248 | 7.3673 | 11111.5 | 11813.6 | 0.1368 | 0.1344 | 5.943 |
| 560 | 11984.8 | 12611.7 | 0.137 | 0.1254 | 4.971 | 12789.2 | 12895.4 | 0.1144 | 0.1158 | 0.8232 |
| 561 | 14097.6 | 24964.4 | 0.1124 | 0.1282 | 43.529 | 11078.6 | 15697.6 | 0.1385 | 0.1297 | 29.425 |
| 562 | 12854.6 | 13356.7 | 0.1121 | 0.116 | 3.7597 | 11239.9 | 11273.5 | 0.1351 | 0.1342 | 0.2984 |
| 563 | 12570.1 | 21888.8 | 0.1362 | 0.1286 | 42.573 | 12332.5 | 12336.6 | 0.1156 | 0.1157 | 0.0336 |
| 564 | 16682.6 | 17183.8 | 0.1225 | 0.1282 | 2.9163 | 12071.6 | 12150.3 | 0.1241 | 0.1265 | 0.6477 |
| 565 | 13003.6 | 22941.9 | 0.1227 | 0.13 | 43.319 | 10703 | 11564.8 | 0.1391 | 0.1382 | 7.4518 |
| 566 | 11210.3 | 17898.1 | 0.1449 | 0.1424 | 37.366 | 12014.2 | 12077.4 | 0.1384 | 0.1376 | 0.5228 |
| 567 | 14044.7 | 22594.5 | 0.1209 | 0.1325 | 37.84 | 12671.4 | 12740.3 | 0.1268 | 0.1307 | 0.5409 |
| 568 | 12610.4 | 21032.3 | 0.1276 | 0.1308 | 40.043 | 12116 | 12424.5 | 0.1326 | 0.131 | 2.4832 |
| 569 | 12490.2 | 13252.4 | 0.1114 | 0.1095 | 5.7509 | 11903.1 | 12009.2 | 0.1408 | 0.1374 | 0.8832 |
| 570 | 11148.6 | 10966.6 | 0.1323 | 0.1334 | -1.659 | 14220.1 | 14211.3 | 0.1284 | 0.1281 | -0.062 |
| 571 | 10957.4 | 15212.3 | 0.137 | 0.1362 | 27.97 | 12402.7 | 12537.9 | 0.1255 | 0.1258 | 1.0782 |
| 572 | 14881.4 | 15528.6 | 0.1242 | 0.1223 | 4.1675 | 12353.2 | 12369.1 | 0.1316 | 0.1324 | 0.1282 |
| 573 | 11916.4 | 15561.4 | 0.1403 | 0.1276 | 23.423 | 10421.1 | 11040.6 | 0.1384 | 0.1355 | 5.6109 |
| 574 | 12531.4 | 16572 | 0.1438 | 0.1394 | 24.382 | 12070 | 12187.9 | 0.1268 | 0.1309 | 0.9674 |
| 575 | 13548.7 | 15167.3 | 0.1213 | 0.1323 | 10.671 | 12283.7 | 12346.8 | 0.1365 | 0.1358 | 0.5114 |
| 576 | 10661.1 | 16365.8 | 0.1425 | 0.1295 | 34.857 | 11761.3 | 11902.6 | 0.1375 | 0.1356 | 1.1874 |
| 577 | 11501.9 | 16730.3 | 0.1267 | 0.1322 | 31.251 | 11376.1 | 12337.9 | 0.14 | 0.1348 | 7.7954 |
| 578 | 13124.4 | 13193 | 0.1289 | 0.1302 | 0.5205 | 14211.3 | 14466.6 | 0.1151 | 0.1147 | 1.7651 |
| 579 | 12801.2 | 22625.8 | 0.1371 | 0.1311 | 43.422 | 11560.4 | 11635 | 0.1439 | 0.1451 | 0.6408 |
| 580 | 11506.2 | 11739.9 | 0.1298 | 0.1337 | 1.9912 | 11469 | 11743.6 | 0.1362 | 0.1364 | 2.3383 |
| 581 | 11290.5 | 17541.7 | 0.141 | 0.1392 | 35.636 | 12291.6 | 12271.4 | 0.136 | 0.1347 | -0.165 |
| 582 | 16327.6 | 19175.6 | 0.126 | 0.1323 | 14.852 | 11804.3 | 15370 | 0.1388 | 0.1295 | 23.199 |
| 583 | 11872.5 | 18217.5 | 0.1436 | 0.1425 | 34.829 | 11235.1 | 11575.2 | 0.1341 | 0.1368 | 2.9387 |
| 584 | 12080.5 | 14024 | 0.1348 | 0.1431 | 13.859 | 11760.1 | 11997.4 | 0.1354 | 0.1363 | 1.9781 |
| 585 | 12906.6 | 13187.5 | 0.1421 | 0.1402 | 2.1302 | 10790.4 | 12898.5 | 0.1374 | 0.1328 | 16.344 |
| 586 | 12052 | 19295.3 | 0.1265 | 0.1334 | 37.539 | 12116.7 | 12252.3 | 0.135 | 0.1404 | 1.1064 |
| 587 | 12572.7 | 16807.3 | 0.1363 | 0.1396 | 25.195 | 11766.4 | 11831 | 0.141 | 0.1413 | 0.5464 |
| 588 | 11946.8 | 27049.8 | 0.1197 | 0.1237 | 55.834 | 12176.5 | 12171.9 | 0.1421 | 0.1395 | -0.038 |
| 589 | 12828.3 | 14071.1 | 0.1129 | 0.1121 | 8.8326 | 13237.5 | 13891.3 | 0.1356 | 0.1349 | 4.7066 |
| 590 | 11894.9 | 17559 | 0.1225 | 0.1302 | 32.258 | 11714 | 11899 | 0.1277 | 0.1283 | 1.5556 |
| 591 | 14243.2 | 16779.8 | 0.1315 | 0.1292 | 15.117 | 13476.7 | 13662.6 | 0.1273 | 0.1285 | 1.3606 |
| 592 | 13282.3 | 27800.6 | 0.1283 | 0.1264 | 52.223 | 12849 | 13190.6 | 0.1219 | 0.1233 | 2.5896 |
| 593 | 11745.9 | 14862.7 | 0.1441 | 0.1393 | 20.97 | 10614.2 | 10910.3 | 0.1448 | 0.1442 | 2.714 |
| 594 | 11314.6 | 17709.2 | 0.1444 | 0.1413 | 36.109 | 13182.5 | 13203 | 0.1277 | 0.1273 | 0.1553 |
| 595 | 12412.3 | 14145.3 | 0.1188 | 0.1281 | 12.251 | 12670.3 | 12674.4 | 0.1139 | 0.1137 | 0.0327 |
| 596 | 12146.3 | 11968.5 | 0.1348 | 0.1292 | -1.485 | 14065.5 | 12806.4 | 0.1243 | 0.1199 | -9.832 |
| 597 | 12939 | 24458 | 0.1123 | 0.1285 | 47.097 | 11983.4 | 12304.3 | 0.145 | 0.1443 | 2.6082 |
| 598 | 15517.7 | 19080.5 | 0.1254 | 0.1306 | 18.672 | 12315.3 | 12365.4 | 0.135 | 0.1419 | 0.4053 |
| 599 | 11601.4 | 11551.3 | 0.1293 | 0.1205 | -0.434 | 11882.4 | 12650.8 | 0.1286 | 0.1285 | 6.0743 |
| 600 | 10816.4 | 17821.7 | 0.136 | 0.1364 | 39.307 | 13381.3 | 13386 | 0.1283 | 0.1286 | 0.0347 |
| 601 | 13303.6 | 13979.5 | 0.1333 | 0.1318 | 4.8346 | 11993.7 | 11987.9 | 0.1423 | 0.1424 | -0.049 |
| 602 | 13153.5 | 13725.3 | 0.1305 | 0.124 | 4.1659 | 12165 | 12140.5 | 0.1293 | 0.1287 | -0.201 |
| 603 | 13546.5 | 13369.7 | 0.1364 | 0.1317 | -1.322 | 12075.1 | 12959.8 | 0.1379 | 0.1379 | 6.8264 |
| 604 | 11598.1 | 15141 | 0.1305 | 0.1281 | 23.399 | 11517.1 | 11533 | 0.1339 | 0.1348 | 0.1375 |
| 605 | 11459 | 16823.7 | 0.1305 | 0.1369 | 31.887 | 11458.8 | 11539.4 | 0.1321 | 0.1362 | 0.6987 |
| 606 | 11931 | 17868.6 | 0.1376 | 0.1272 | 33.229 | 12854.7 | 12989.9 | 0.1242 | 0.1246 | 1.0407 |
| 607 | 12588.5 | 23493.8 | 0.134 | 0.1319 | 46.418 | 11375.9 | 11469.5 | 0.1385 | 0.1383 | 0.8157 |
| 608 | 17500.9 | 17412 | 0.1235 | 0.1259 | -0.51 | 11964.6 | 11967.1 | 0.1374 | 0.1354 | 0.021 |
| 609 | 12933.9 | 22258.4 | 0.1204 | 0.1332 | 41.892 | 10688.8 | 11034.8 | 0.1409 | 0.1391 | 3.1357 |
| 610 | 12718.1 | 12790.7 | 0.1117 | 0.115 | 0.5679 | 10913.2 | 14662 | 0.1409 | 0.1305 | 25.568 |
| 611 | 11153.8 | 11241.3 | 0.1142 | 0.1167 | 0.7783 | 13177.6 | 13312.8 | 0.1261 | 0.1261 | 1.0154 |
| 612 | 14728.3 | 15274 | 0.1209 | 0.125 | 3.5724 | 13223.6 | 13223.6 | 0.1161 | 0.1161 | 0 |
| 613 | 12298.3 | 19217.2 | 0.1348 | 0.1399 | 36.004 | 14041.7 | 14923.5 | 0.1335 | 0.1336 | 5.9086 |
| 614 | 13205.2 | 12208.1 | 0.1325 | 0.1284 | -8.168 | 12771.8 | 13009.8 | 0.1335 | 0.1393 | 1.8293 |
| 615 | 12097.6 | 19804.6 | 0.1355 | 0.1403 | 38.916 | 11951.9 | 12542.9 | 0.1327 | 0.1328 | 4.7119 |
| 616 | 12181.1 | 16095.3 | 0.146 | 0.14 | 24.319 | 15199.6 | 15220.1 | 0.1288 | 0.1283 | 0.1347 |
| 617 | 13910.1 | 15848.8 | 0.1244 | 0.1329 | 12.233 | 16087.5 | 16917.7 | 0.1243 | 0.123 | 4.9071 |
| 618 | 11427.8 | 11347.4 | 0.1164 | 0.1148 | -0.708 | 13089.4 | 14188.8 | 0.1442 | 0.1382 | 7.748 |
| 619 | 12952.5 | 13535.8 | 0.1342 | 0.1324 | 4.3096 | 15862.8 | 15892.1 | 0.1287 | 0.1282 | 0.1843 |
| 620 | 11961.9 | 19073.2 | 0.1283 | 0.1289 | 37.284 | 15097.9 | 15237.3 | 0.1297 | 0.1299 | 0.9147 |
| 621 | 11415.8 | 11531.6 | 0.1288 | 0.1268 | 1.004 | 13868.6 | 13938 | 0.13 | 0.1285 | 0.498 |
| 622 | 16486.4 | 17444 | 0.1246 | 0.1209 | 5.4894 | 13831.5 | 13970.9 | 0.1298 | 0.1293 | 0.9976 |
| 623 | 10746.8 | 11666.1 | 0.1369 | 0.1397 | 7.8799 | 12962 | 13274 | 0.1321 | 0.1344 | 2.3511 |
| 624 | 11880 | 11570 | 0.1097 | 0.1151 | -2.679 | 12184.1 | 13213.6 | 0.1271 | 0.1265 | 7.7914 |
| 625 | 11646.4 | 15870.3 | 0.1447 | 0.1408 | 26.615 | 11986.5 | 12231.2 | 0.1357 | 0.1312 | 2.0002 |
| 626 | 13670.8 | 16197.9 | 0.1292 | 0.1302 | 15.601 | 11908.7 | 12323.6 | 0.1361 | 0.1411 | 3.3666 |
| 627 | 14764.5 | 15634.7 | 0.13 | 0.1276 | 5.5659 | 11622 | 11624.5 | 0.135 | 0.1333 | 0.0216 |
| 628 | 12468.6 | 22383.1 | 0.1213 | 0.1335 | 44.294 | 10107 | 10661.8 | 0.1433 | 0.1442 | 5.2036 |
| 629 | 13259.3 | 20072.6 | 0.12 | 0.1228 | 33.944 | 12255.1 | 12434.3 | 0.138 | 0.1381 | 1.4412 |
| 630 | 13491.4 | 14234.8 | 0.1283 | 0.1254 | 5.222 | 15796.4 | 15787.6 | 0.1294 | 0.1294 | -0.056 |
| 631 | 12282.4 | 20641.6 | 0.1231 | 0.1238 | 40.497 | 10914.5 | 11138.1 | 0.1361 | 0.1367 | 2.0082 |
| 632 | 14345 | 18351.4 | 0.1316 | 0.1334 | 21.831 | 12618.9 | 12657 | 0.1233 | 0.1224 | 0.3008 |
| 633 | 15355.8 | 21112.2 | 0.1227 | 0.1306 | 27.266 | 14611.3 | 15372.6 | 0.1375 | 0.1391 | 4.9521 |
| 634 | 12644.2 | 19477.9 | 0.1333 | 0.1293 | 35.085 | 12156.8 | 12160.9 | 0.1125 | 0.1124 | 0.0341 |
| 635 | 13970.1 | 19662.1 | 0.1293 | 0.1225 | 28.949 | 13428.5 | 13432.6 | 0.1171 | 0.117 | 0.0308 |
| 636 | 12195.1 | 13422 | 0.1417 | 0.1466 | 9.141 | 13078.6 | 13194.5 | 0.1292 | 0.1307 | 0.8781 |
| 637 | 11678.8 | 11531.3 | 0.1197 | 0.1154 | -1.279 | 13486.1 | 13442.4 | 0.1295 | 0.1322 | -0.325 |
| 638 | 12885 | 24375.8 | 0.113 | 0.1287 | 47.14 | 11051.7 | 11021.5 | 0.143 | 0.1425 | -0.274 |
| 639 | 11803 | 11639.5 | 0.133 | 0.1342 | -1.405 | 13584 | 14238.1 | 0.1402 | 0.1406 | 4.5941 |
| 640 | 12611.7 | 12382.3 | 0.1085 | 0.1172 | -1.853 | 12822.6 | 13051.2 | 0.1267 | 0.1258 | 1.751 |
| 641 | 12795.6 | 22515.5 | 0.1192 | 0.134 | 43.17 | 14212.9 | 14196.5 | 0.1328 | 0.1332 | -0.115 |
| 642 | 13983 | 14633.1 | 0.1288 | 0.1282 | 4.4428 | 13361.9 | 13544.8 | 0.1273 | 0.1302 | 1.3508 |
| 643 | 10896 | 14771.9 | 0.1322 | 0.1283 | 26.239 | 12719.5 | 13211.9 | 0.1431 | 0.1425 | 3.7271 |
| 644 | 16839.1 | 17625.6 | 0.1244 | 0.1191 | 4.462 | 13904.6 | 13955.2 | 0.1297 | 0.1297 | 0.3628 |
| 645 | 10557.4 | 17412.2 | 0.1364 | 0.1307 | 39.368 | 12207.6 | 12305.8 | 0.1404 | 0.1387 | 0.7987 |
| 646 | 13365.7 | 13193.7 | 0.1349 | 0.13 | -1.303 | 12930.2 | 13627.8 | 0.132 | 0.1313 | 5.1194 |
| 647 | 12405.4 | 13341.8 | 0.1113 | 0.115 | 7.0182 | 12725.3 | 13298.5 | 0.1462 | 0.1454 | 4.3104 |
| 648 | 14028.7 | 23729.5 | 0.1159 | 0.1342 | 40.881 | 13289.3 | 13384.7 | 0.1262 | 0.1245 | 0.7131 |
| 649 | 14741.7 | 14680.2 | 0.1218 | 0.1161 | -0.419 | 13576.8 | 13552.4 | 0.1311 | 0.1306 | -0.18 |
| 650 | 13841 | 24937.9 | 0.1239 | 0.1339 | 44.498 | 12721.5 | 13095.1 | 0.1299 | 0.1298 | 2.8529 |
| 651 | 12170.3 | 15621.7 | 0.146 | 0.1416 | 22.094 | 12595.3 | 12681 | 0.1332 | 0.1326 | 0.6757 |
| 652 | 12162.4 | 12273.1 | 0.1358 | 0.1369 | 0.9021 | 13356.9 | 13407.6 | 0.1298 | 0.1298 | 0.3776 |
| 653 | 13379.4 | 21546.6 | 0.1375 | 0.1299 | 37.905 | 13370.3 | 13739.6 | 0.1252 | 0.1252 | 2.6883 |
| 654 | 12975.9 | 12540.9 | 0.1116 | 0.1143 | -3.469 | 12165.4 | 12222.3 | 0.1248 | 0.1263 | 0.4662 |
| 655 | 12204.3 | 18153.4 | 0.1423 | 0.1412 | 32.771 | 13499.8 | 13728.3 | 0.1247 | 0.1238 | 1.6646 |
| 656 | 12359.2 | 12812.6 | 0.1237 | 0.1169 | 3.5392 | 14019.7 | 13975.8 | 0.1264 | 0.1256 | -0.314 |
| 657 | 16006 | 16596.5 | 0.1238 | 0.1214 | 3.558 | 14018.5 | 14206 | 0.1278 | 0.1243 | 1.3198 |
| 658 | 13642.6 | 20502.2 | 0.1313 | 0.1253 | 33.458 | 10708.1 | 10771.3 | 0.142 | 0.1411 | 0.5862 |
| 659 | 11416.4 | 19661.4 | 0.1402 | 0.1406 | 41.935 | 12978.2 | 12982.8 | 0.1281 | 0.1284 | 0.0358 |
| 660 | 14234.6 | 14906.1 | 0.1226 | 0.1131 | 4.5046 | 11830.8 | 11830.8 | 0.1153 | 0.1154 | 0 |
| 661 | 12831.5 | 21892.3 | 0.1223 | 0.1236 | 41.388 | 11398.4 | 11845.9 | 0.1283 | 0.1296 | 3.7782 |
| 662 | 12861.9 | 13354.1 | 0.1292 | 0.1275 | 3.6859 | 11495.8 | 11809.2 | 0.1354 | 0.1339 | 2.6537 |
| 663 | 11959.6 | 16441.4 | 0.1301 | 0.1307 | 27.259 | 12969.4 | 13103 | 0.1319 | 0.1318 | 1.019 |
| 664 | 12740.3 | 19838 | 0.1235 | 0.1225 | 35.778 | 11305.9 | 15618.5 | 0.1405 | 0.1336 | 27.612 |
| 665 | 13510.7 | 26375.3 | 0.1247 | 0.1233 | 48.775 | 13907.6 | 14252.2 | 0.1361 | 0.1361 | 2.4182 |
| 666 | 12452.4 | 12456.3 | 0.1114 | 0.1116 | 0.0316 | 11369.1 | 11838.8 | 0.1388 | 0.1324 | 3.9681 |
| 667 | 13380 | 22679.9 | 0.1192 | 0.1335 | 41.005 | 14628.3 | 15446.3 | 0.1284 | 0.1271 | 5.2957 |
| 668 | 13034.9 | 23494 | 0.1155 | 0.1315 | 44.518 | 11381.1 | 11713 | 0.1348 | 0.1372 | 2.8334 |
| 669 | 12060.4 | 12247.3 | 0.1147 | 0.1123 | 1.5258 | 11259.8 | 11609 | 0.1362 | 0.1322 | 3.0083 |
| 670 | 11204.1 | 19899.3 | 0.1383 | 0.1418 | 43.696 | 12911.3 | 13057.4 | 0.1268 | 0.1251 | 1.1186 |
| 671 | 12822.4 | 12795.6 | 0.1106 | 0.1116 | -0.209 | 11203.9 | 11183.7 | 0.1409 | 0.1395 | -0.181 |
| 672 | 12833.7 | 25177.5 | 0.1281 | 0.1308 | 49.027 | 13332.1 | 13488 | 0.1342 | 0.1319 | 1.1562 |
| 673 | 12353.6 | 12275.6 | 0.1345 | 0.1294 | -0.635 | 12223.9 | 12223.9 | 0.1186 | 0.1186 | 0 |
| 674 | 12145.3 | 19114.2 | 0.1408 | 0.1401 | 36.459 | 11397.9 | 11706.4 | 0.1337 | 0.1321 | 2.6356 |
| 675 | 12817.4 | 14896.1 | 0.1218 | 0.1121 | 13.955 | 12660.1 | 12672 | 0.1289 | 0.1332 | 0.0934 |
| 676 | 11711.4 | 12125.7 | 0.1307 | 0.1336 | 3.4167 | 13460.1 | 13435.7 | 0.1322 | 0.1314 | -0.182 |
| 677 | 15050 | 15018.1 | 0.1342 | 0.13 | -0.213 | 12460.5 | 12663.8 | 0.1413 | 0.1435 | 1.605 |
| 678 | 12844.8 | 13719 | 0.1135 | 0.1149 | 6.3724 | 14006.4 | 13915.6 | 0.1289 | 0.1303 | -0.652 |
| 679 | 14387.8 | 17218.3 | 0.1296 | 0.131 | 16.439 | 11655.2 | 11643.4 | 0.1303 | 0.1345 | -0.101 |
| 680 | 13319.5 | 13182.4 | 0.1341 | 0.1295 | -1.04 | 16363 | 16488.2 | 0.1277 | 0.1277 | 0.7595 |
| 681 | 13170.5 | 14133.4 | 0.1255 | 0.1248 | 6.8126 | 11509.2 | 11920 | 0.1268 | 0.1275 | 3.4462 |
| 682 | 16894.3 | 16999.9 | 0.1284 | 0.1338 | 0.6215 | 10889.5 | 10853.6 | 0.1364 | 0.1358 | -0.332 |
| 683 | 14076.5 | 16381.1 | 0.1279 | 0.1318 | 14.068 | 12088.7 | 12228.4 | 0.1395 | 0.1388 | 1.1425 |
| 684 | 12556.6 | 13091.5 | 0.1282 | 0.1278 | 4.0855 | 14620.5 | 15546.1 | 0.1297 | 0.1301 | 5.954 |
| 685 | 12015.4 | 14836.1 | 0.1192 | 0.1311 | 19.013 | 11002.9 | 11188.1 | 0.1424 | 0.1433 | 1.6552 |
| 686 | 12945 | 12666.4 | 0.1125 | 0.1164 | -2.199 | 12730.9 | 12788.1 | 0.1335 | 0.1332 | 0.4472 |
| 687 | 12533.3 | 12657.1 | 0.1114 | 0.1146 | 0.9785 | 14191 | 14221.9 | 0.1233 | 0.1242 | 0.2174 |
| 688 | 12608.4 | 15750.6 | 0.1186 | 0.1329 | 19.949 | 12763.6 | 13019.2 | 0.1398 | 0.1398 | 1.9639 |
| 689 | 12573.8 | 19271.5 | 0.1245 | 0.1336 | 34.754 | 13272.9 | 13329.9 | 0.1213 | 0.1227 | 0.4275 |
| 690 | 10453.4 | 18891.6 | 0.1399 | 0.1419 | 44.666 | 11320.6 | 11582.6 | 0.1352 | 0.1418 | 2.2617 |
| 691 | 11597.9 | 11847.4 | 0.1312 | 0.1293 | 2.1059 | 16448.8 | 16432.4 | 0.1316 | 0.1319 | -0.1 |
| 692 | 13529.4 | 14580.7 | 0.1227 | 0.1287 | 7.2104 | 11851.1 | 11652.5 | 0.1315 | 0.1304 | -1.704 |
| 693 | 13011.3 | 21601.6 | 0.1308 | 0.1265 | 39.767 | 11286.2 | 11457.2 | 0.1325 | 0.1298 | 1.4929 |
| 694 | 13741.5 | 20987.7 | 0.1259 | 0.1327 | 34.526 | 12134.7 | 12389.4 | 0.1334 | 0.1327 | 2.0563 |
| 695 | 11055.5 | 17485.8 | 0.1346 | 0.1312 | 36.774 | 10401.3 | 10622 | 0.1355 | 0.1379 | 2.0782 |
| 696 | 11656.5 | 18000.9 | 0.1276 | 0.1363 | 35.245 | 12536.6 | 12527.9 | 0.1306 | 0.1303 | -0.07 |
| 697 | 15351.7 | 16274.9 | 0.128 | 0.1223 | 5.6729 | 12444.4 | 12507.9 | 0.1385 | 0.1391 | 0.5081 |
| 698 | 12424.2 | 18072.3 | 0.1328 | 0.1263 | 31.253 | 11380.8 | 11764.4 | 0.132 | 0.1311 | 3.2606 |
| 699 | 13162 | 25504.2 | 0.123 | 0.1314 | 48.393 | 13289 | 13264.6 | 0.1319 | 0.1312 | -0.184 |
| 700 | 11820.4 | 18832.9 | 0.1344 | 0.1397 | 37.235 | 14247.5 | 14418.4 | 0.1246 | 0.1244 | 1.1854 |
| 701 | 11657.6 | 13602.8 | 0.121 | 0.1137 | 14.3 | 12830.9 | 13036.5 | 0.1348 | 0.1351 | 1.5766 |
| 702 | 12134.3 | 15044.5 | 0.1464 | 0.1422 | 19.344 | 11908.3 | 11933.9 | 0.1403 | 0.1395 | 0.2149 |
| 703 | 13016.9 | 15533.8 | 0.1232 | 0.1292 | 16.203 | 10641.1 | 11361.5 | 0.1439 | 0.1416 | 6.3412 |
| 704 | 12634.4 | 13248.3 | 0.1342 | 0.1326 | 4.6334 | 12962.7 | 13608.6 | 0.1388 | 0.1406 | 4.7457 |
| 705 | 12753.6 | 12632.4 | 0.1191 | 0.1131 | -0.96 | 12624.5 | 12688.5 | 0.1458 | 0.1456 | 0.5039 |
| 706 | 12136.9 | 17915.7 | 0.1257 | 0.1343 | 32.256 | 13171.5 | 13233.7 | 0.1155 | 0.1167 | 0.4702 |
| 707 | 12901 | 13260.9 | 0.1267 | 0.125 | 2.7135 | 11063 | 11211.6 | 0.1335 | 0.1368 | 1.3255 |
| 708 | 13303.6 | 15366.9 | 0.1235 | 0.1289 | 13.427 | 11364 | 11323.8 | 0.137 | 0.1356 | -0.355 |
| 709 | 11248.2 | 11289.1 | 0.1105 | 0.1125 | 0.3625 | 11177.4 | 11718.4 | 0.1405 | 0.1366 | 4.6166 |
| 710 | 12378.8 | 13781.7 | 0.125 | 0.1317 | 10.179 | 11720.2 | 12217.1 | 0.1348 | 0.1356 | 4.0675 |
| 711 | 12183.4 | 18833.6 | 0.1304 | 0.1267 | 35.31 | 12261.2 | 12547.7 | 0.1267 | 0.1263 | 2.2834 |
| 712 | 11316.6 | 20418.1 | 0.1328 | 0.1403 | 44.576 | 11411.7 | 11548.4 | 0.1407 | 0.1429 | 1.1836 |
| 713 | 12251.1 | 17917.5 | 0.132 | 0.1295 | 31.625 | 15896.4 | 16082.6 | 0.129 | 0.1312 | 1.158 |
| 714 | 14742.3 | 20286.9 | 0.122 | 0.1329 | 27.331 | 11814.3 | 11954 | 0.1392 | 0.1383 | 1.1687 |
| 715 | 13030.5 | 12864.4 | 0.1313 | 0.1257 | -1.292 | 12446.1 | 12811.4 | 0.1404 | 0.1411 | 2.8515 |
| 716 | 12861.1 | 22939.7 | 0.1348 | 0.1266 | 43.935 | 11524.6 | 11825.7 | 0.1338 | 0.1337 | 2.5467 |
| 717 | 12455.7 | 14921.8 | 0.1142 | 0.1286 | 16.527 | 12656.3 | 12782.6 | 0.1119 | 0.1131 | 0.9885 |
| 718 | 12776.6 | 15583 | 0.1234 | 0.1303 | 18.009 | 11955.2 | 12012.4 | 0.1327 | 0.1325 | 0.4761 |
| 719 | 14424.6 | 22159.1 | 0.1194 | 0.131 | 34.904 | 15604.8 | 15625.3 | 0.1268 | 0.1264 | 0.1312 |
| 720 | 12633.3 | 21433.2 | 0.1352 | 0.1379 | 41.057 | 12249.2 | 12424.1 | 0.1313 | 0.1295 | 1.4077 |
| 721 | 11986.6 | 19546.7 | 0.1317 | 0.127 | 38.677 | 14387.9 | 15269.7 | 0.1355 | 0.136 | 5.7746 |
| 722 | 12991.5 | 14658.6 | 0.1199 | 0.114 | 11.373 | 14454.8 | 14492.8 | 0.1207 | 0.12 | 0.2627 |
| 723 | 11496 | 11345.7 | 0.141 | 0.1301 | -1.325 | 13637.3 | 13694.3 | 0.1229 | 0.1242 | 0.4161 |
| 724 | 11086.5 | 10945.6 | 0.12 | 0.1164 | -1.287 | 14177.9 | 14297.5 | 0.1246 | 0.1248 | 0.8364 |
| 725 | 11908 | 11534.2 | 0.1183 | 0.1119 | -3.242 | 11893.7 | 12028.9 | 0.1255 | 0.1259 | 1.1238 |
| 726 | 10604.7 | 10669.5 | 0.1401 | 0.1396 | 0.6067 | 13124.7 | 13259.9 | 0.1257 | 0.126 | 1.0195 |
| 727 | 11545.4 | 11327.4 | 0.1367 | 0.1318 | -1.925 | 11344 | 11841.4 | 0.1359 | 0.1324 | 4.2008 |
| 728 | 14614.1 | 16568.3 | 0.1281 | 0.1292 | 11.795 | 12272.4 | 12702.4 | 0.1314 | 0.131 | 3.3852 |
| 729 | 13679.3 | 21733.2 | 0.1201 | 0.1333 | 37.058 | 10233.5 | 10553.2 | 0.1342 | 0.1346 | 3.0298 |
| 730 | 12460.5 | 12339.7 | 0.1427 | 0.1271 | -0.979 | 12029.2 | 12048.1 | 0.1392 | 0.141 | 0.1569 |
| 731 | 12722.9 | 14355.3 | 0.1478 | 0.1429 | 11.371 | 12947.2 | 13141.1 | 0.1341 | 0.1308 | 1.4758 |
| 732 | 13120.9 | 21816.7 | 0.1356 | 0.1302 | 39.859 | 11541.9 | 11604.8 | 0.1421 | 0.1414 | 0.5423 |
| 733 | 12956.3 | 24650.4 | 0.1143 | 0.1265 | 47.44 | 13634.2 | 14814.2 | 0.125 | 0.1244 | 7.965 |
| 734 | 13063.7 | 13897.9 | 0.1296 | 0.1294 | 6.0022 | 10474.1 | 10791.9 | 0.1389 | 0.1415 | 2.945 |
| 735 | 12755.6 | 18648.1 | 0.1389 | 0.1251 | 31.598 | 11325.2 | 13330.1 | 0.139 | 0.137 | 15.04 |
| 736 | 12414.7 | 20084.4 | 0.1351 | 0.142 | 38.188 | 16142.4 | 16885.4 | 0.1344 | 0.1358 | 4.4001 |
| 737 | 14170.2 | 16229.1 | 0.1269 | 0.1324 | 12.686 | 15500.4 | 15377.8 | 0.1244 | 0.1284 | -0.797 |
| 738 | 12413.3 | 20868.9 | 0.1308 | 0.1226 | 40.518 | 11939.7 | 11984.1 | 0.1281 | 0.1281 | 0.3701 |
| 739 | 12855.8 | 12721.4 | 0.1275 | 0.1282 | -1.057 | 11427.2 | 11465 | 0.1418 | 0.1388 | 0.3295 |
| 740 | 14049.9 | 13991.7 | 0.1251 | 0.1239 | -0.416 | 11422.1 | 11574.6 | 0.1395 | 0.1425 | 1.318 |
| 741 | 11020.4 | 16166.8 | 0.1452 | 0.1433 | 31.833 | 12339 | 12410.5 | 0.1355 | 0.1332 | 0.5762 |
| 742 | 14947.3 | 14738.6 | 0.1351 | 0.1302 | -1.416 | 11473.1 | 11671.9 | 0.1341 | 0.1338 | 1.7034 |
| 743 | 13350 | 13262.6 | 0.1367 | 0.1321 | -0.659 | 10991.3 | 11098.9 | 0.1364 | 0.1374 | 0.9696 |
| 744 | 11761.8 | 17718.9 | 0.13 | 0.1296 | 33.62 | 12585.7 | 12614.6 | 0.1126 | 0.1122 | 0.2299 |
| 745 | 11304.9 | 18613.2 | 0.1326 | 0.1284 | 39.264 | 13983.6 | 14287 | 0.1312 | 0.1333 | 2.1238 |
| 746 | 13510 | 22972.1 | 0.1321 | 0.1312 | 41.19 | 13212 | 13216.1 | 0.1146 | 0.1145 | 0.0313 |
| 747 | 12172.3 | 19573 | 0.1357 | 0.141 | 37.811 | 15180.5 | 15342.2 | 0.124 | 0.1236 | 1.0541 |
| 748 | 13269.6 | 20979.5 | 0.1264 | 0.1329 | 36.749 | 11207.1 | 11171.7 | 0.1339 | 0.1371 | -0.316 |
| 749 | 13537.8 | 13807.7 | 0.1219 | 0.1165 | 1.9548 | 13798.1 | 13924.2 | 0.1298 | 0.1334 | 0.9062 |
| 750 | 12005 | 14366.3 | 0.1408 | 0.1268 | 16.436 | 11385.4 | 11578.2 | 0.1292 | 0.1315 | 1.6648 |
| 751 | 14379.2 | 19321.3 | 0.1343 | 0.1224 | 25.579 | 11318.4 | 11327.7 | 0.1338 | 0.1353 | 0.082 |
| 752 | 12593.1 | 12809.9 | 0.1135 | 0.1121 | 1.6923 | 10714.4 | 10956.1 | 0.1357 | 0.1402 | 2.2065 |
| 753 | 12648.3 | 24779.8 | 0.1123 | 0.1268 | 48.957 | 12100.3 | 12682.6 | 0.1341 | 0.1337 | 4.591 |
| 754 | 12264.3 | 16953.1 | 0.1462 | 0.1424 | 27.658 | 11152 | 11376.9 | 0.1345 | 0.1357 | 1.9767 |
| 755 | 12582.7 | 18388.1 | 0.1258 | 0.1347 | 31.571 | 11863.8 | 11885.3 | 0.1436 | 0.1425 | 0.181 |
| 756 | 13901.9 | 21406.3 | 0.1296 | 0.1278 | 35.057 | 11337 | 15825.5 | 0.1371 | 0.1299 | 28.362 |
| 757 | 14631.9 | 14692.2 | 0.136 | 0.1308 | 0.4103 | 12500.1 | 12715.9 | 0.1239 | 0.1287 | 1.6972 |
| 758 | 12769.3 | 21788.4 | 0.138 | 0.134 | 41.394 | 11784 | 11872.4 | 0.1347 | 0.1369 | 0.7446 |
| 759 | 13155.2 | 17164.2 | 0.1327 | 0.1353 | 23.357 | 16202 | 16217.2 | 0.1268 | 0.1293 | 0.0942 |
| 760 | 10254.7 | 16316.3 | 0.1458 | 0.1429 | 37.151 | 13030.9 | 13494.5 | 0.1347 | 0.1355 | 3.4361 |
| 761 | 11552.1 | 17755.3 | 0.1355 | 0.1377 | 34.938 | 12389.7 | 12634.9 | 0.1363 | 0.1363 | 1.9412 |
| 762 | 11446.4 | 19750.5 | 0.1349 | 0.1377 | 42.045 | 11123 | 11555.8 | 0.1384 | 0.1361 | 3.7452 |
| 763 | 13301 | 25721.3 | 0.1143 | 0.1261 | 48.288 | 14718.1 | 12372.3 | 0.1229 | 0.1176 | -18.96 |
| 764 | 12220.9 | 18990.3 | 0.1315 | 0.1308 | 35.647 | 14237.8 | 14643.2 | 0.1153 | 0.115 | 2.7682 |
| 765 | 11305.6 | 17213.5 | 0.1293 | 0.1331 | 34.321 | 11450.1 | 11872.9 | 0.136 | 0.1407 | 3.5607 |
| 766 | 11189.1 | 17167.2 | 0.1445 | 0.1425 | 34.823 | 11185 | 11321 | 0.1399 | 0.1394 | 1.2011 |
| 767 | 11156.5 | 11313.3 | 0.1335 | 0.1322 | 1.3858 | 12760.9 | 12896.1 | 0.1226 | 0.1228 | 1.0482 |
| 768 | 11924.4 | 12180.2 | 0.1445 | 0.1434 | 2.0999 | 14358 | 14835.5 | 0.1263 | 0.127 | 3.2185 |
| 769 | 11717.8 | 11721.7 | 0.1108 | 0.1108 | 0.0336 | 13162.1 | 13644.6 | 0.1312 | 0.1285 | 3.5363 |
| 770 | 14988.3 | 16469.6 | 0.1341 | 0.1368 | 8.9941 | 14078.5 | 15120 | 0.1277 | 0.1314 | 6.8885 |
| 771 | 12253.3 | 14763.2 | 0.1432 | 0.1393 | 17.001 | 11539.5 | 11717.6 | 0.1343 | 0.1374 | 1.52 |
| 772 | 12069.7 | 16518 | 0.1343 | 0.1343 | 26.93 | 12747.8 | 12752 | 0.1173 | 0.1172 | 0.0325 |
| 773 | 11840.7 | 18929.3 | 0.1356 | 0.141 | 37.448 | 12034.1 | 11978.9 | 0.1411 | 0.14 | -0.46 |
| 774 | 15445.5 | 22365.1 | 0.1178 | 0.1288 | 30.939 | 11353.6 | 11640.5 | 0.1406 | 0.1349 | 2.4647 |
| 775 | 12637.3 | 16168.5 | 0.1251 | 0.1274 | 21.84 | 10938.3 | 10954.1 | 0.1325 | 0.1321 | 0.1448 |
| 776 | 12877.5 | 12810.7 | 0.1174 | 0.1125 | -0.521 | 12089.1 | 12371.4 | 0.1259 | 0.1268 | 2.2825 |
| 777 | 11744.4 | 12698.3 | 0.125 | 0.1296 | 7.5118 | 13432.3 | 13351 | 0.1286 | 0.13 | -0.609 |
| 778 | 15120.4 | 19431.3 | 0.1265 | 0.1309 | 22.185 | 13221.4 | 13396.8 | 0.1133 | 0.1145 | 1.3089 |
| 779 | 13434.2 | 19232.2 | 0.1324 | 0.1281 | 30.147 | 15903.3 | 16359.2 | 0.1303 | 0.1313 | 2.7869 |
| 780 | 12780.2 | 19525.4 | 0.1223 | 0.1206 | 34.546 | 12045.9 | 12096.5 | 0.1285 | 0.1286 | 0.4185 |
| 781 | 13405.5 | 13745.1 | 0.1309 | 0.1333 | 2.4706 | 11778.4 | 11876.2 | 0.1343 | 0.136 | 0.8241 |
| 782 | 15834.4 | 21212.7 | 0.1233 | 0.1299 | 25.354 | 11964 | 11948.2 | 0.1374 | 0.1353 | -0.133 |
| 783 | 12486 | 13919.7 | 0.1217 | 0.1098 | 10.3 | 14253.1 | 14378.4 | 0.1233 | 0.1221 | 0.8718 |
| 784 | 16189.6 | 16512 | 0.1271 | 0.1268 | 1.9527 | 11285.3 | 11894.8 | 0.1362 | 0.1392 | 5.1246 |
| 785 | 11755.8 | 11654.6 | 0.133 | 0.1277 | -0.868 | 13695 | 14576.8 | 0.1354 | 0.1355 | 6.0491 |
| 786 | 14753.5 | 21508.2 | 0.123 | 0.1305 | 31.405 | 11579.6 | 11607.6 | 0.1342 | 0.1362 | 0.2411 |
| 787 | 12256.2 | 12920.2 | 0.1457 | 0.1446 | 5.1393 | 11436.9 | 13716.7 | 0.1353 | 0.1348 | 16.621 |
| 788 | 13757.7 | 19712.5 | 0.1315 | 0.1317 | 30.208 | 12094.3 | 12069.9 | 0.1322 | 0.132 | -0.202 |
| 789 | 11734 | 11998.8 | 0.1376 | 0.1363 | 2.2066 | 11615.9 | 16269 | 0.1346 | 0.1291 | 28.601 |
| 790 | 16027.4 | 20757.9 | 0.123 | 0.1289 | 22.789 | 14068.8 | 14351.5 | 0.1286 | 0.1291 | 1.9694 |
| 791 | 13123.9 | 20512.5 | 0.1192 | 0.1229 | 36.02 | 13804.2 | 13787.8 | 0.1321 | 0.1322 | -0.119 |
| 792 | 12016.9 | 15443.2 | 0.1447 | 0.1421 | 22.187 | 13469.8 | 13445.4 | 0.1318 | 0.1311 | -0.182 |
| 793 | 12689.9 | 13296.1 | 0.1306 | 0.1274 | 4.5594 | 11555.7 | 11943.3 | 0.1274 | 0.1281 | 3.2447 |
| 794 | 11515 | 18903.6 | 0.1252 | 0.135 | 39.086 | 14486.2 | 15140 | 0.1324 | 0.1321 | 4.3184 |
| 795 | 11626.4 | 12230.5 | 0.1312 | 0.1298 | 4.9389 | 13002.2 | 13454 | 0.1296 | 0.1325 | 3.3581 |
| 796 | 13786 | 20920.6 | 0.1286 | 0.1206 | 34.103 | 14016.2 | 14112.2 | 0.1289 | 0.1294 | 0.6807 |
| 797 | 13607.2 | 18743 | 0.1304 | 0.1263 | 27.401 | 11094.9 | 11095.4 | 0.1414 | 0.1401 | 0.0045 |
| 798 | 13158.9 | 20409.3 | 0.1247 | 0.1291 | 35.525 | 12187 | 12923.3 | 0.1448 | 0.1451 | 5.6977 |
| 799 | 12534.8 | 12459.3 | 0.1272 | 0.1223 | -0.606 | 14030.2 | 14057.4 | 0.1279 | 0.1307 | 0.1931 |
| 800 | 12831.7 | 12646.7 | 0.1307 | 0.1379 | -1.463 | 10647 | 10732.2 | 0.1351 | 0.1352 | 0.7945 |
| 801 | 15980.9 | 16133.3 | 0.1276 | 0.1322 | 0.9451 | 12108.1 | 12416.6 | 0.1331 | 0.1314 | 2.4848 |
| 802 | 11530.2 | 12214.4 | 0.1333 | 0.1233 | 5.6017 | 11379.7 | 11486.1 | 0.141 | 0.1429 | 0.9271 |
| 803 | 13013.6 | 19456.5 | 0.1364 | 0.1326 | 33.114 | 14375.7 | 14432.7 | 0.12 | 0.1213 | 0.3948 |
| 804 | 13183.5 | 24082.8 | 0.1273 | 0.1298 | 45.258 | 15391.1 | 15552.8 | 0.1212 | 0.1208 | 1.0398 |
| 805 | 11648.7 | 18569.4 | 0.1331 | 0.1395 | 37.269 | 12734.9 | 12739 | 0.1135 | 0.1134 | 0.0325 |
| 806 | 12309 | 15169.5 | 0.1286 | 0.1311 | 18.857 | 16145.4 | 17339.7 | 0.1278 | 0.1277 | 6.8877 |
| 807 | 11653.4 | 18223.1 | 0.1373 | 0.1411 | 36.051 | 14691.1 | 15555.2 | 0.128 | 0.1276 | 5.5553 |
| 808 | 12260.3 | 12447.7 | 0.1372 | 0.135 | 1.5055 | 12494.1 | 12500.3 | 0.1354 | 0.1341 | 0.0492 |
| 809 | 14522.5 | 14488.7 | 0.1237 | 0.1226 | -0.234 | 15245.5 | 16981.6 | 0.1319 | 0.1324 | 10.224 |
| 810 | 13024.1 | 21380.9 | 0.1288 | 0.1211 | 39.085 | 12466.5 | 13747.7 | 0.1359 | 0.1365 | 9.3194 |
| 811 | 11663.1 | 11767.7 | 0.1326 | 0.1305 | 0.8885 | 12699.1 | 12981.8 | 0.1285 | 0.1293 | 2.1772 |
| 812 | 13370.9 | 13370.7 | 0.1136 | 0.1172 | -0.002 | 13098.6 | 13088.8 | 0.1397 | 0.1386 | -0.075 |
| 813 | 12238 | 15087.5 | 0.1309 | 0.1247 | 18.887 | 15193.7 | 15214.2 | 0.1267 | 0.1263 | 0.1348 |
| 814 | 12329.1 | 19053.1 | 0.1236 | 0.1225 | 35.291 | 11433.4 | 11436.8 | 0.1324 | 0.133 | 0.03 |
| 815 | 14671.9 | 20286.7 | 0.1284 | 0.1314 | 27.677 | 13951.3 | 14028.7 | 0.1123 | 0.1119 | 0.5523 |
| 816 | 13637.3 | 23014.4 | 0.1237 | 0.1327 | 40.744 | 12656.1 | 12755.7 | 0.132 | 0.1299 | 0.7807 |
| 817 | 14567.4 | 14164.9 | 0.1308 | 0.1257 | -2.841 | 13142.3 | 13319.7 | 0.1316 | 0.1324 | 1.3322 |
| 818 | 13768.6 | 18150.1 | 0.1345 | 0.1323 | 24.14 | 13760.1 | 13950.4 | 0.1261 | 0.1291 | 1.3637 |
| 819 | 11214.1 | 16653.8 | 0.1463 | 0.1433 | 32.664 | 14394.8 | 12177.5 | 0.1224 | 0.1174 | -18.21 |
| 820 | 12953.9 | 13759.1 | 0.1262 | 0.1237 | 5.852 | 11634.9 | 12269 | 0.1419 | 0.1369 | 5.1682 |
| 821 | 11692.3 | 11885.5 | 0.1268 | 0.1261 | 1.625 | 11047.4 | 11472.4 | 0.1383 | 0.1349 | 3.7046 |
| 822 | 11131.8 | 18363.9 | 0.1287 | 0.1356 | 39.382 | 12611.3 | 12653.1 | 0.1362 | 0.1358 | 0.3307 |
| 823 | 12059.7 | 15567.8 | 0.1343 | 0.1361 | 22.534 | 12101.6 | 12155.9 | 0.1336 | 0.1331 | 0.4464 |
| 824 | 12404 | 18805.7 | 0.1201 | 0.1242 | 34.041 | 10849.4 | 10770.2 | 0.1427 | 0.1386 | -0.735 |
| 825 | 13604.4 | 21021.7 | 0.1317 | 0.13 | 35.284 | 11727.1 | 12056.1 | 0.1337 | 0.133 | 2.7282 |
| 826 | 12676.8 | 20486.6 | 0.1287 | 0.1301 | 38.122 | 12581.9 | 12395.6 | 0.136 | 0.1347 | -1.503 |
| 827 | 13422.6 | 14265.8 | 0.1106 | 0.1127 | 5.9107 | 12027.3 | 12415.3 | 0.1352 | 0.1319 | 3.1258 |
| 828 | 13333.9 | 21915.7 | 0.1195 | 0.1328 | 39.158 | 11956.1 | 11983.5 | 0.1418 | 0.1411 | 0.2284 |
| 829 | 12334.2 | 18042.6 | 0.1222 | 0.1292 | 31.639 | 11256.5 | 11601.4 | 0.145 | 0.1408 | 2.9728 |
| 830 | 14046.8 | 18990.5 | 0.1318 | 0.1332 | 26.032 | 11788.4 | 11955.1 | 0.1433 | 0.1445 | 1.3943 |
| 831 | 10961.6 | 17757 | 0.1447 | 0.1424 | 38.269 | 11464.7 | 11480.8 | 0.1406 | 0.1402 | 0.1407 |
| 832 | 14588.4 | 14684 | 0.1312 | 0.1302 | 0.6508 | 13042.3 | 13927 | 0.1367 | 0.1369 | 6.3524 |
| 833 | 11856.6 | 11806.1 | 0.111 | 0.1102 | -0.428 | 12368.3 | 12503.4 | 0.127 | 0.1272 | 1.0812 |
| 834 | 15873.8 | 21725.4 | 0.1188 | 0.1296 | 26.934 | 11995.7 | 11938.3 | 0.1407 | 0.1389 | -0.481 |
| 835 | 13672.6 | 14289 | 0.1287 | 0.127 | 4.3135 | 15098.5 | 16103.8 | 0.1227 | 0.123 | 6.243 |
| 836 | 11757.3 | 20155.7 | 0.1309 | 0.1223 | 41.668 | 12614.9 | 12527.7 | 0.1273 | 0.1309 | -0.696 |
| 837 | 14187.9 | 14426.6 | 0.1215 | 0.1167 | 1.6546 | 11502.6 | 11577 | 0.143 | 0.1403 | 0.6422 |
| 838 | 12415.5 | 17969 | 0.1213 | 0.1305 | 30.906 | 11531.8 | 11583.2 | 0.1285 | 0.129 | 0.4439 |
| 839 | 17055 | 17599.4 | 0.1231 | 0.1227 | 3.0935 | 13235.1 | 13517.8 | 0.1296 | 0.1302 | 2.0908 |
| 840 | 11442 | 15632.7 | 0.121 | 0.1244 | 26.808 | 12802.7 | 12859.5 | 0.1351 | 0.1351 | 0.4415 |
| 841 | 12054.2 | 14148.7 | 0.1371 | 0.1241 | 14.803 | 12593.9 | 12644.5 | 0.1296 | 0.1289 | 0.4004 |
| 842 | 15217.3 | 16007.1 | 0.1291 | 0.1256 | 4.934 | 11245.2 | 11482.1 | 0.1314 | 0.1282 | 2.0625 |
| 843 | 13071 | 23461.5 | 0.1122 | 0.1342 | 44.287 | 12587.1 | 12999.1 | 0.1236 | 0.1243 | 3.1694 |
| 844 | 11478.2 | 11851.9 | 0.1359 | 0.1305 | 3.1529 | 12171 | 12228 | 0.1263 | 0.1278 | 0.466 |
| 845 | 14391 | 14854.9 | 0.1234 | 0.1221 | 3.1231 | 16089.7 | 16119 | 0.1276 | 0.1272 | 0.1817 |
| 846 | 11756.9 | 18237.6 | 0.1312 | 0.1277 | 35.535 | 11642 | 12146.1 | 0.1317 | 0.1314 | 4.1502 |
| 847 | 13655.4 | 14667.4 | 0.1236 | 0.131 | 6.8996 | 13120.3 | 13229.2 | 0.1276 | 0.1301 | 0.8232 |
| 848 | 14655.1 | 18720.2 | 0.1285 | 0.1307 | 21.715 | 13546.1 | 13649.4 | 0.1279 | 0.132 | 0.7565 |
| 849 | 12812.3 | 12952.2 | 0.1128 | 0.1131 | 1.0802 | 11796.5 | 12036.8 | 0.146 | 0.1458 | 1.9966 |
| 850 | 14024.1 | 25107.7 | 0.1119 | 0.1303 | 44.144 | 13308.5 | 13379.7 | 0.1276 | 0.1239 | 0.5322 |
| 851 | 14005.4 | 13536.6 | 0.1166 | 0.1233 | -3.463 | 13605.2 | 14085.9 | 0.1228 | 0.1238 | 3.4126 |
| 852 | 10769.6 | 14193.8 | 0.1379 | 0.136 | 24.125 | 9967.34 | 10312.5 | 0.1449 | 0.1436 | 3.3472 |
| 853 | 11363.7 | 14974.6 | 0.1469 | 0.1424 | 24.113 | 15712.9 | 16576.2 | 0.1223 | 0.1212 | 5.2081 |
| 854 | 14417.9 | 14501 | 0.1348 | 0.1335 | 0.5733 | 14517.7 | 14427.6 | 0.1269 | 0.1308 | -0.624 |
| 855 | 11172.7 | 11043.7 | 0.1202 | 0.1137 | -1.168 | 13500.7 | 13557.7 | 0.1243 | 0.1257 | 0.4203 |
| 856 | 13449.9 | 15107.2 | 0.122 | 0.1336 | 10.97 | 11847.7 | 12127.2 | 0.1383 | 0.1446 | 2.3047 |
| 857 | 12174.8 | 12244.5 | 0.1105 | 0.1169 | 0.5693 | 10601.1 | 10922.6 | 0.1372 | 0.1331 | 2.943 |
| 858 | 11420.2 | 15723.7 | 0.1306 | 0.1324 | 27.37 | 11995.9 | 12185.8 | 0.1279 | 0.1361 | 1.5581 |
| 859 | 12040.9 | 12179.4 | 0.1392 | 0.1407 | 1.1371 | 10728.3 | 11119.9 | 0.1367 | 0.1367 | 3.5214 |
| 860 | 11855 | 20788.9 | 0.1369 | 0.1327 | 42.974 | 14617.6 | 14975.9 | 0.1282 | 0.1306 | 2.3924 |
| 861 | 13678.3 | 14234.9 | 0.1308 | 0.1317 | 3.9099 | 10773.9 | 11132.1 | 0.1325 | 0.1307 | 3.218 |
| 862 | 13200.2 | 22420.5 | 0.1192 | 0.133 | 41.124 | 12918.2 | 16474.1 | 0.1293 | 0.1249 | 21.585 |
| 863 | 12741.2 | 23770.8 | 0.1247 | 0.1291 | 46.4 | 14172.9 | 14428.1 | 0.132 | 0.1327 | 1.7684 |
| 864 | 11655.2 | 14426.5 | 0.1293 | 0.1316 | 19.21 | 12194.4 | 12198.8 | 0.132 | 0.1305 | 0.0357 |
| 865 | 12293.6 | 12513.9 | 0.1347 | 0.1384 | 1.7607 | 14033.6 | 14477.8 | 0.1307 | 0.1319 | 3.0681 |
| 866 | 12042.1 | 15936.7 | 0.1469 | 0.1417 | 24.438 | 13688.1 | 14787.7 | 0.1272 | 0.1318 | 7.4355 |
| 867 | 10962.4 | 17232.9 | 0.1293 | 0.1364 | 36.387 | 12433.5 | 12433.5 | 0.1137 | 0.1137 | 0 |
| 868 | 12898 | 13253 | 0.1132 | 0.1119 | 2.6788 | 12235.5 | 12456.8 | 0.1386 | 0.1357 | 1.7768 |
| 869 | 14385.8 | 20684.2 | 0.1277 | 0.1321 | 30.45 | 11233.9 | 11330.2 | 0.1449 | 0.1418 | 0.8497 |
| 870 | 11625.8 | 11879.1 | 0.1296 | 0.134 | 2.132 | 11590.8 | 11984 | 0.1331 | 0.1325 | 3.2818 |
| 871 | 11157.1 | 18677.2 | 0.1376 | 0.1399 | 40.263 | 10852.7 | 10951.5 | 0.144 | 0.1424 | 0.902 |
| 872 | 13413 | 24483 | 0.1303 | 0.132 | 45.215 | 13252.7 | 13410.6 | 0.1239 | 0.1286 | 1.1768 |
| 873 | 11999.7 | 18223.9 | 0.1232 | 0.1283 | 34.154 | 11723 | 11735.5 | 0.1379 | 0.1366 | 0.1066 |
| 874 | 12404.8 | 19482.5 | 0.1252 | 0.1359 | 36.328 | 12651.5 | 12974.6 | 0.1333 | 0.1331 | 2.4896 |
| 875 | 11781.3 | 15242.5 | 0.1463 | 0.1432 | 22.707 | 11264.9 | 11225.4 | 0.1308 | 0.1337 | -0.352 |
| 876 | 13405.2 | 13979.1 | 0.1326 | 0.1324 | 4.1051 | 12127.9 | 12346.9 | 0.1311 | 0.1288 | 1.774 |
| 877 | 14734.4 | 20712.8 | 0.1203 | 0.1329 | 28.863 | 11012.8 | 11011 | 0.1358 | 0.1356 | -0.016 |
| 878 | 11424.9 | 11416.3 | 0.1328 | 0.1362 | -0.075 | 11006.5 | 11033.9 | 0.1435 | 0.1424 | 0.248 |
| 879 | 13336.8 | 15964.9 | 0.1389 | 0.1393 | 16.461 | 14537.8 | 15566.7 | 0.1275 | 0.1305 | 6.6097 |
| 880 | 12758.4 | 15048.4 | 0.1416 | 0.1384 | 15.218 | 13226.9 | 13350.1 | 0.1372 | 0.1382 | 0.9233 |
| 881 | 16097.3 | 17295.4 | 0.1272 | 0.1202 | 6.9273 | 13601.3 | 13651.9 | 0.1302 | 0.13 | 0.3708 |
| 882 | 12341.2 | 12407.6 | 0.1203 | 0.1151 | 0.5346 | 11199.7 | 11471.8 | 0.1346 | 0.1381 | 2.3725 |
| 883 | 12805.1 | 19317.8 | 0.1206 | 0.1235 | 33.714 | 12109.5 | 12161.2 | 0.1305 | 0.1305 | 0.4253 |
| 884 | 14336.2 | 15977.9 | 0.1325 | 0.1312 | 10.275 | 11949.6 | 12171.9 | 0.1313 | 0.1327 | 1.826 |
| 885 | 16172.3 | 16494.7 | 0.1256 | 0.1253 | 1.9547 | 12956.4 | 12973 | 0.1125 | 0.1124 | 0.1277 |
| 886 | 12065.8 | 12546.2 | 0.1315 | 0.1258 | 3.8288 | 10905.3 | 11074 | 0.1429 | 0.1429 | 1.5234 |
| 887 | 14720.2 | 16468.1 | 0.1254 | 0.1301 | 10.614 | 12590.6 | 12924.8 | 0.1317 | 0.1407 | 2.5859 |
| 888 | 12320.8 | 18662.7 | 0.127 | 0.1366 | 33.982 | 12133.2 | 12316.1 | 0.1459 | 0.1443 | 1.4846 |
| 889 | 15024.7 | 17342.2 | 0.1256 | 0.1287 | 13.363 | 11821.4 | 11912.7 | 0.1353 | 0.1371 | 0.7667 |
| 890 | 13615.7 | 23015.9 | 0.1273 | 0.1341 | 40.843 | 11805.6 | 11910 | 0.1334 | 0.1388 | 0.8765 |
| 891 | 11862.5 | 18539.7 | 0.1435 | 0.1416 | 36.015 | 12079 | 12221.6 | 0.1472 | 0.1462 | 1.1671 |
| 892 | 12014.4 | 13386.3 | 0.1212 | 0.1317 | 10.249 | 10521.2 | 10798 | 0.1438 | 0.1416 | 2.5636 |
| 893 | 11829.8 | 20065.3 | 0.1345 | 0.1362 | 41.044 | 11047.9 | 11251.6 | 0.1283 | 0.1273 | 1.8102 |
| 894 | 11627.5 | 12029.6 | 0.1314 | 0.1335 | 3.3431 | 13830 | 13859.3 | 0.1316 | 0.1311 | 0.2113 |
| 895 | 14466.7 | 14698 | 0.1226 | 0.1145 | 1.5733 | 12247.3 | 16133.8 | 0.1381 | 0.1304 | 24.089 |
| 896 | 13824.9 | 14232.2 | 0.1297 | 0.1337 | 2.8617 | 13593 | 14290.7 | 0.1319 | 0.1312 | 4.8819 |
| 897 | 13157.2 | 23926.4 | 0.1142 | 0.1297 | 45.01 | 11456.1 | 11688.1 | 0.1357 | 0.1369 | 1.9846 |
| 898 | 12283.2 | 15948.1 | 0.1381 | 0.1261 | 22.98 | 11542.7 | 11784.5 | 0.1272 | 0.1331 | 2.0525 |
| 899 | 12822.1 | 13416.7 | 0.1456 | 0.1471 | 4.4317 | 16872.1 | 16901.3 | 0.1283 | 0.1277 | 0.1733 |
[truncated: 10,141 more chars]
